# Author Correction: Automated Gleason grading of prostate cancer tissue microarrays via deep learning

**DOI:** 10.1038/s41598-019-43989-8

**Published:** 2019-05-16

**Authors:** Eirini Arvaniti, Kim S. Fricker, Michael Moret, Niels Rupp, Thomas Hermanns, Christian Fankhauser, Norbert Wey, Peter J. Wild, Jan H. Rüschoff, Manfred Claassen

**Affiliations:** 1 Institute for Molecular Systems Biology, ETH Zurich, Zurich, Switzerland; 2 Department of Pathology and Molecular Pathology, University of Zurich, Zurich, Switzerland; 3 Department of Urology, University of Zurich, Zurich, Switzerland; 4 Dr. Senckenberg Institute of Pathology, University Hospital Frankfurt, Frankfurt, Germany; 5 Swiss Institute of Bioinformatics (SIB), Zurich, Switzerland

Correction to: *Scientific Reports* 10.1038/s41598-018-30535-1, published online 13 August 2018

This Article contains an error in the Discussion section.

“The low-risk and intermediate-risk groups defined by the model’s predictions were more significantly separated compared to the sfirst study where deep learning-based predictions are used for survival analysis in a prostate cancer cohort.”

should read:

“The low-risk and intermediate-risk groups defined by the model’s predictions were more significantly separated compared to the corresponding groups defined by either pathologist’s annotations. To our knowledge, this is the first study where deep learning-based predictions are used for survival analysis in a prostate cancer cohort.”

