# Author Correction: Automated Gleason grading of prostate cancer tissue microarrays via deep learning

**DOI:** 10.1038/s41598-021-02195-1

**Published:** 2021-11-23

**Authors:** Eirini Arvaniti, Kim S. Fricker, Michael Moret, Niels Rupp, Thomas Hermanns, Christian Fankhauser, Norbert Wey, Peter J. Wild, Jan H. Rüschoff, Manfred Claassen

**Affiliations:** 1 Institute for Molecular Systems Biology, ETH Zurich, Zurich, Switzerland; 2 Department of Pathology and Molecular Pathology, University of Zurich, Zurich, Switzerland; 3 Department of Urology, University of Zurich, Zurich, Switzerland; 4 Dr. Senckenberg Institute of Pathology, University Hospital Frankfurt, Frankfurt, Germany; 5 Swiss Institute of Bioinformatics (SIB), Zurich, Switzerland

Correction to: *Scientific Reports* 10.1038/s41598-018-30535-1, published online 13 August 2018

In the original version of this Article Jan H. Rüschoff and Manfred Claassen figured as co-corresponding authors. This status implied their role as jointly supervising authors. This role is now explicitly indicated by specifying “Jan H. Rüschoff and Manfred Claassen jointly supervised this work”.

The original Article and accompanying Supplementary Information file have been corrected.

