# Supplementary material for: Automated Gleason grading of prostate cancer tissue microarrays via deep learning
Source: Sci Rep. 2018 Aug 13;8:12054. doi: 10.1038/s41598-018-30535-1 (PMC6089889; doi:10.1038/s41598-018-30535-1)
Supplement: Supplementary file 1 — Supplementary Information [file 41598_2018_30535_MOESM1_ESM.pdf]

Supplementary Information for the manuscript:

Automated Gleason grading of prostate cancer tissue microarrays via deep learning

Eirini Arvaniti[1,5,†], Kim S. Fricker[2,†], Michael Moret[1], Niels Rupp[2], Thomas Hermanns[3], Christian Fankhauser[3], Norbert Wey[2], Peter J. Wild[2,4], Jan H. Rüschoff[2,*,#] & Manfred Claassen[1,5,*,#]

[1]Institute for Molecular Systems Biology, ETH Zurich, Switzerland
[2]Department of Pathology and Molecular Pathology, University of Zurich, Switzerland
[3]Department of Urology, University of Zurich, Switzerland
[4]Dr. Senckenberg Institute of Pathology, University Hospital Frankfurt, Germany
[5]Swiss Institute of Bioinformatics (SIB), Switzerland

[†] Co-first authors

[*] Corresponding authors

[#]These authors jointly supervised this work

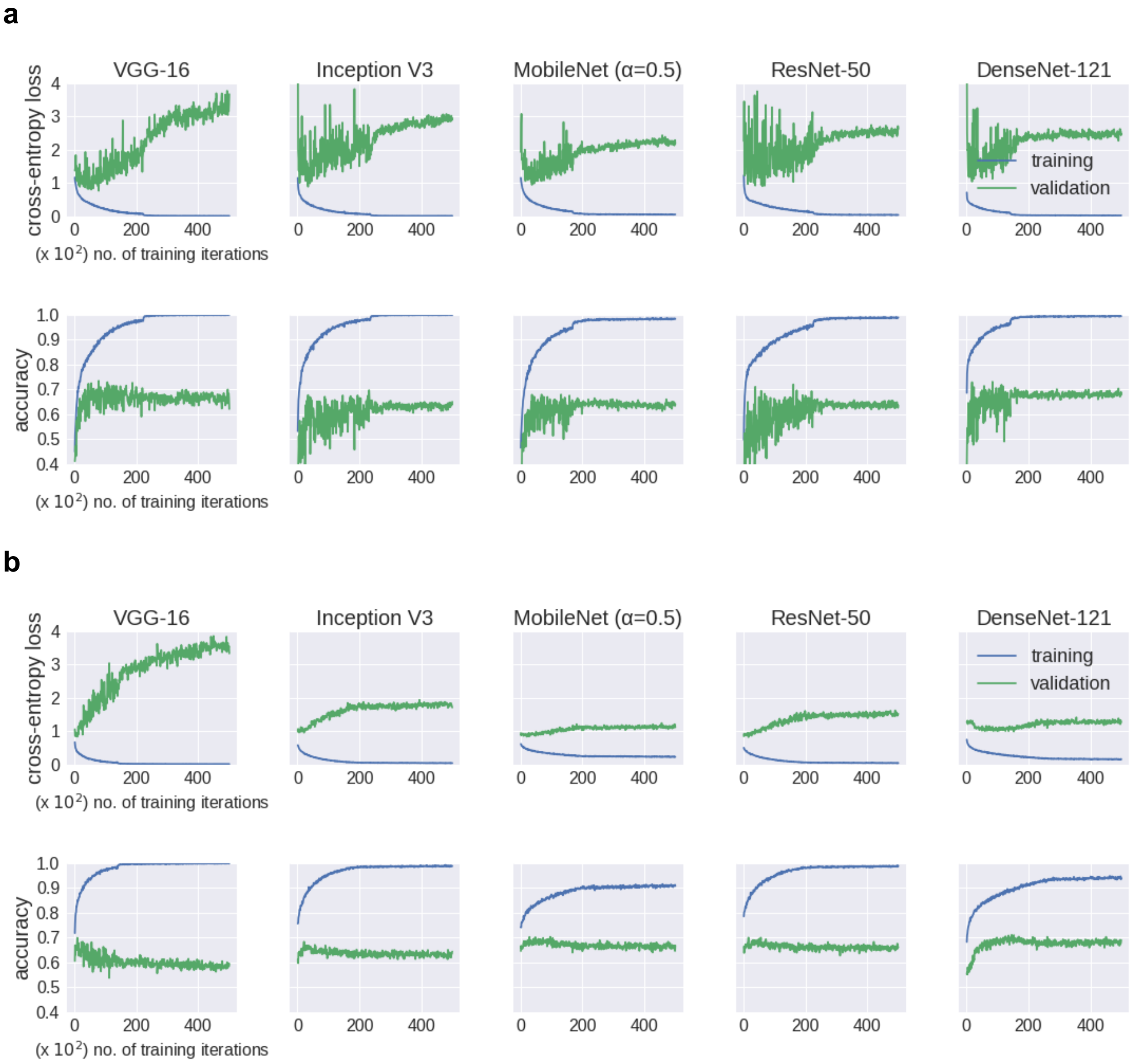

Supplementary Figure S1: Model selection benchmark. Cross-entropy loss and accuracy over training iterations for model variants **(a)** trained from scratch, and **(b)** initialized with ImageNet pre-trained weights. Training and validation mini-batches were balanced with respect to Gleason class labels.

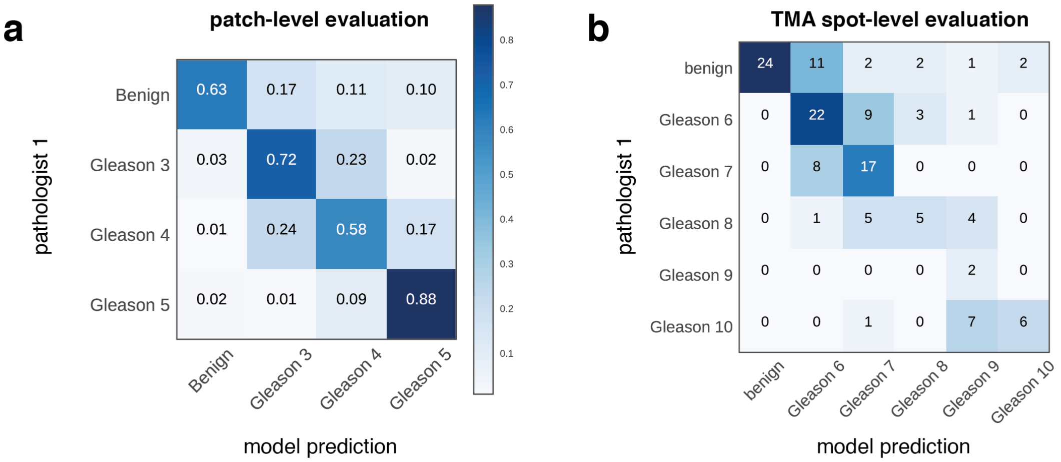

Supplementary Figure S2: Model evaluation on validation cohort. **(a)** Confusion matrix of patch-based model predictions versus pathologist annotations. The confusion matrix is normalized per row (ground truth label) reflecting the recall metric per class. **(b)** Gleason score model predictions for each TMA spot compared with Gleason score assignments by the pathologist. This confusion matrix is not normalized.

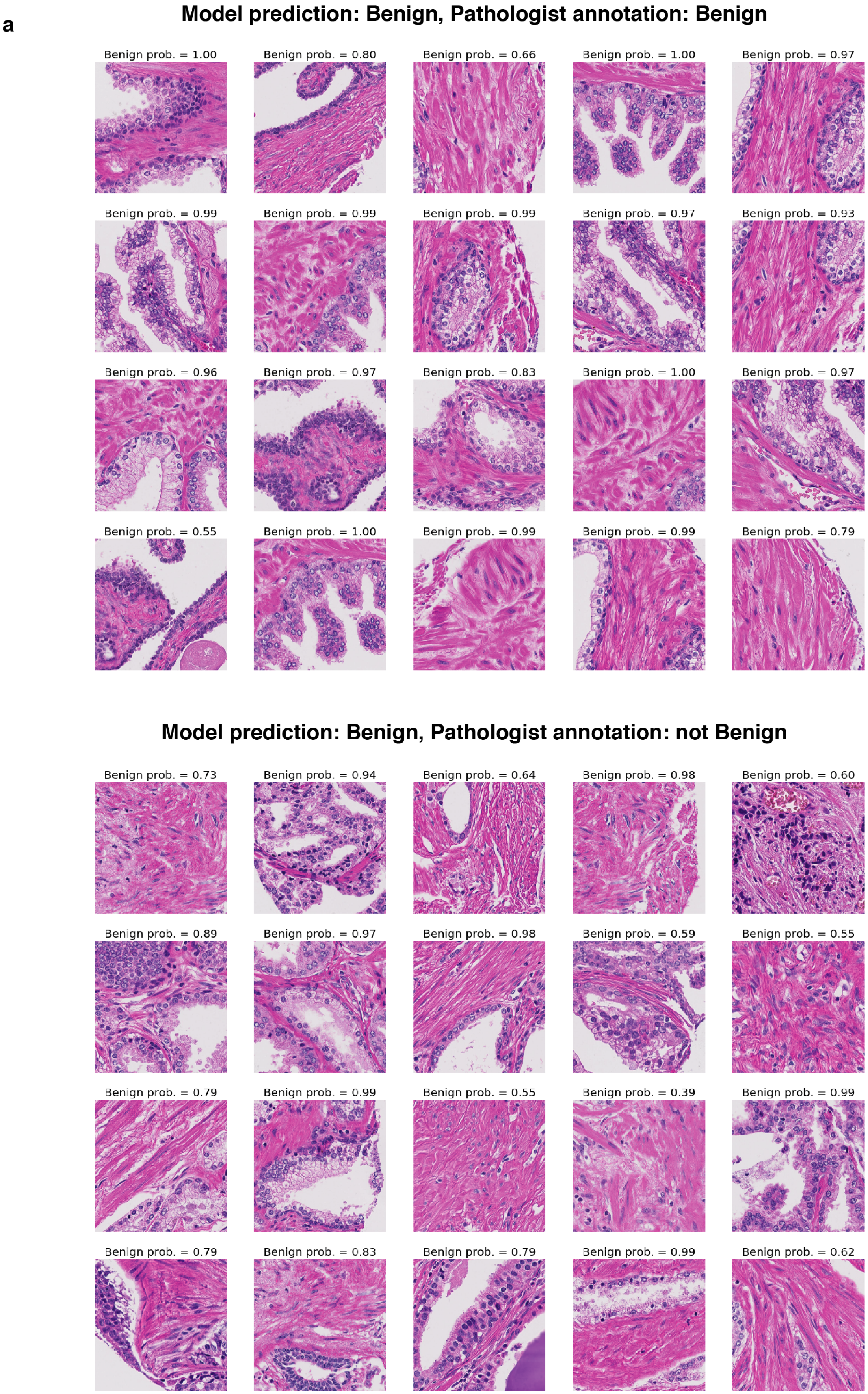
a
Model prediction: Benign, Pathologist annotation: Benign
Benign prob. = 1.00
Benign prob. = 0.80
Benign prob. = 0.66
Benign prob. = 1.00
Benign prob. = 0.97
Benign prob. = 0.99
Benign prob. = 0.99
Benign prob. = 0.99
Benign prob. = 0.97
Benign prob. = 0.93
Benign prob. = 0.96
Benign prob. = 0.97
Benign prob. = 0.83
Benign prob. = 1.00
Benign prob. = 0.97
Benign prob. = 0.55
Benign prob. = 1.00
Benign prob. = 0.99
Benign prob. = 0.99
Benign prob. = 0.79
Model prediction: Benign, Pathologist annotation: not Benign
Benign prob. = 0.73
Benign prob. = 0.94
Benign prob. = 0.64
Benign prob. = 0.98
Benign prob. = 0.60
Benign prob. = 0.89
Benign prob. = 0.97
Benign prob. = 0.98
Benign prob. = 0.59
Benign prob. = 0.55
Benign prob. = 0.79
Benign prob. = 0.99
Benign prob. = 0.55
Benign prob. = 0.39
Benign prob. = 0.99
Benign prob. = 0.79
Benign prob. = 0.83
Benign prob. = 0.79
Benign prob. = 0.99
Benign prob. = 0.62

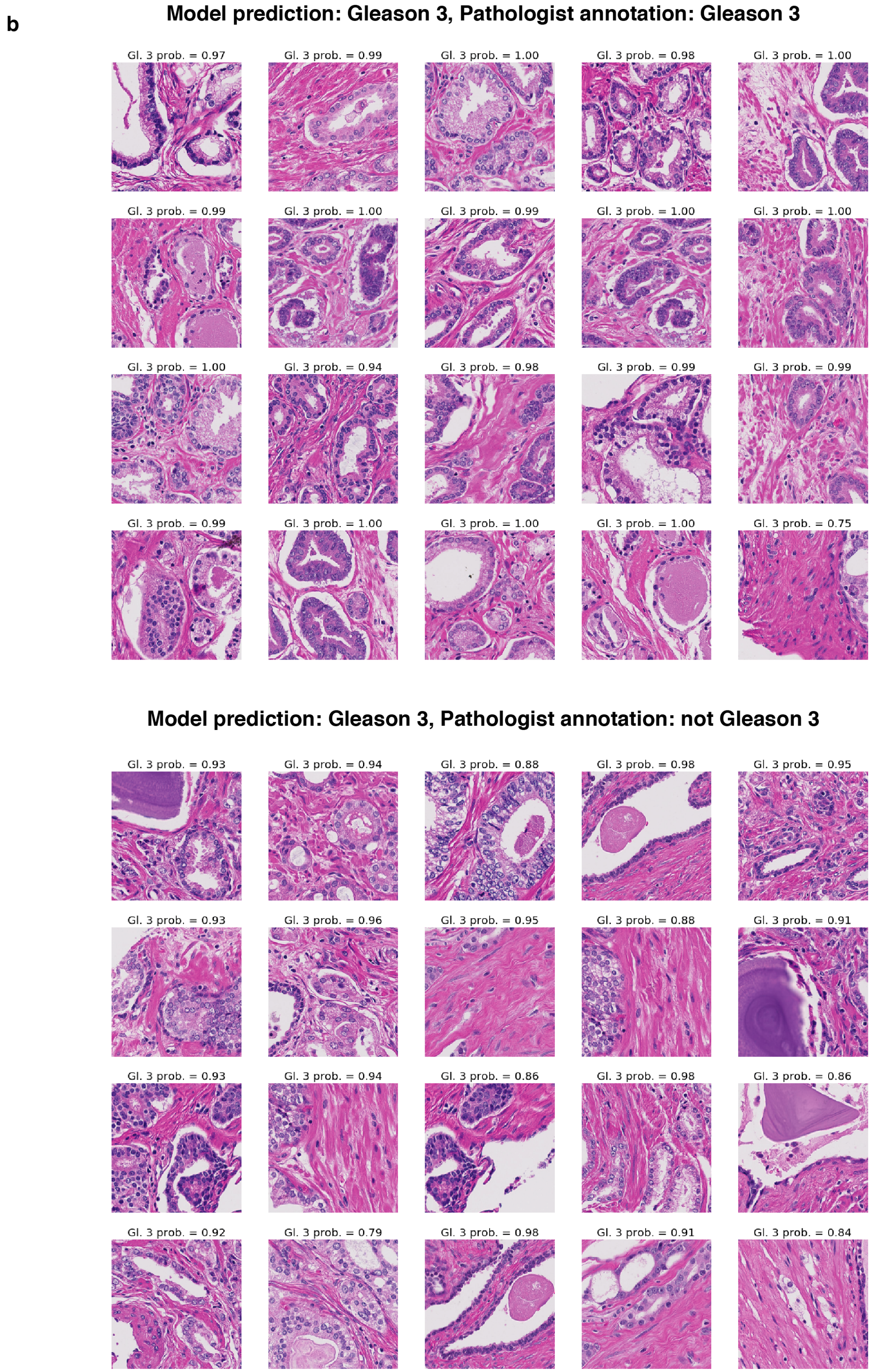
b
Model prediction: Gleason 3, Pathologist annotation: Gleason 3
Gl. 3 prob. = 0.97
Gl. 3 prob. = 0.99
Gl. 3 prob. = 1.00
Gl. 3 prob. = 0.98
Gl. 3 prob. = 1.00
Gl. 3 prob. = 0.99
Gl. 3 prob. = 1.00
Gl. 3 prob. = 0.99
Gl. 3 prob. = 1.00
Gl. 3 prob. = 1.00
Gl. 3 prob. = 1.00
Gl. 3 prob. = 0.94
Gl. 3 prob. = 0.98
Gl. 3 prob. = 0.99
Gl. 3 prob. = 0.99
Gl. 3 prob. = 0.99
Gl. 3 prob. = 1.00
Gl. 3 prob. = 1.00
Gl. 3 prob. = 1.00
Gl. 3 prob. = 0.75
Model prediction: Gleason 3, Pathologist annotation: not Gleason 3
Gl. 3 prob. = 0.93
Gl. 3 prob. = 0.94
Gl. 3 prob. = 0.88
Gl. 3 prob. = 0.98
Gl. 3 prob. = 0.95
Gl. 3 prob. = 0.93
Gl. 3 prob. = 0.96
Gl. 3 prob. = 0.95
Gl. 3 prob. = 0.88
Gl. 3 prob. = 0.91
Gl. 3 prob. = 0.93
Gl. 3 prob. = 0.94
Gl. 3 prob. = 0.86
Gl. 3 prob. = 0.98
Gl. 3 prob. = 0.86
Gl. 3 prob. = 0.92
Gl. 3 prob. = 0.79
Gl. 3 prob. = 0.98
Gl. 3 prob. = 0.91
Gl. 3 prob. = 0.84

c

Model prediction: Gleason 4, Pathologist annotation: Gleason 4

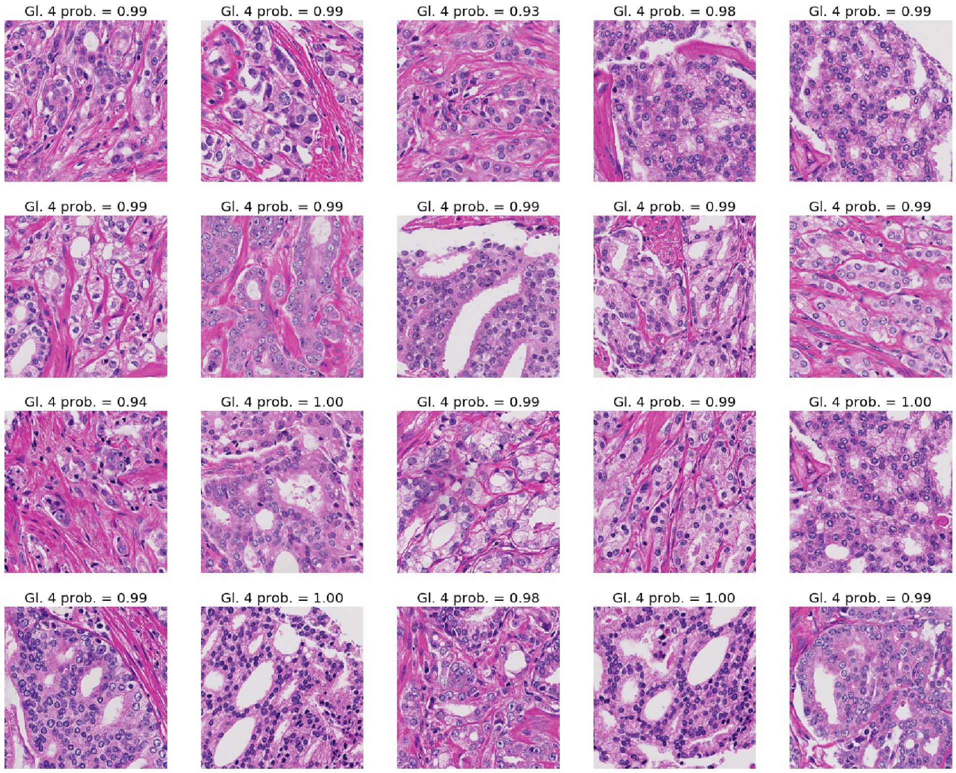

Model prediction: Gleason 4, Pathologist annotation: not Gleason 4

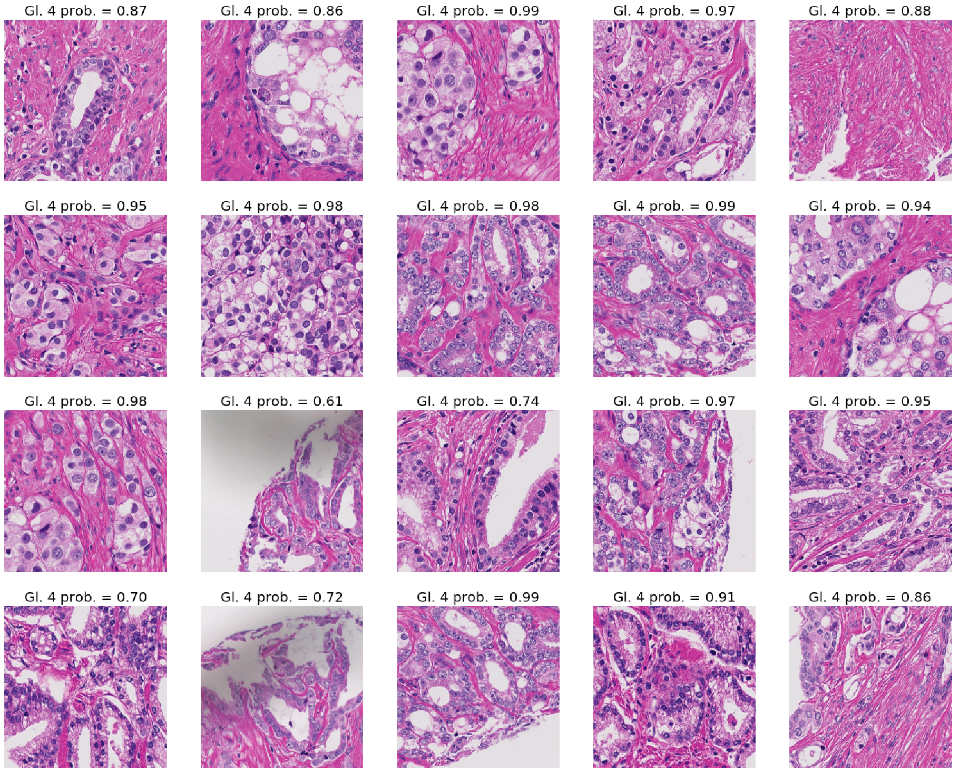

d

Model prediction: Gleason 5, Pathologist annotation: Gleason 5

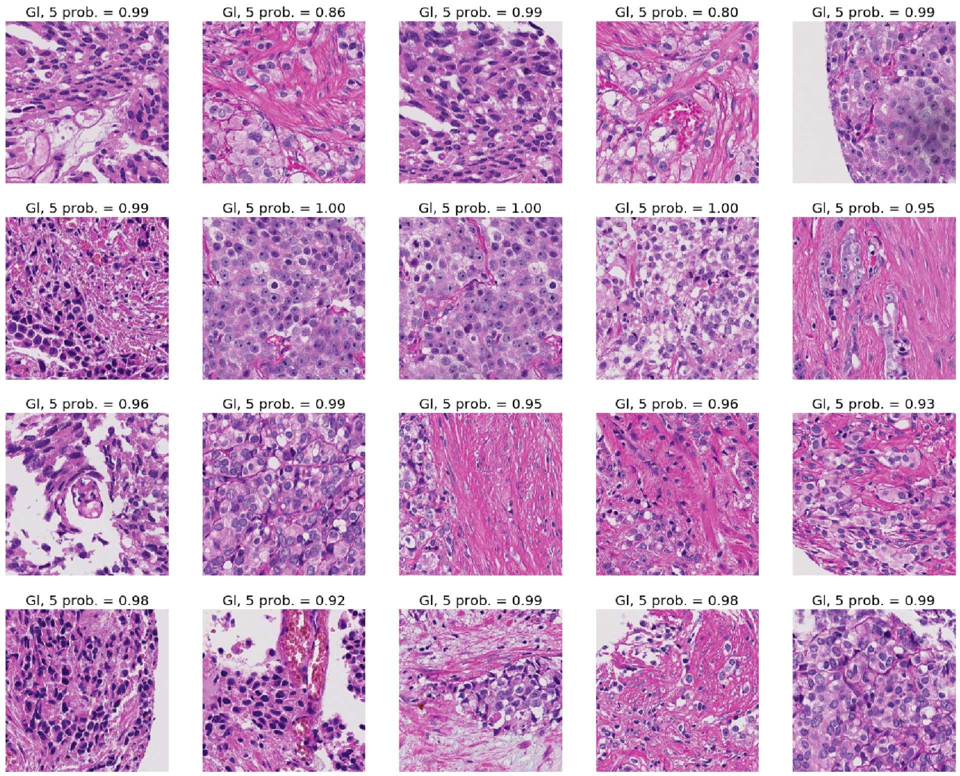

Model prediction: Gleason 5, Pathologist annotation: not Gleason 5

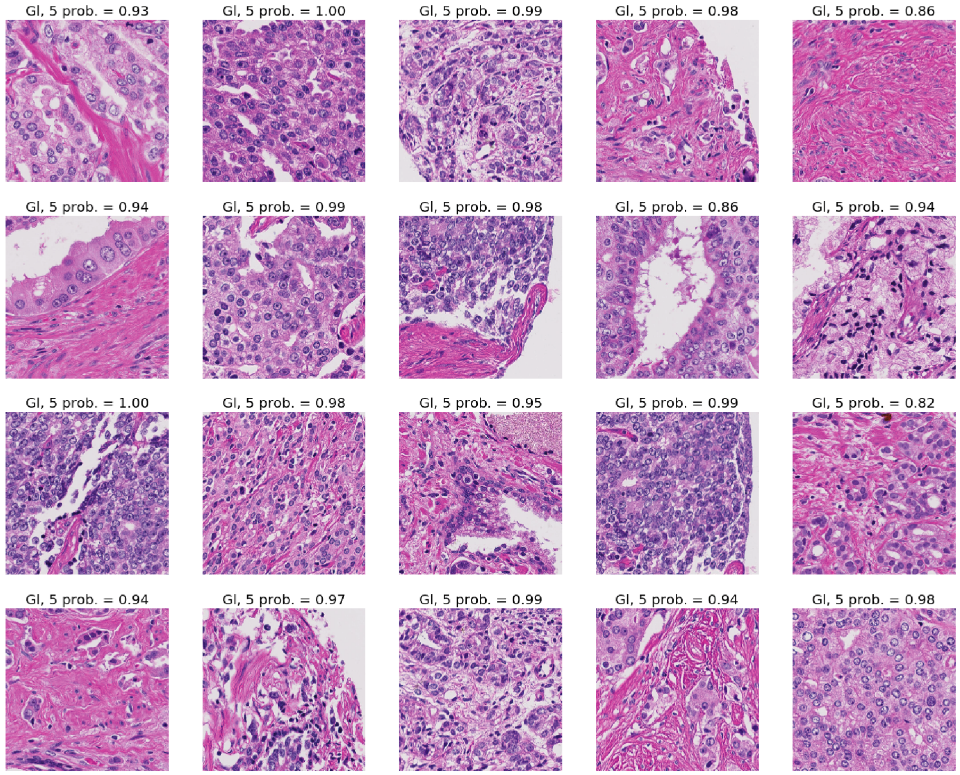

Supplementary Figure S3: Comparison of misclassified versus correctly classified small tissue regions (image patches), corresponding to **Figure 2d**. Patches predicted by the model as **(a)** Benign, **(b)** Gleason 3, **(c)** Gleason 4, **(d)** Gleason 5. For each class, we show both example patches for which the model agrees with both pathologists and example patches for which the model does not agree with either pathologist. In all cases, 20 clusters of image patches were obtained via k-means and, from each cluster, the patch with highest predicted probability for the corresponding class was visualized.

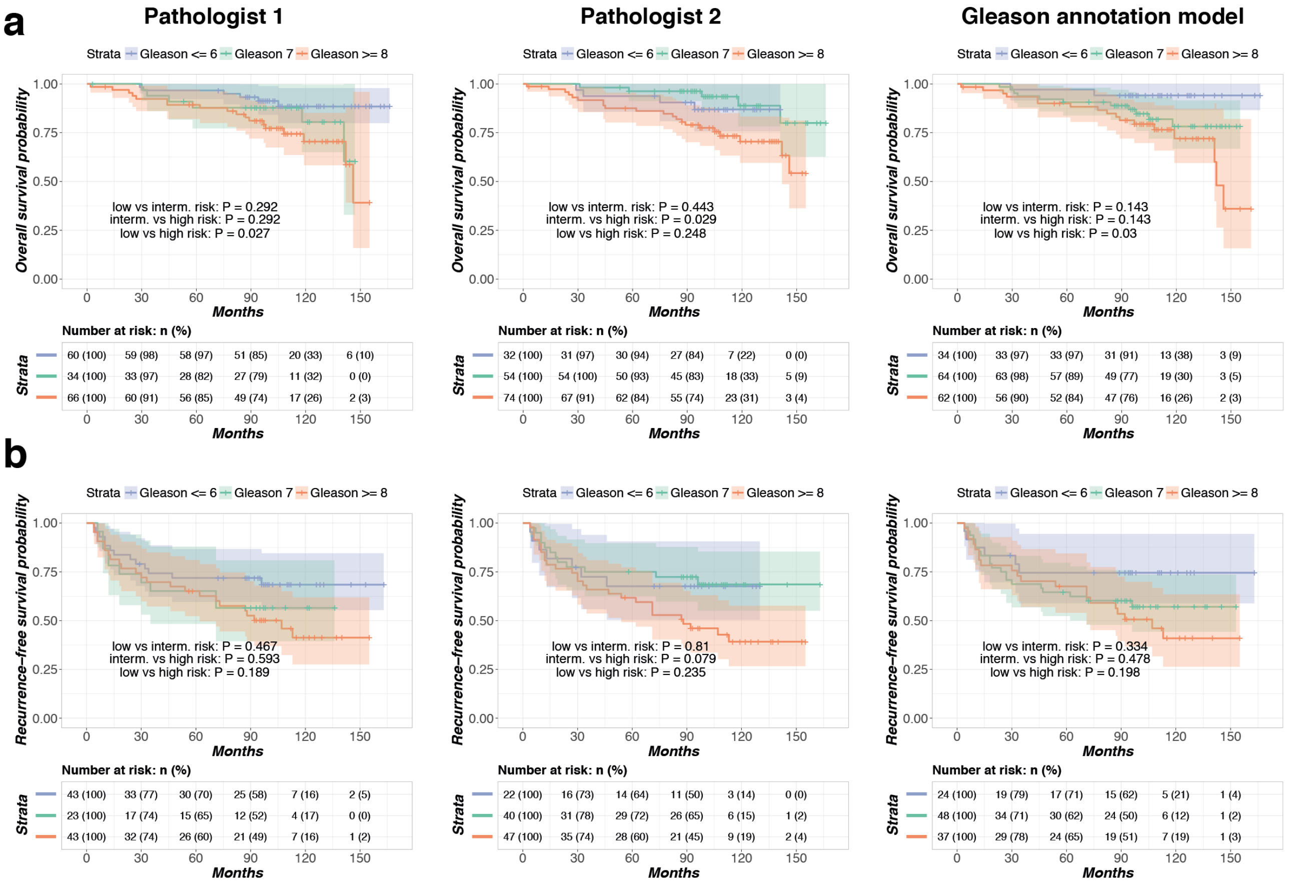

Supplementary Figure S4: (a) Overall survival and **(b)** recurrence-free survival analysis results. Kaplan-Meier curves for patients who were split into three risk groups according to Gleason score annotations by the model and two pathologists. The shaded regions indicate 95% confidence bands. P-values for pairwise two-tailed logrank tests with Benjamini-Hochberg correction are reported.

Supplementary Table S1: Univariate Cox proportional hazards regression results for patients who were split into three risk groups (Gleason ≤ 6, Gleason 7, Gleason ≥ 8) according to Gleason score annotations by the model and two pathologists. Hazard ratios for 1-unit increase in the explanatory variable (risk group 1, 2 or 3) are reported.

	**Overall survival**		**Disease-specific survival**		**Recurrence-free survival**	
	**Hazard Ratio (95% CI)**	**p-value**	**Hazard Ratio (95% CI)**	**p-value**	**Hazard Ratio (95% CI)**	**p-value**
**Model**	2.063 (1.192-3.571)	0.0097	3.048 (1.319-7.042)	0.0091	1.441 (0.962-2.159)	0.0763
**Pathologist 1**	1.784 (1.145- 2.781)	0.0106	2.164 (1.141-4.106)	0.0181	1.361 (0.976-1.899)	0.0694
**Pathologist 2**	1.831 (1.037-3.231)	0.0369	2.79 (1.150-6.770)	0.0233	1.508 (0.990-2.298)	0.0556

Supplementary Data

Model predictions as pixel-level probability maps and visual comparison with pathologist annotations for all TMA spots in the test cohort, corresponding to Figure 3. For each example**,** the subplots in the right-most column show the Gleason patterns assigned by the two pathologists (blue: Gleason 3 region, yellow: Gleason 4 region, red: Gleason 5 region). The other four subplots show the model's Gleason annotations.

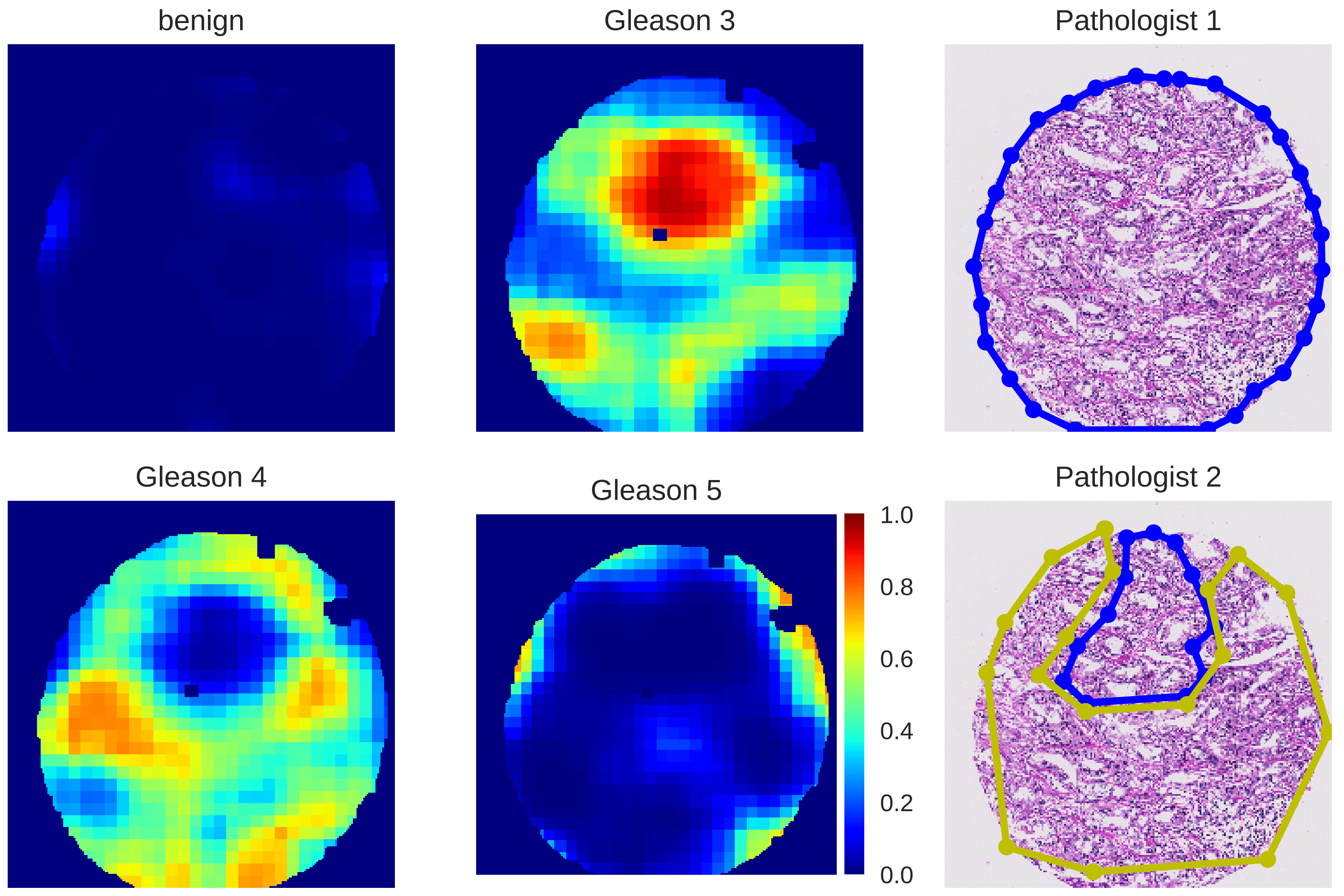

benign
Gleason 3
Pathologist 1
Gleason 4
Gleason 5
Pathologist 2
1.0
0.8
0.6
0.4
0.2
0.0

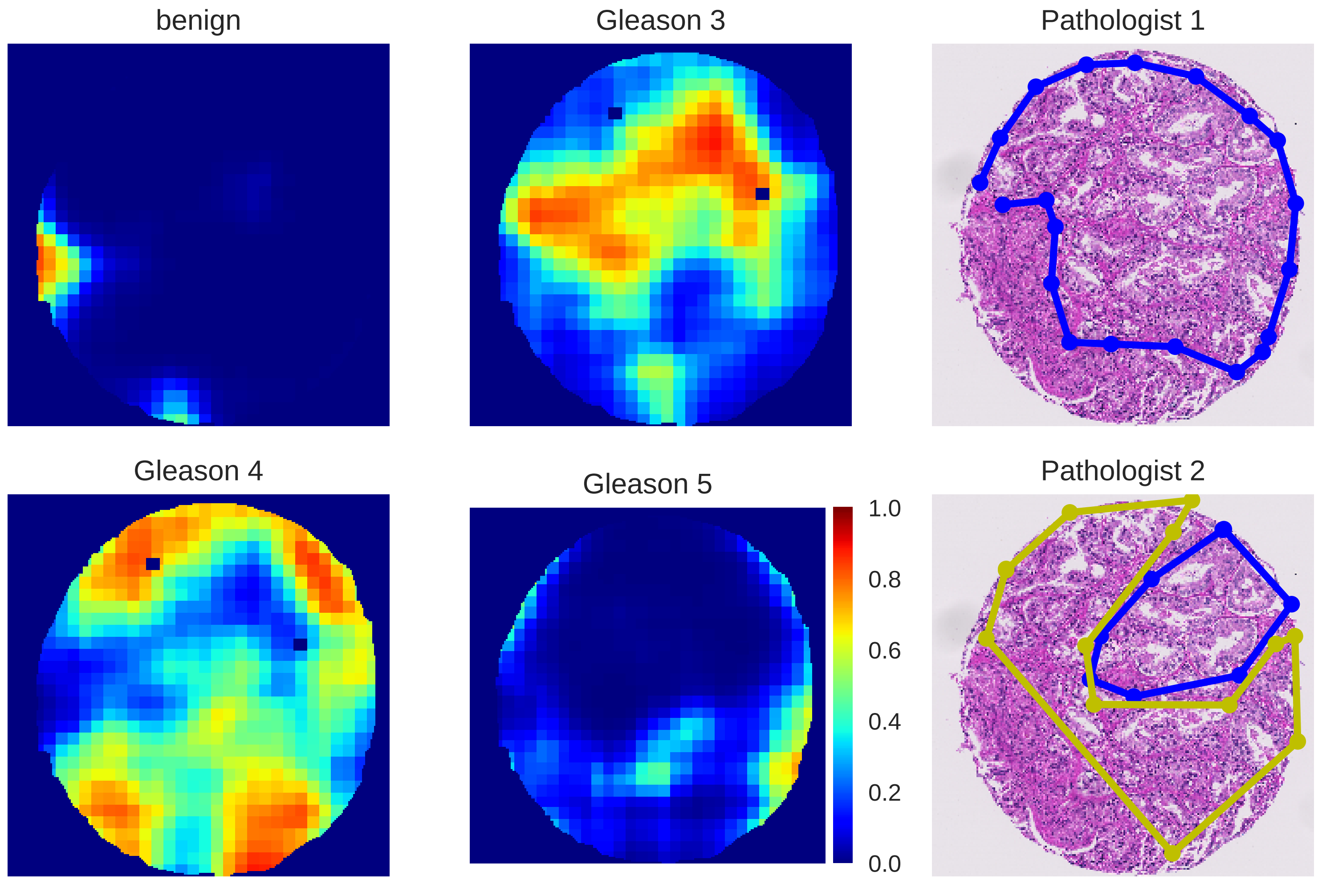
benign
Gleason 3
Pathologist 1
Gleason 4
Gleason 5
Pathologist 2
1.0
0.8
0.6
0.4
0.2
0.0

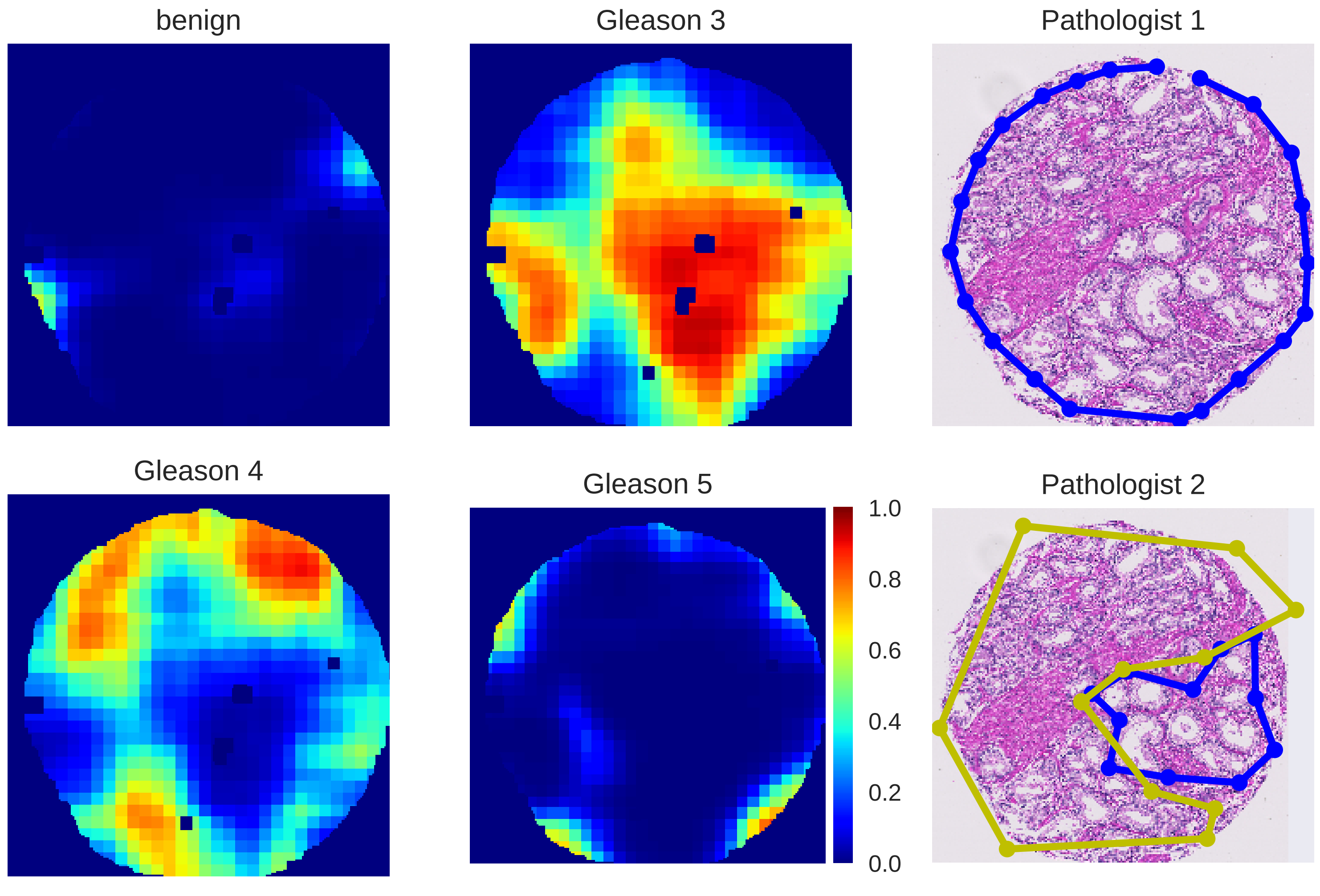
benign
Gleason 3
Pathologist 1
Gleason 4
Gleason 5
Pathologist 2
1.0
0.8
0.6
0.4
0.2
0.0

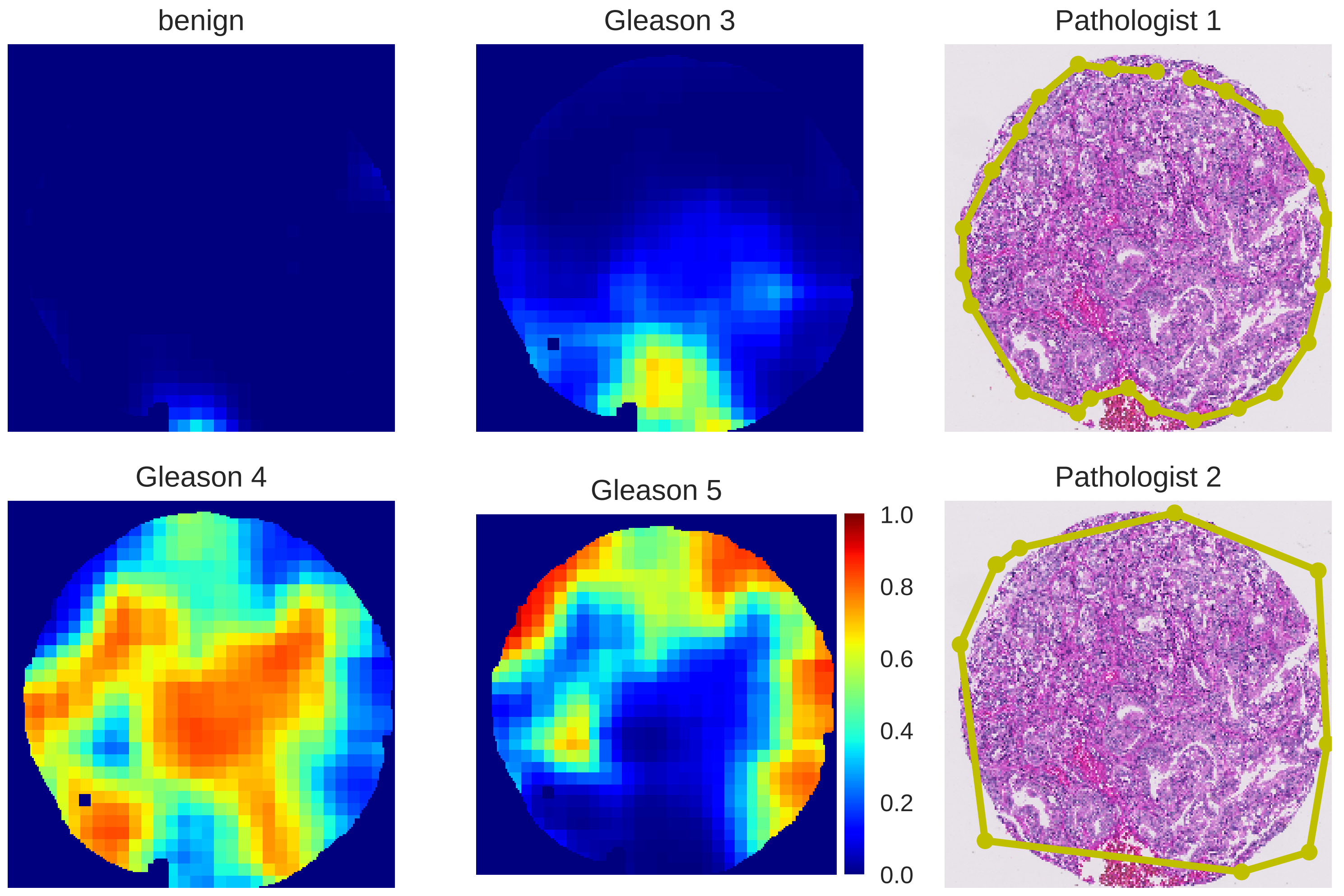
benign
Gleason 3
Pathologist 1
Gleason 4
Gleason 5
Pathologist 2
1.0
0.8
0.6
0.4
0.2
0.0

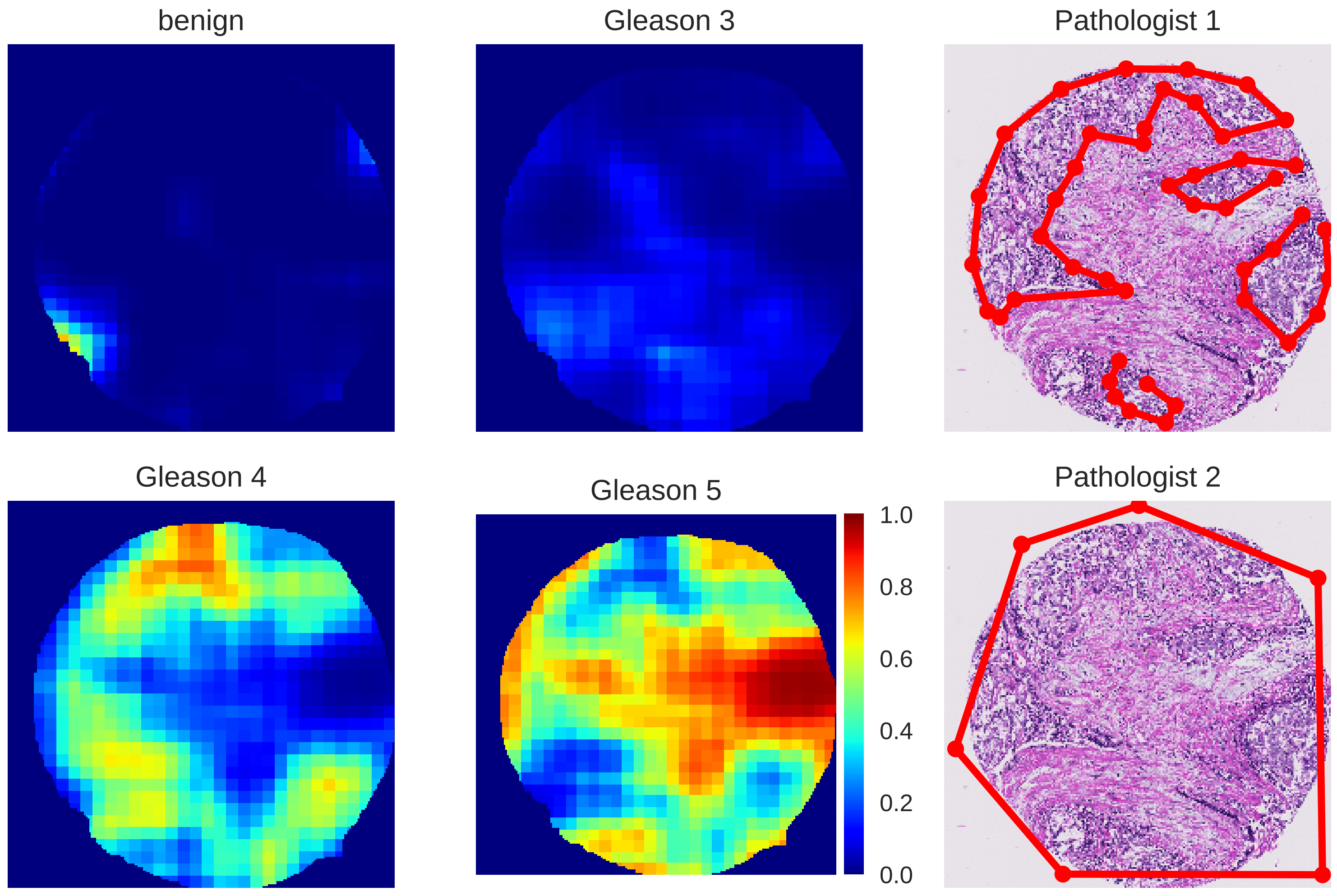
benign
Gleason 3
Pathologist 1
Gleason 4
Gleason 5
Pathologist 2
1.0
0.8
0.6
0.4
0.2
0.0

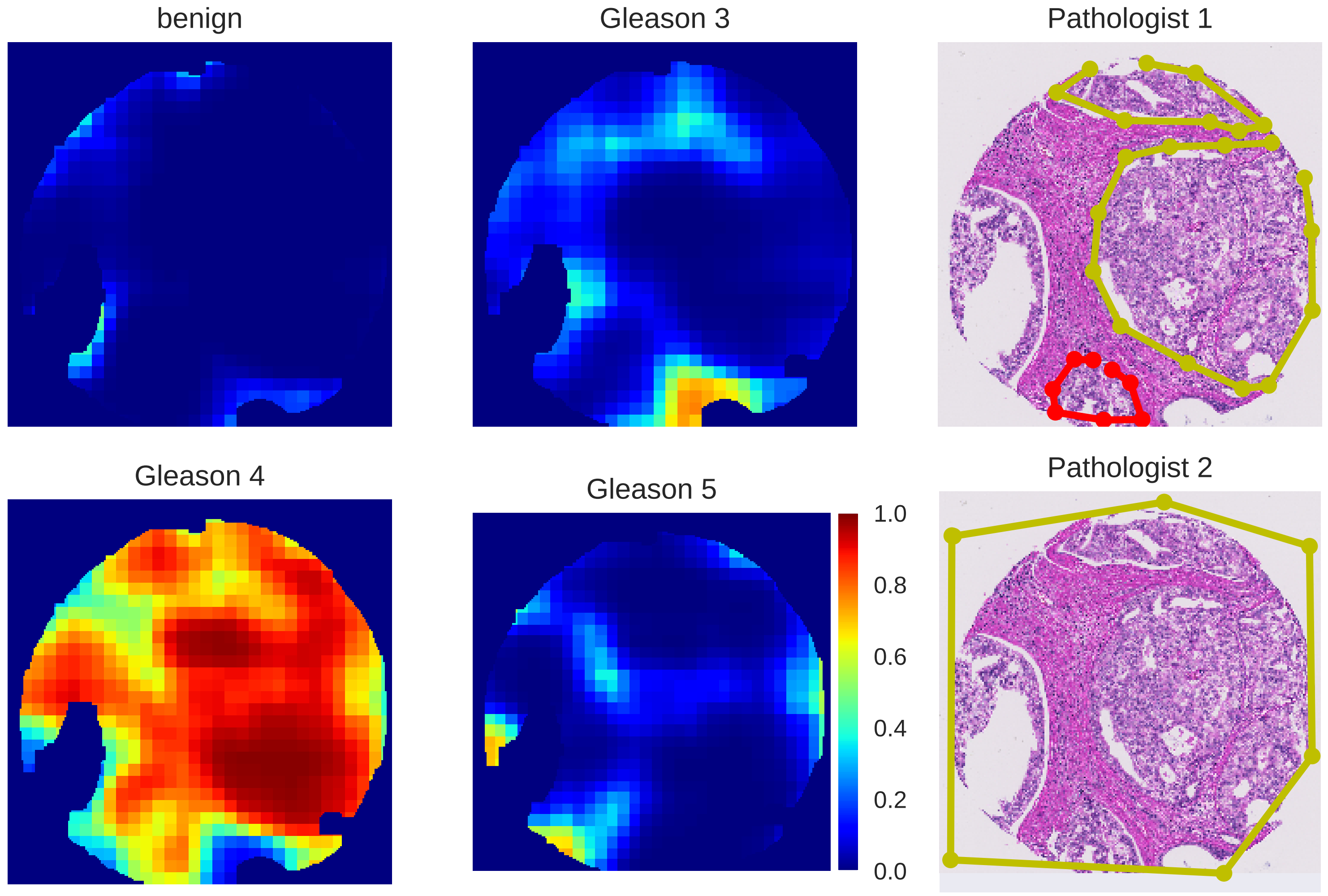
benign
Gleason 3
Pathologist 1
Gleason 4
Gleason 5
Pathologist 2
1.0
0.8
0.6
0.4
0.2
0.0

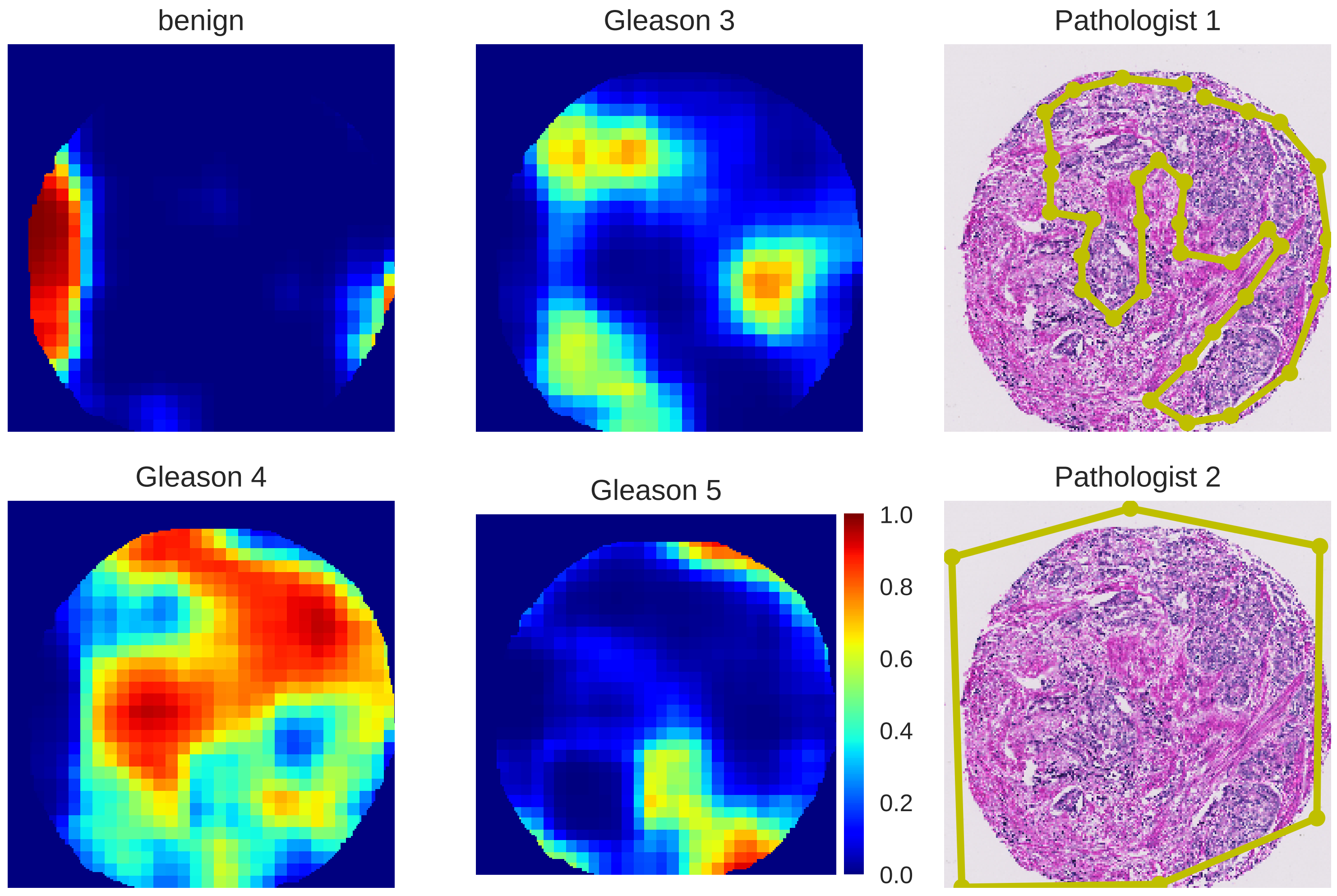
benign
Gleason 3
Pathologist 1
Gleason 4
Gleason 5
1.0
0.8
0.6
0.4
0.2
0.0
Pathologist 2

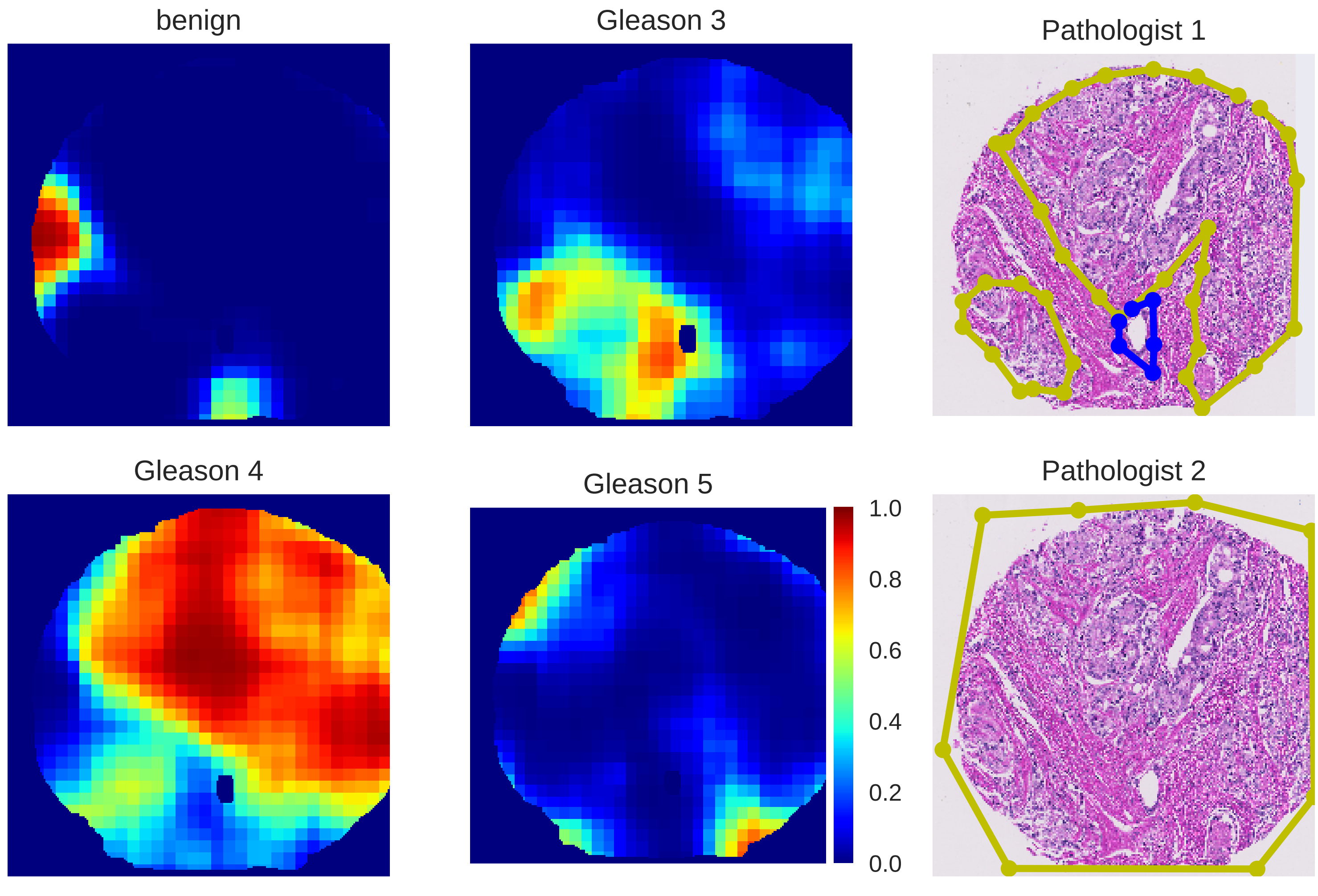
benign
Gleason 3
Pathologist 1
Gleason 4
Gleason 5
Pathologist 2
1.0
0.8
0.6
0.4
0.2
0.0

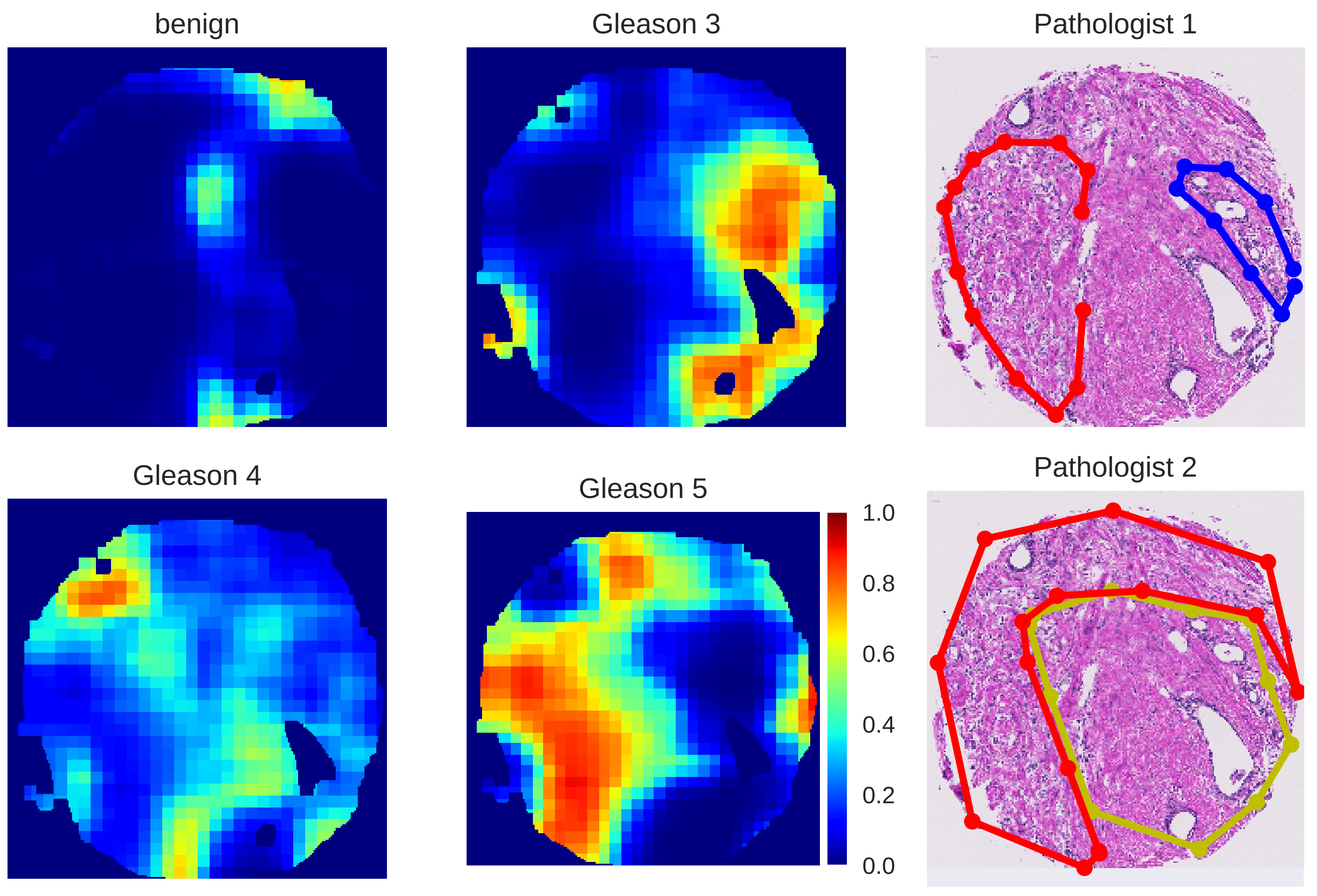

benign
Gleason 3
Pathologist 1
Gleason 4
Gleason 5
Pathologist 2
1.0
0.8
0.6
0.4
0.2
0.0

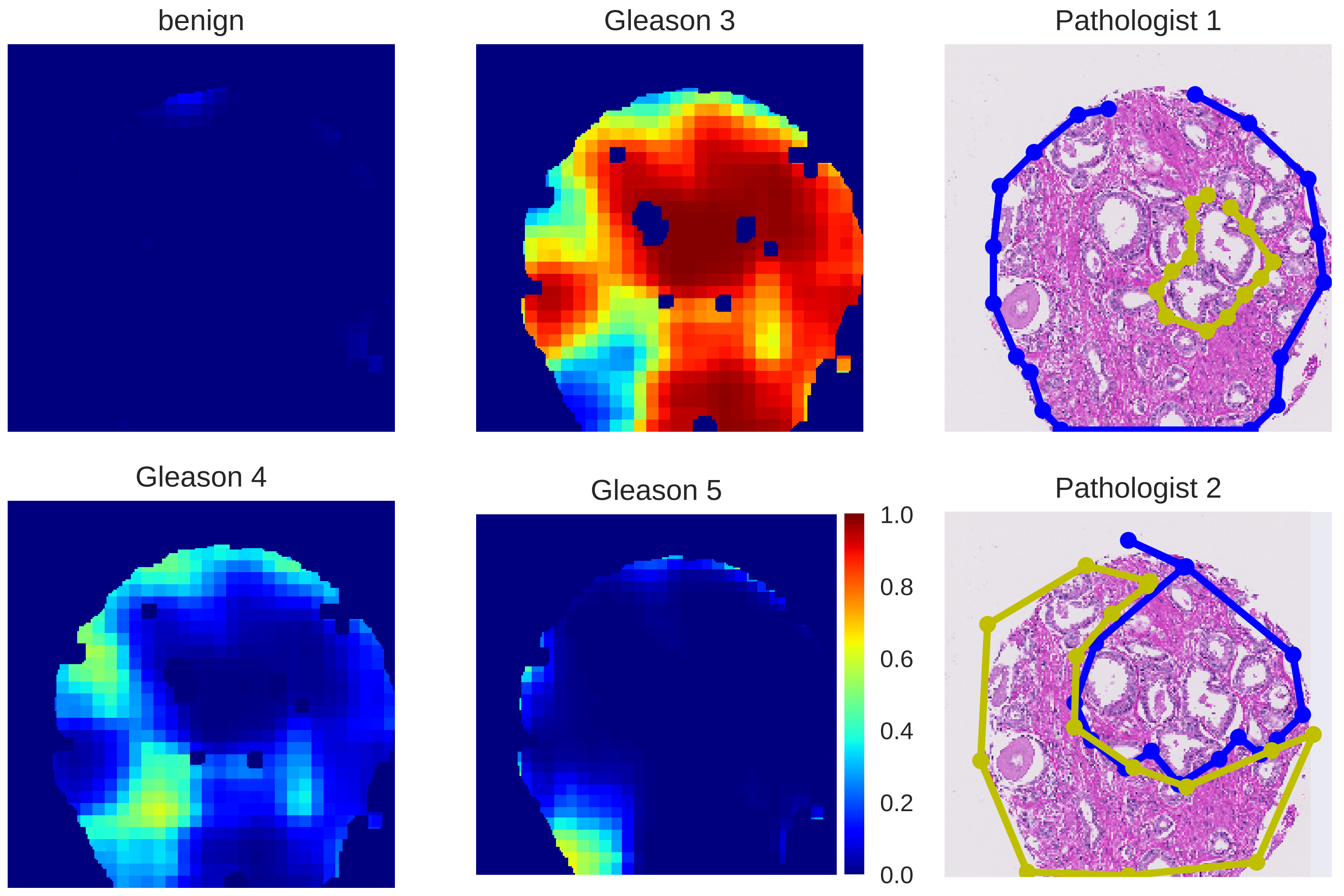
benign
Gleason 3
Pathologist 1
Gleason 4
Gleason 5
Pathologist 2
1.0
0.8
0.6
0.4
0.2
0.0

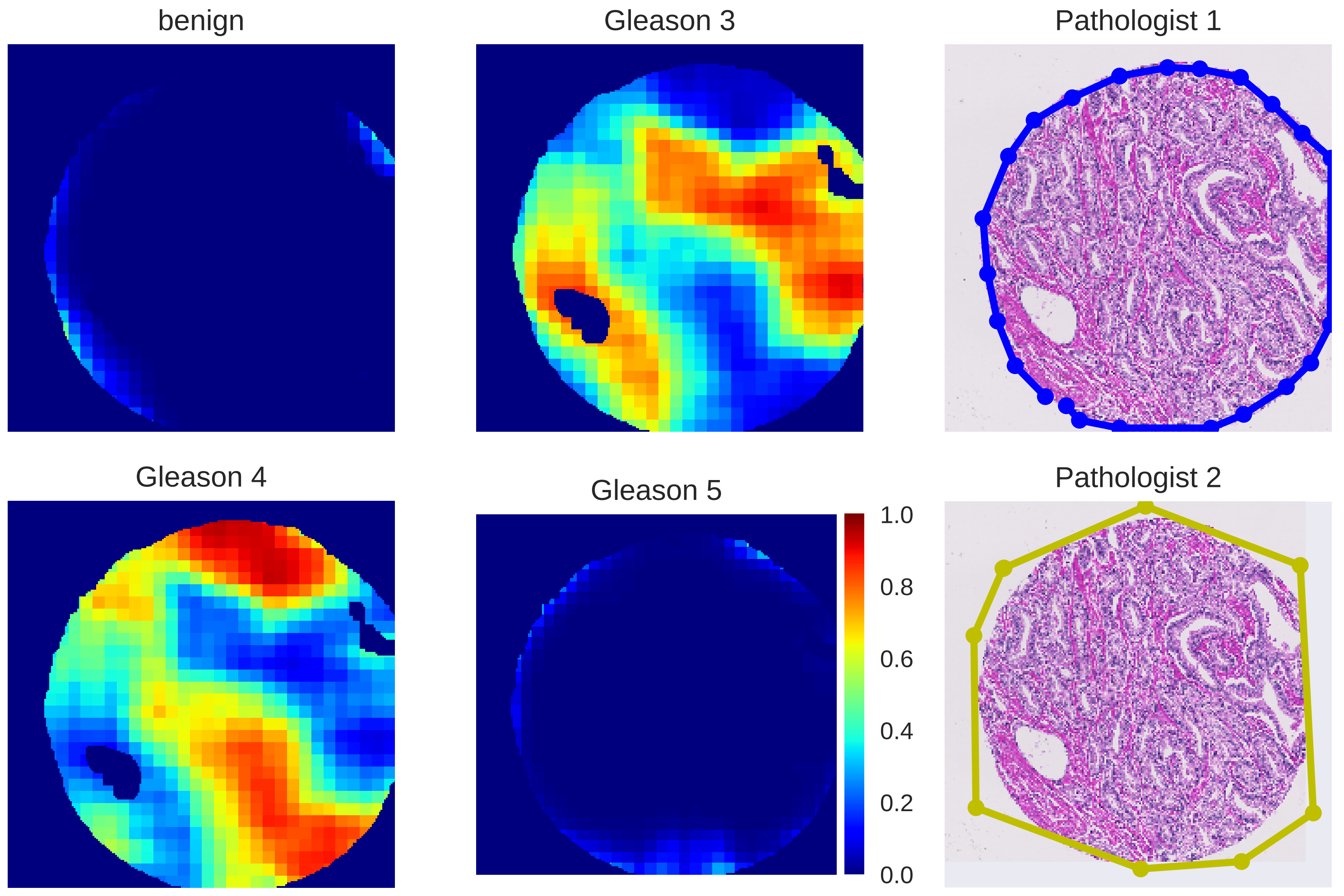
benign
Gleason 3
Pathologist 1
Gleason 4
Gleason 5
Pathologist 2
1.0
0.8
0.6
0.4
0.2
0.0

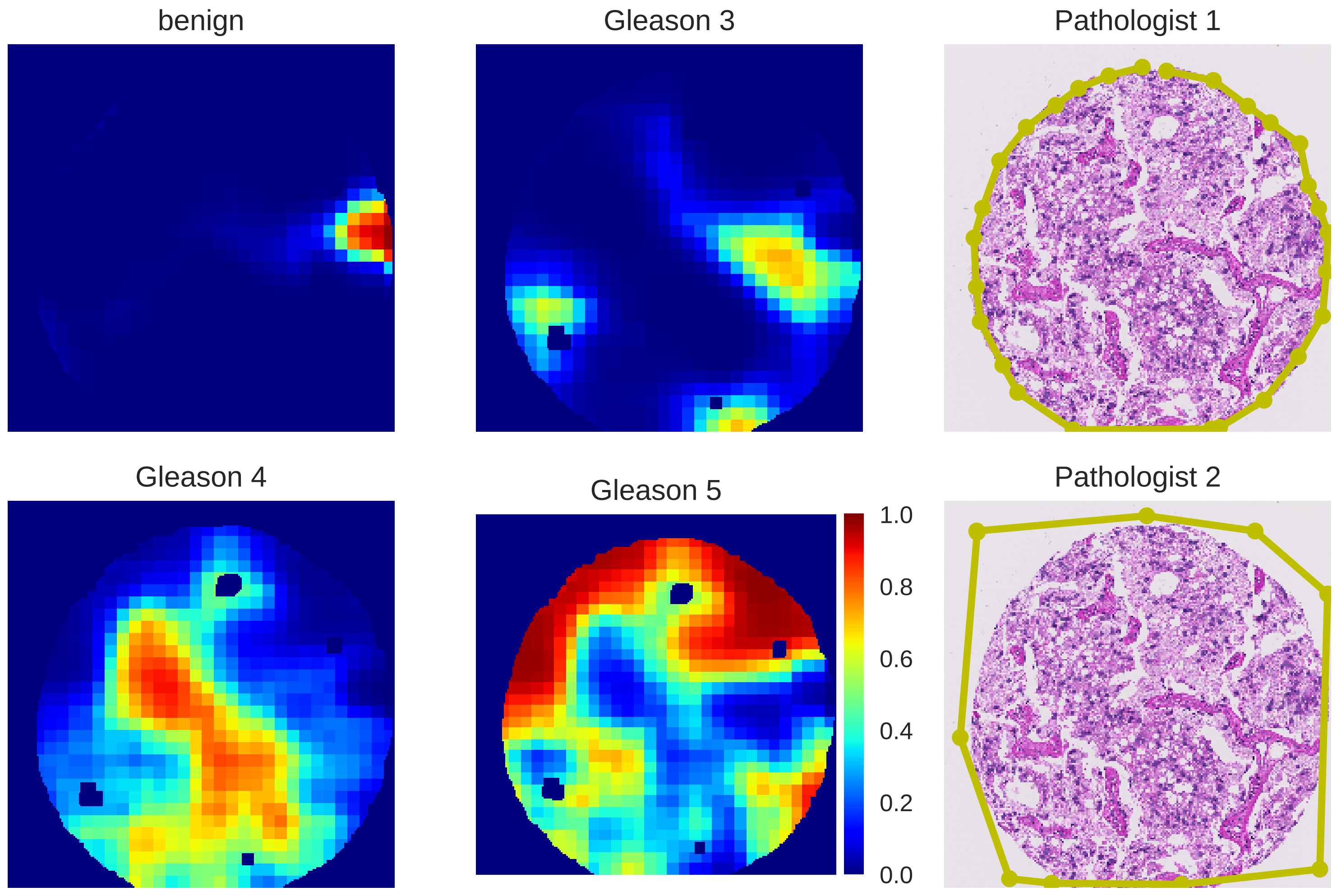
benign
Gleason 3
Pathologist 1
Gleason 4
Gleason 5
Pathologist 2
1.0
0.8
0.6
0.4
0.2
0.0

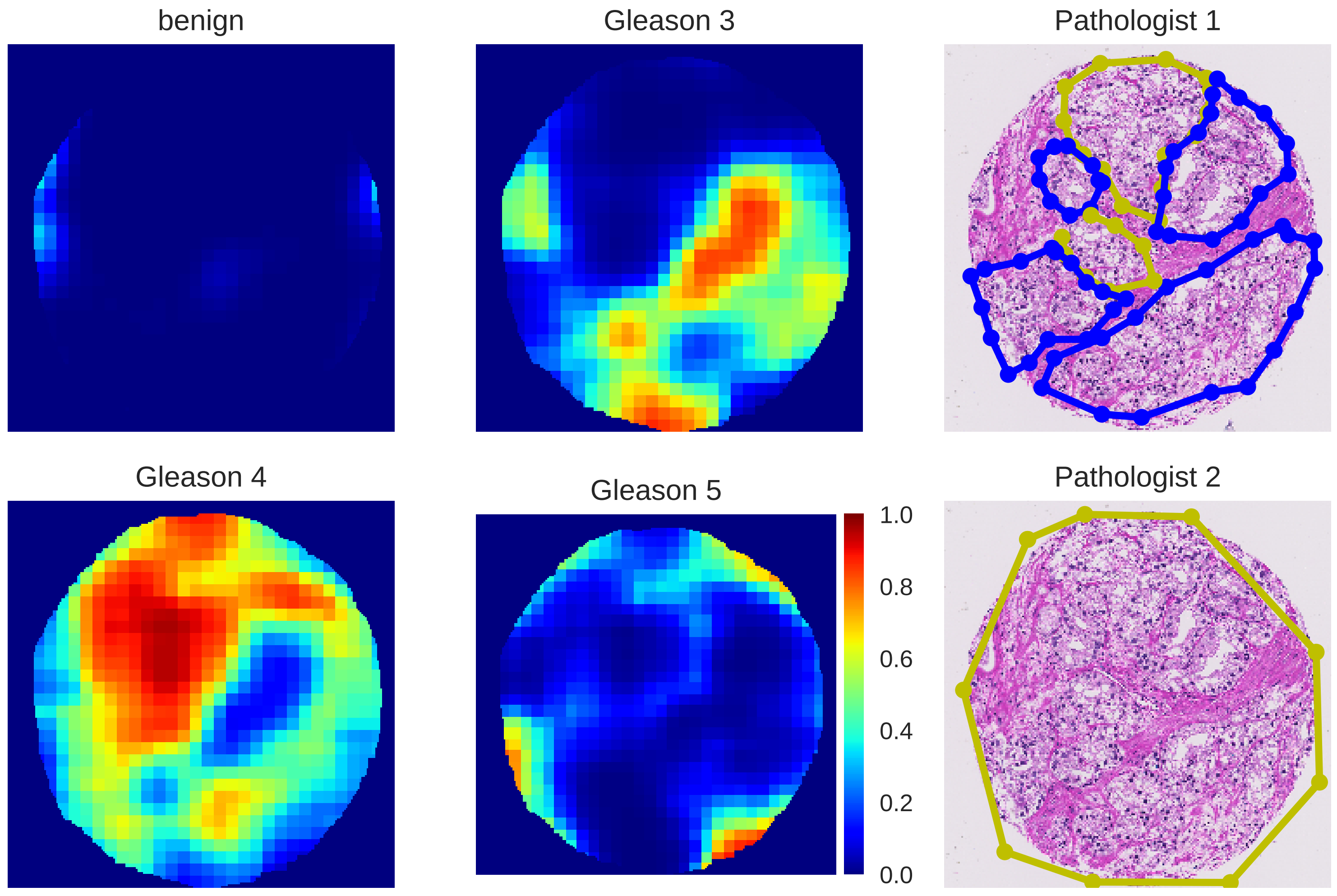

benign
Gleason 3
Pathologist 1
Gleason 4
Gleason 5
Pathologist 2
1.0
0.8
0.6
0.4
0.2
0.0

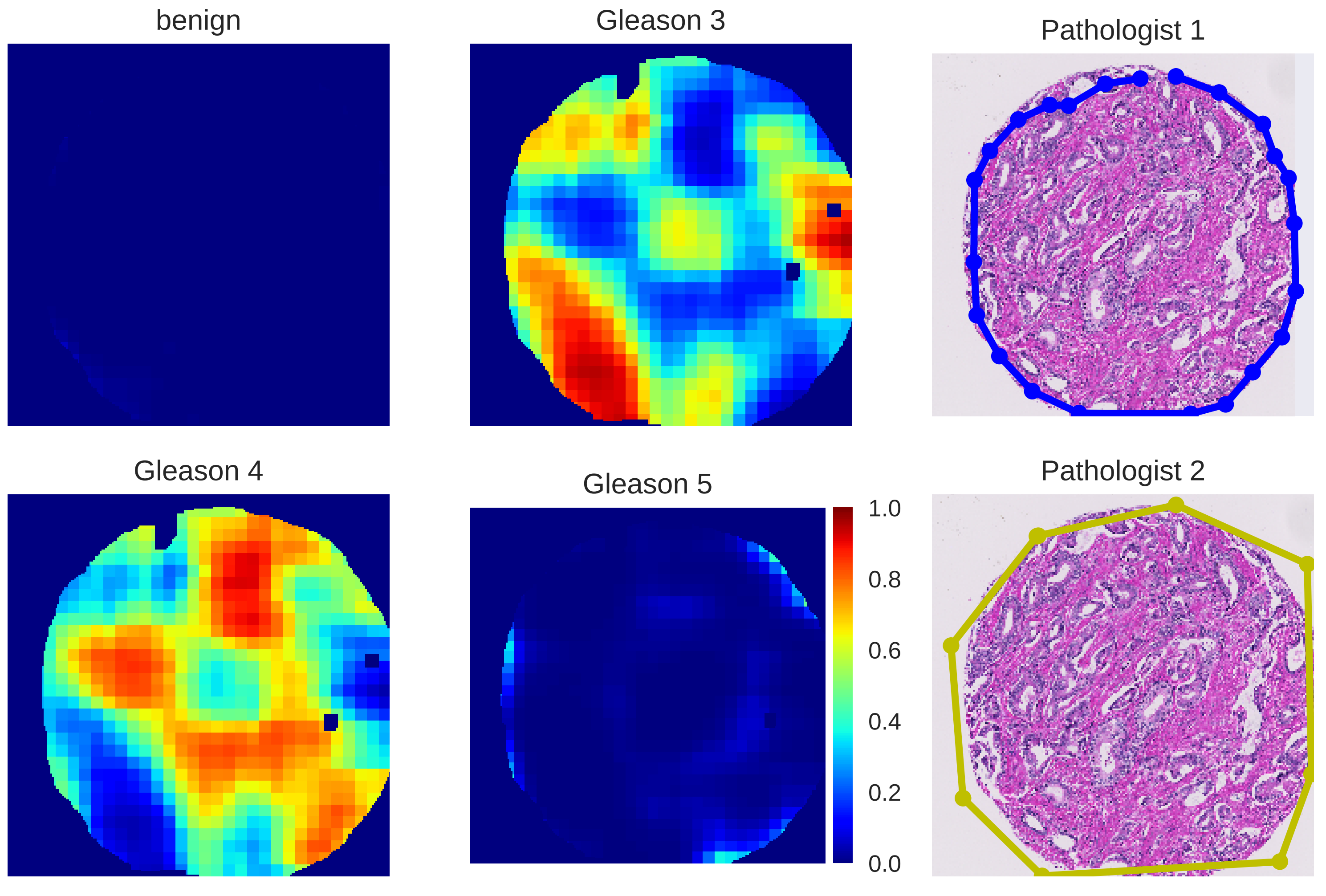

benign
Gleason 3
Pathologist 1
Gleason 4
Gleason 5
Pathologist 2
1.0
0.8
0.6
0.4
0.2
0.0

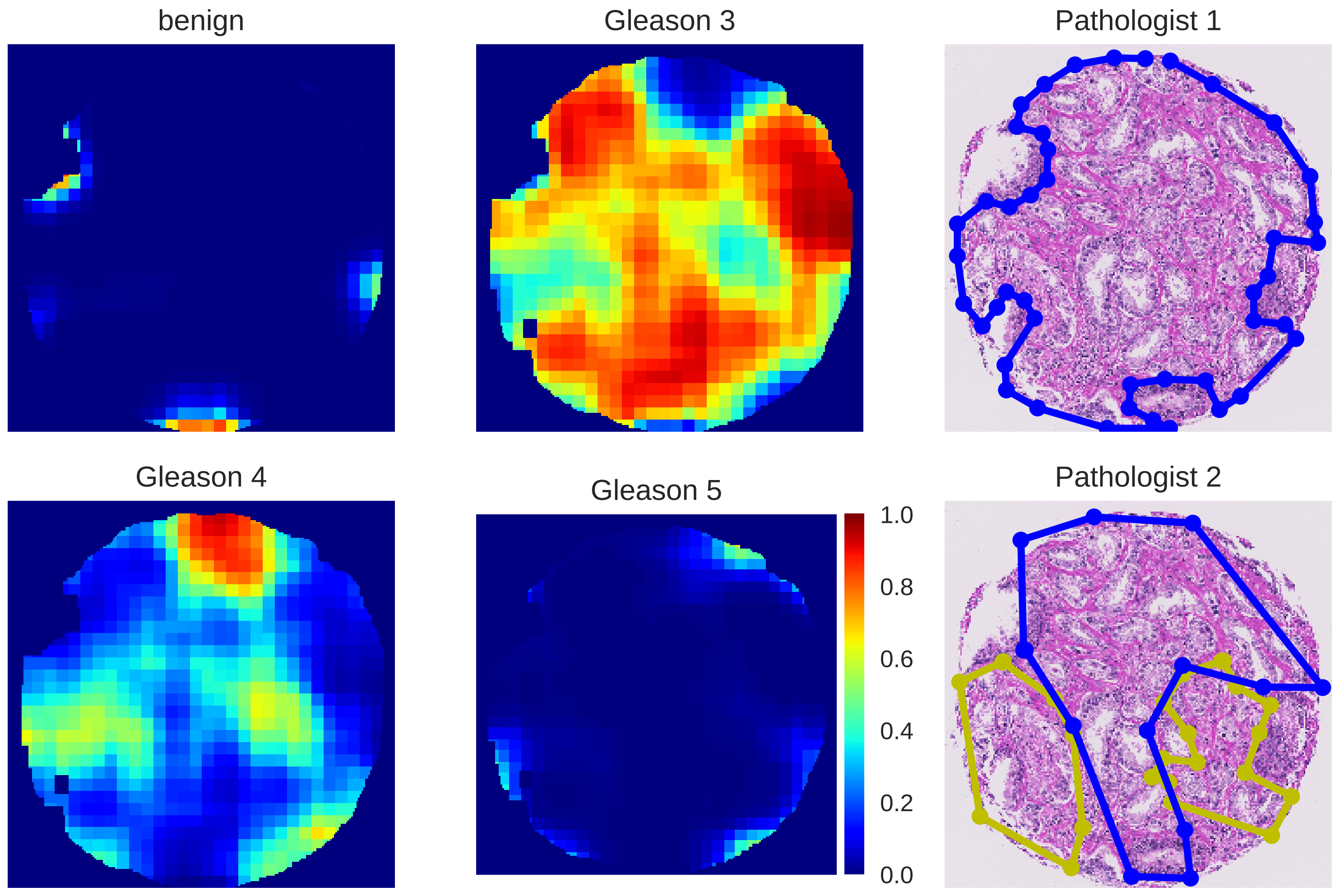
benign
Gleason 3
Pathologist 1
Gleason 4
Gleason 5
Pathologist 2
1.0
0.8
0.6
0.4
0.2
0.0

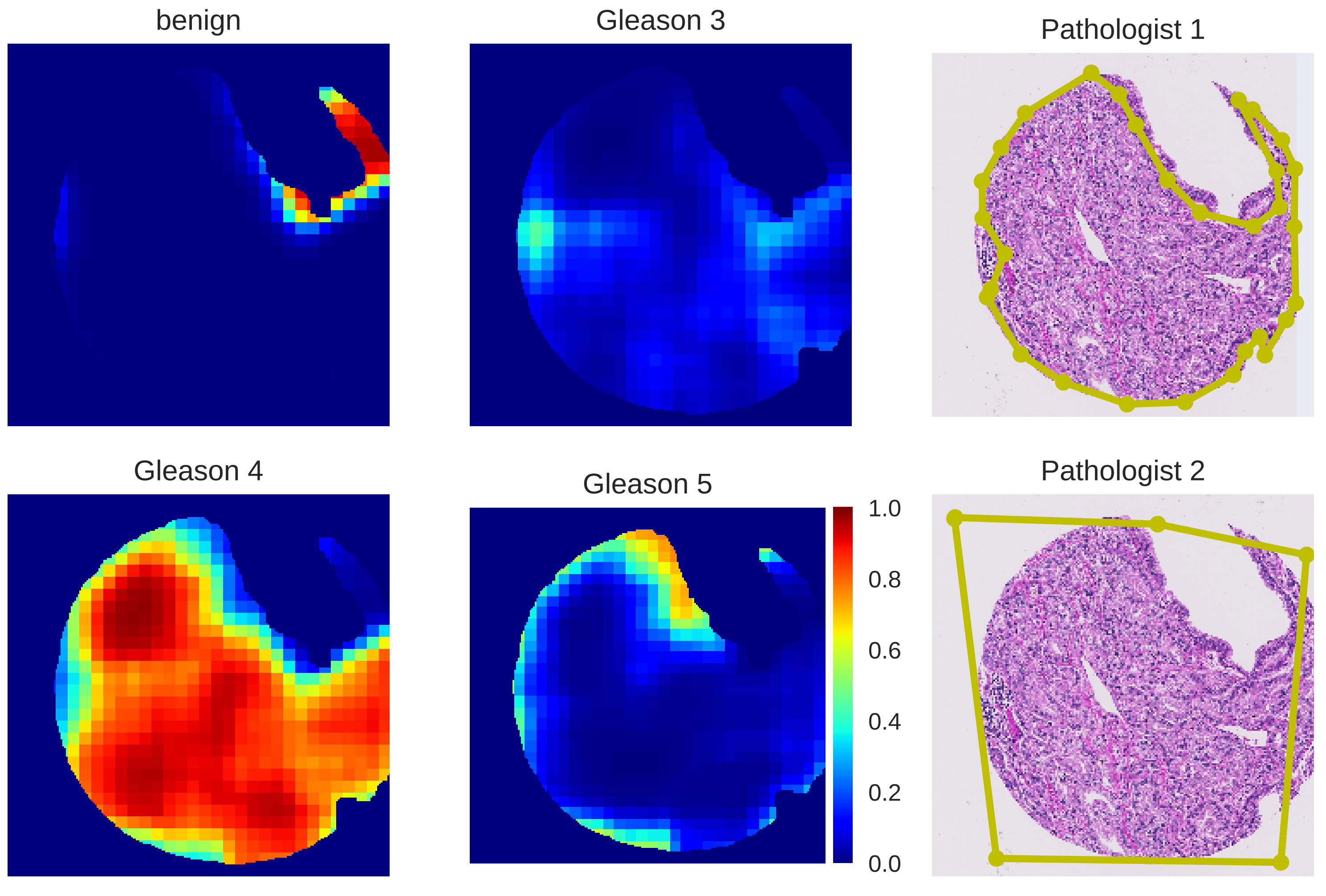

benign
Gleason 3
Pathologist 1
Gleason 4
Gleason 5
Pathologist 2
1.0
0.8
0.6
0.4
0.2
0.0

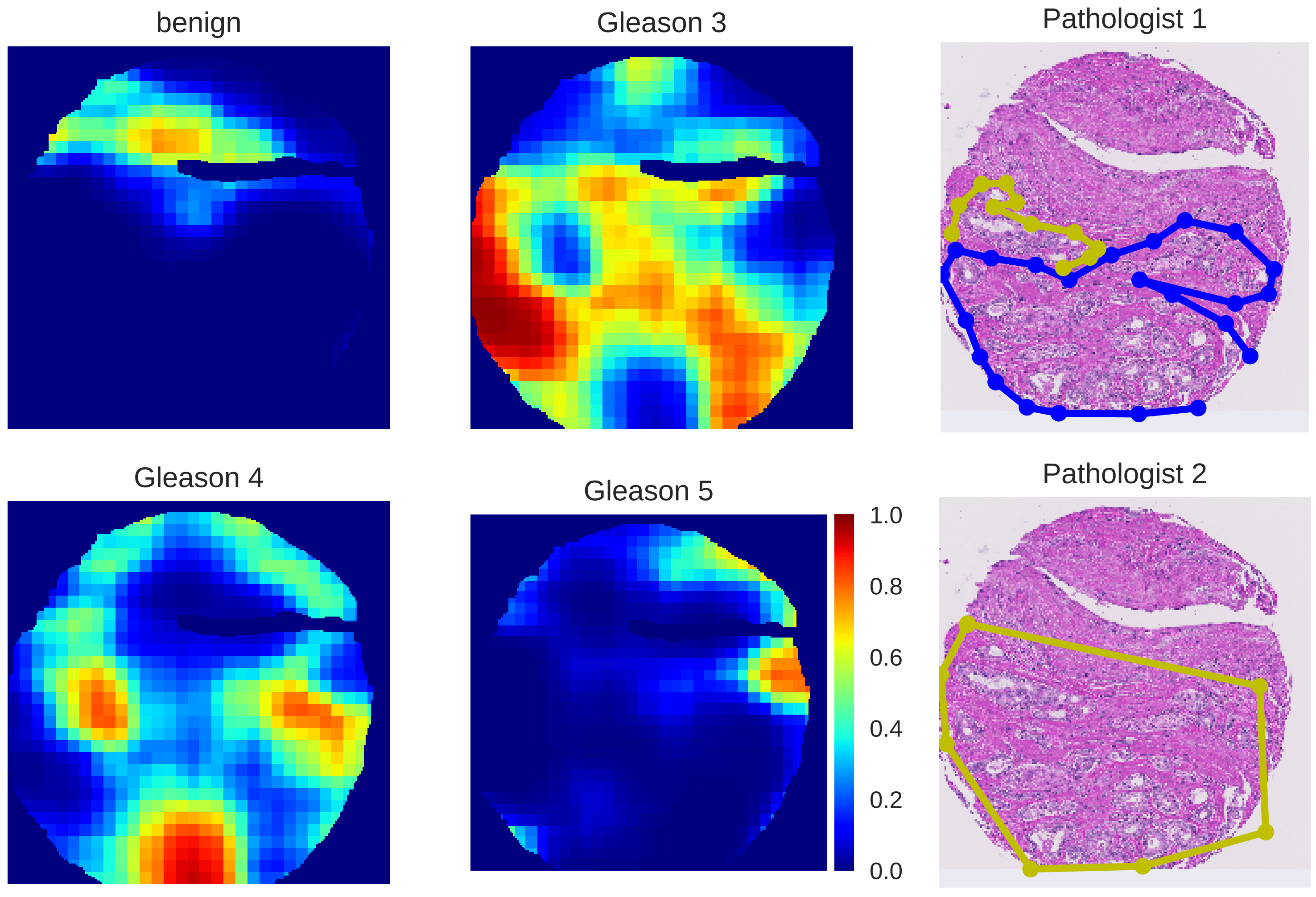

benign
Gleason 3
Pathologist 1
Gleason 4
Gleason 5
Pathologist 2
1.0
0.8
0.6
0.4
0.2
0.0

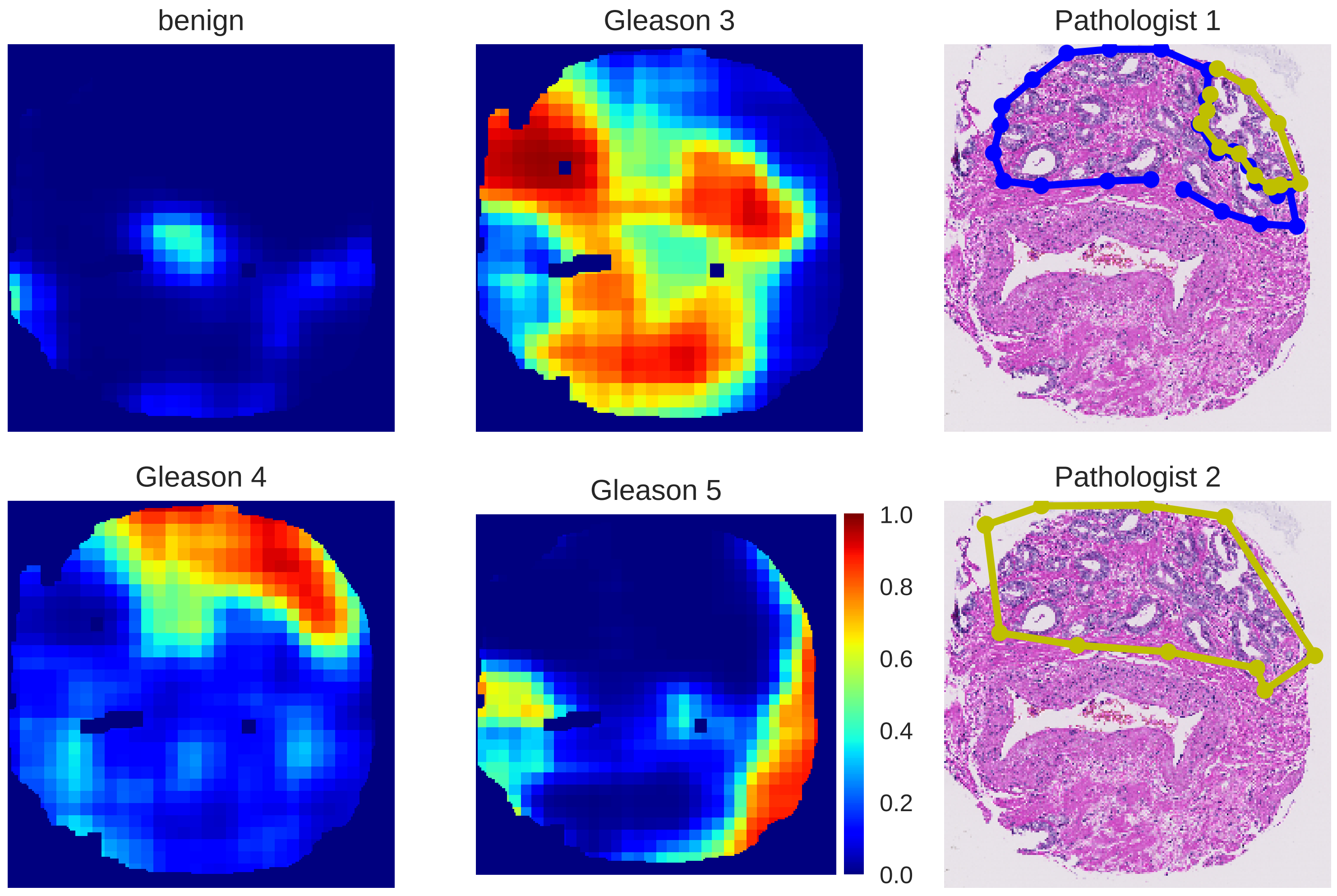

benign
Gleason 3
Pathologist 1
Gleason 4
Gleason 5
Pathologist 2
1.0
0.8
0.6
0.4
0.2
0.0

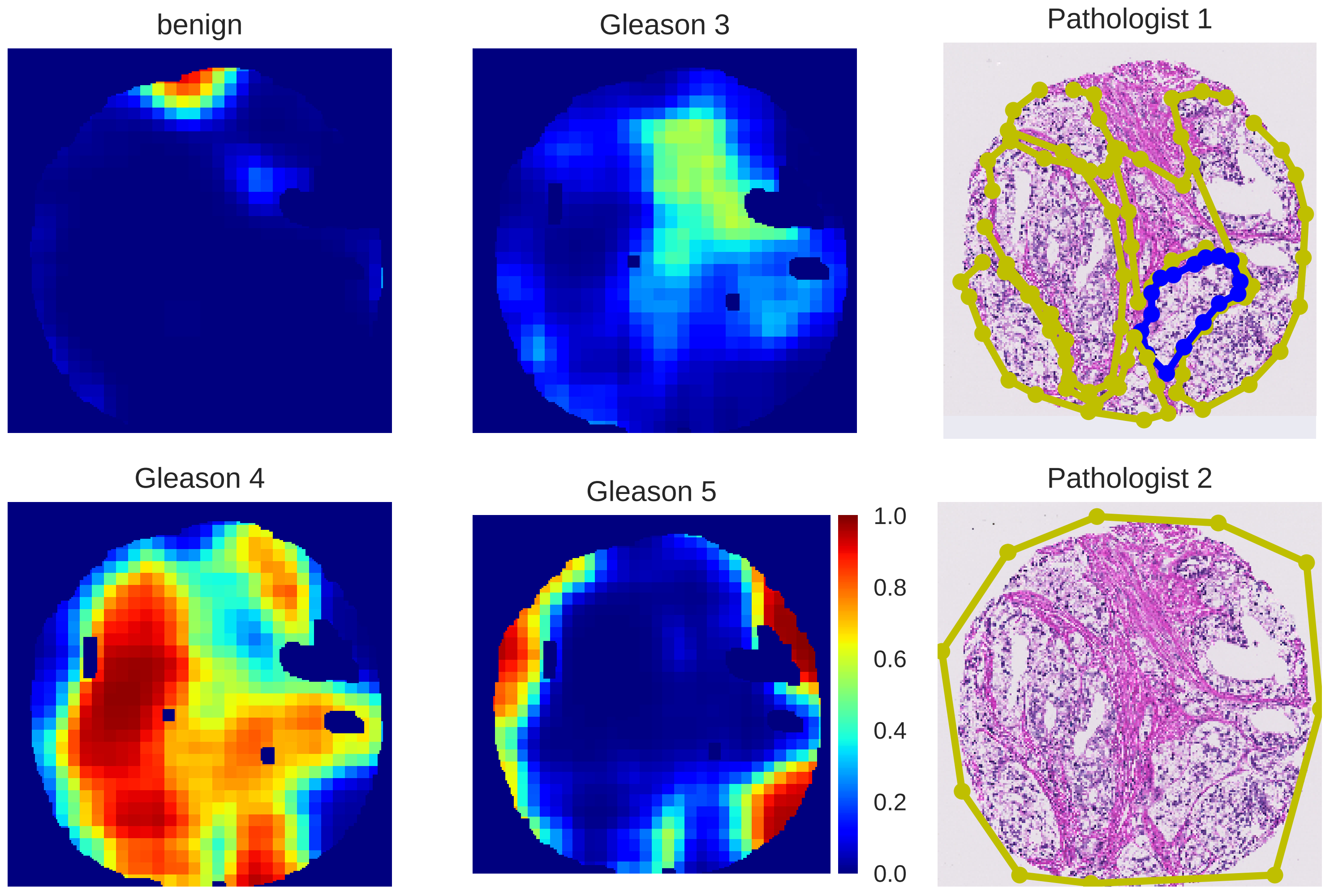

benign
Gleason 3
Pathologist 1
Gleason 4
Gleason 5
Pathologist 2
1.0
0.8
0.6
0.4
0.2
0.0

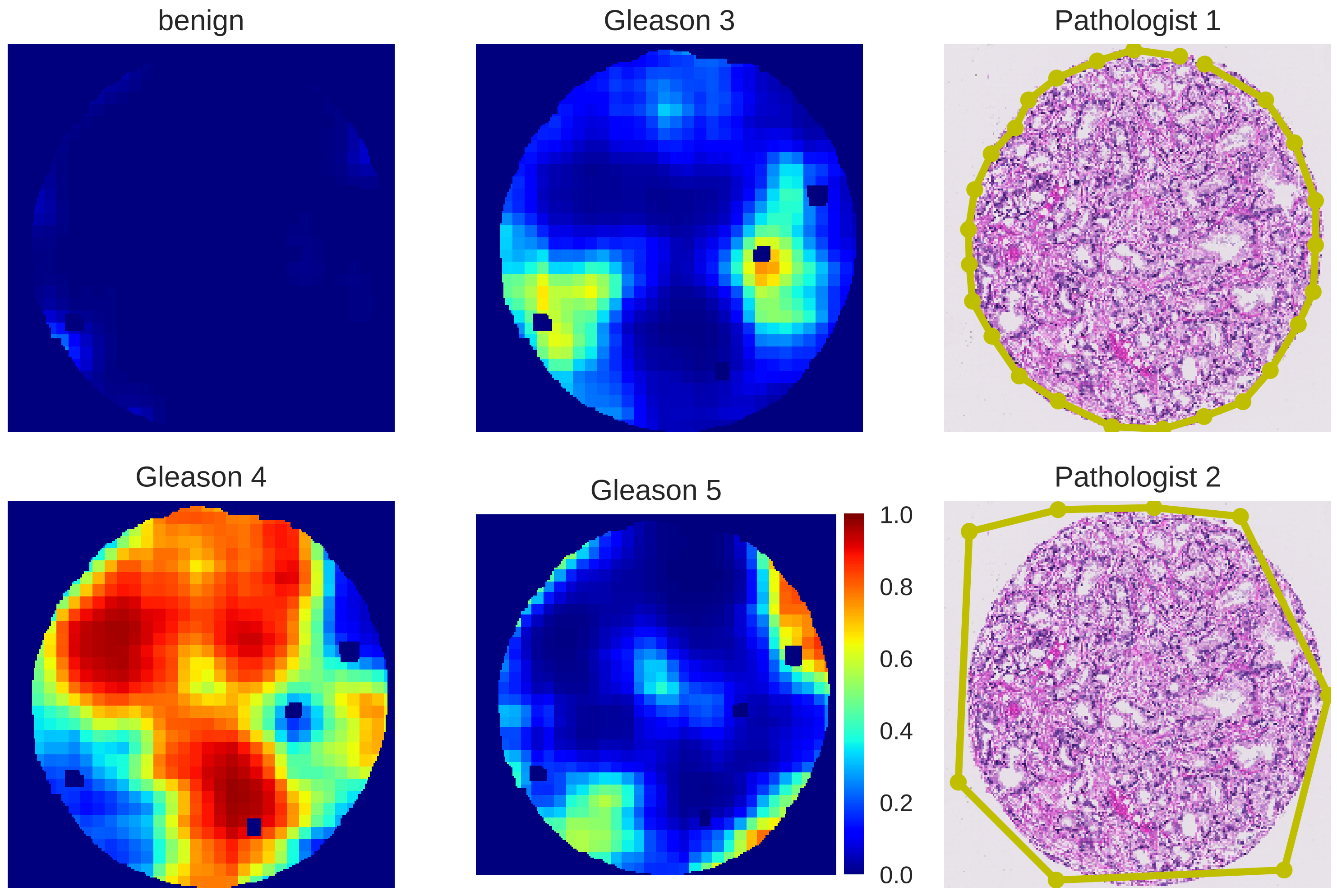

benign
Gleason 3
Pathologist 1
Gleason 4
Gleason 5
Pathologist 2
1.0
0.8
0.6
0.4
0.2
0.0

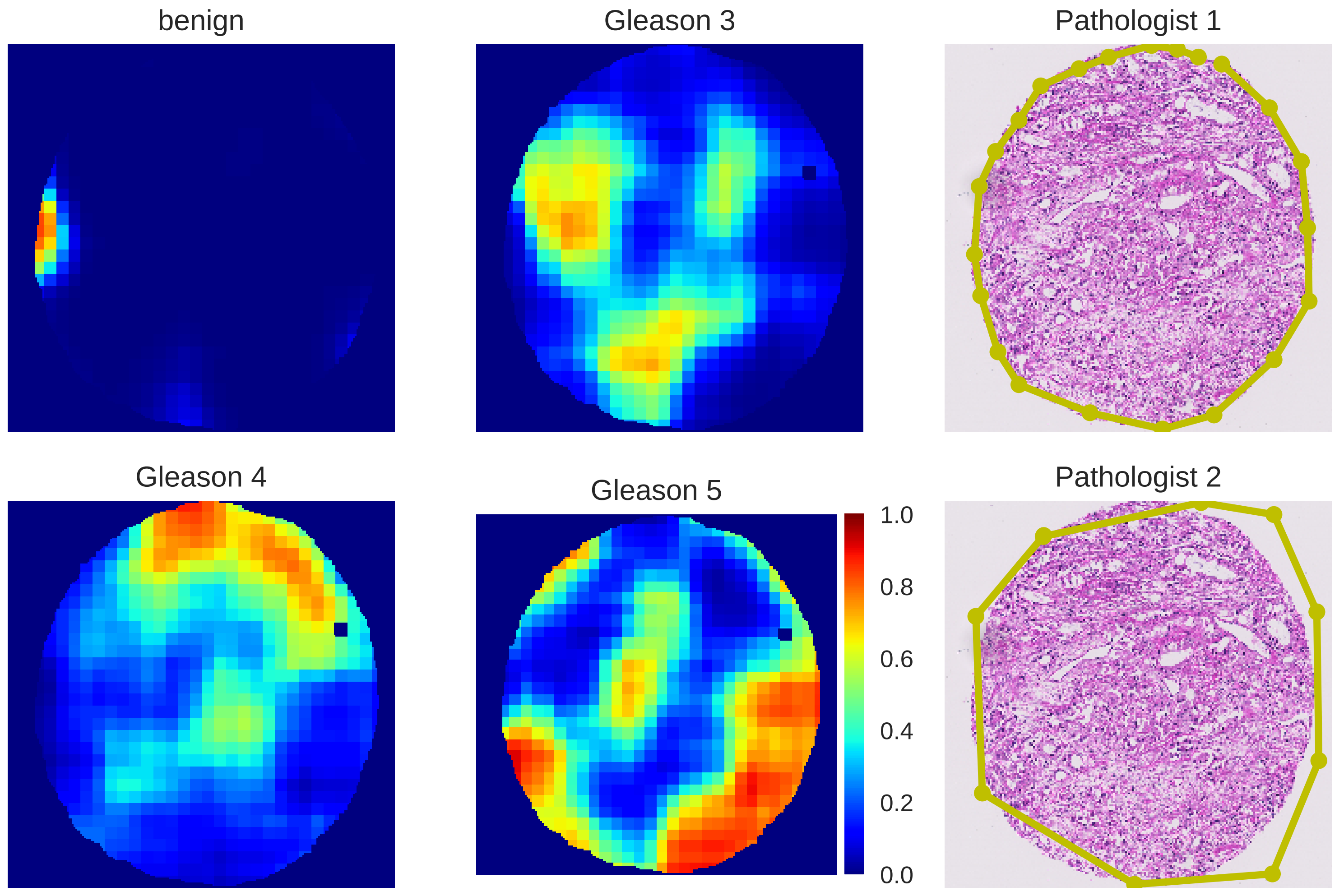

benign
Gleason 3
Pathologist 1
Gleason 4
Gleason 5
Pathologist 2
1.0
0.8
0.6
0.4
0.2
0.0

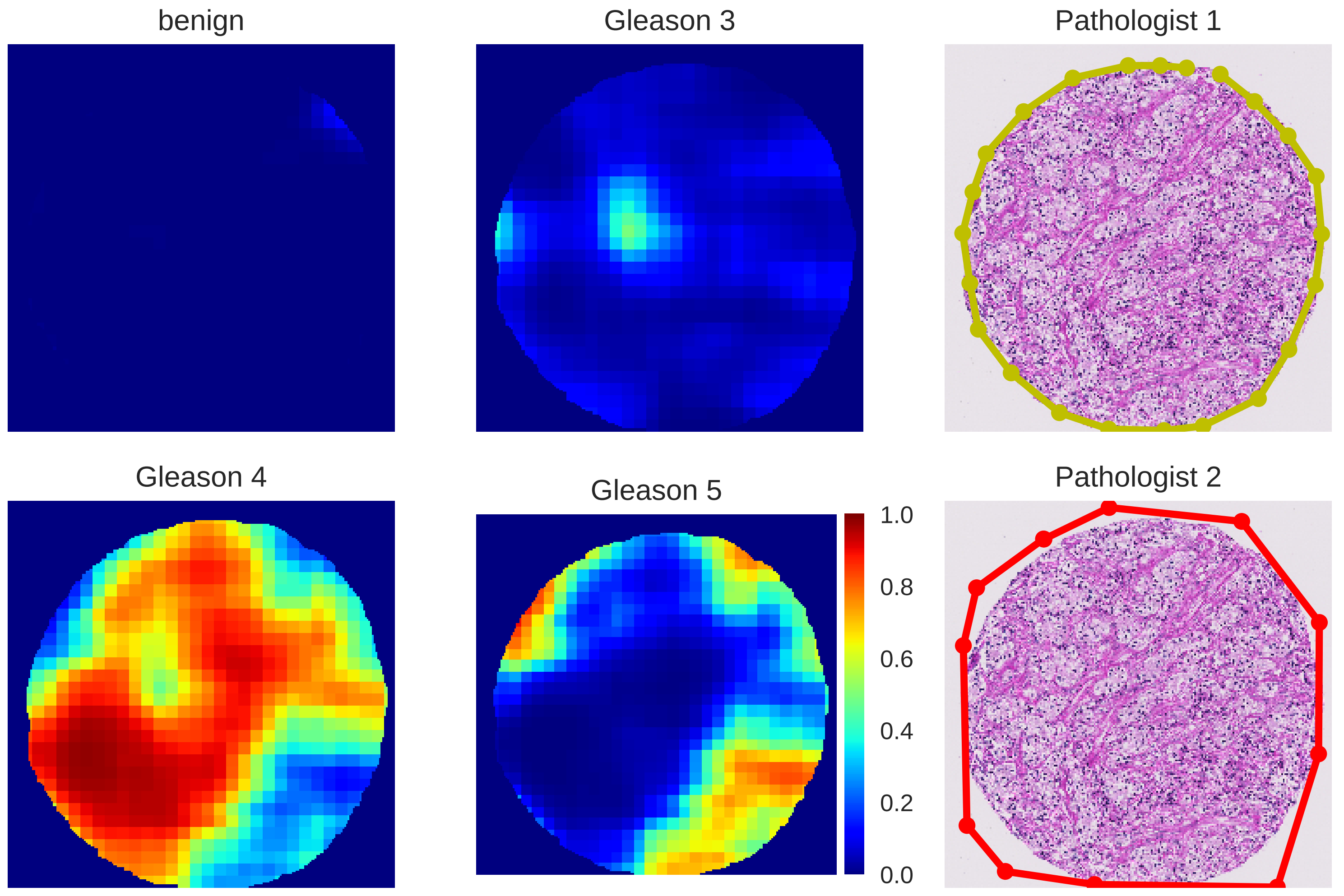

benign
Gleason 3
Pathologist 1
Gleason 4
Gleason 5
Pathologist 2
1.0
0.8
0.6
0.4
0.2
0.0

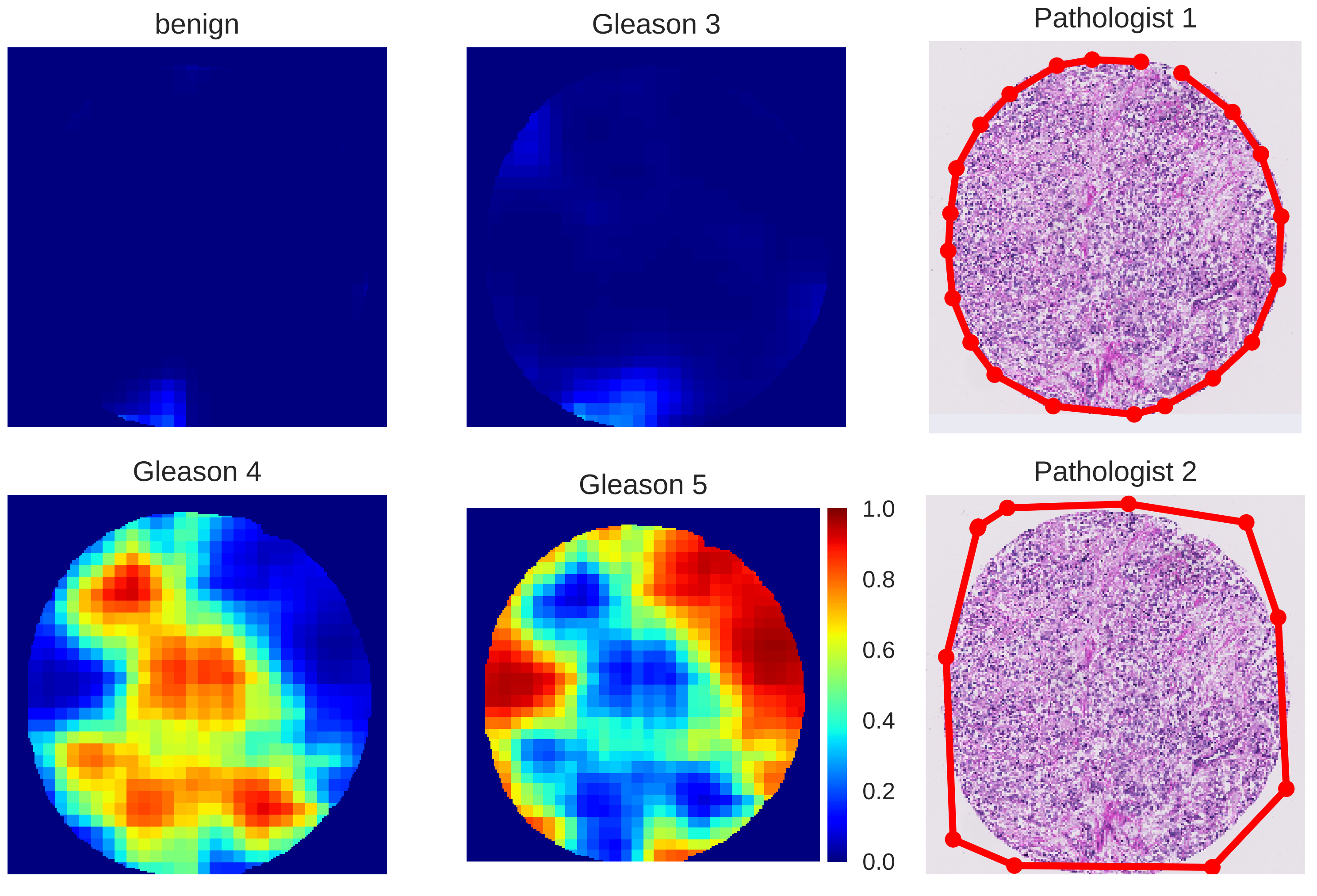

benign
Gleason 3
Pathologist 1
Gleason 4
Gleason 5
Pathologist 2
1.0
0.8
0.6
0.4
0.2
0.0

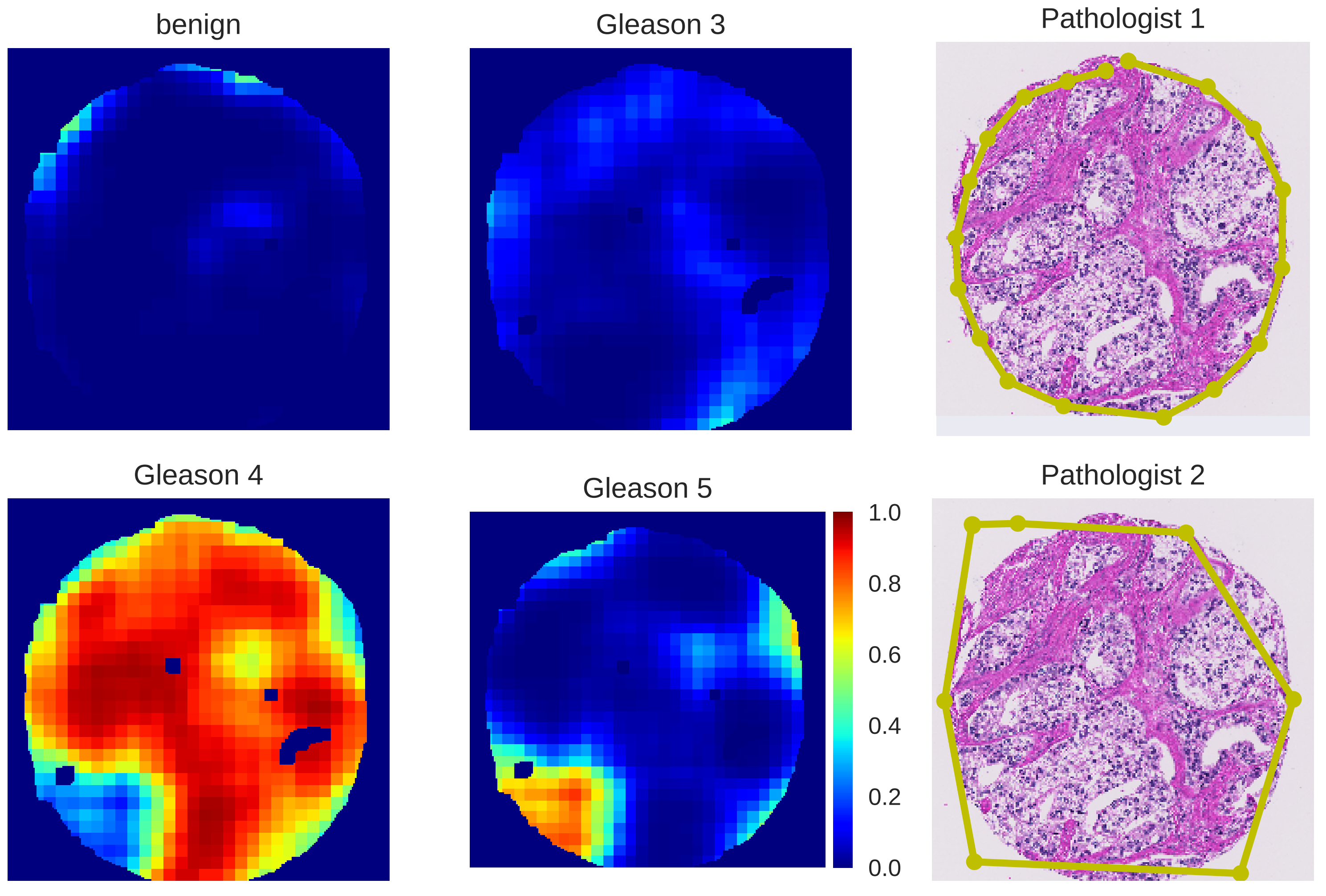

benign
Gleason 3
Pathologist 1
Gleason 4
Gleason 5
Pathologist 2
1.0
0.8
0.6
0.4
0.2
0.0

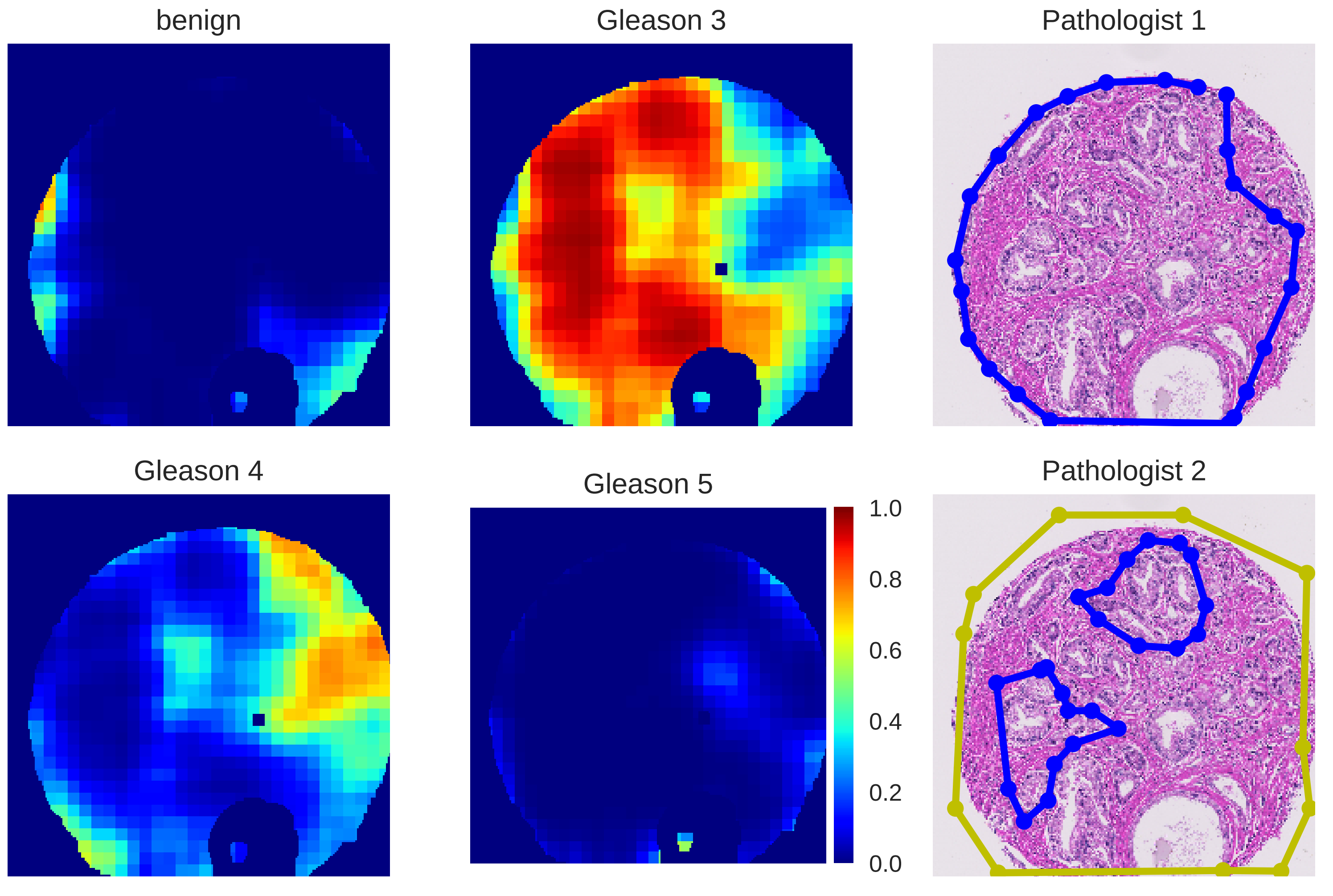

benign
Gleason 3
Pathologist 1
Gleason 4
Gleason 5
Pathologist 2
1.0
0.8
0.6
0.4
0.2
0.0

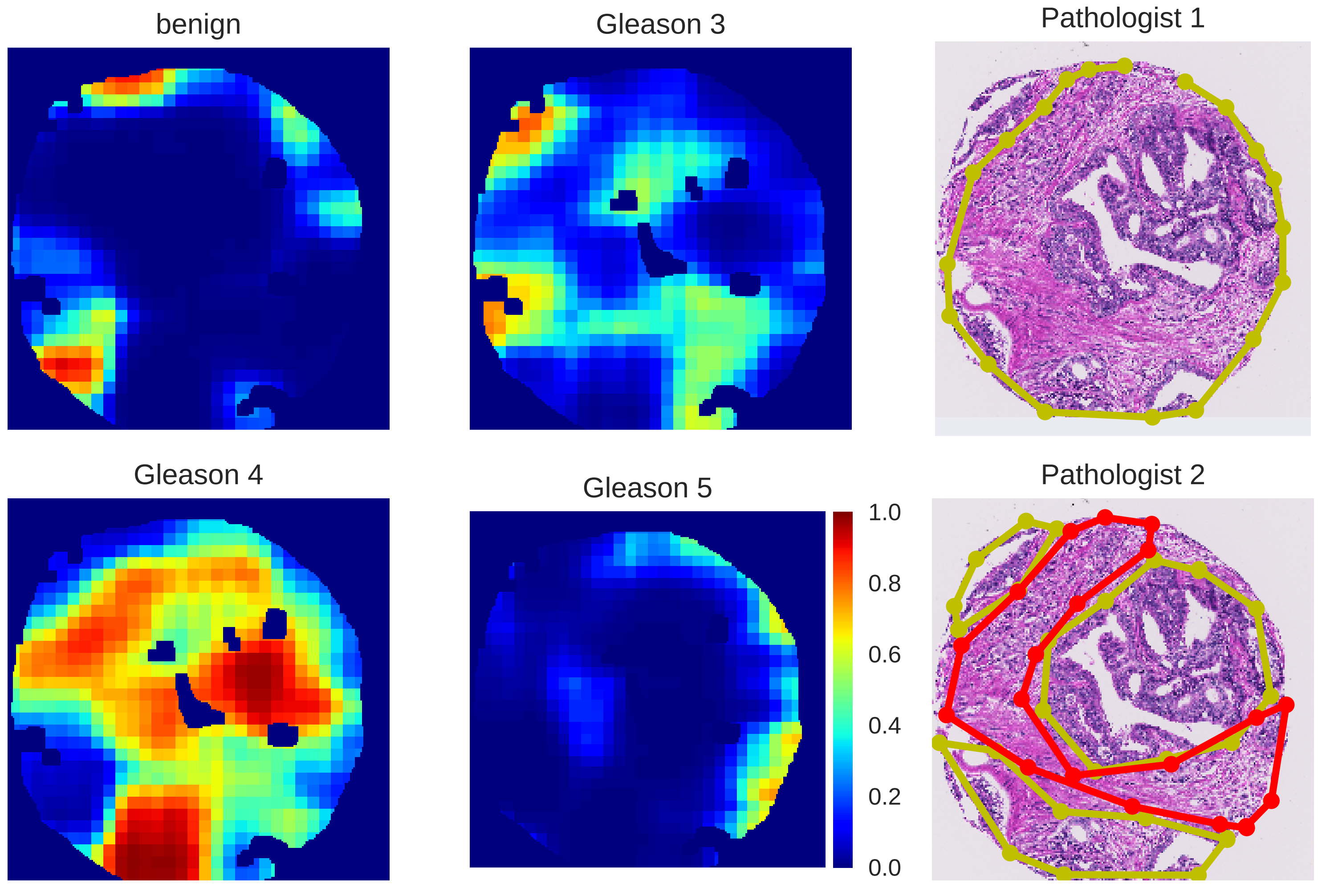

benign
Gleason 3
Pathologist 1
Gleason 4
Gleason 5
Pathologist 2
1.0
0.8
0.6
0.4
0.2
0.0

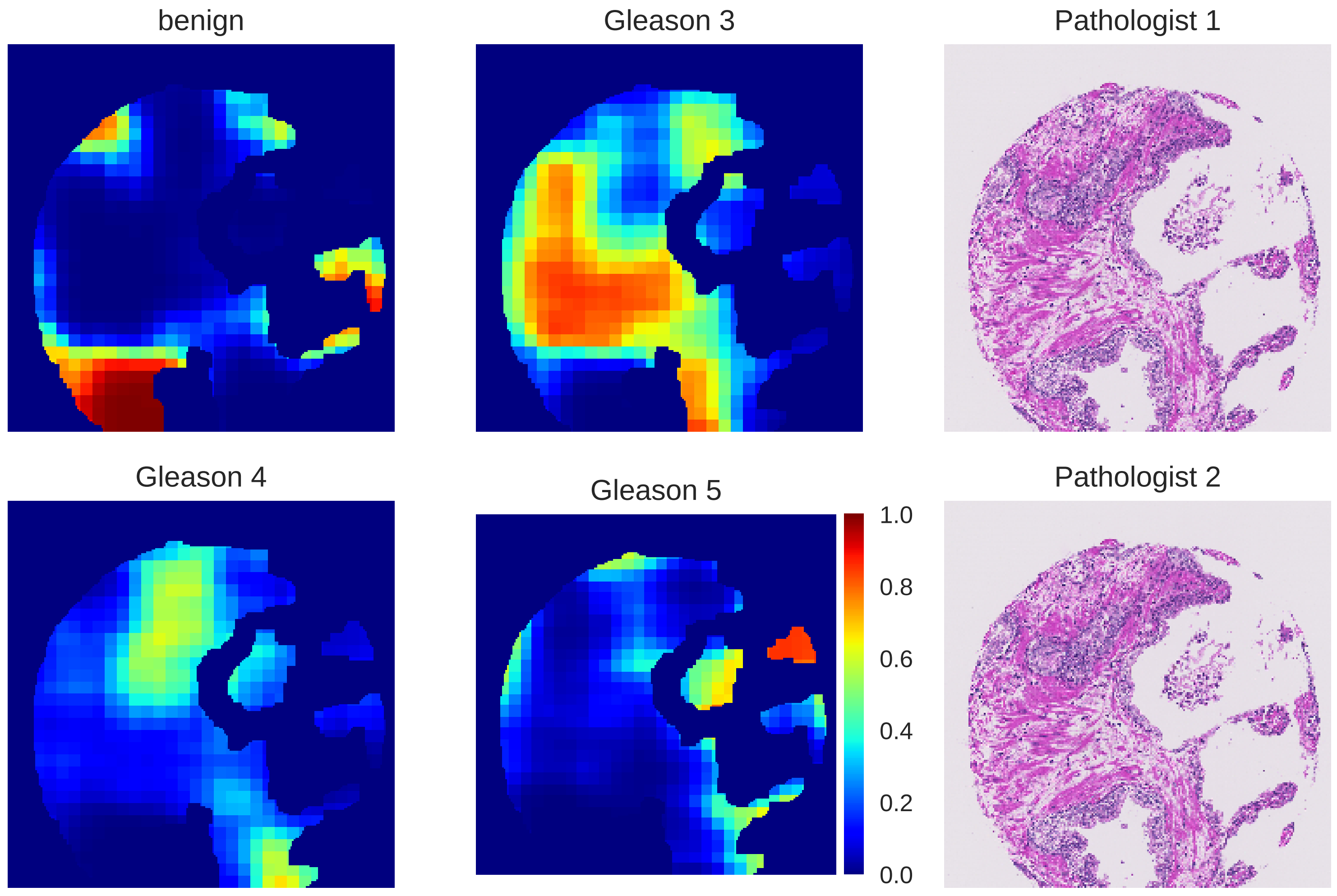

benign
Gleason 3
Pathologist 1
Gleason 4
Gleason 5
Pathologist 2
1.0
0.8
0.6
0.4
0.2
0.0

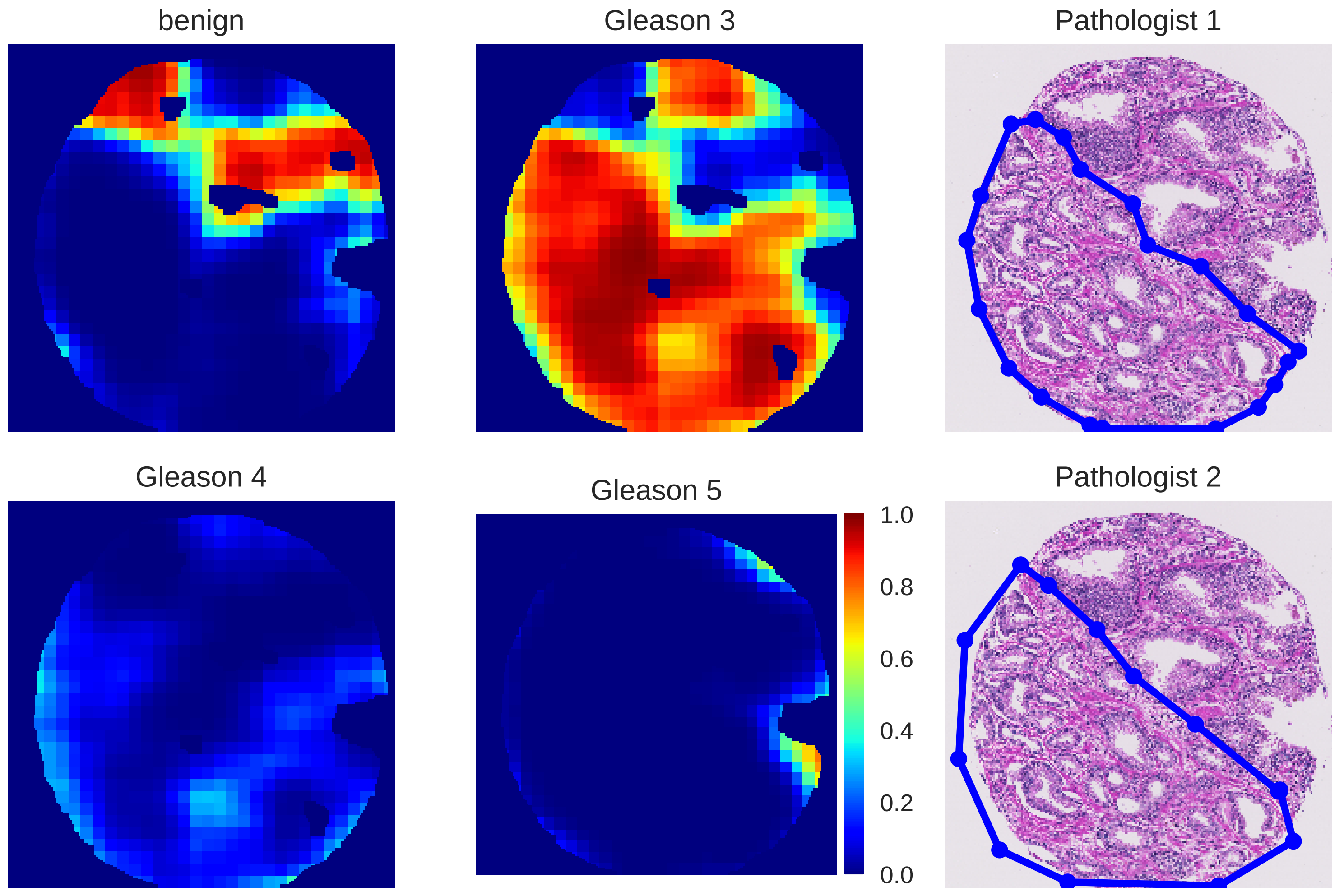

benign
Gleason 3
Pathologist 1
Gleason 4
Gleason 5
Pathologist 2
1.0
0.8
0.6
0.4
0.2
0.0

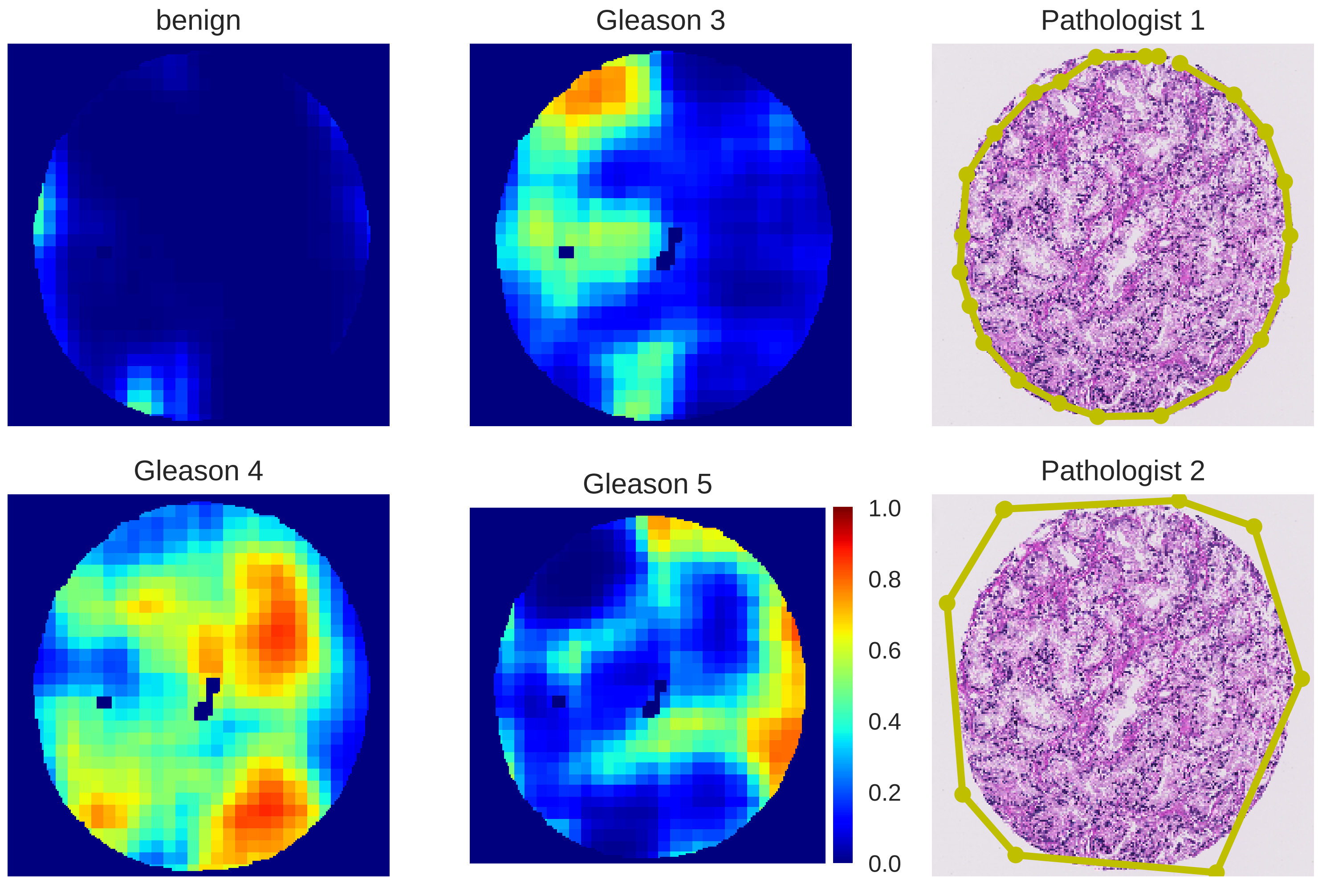
benign
Gleason 3
Pathologist 1
Gleason 4
Gleason 5
Pathologist 2
1.0
0.8
0.6
0.4
0.2
0.0

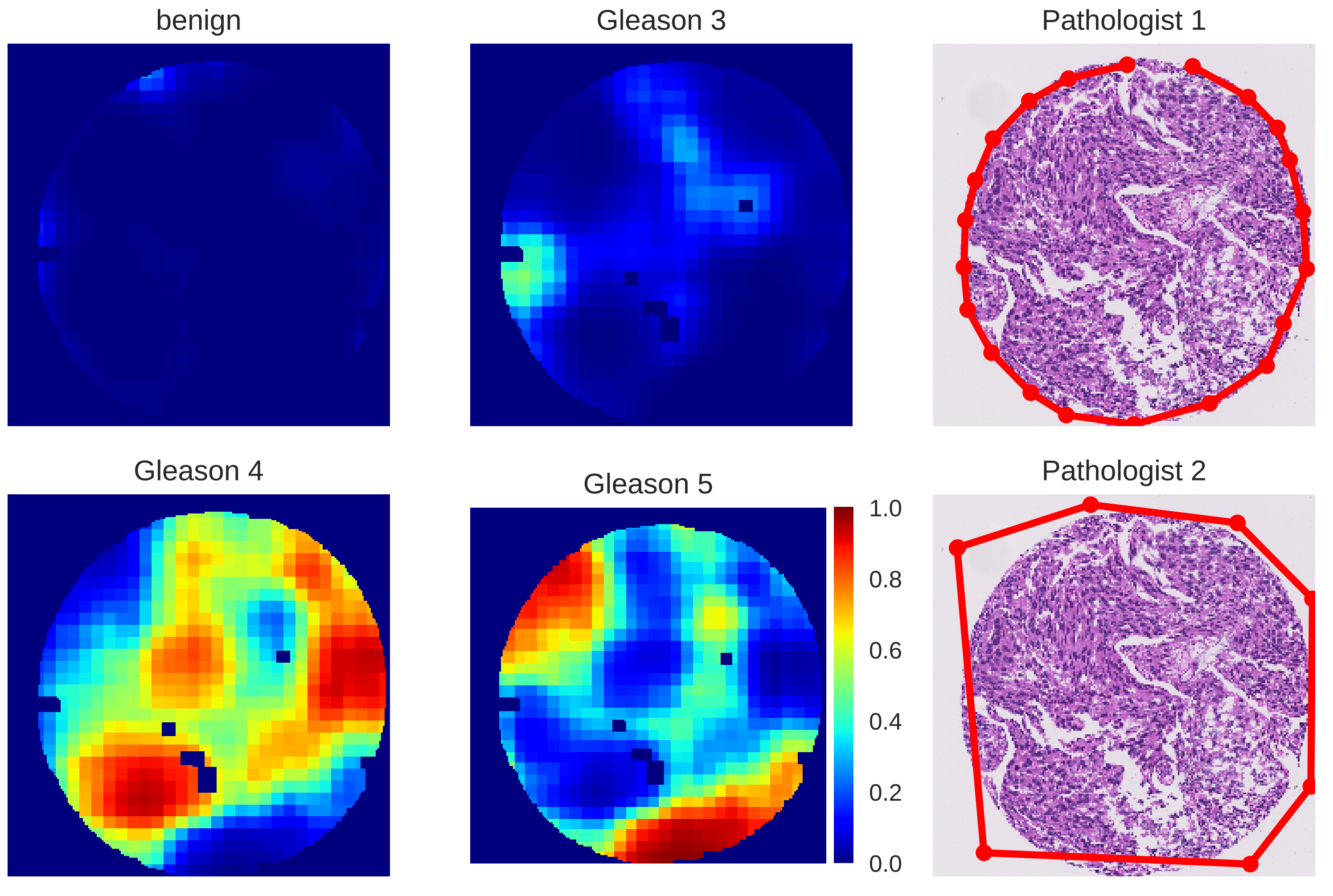

benign
Gleason 3
Pathologist 1
Gleason 4
Gleason 5
Pathologist 2
1.0
0.8
0.6
0.4
0.2
0.0

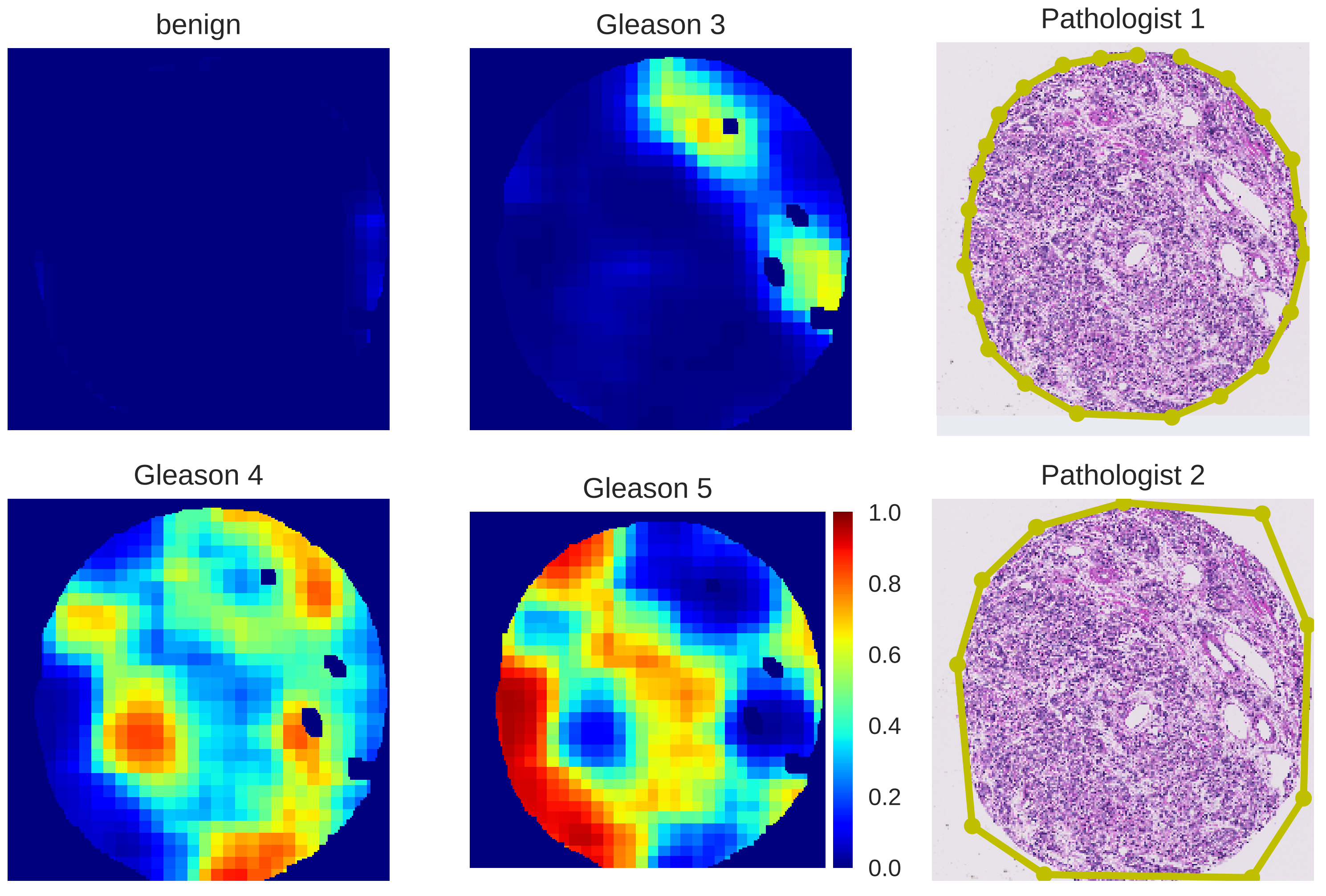
benign
Gleason 3
Pathologist 1
Gleason 4
Gleason 5
Pathologist 2
1.0
0.8
0.6
0.4
0.2
0.0

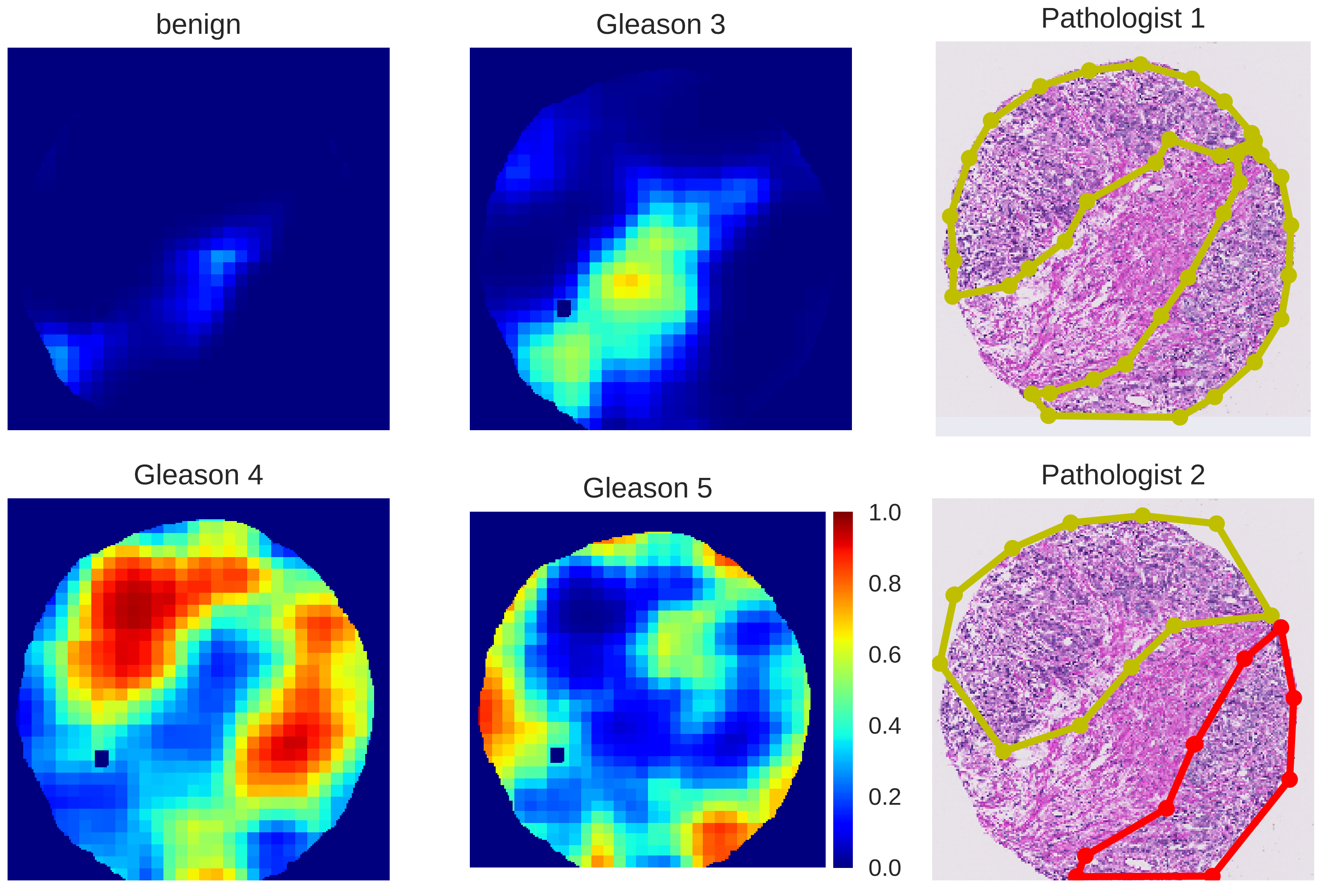

benign
Gleason 3
Pathologist 1
Gleason 4
Gleason 5
Pathologist 2
1.0
0.8
0.6
0.4
0.2
0.0

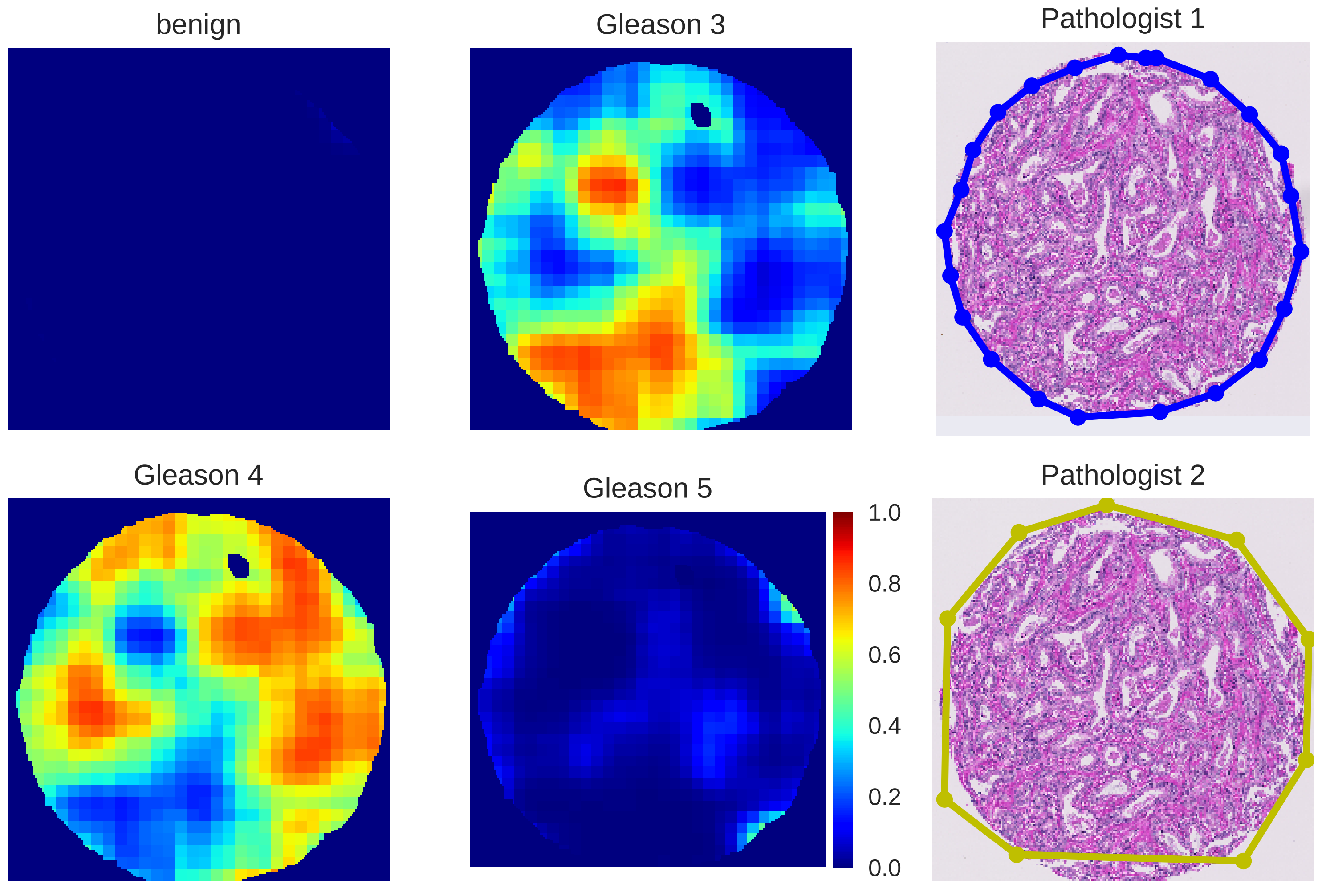
benign
Gleason 3
Pathologist 1
Gleason 4
Gleason 5
Pathologist 2
1.0
0.8
0.6
0.4
0.2
0.0

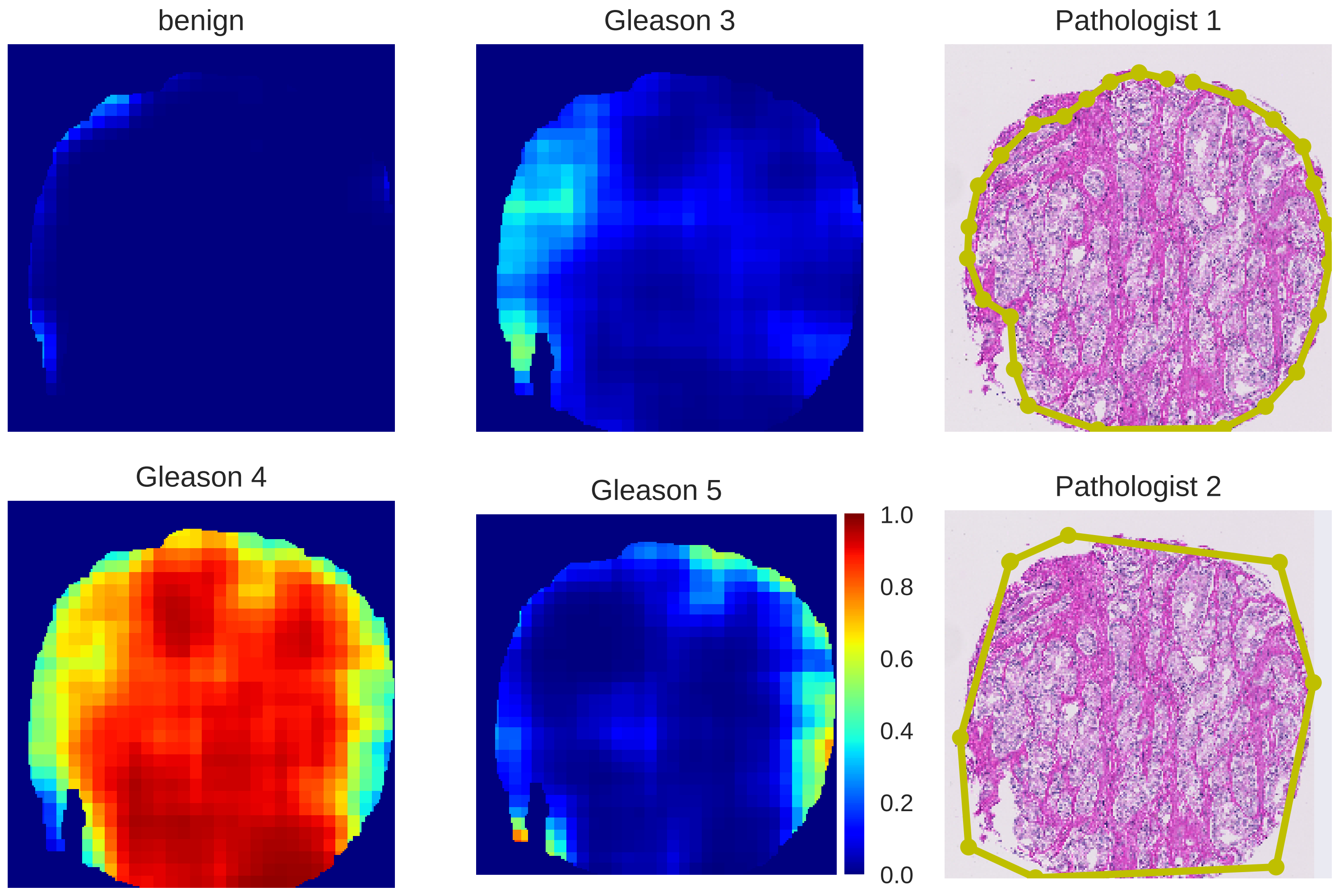
benign
Gleason 3
Pathologist 1
Gleason 4
Gleason 5
Pathologist 2
1.0
0.8
0.6
0.4
0.2
0.0

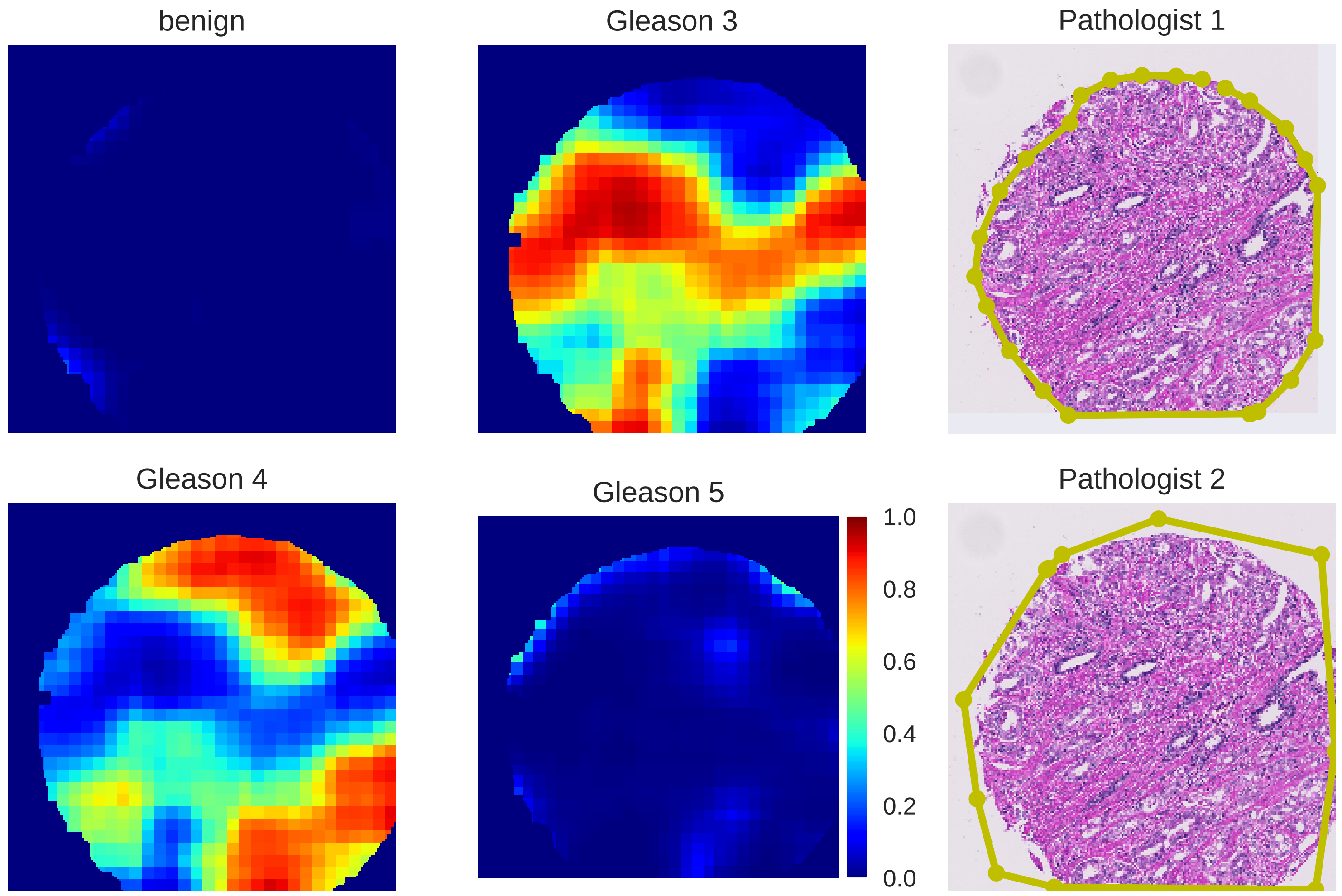

benign
Gleason 3
Pathologist 1
Gleason 4
Gleason 5
Pathologist 2
1.0
0.8
0.6
0.4
0.2
0.0

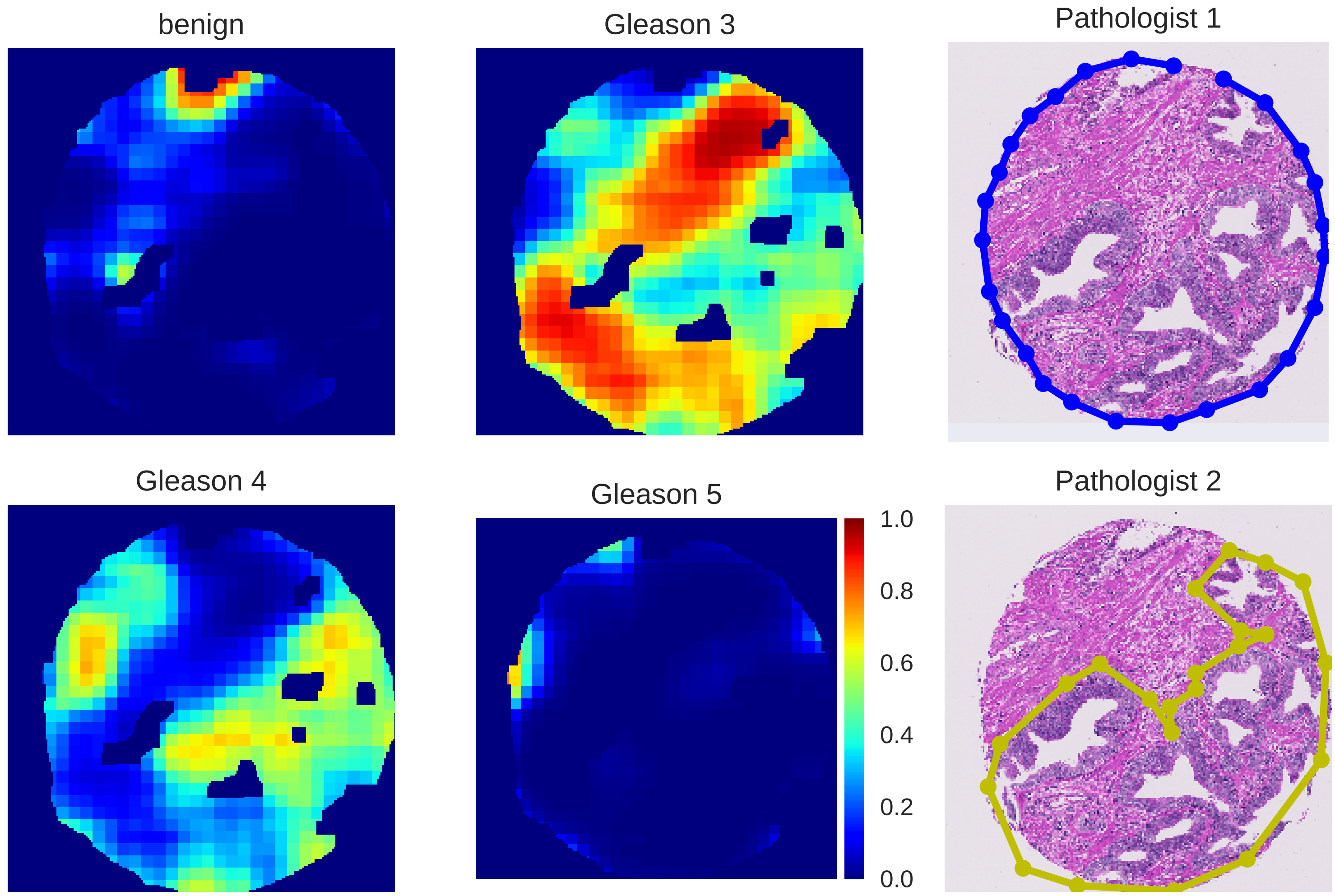

benign
Gleason 3
Pathologist 1
Gleason 4
Gleason 5
Pathologist 2
1.0
0.8
0.6
0.4
0.2
0.0

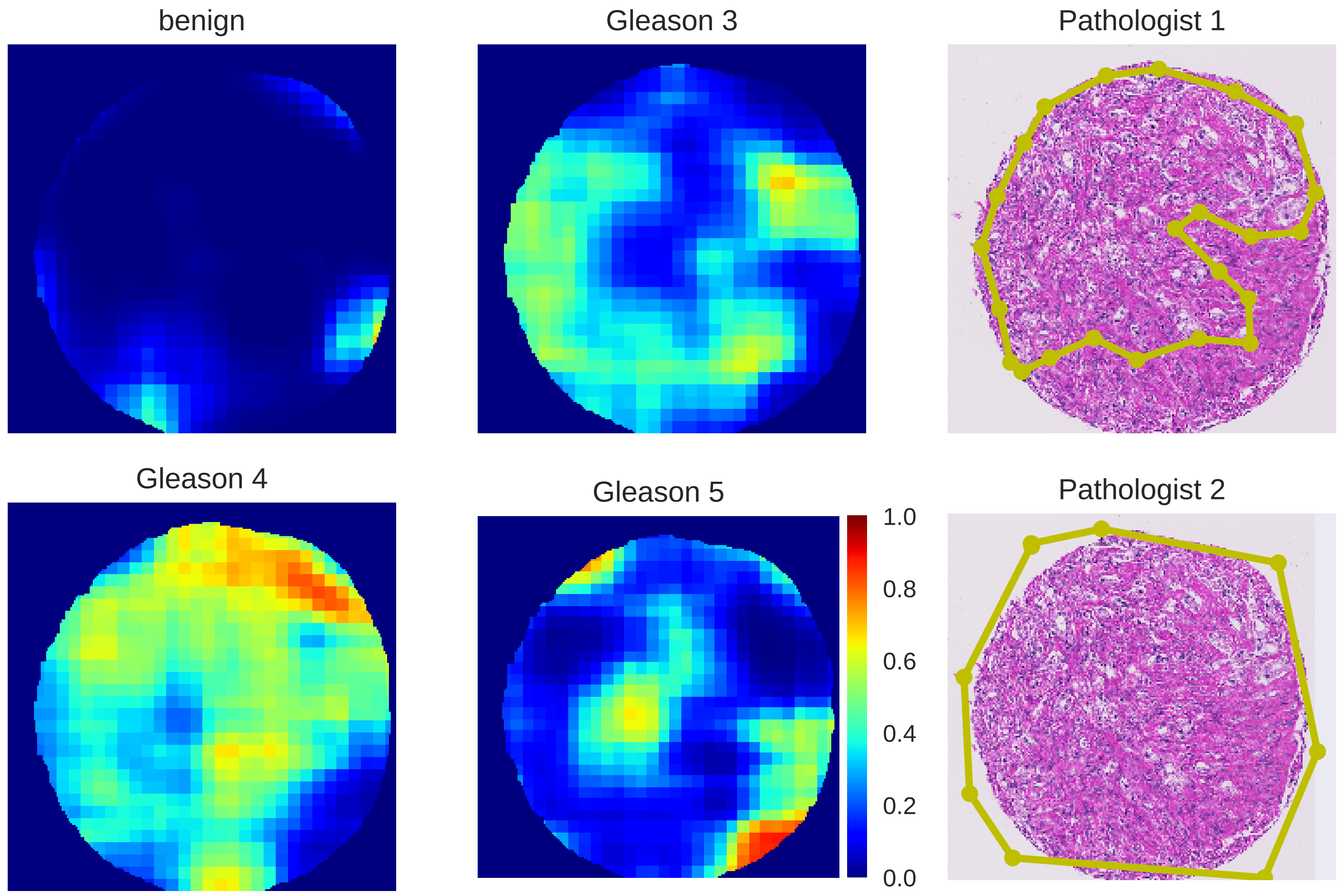
benign
Gleason 3
Pathologist 1
Gleason 4
Gleason 5
Pathologist 2
1.0
0.8
0.6
0.4
0.2
0.0

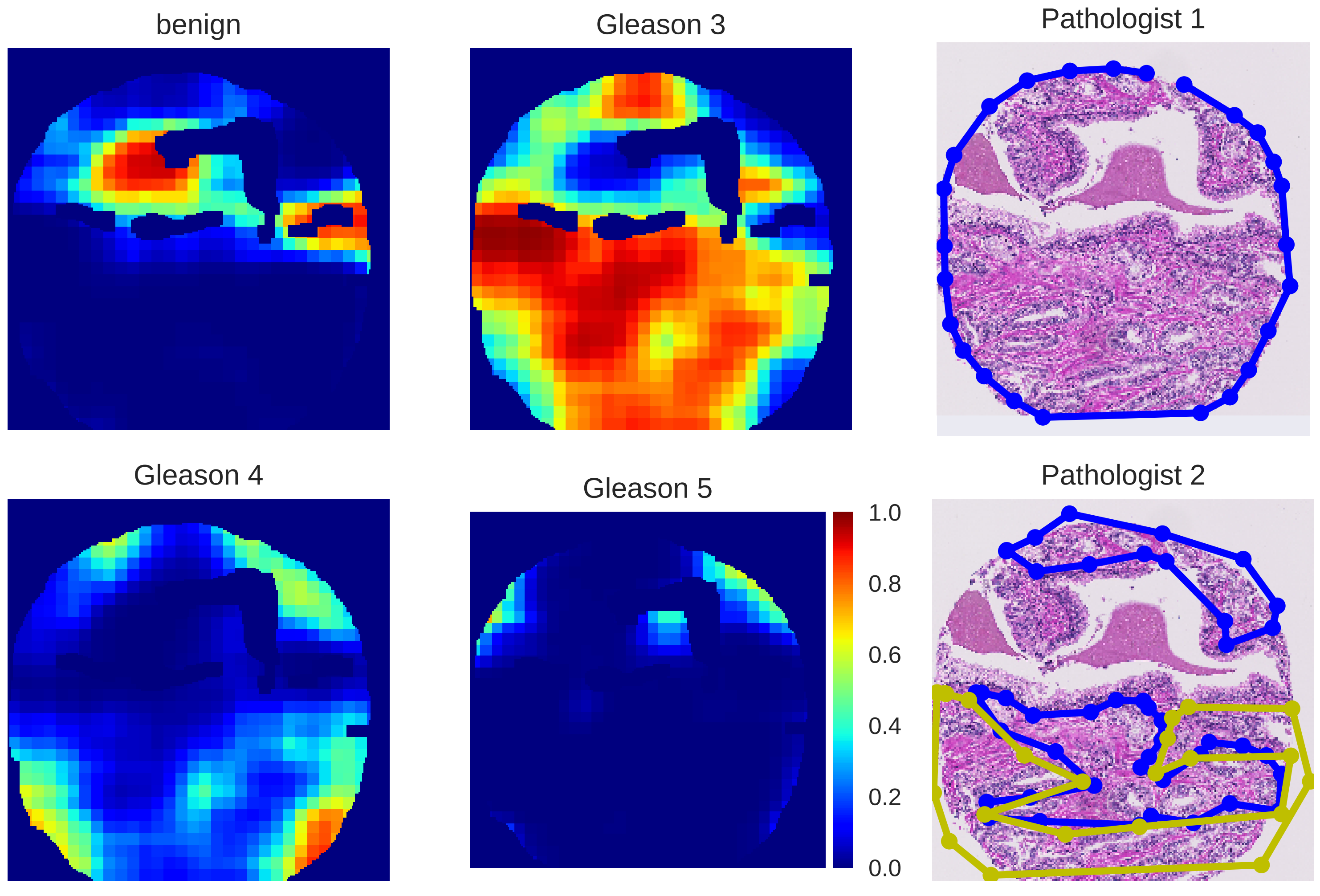

benign
Gleason 3
Pathologist 1
Gleason 4
Gleason 5
Pathologist 2
1.0
0.8
0.6
0.4
0.2
0.0

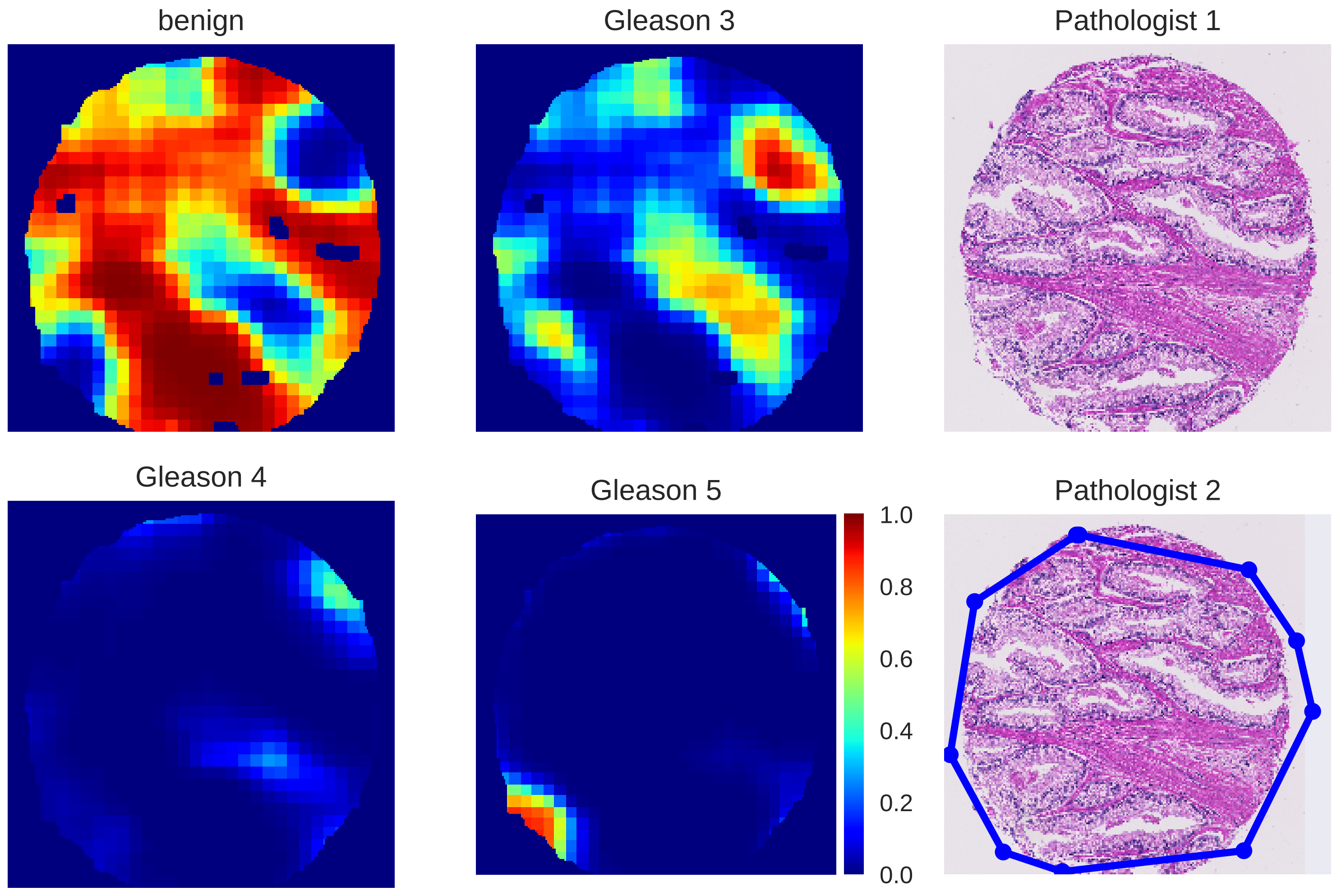

benign
Gleason 3
Pathologist 1
Gleason 4
Gleason 5
Pathologist 2
1.0
0.8
0.6
0.4
0.2
0.0

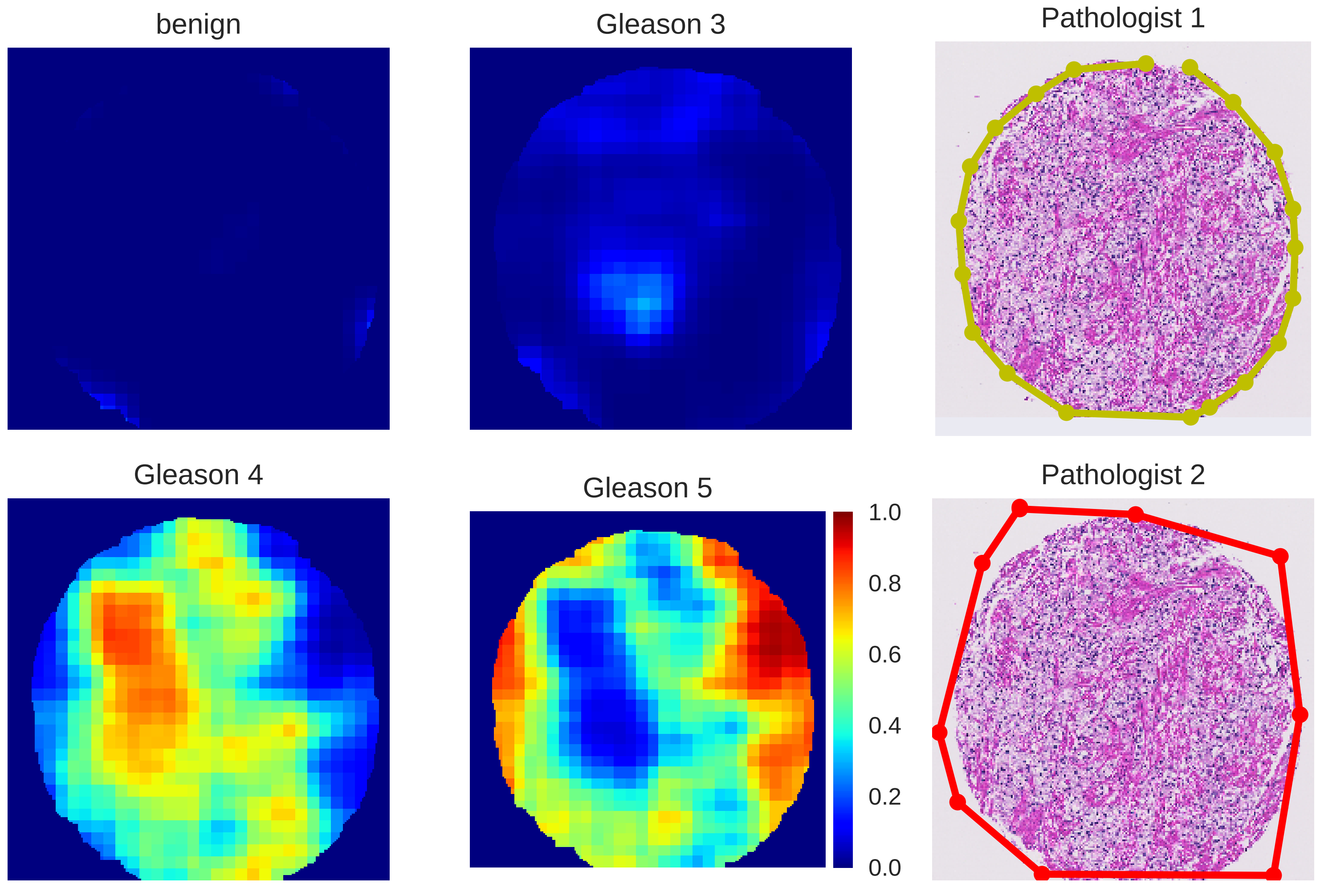

benign
Gleason 3
Pathologist 1
Gleason 4
Gleason 5
Pathologist 2
1.0
0.8
0.6
0.4
0.2
0.0

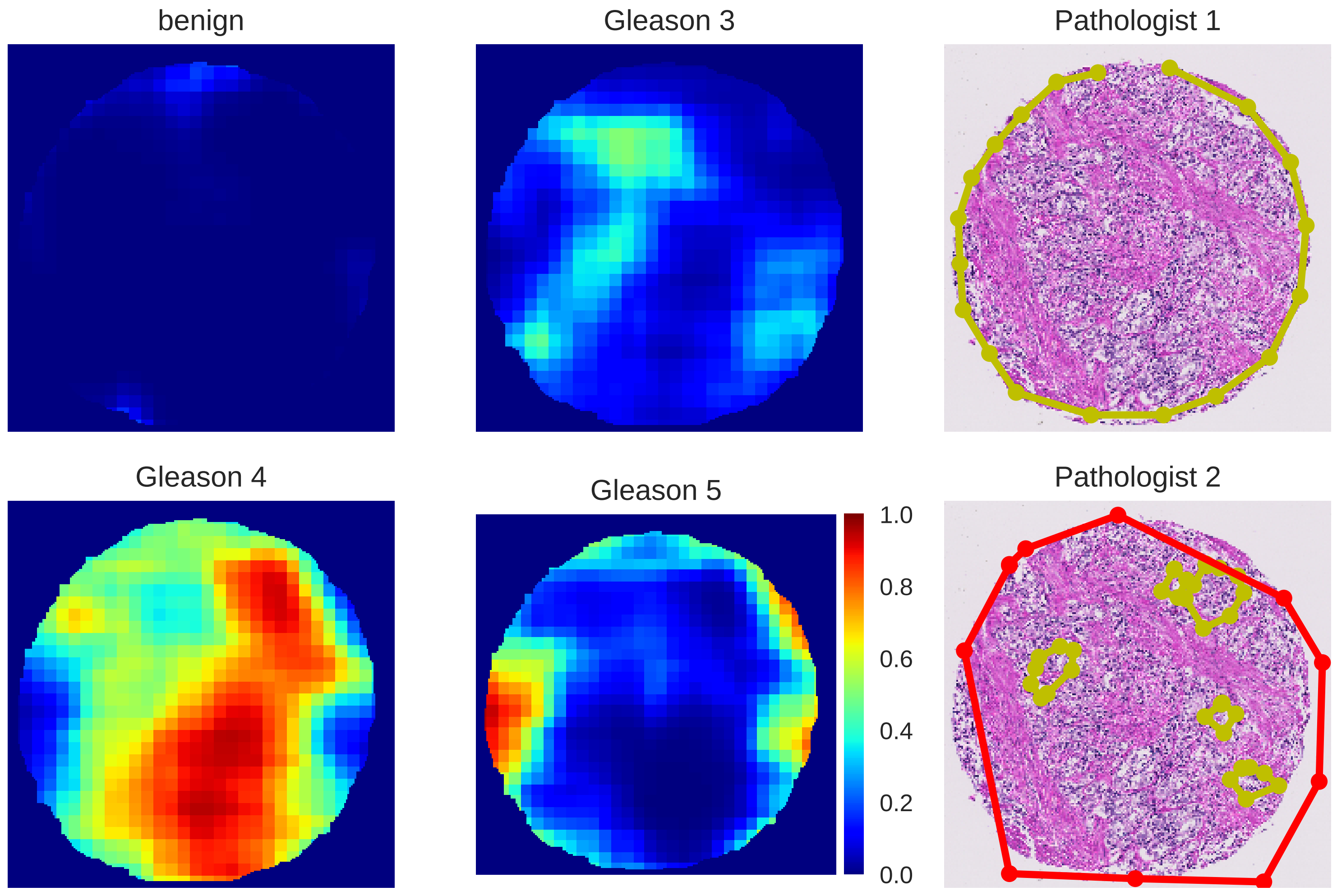

benign
Gleason 3
Pathologist 1
Gleason 4
Gleason 5
Pathologist 2
1.0
0.8
0.6
0.4
0.2
0.0

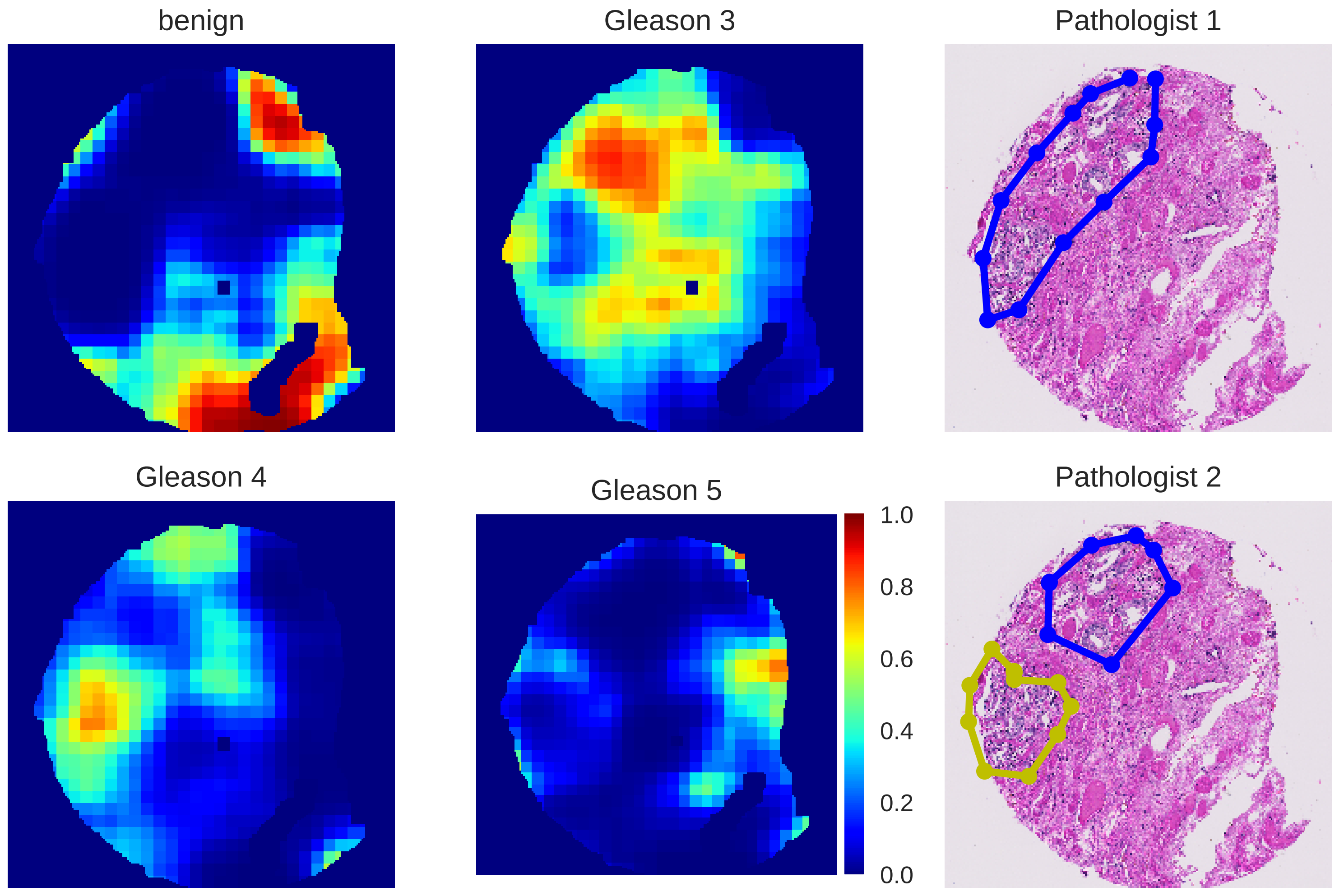

benign
Gleason 3
Pathologist 1
Gleason 4
Gleason 5
Pathologist 2
1.0
0.8
0.6
0.4
0.2
0.0

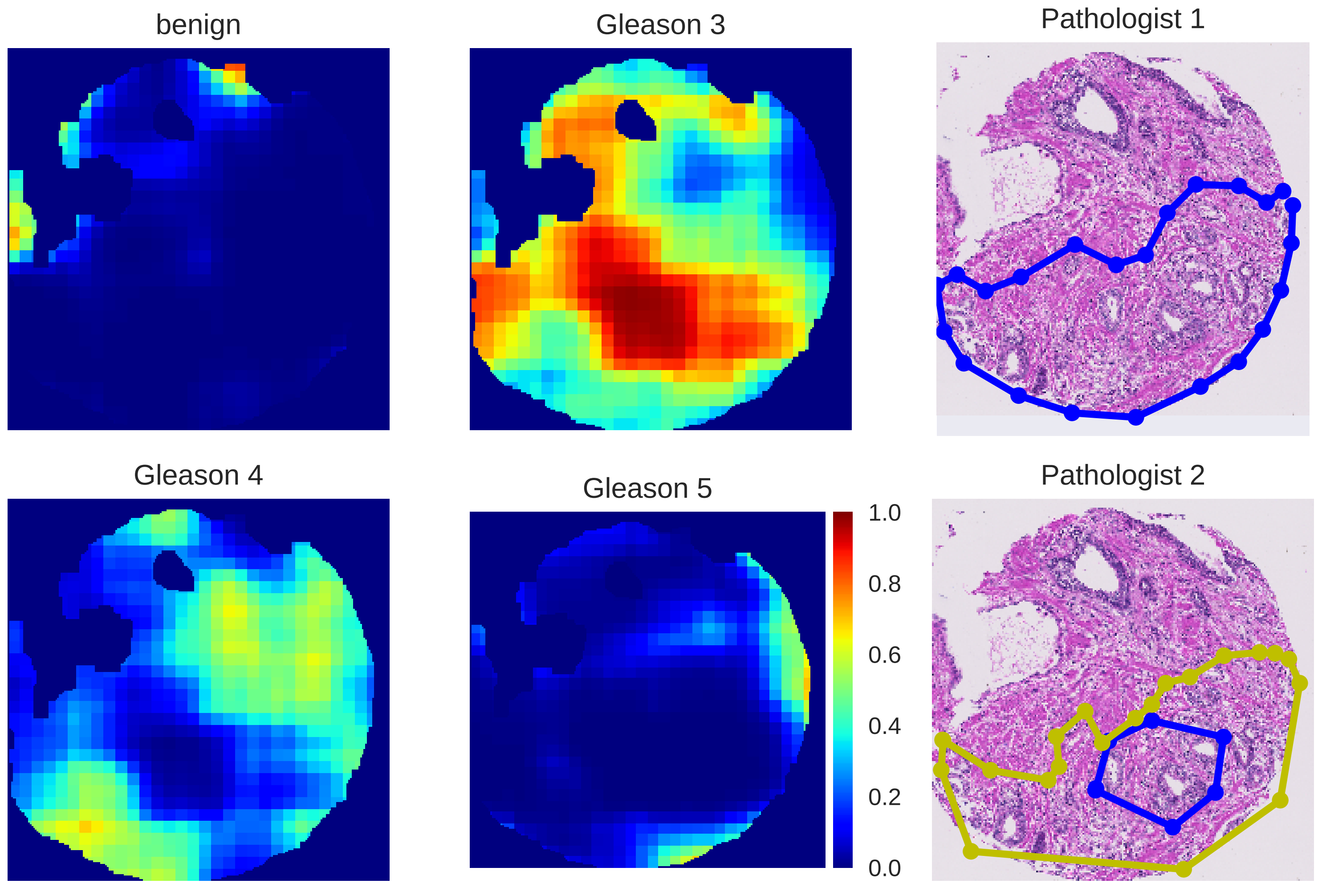
benign
Gleason 3
Pathologist 1
Gleason 4
Gleason 5
Pathologist 2
1.0
0.8
0.6
0.4
0.2
0.0

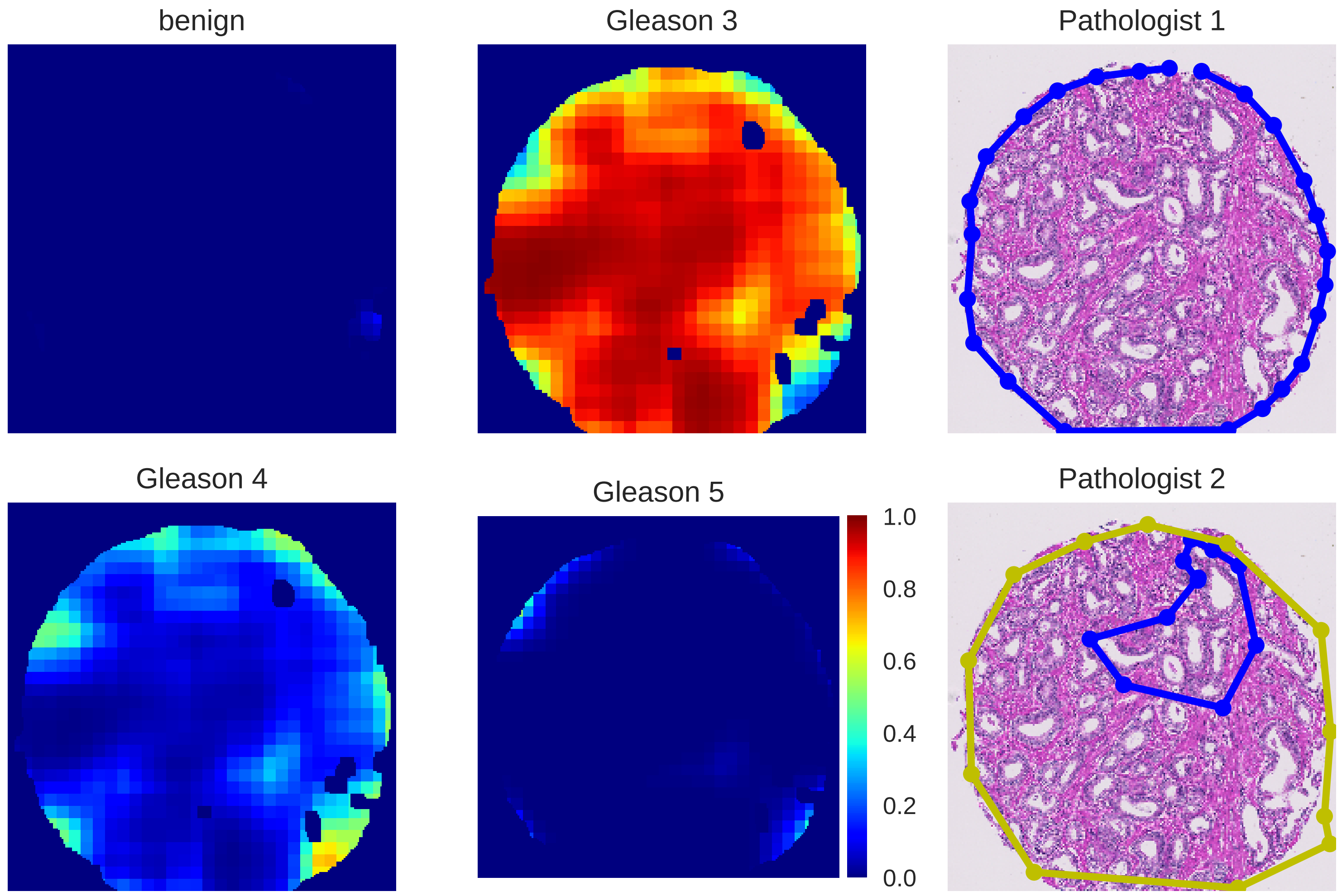
benign
Gleason 3
Pathologist 1
Gleason 4
Gleason 5
Pathologist 2
1.0
0.8
0.6
0.4
0.2
0.0

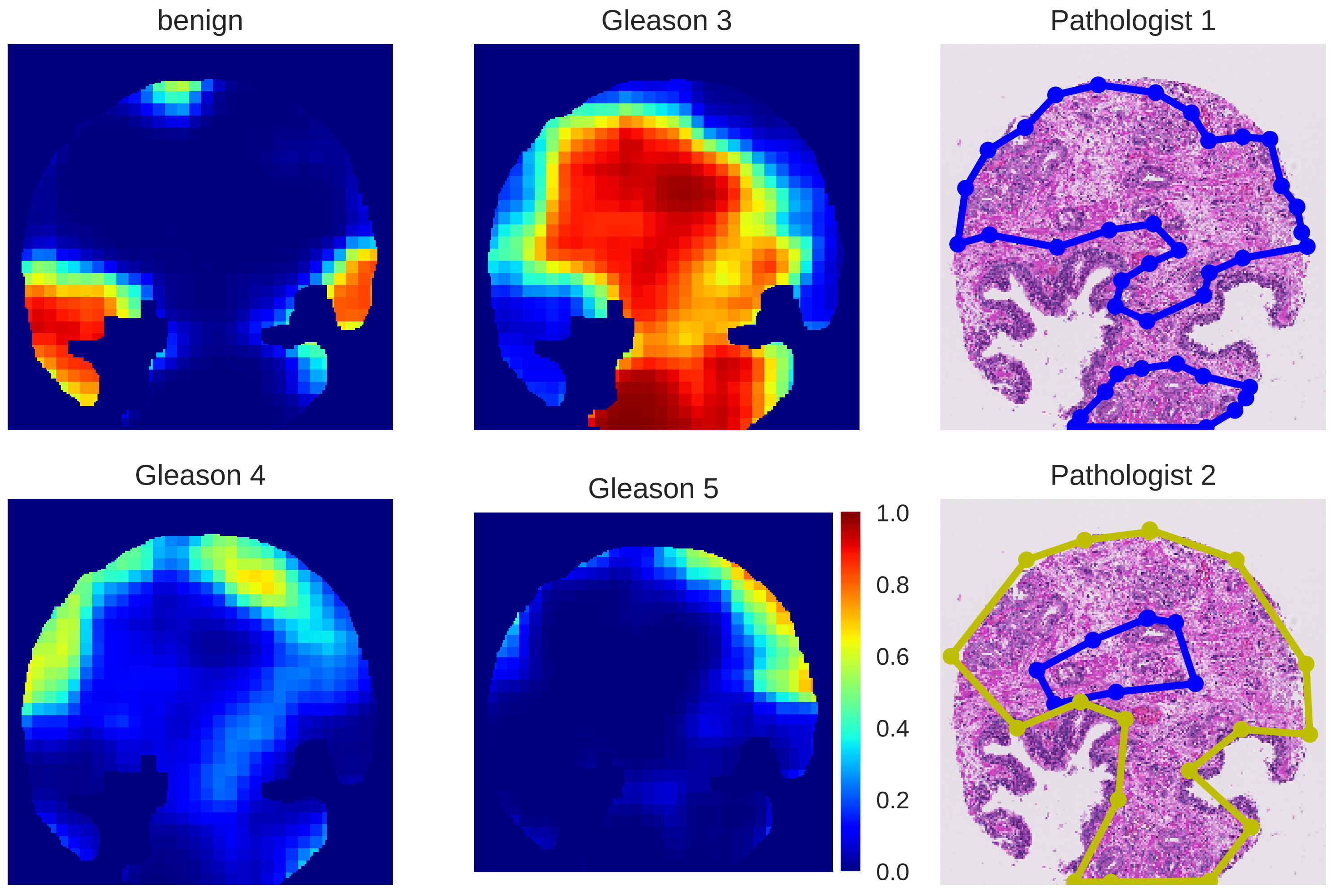

benign
Gleason 3
Pathologist 1
Gleason 4
Gleason 5
Pathologist 2
1.0
0.8
0.6
0.4
0.2
0.0

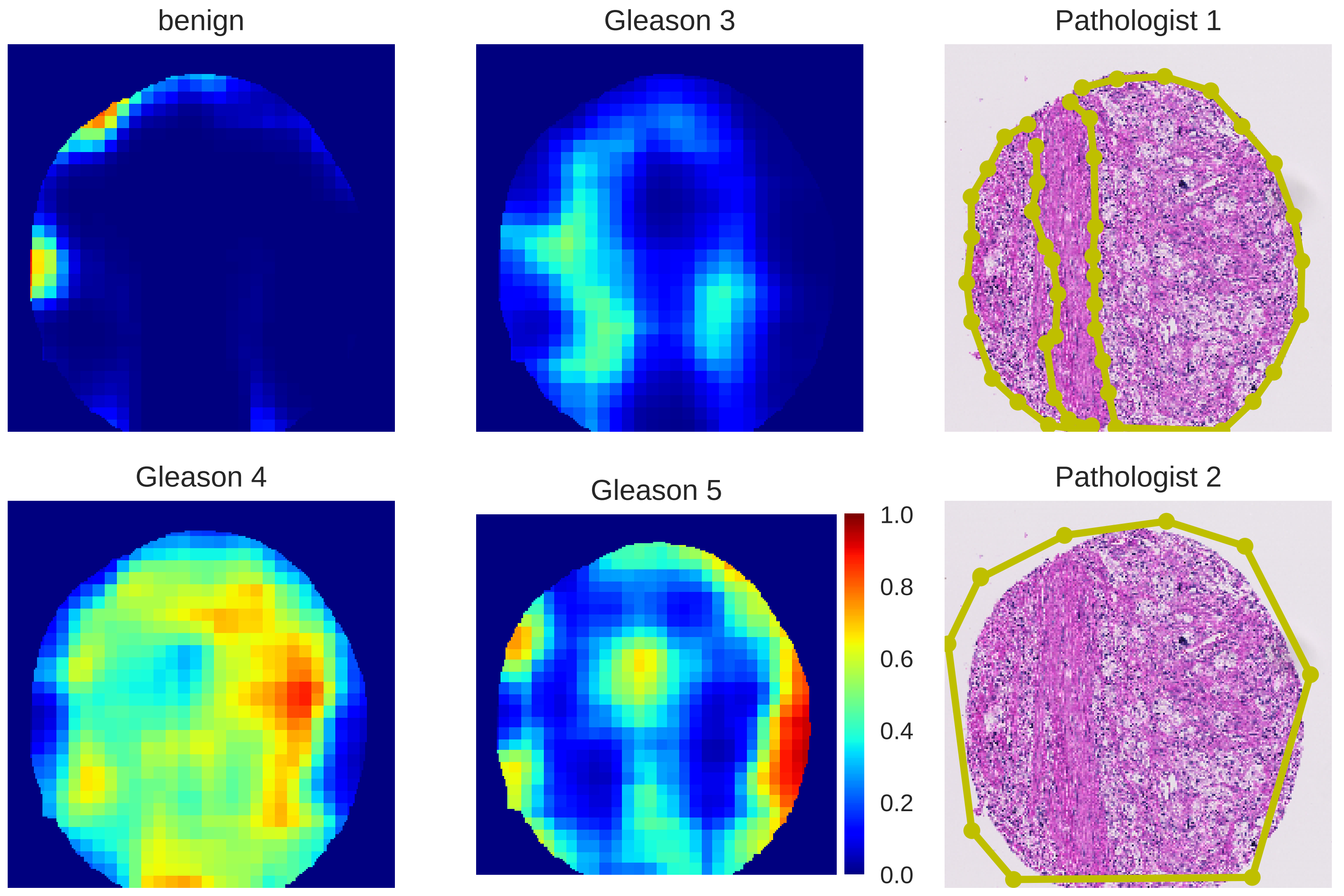
benign
Gleason 3
Pathologist 1
Gleason 4
Gleason 5
Pathologist 2
1.0
0.8
0.6
0.4
0.2
0.0

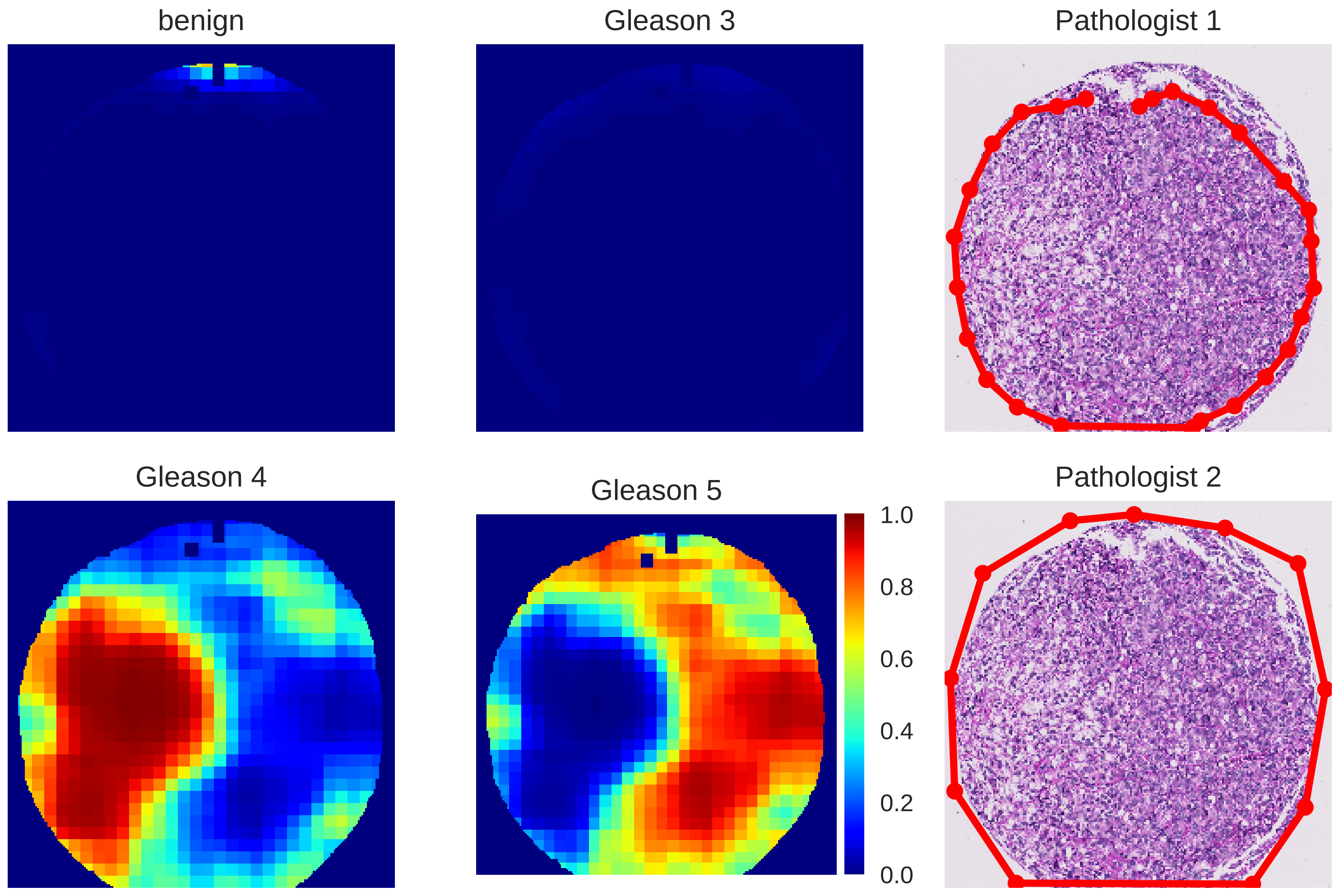
benign
Gleason 3
Pathologist 1
Gleason 4
Gleason 5
Pathologist 2
1.0
0.8
0.6
0.4
0.2
0.0

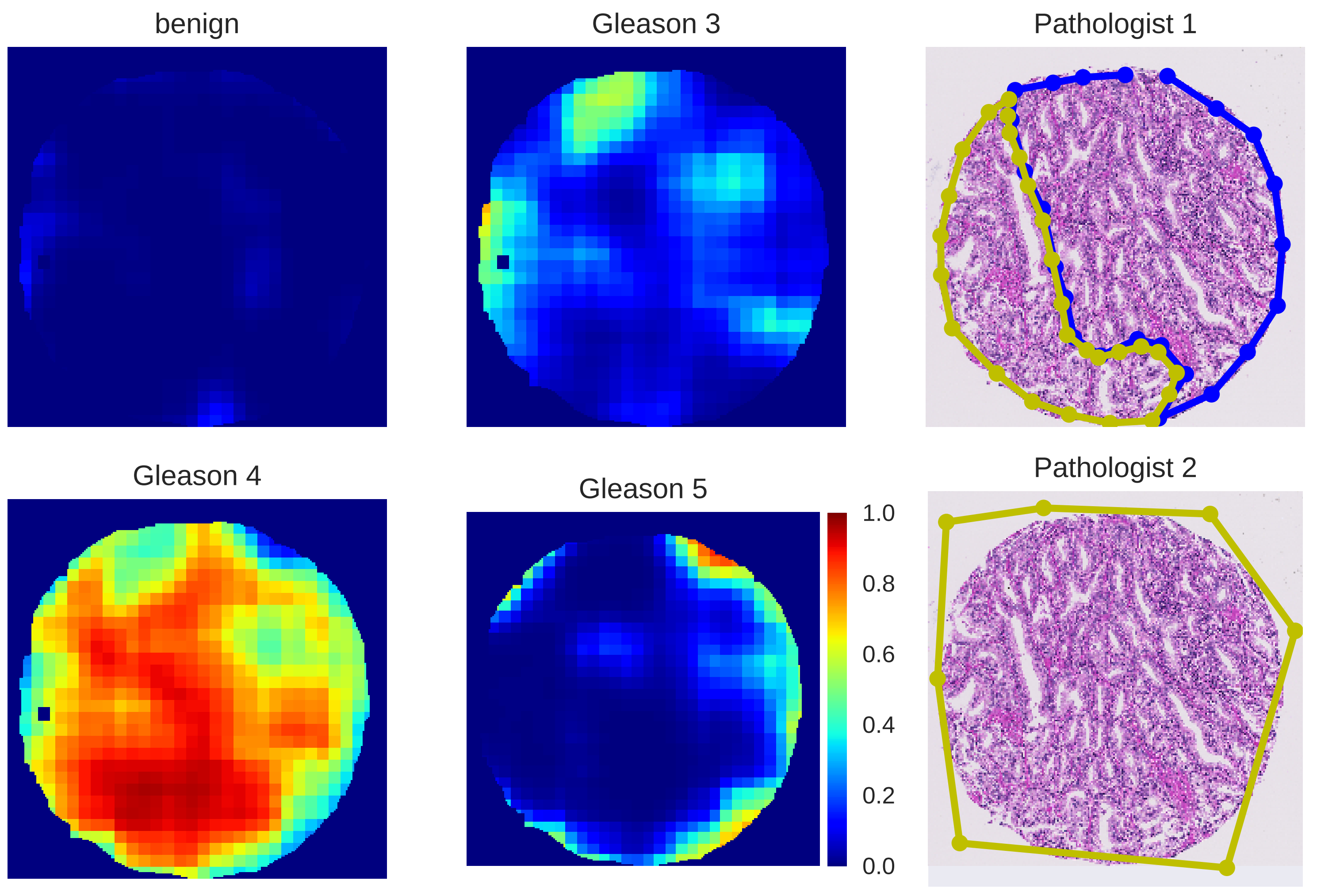

benign
Gleason 3
Pathologist 1
Gleason 4
Gleason 5
Pathologist 2
1.0
0.8
0.6
0.4
0.2
0.0

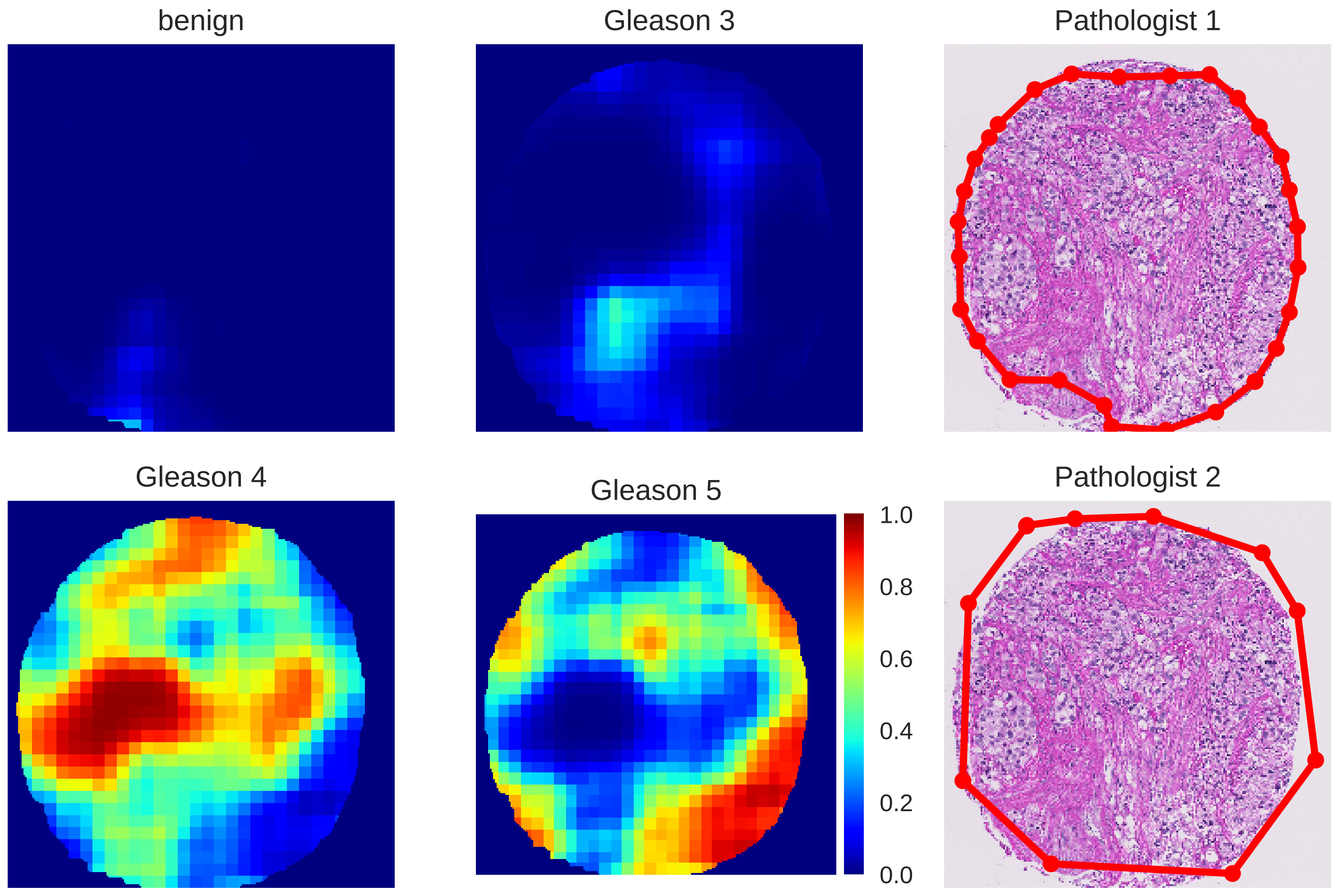

benign
Gleason 3
Pathologist 1
Gleason 4
Gleason 5
Pathologist 2
1.0
0.8
0.6
0.4
0.2
0.0

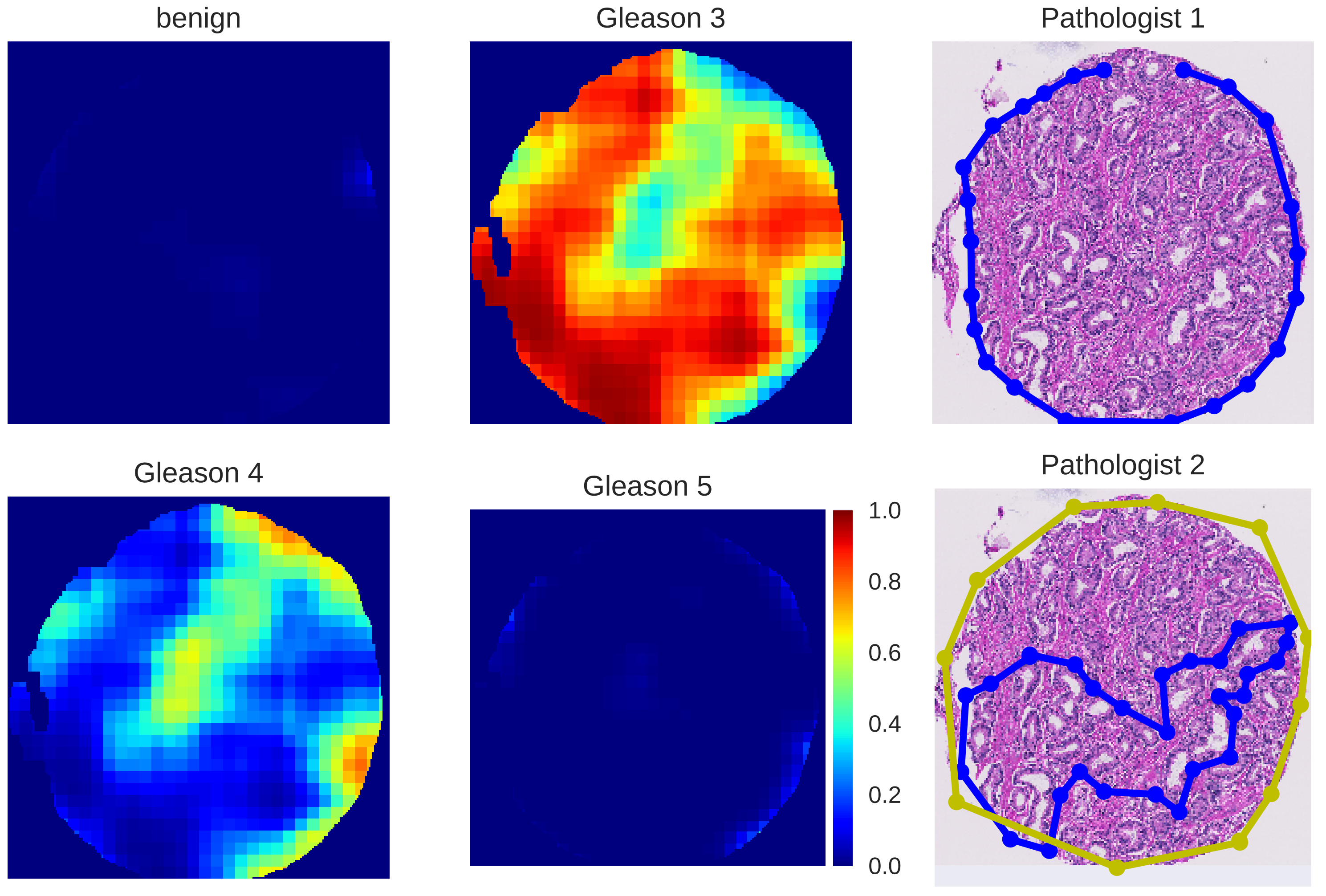

benign
Gleason 3
Pathologist 1
Gleason 4
Gleason 5
Pathologist 2
1.0
0.8
0.6
0.4
0.2
0.0

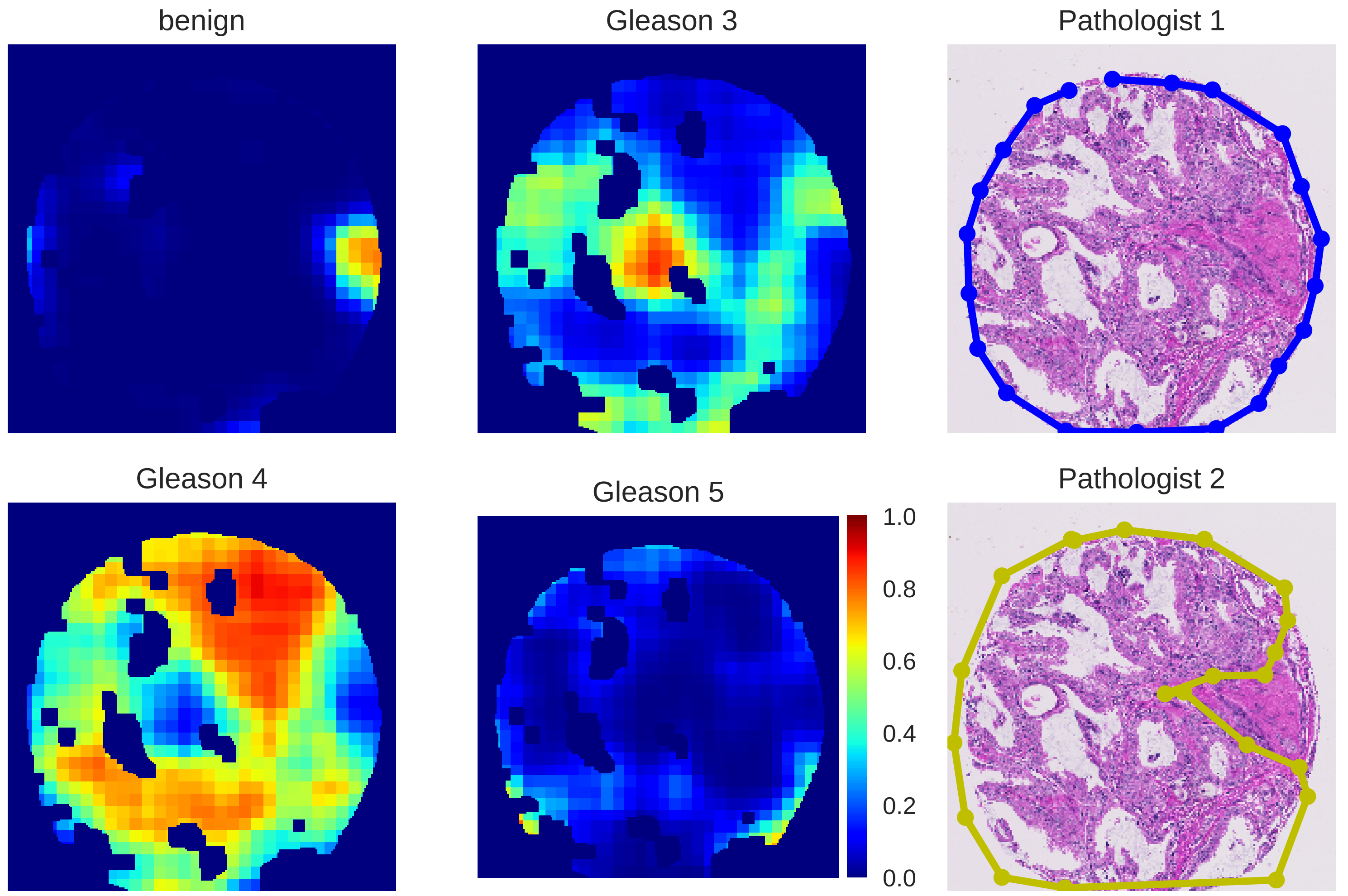
benign
Gleason 3
Pathologist 1
Gleason 4
Gleason 5
Pathologist 2
1.0
0.8
0.6
0.4
0.2
0.0

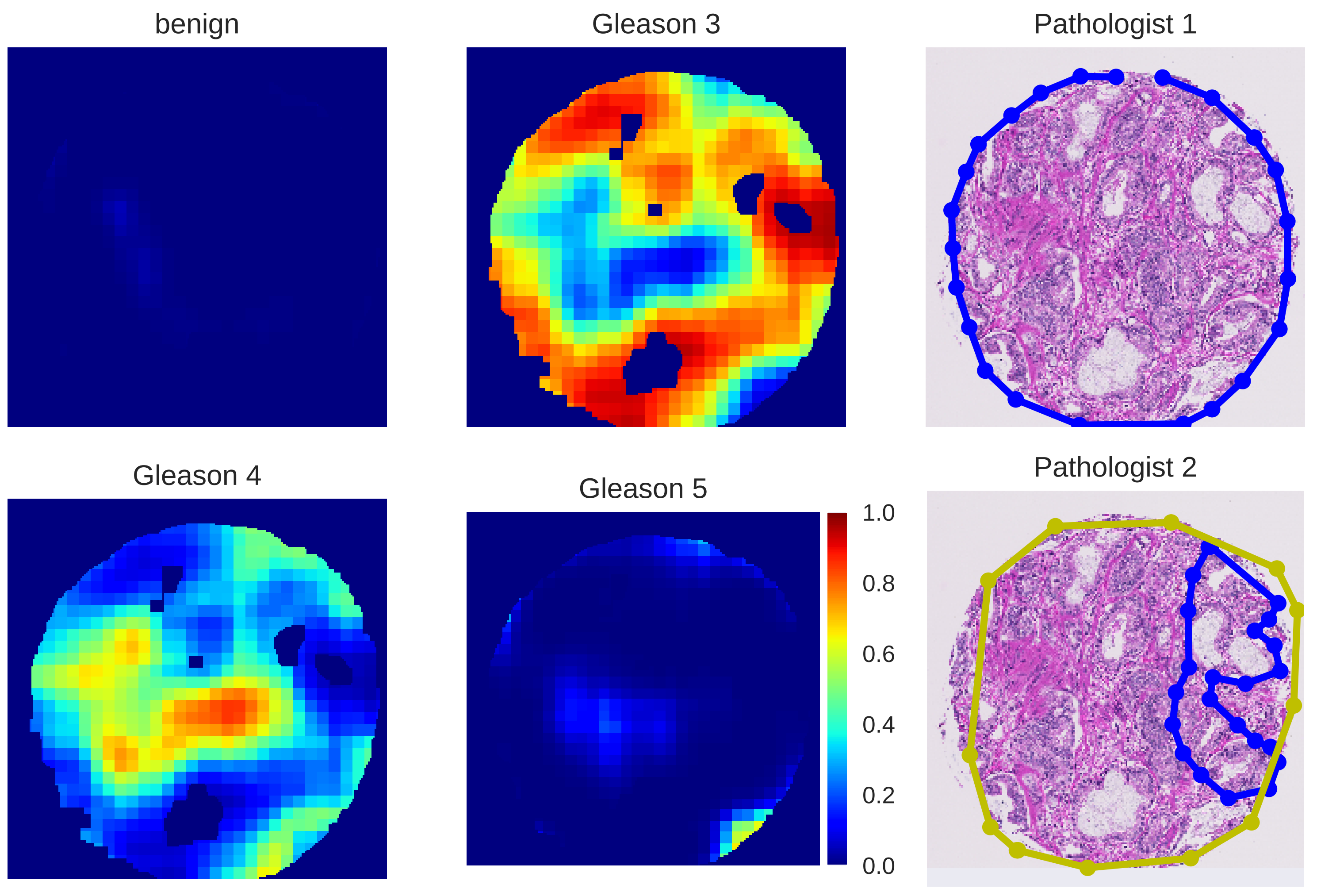
benign
Gleason 3
Pathologist 1
Gleason 4
Gleason 5
Pathologist 2
1.0
0.8
0.6
0.4
0.2
0.0

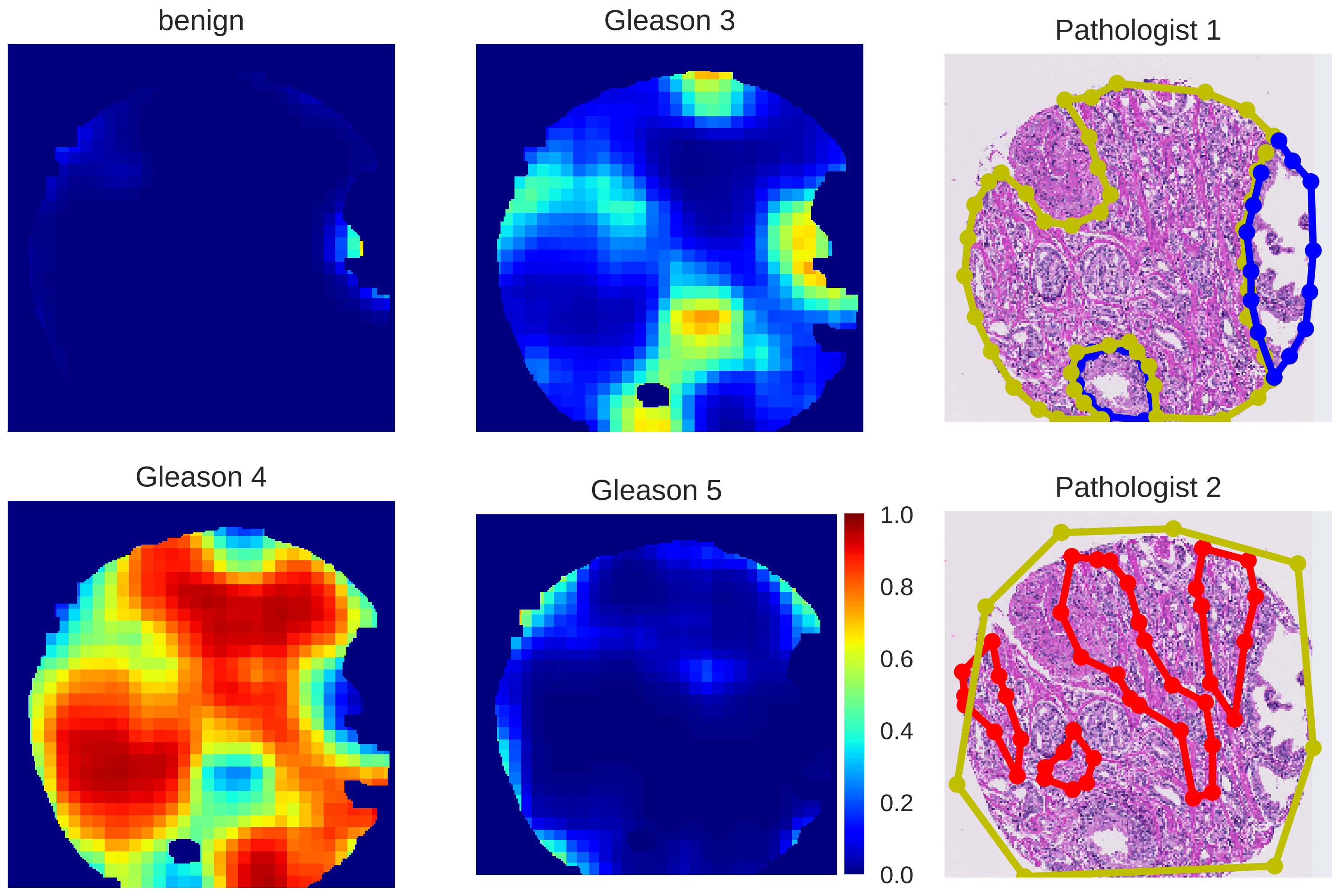

benign
Gleason 3
Pathologist 1
Gleason 4
Gleason 5
Pathologist 2
1.0
0.8
0.6
0.4
0.2
0.0

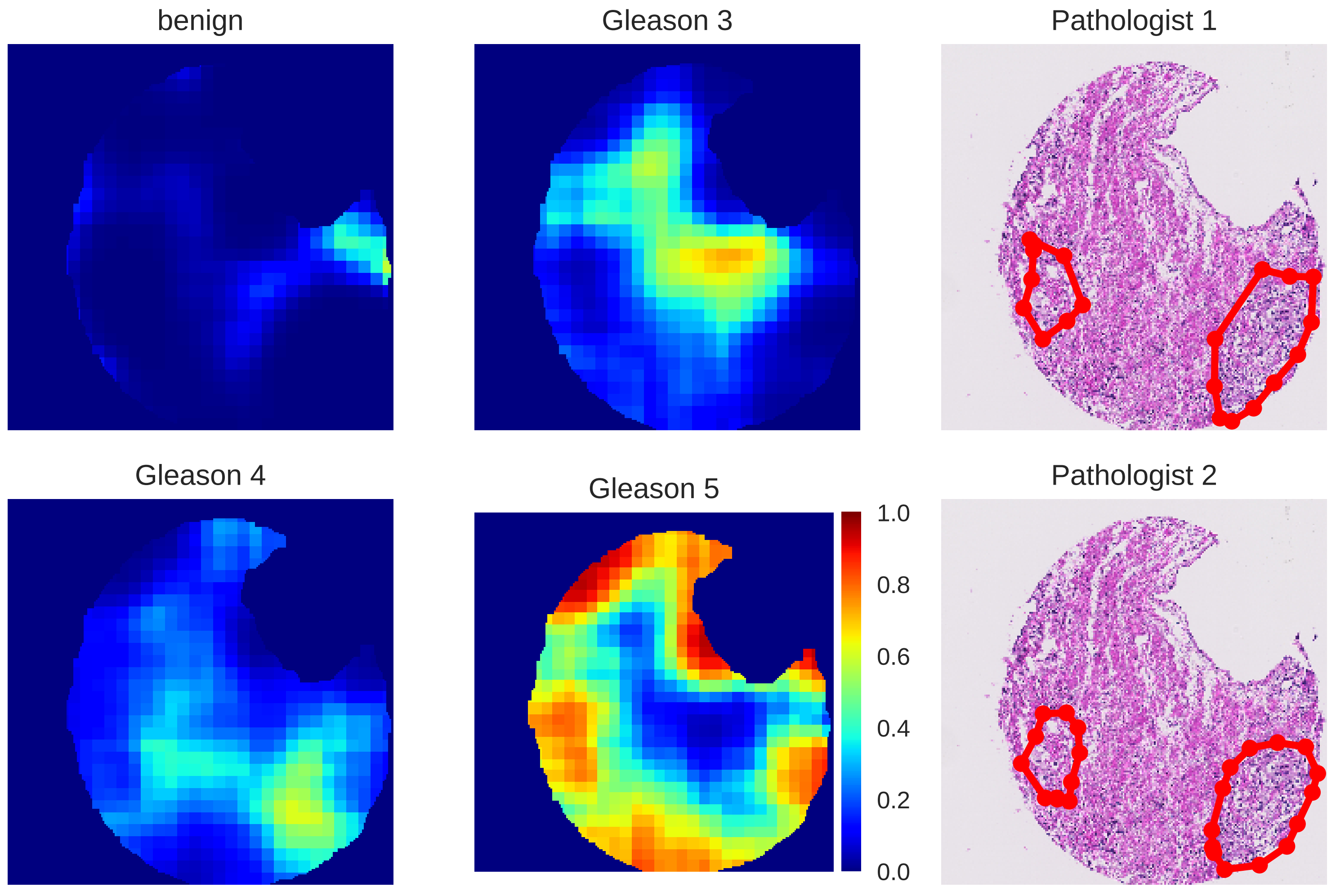
benign
Gleason 3
Pathologist 1
Gleason 4
Gleason 5
Pathologist 2
1.0
0.8
0.6
0.4
0.2
0.0

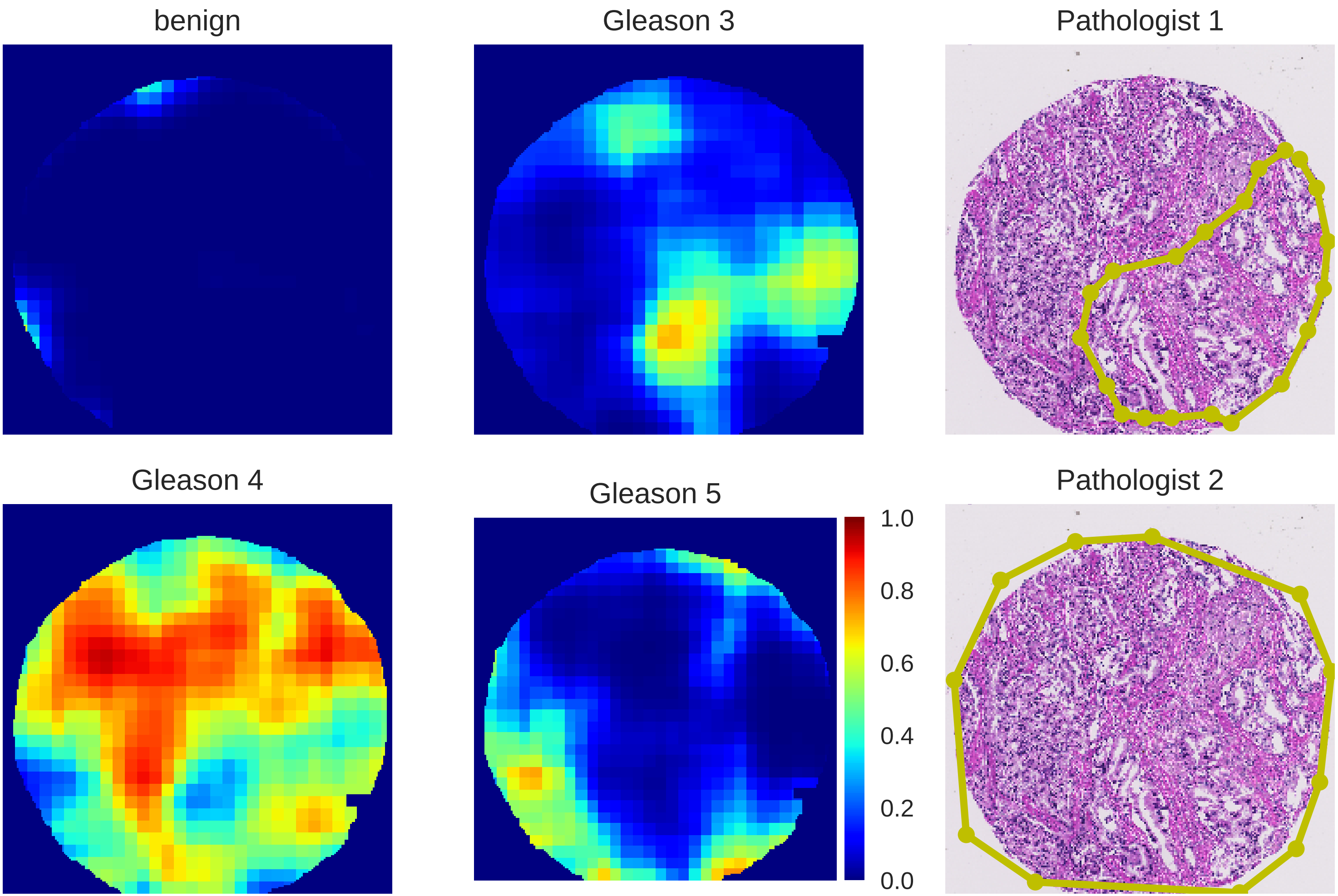

benign
Gleason 3
Pathologist 1
Gleason 4
Gleason 5
Pathologist 2
1.0
0.8
0.6
0.4
0.2
0.0

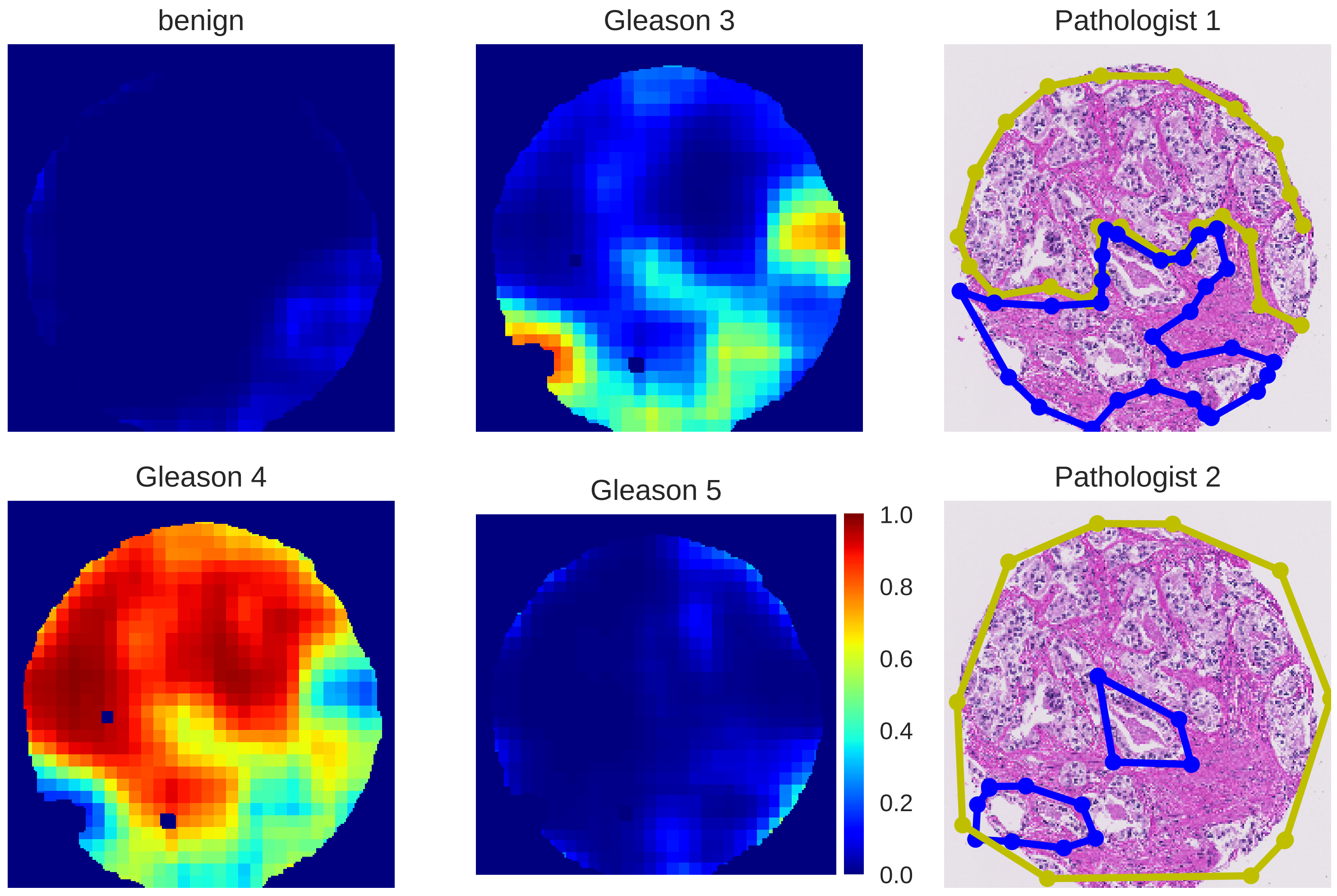
benign
Gleason 3
Pathologist 1
Gleason 4
Gleason 5
Pathologist 2
1.0
0.8
0.6
0.4
0.2
0.0

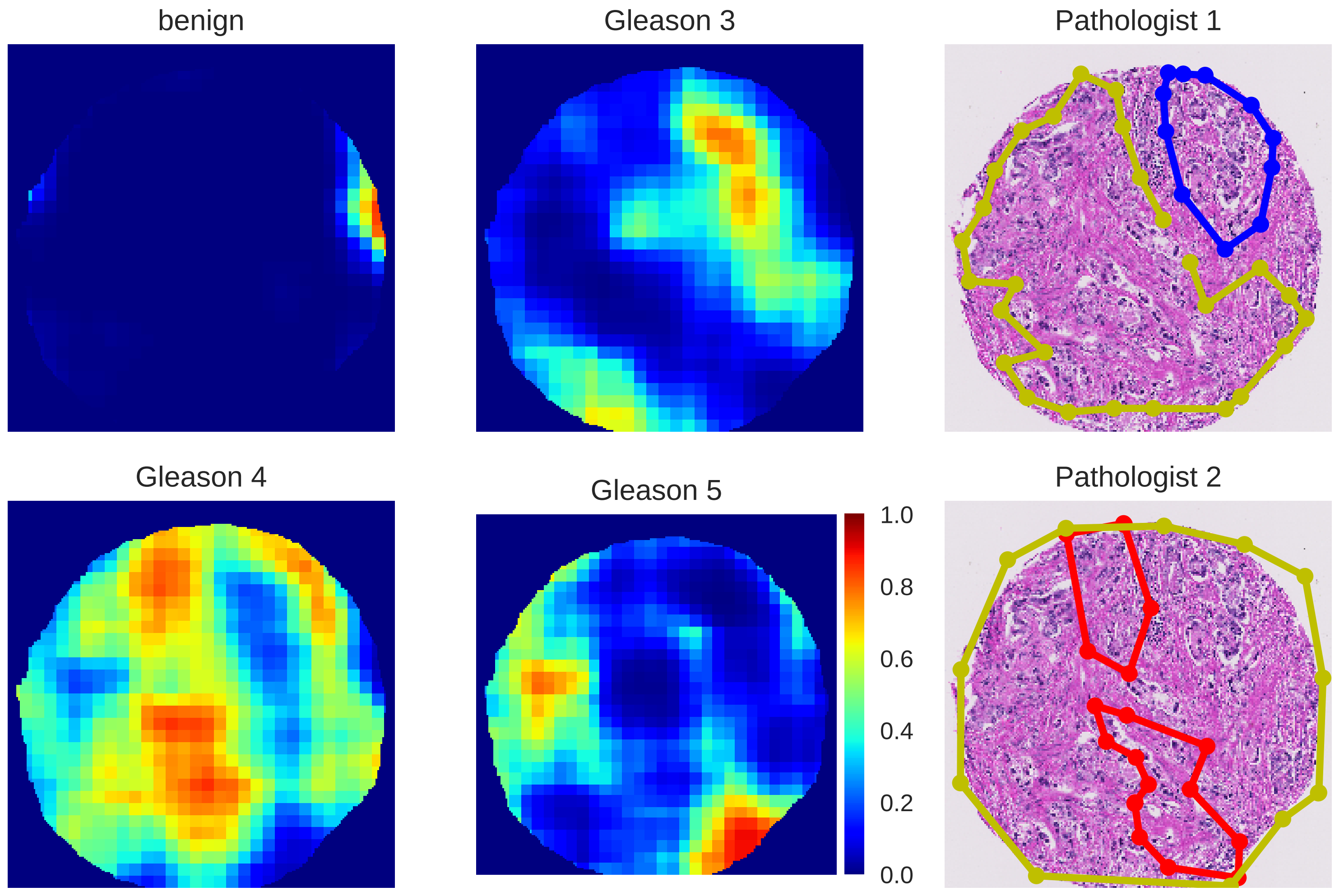

benign
Gleason 3
Pathologist 1
Gleason 4
Gleason 5
Pathologist 2
1.0
0.8
0.6
0.4
0.2
0.0

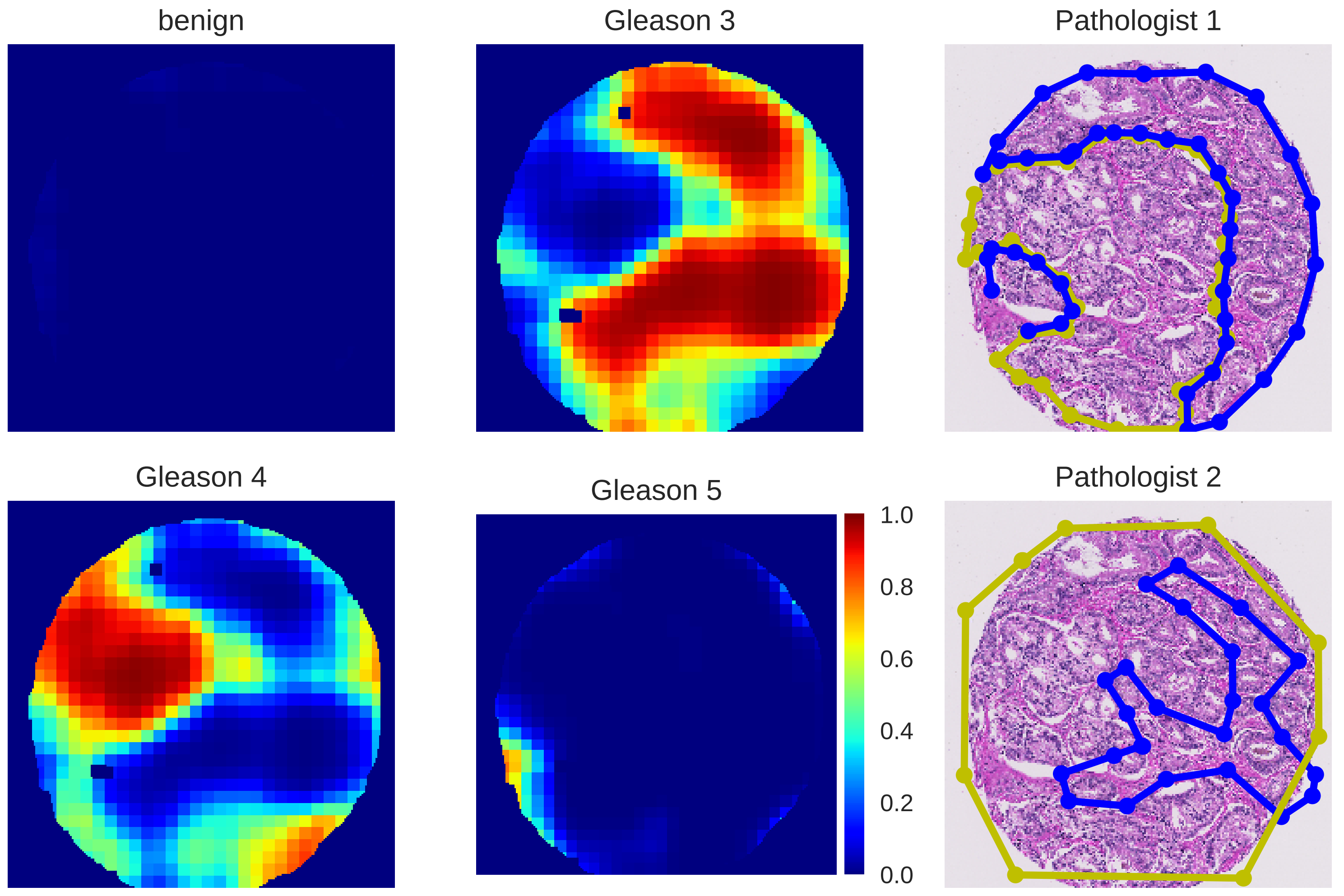
benign
Gleason 3
Pathologist 1
Gleason 4
Gleason 5
Pathologist 2
1.0
0.8
0.6
0.4
0.2
0.0

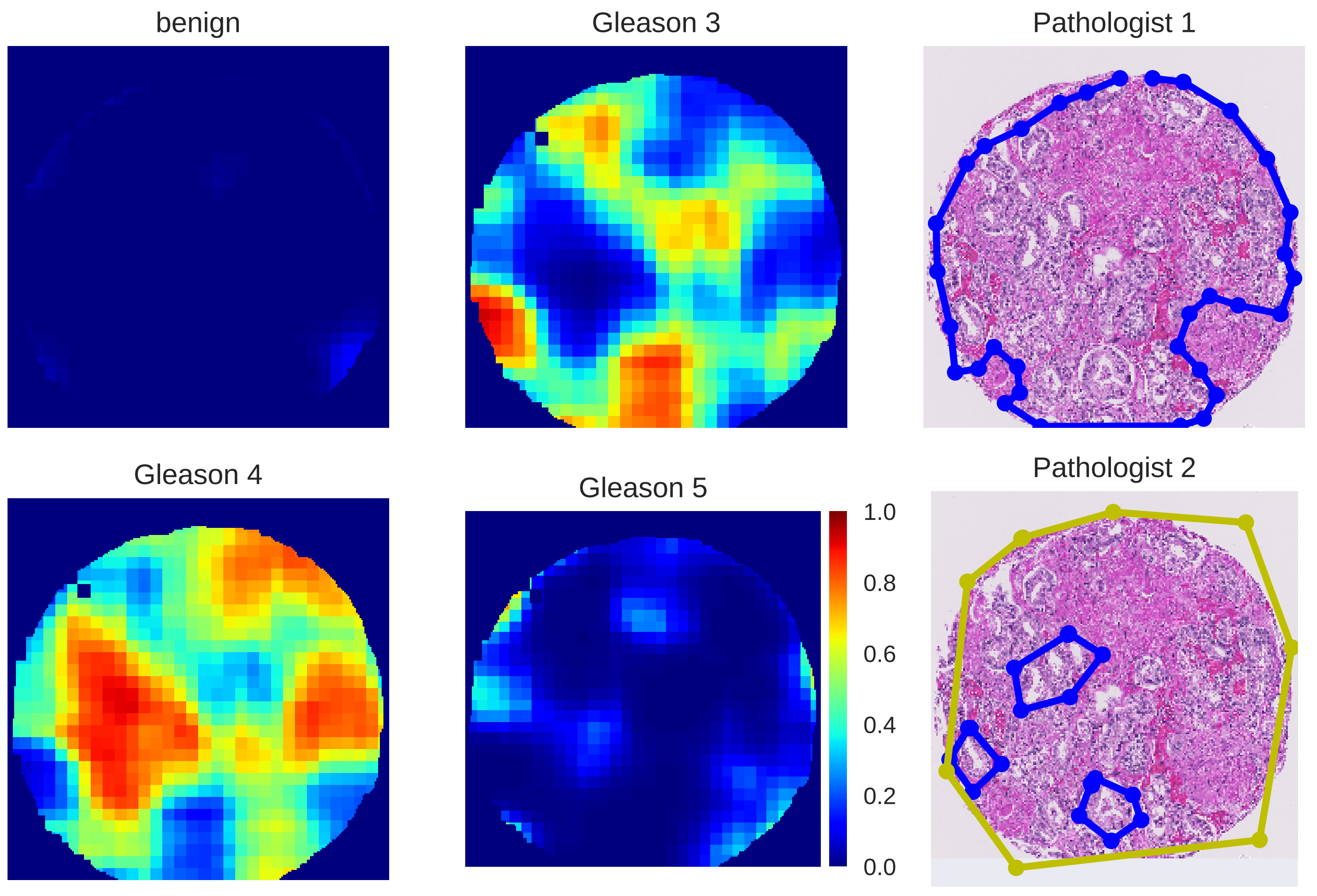

benign
Gleason 3
Pathologist 1
Gleason 4
Gleason 5
Pathologist 2
1.0
0.8
0.6
0.4
0.2
0.0

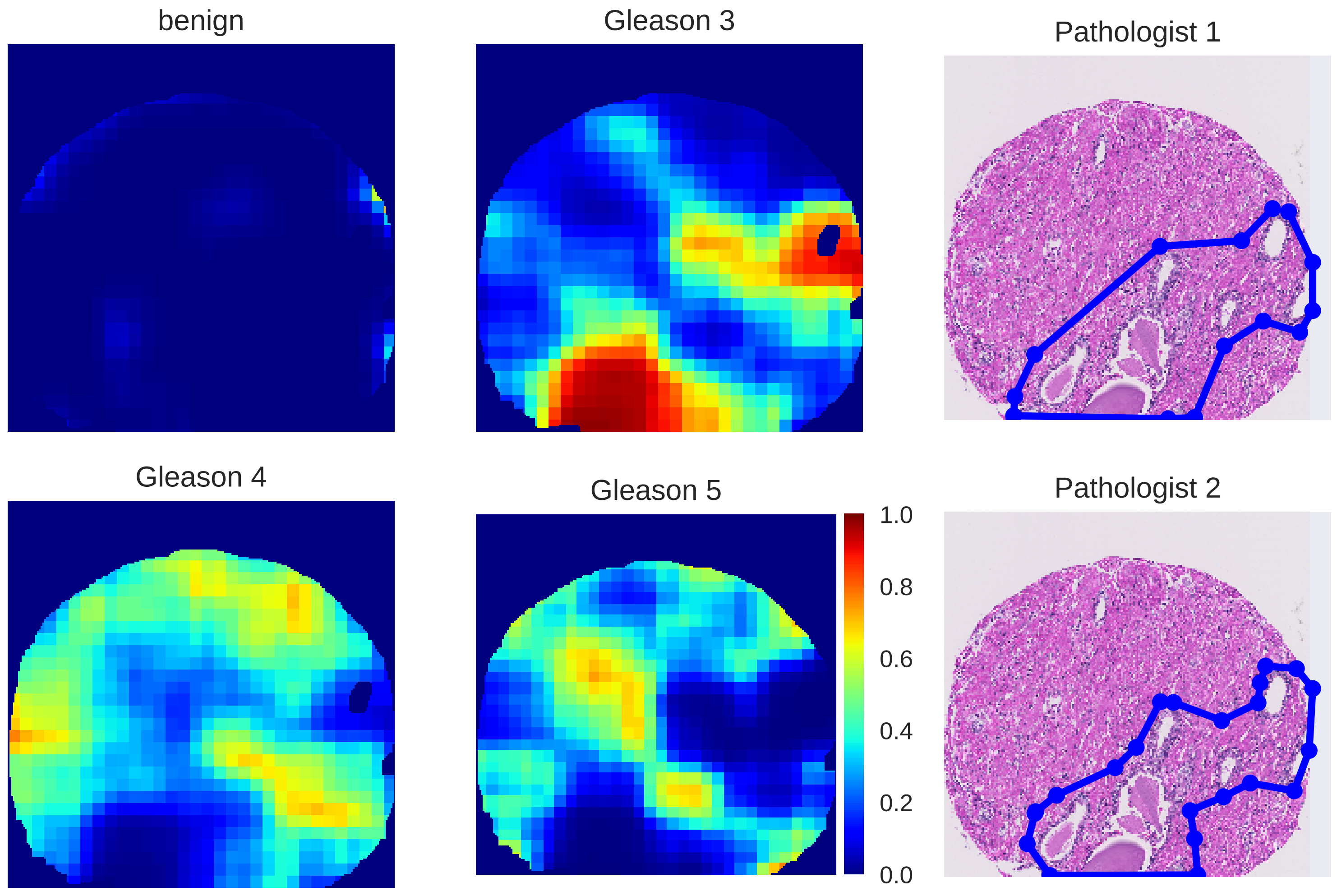
benign
Gleason 3
Pathologist 1
Gleason 4
Gleason 5
Pathologist 2
1.0
0.8
0.6
0.4
0.2
0.0

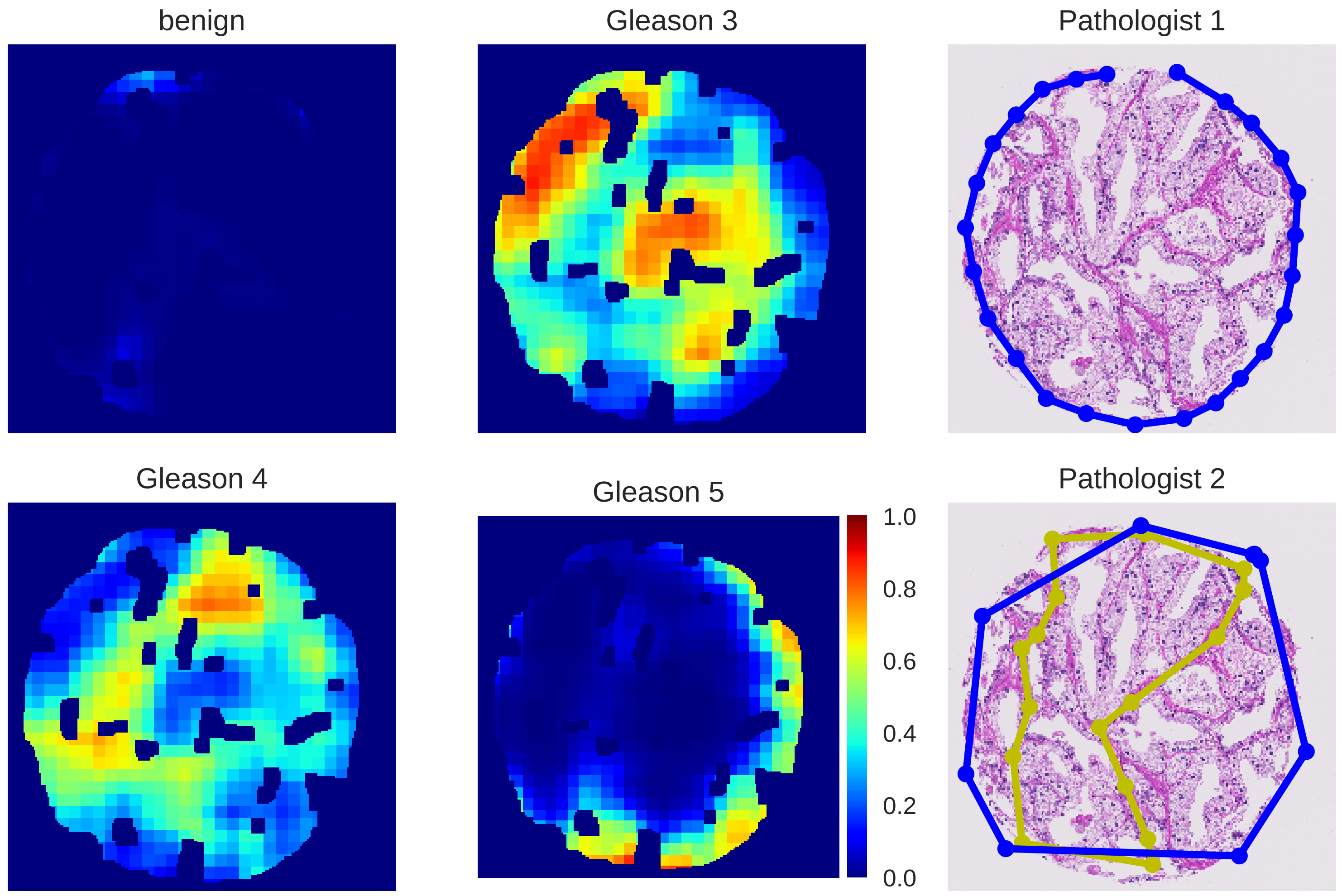

benign
Gleason 3
Pathologist 1
Gleason 4
Gleason 5
Pathologist 2
1.0
0.8
0.6
0.4
0.2
0.0

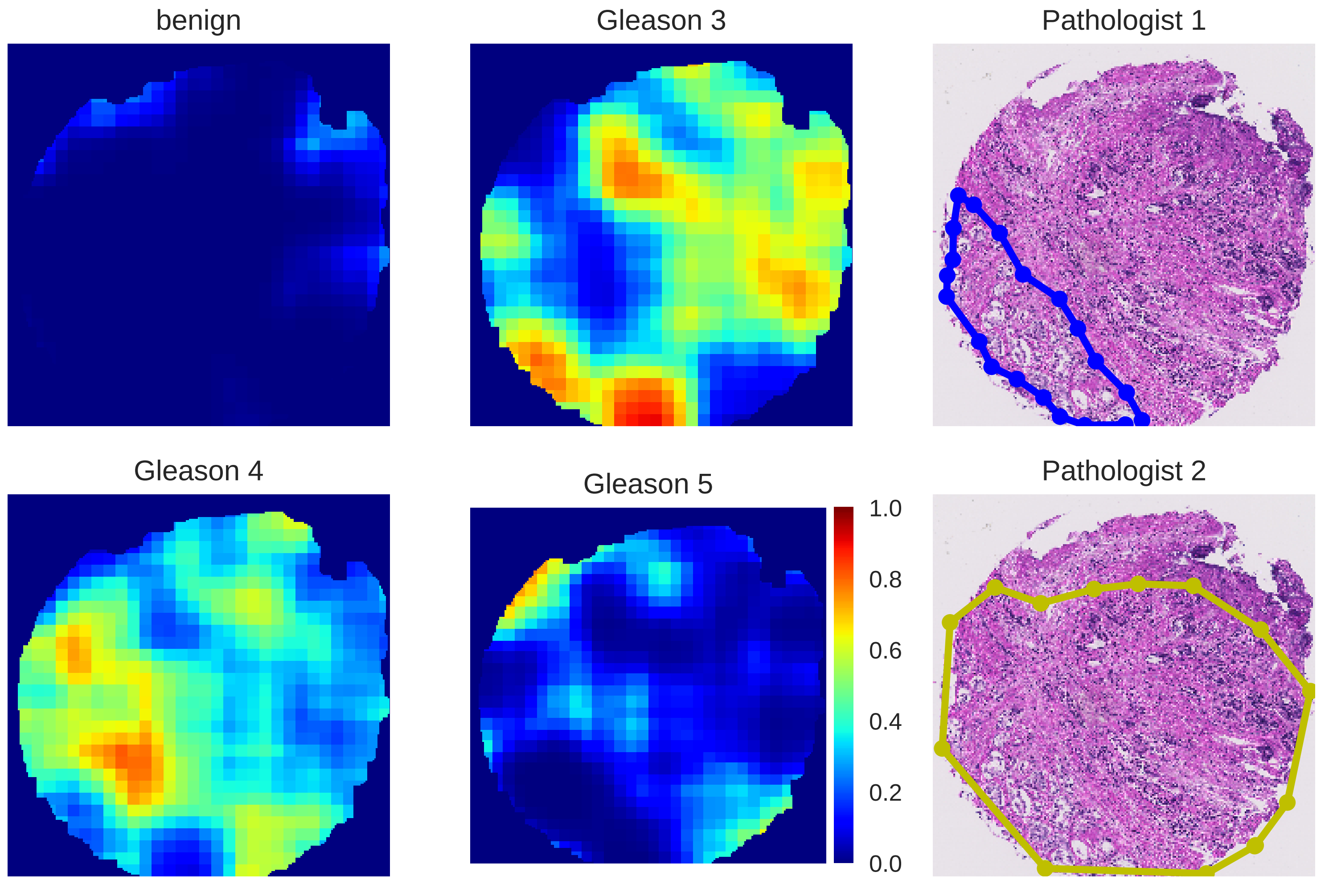

benign
Gleason 3
Pathologist 1
Gleason 4
Gleason 5
Pathologist 2
1.0
0.8
0.6
0.4
0.2
0.0

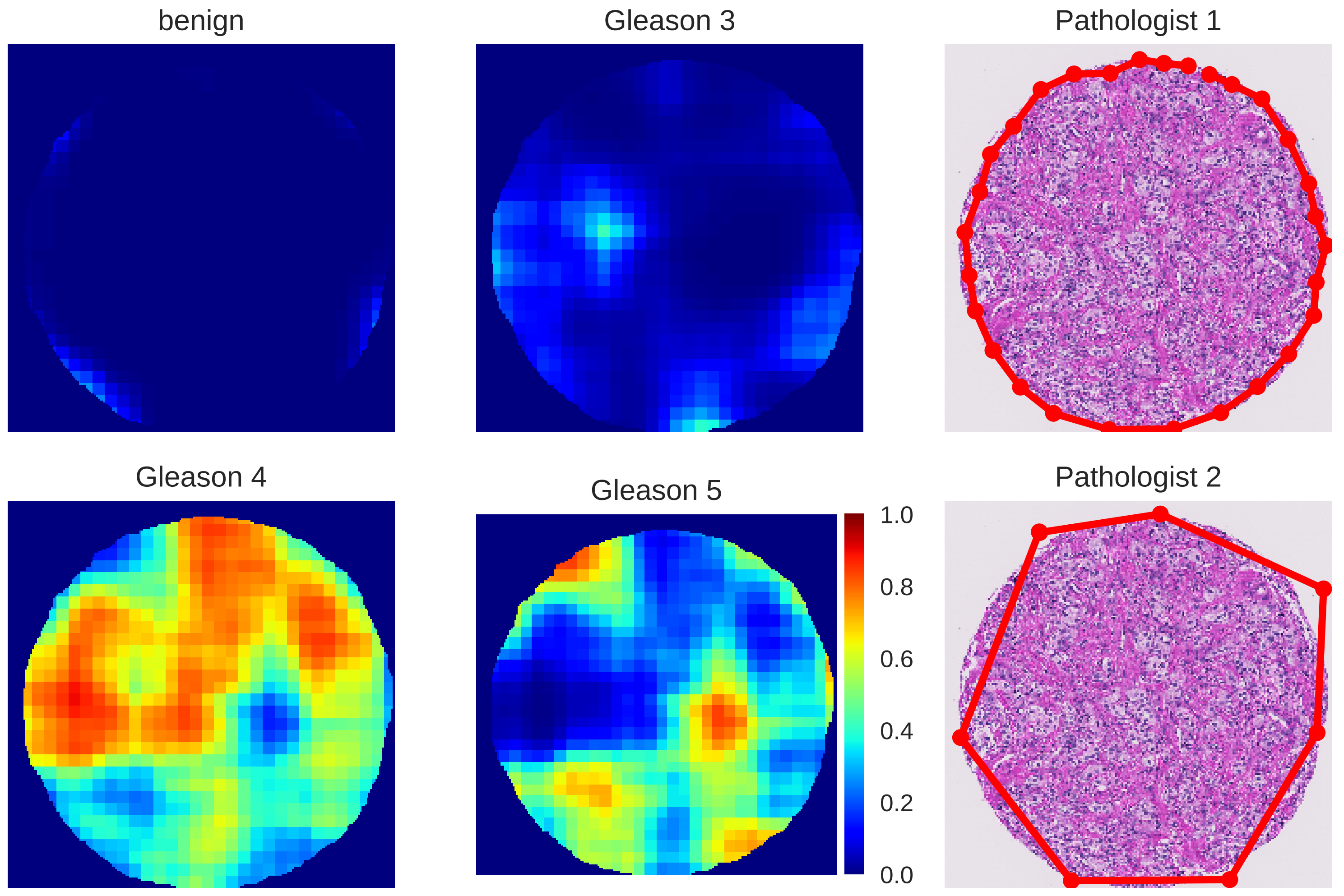

benign
Gleason 3
Pathologist 1
Gleason 4
Gleason 5
Pathologist 2
1.0
0.8
0.6
0.4
0.2
0.0

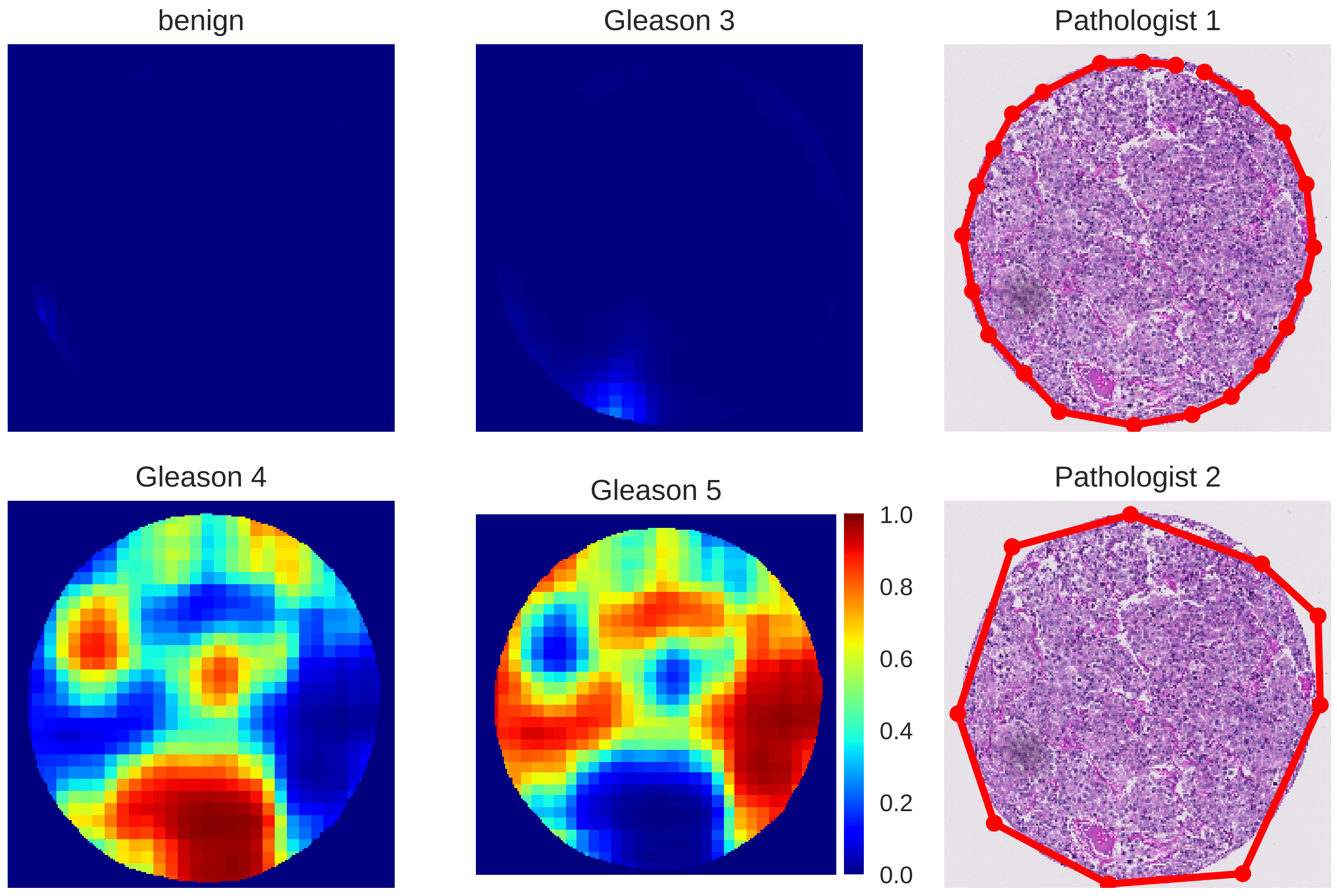

benign
Gleason 3
Pathologist 1
Gleason 4
Gleason 5
Pathologist 2
1.0
0.8
0.6
0.4
0.2
0.0

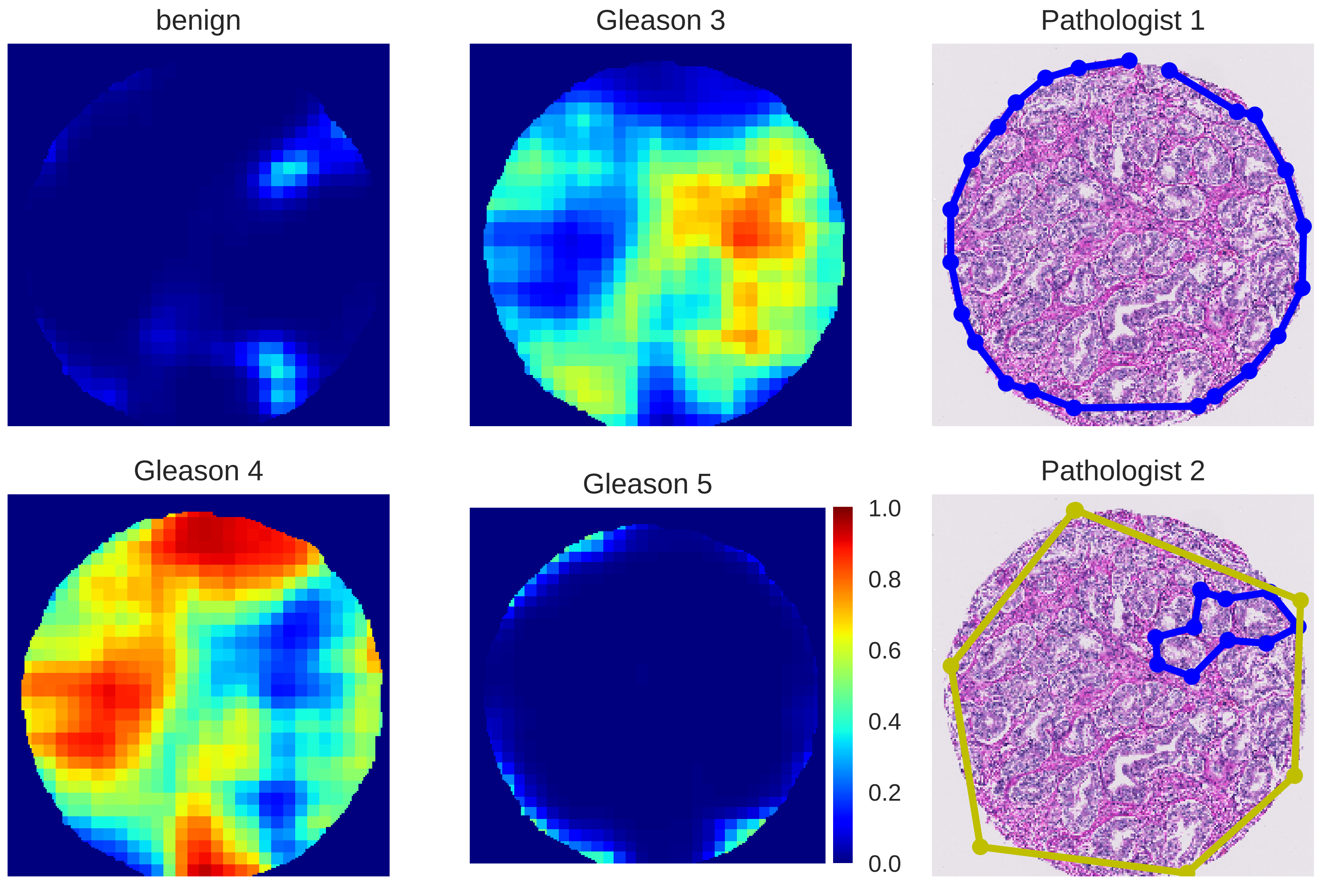

benign
Gleason 3
Pathologist 1
Gleason 4
Gleason 5
Pathologist 2
1.0
0.8
0.6
0.4
0.2
0.0

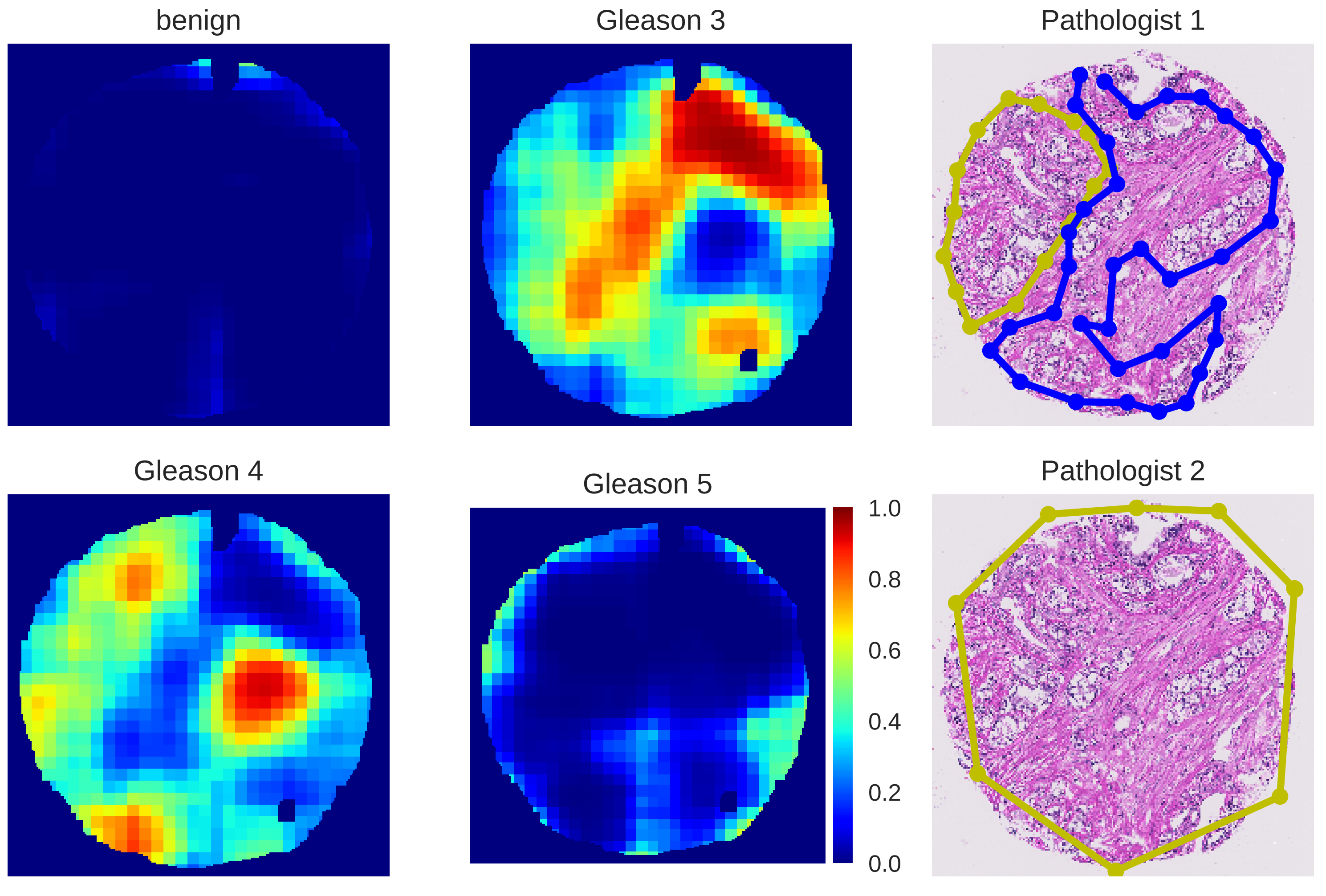

benign
Gleason 3
Pathologist 1
Gleason 4
Gleason 5
Pathologist 2
1.0
0.8
0.6
0.4
0.2
0.0

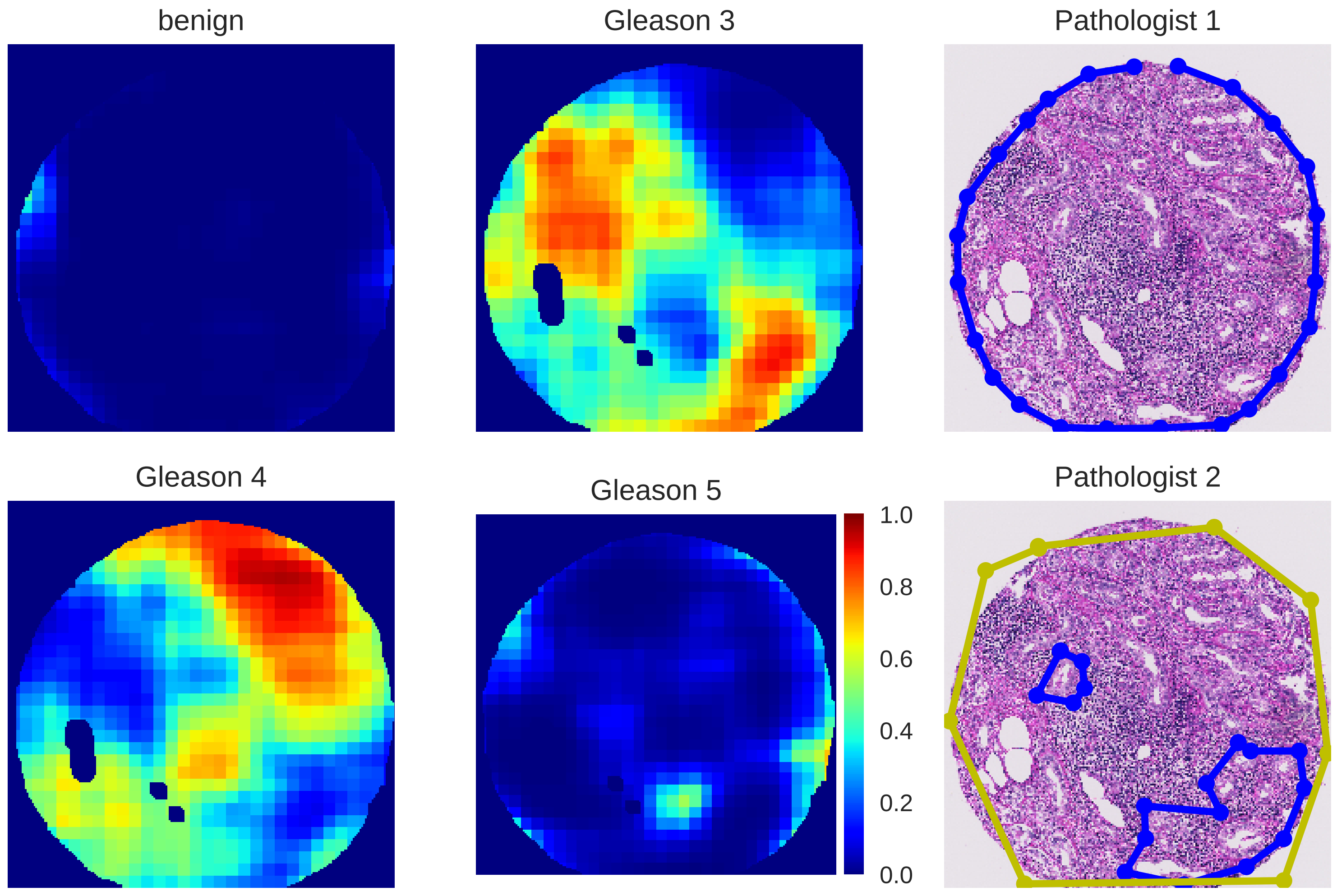

benign
Gleason 3
Pathologist 1
Gleason 4
Gleason 5
Pathologist 2
1.0
0.8
0.6
0.4
0.2
0.0

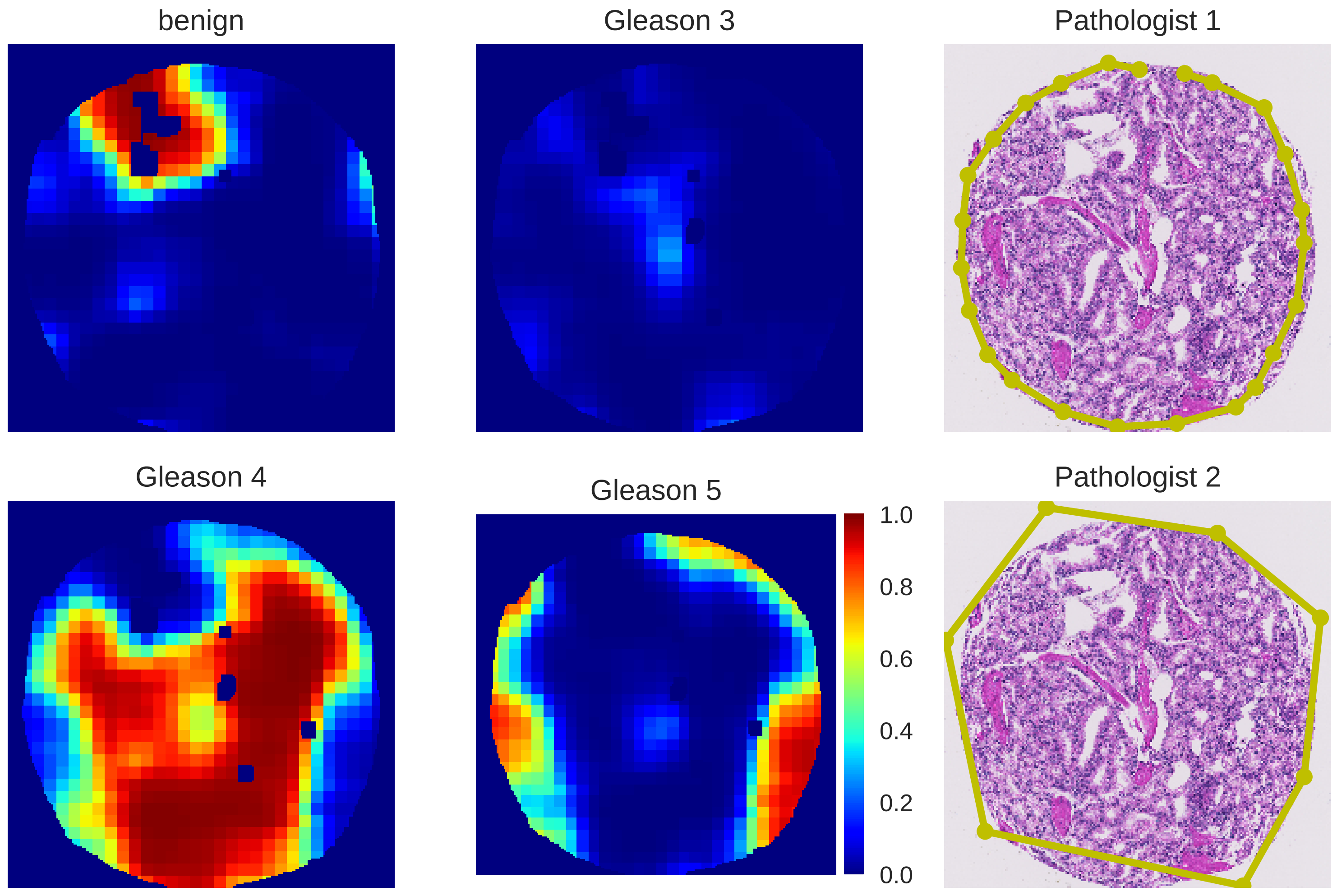
benign
Gleason 3
Pathologist 1
Gleason 4
Gleason 5
Pathologist 2
1.0
0.8
0.6
0.4
0.2
0.0

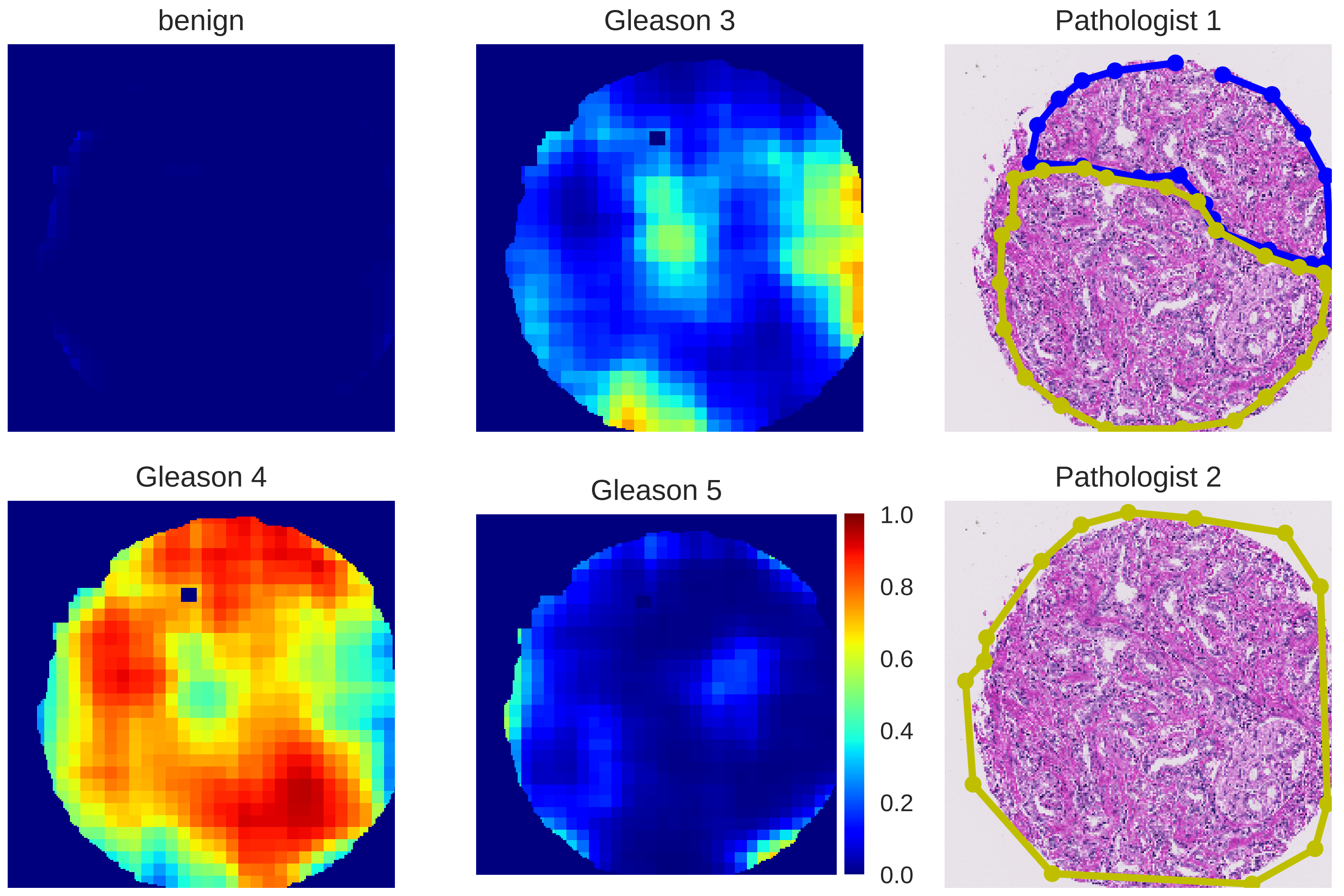

benign
Gleason 3
Pathologist 1
Gleason 4
Gleason 5
Pathologist 2
1.0
0.8
0.6
0.4
0.2
0.0

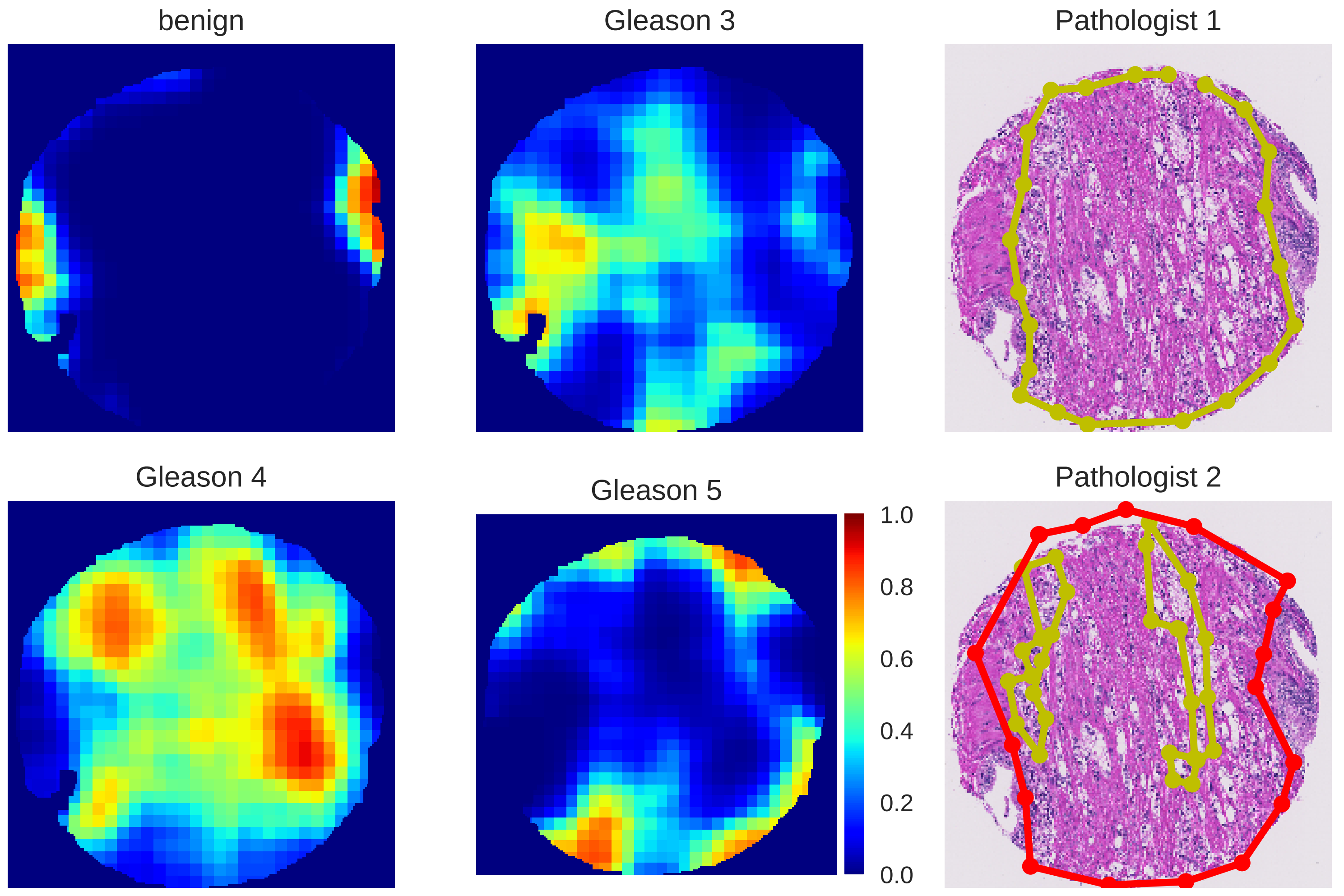
benign
Gleason 3
Pathologist 1
Gleason 4
Gleason 5
Pathologist 2
1.0
0.8
0.6
0.4
0.2
0.0

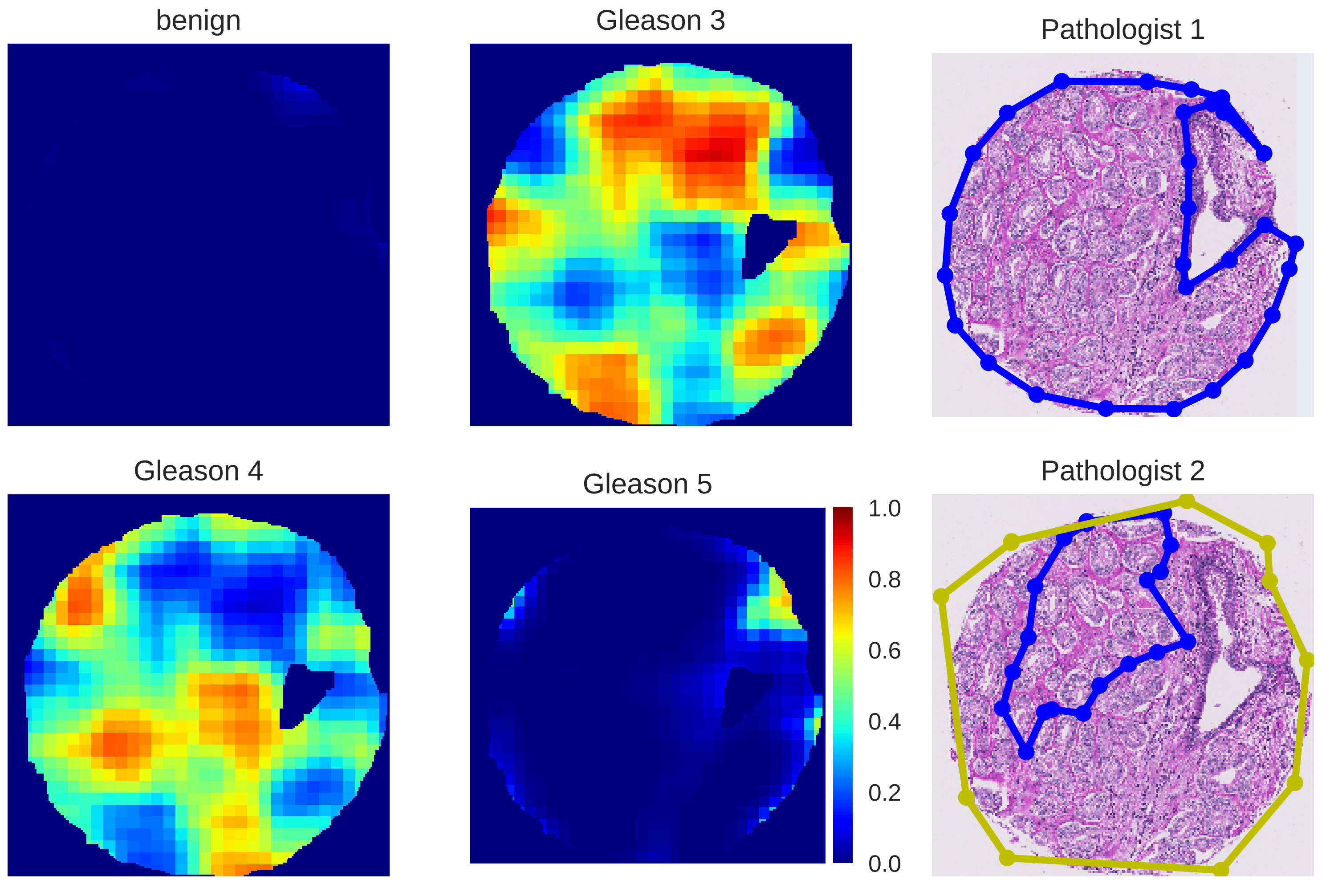

benign
Gleason 3
Pathologist 1
Gleason 4
Gleason 5
Pathologist 2
1.0
0.8
0.6
0.4
0.2
0.0

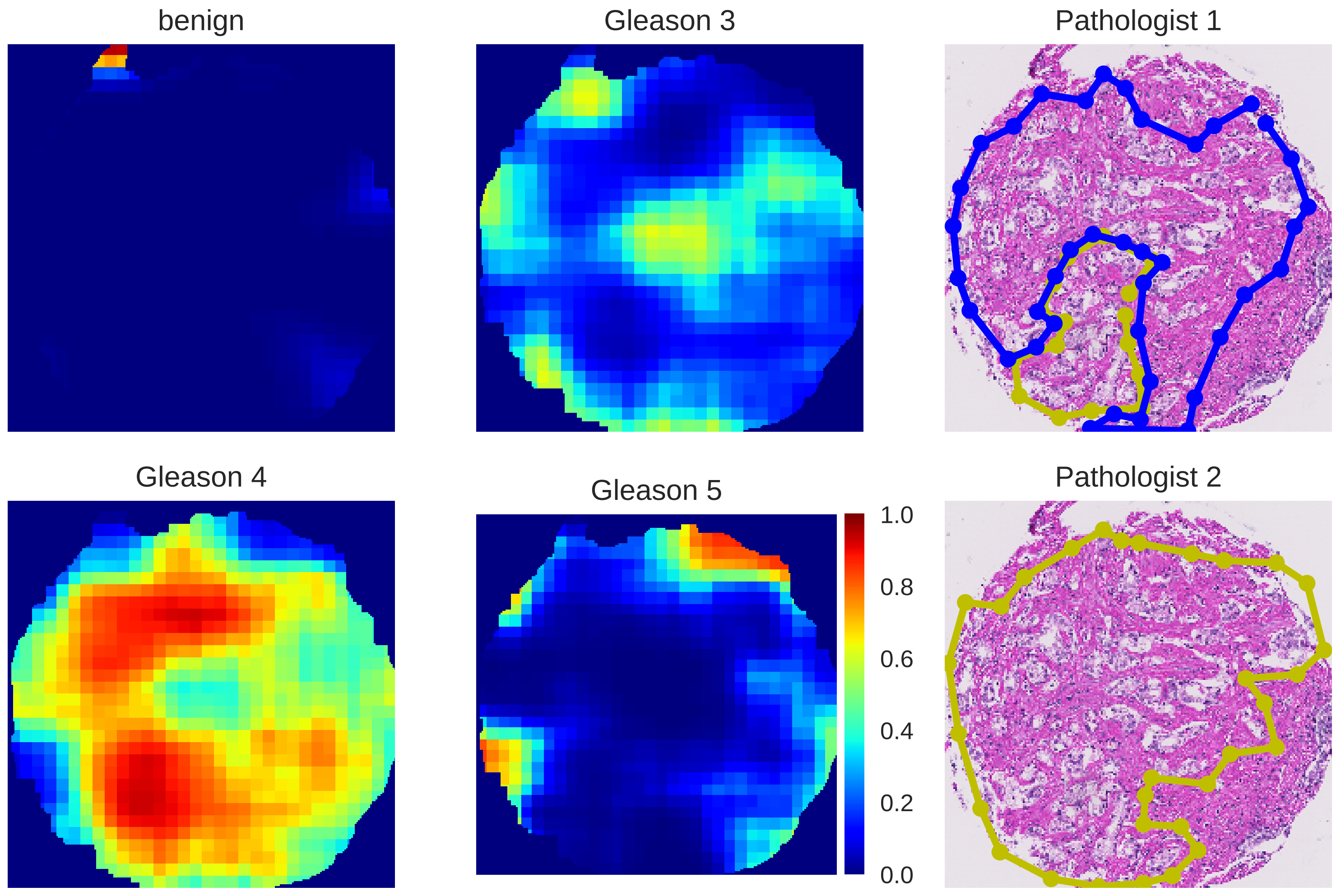

benign
Gleason 3
Pathologist 1
Gleason 4
Gleason 5
Pathologist 2
1.0
0.8
0.6
0.4
0.2
0.0

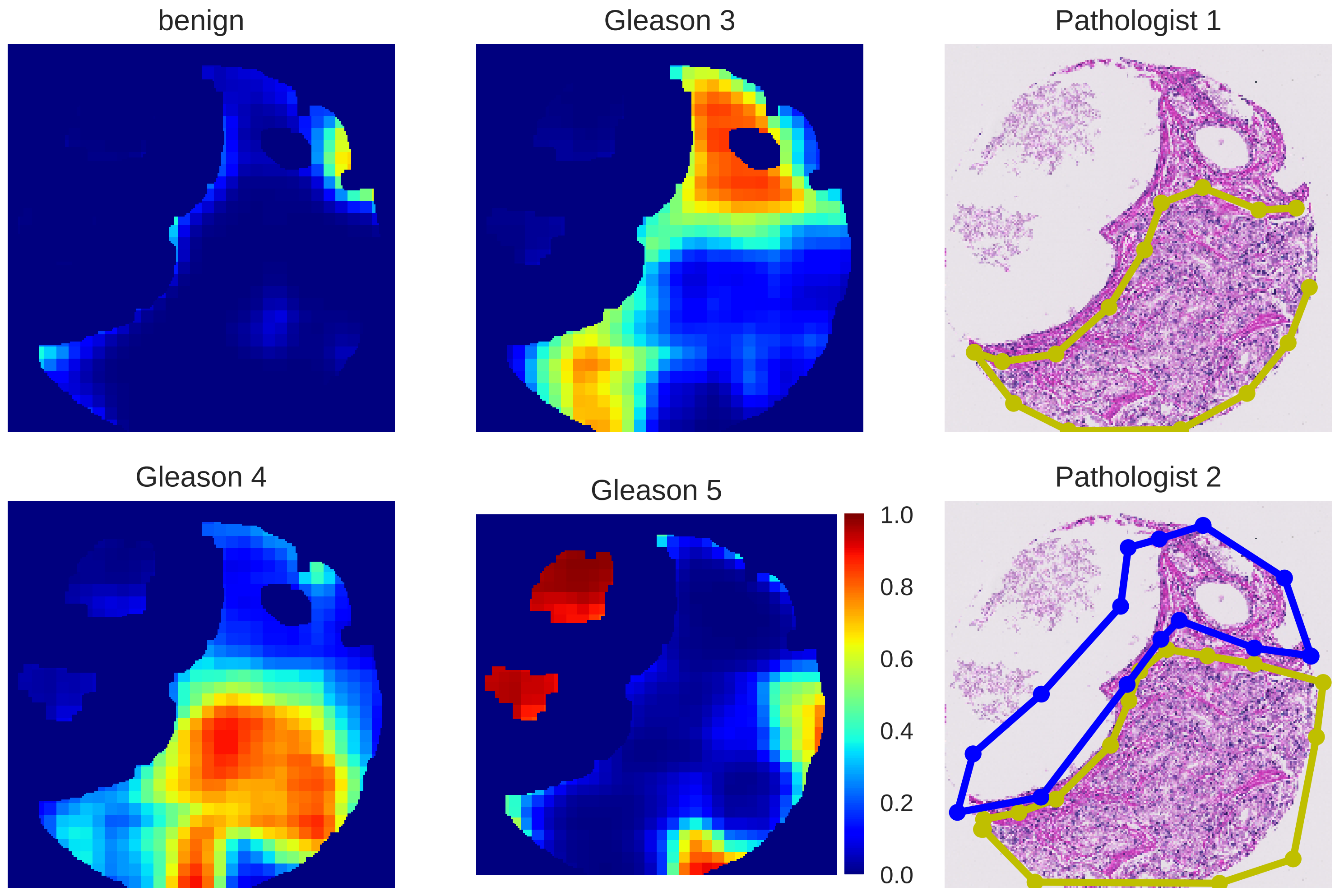
benign
Gleason 3
Pathologist 1
Gleason 4
Gleason 5
Pathologist 2
1.0
0.8
0.6
0.4
0.2
0.0

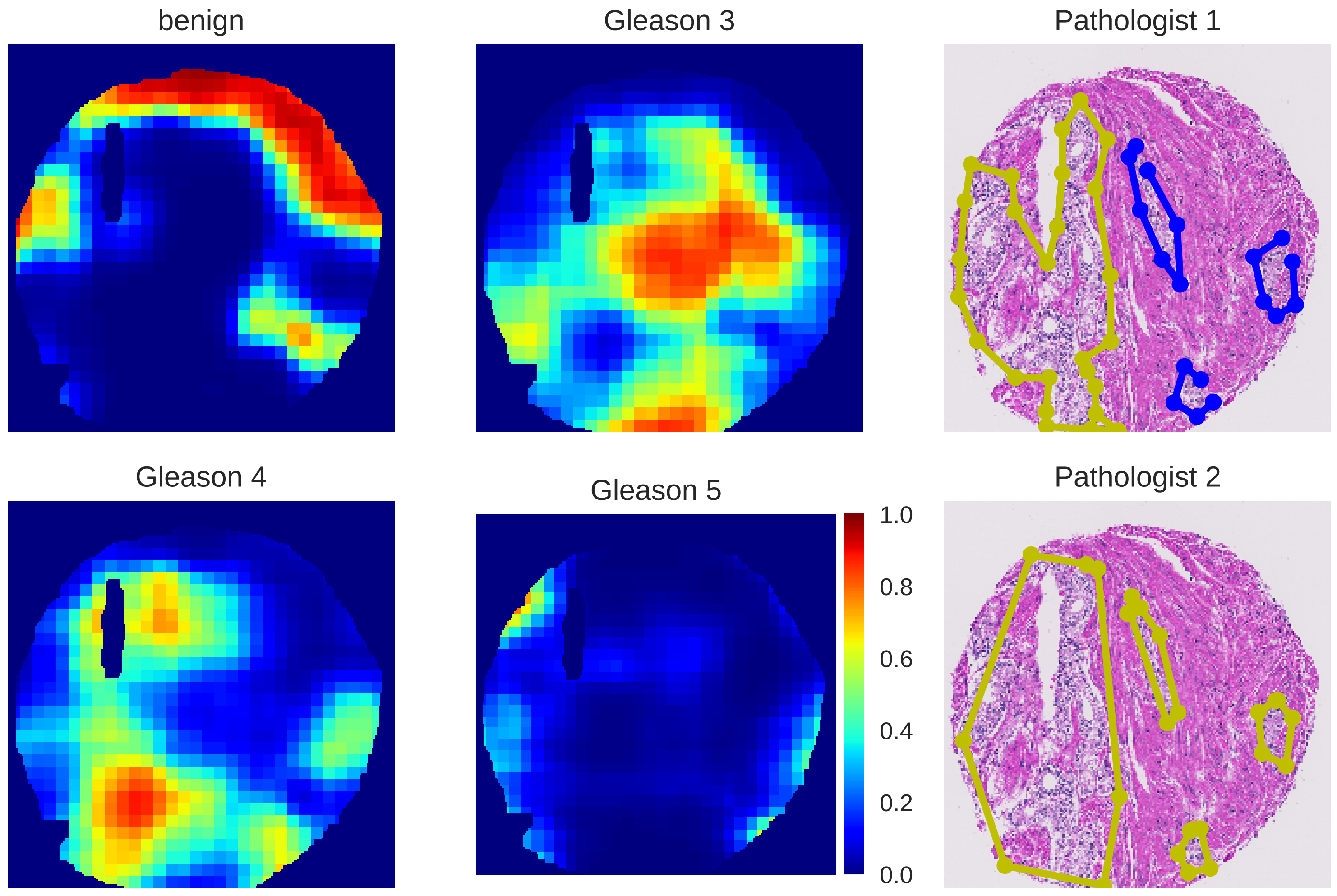

benign
Gleason 3
Pathologist 1
Gleason 4
Gleason 5
Pathologist 2
1.0
0.8
0.6
0.4
0.2
0.0

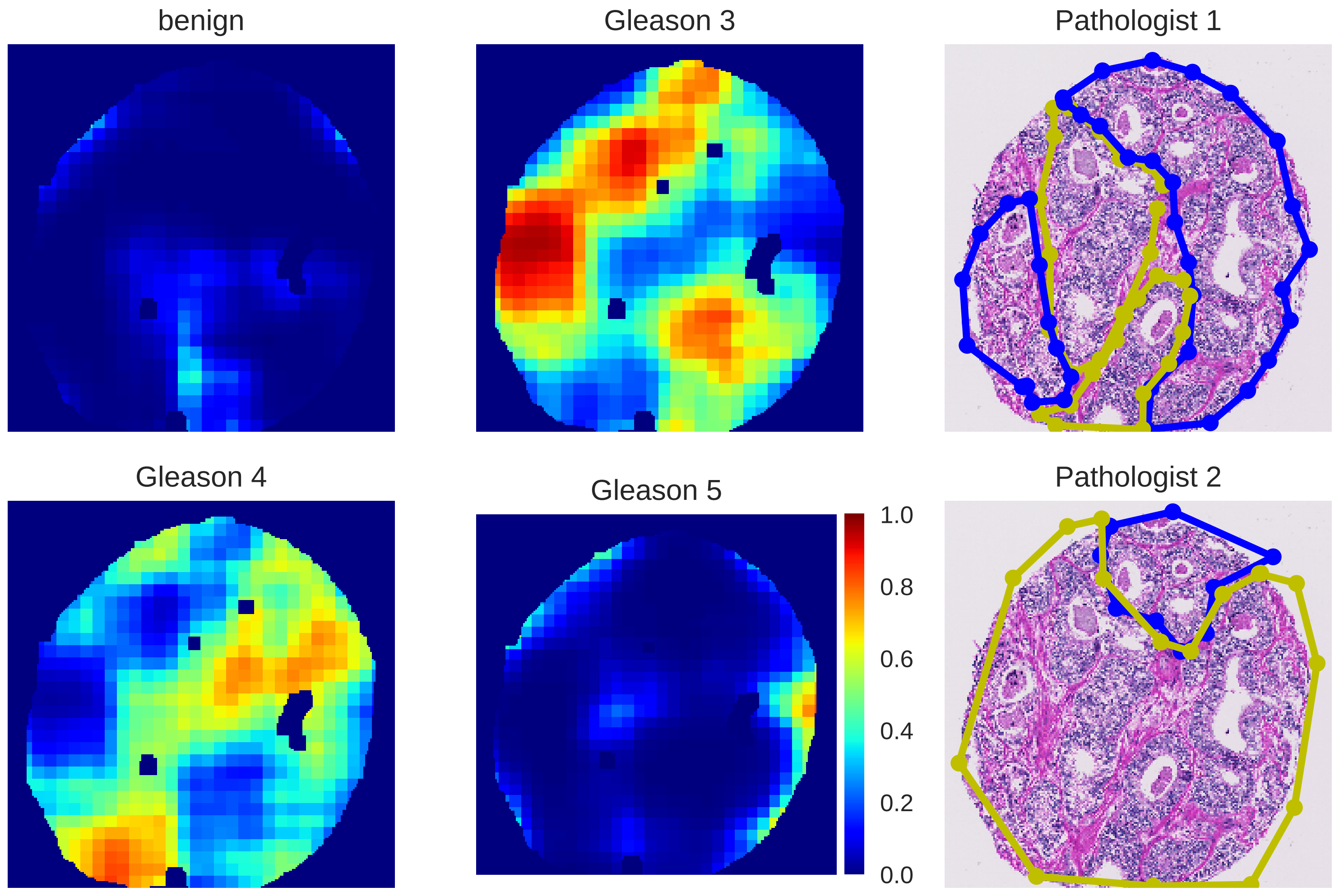

benign
Gleason 3
Pathologist 1
Gleason 4
Gleason 5
Pathologist 2
1.0
0.8
0.6
0.4
0.2
0.0

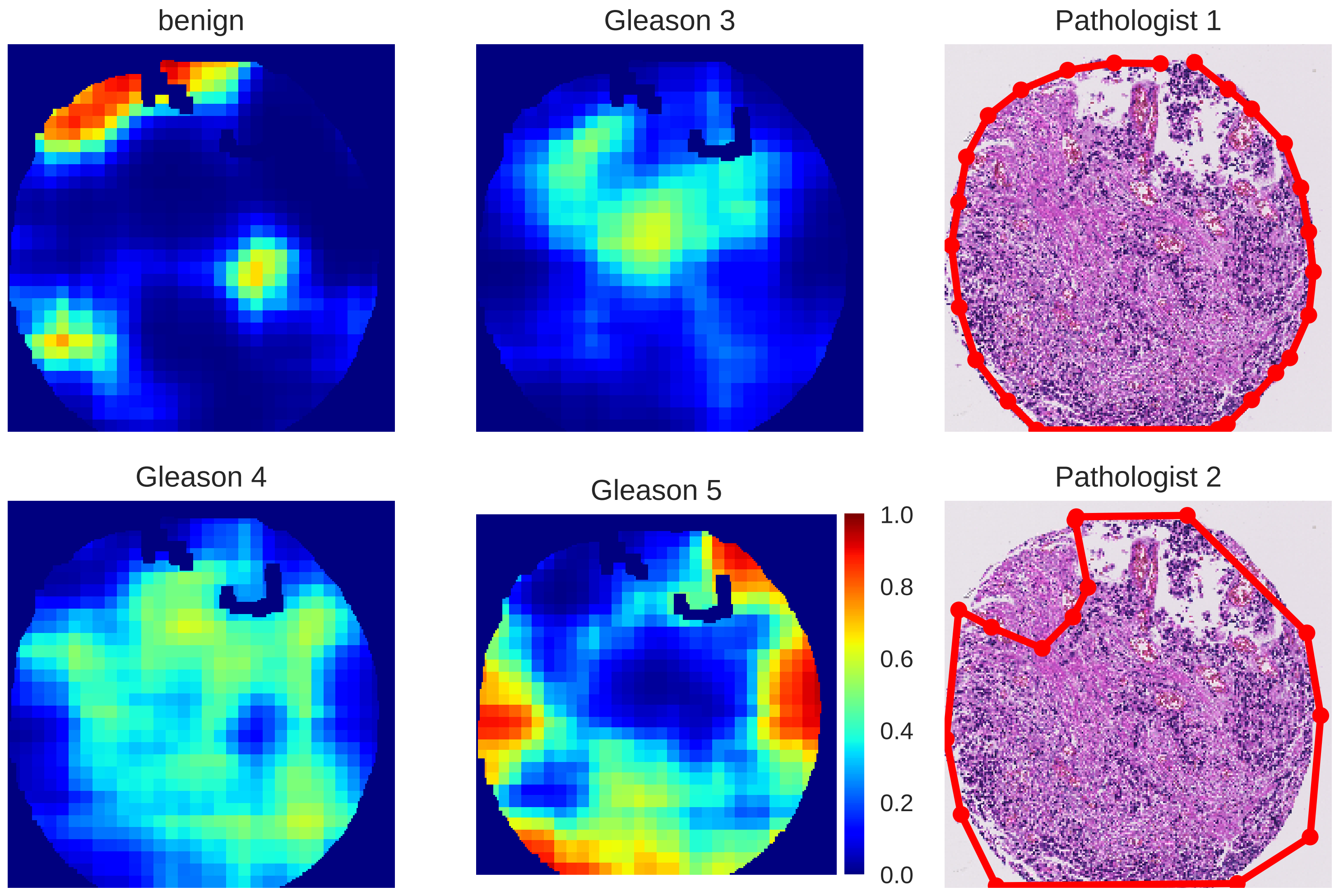

benign
Gleason 3
Pathologist 1
Gleason 4
Gleason 5
Pathologist 2
1.0
0.8
0.6
0.4
0.2
0.0

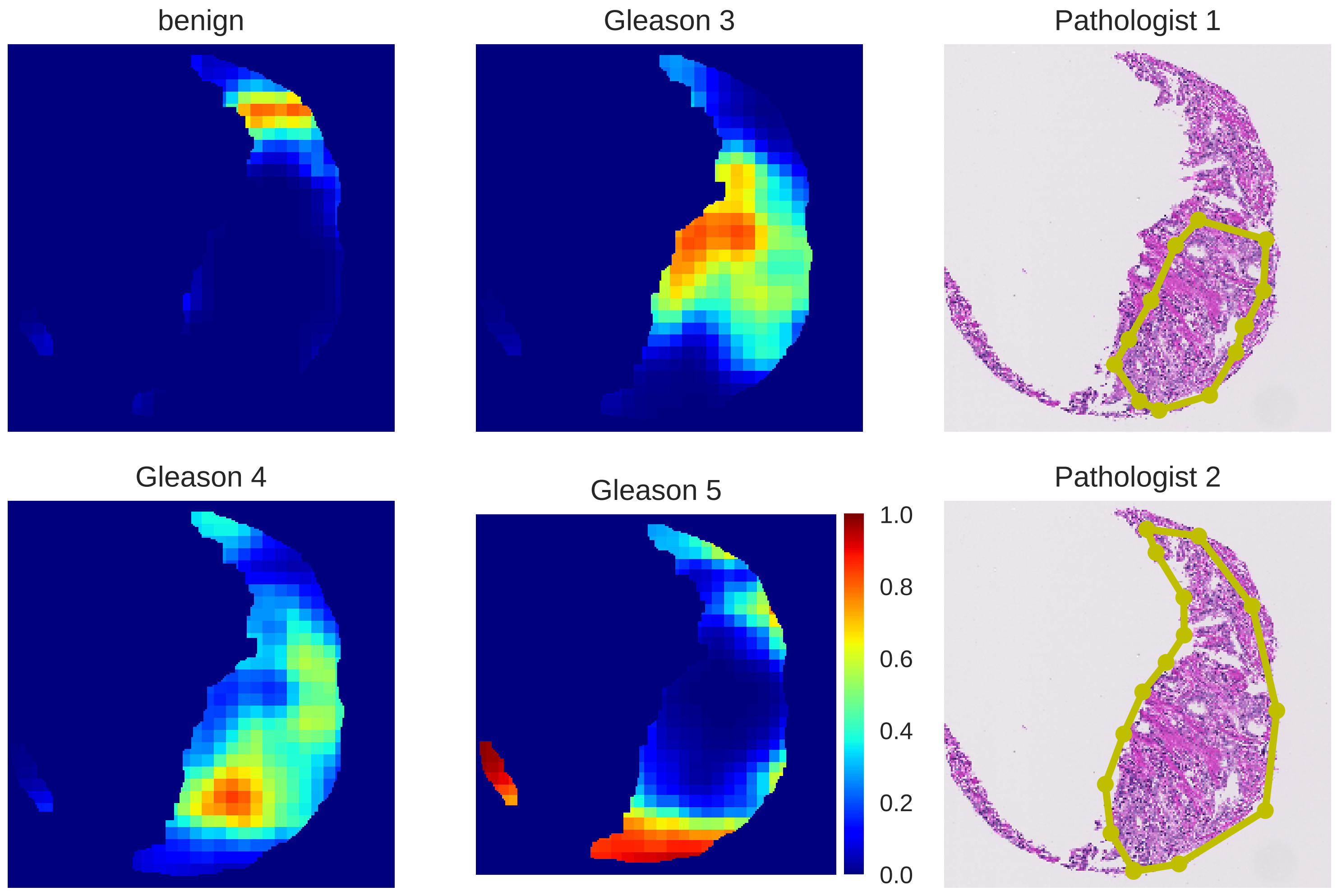
benign
Gleason 3
Pathologist 1
Gleason 4
Gleason 5
Pathologist 2
1.0
0.8
0.6
0.4
0.2
0.0

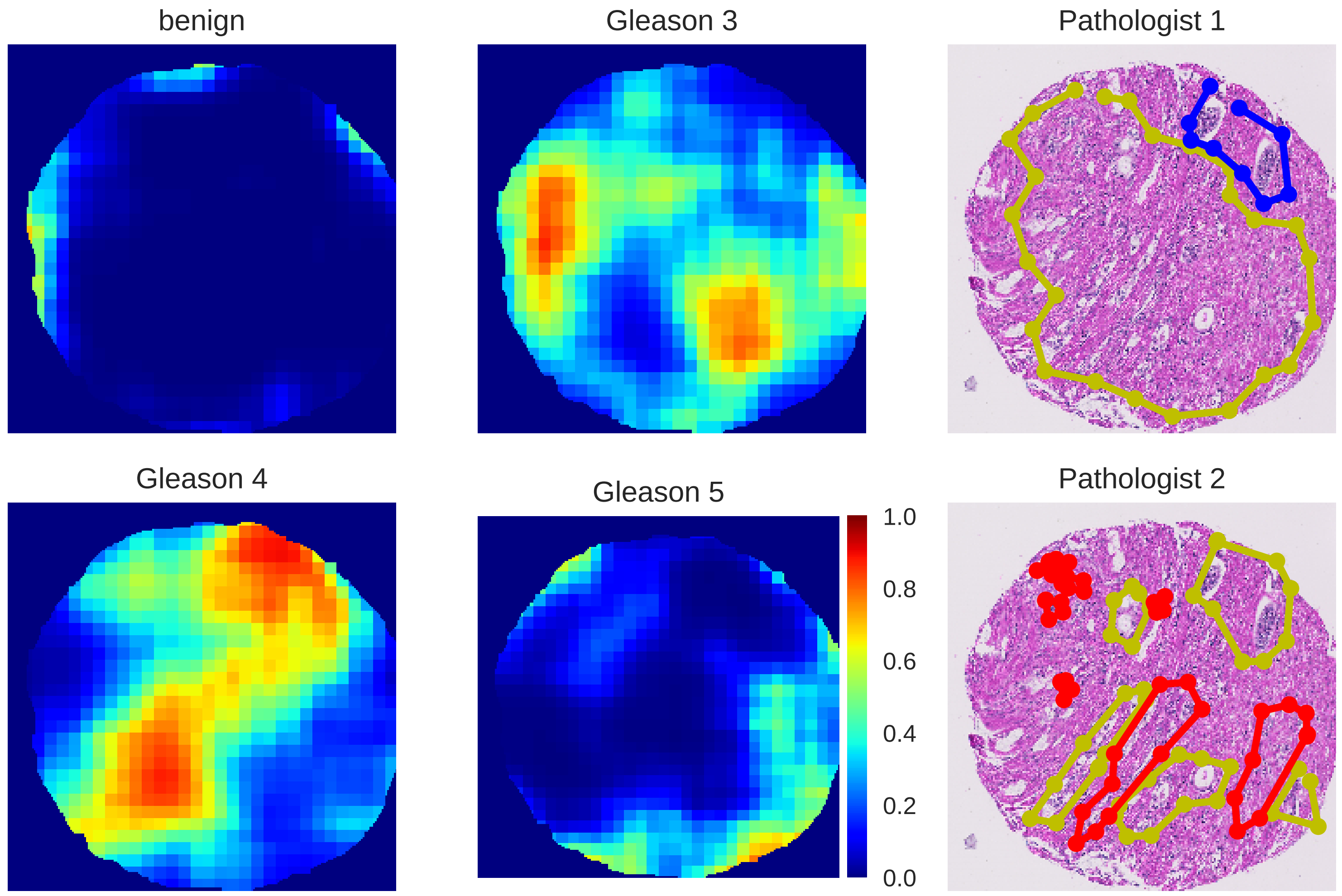
benign
Gleason 3
Pathologist 1
Gleason 4
Gleason 5
Pathologist 2
1.0
0.8
0.6
0.4
0.2
0.0

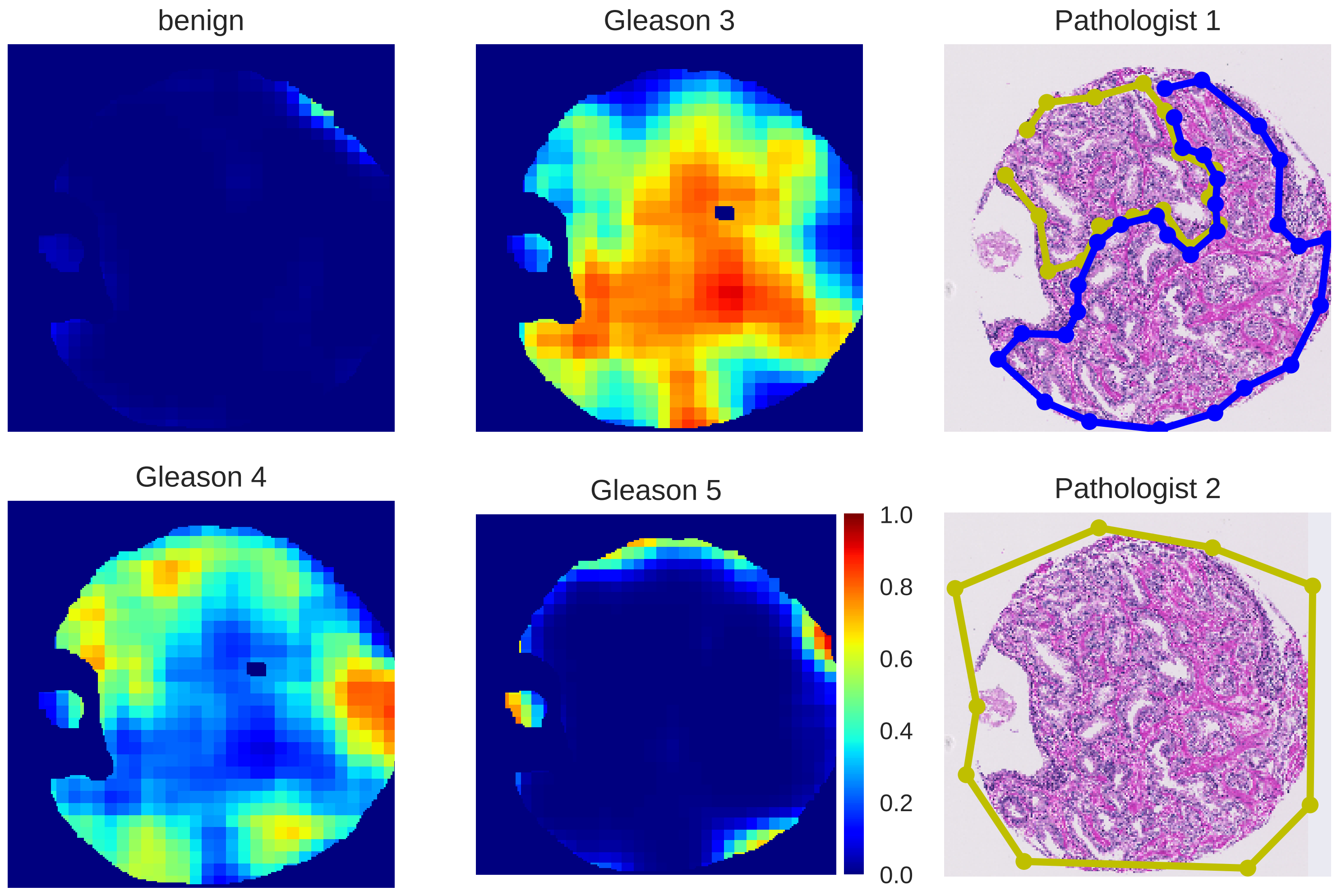

benign
Gleason 3
Pathologist 1
Gleason 4
Gleason 5
Pathologist 2
1.0
0.8
0.6
0.4
0.2
0.0

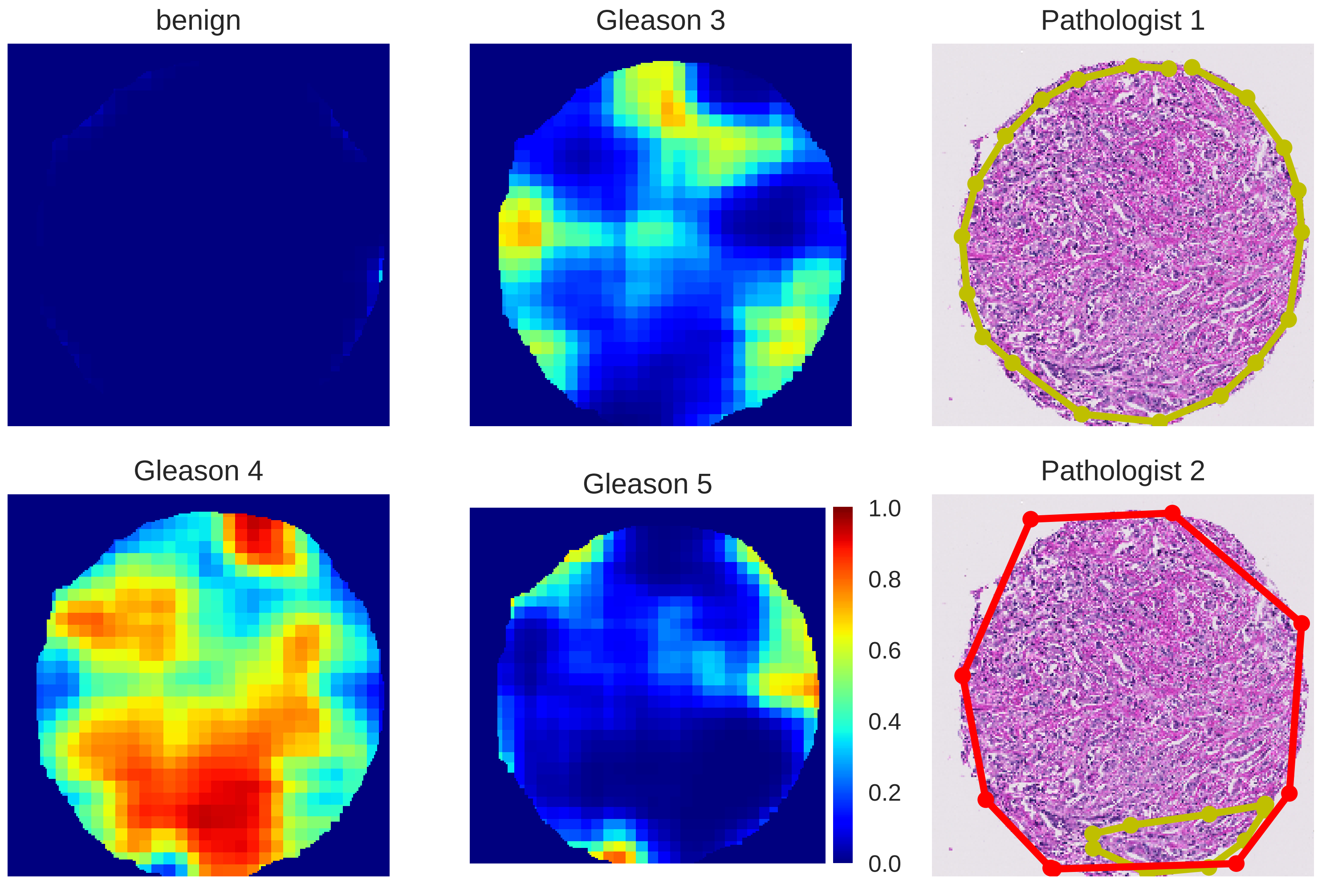
benign
Gleason 3
Pathologist 1
Gleason 4
Gleason 5
Pathologist 2
1.0
0.8
0.6
0.4
0.2
0.0

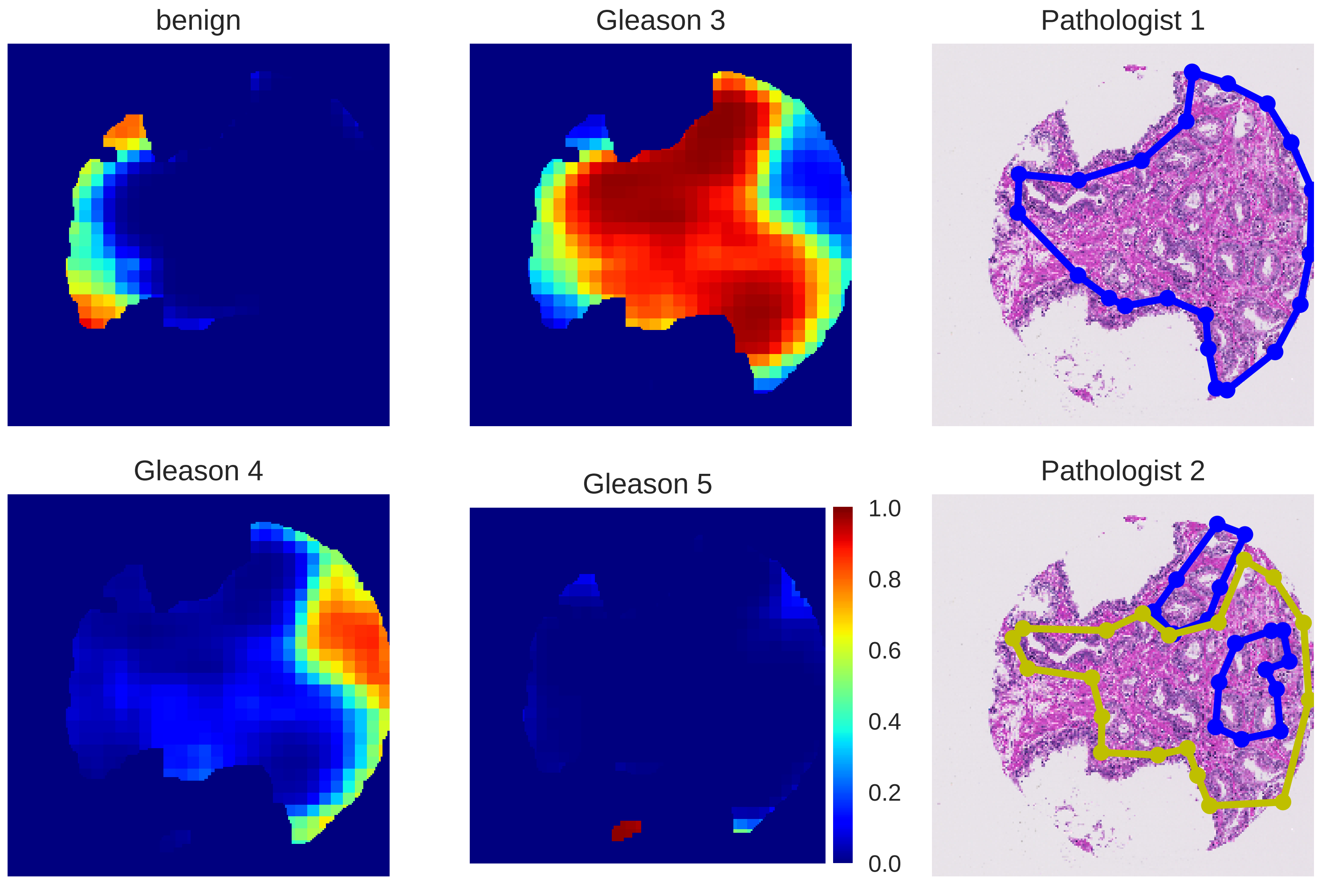
benign
Gleason 3
Pathologist 1
Gleason 4
Gleason 5
Pathologist 2
1.0
0.8
0.6
0.4
0.2
0.0

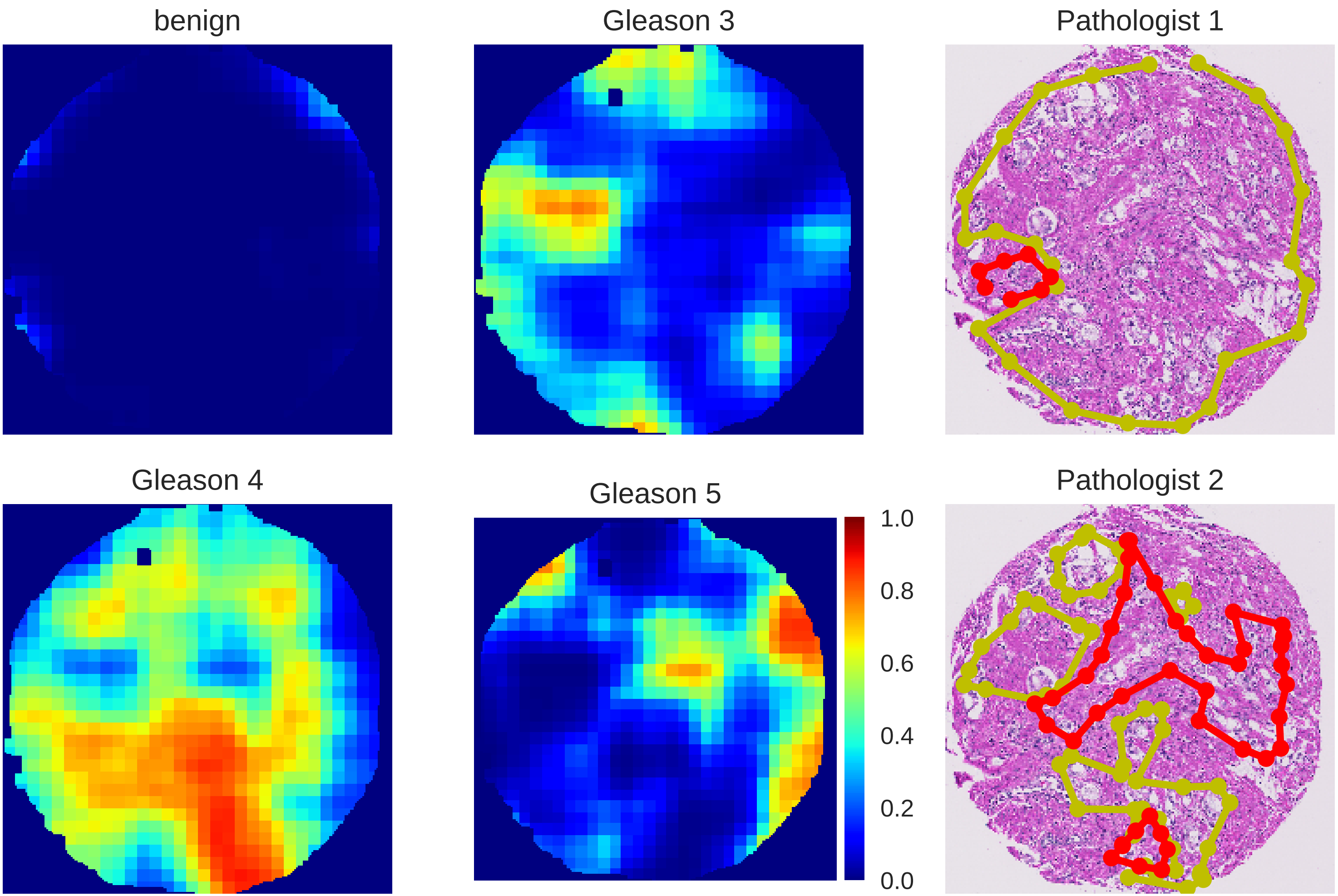

benign
Gleason 3
Pathologist 1
Gleason 4
Gleason 5
Pathologist 2
1.0
0.8
0.6
0.4
0.2
0.0

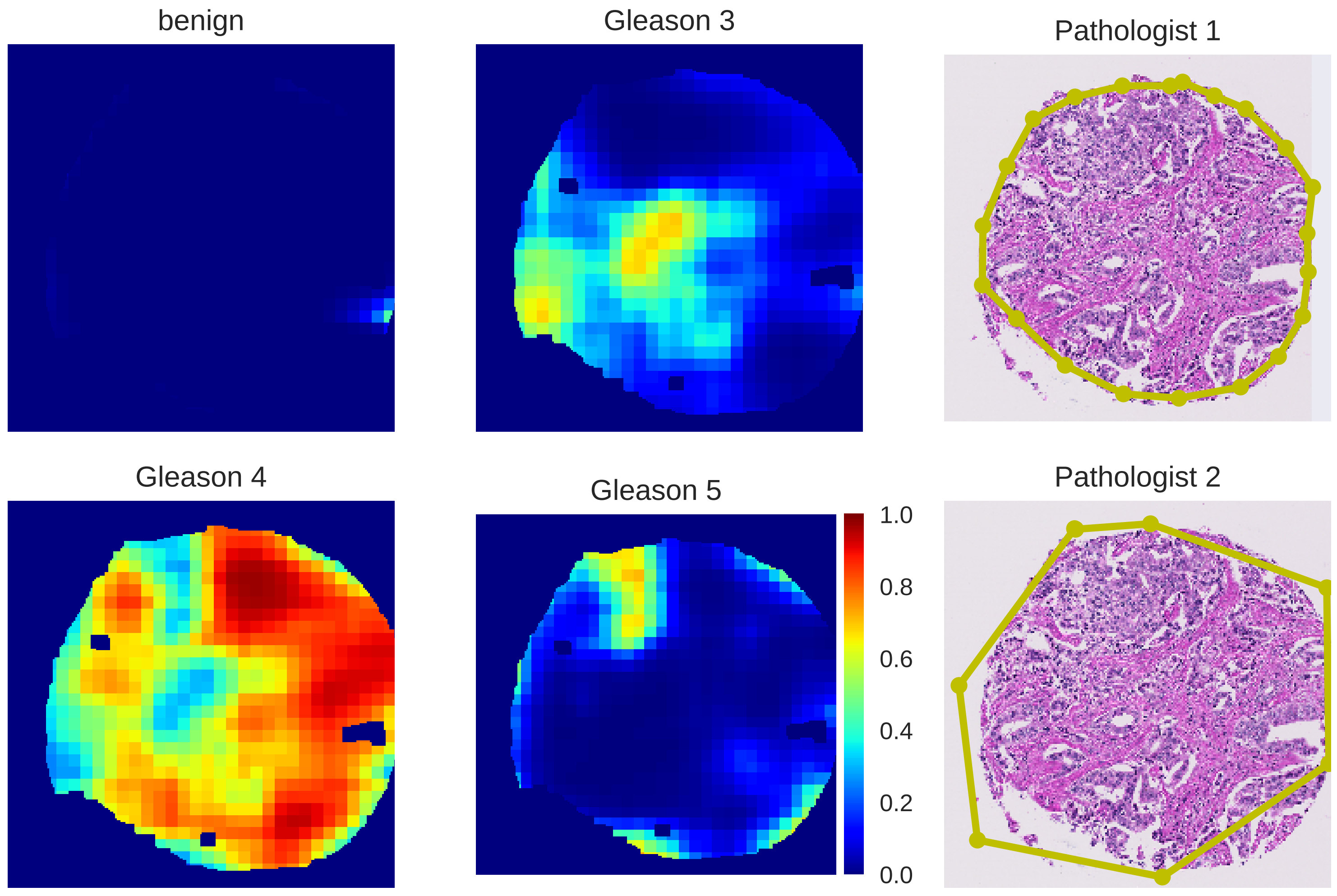
benign
Gleason 3
Pathologist 1
Gleason 4
Gleason 5
Pathologist 2
1.0
0.8
0.6
0.4
0.2
0.0

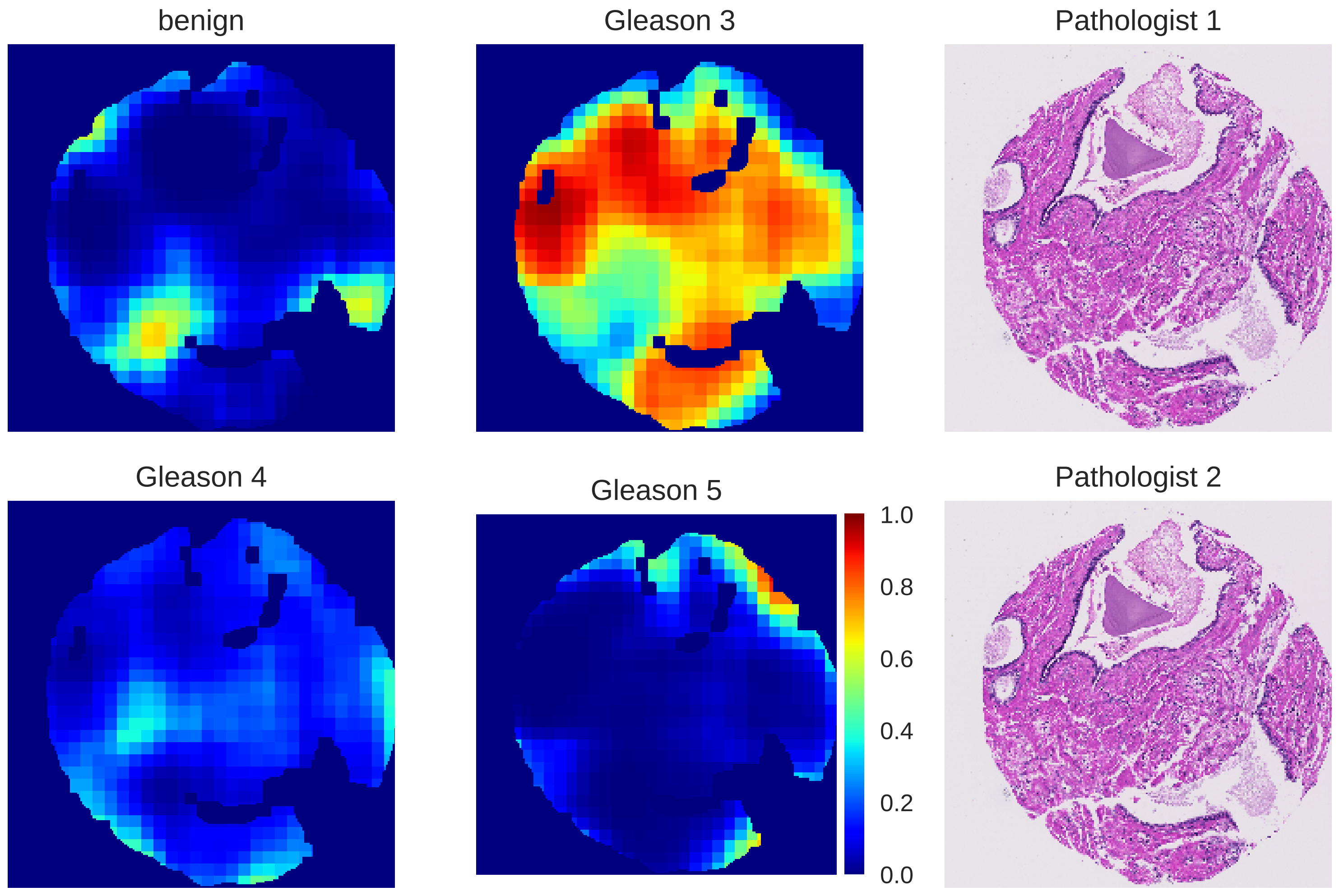
benign
Gleason 3
Pathologist 1
Gleason 4
Gleason 5
Pathologist 2
1.0
0.8
0.6
0.4
0.2
0.0

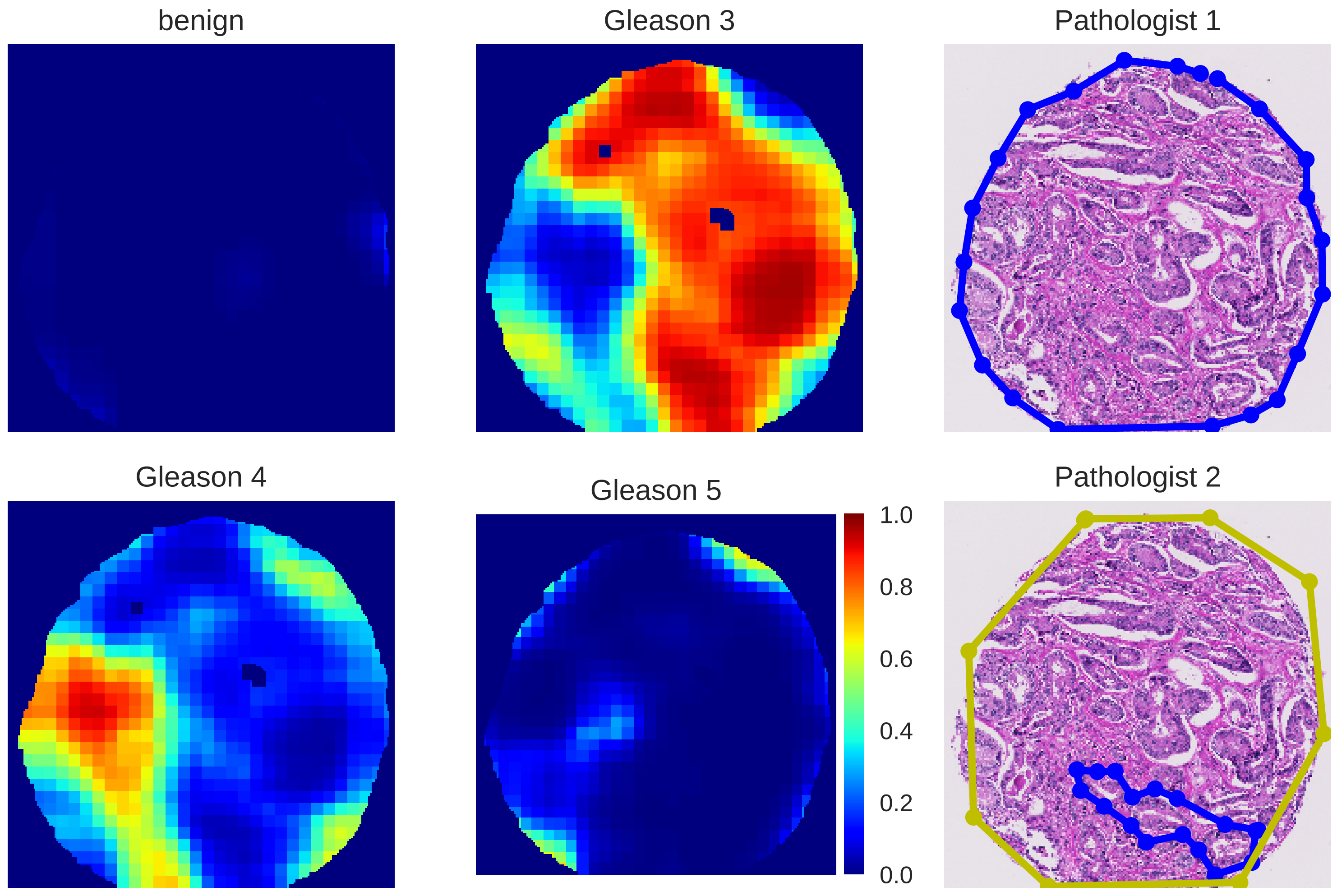

benign
Gleason 3
Pathologist 1
Gleason 4
Gleason 5
Pathologist 2
1.0
0.8
0.6
0.4
0.2
0.0

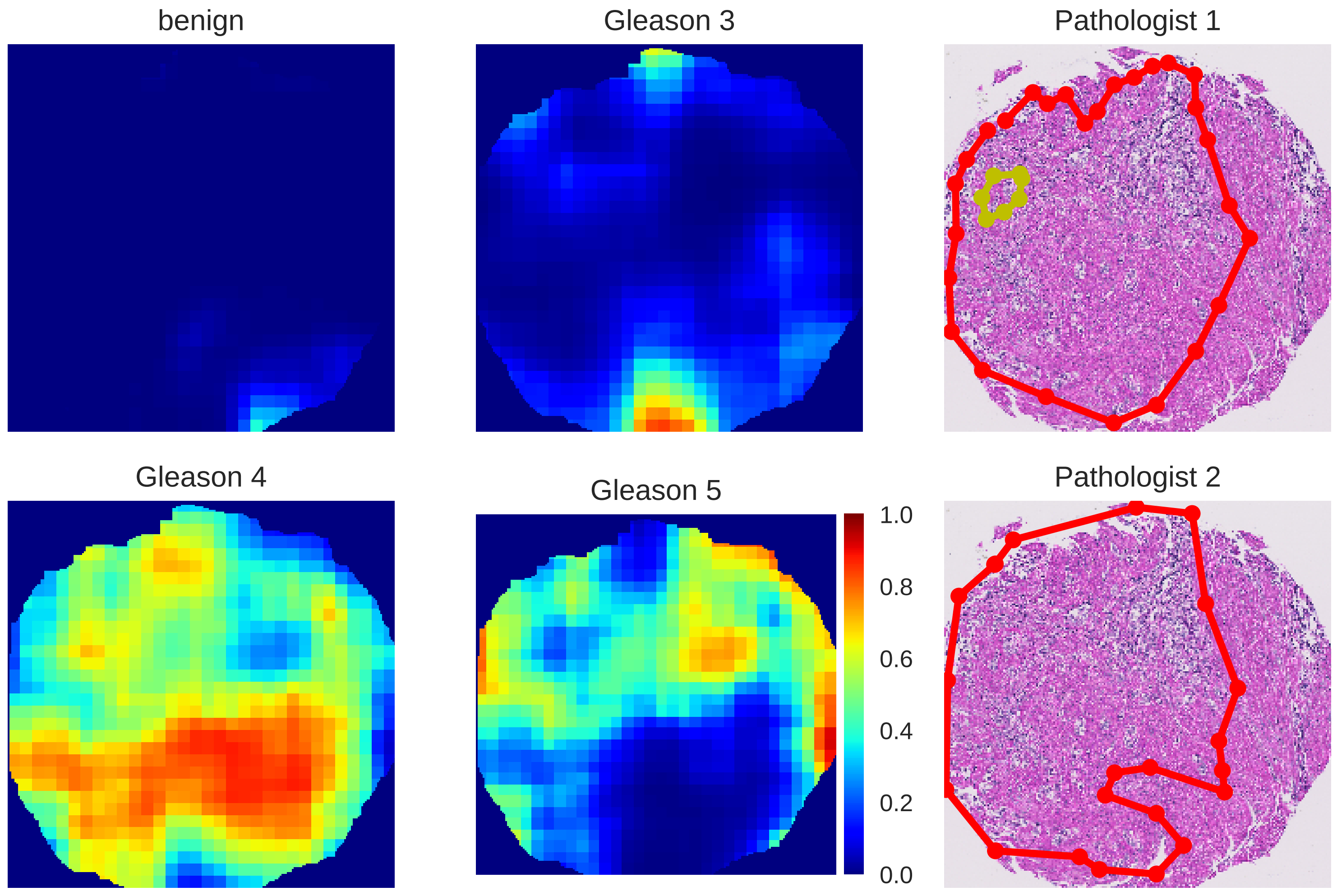
benign
Gleason 3
Pathologist 1
Gleason 4
Gleason 5
Pathologist 2
1.0
0.8
0.6
0.4
0.2
0.0

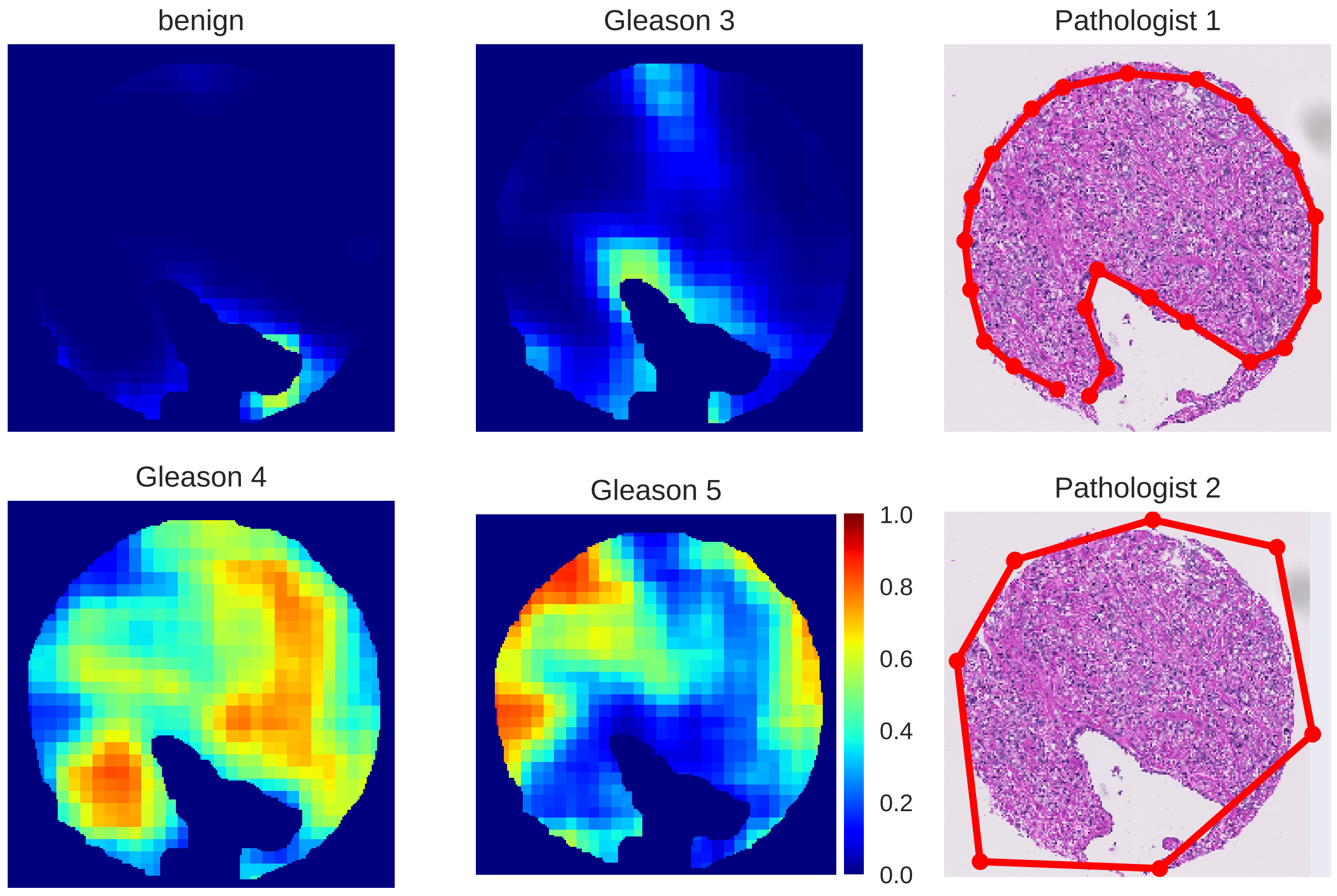
benign
Gleason 3
Pathologist 1
Gleason 4
Gleason 5
Pathologist 2
1.0
0.8
0.6
0.4
0.2
0.0

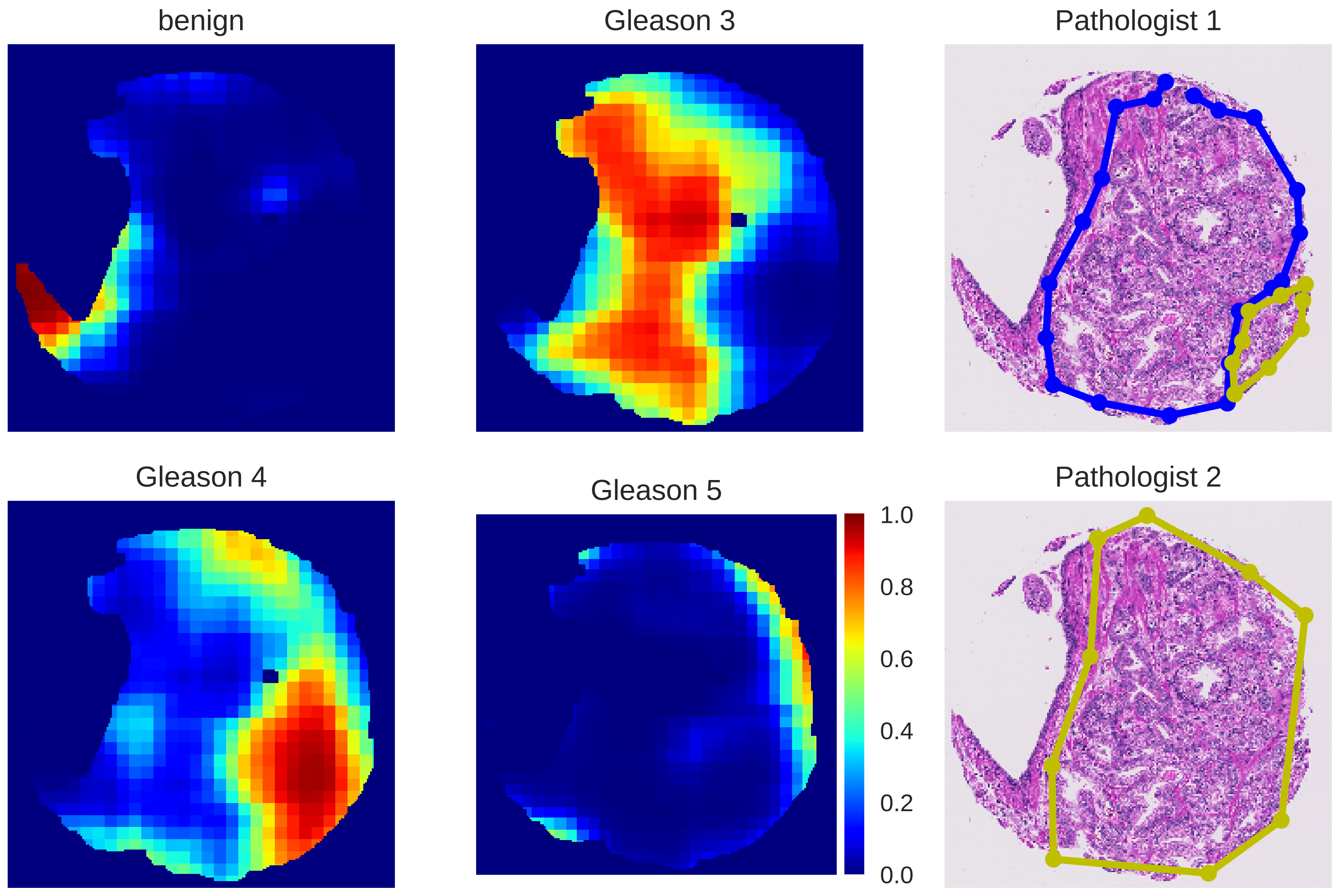

benign
Gleason 3
Pathologist 1
Gleason 4
Gleason 5
Pathologist 2
1.0
0.8
0.6
0.4
0.2
0.0

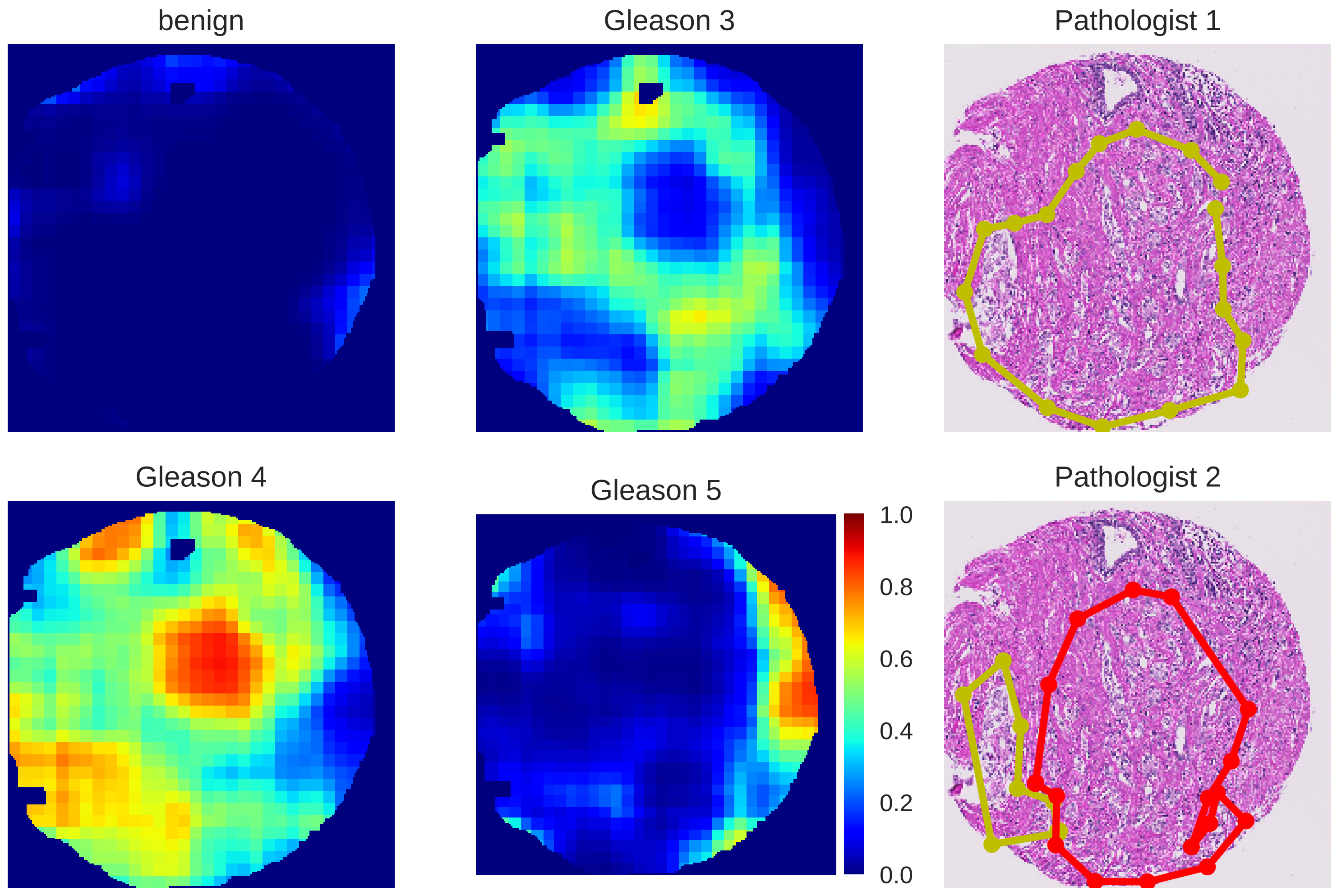

benign
Gleason 3
Pathologist 1
Gleason 4
Gleason 5
Pathologist 2
1.0
0.8
0.6
0.4
0.2
0.0

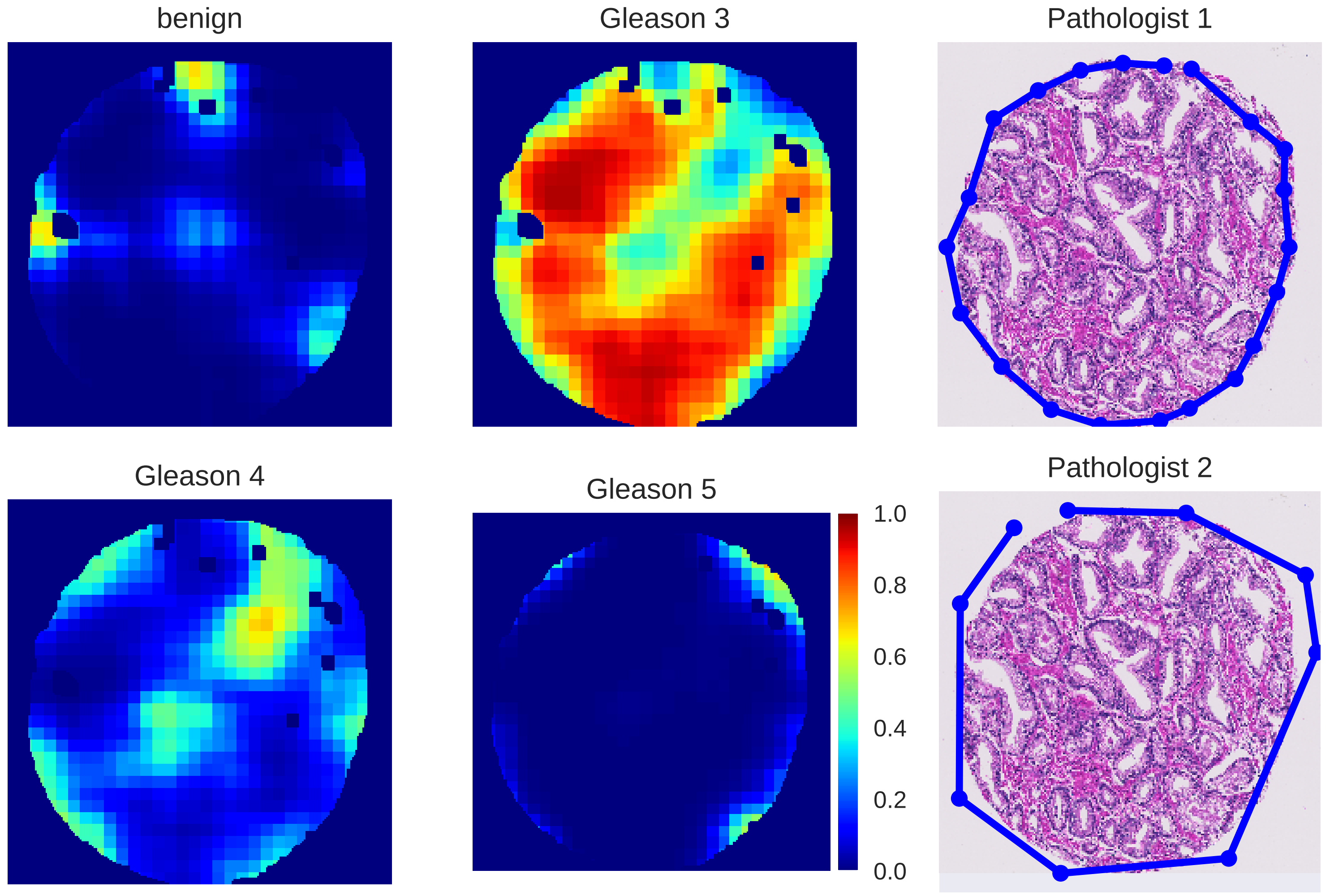

benign
Gleason 3
Pathologist 1
Gleason 4
Gleason 5
Pathologist 2
1.0
0.8
0.6
0.4
0.2
0.0

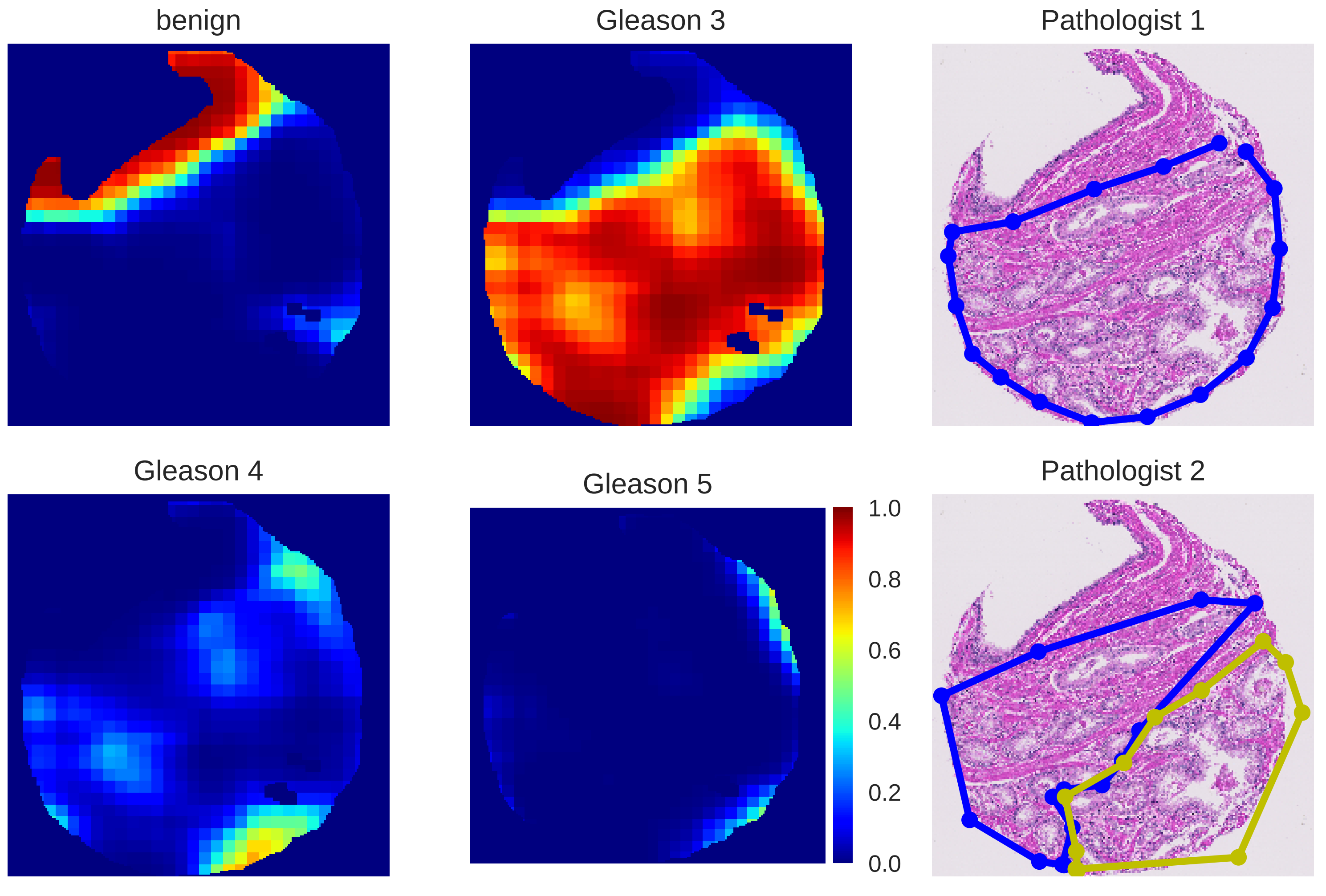
benign
Gleason 3
Pathologist 1
Gleason 4
Gleason 5
Pathologist 2
1.0
0.8
0.6
0.4
0.2
0.0

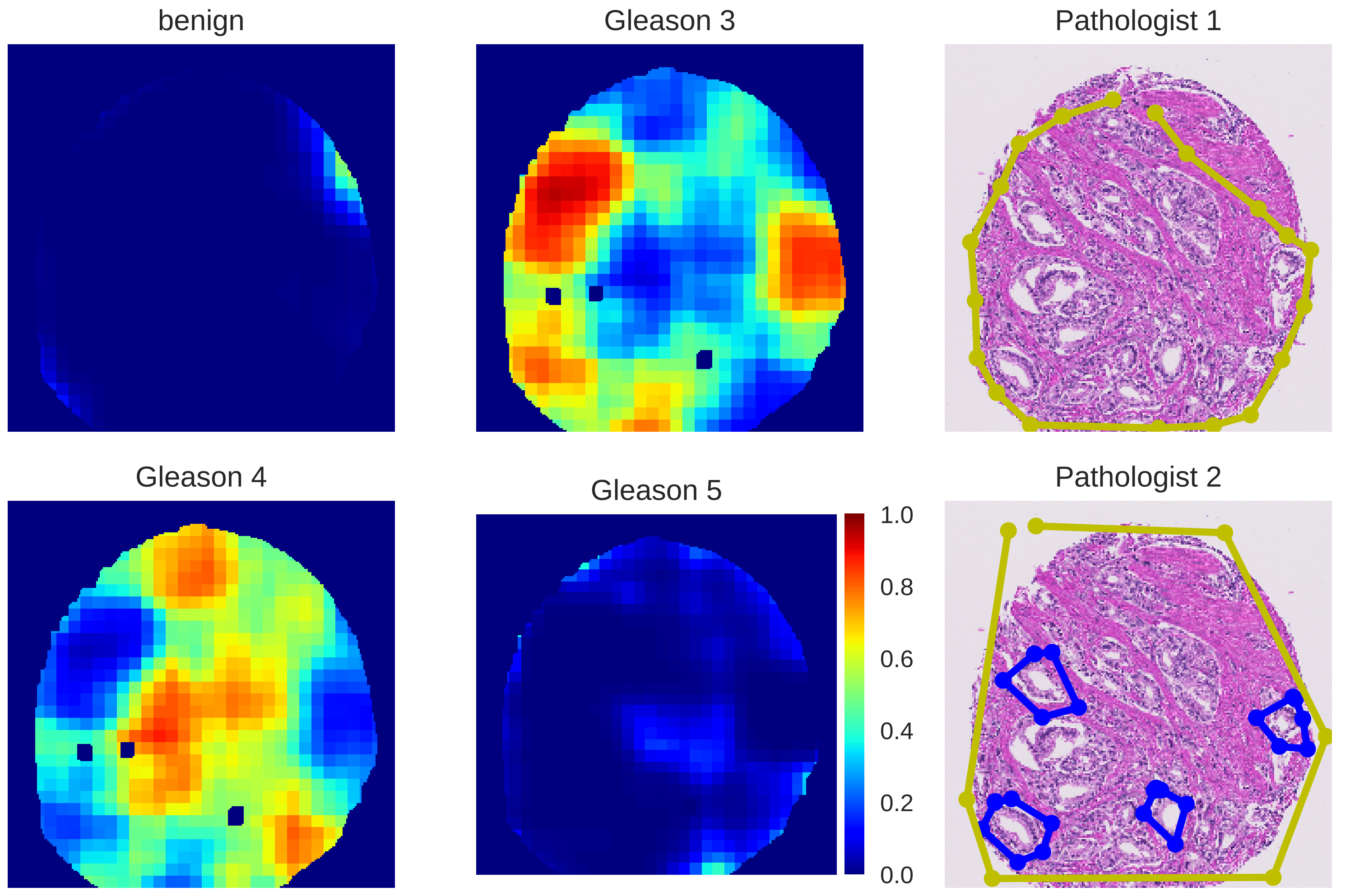

benign
Gleason 3
Pathologist 1
Gleason 4
Gleason 5
Pathologist 2
1.0
0.8
0.6
0.4
0.2
0.0

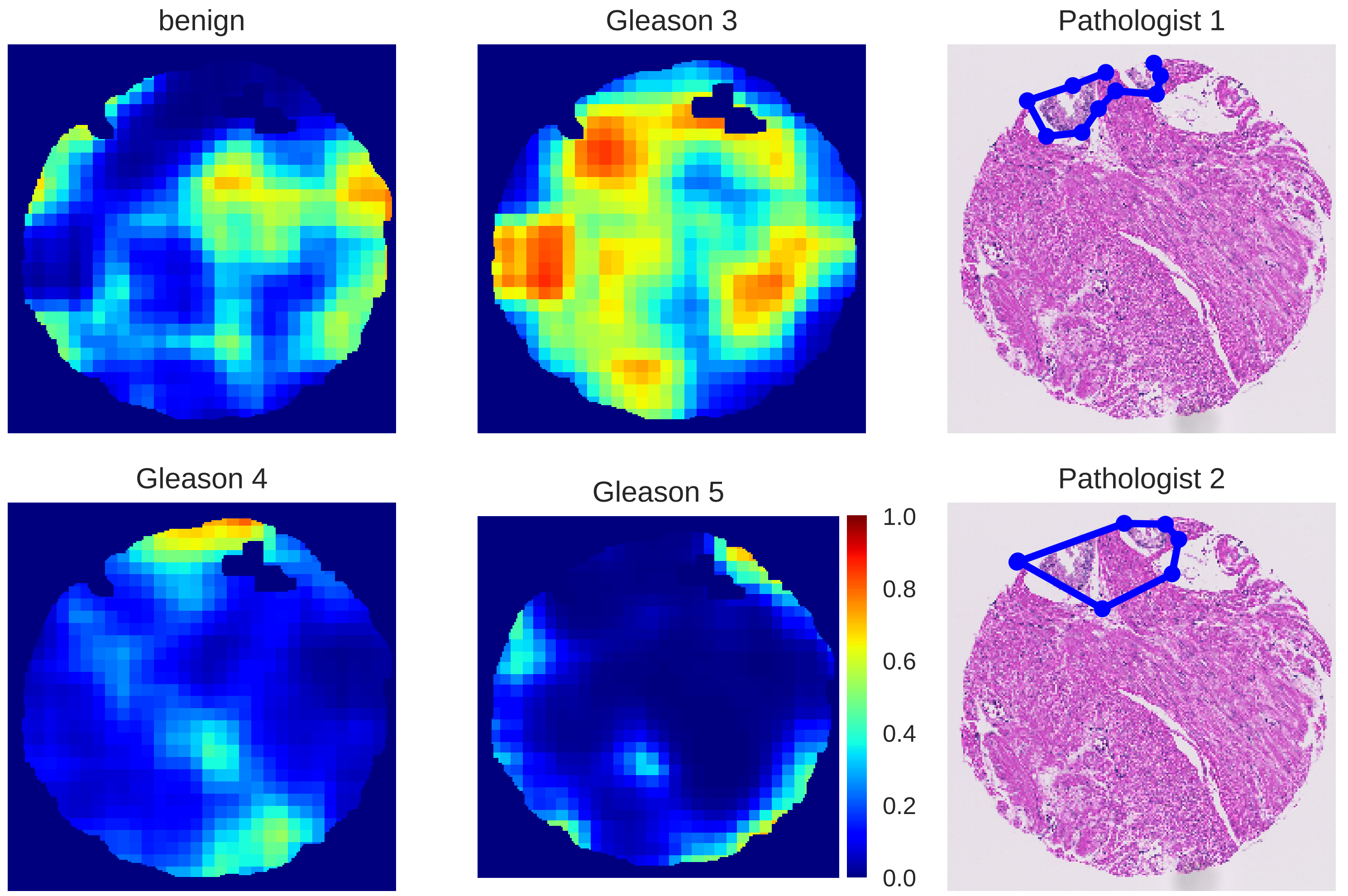

benign
Gleason 3
Pathologist 1
Gleason 4
Gleason 5
Pathologist 2
1.0
0.8
0.6
0.4
0.2
0.0

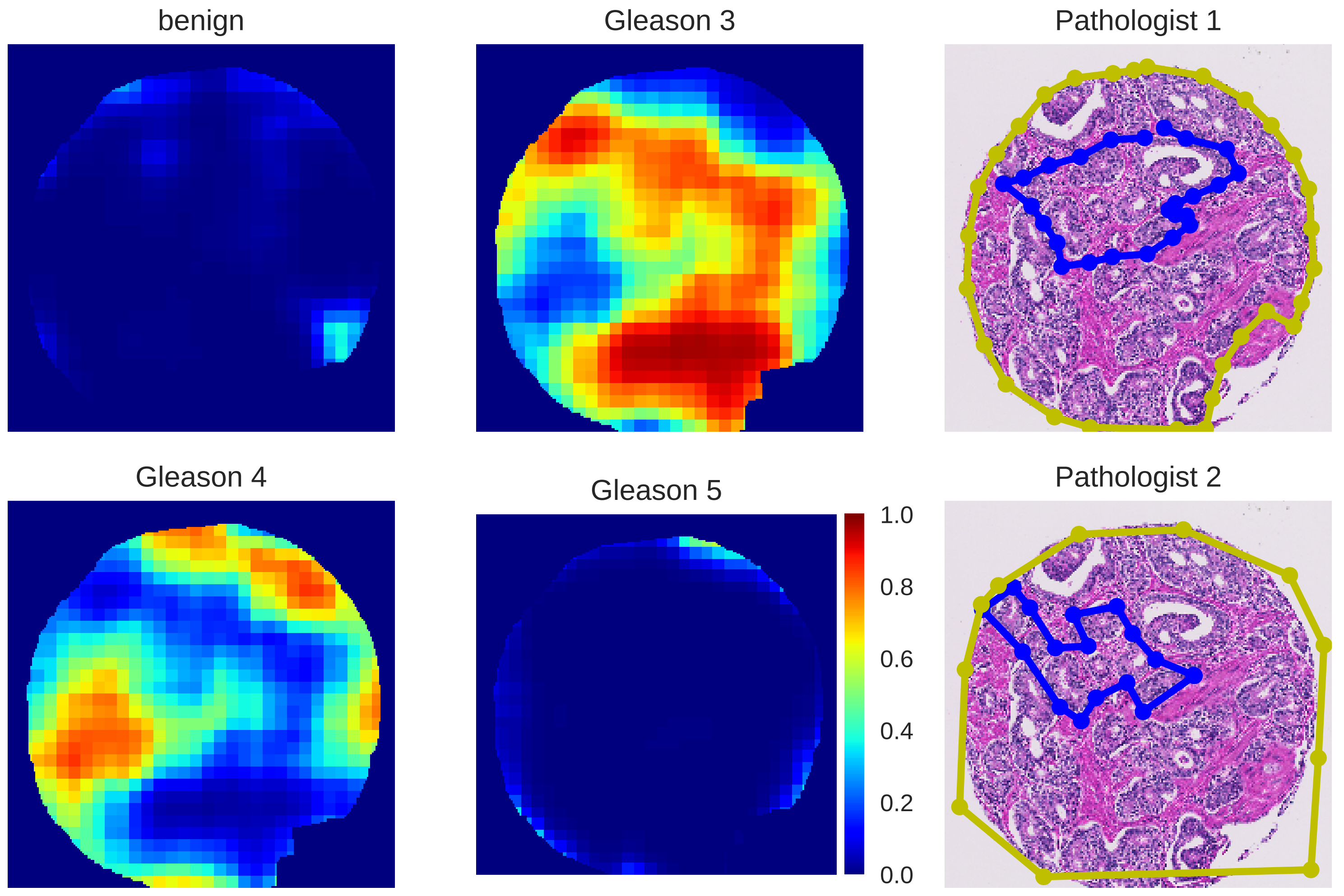
benign
Gleason 3
Pathologist 1
Gleason 4
Gleason 5
Pathologist 2
1.0
0.8
0.6
0.4
0.2
0.0

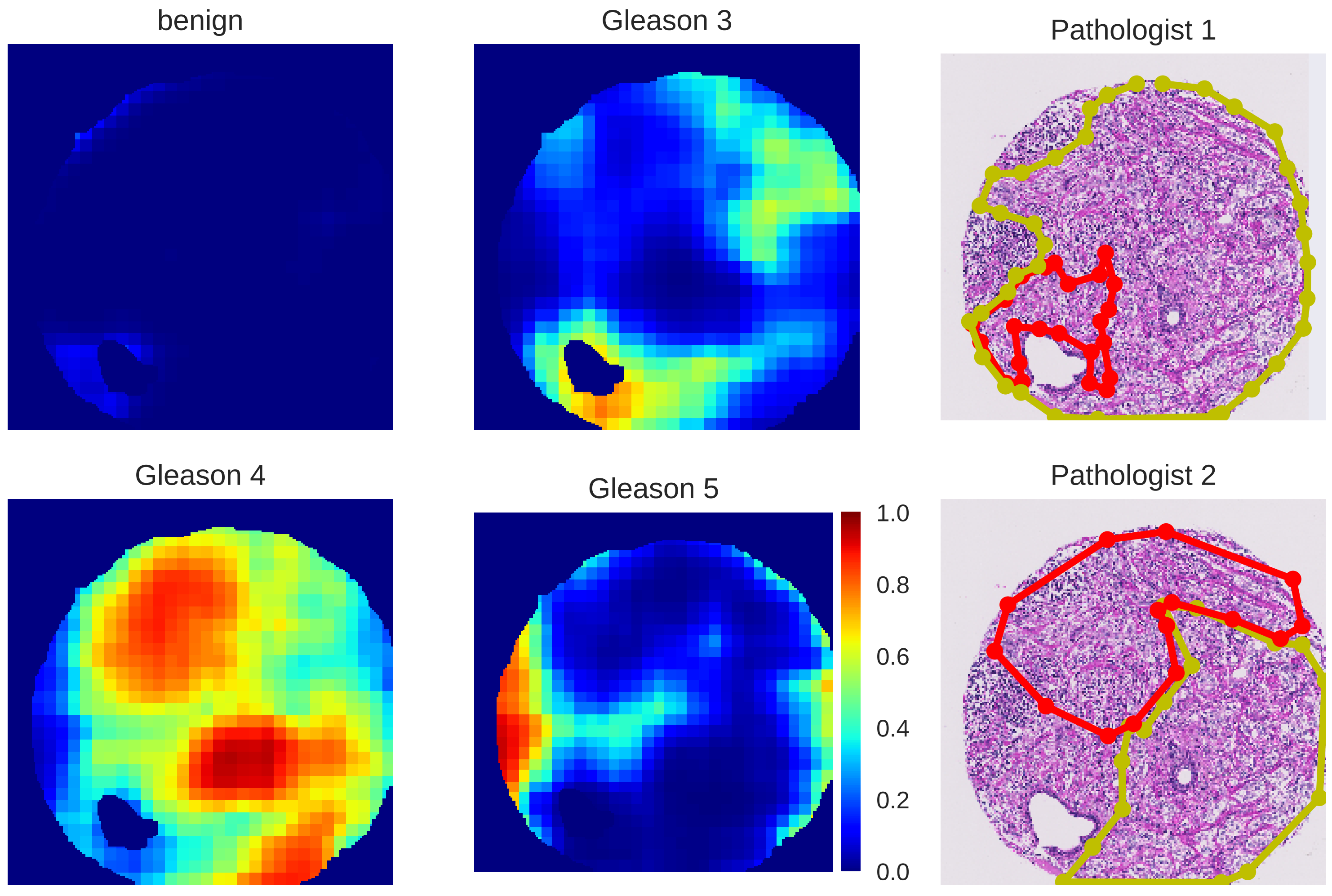

benign
Gleason 3
Pathologist 1
Gleason 4
Gleason 5
Pathologist 2
1.0
0.8
0.6
0.4
0.2
0.0

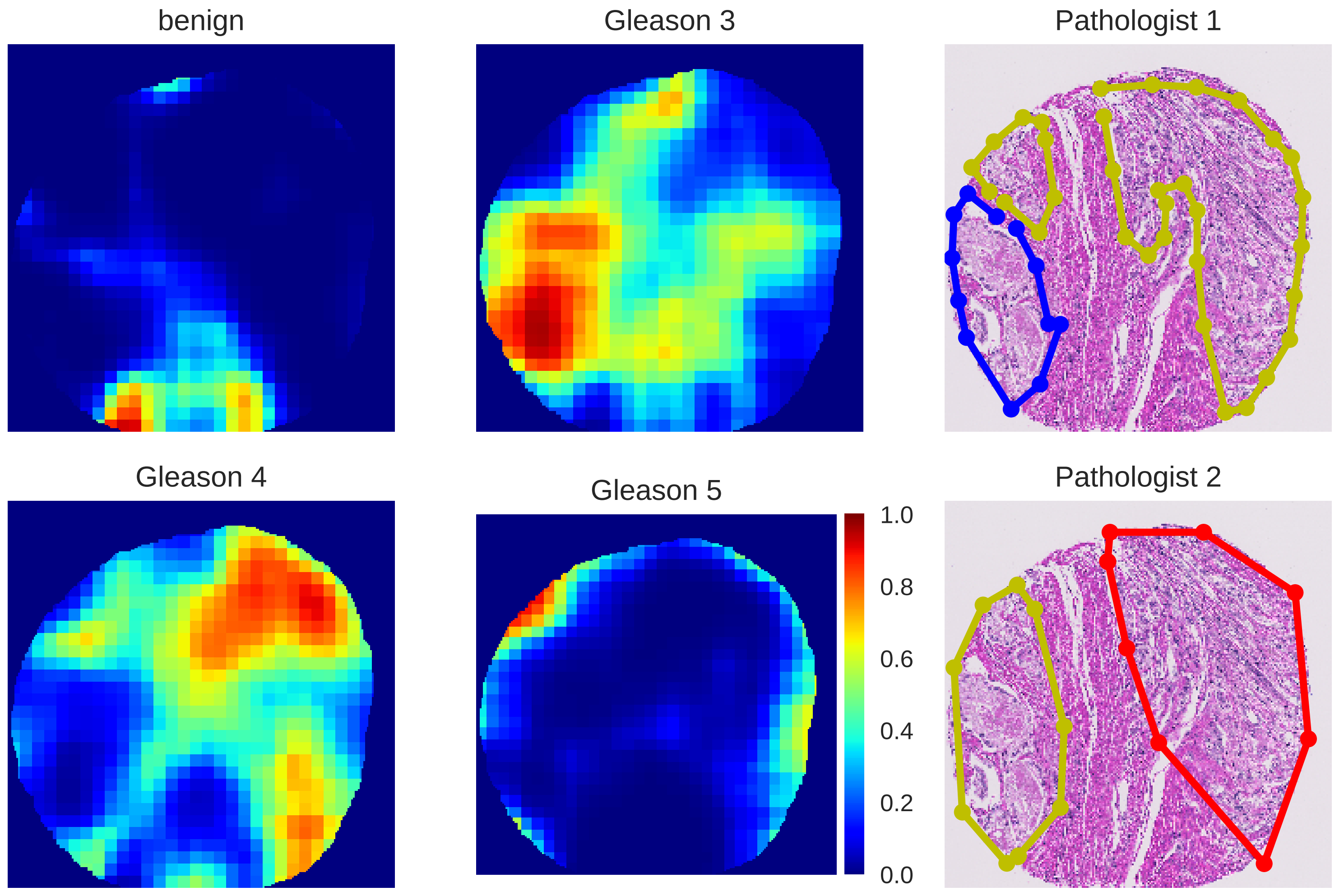

benign
Gleason 3
Pathologist 1
Gleason 4
Gleason 5
Pathologist 2
1.0
0.8
0.6
0.4
0.2
0.0

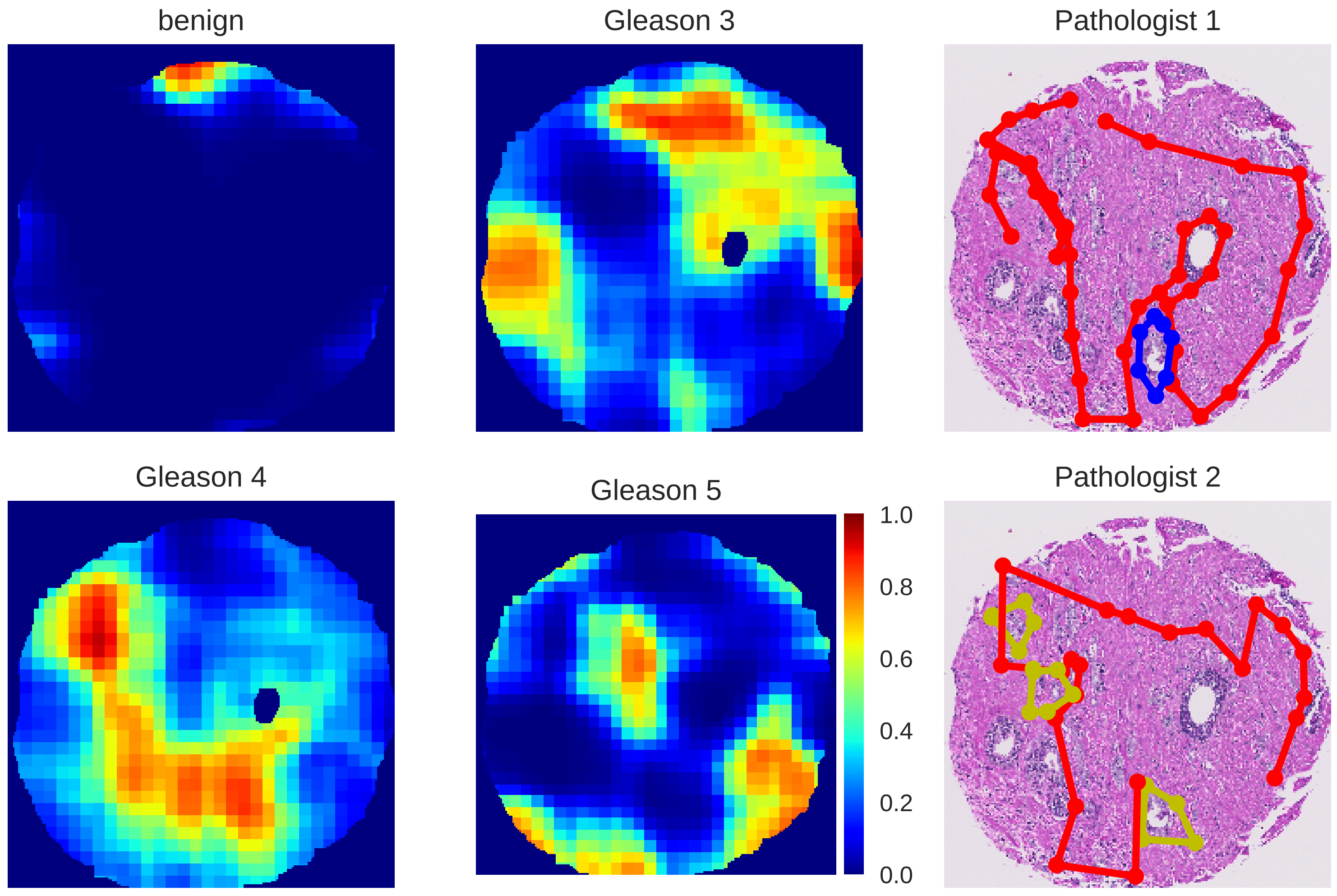
benign
Gleason 3
Pathologist 1
Gleason 4
Gleason 5
Pathologist 2
1.0
0.8
0.6
0.4
0.2
0.0

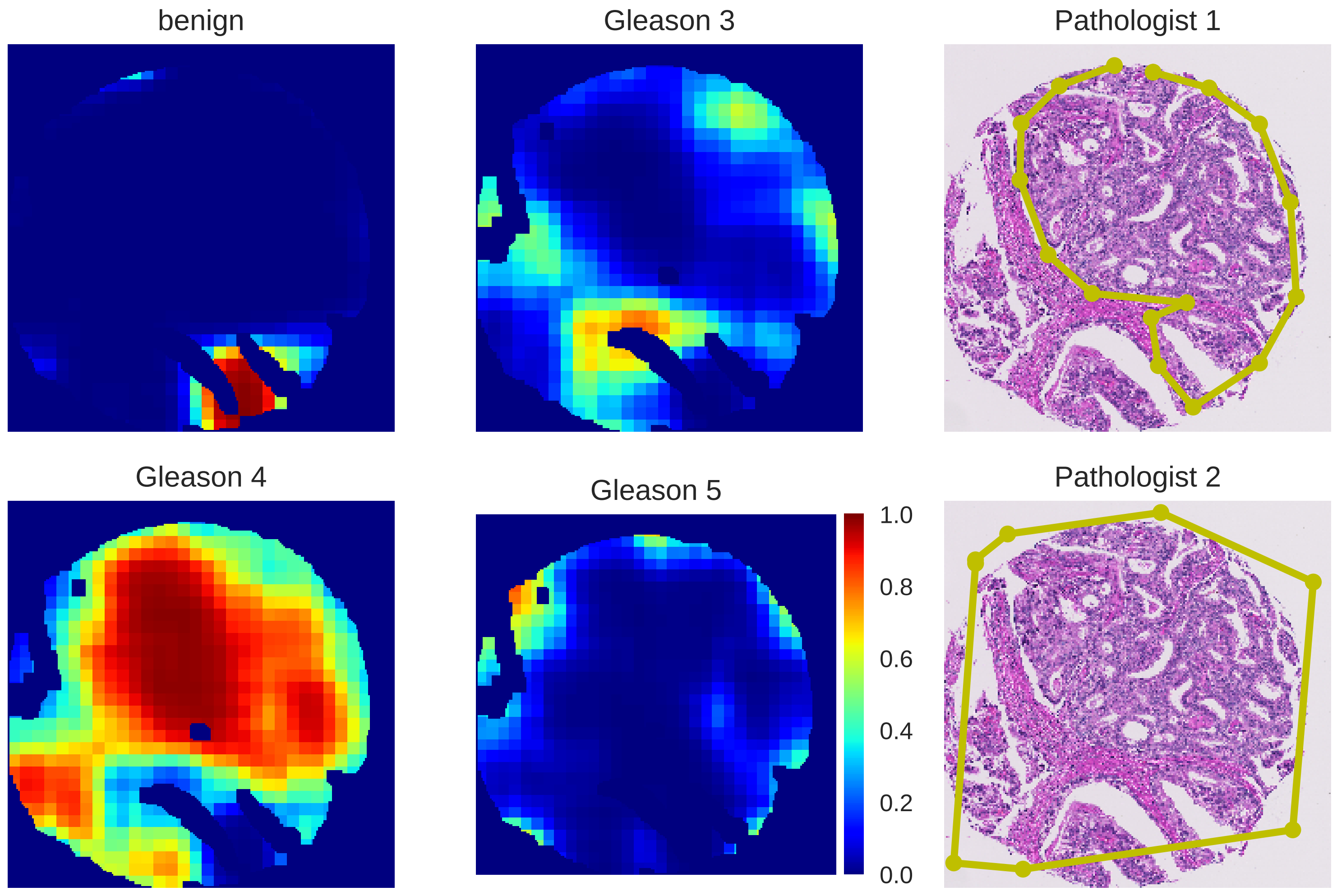

benign
Gleason 3
Pathologist 1
Gleason 4
Gleason 5
Pathologist 2
1.0
0.8
0.6
0.4
0.2
0.0

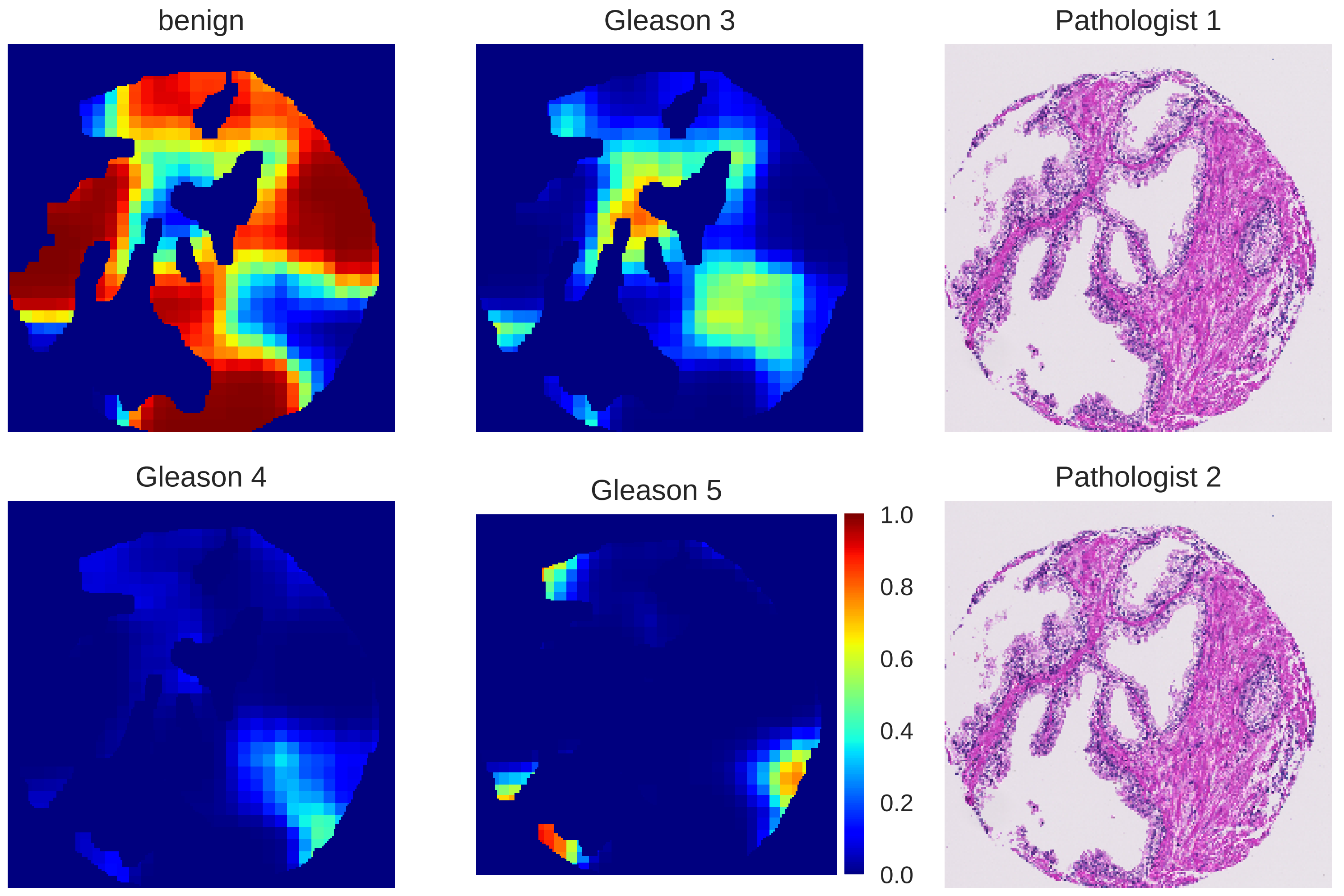
benign
Gleason 3
Pathologist 1
Gleason 4
Gleason 5
Pathologist 2
1.0
0.8
0.6
0.4
0.2
0.0

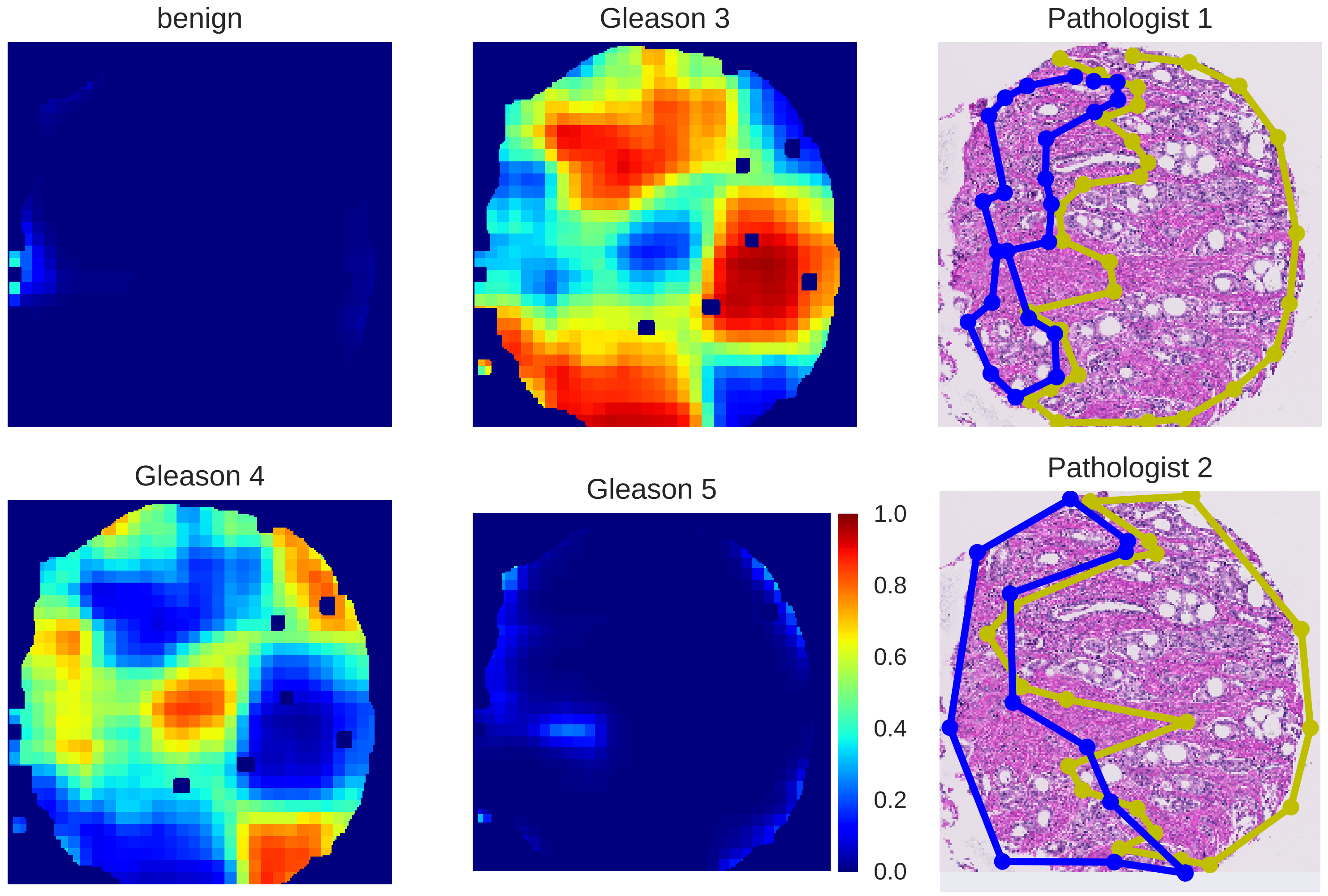
benign
Gleason 3
Pathologist 1
Gleason 4
Gleason 5
Pathologist 2
1.0
0.8
0.6
0.4
0.2
0.0

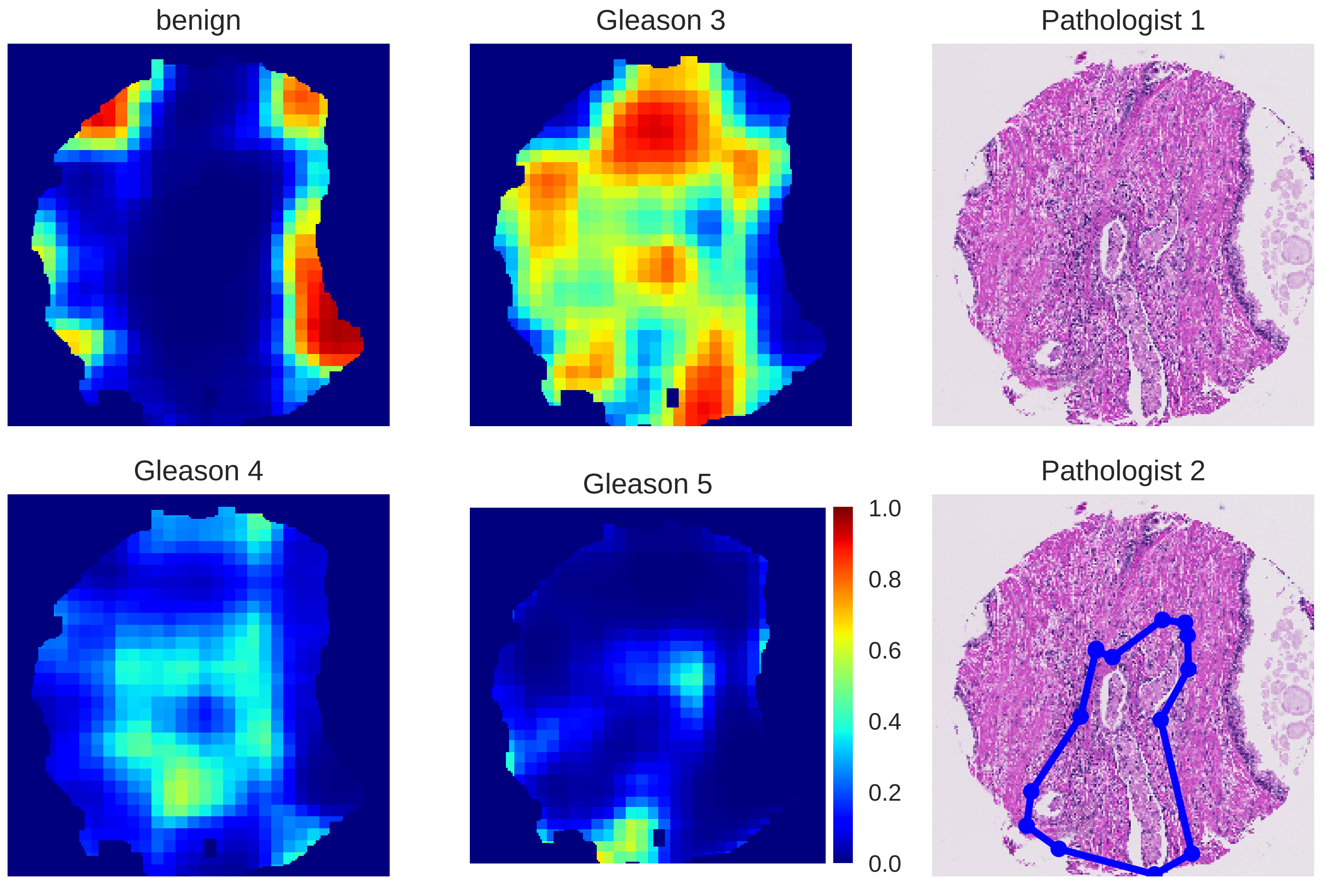

benign
Gleason 3
Pathologist 1
Gleason 4
Gleason 5
Pathologist 2
1.0
0.8
0.6
0.4
0.2
0.0

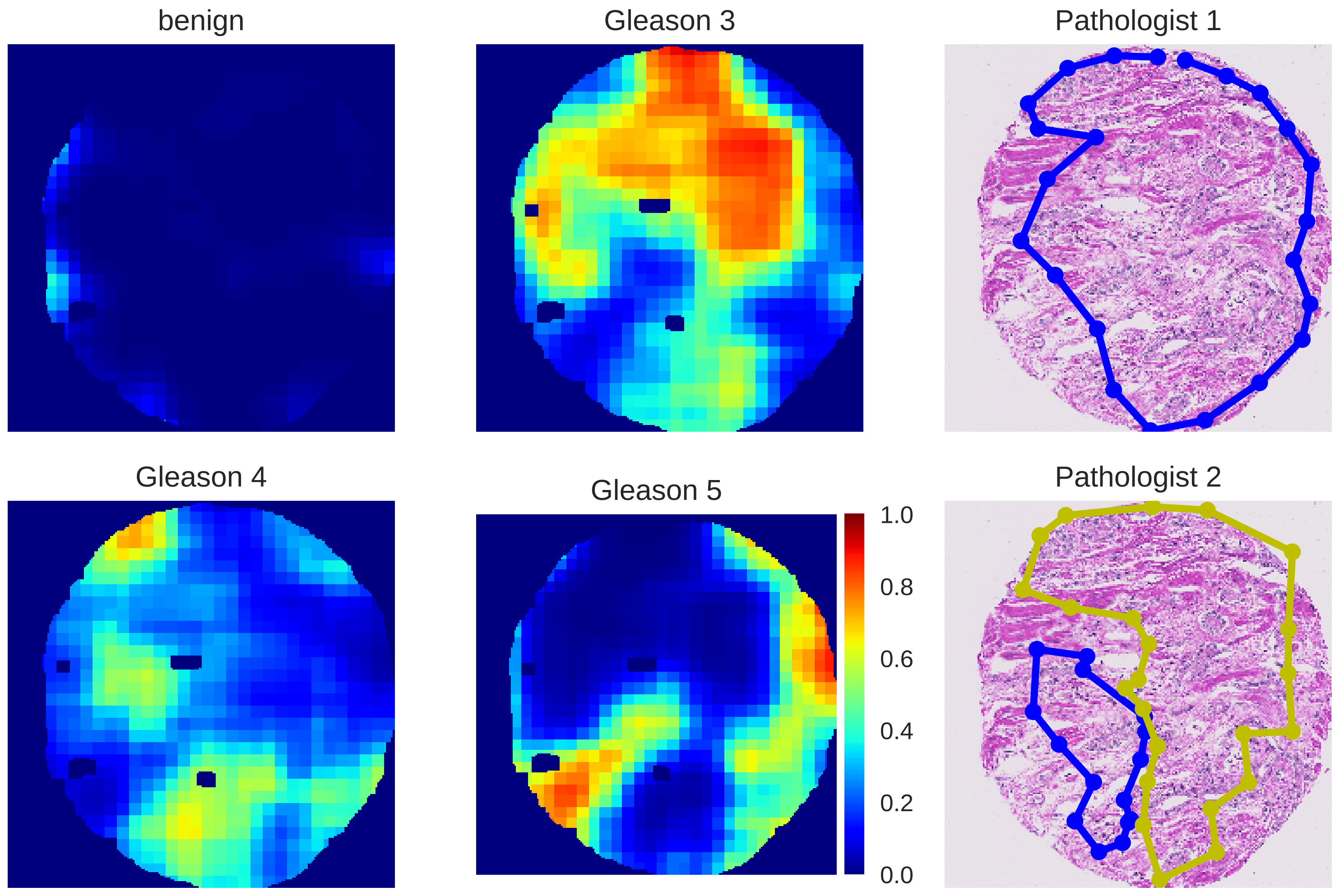
benign
Gleason 3
Pathologist 1
Gleason 4
Gleason 5
1.0
0.8
0.6
0.4
0.2
0.0
Pathologist 2

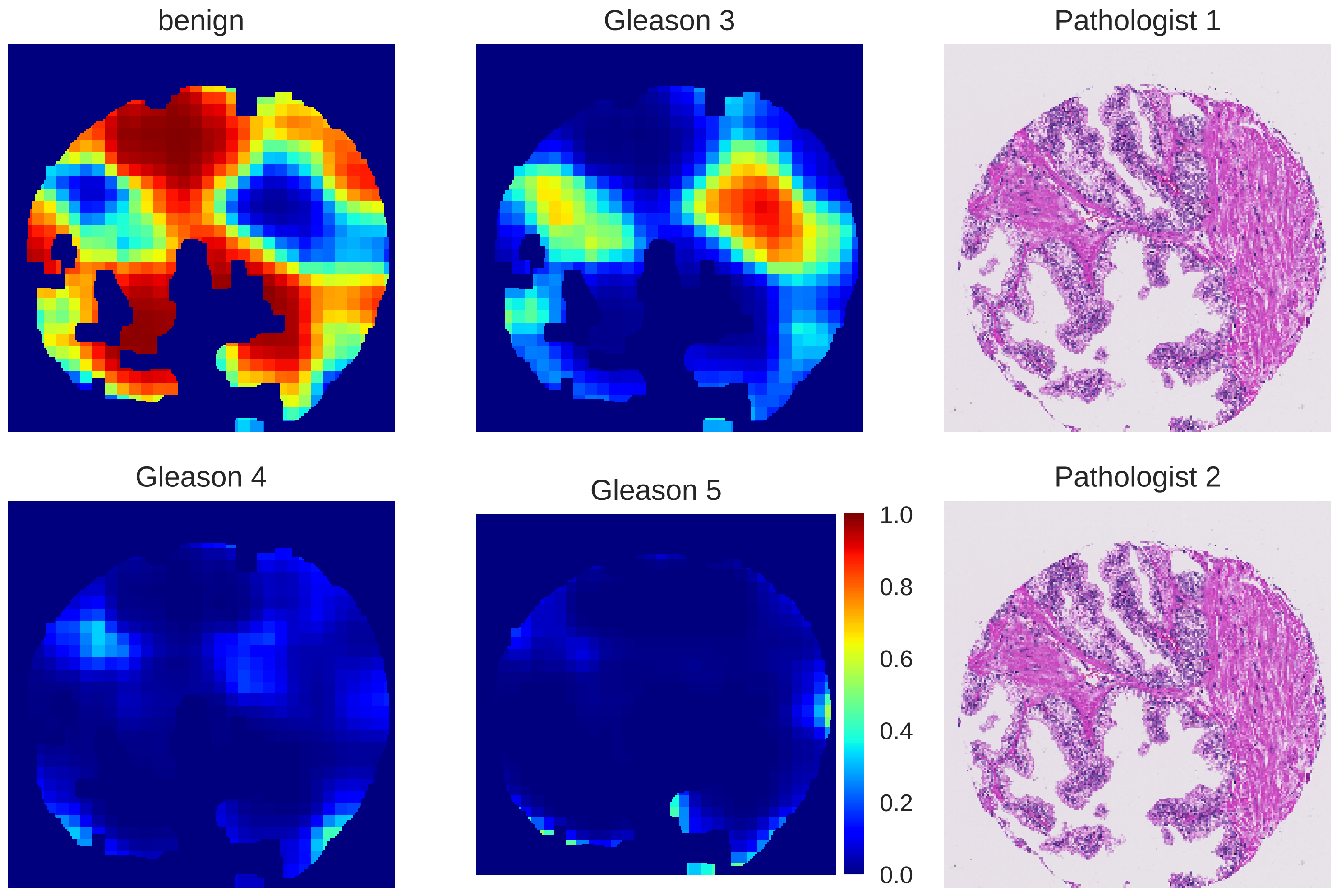
benign
Gleason 3
Pathologist 1
Gleason 4
Gleason 5
Pathologist 2
1.0
0.8
0.6
0.4
0.2
0.0

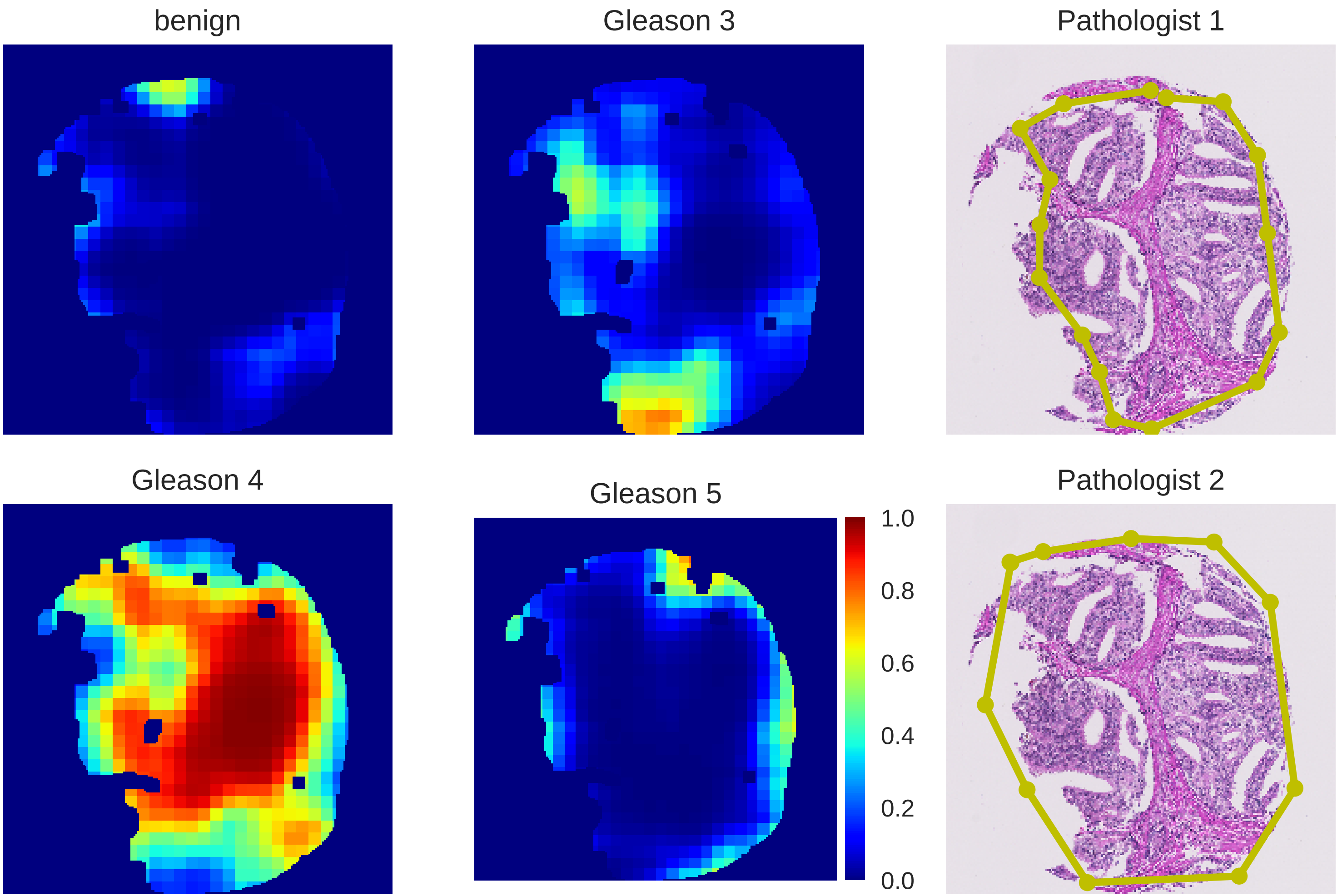

benign
Gleason 3
Pathologist 1
Gleason 4
Gleason 5
Pathologist 2
1.0
0.8
0.6
0.4
0.2
0.0

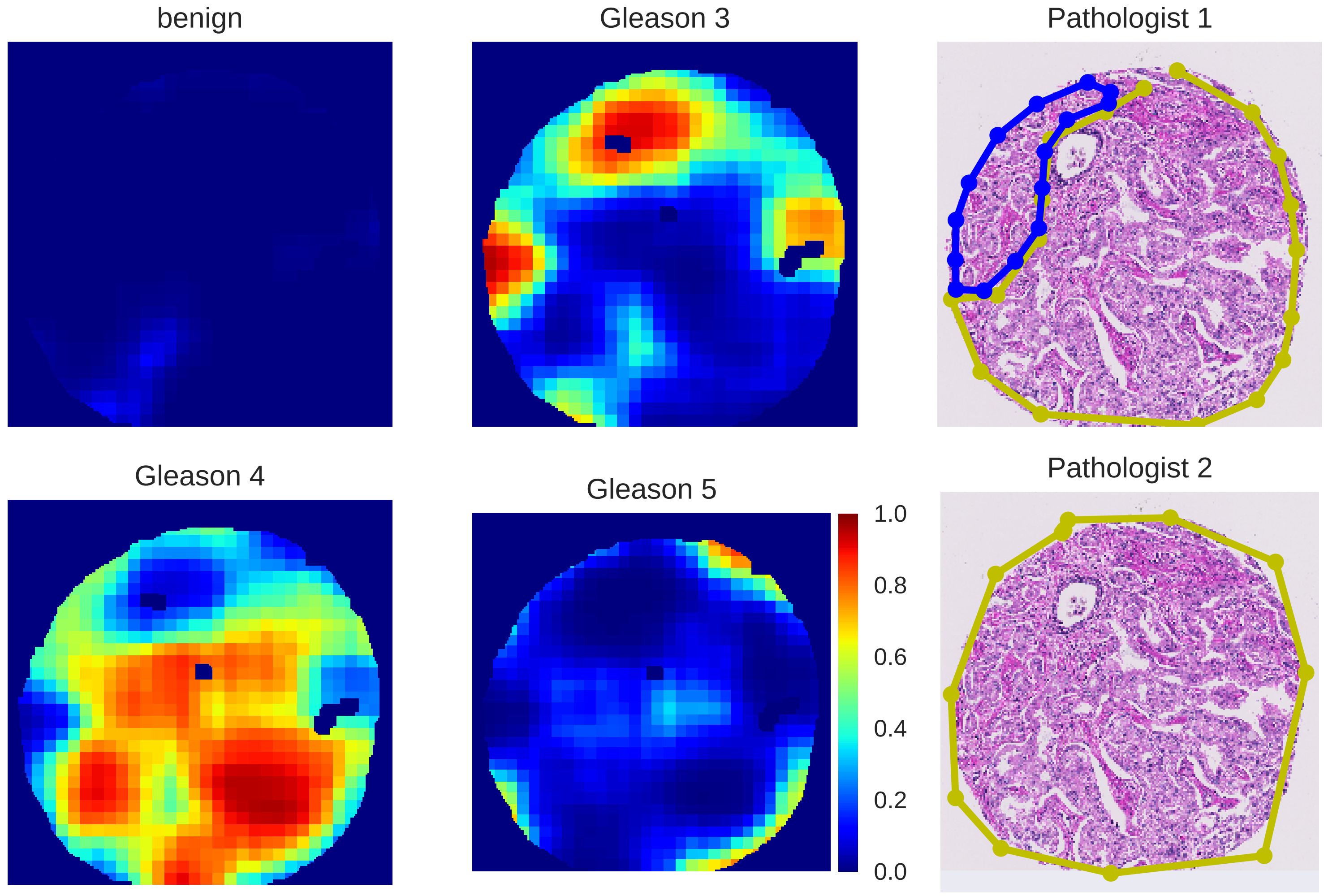
benign
Gleason 3
Pathologist 1
Gleason 4
Gleason 5
Pathologist 2
1.0
0.8
0.6
0.4
0.2
0.0

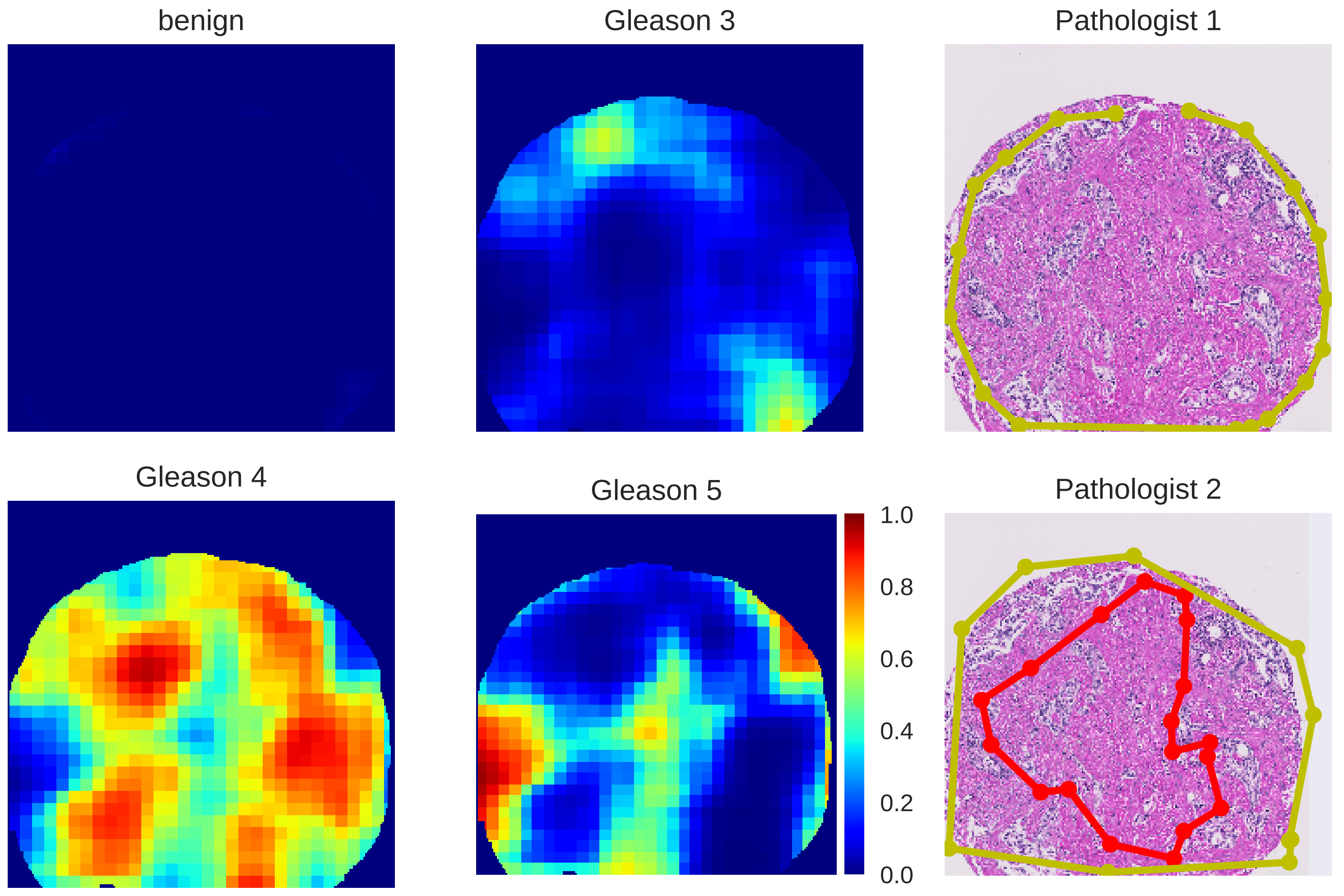
benign
Gleason 3
Pathologist 1
Gleason 4
Gleason 5
1.0
0.8
0.6
0.4
0.2
0.0
Pathologist 2

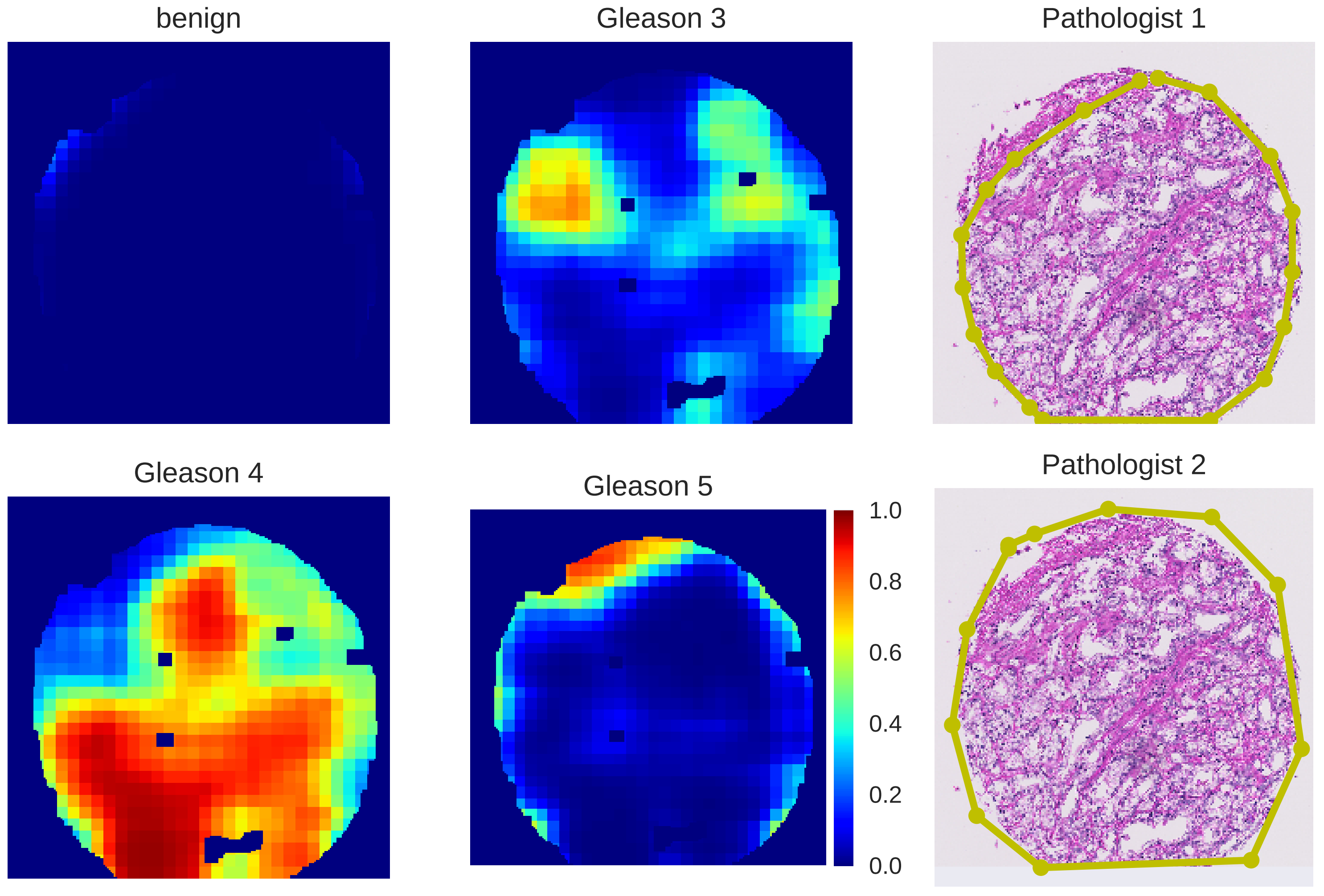

benign
Gleason 3
Pathologist 1
Gleason 4
Gleason 5
Pathologist 2
1.0
0.8
0.6
0.4
0.2
0.0

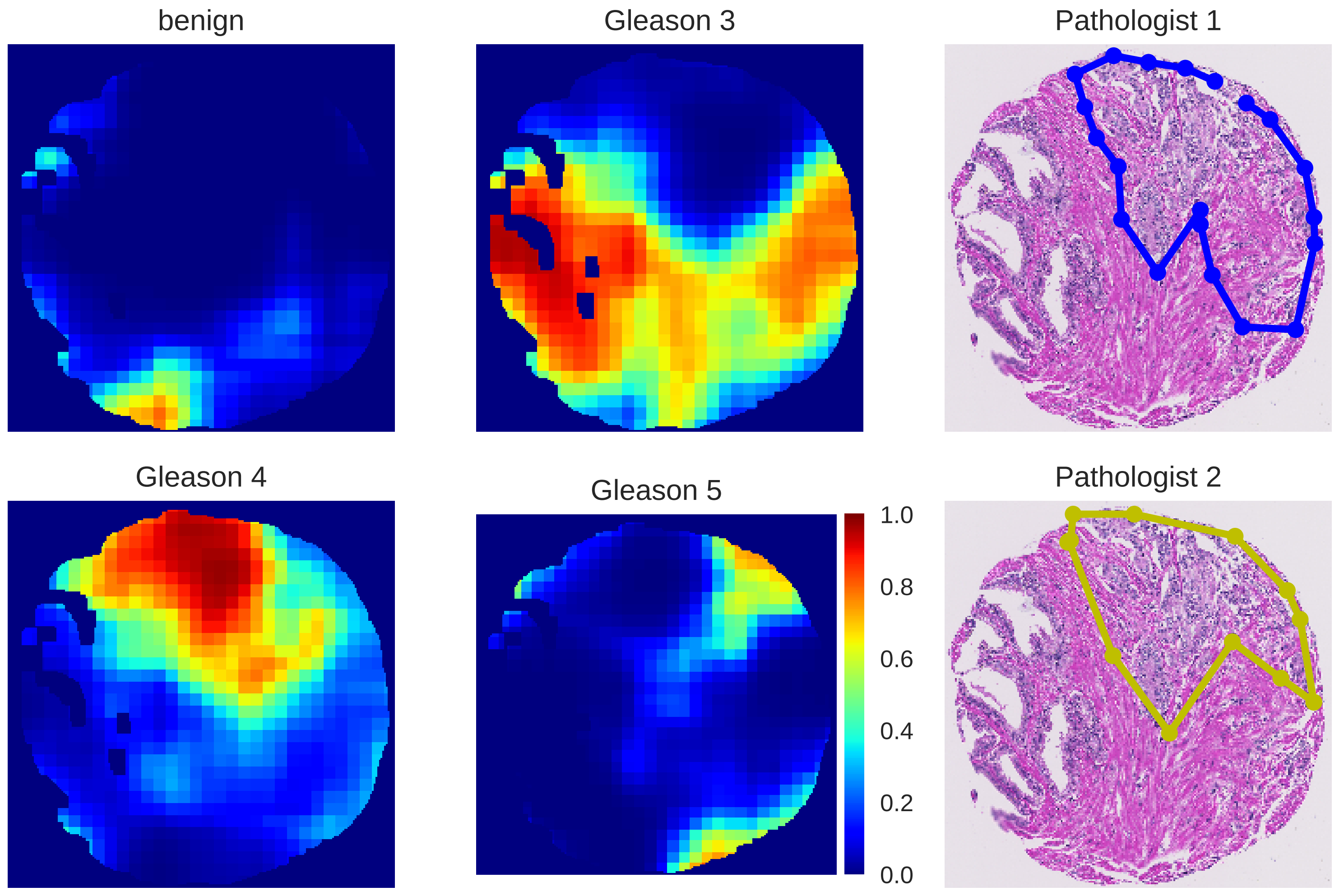

benign
Gleason 3
Pathologist 1
Gleason 4
Gleason 5
Pathologist 2
1.0
0.8
0.6
0.4
0.2
0.0

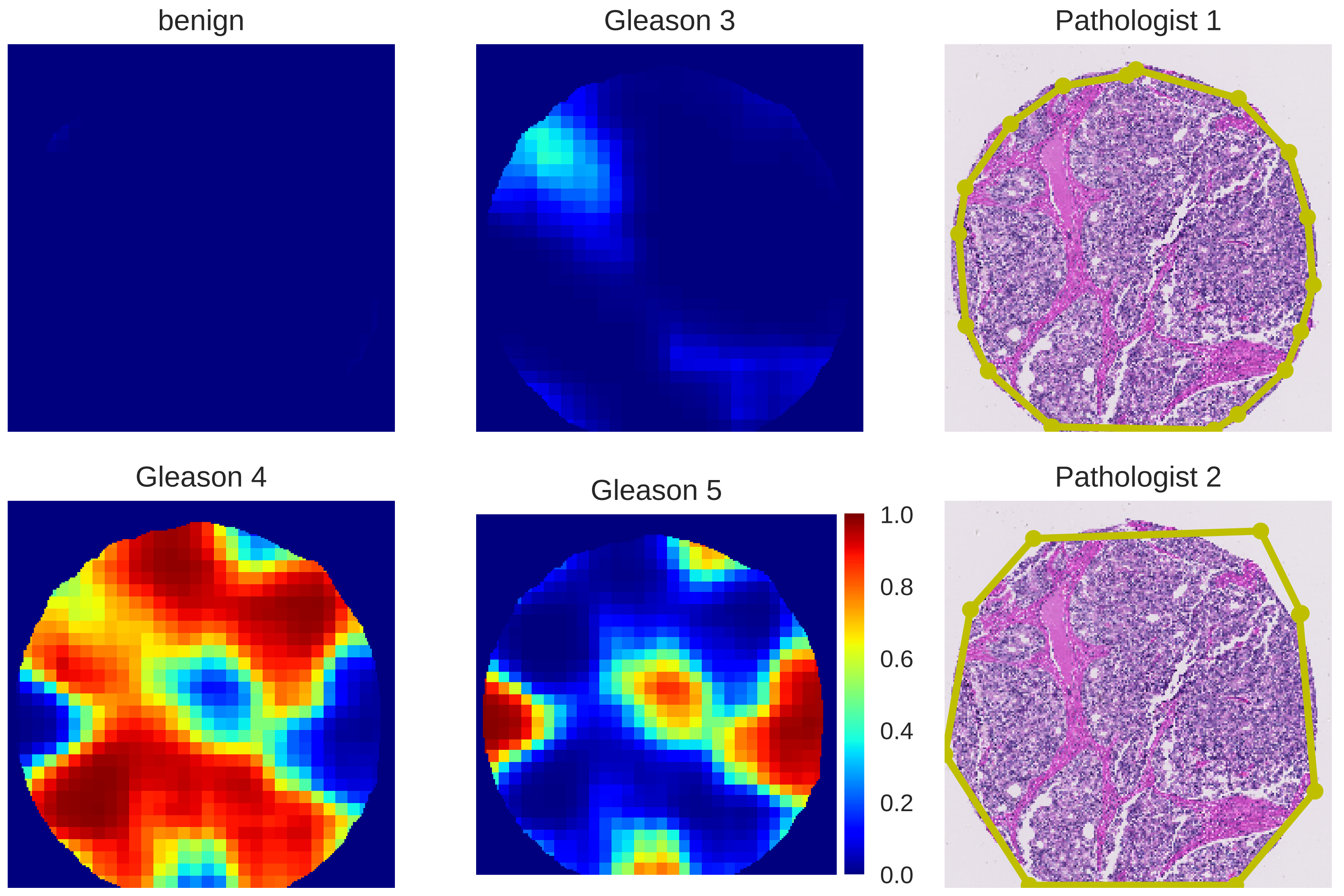

benign
Gleason 3
Pathologist 1
Gleason 4
Gleason 5
Pathologist 2
1.0
0.8
0.6
0.4
0.2
0.0

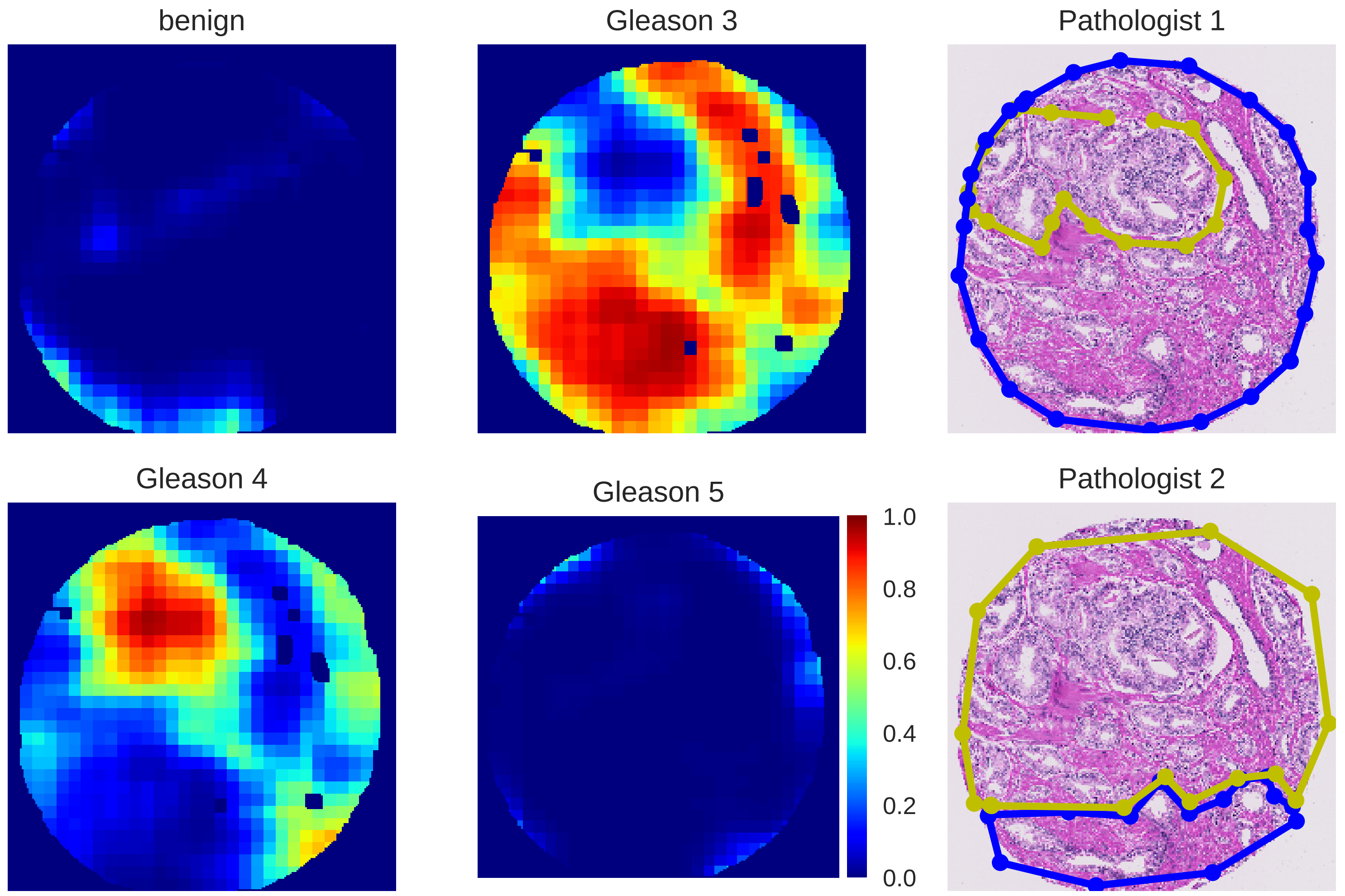
benign
Gleason 3
Pathologist 1
Gleason 4
Gleason 5
Pathologist 2
1.0
0.8
0.6
0.4
0.2
0.0

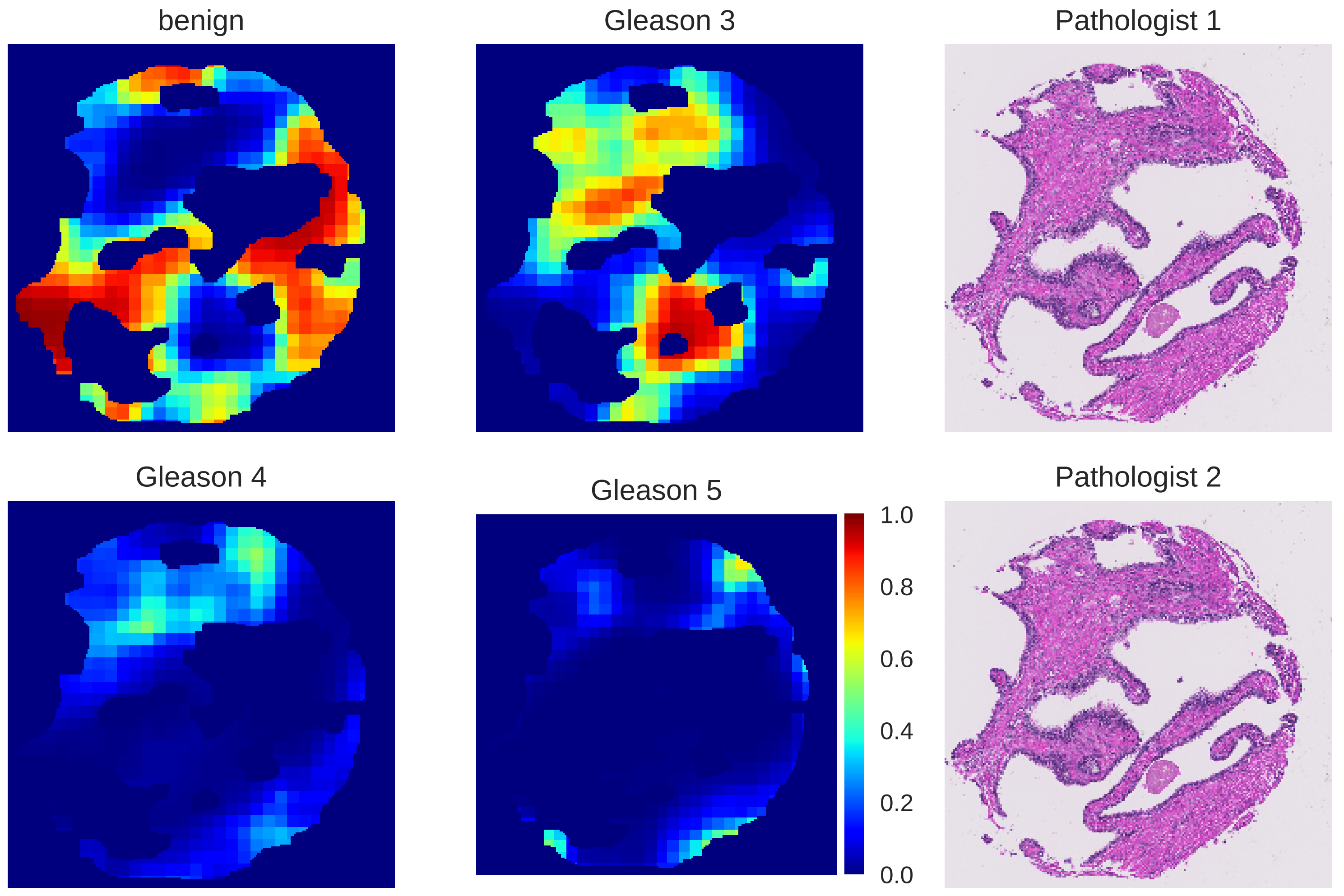

benign
Gleason 3
Pathologist 1
Gleason 4
Gleason 5
Pathologist 2
1.0
0.8
0.6
0.4
0.2
0.0

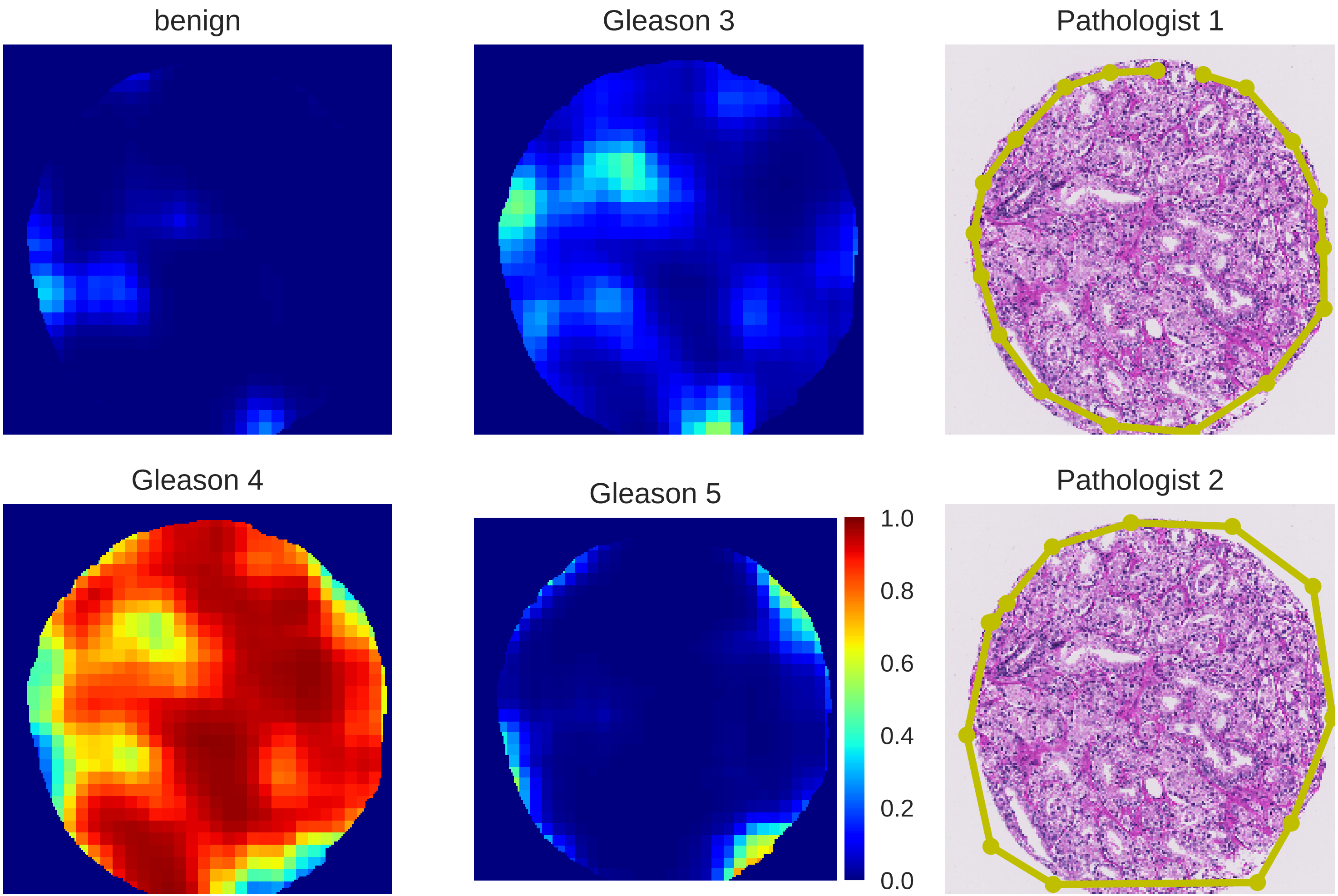

benign
Gleason 3
Pathologist 1
Gleason 4
Gleason 5
Pathologist 2
1.0
0.8
0.6
0.4
0.2
0.0

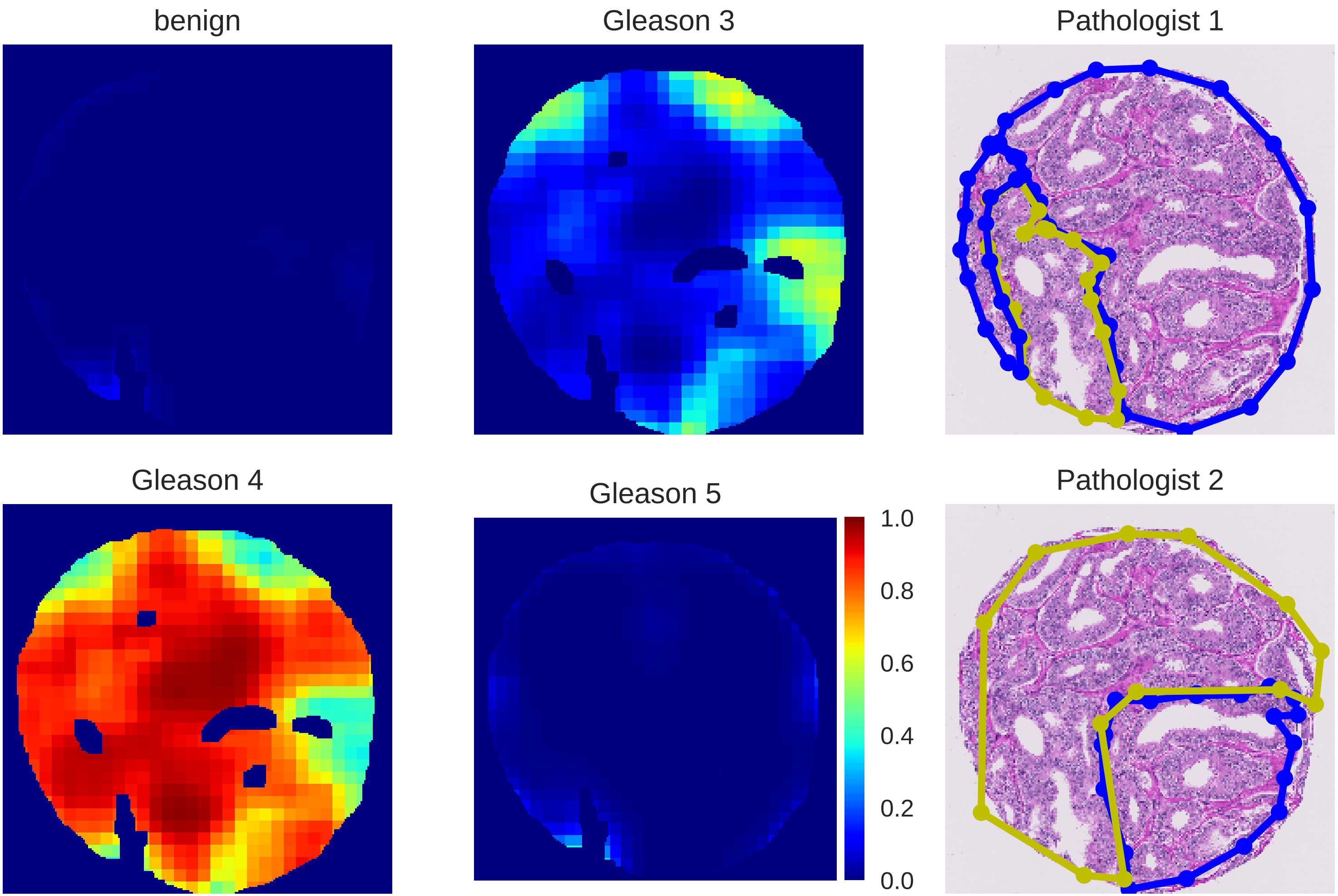

benign
Gleason 3
Pathologist 1
Gleason 4
Gleason 5
Pathologist 2
1.0
0.8
0.6
0.4
0.2
0.0

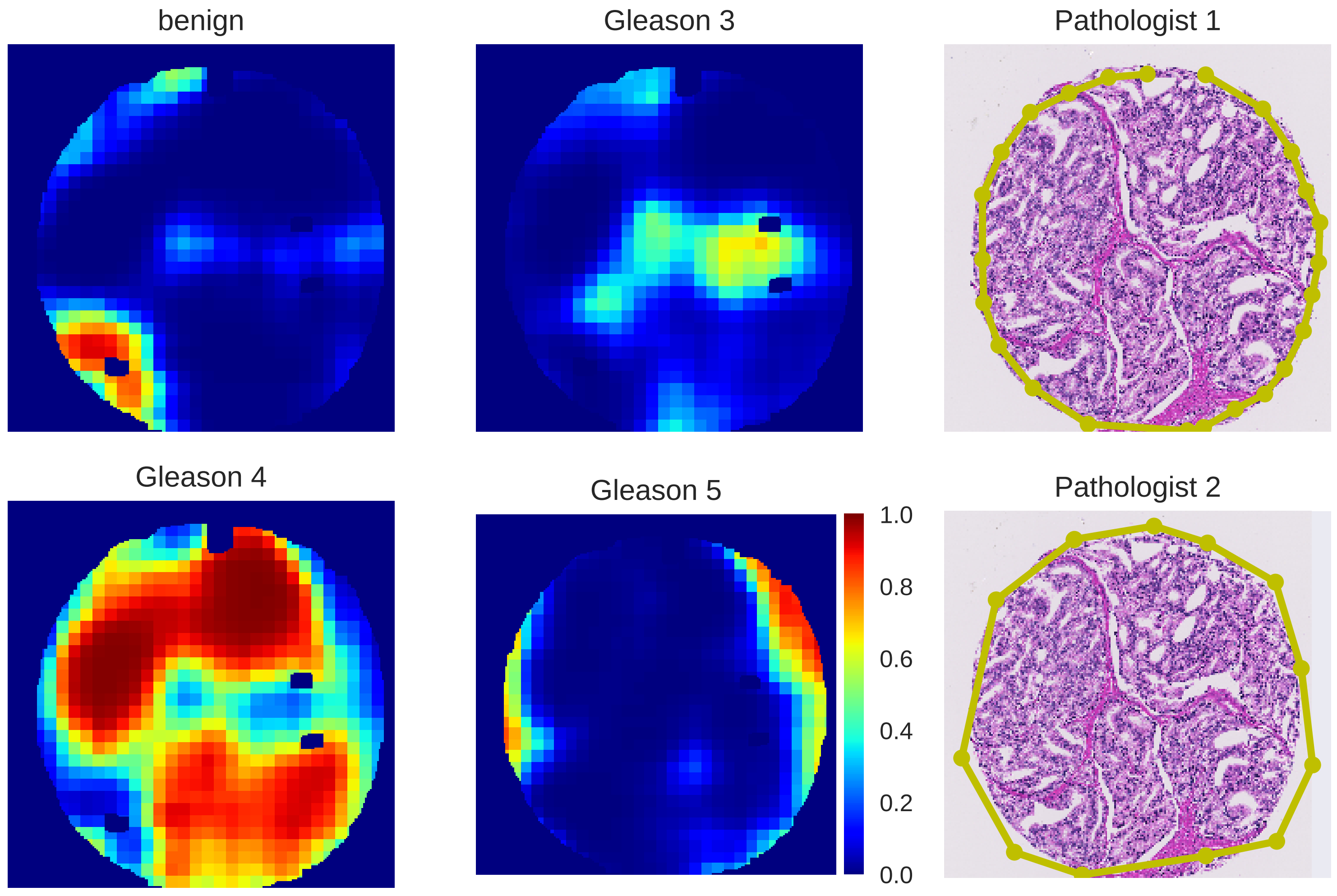
benign
Gleason 3
Pathologist 1
Gleason 4
Gleason 5
Pathologist 2
1.0
0.8
0.6
0.4
0.2
0.0

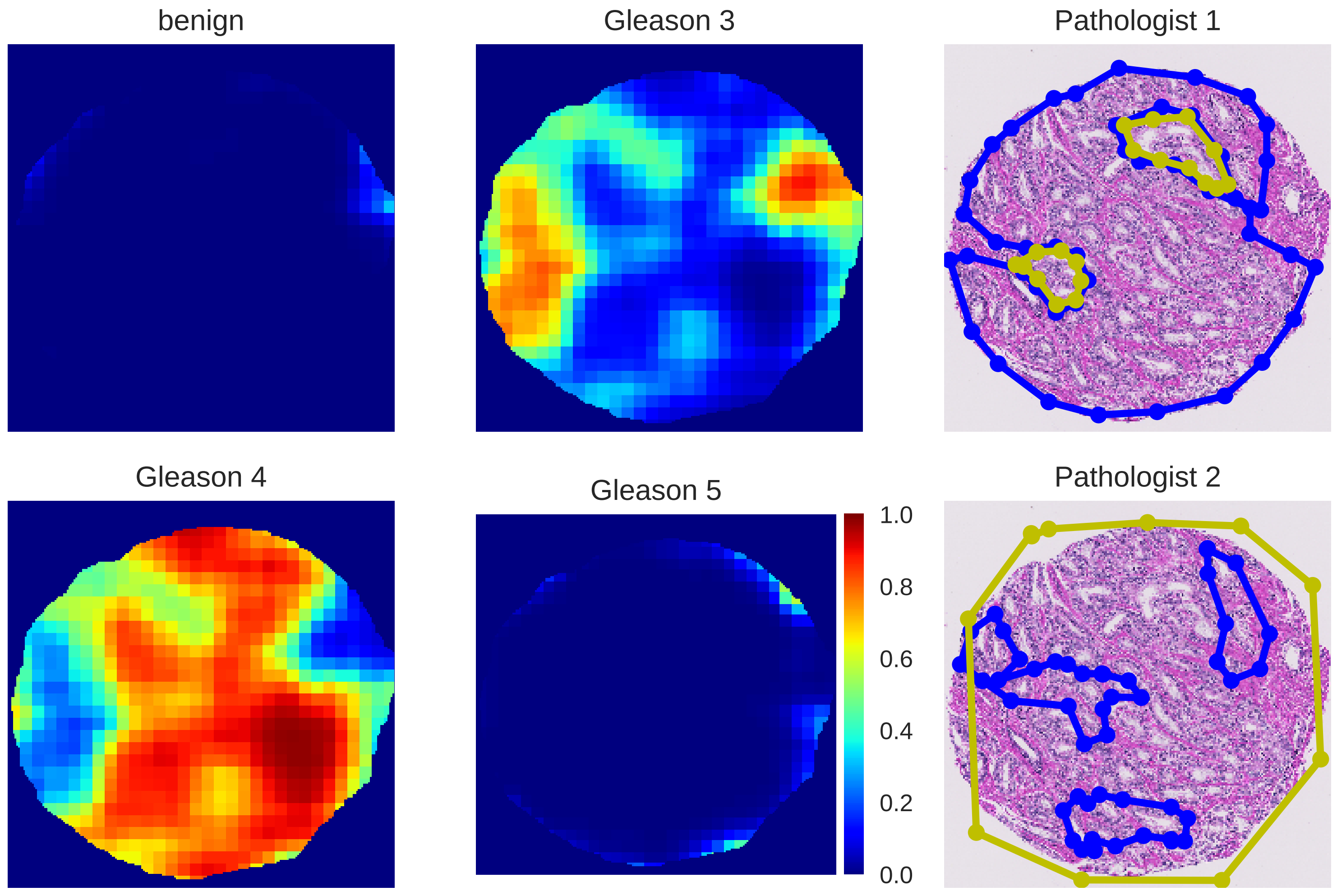
benign
Gleason 3
Pathologist 1
Gleason 4
Gleason 5
Pathologist 2
1.0
0.8
0.6
0.4
0.2
0.0

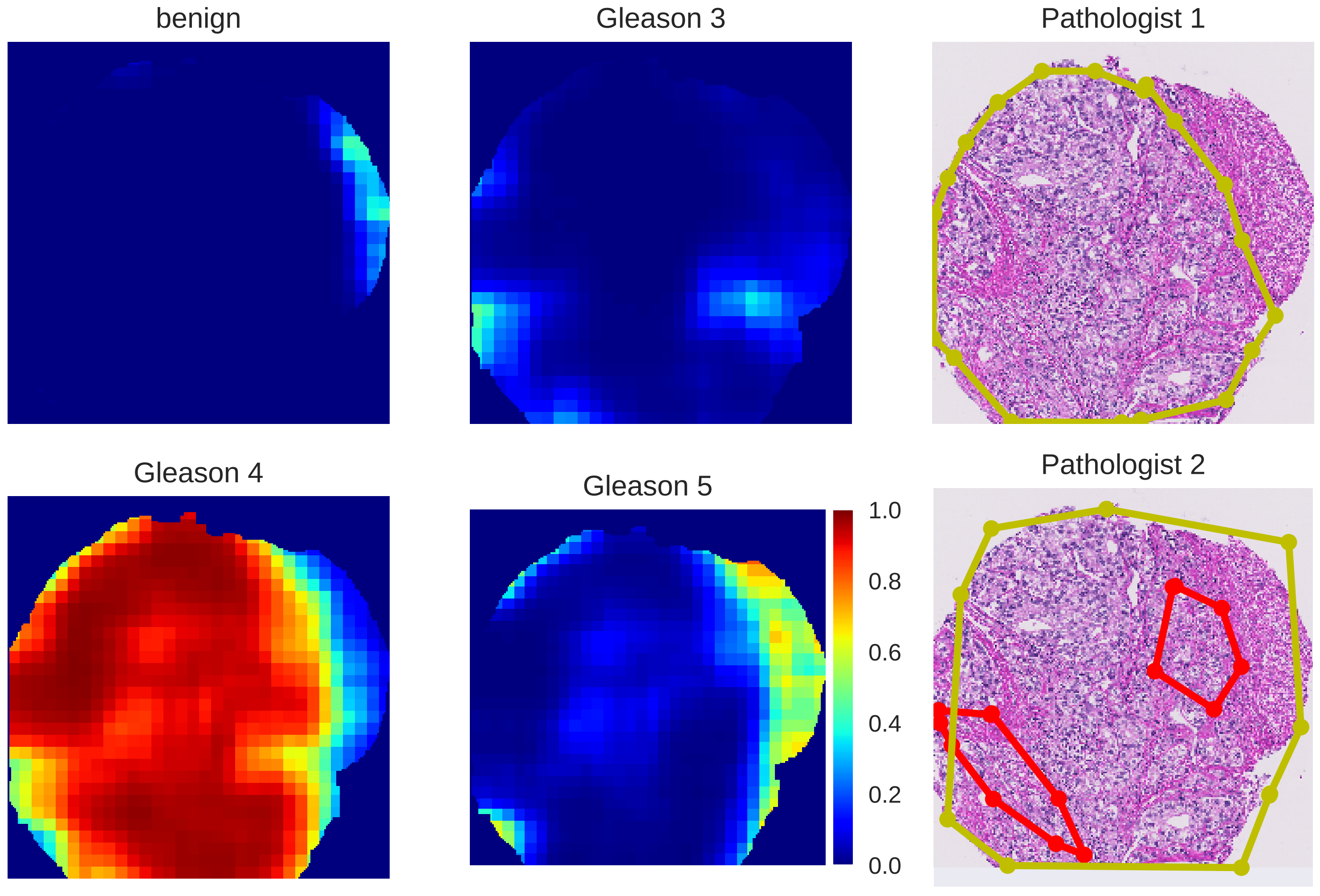

benign
Gleason 3
Pathologist 1
Gleason 4
Gleason 5
Pathologist 2
1.0
0.8
0.6
0.4
0.2
0.0

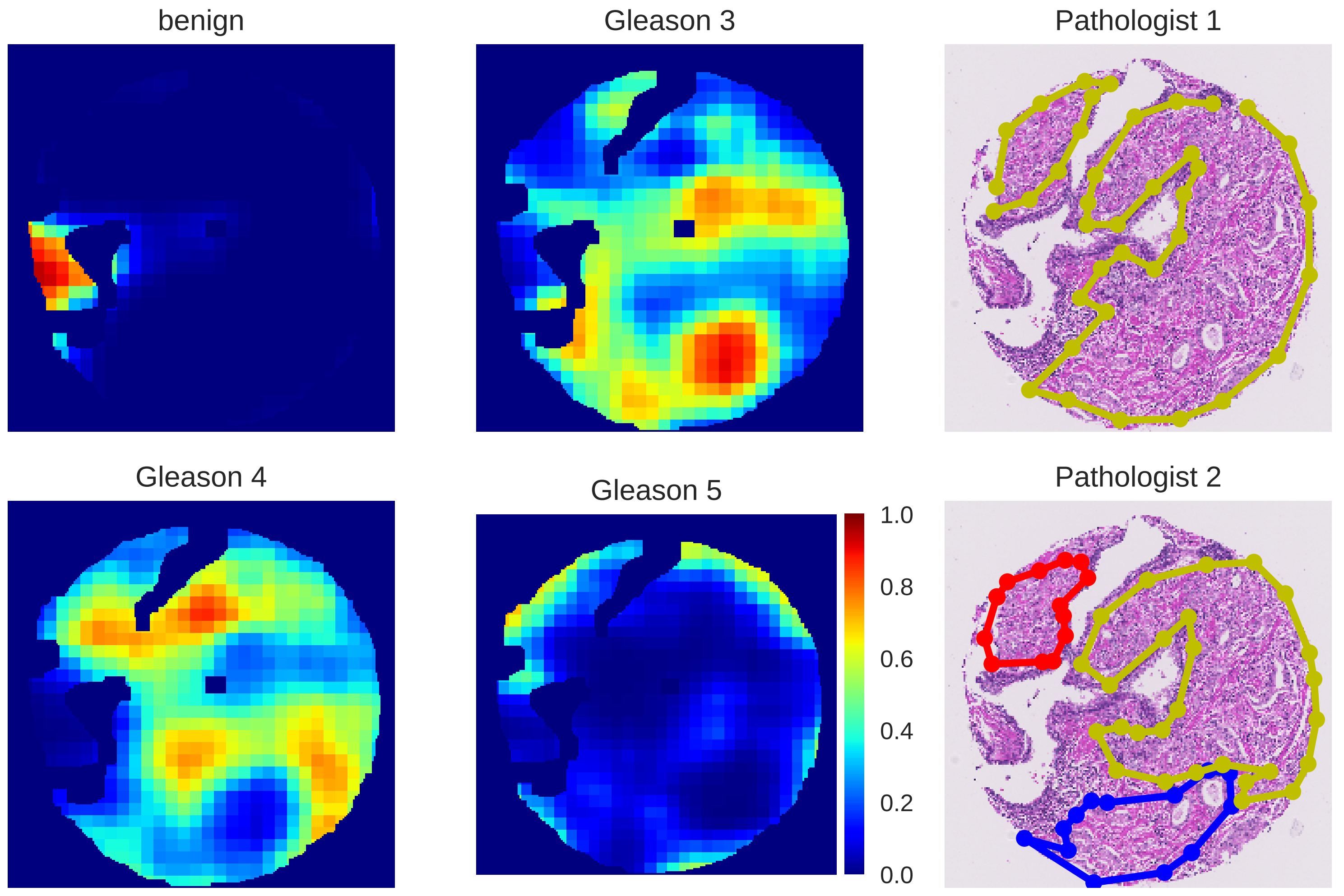
benign
Gleason 3
Pathologist 1
Gleason 4
Gleason 5
Pathologist 2
1.0
0.8
0.6
0.4
0.2
0.0

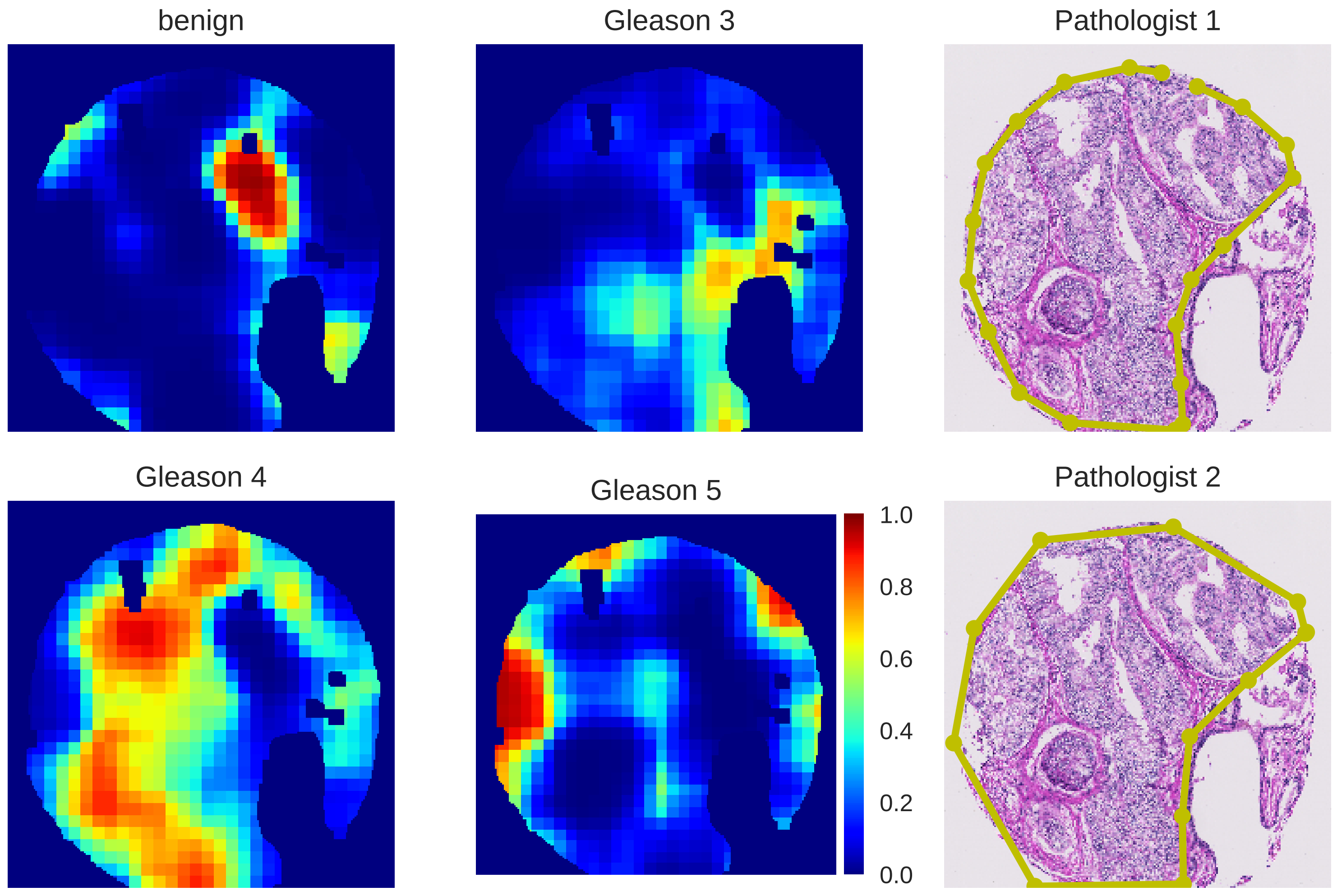

benign
Gleason 3
Pathologist 1
Gleason 4
Gleason 5
Pathologist 2
1.0
0.8
0.6
0.4
0.2
0.0

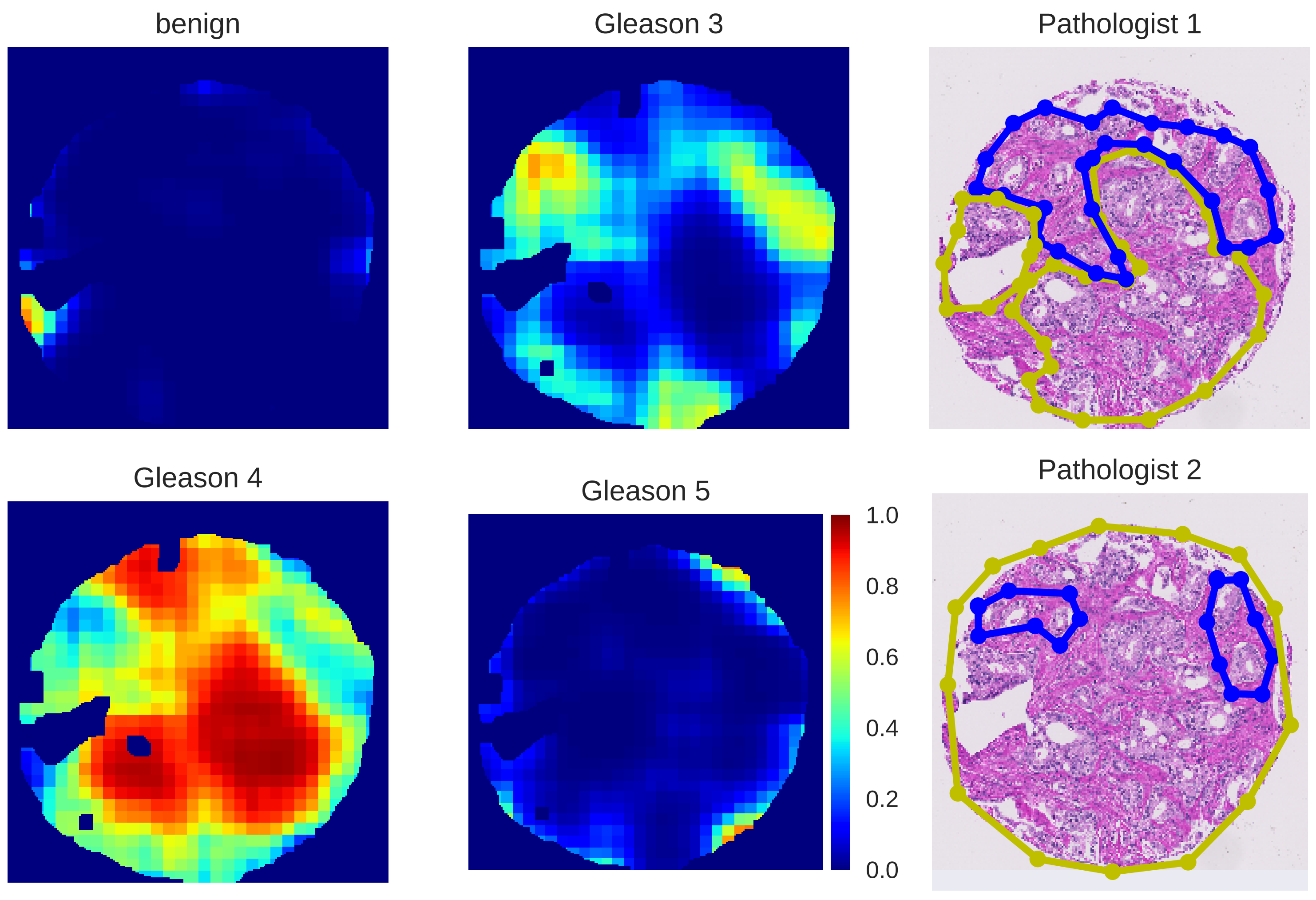

benign
Gleason 3
Pathologist 1
Gleason 4
Gleason 5
Pathologist 2
1.0
0.8
0.6
0.4
0.2
0.0

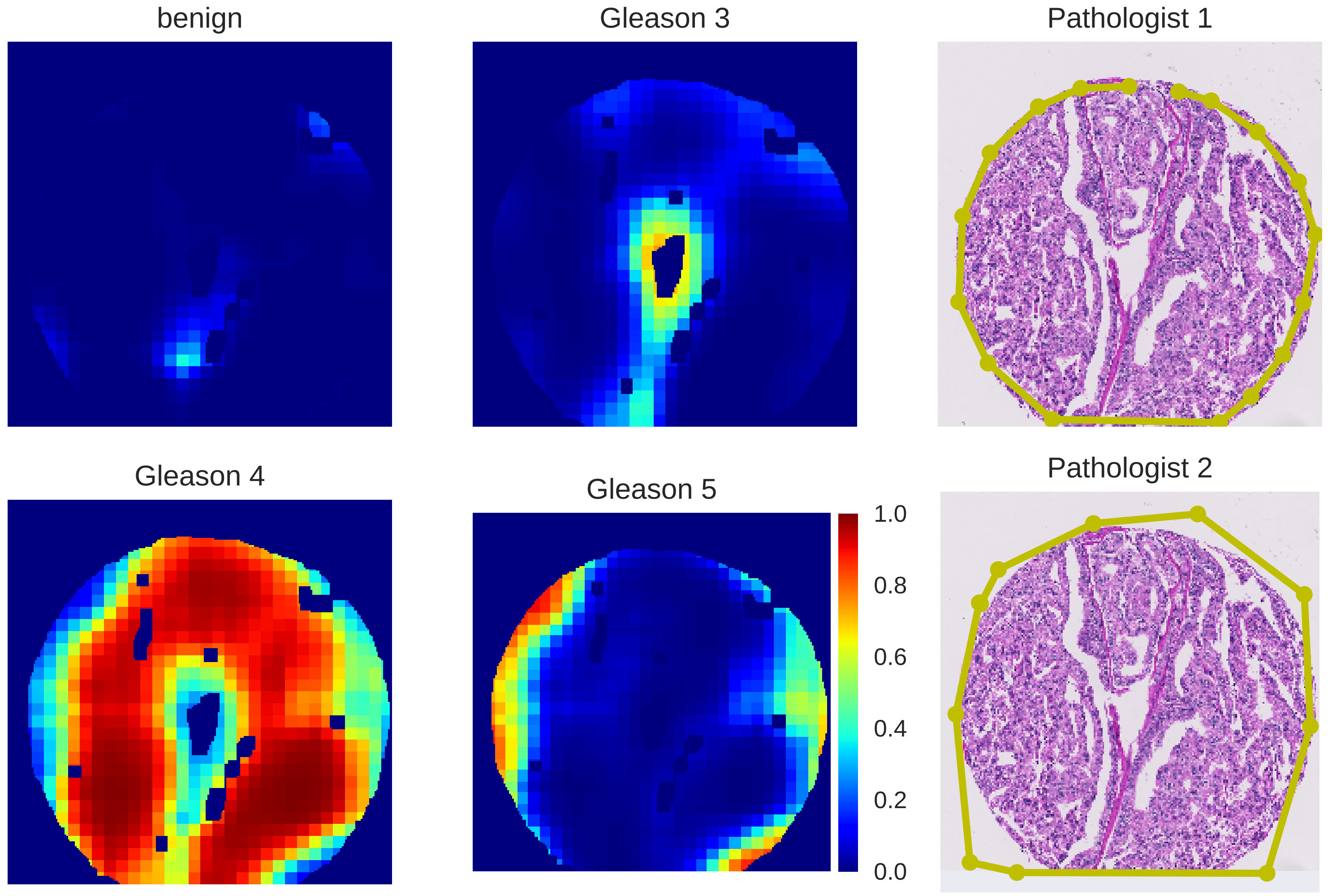
benign
Gleason 3
Pathologist 1
Gleason 4
Gleason 5
Pathologist 2
1.0
0.8
0.6
0.4
0.2
0.0

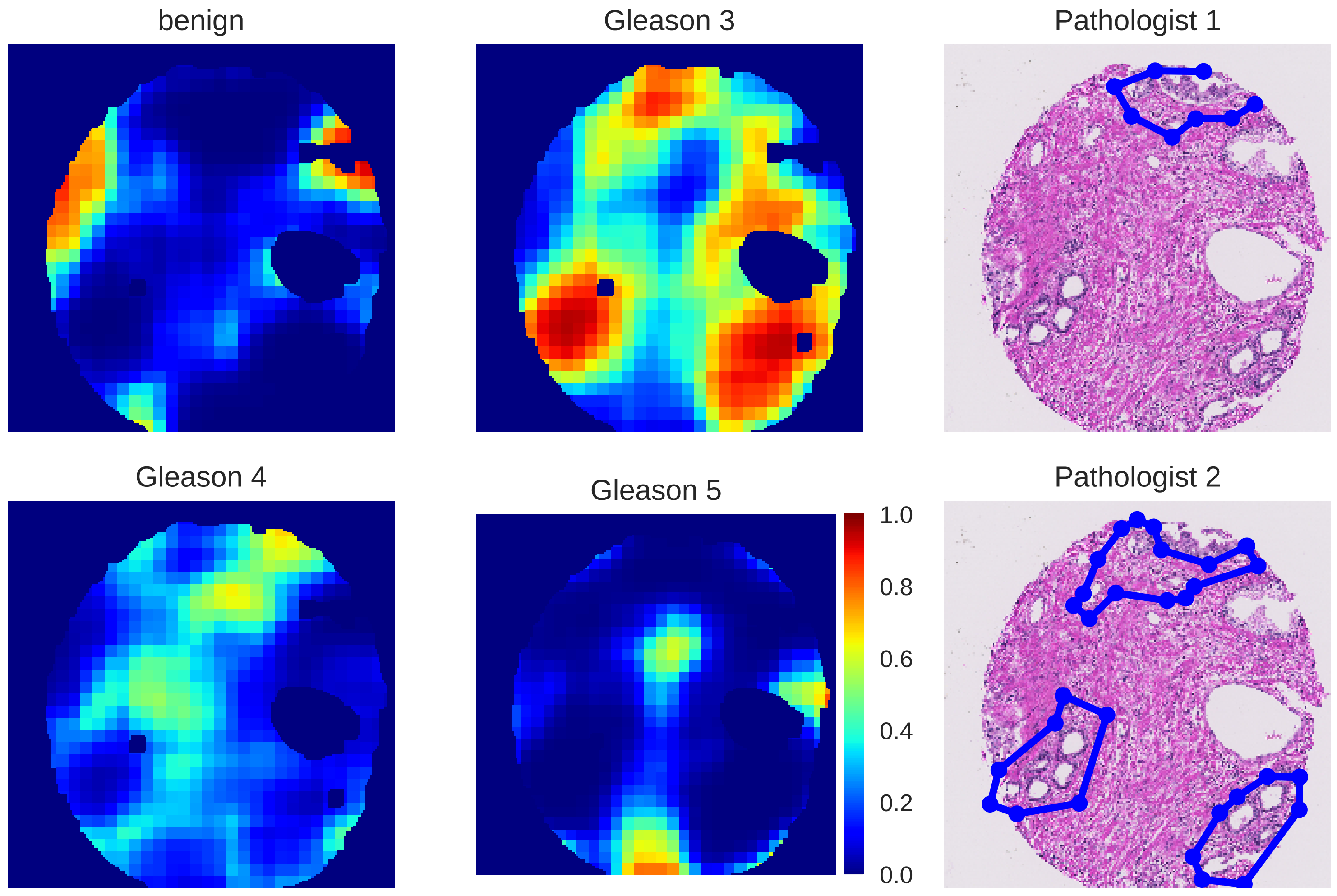

benign
Gleason 3
Pathologist 1
Gleason 4
Gleason 5
Pathologist 2
1.0
0.8
0.6
0.4
0.2
0.0

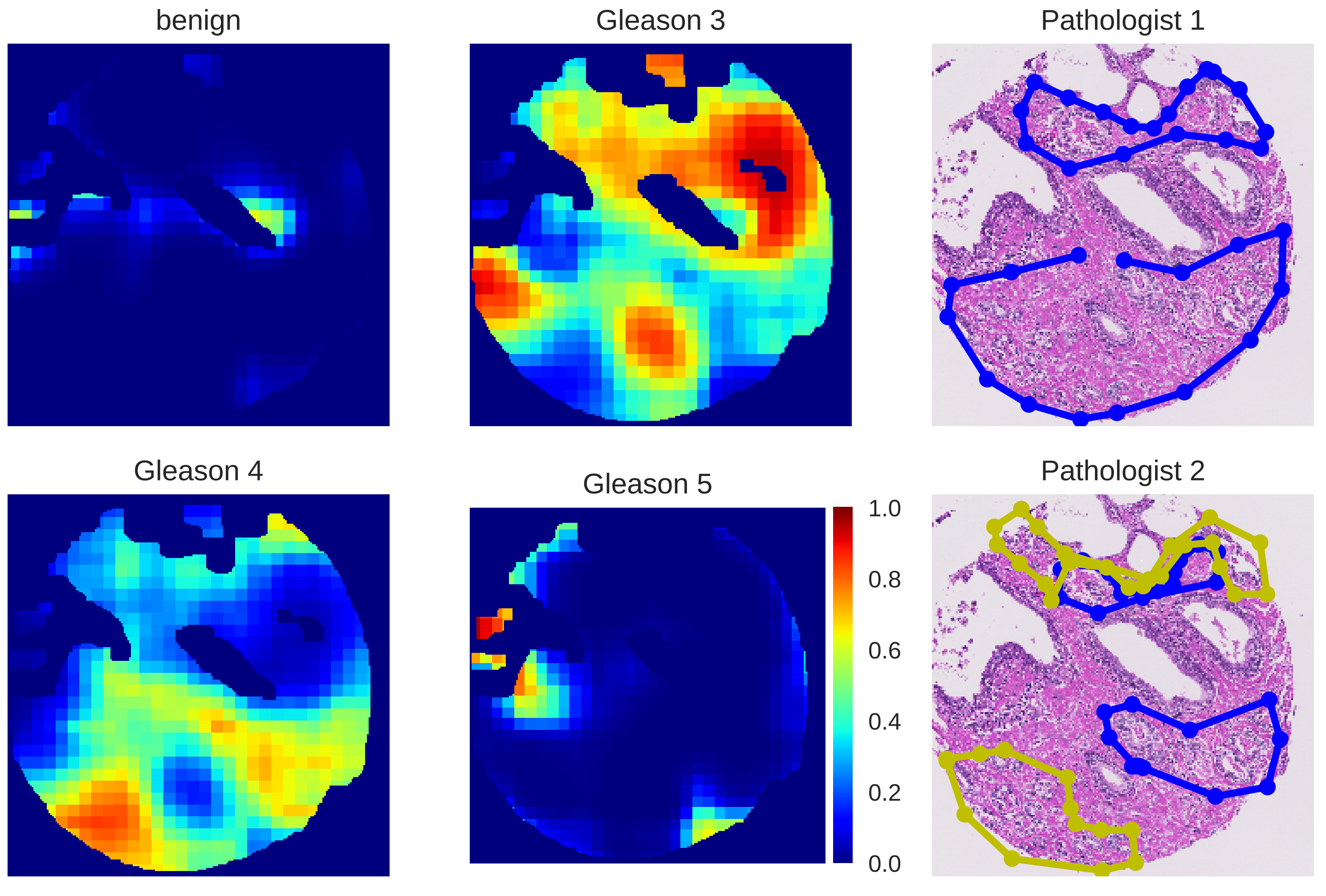
benign
Gleason 3
Pathologist 1
Gleason 4
Gleason 5
Pathologist 2
1.0
0.8
0.6
0.4
0.2
0.0

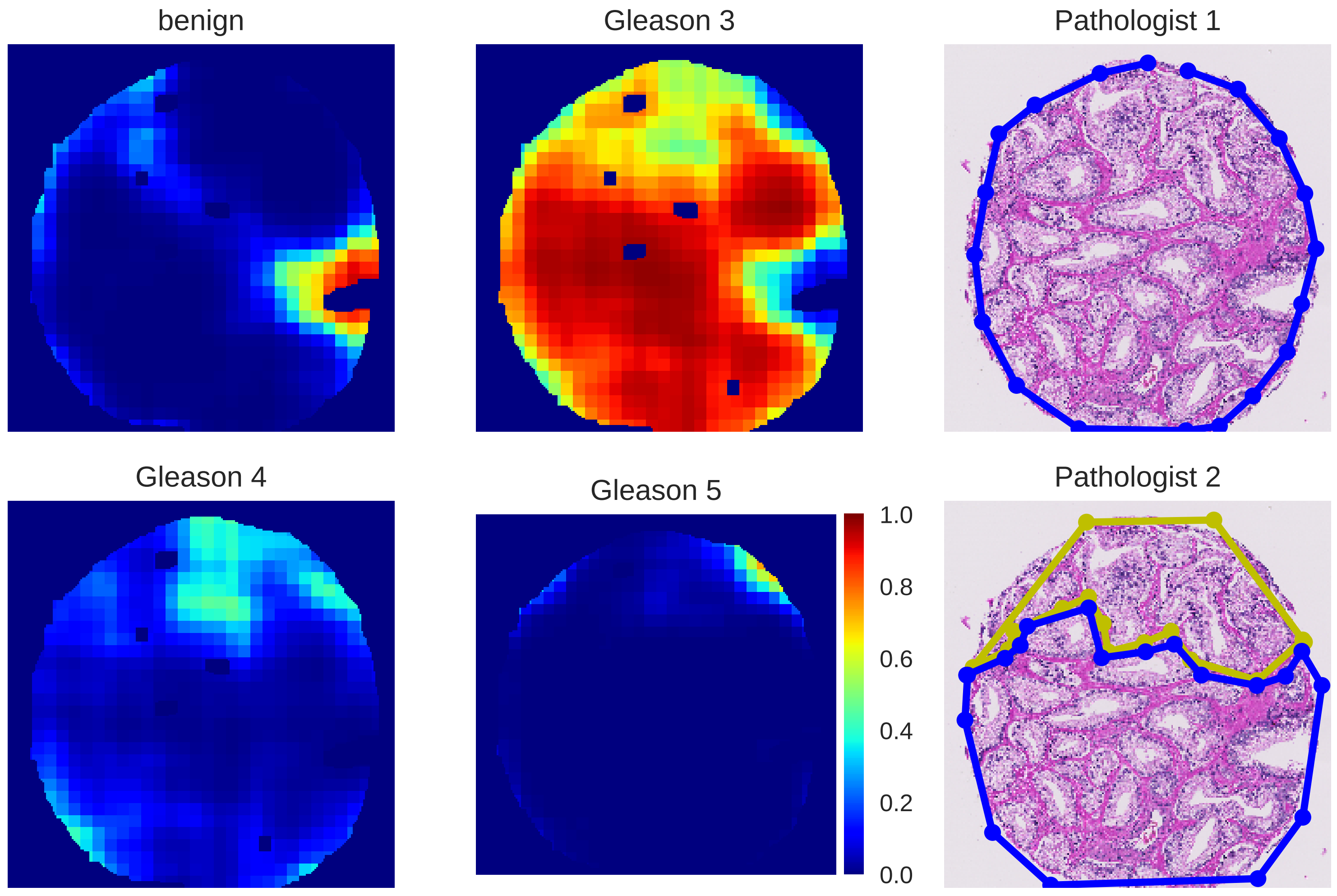
benign
Gleason 3
Pathologist 1
Gleason 4
Gleason 5
Pathologist 2
1.0
0.8
0.6
0.4
0.2
0.0

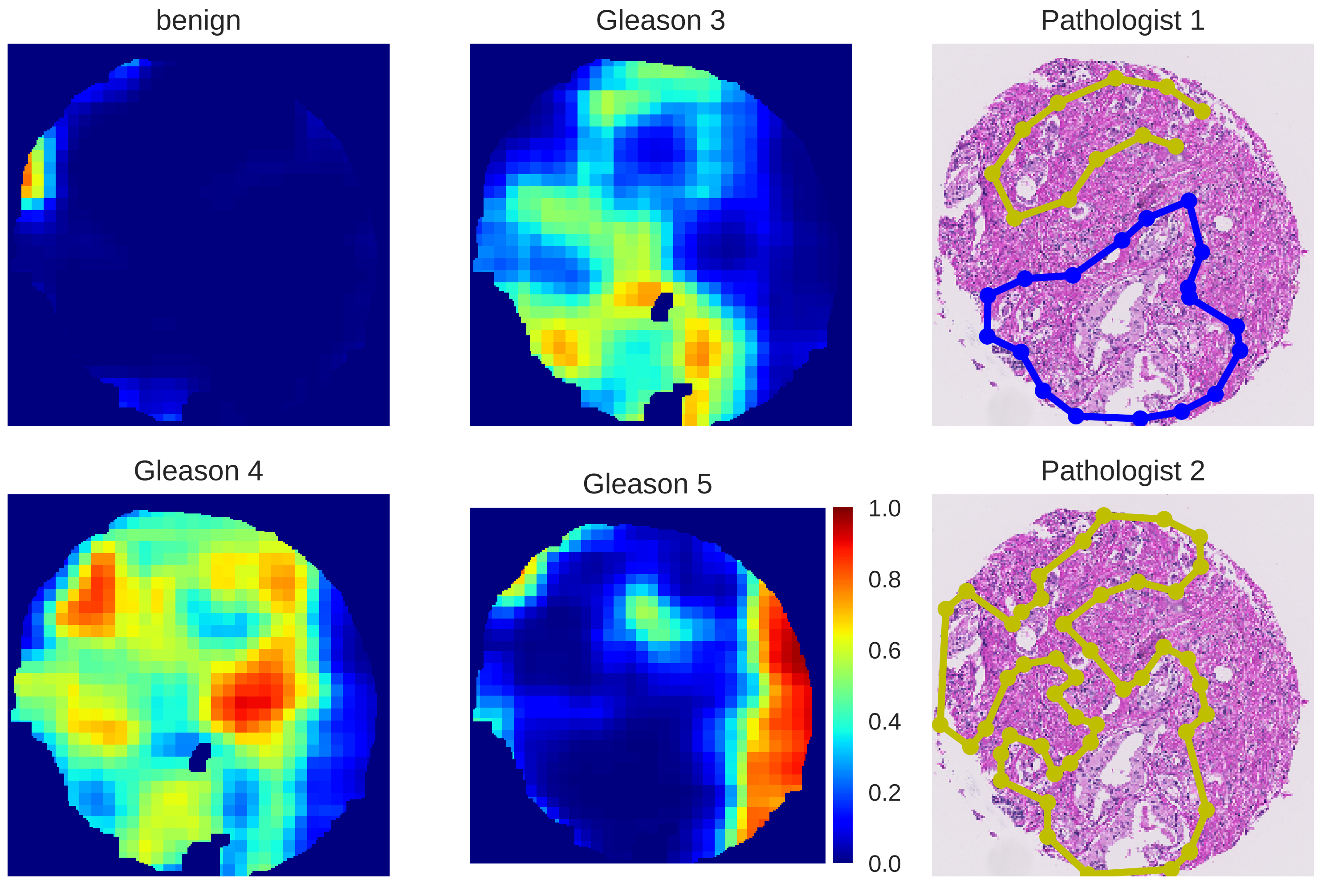
benign
Gleason 3
Pathologist 1
Gleason 4
Gleason 5
Pathologist 2
1.0
0.8
0.6
0.4
0.2
0.0

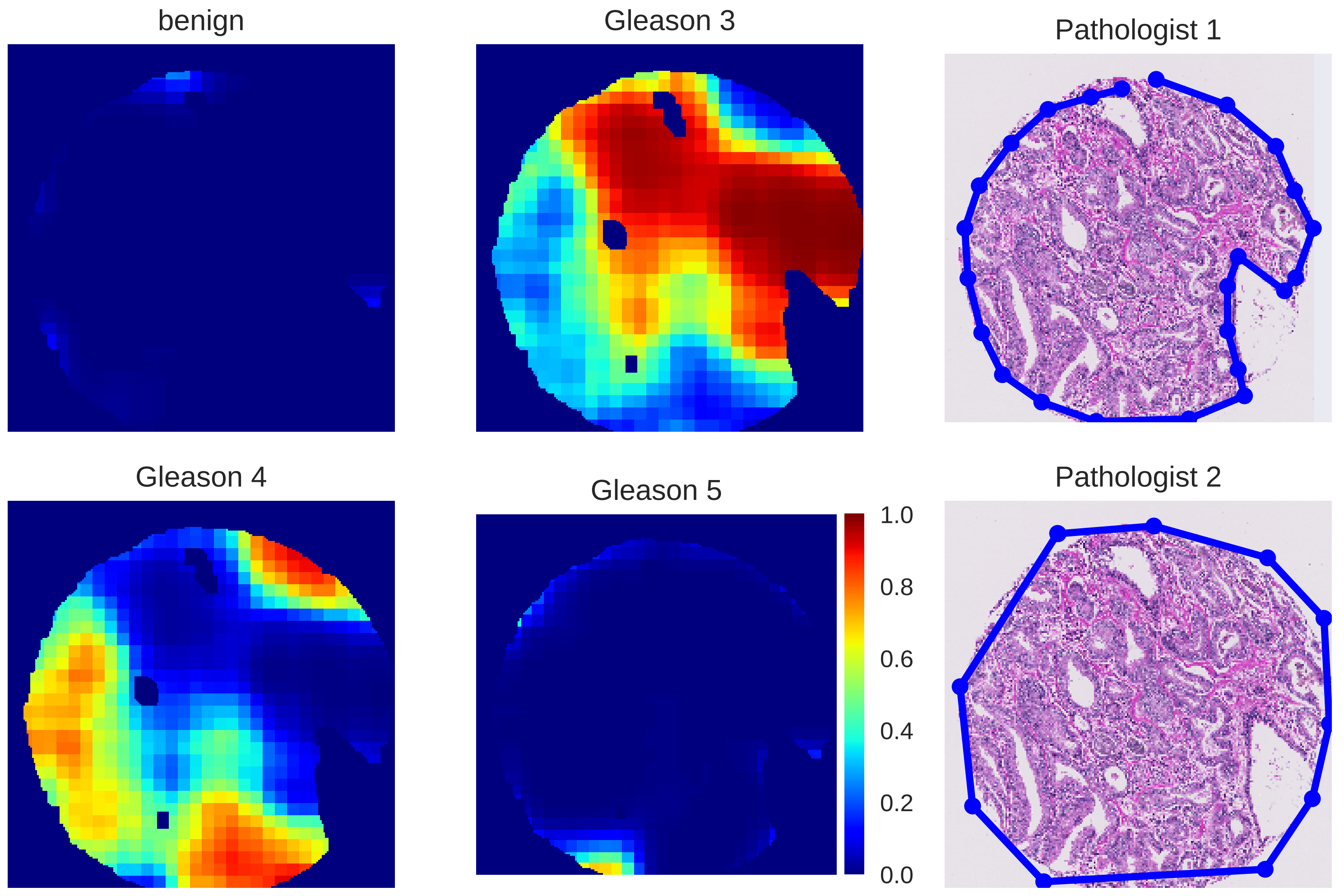
benign
Gleason 3
Pathologist 1
Gleason 4
Gleason 5
Pathologist 2
1.0
0.8
0.6
0.4
0.2
0.0

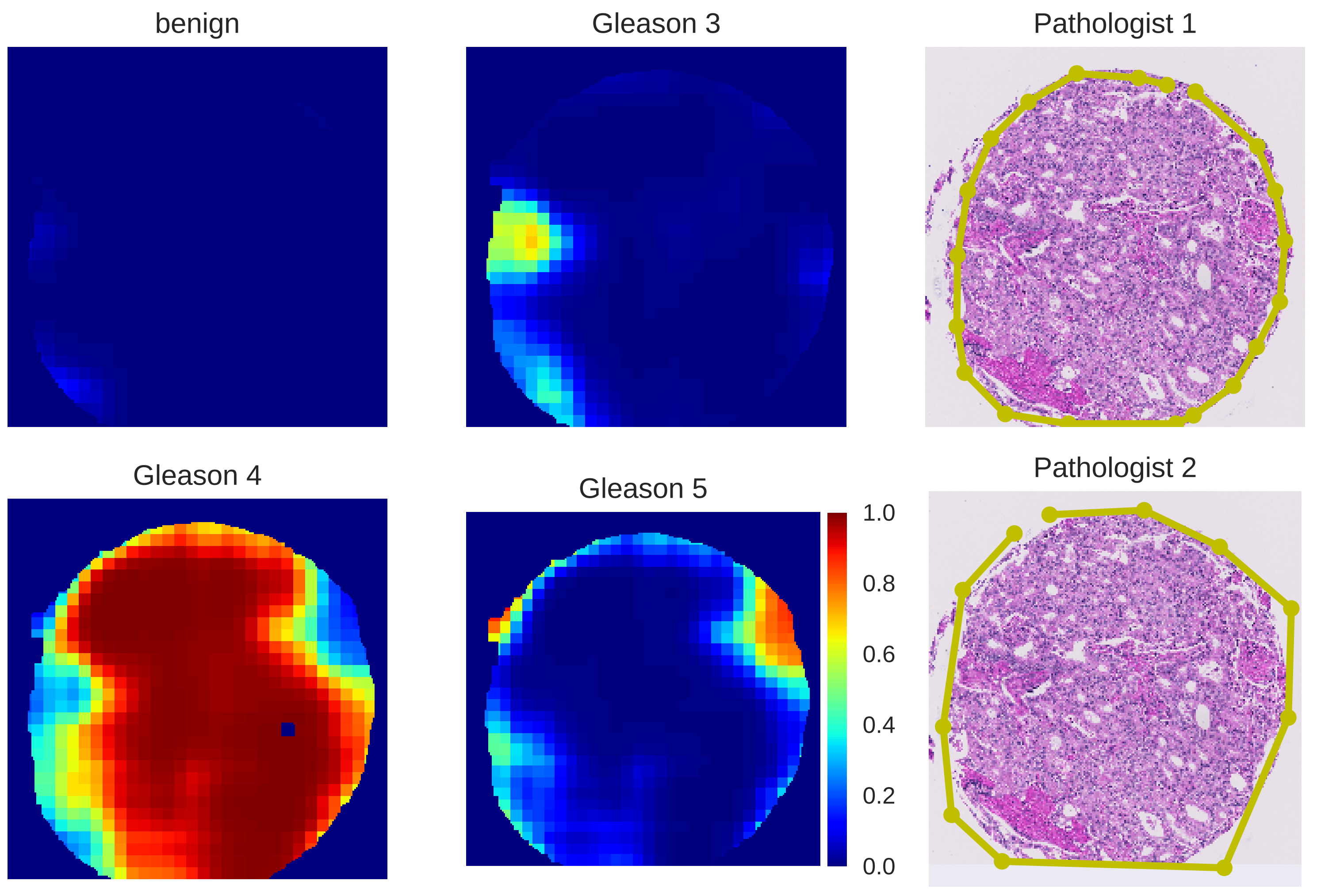

benign
Gleason 3
Pathologist 1
Gleason 4
Gleason 5
Pathologist 2
1.0
0.8
0.6
0.4
0.2
0.0

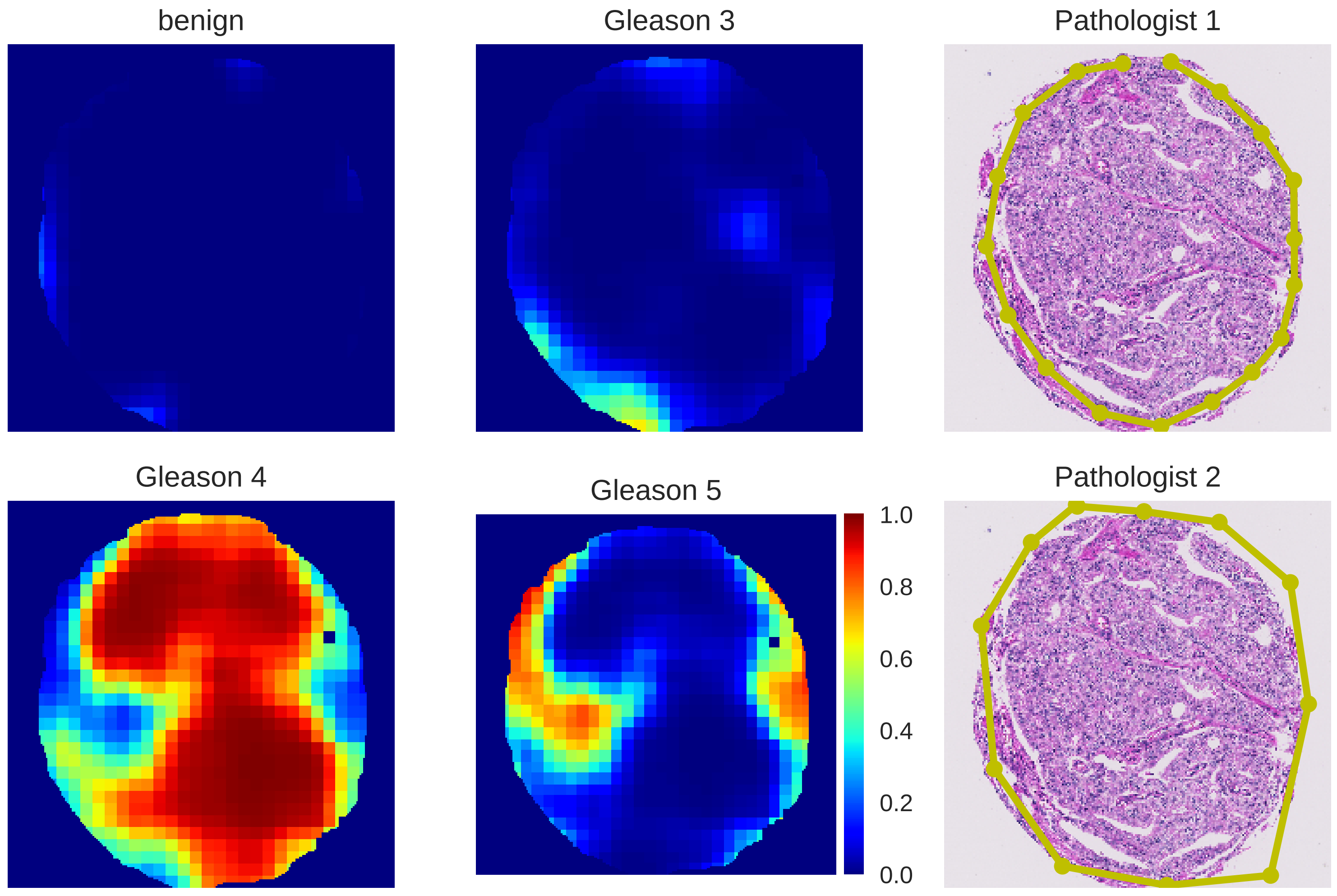

benign
Gleason 3
Pathologist 1
Gleason 4
Gleason 5
Pathologist 2
1.0
0.8
0.6
0.4
0.2
0.0

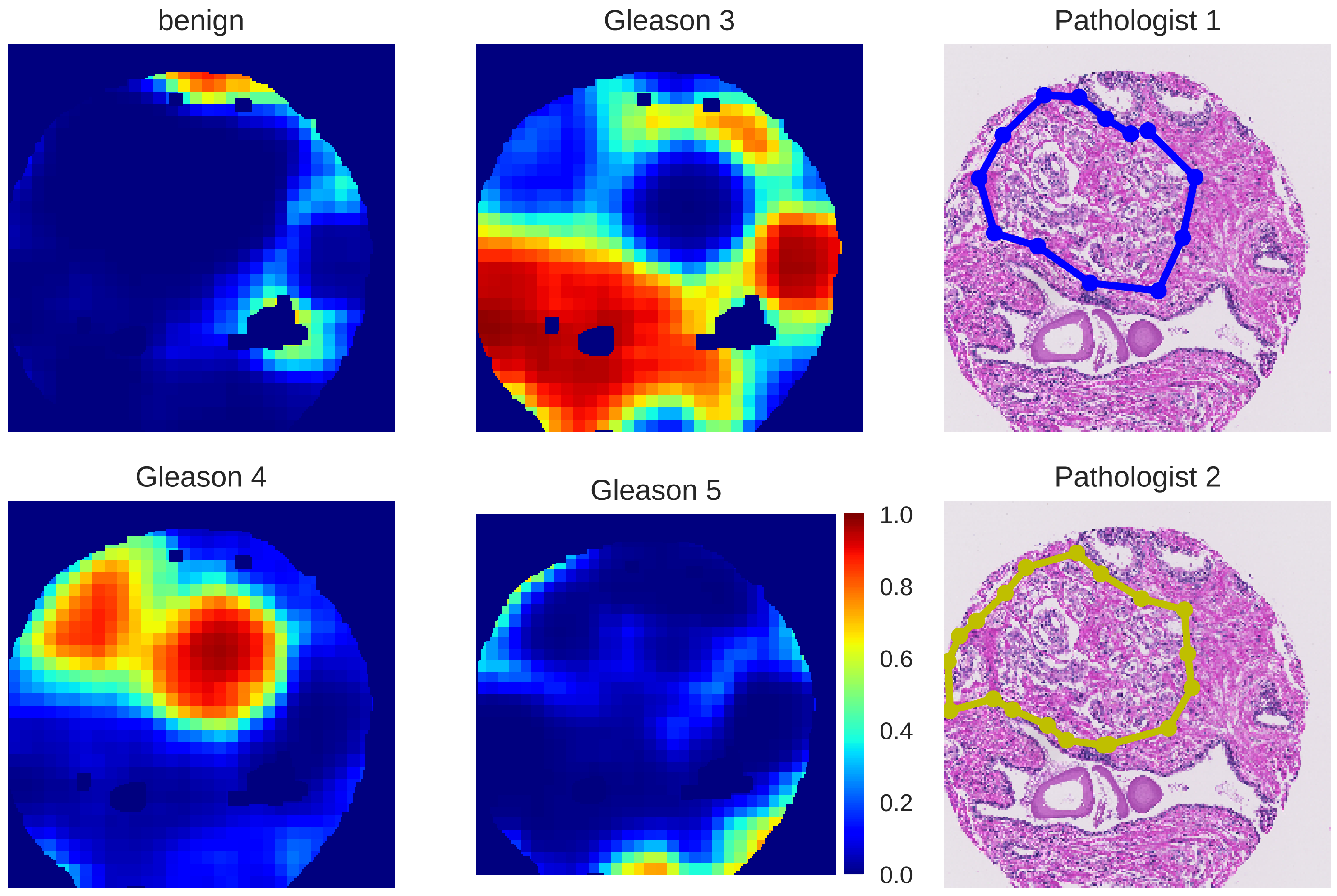
benign
Gleason 3
Pathologist 1
Gleason 4
Gleason 5
Pathologist 2
1.0
0.8
0.6
0.4
0.2
0.0

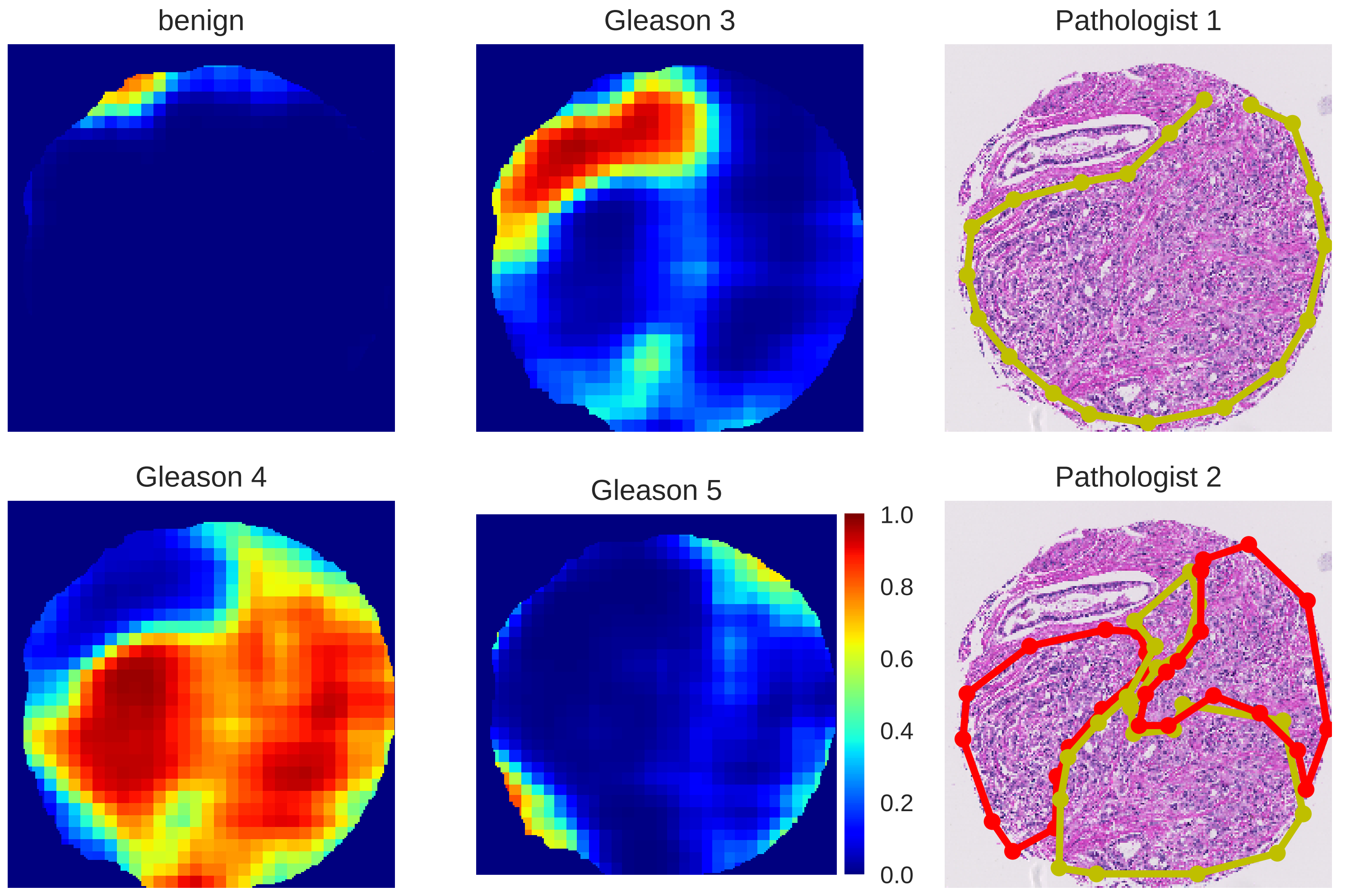

benign
Gleason 3
Pathologist 1
Gleason 4
Gleason 5
Pathologist 2
1.0
0.8
0.6
0.4
0.2
0.0

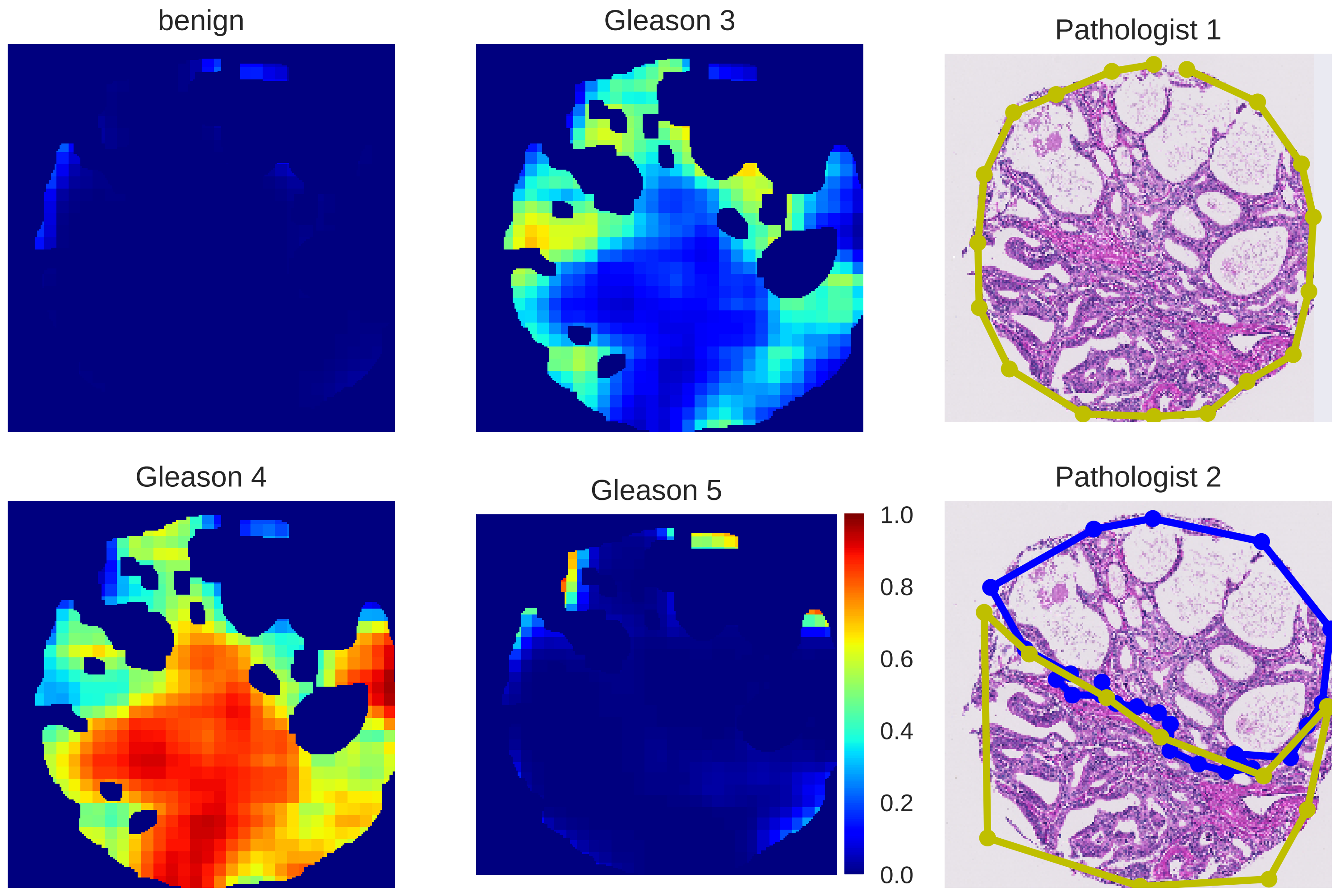
benign
Gleason 3
Pathologist 1
Gleason 4
Gleason 5
Pathologist 2
1.0
0.8
0.6
0.4
0.2
0.0

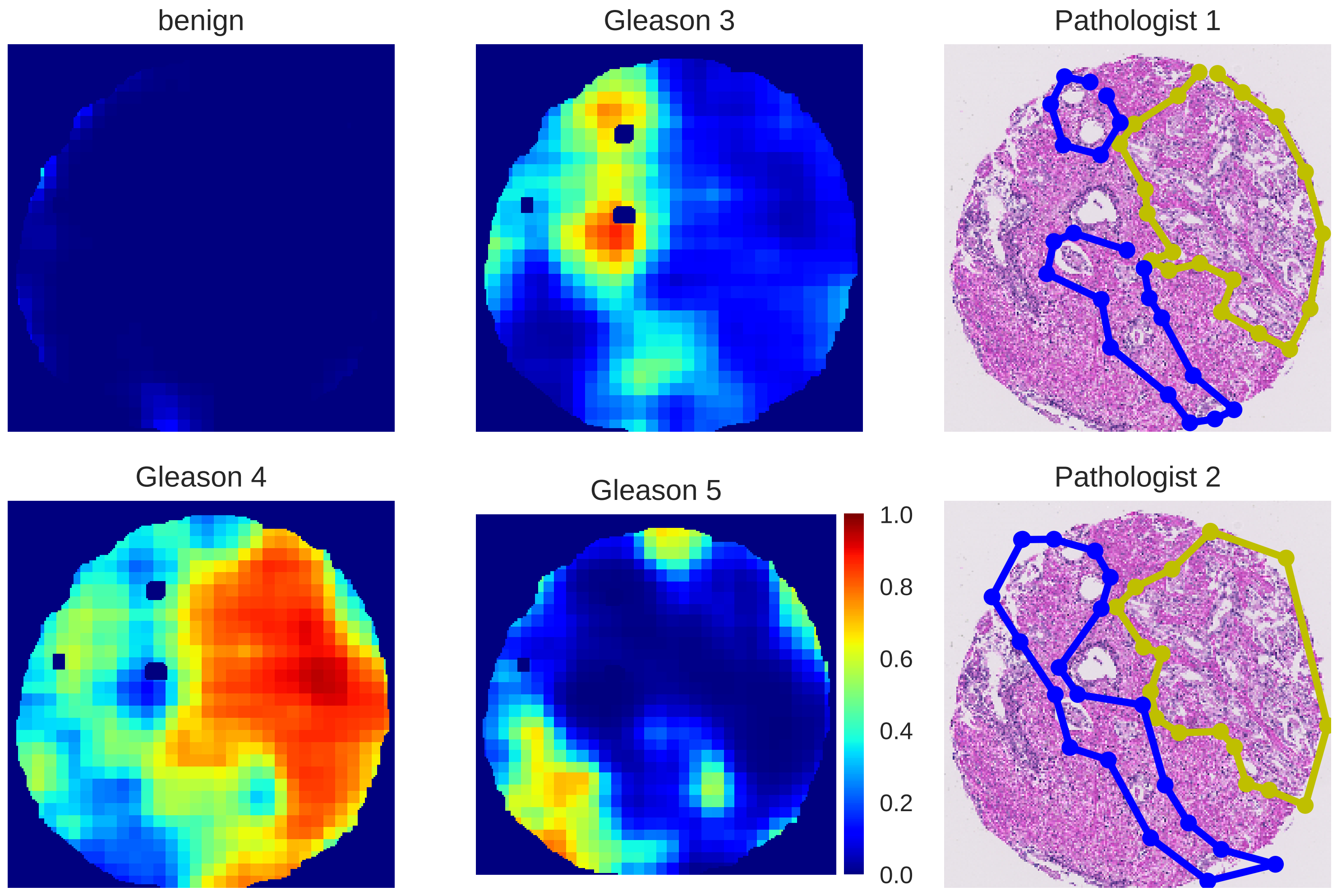
benign
Gleason 3
Pathologist 1
Gleason 4
Gleason 5
Pathologist 2
1.0
0.8
0.6
0.4
0.2
0.0

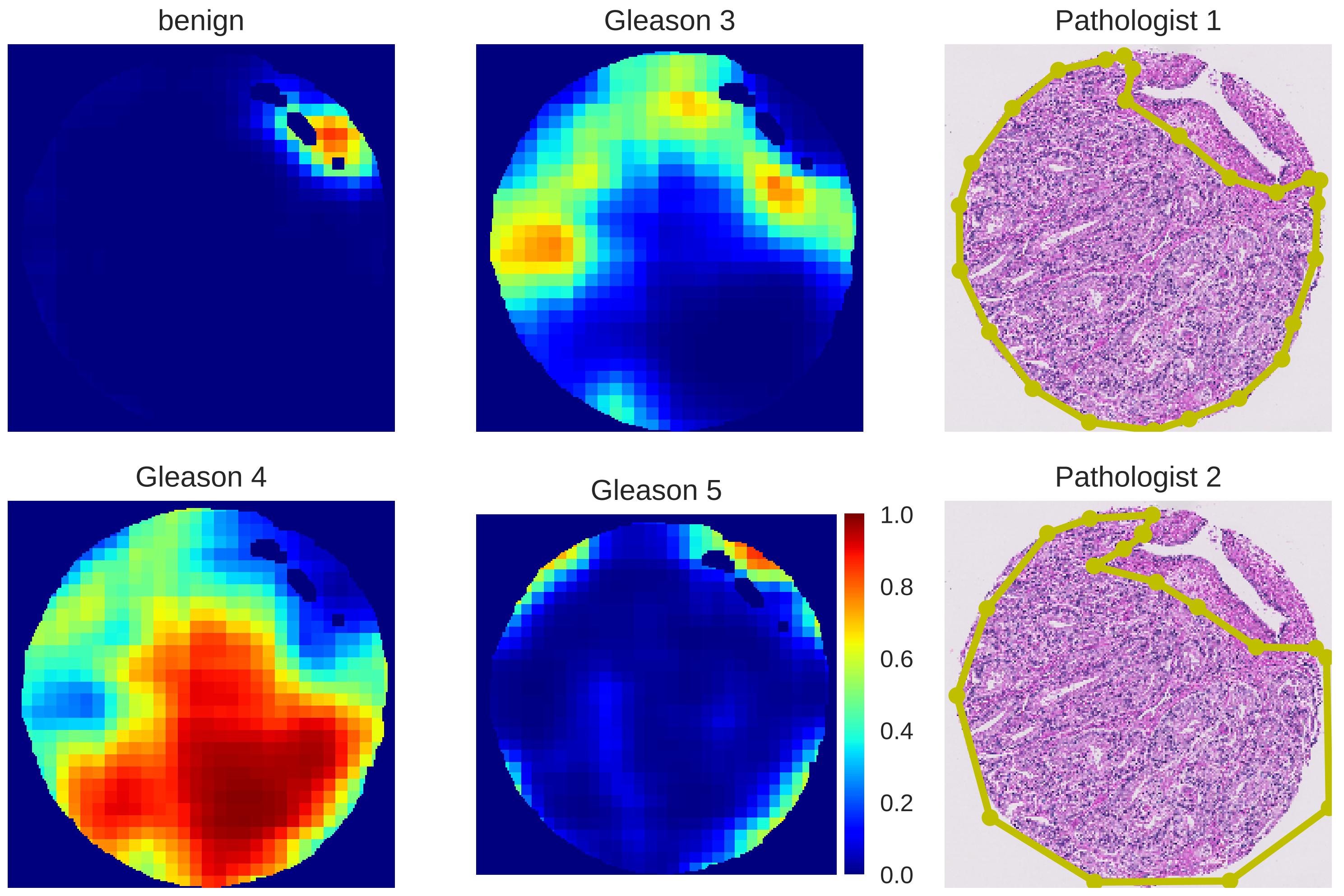
benign
Gleason 3
Pathologist 1
Gleason 4
Gleason 5
Pathologist 2
1.0
0.8
0.6
0.4
0.2
0.0

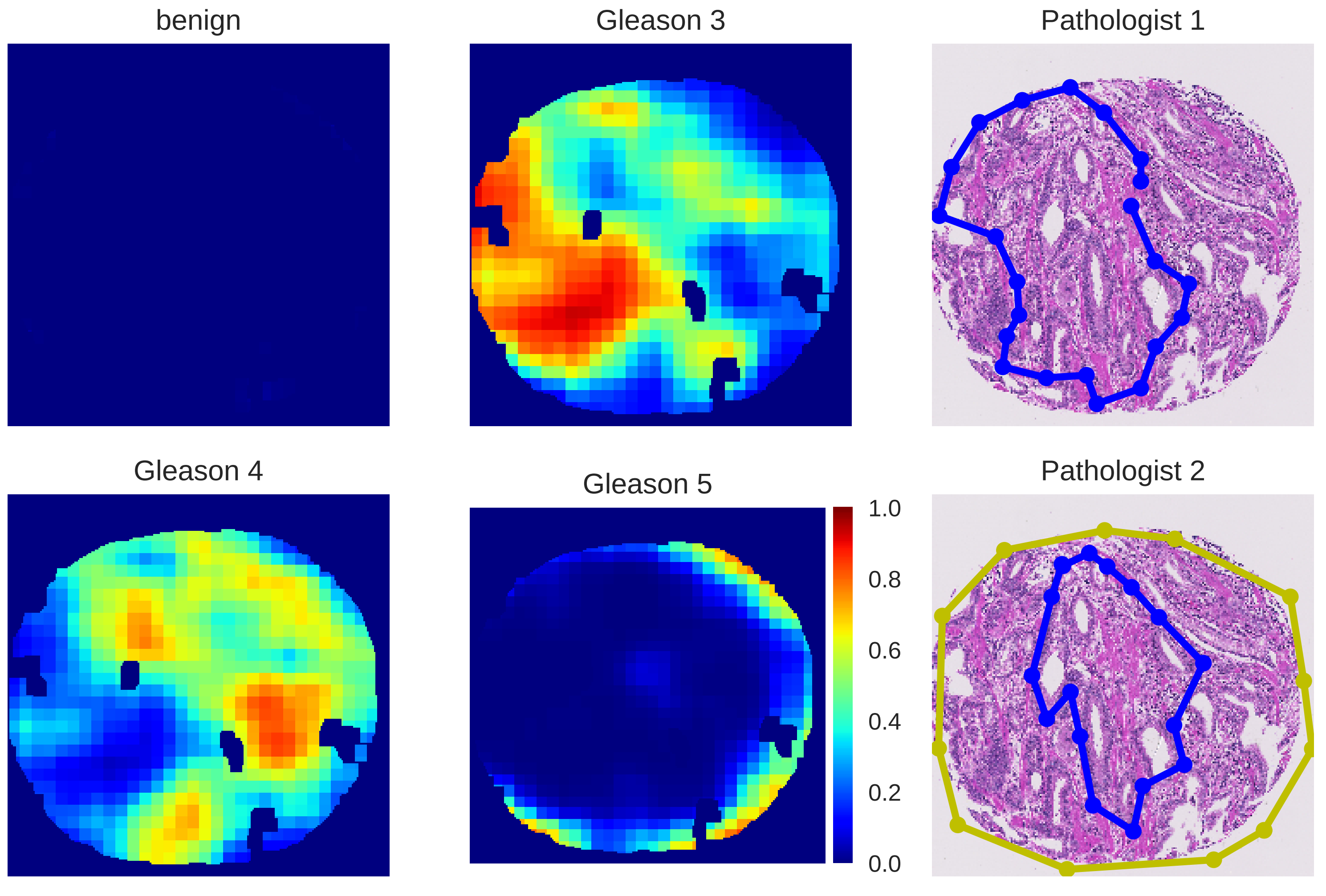
benign
Gleason 3
Pathologist 1
Gleason 4
Gleason 5
Pathologist 2
1.0
0.8
0.6
0.4
0.2
0.0

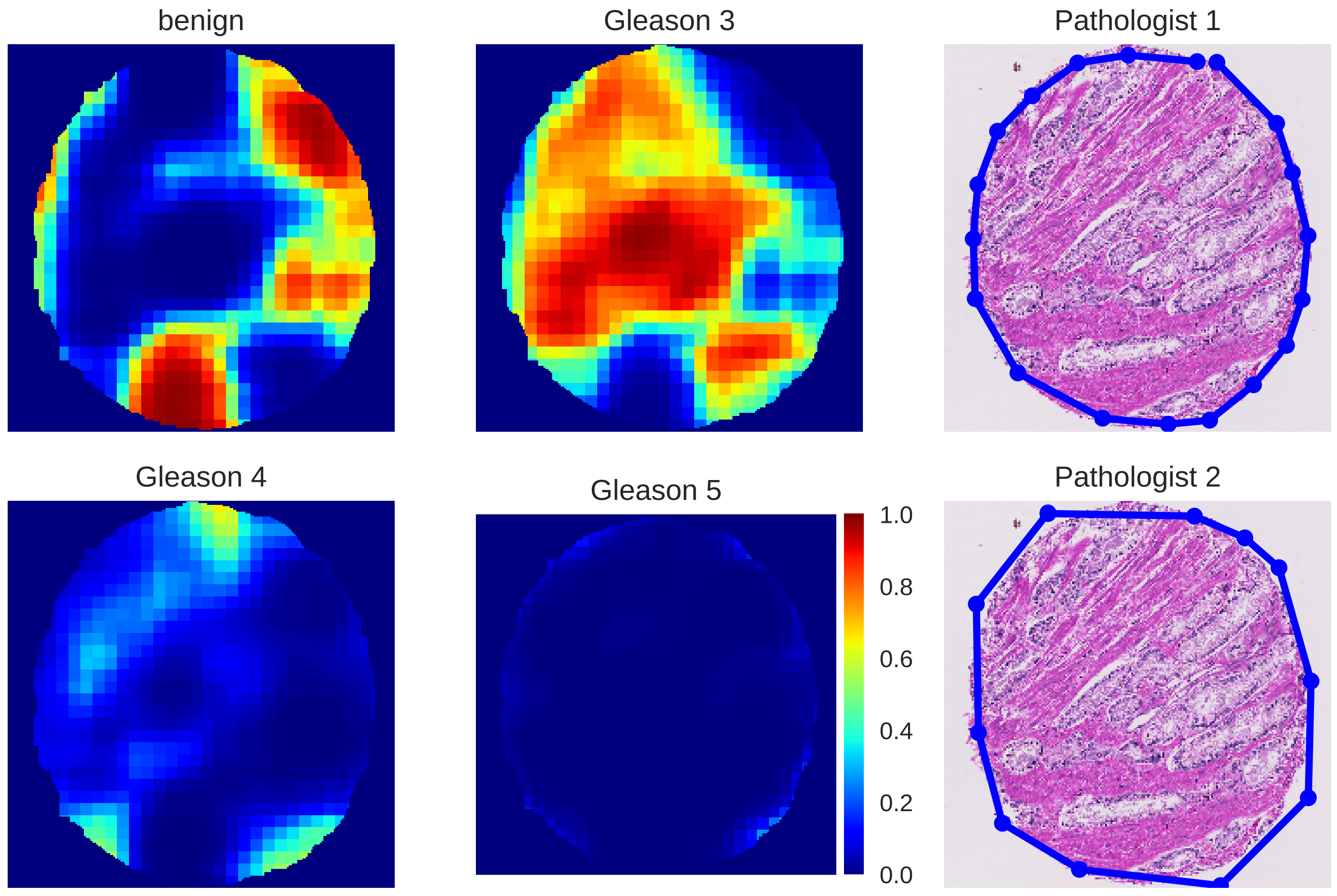

benign
Gleason 3
Pathologist 1
Gleason 4
Gleason 5
Pathologist 2
1.0
0.8
0.6
0.4
0.2
0.0

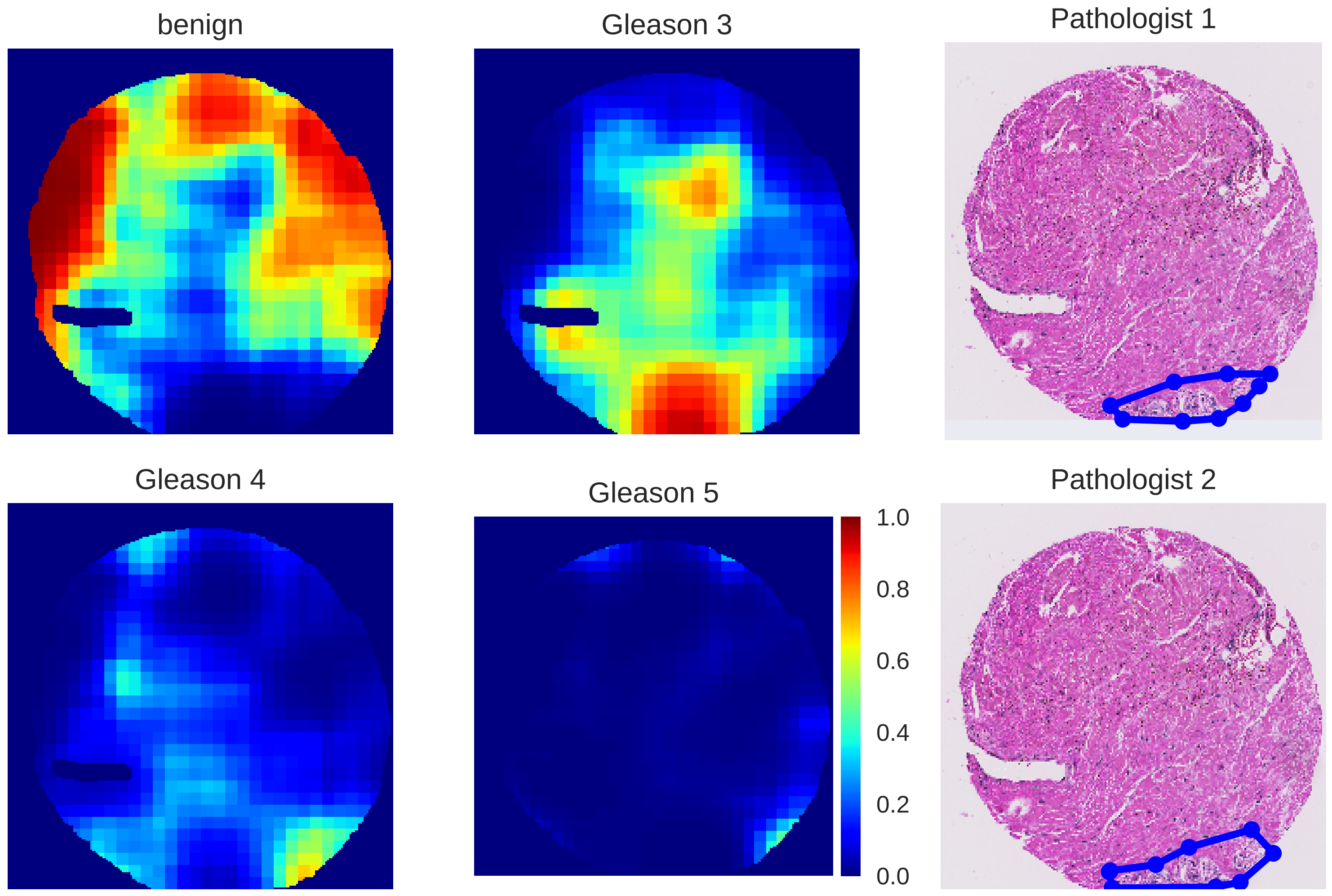

benign
Gleason 3
Pathologist 1
Gleason 4
Gleason 5
Pathologist 2
1.0
0.8
0.6
0.4
0.2
0.0

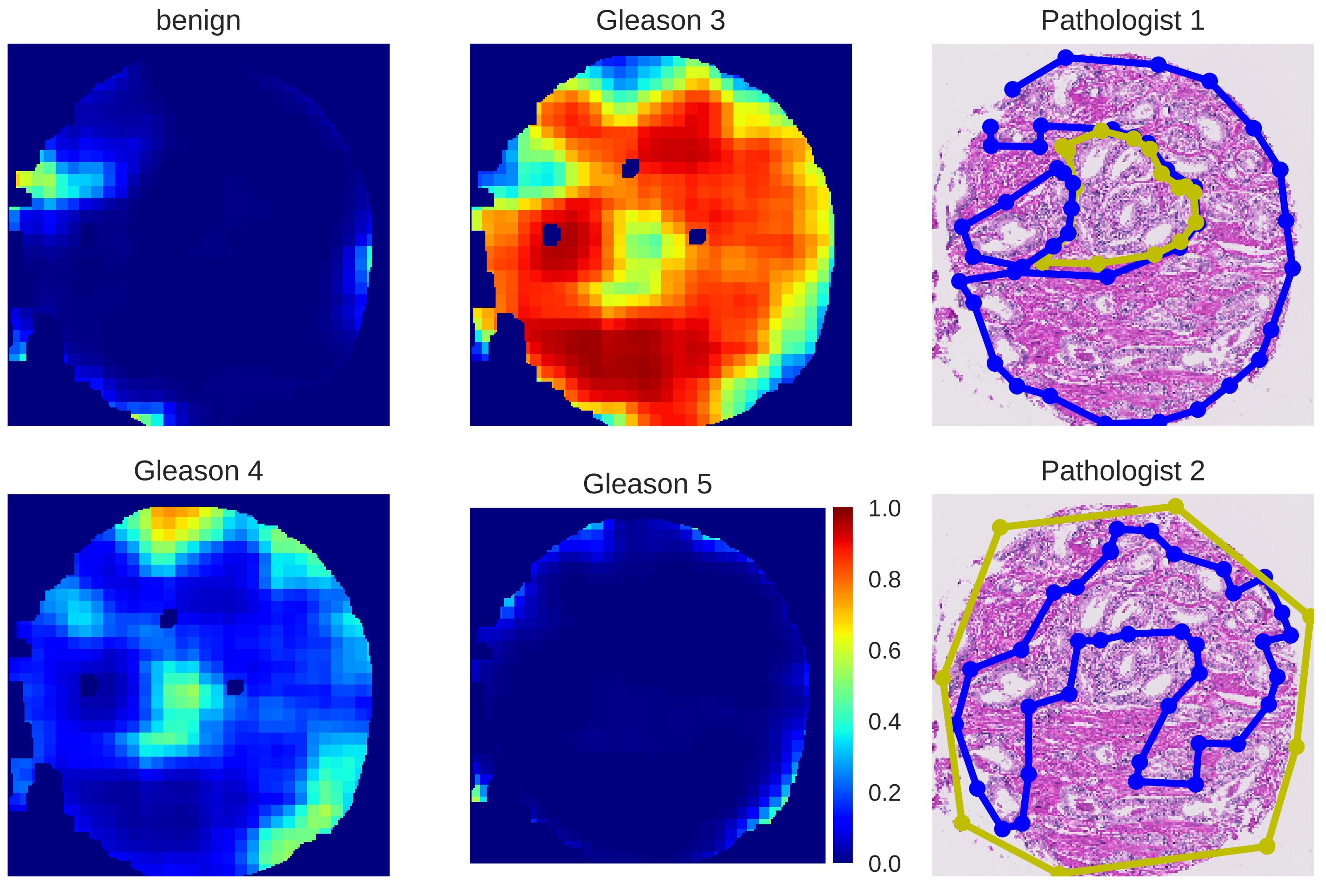

benign
Gleason 3
Pathologist 1
Gleason 4
Gleason 5
Pathologist 2
1.0
0.8
0.6
0.4
0.2
0.0

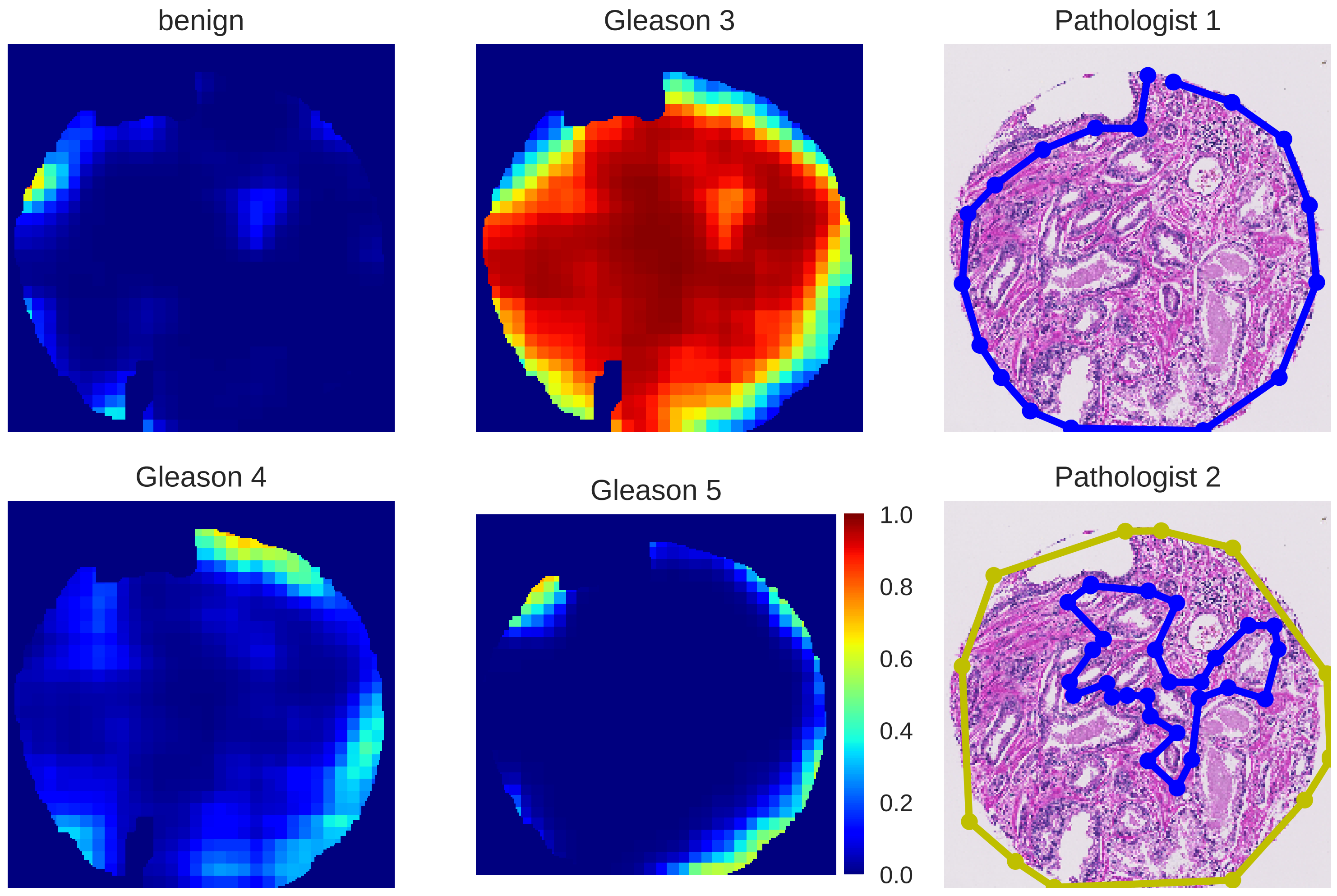

benign
Gleason 3
Pathologist 1
Gleason 4
Gleason 5
Pathologist 2
1.0
0.8
0.6
0.4
0.2
0.0

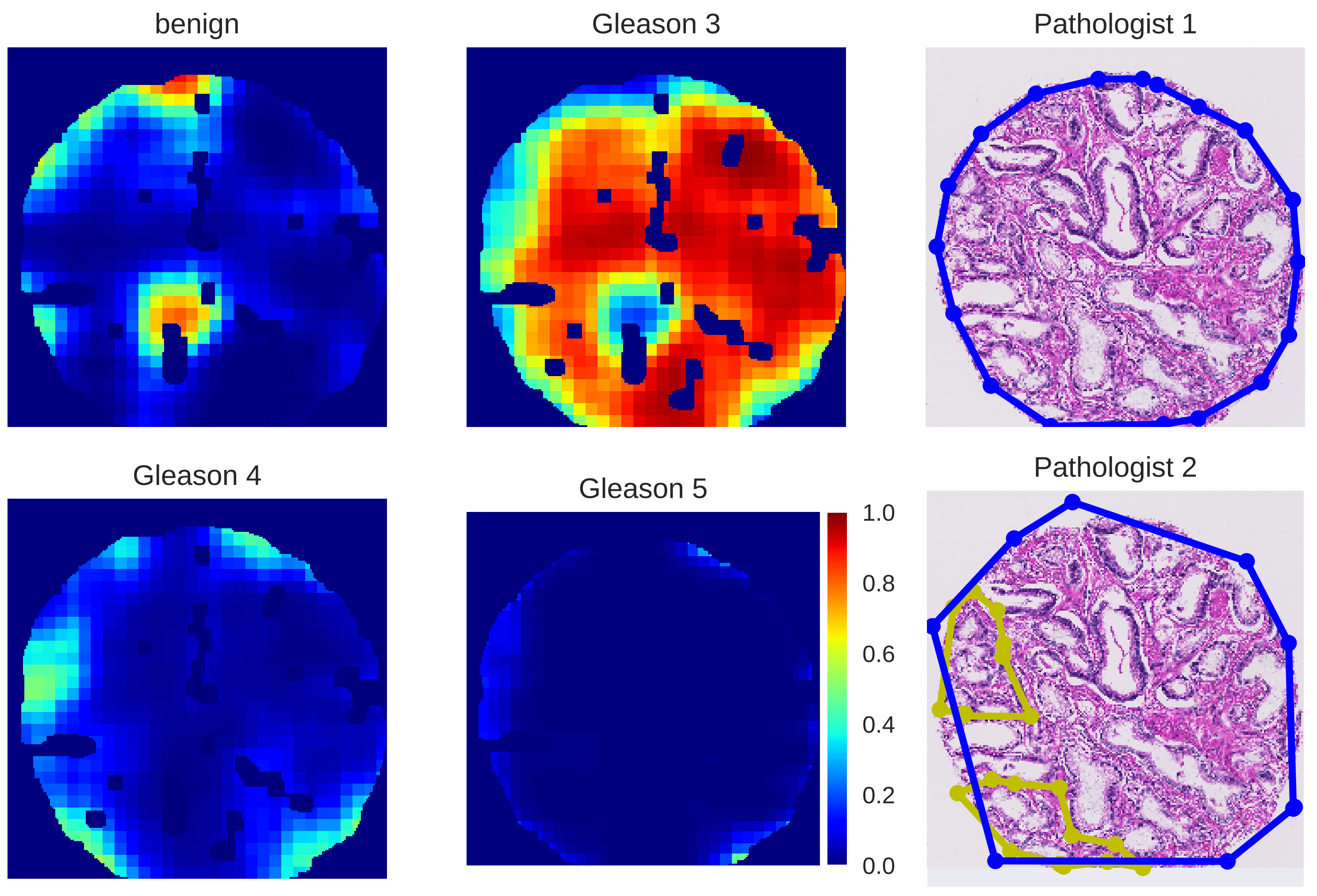
benign
Gleason 3
Pathologist 1
Gleason 4
Gleason 5
Pathologist 2
1.0
0.8
0.6
0.4
0.2
0.0

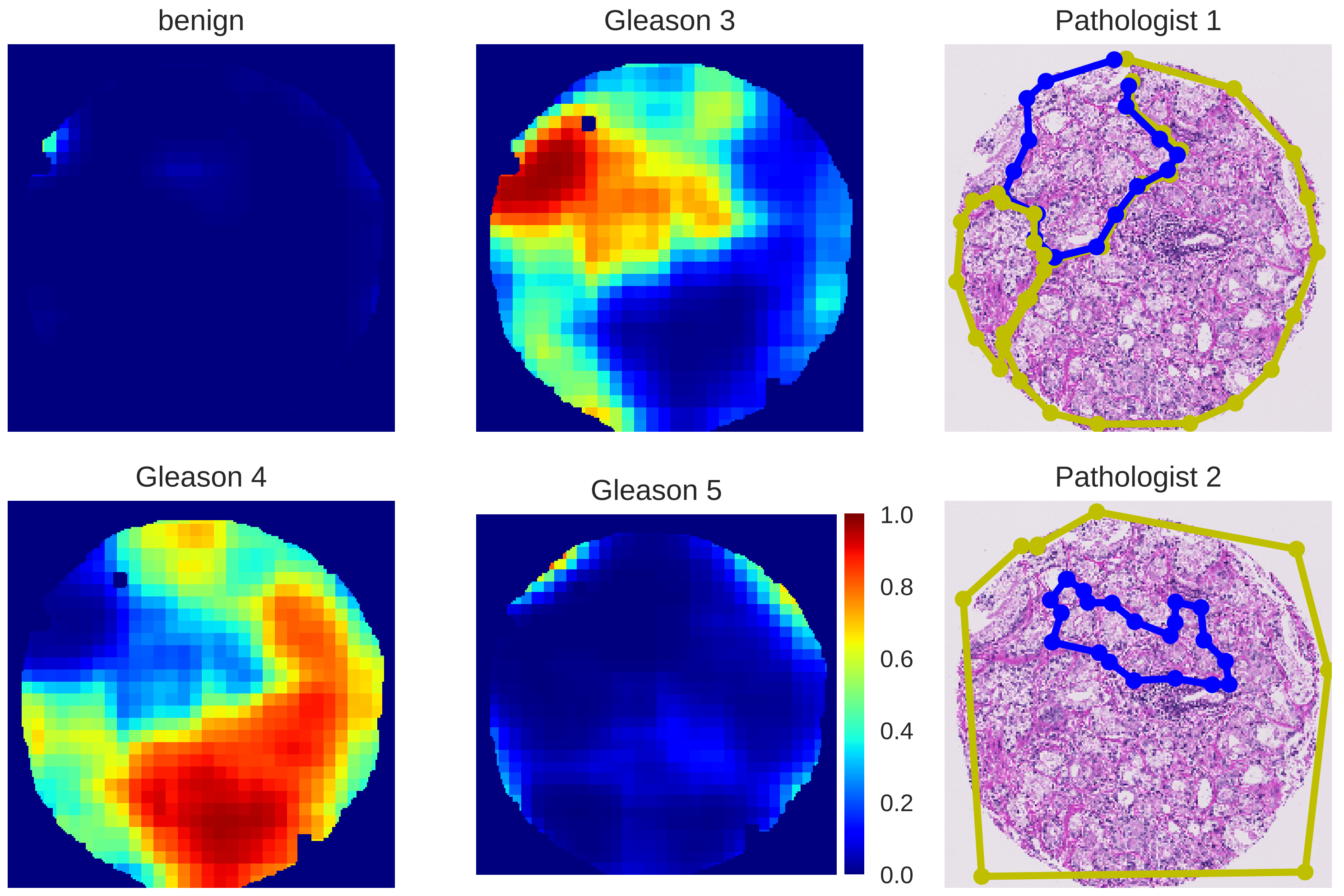

benign
Gleason 3
Pathologist 1
Gleason 4
Gleason 5
Pathologist 2
1.0
0.8
0.6
0.4
0.2
0.0

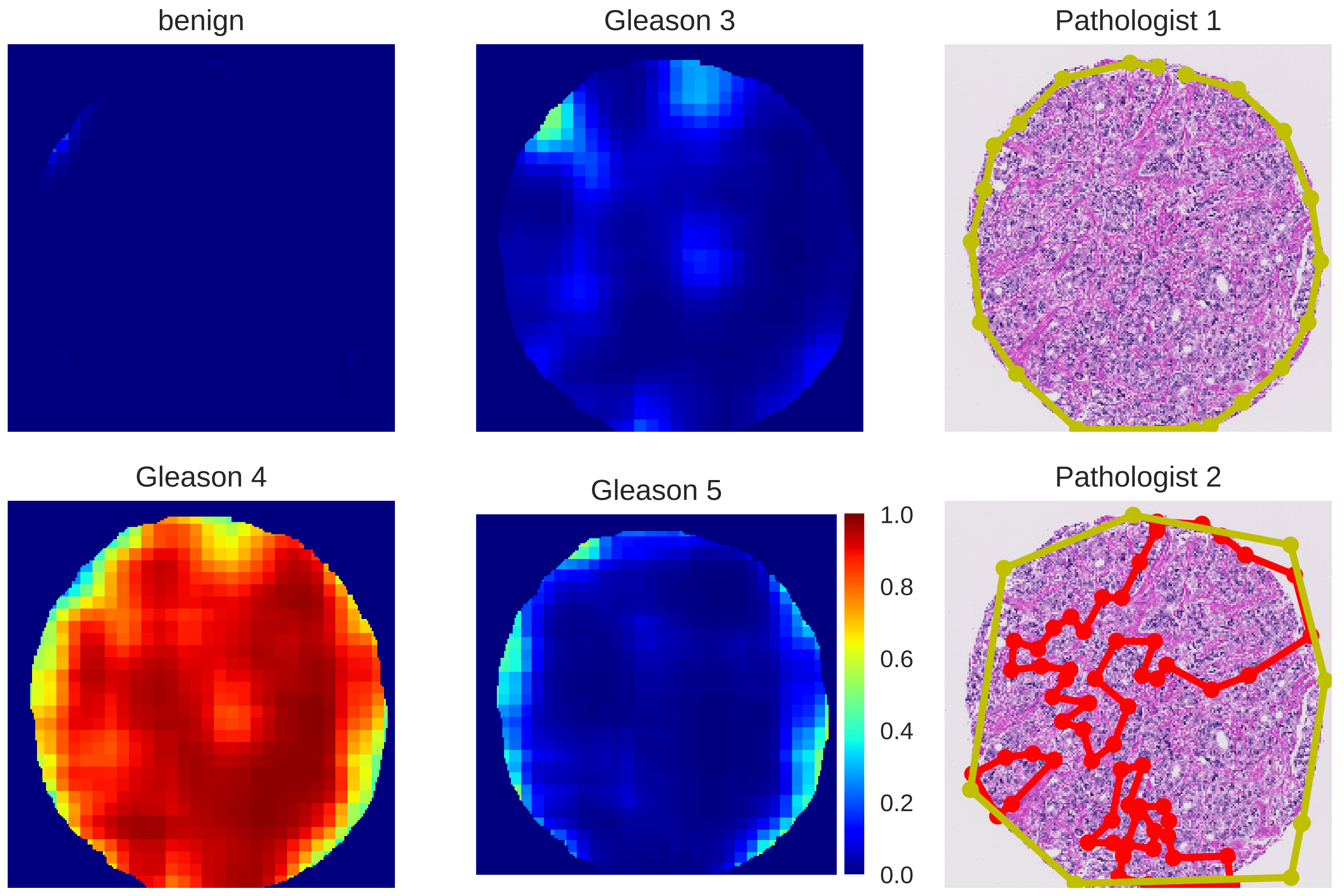

benign
Gleason 3
Pathologist 1
Gleason 4
Gleason 5
Pathologist 2
1.0
0.8
0.6
0.4
0.2
0.0

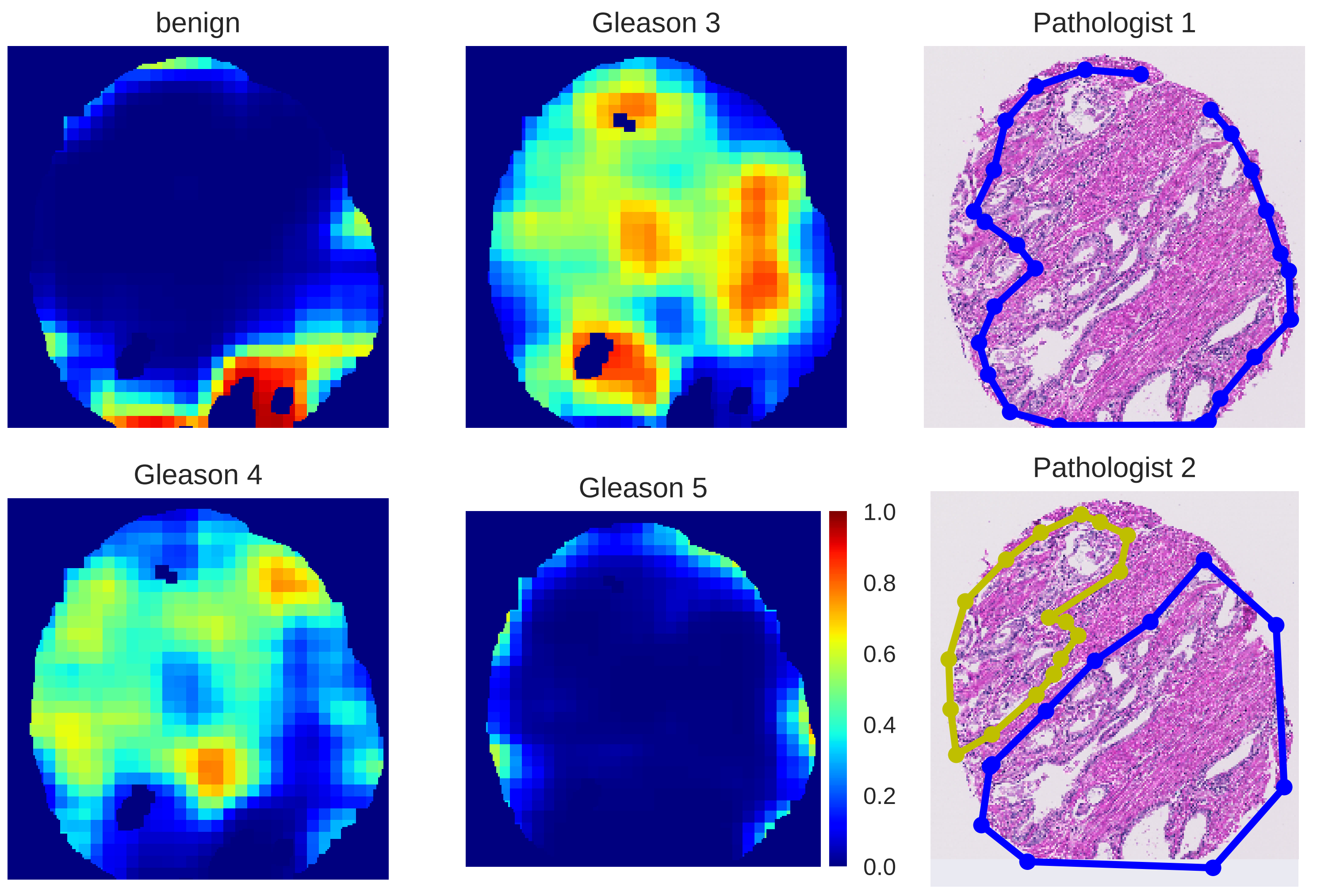

benign
Gleason 3
Pathologist 1
Gleason 4
Gleason 5
Pathologist 2
1.0
0.8
0.6
0.4
0.2
0.0

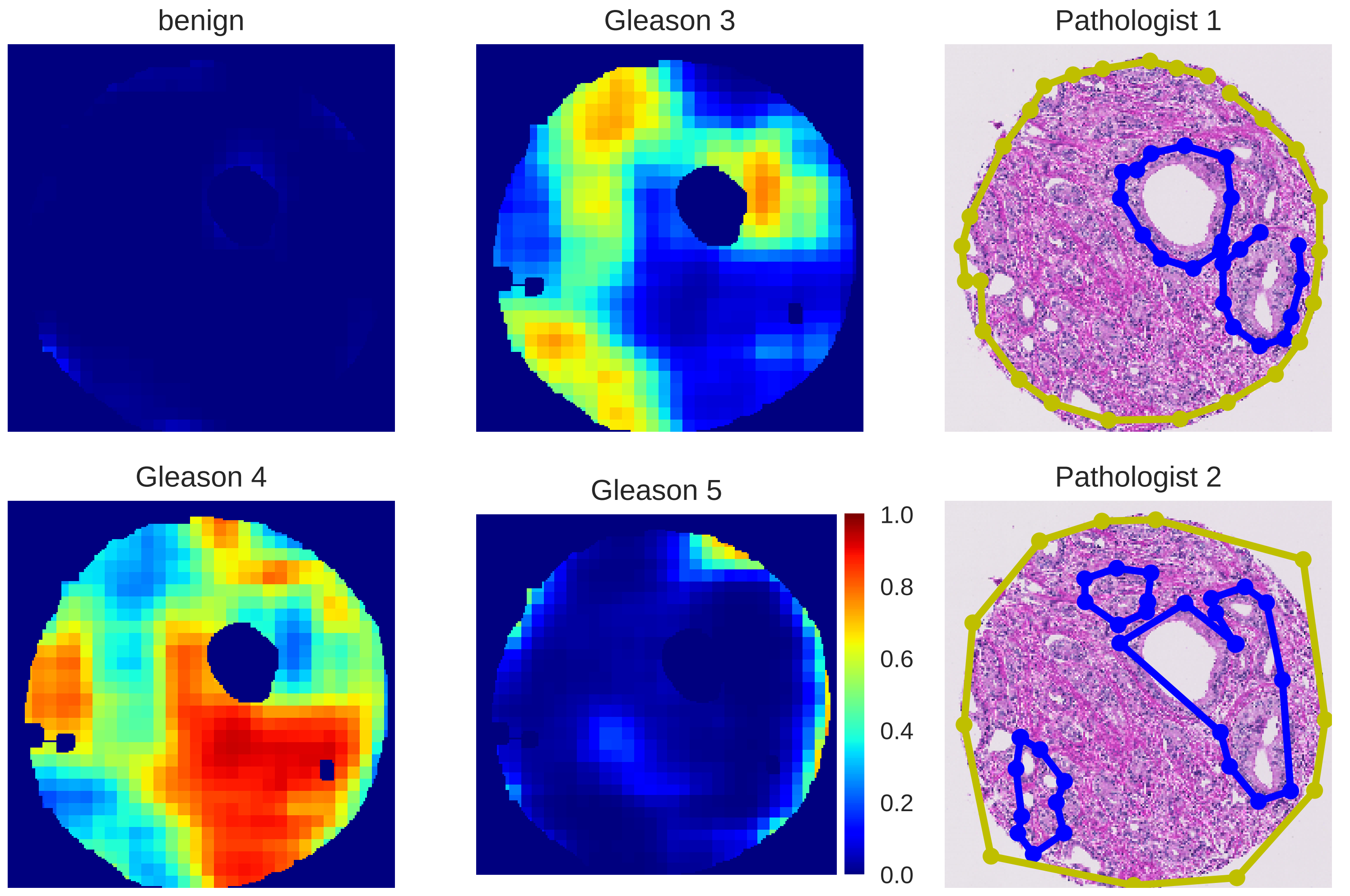

benign
Gleason 3
Pathologist 1
Gleason 4
Gleason 5
Pathologist 2
1.0
0.8
0.6
0.4
0.2
0.0

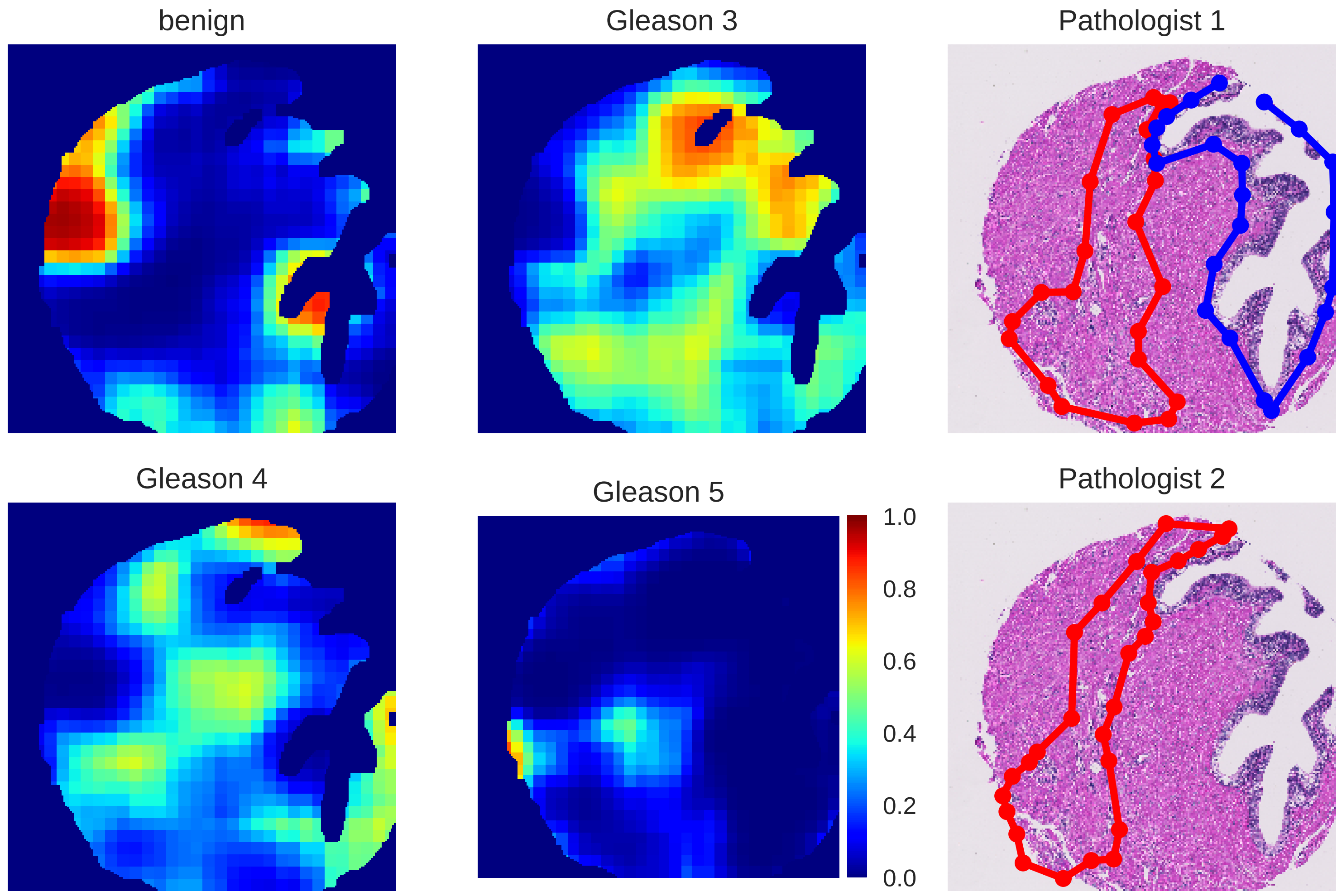

benign
Gleason 3
Pathologist 1
Gleason 4
Gleason 5
Pathologist 2
1.0
0.8
0.6
0.4
0.2
0.0

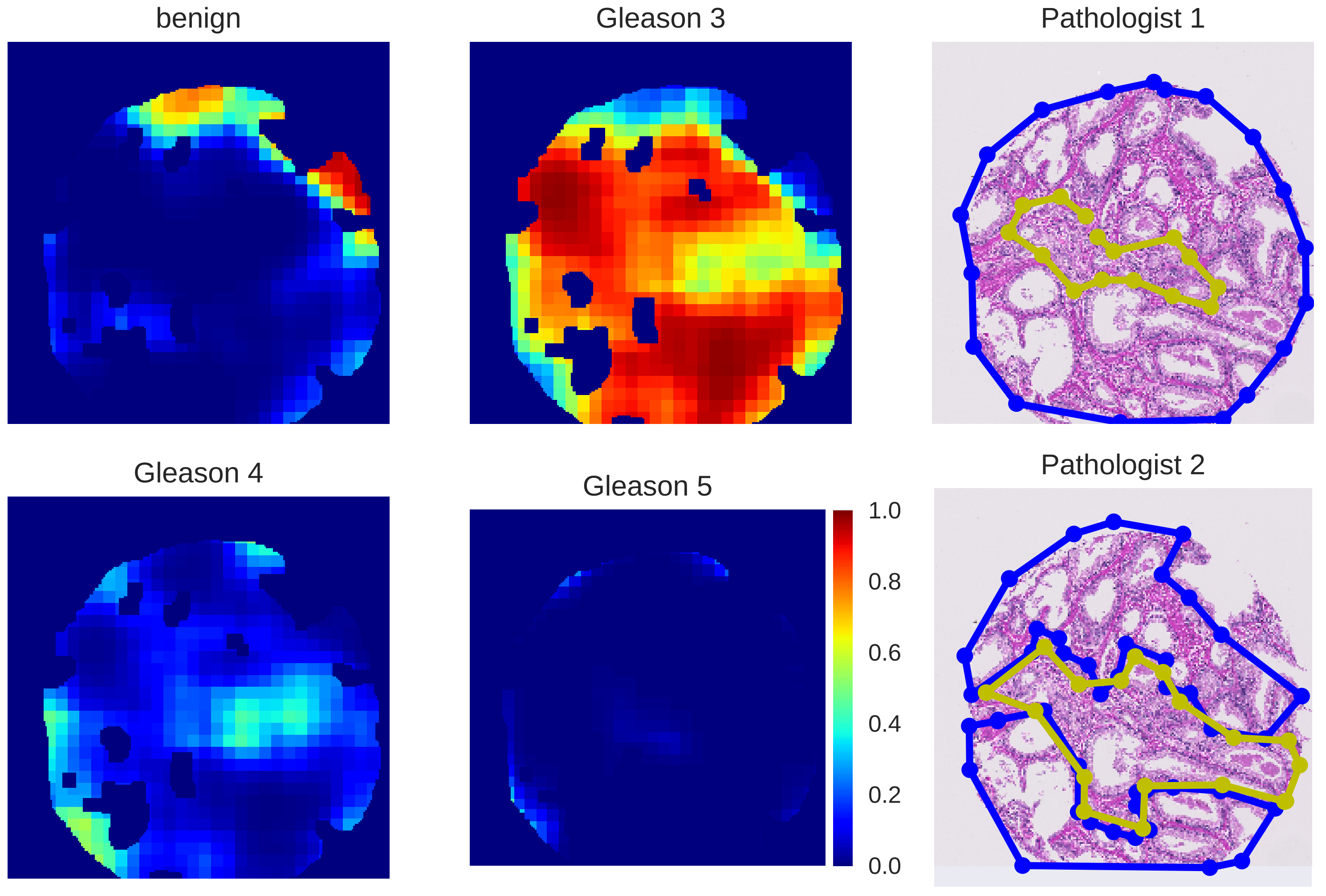

benign
Gleason 3
Pathologist 1
Gleason 4
Gleason 5
Pathologist 2
1.0
0.8
0.6
0.4
0.2
0.0

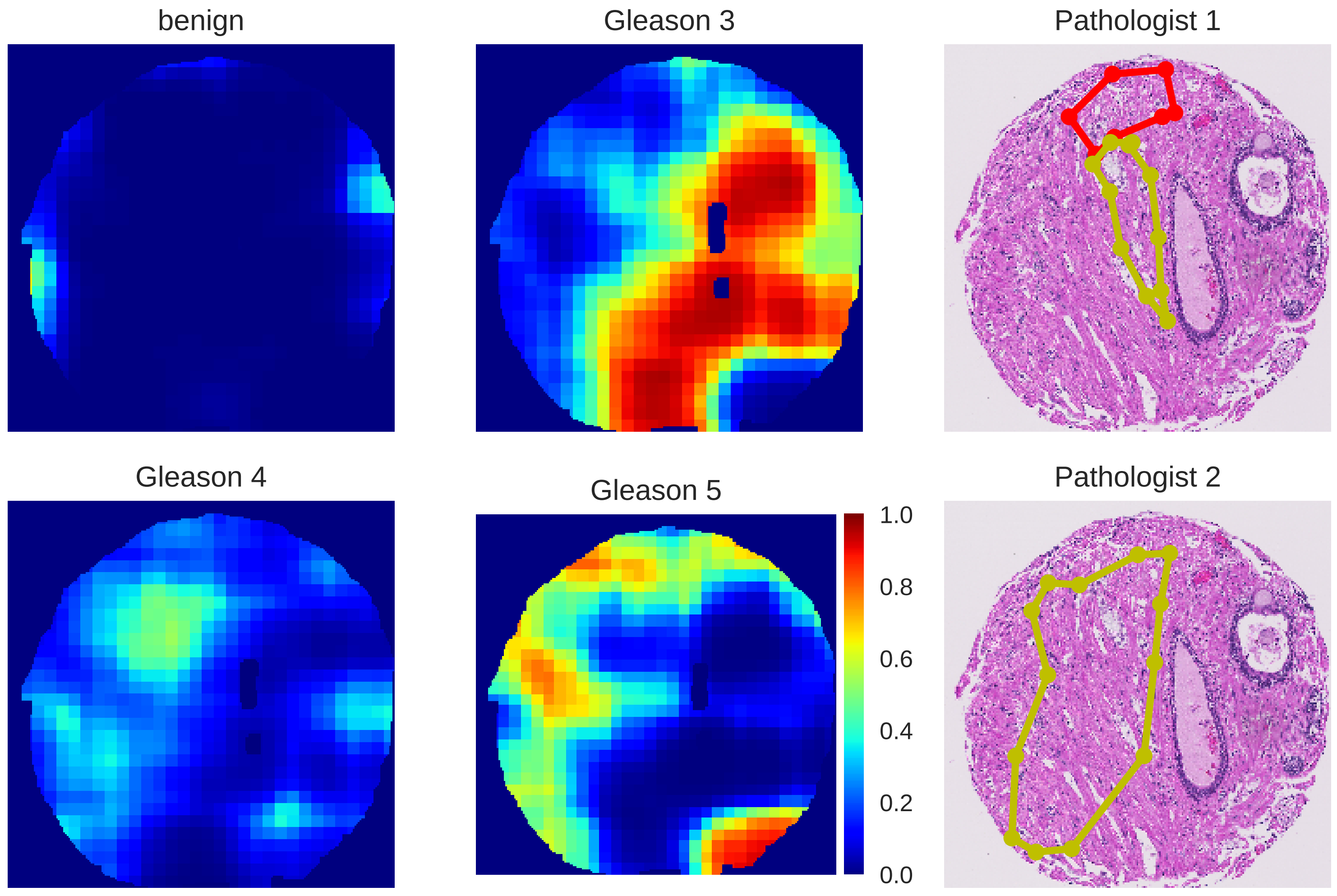

benign
Gleason 3
Pathologist 1
Gleason 4
Gleason 5
Pathologist 2
1.0
0.8
0.6
0.4
0.2
0.0

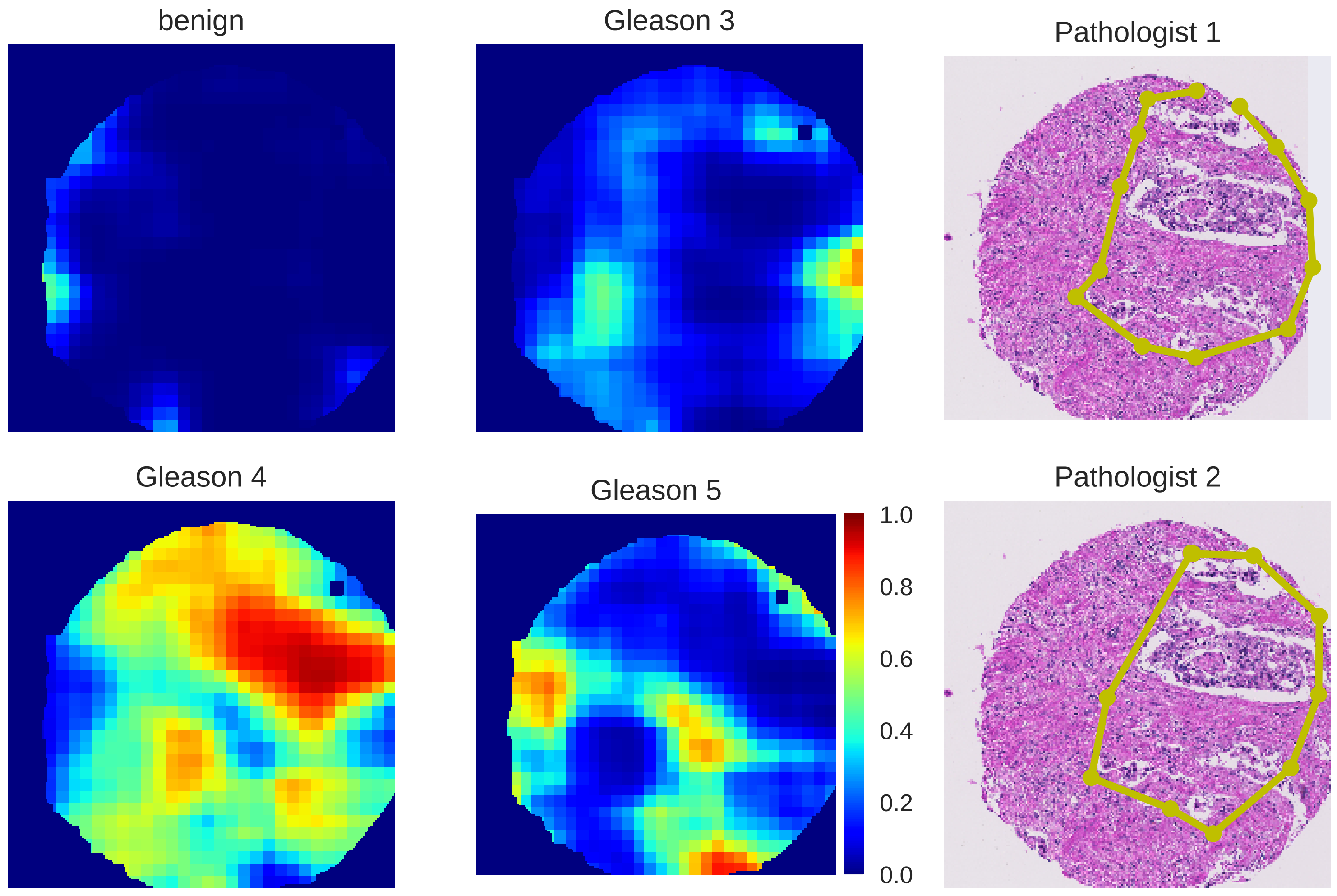
benign
Gleason 3
Pathologist 1
Gleason 4
Gleason 5
Pathologist 2
1.0
0.8
0.6
0.4
0.2
0.0

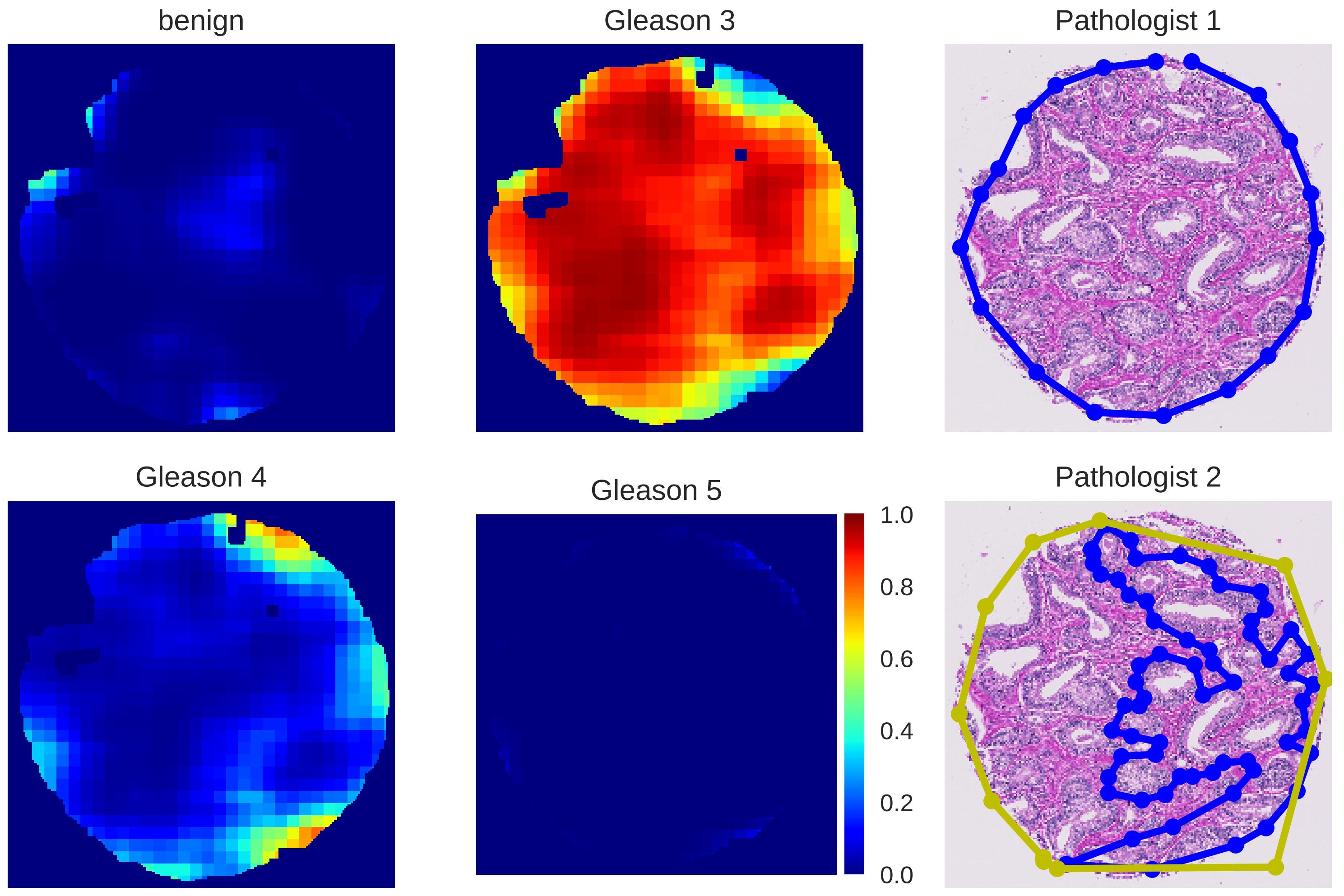
benign
Gleason 3
Pathologist 1
Gleason 4
Gleason 5
Pathologist 2
1.0
0.8
0.6
0.4
0.2
0.0

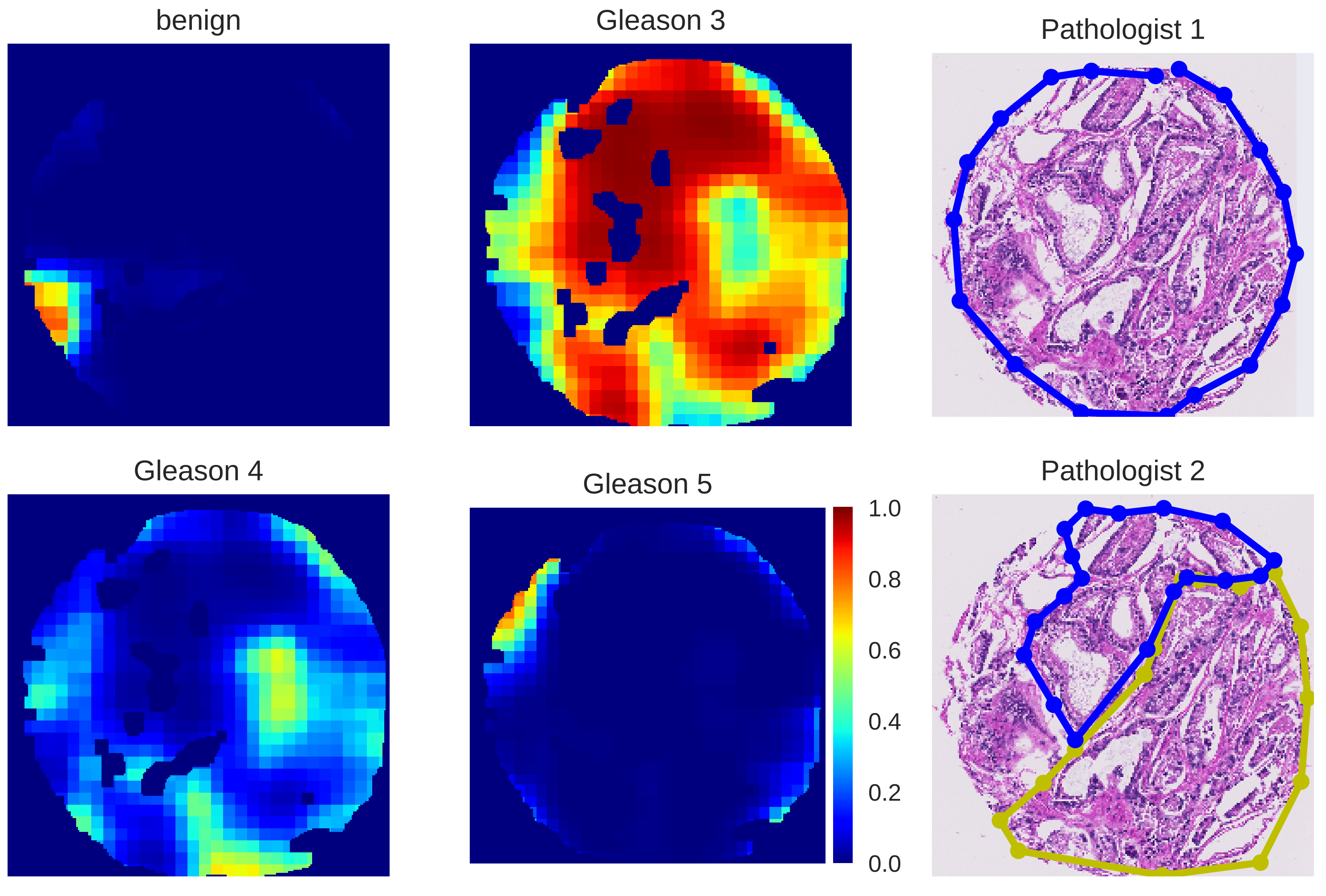

benign
Gleason 3
Pathologist 1
Gleason 4
Gleason 5
Pathologist 2
1.0
0.8
0.6
0.4
0.2
0.0

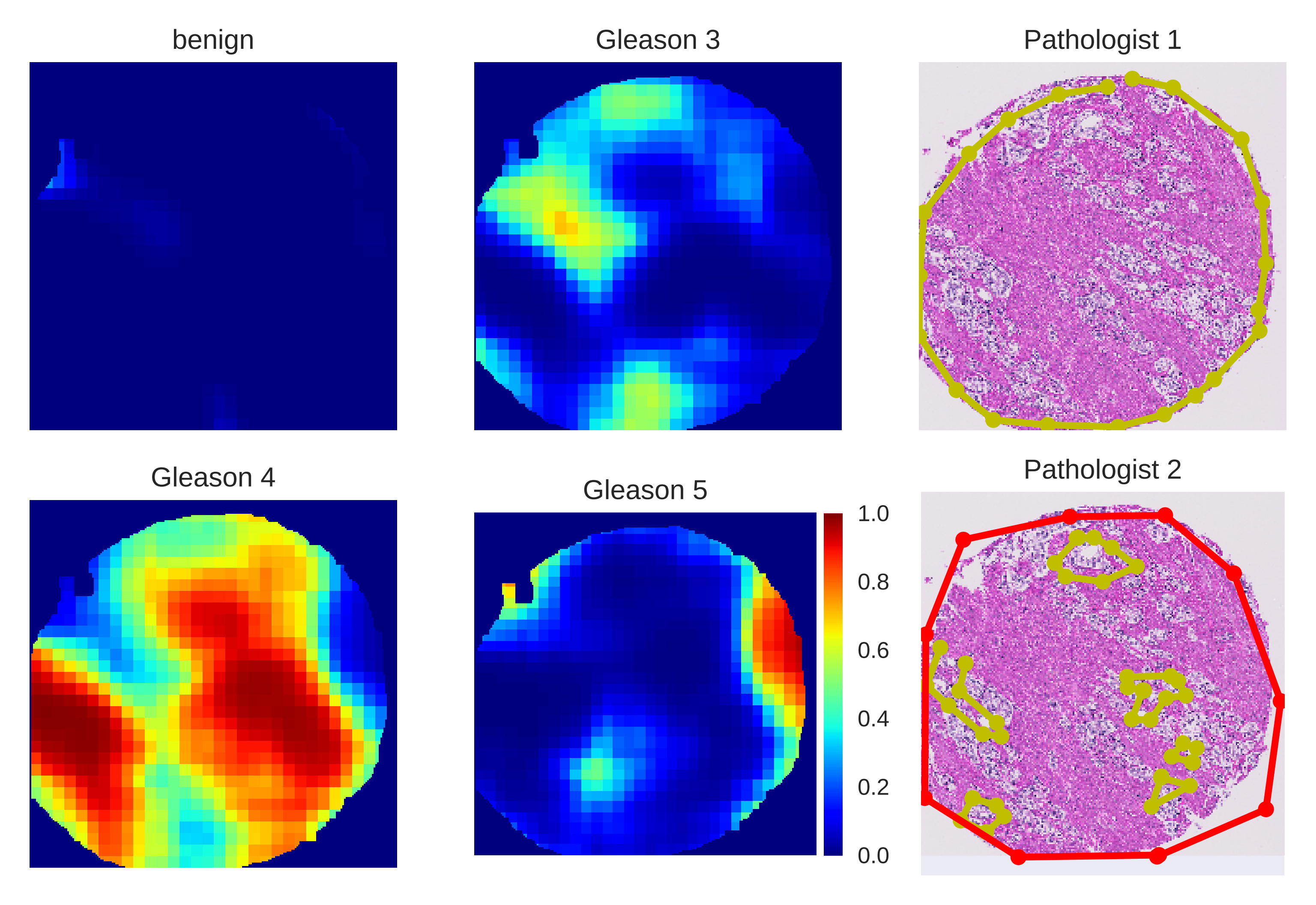
benign
Gleason 3
Pathologist 1
Gleason 4
Gleason 5
Pathologist 2
1.0
0.8
0.6
0.4
0.2
0.0

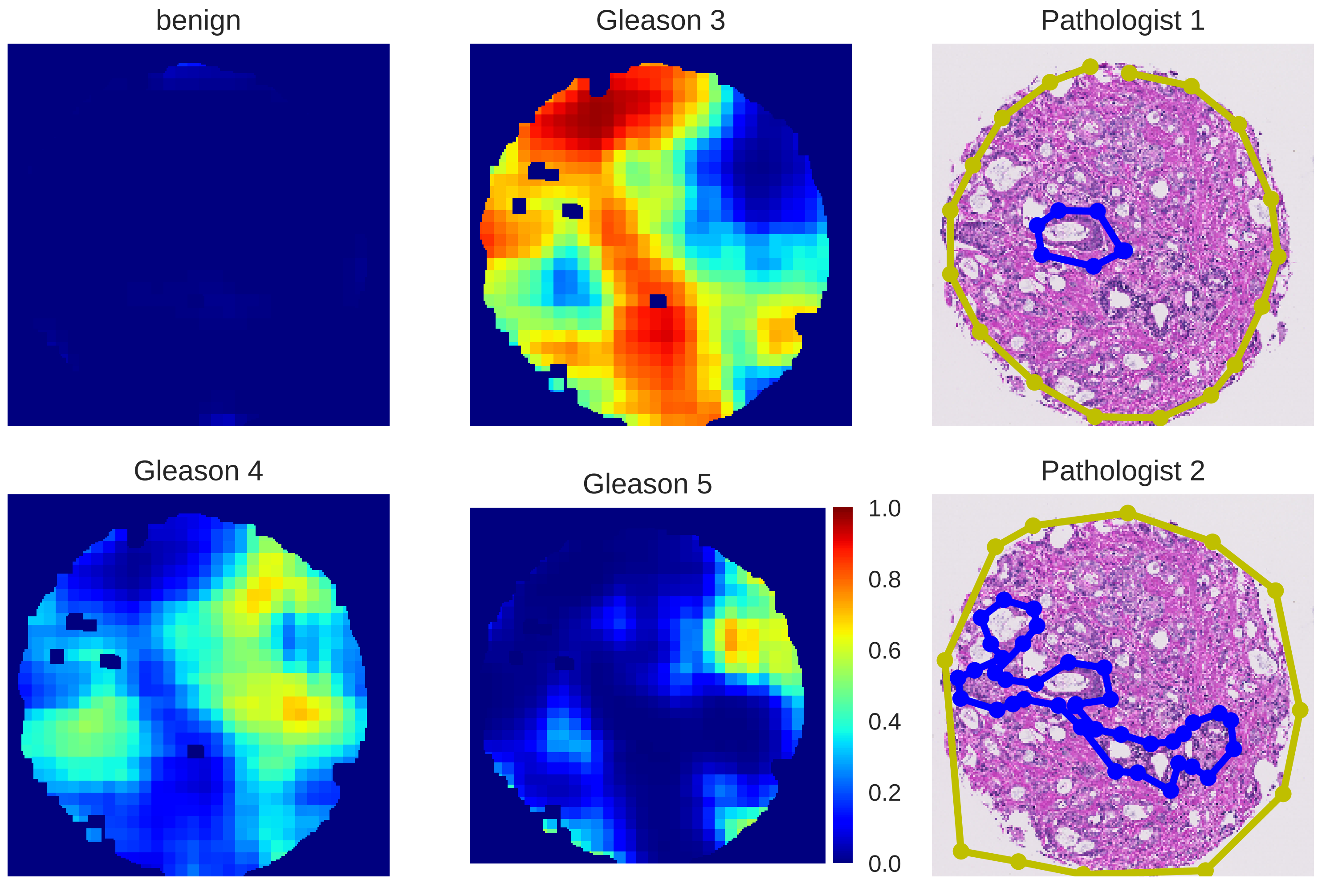
benign
Gleason 3
Pathologist 1
Gleason 4
Gleason 5
Pathologist 2
1.0
0.8
0.6
0.4
0.2
0.0

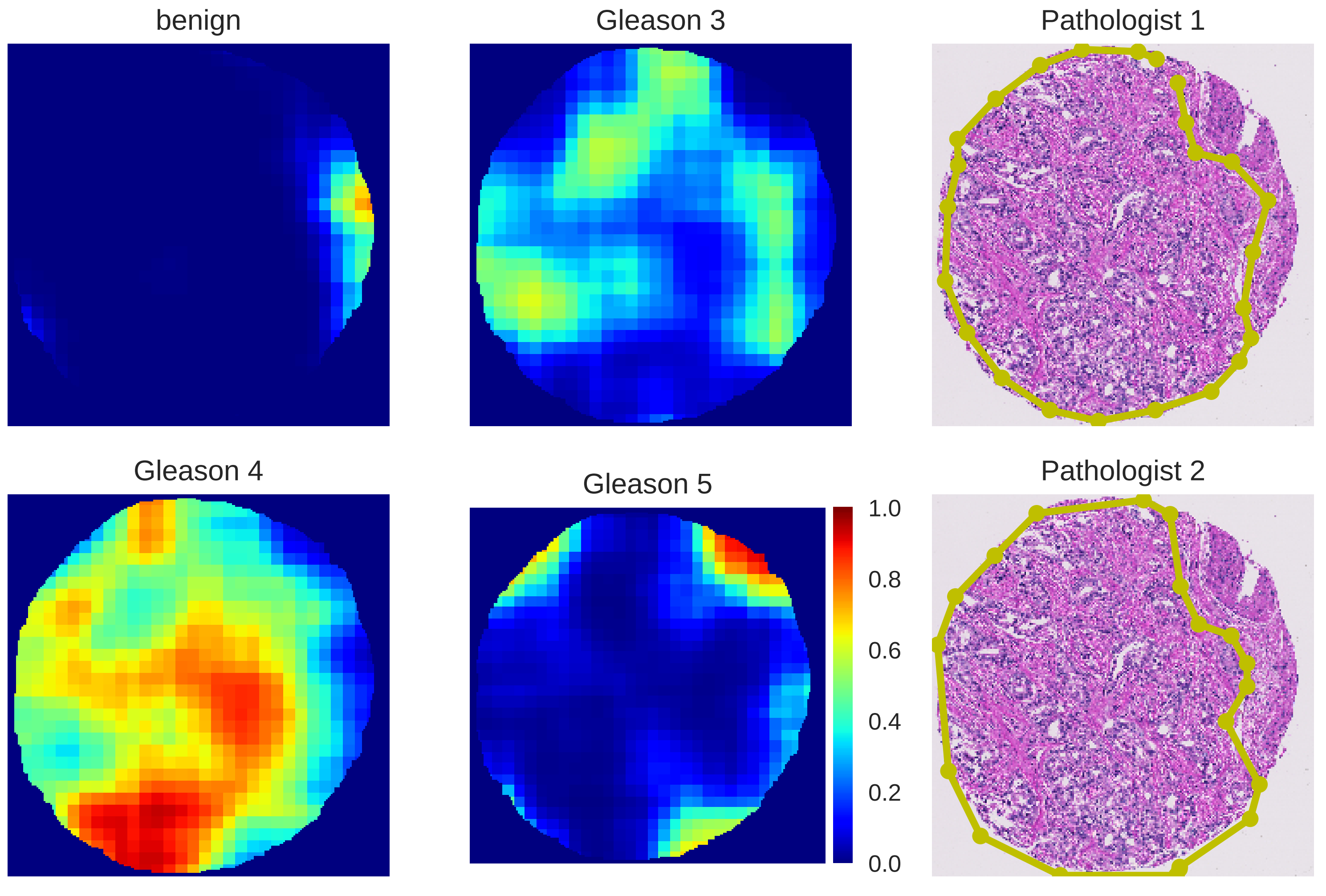

benign
Gleason 3
Pathologist 1
Gleason 4
Gleason 5
Pathologist 2
1.0
0.8
0.6
0.4
0.2
0.0

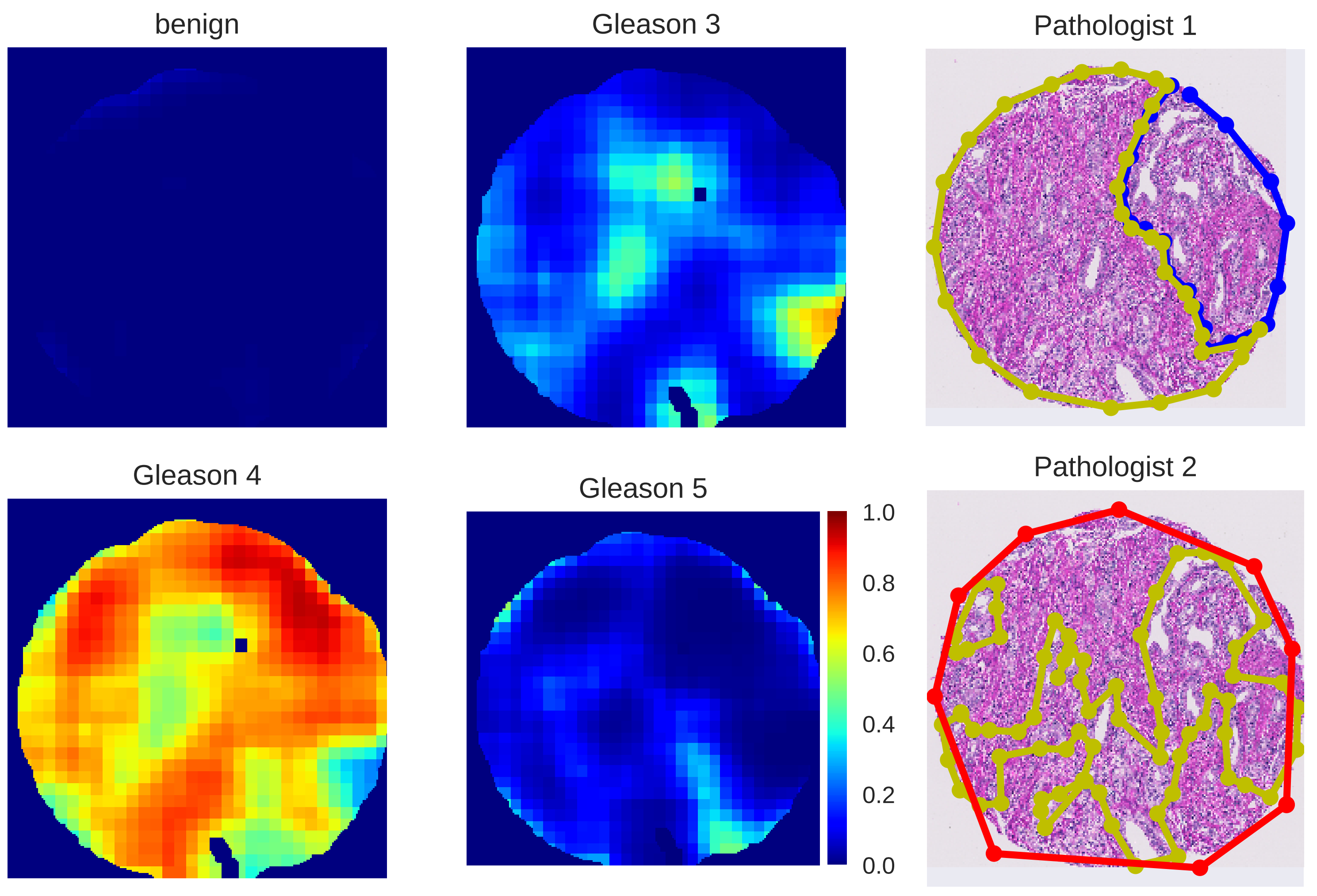

benign
Gleason 3
Pathologist 1
Gleason 4
Gleason 5
Pathologist 2
1.0
0.8
0.6
0.4
0.2
0.0

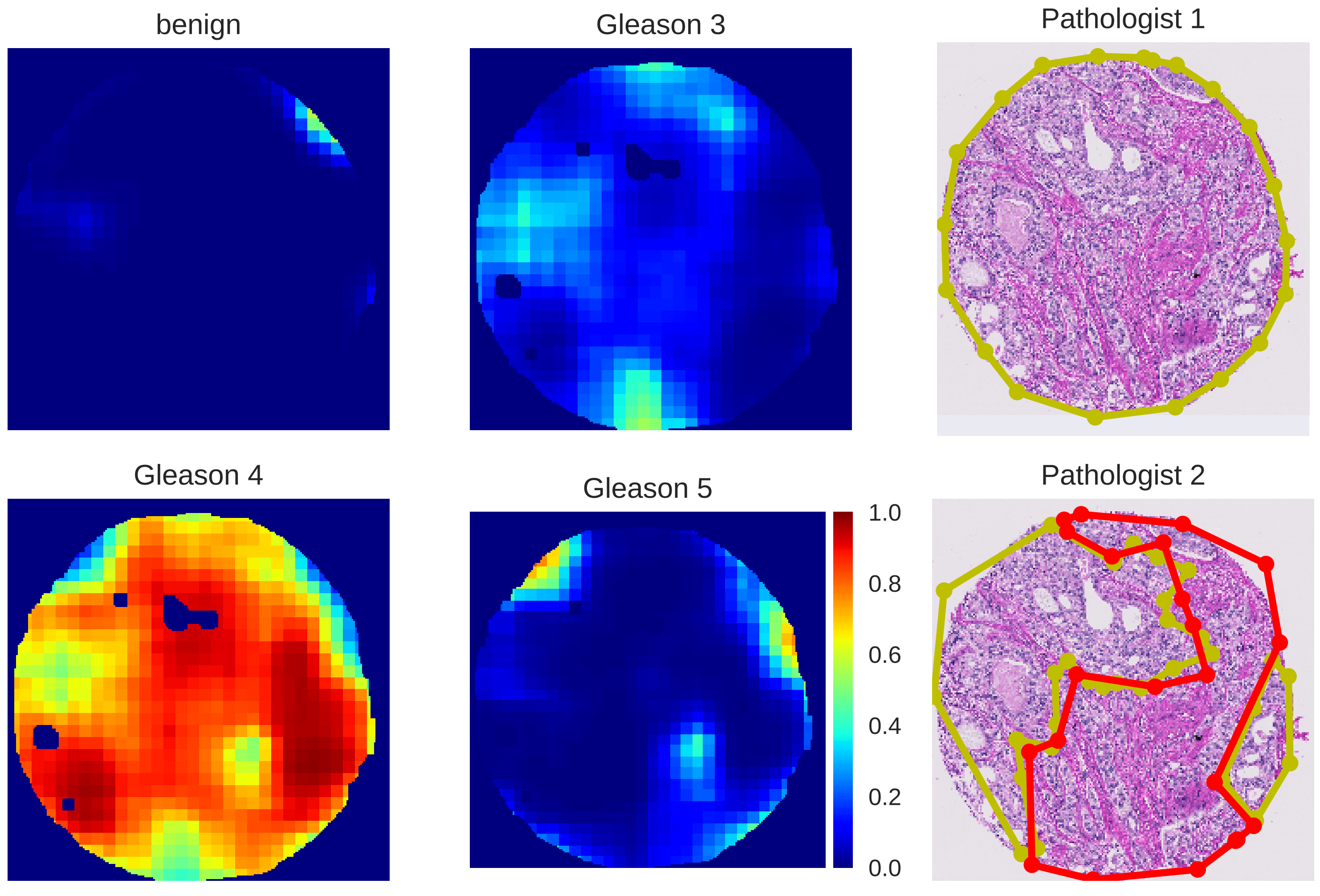
benign
Gleason 3
Pathologist 1
Gleason 4
Gleason 5
Pathologist 2
1.0
0.8
0.6
0.4
0.2
0.0

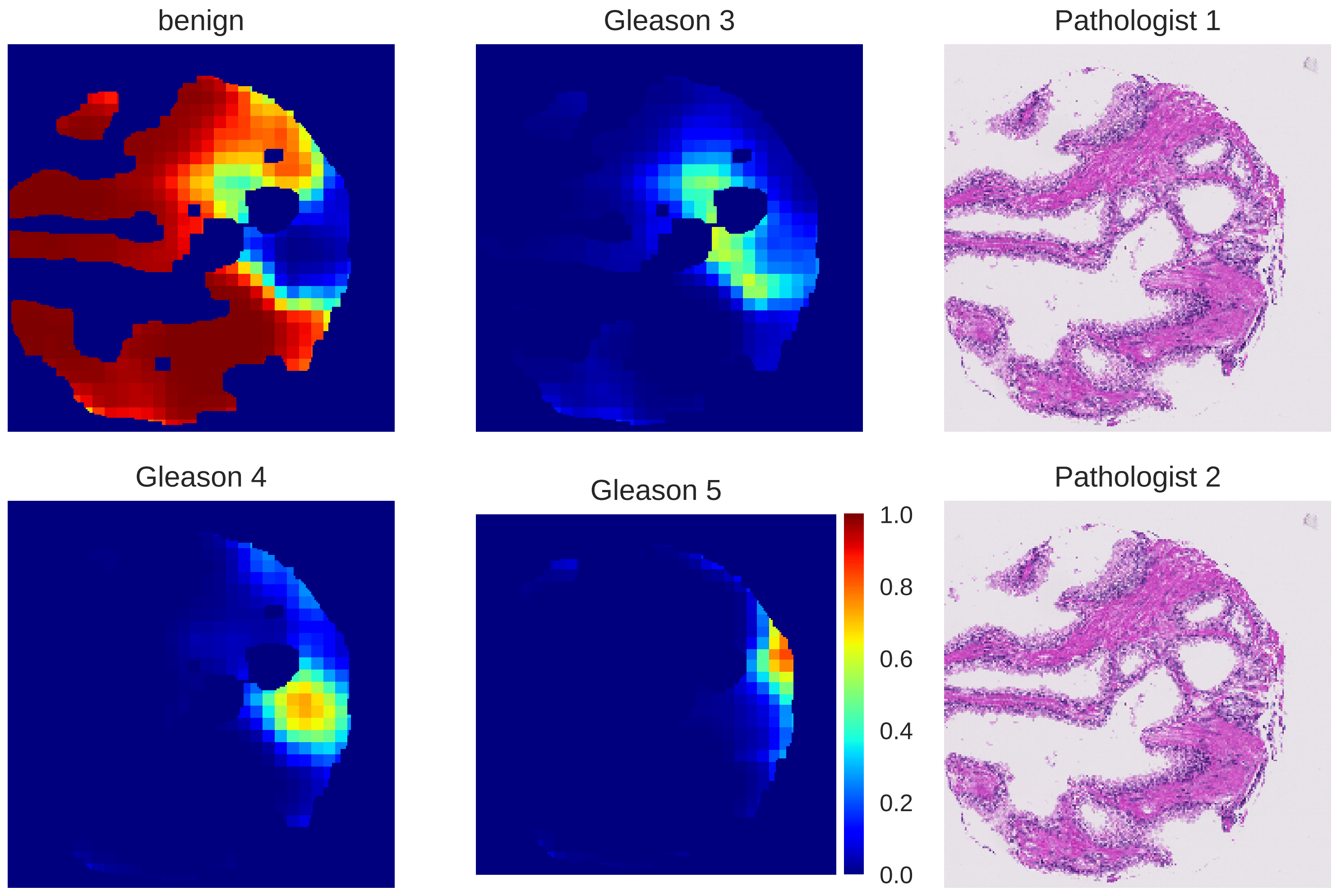

benign
Gleason 3
Pathologist 1
Gleason 4
Gleason 5
Pathologist 2
1.0
0.8
0.6
0.4
0.2
0.0

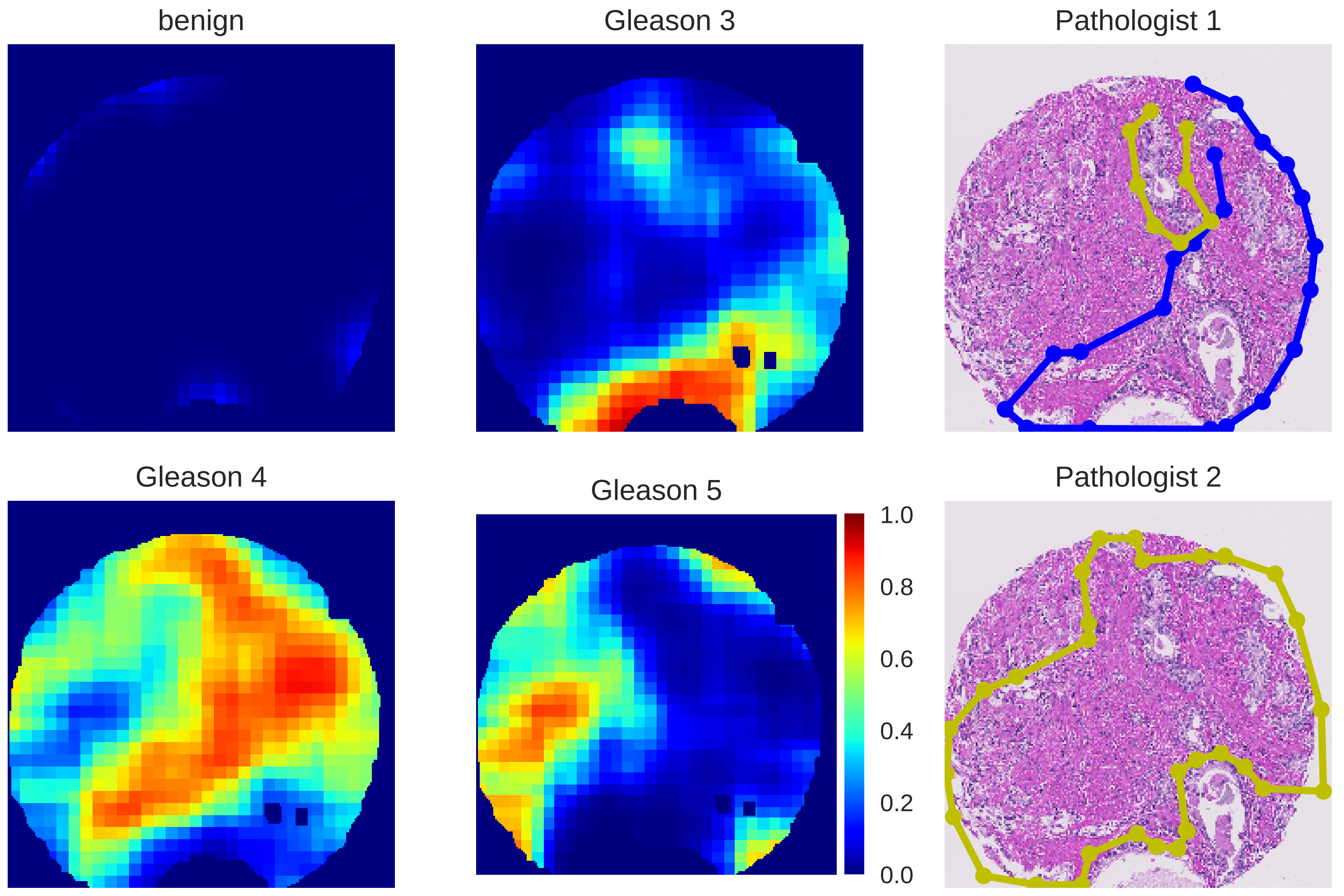
benign
Gleason 3
Pathologist 1
Gleason 4
Gleason 5
Pathologist 2
1.0
0.8
0.6
0.4
0.2
0.0

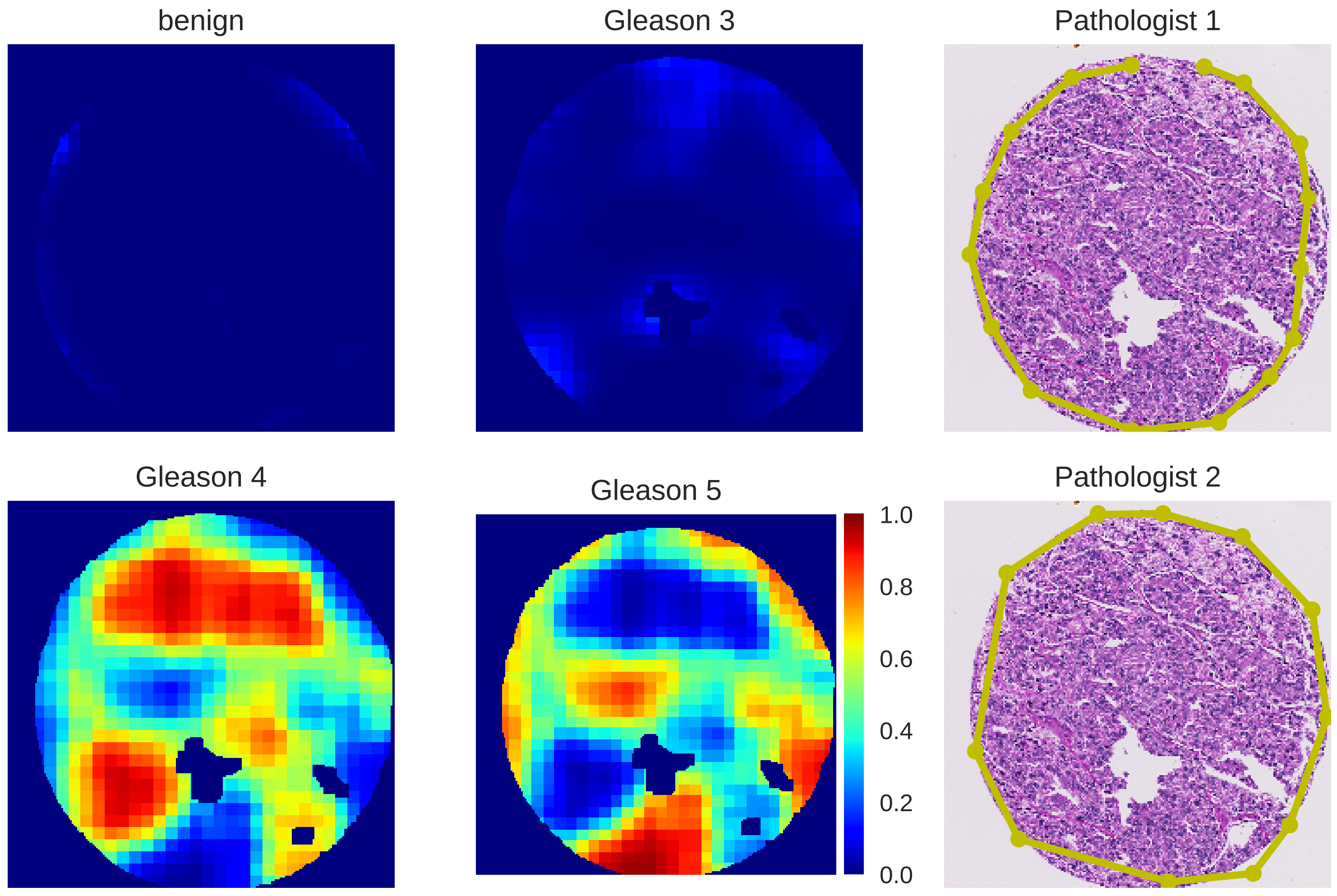

benign
Gleason 3
Pathologist 1
Gleason 4
Gleason 5
Pathologist 2
1.0
0.8
0.6
0.4
0.2
0.0

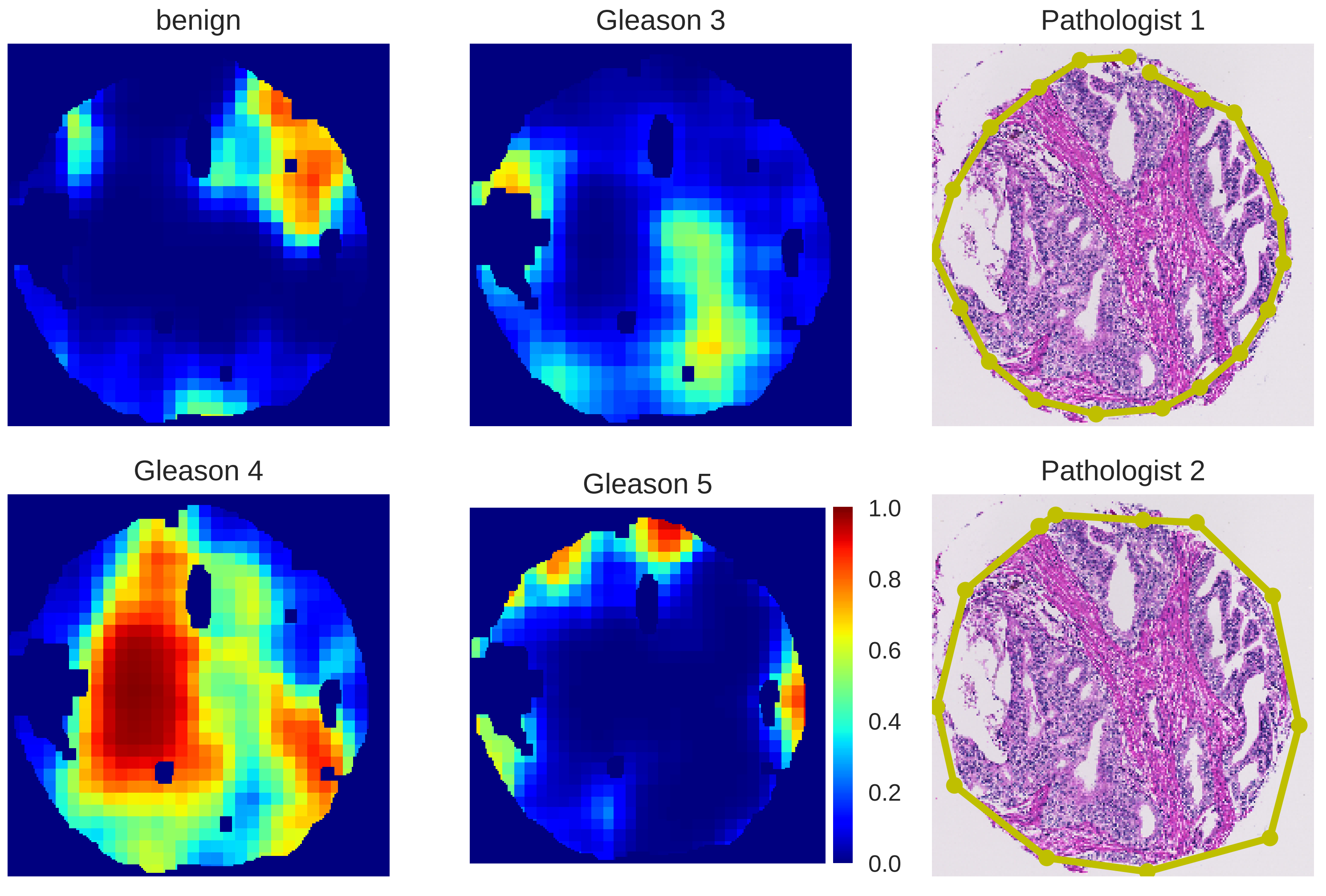

benign
Gleason 3
Pathologist 1
Gleason 4
Gleason 5
Pathologist 2
1.0
0.8
0.6
0.4
0.2
0.0

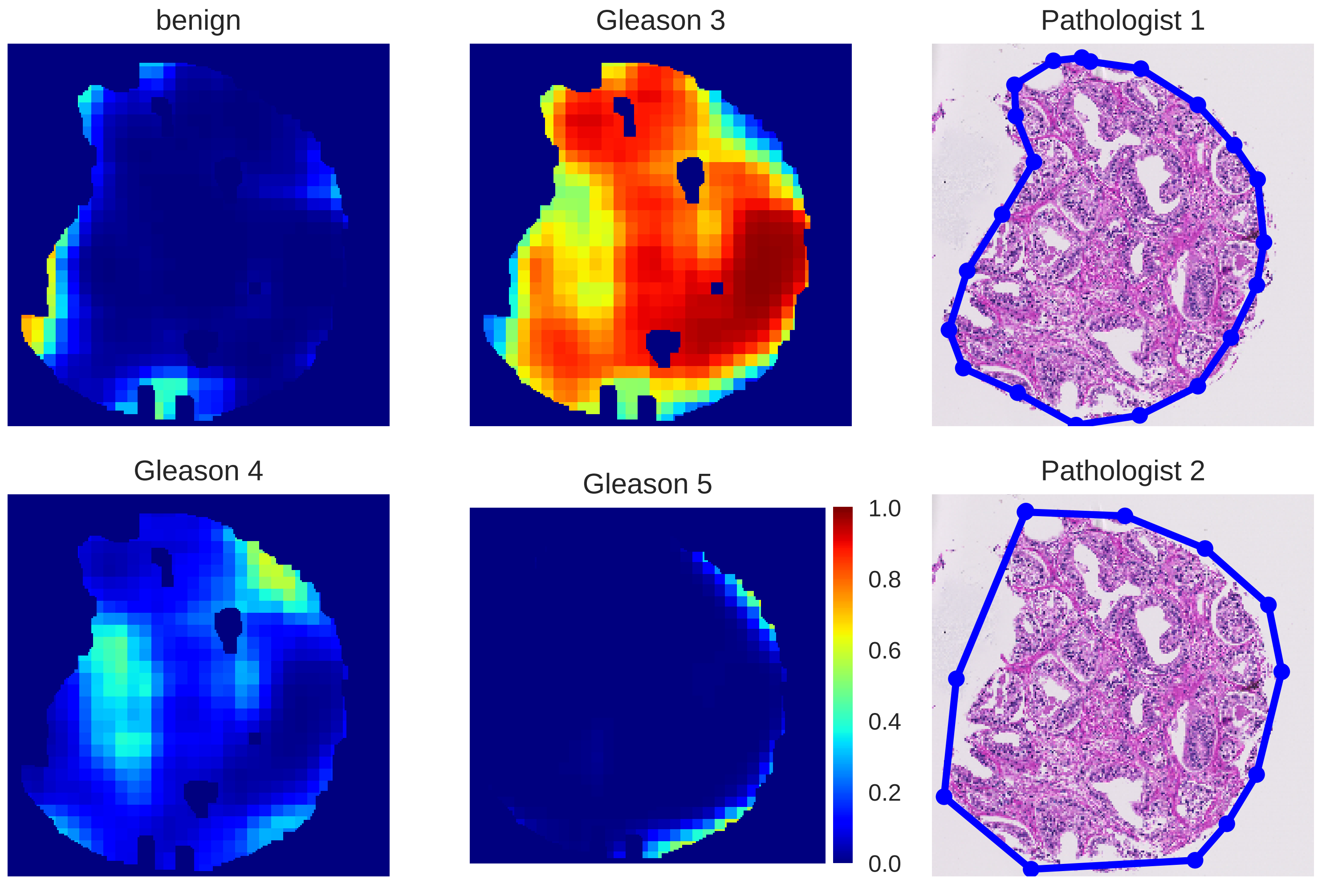

benign
Gleason 3
Pathologist 1
Gleason 4
Gleason 5
Pathologist 2
1.0
0.8
0.6
0.4
0.2
0.0

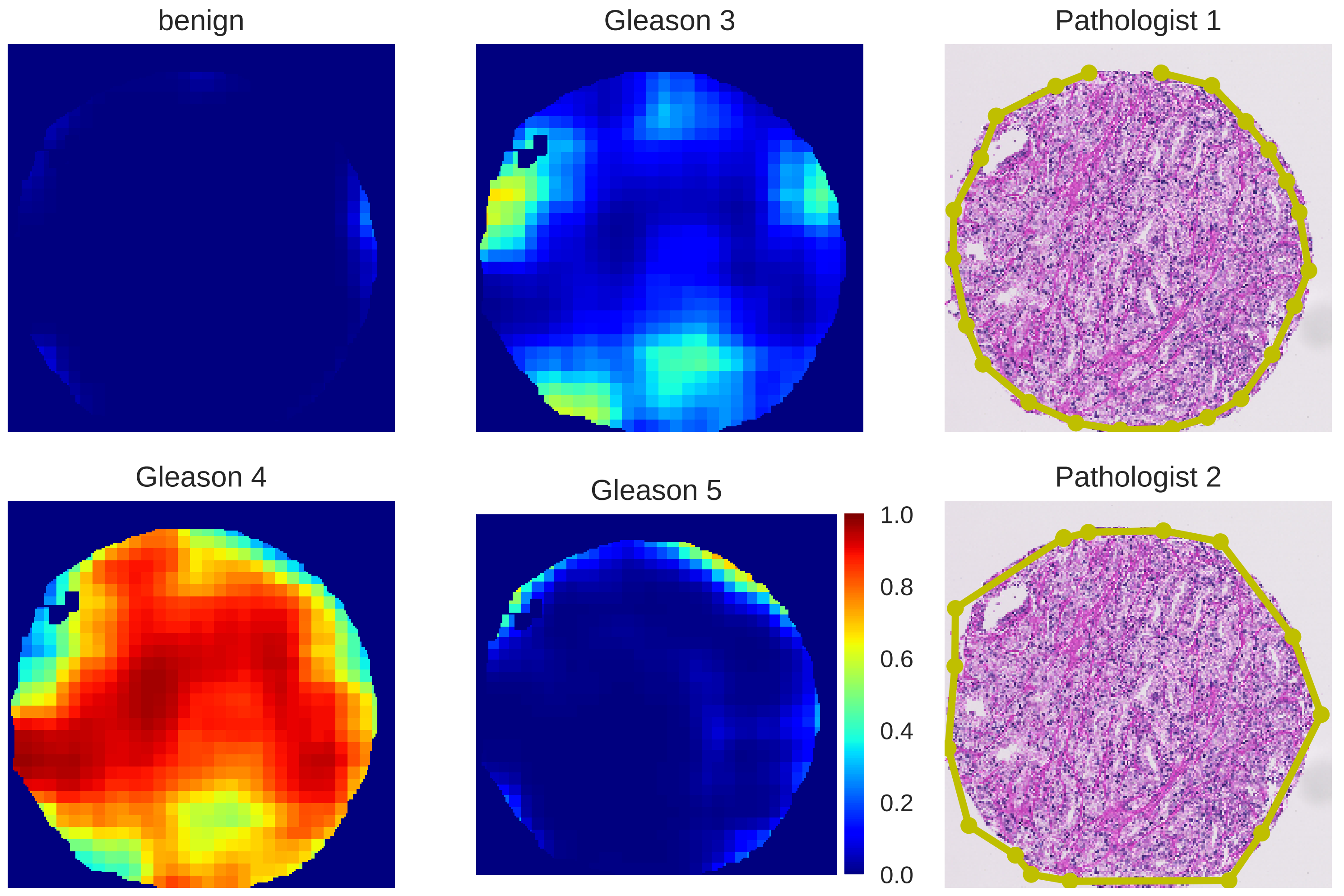

benign
Gleason 3
Pathologist 1
Gleason 4
Gleason 5
Pathologist 2
1.0
0.8
0.6
0.4
0.2
0.0

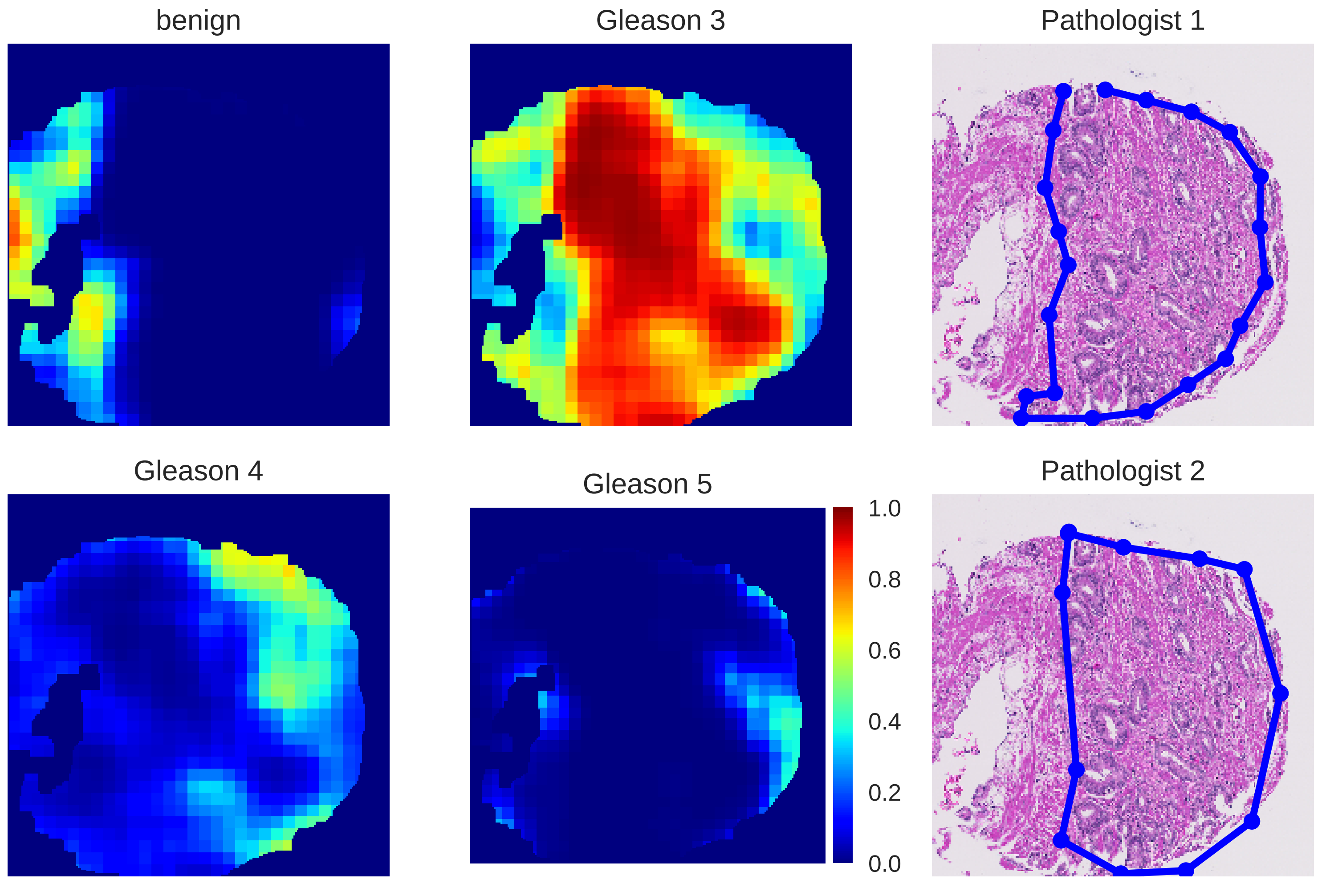

benign
Gleason 3
Pathologist 1
Gleason 4
Gleason 5
Pathologist 2
1.0
0.8
0.6
0.4
0.2
0.0

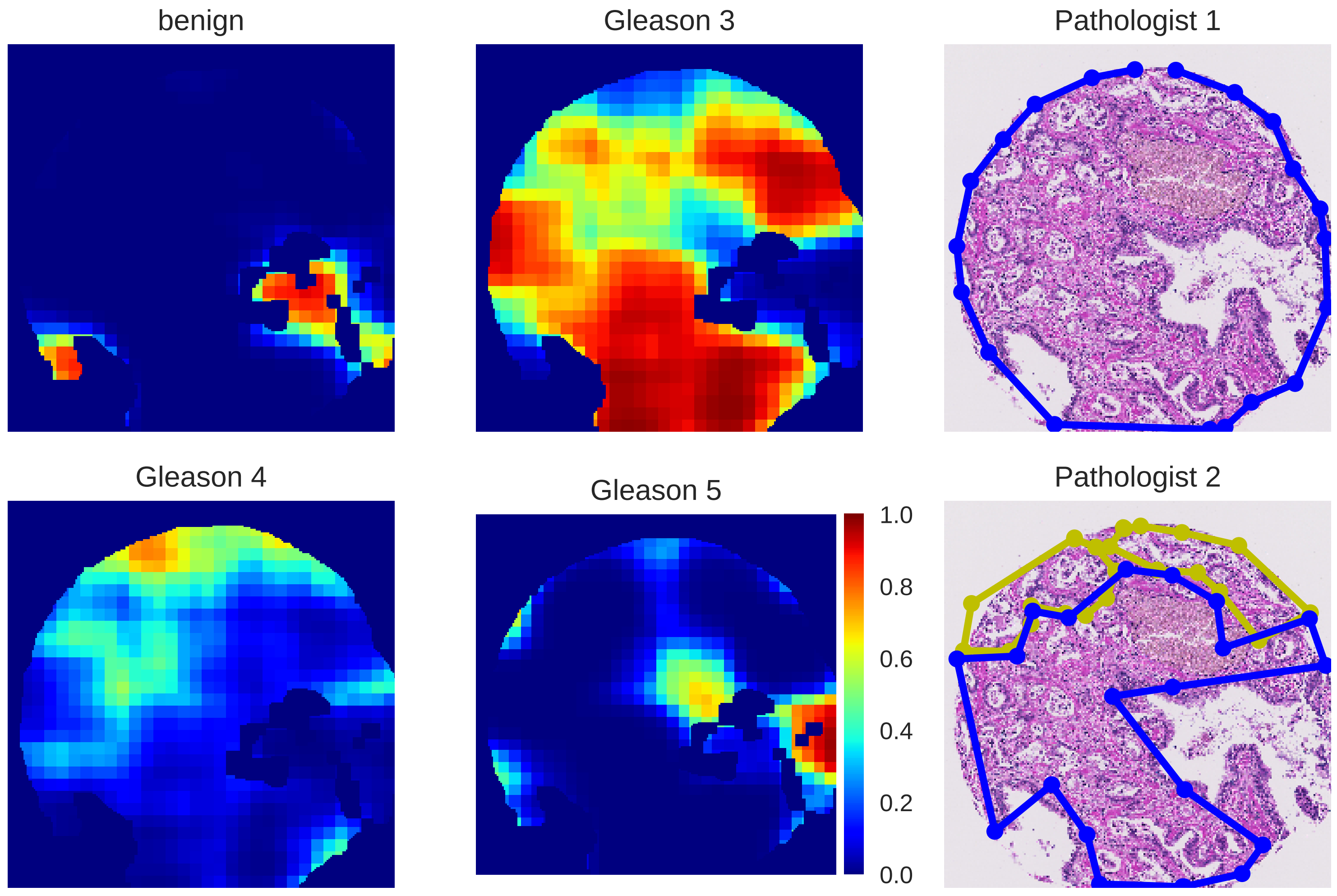
benign
Gleason 3
Pathologist 1
Gleason 4
Gleason 5
Pathologist 2
1.0
0.8
0.6
0.4
0.2
0.0

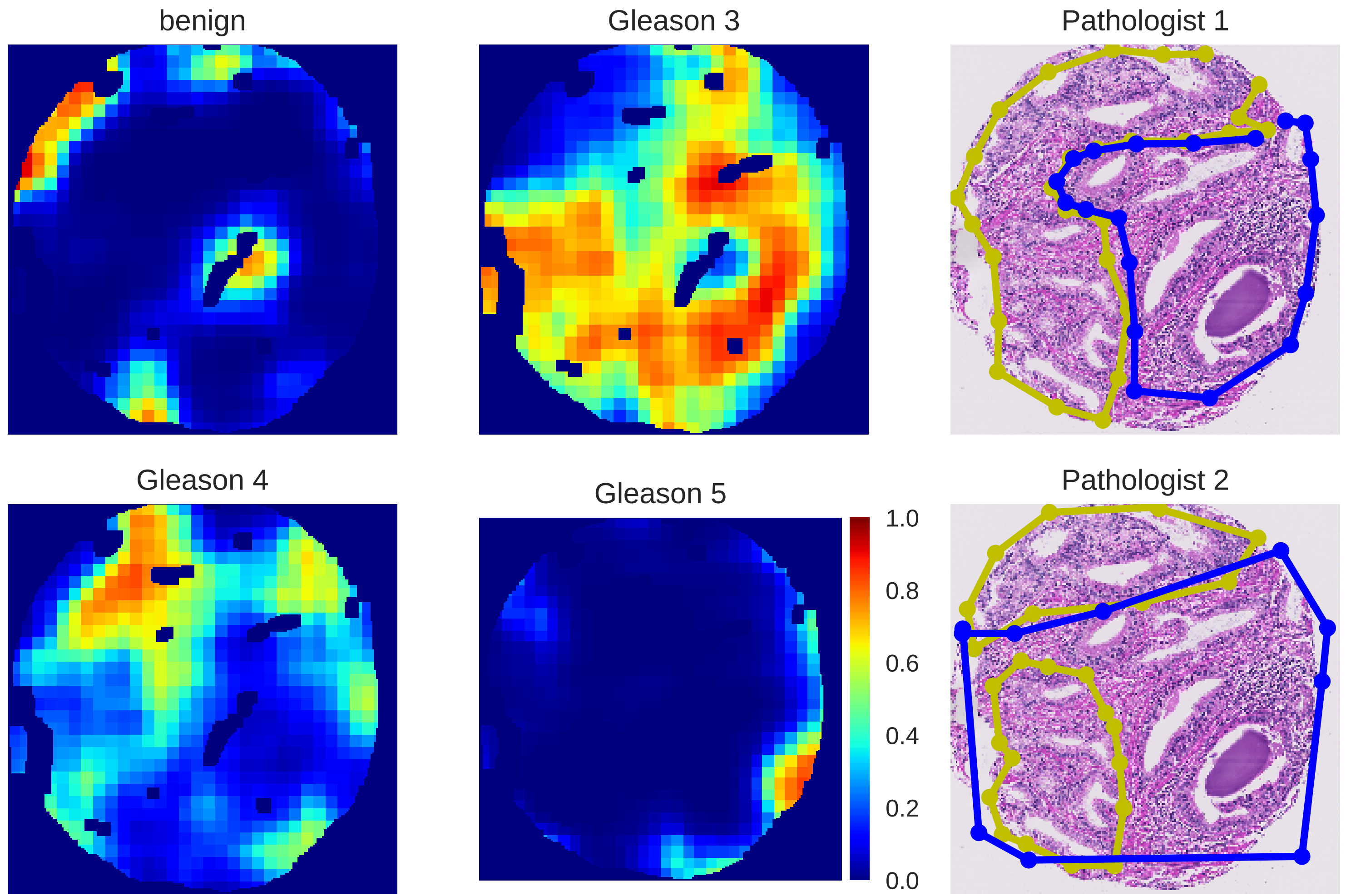

benign
Gleason 3
Pathologist 1
Gleason 4
Gleason 5
Pathologist 2
1.0
0.8
0.6
0.4
0.2
0.0

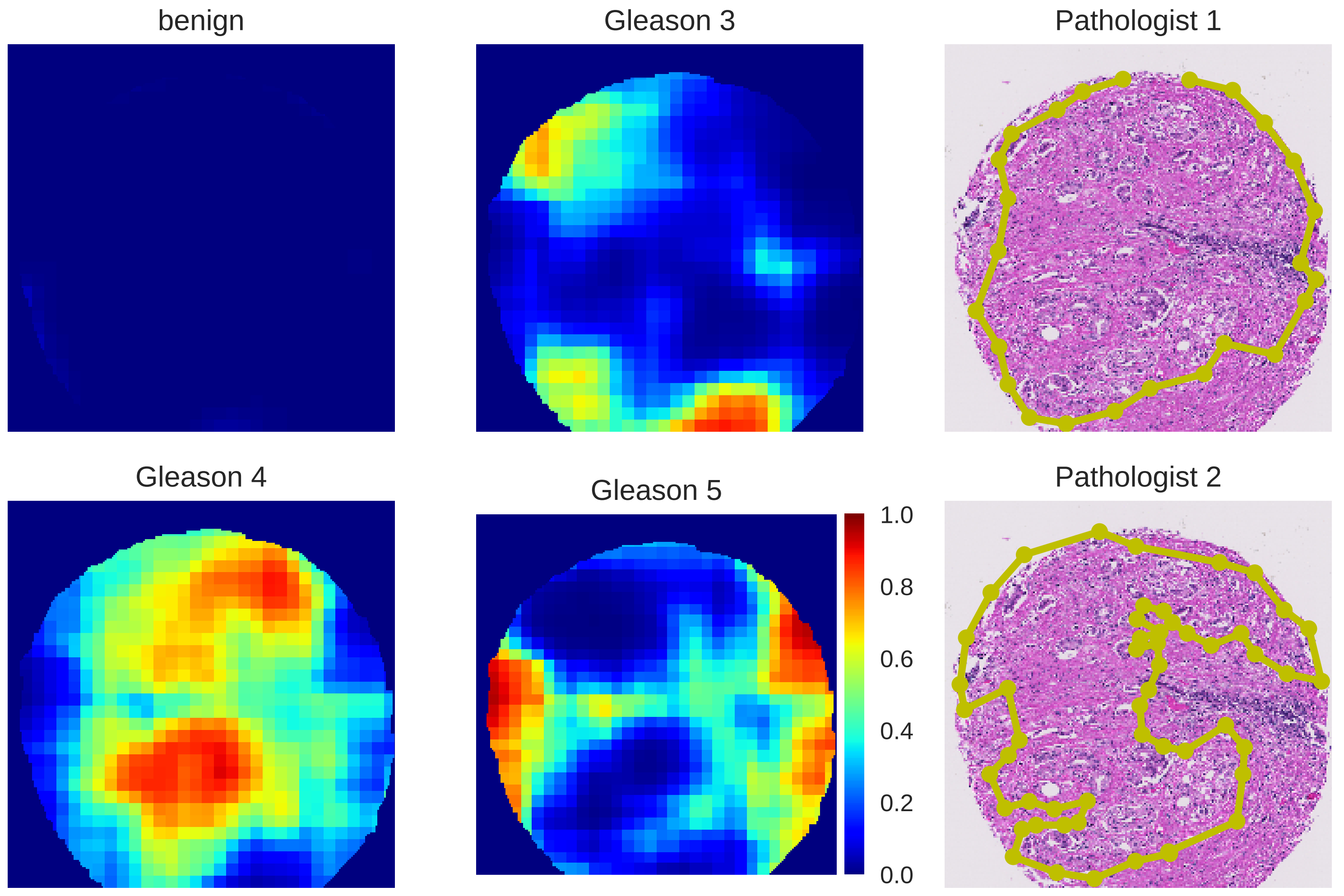
benign
Gleason 3
Pathologist 1
Gleason 4
Gleason 5
Pathologist 2
1.0
0.8
0.6
0.4
0.2
0.0

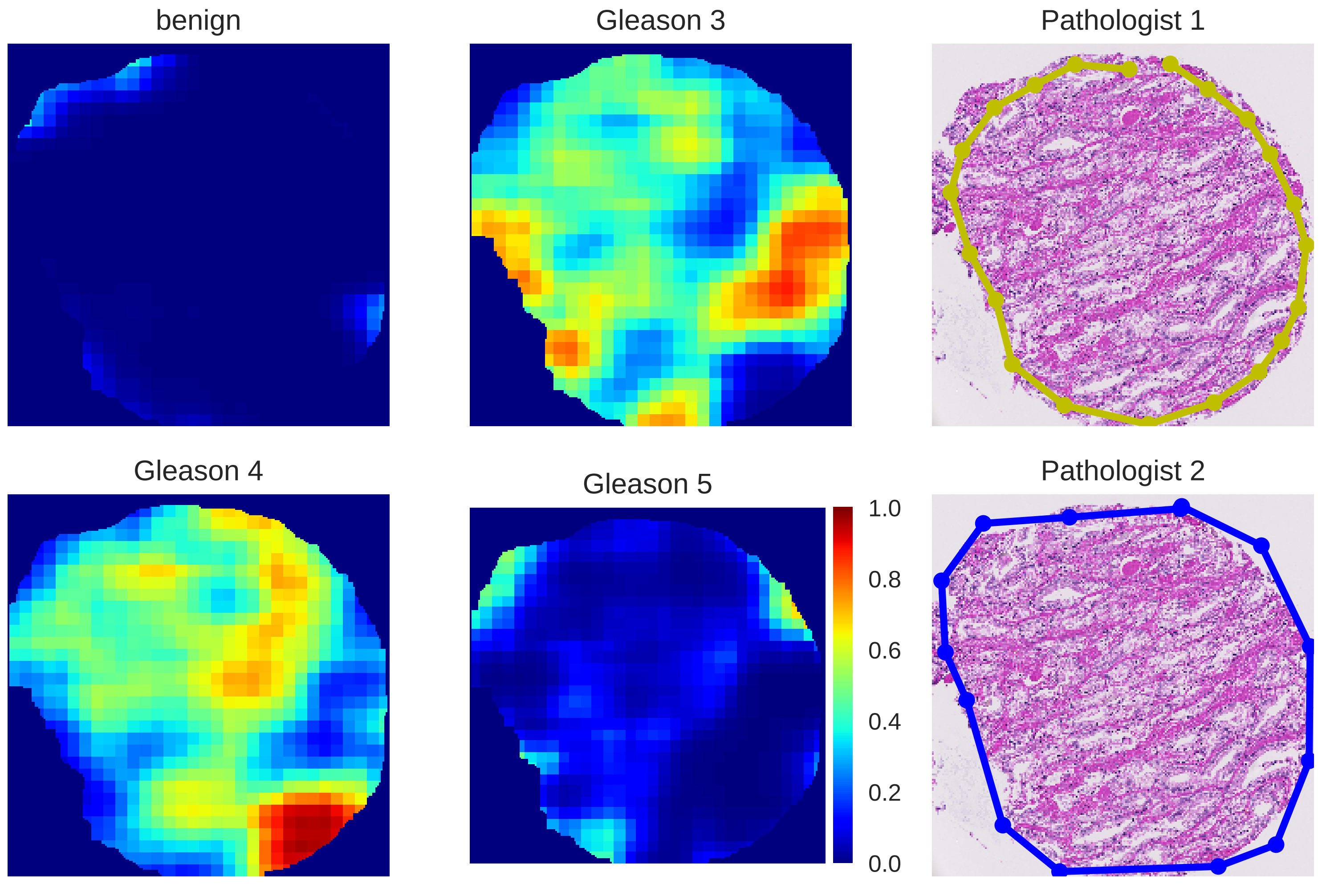
benign
Gleason 3
Pathologist 1
Gleason 4
Gleason 5
Pathologist 2
1.0
0.8
0.6
0.4
0.2
0.0

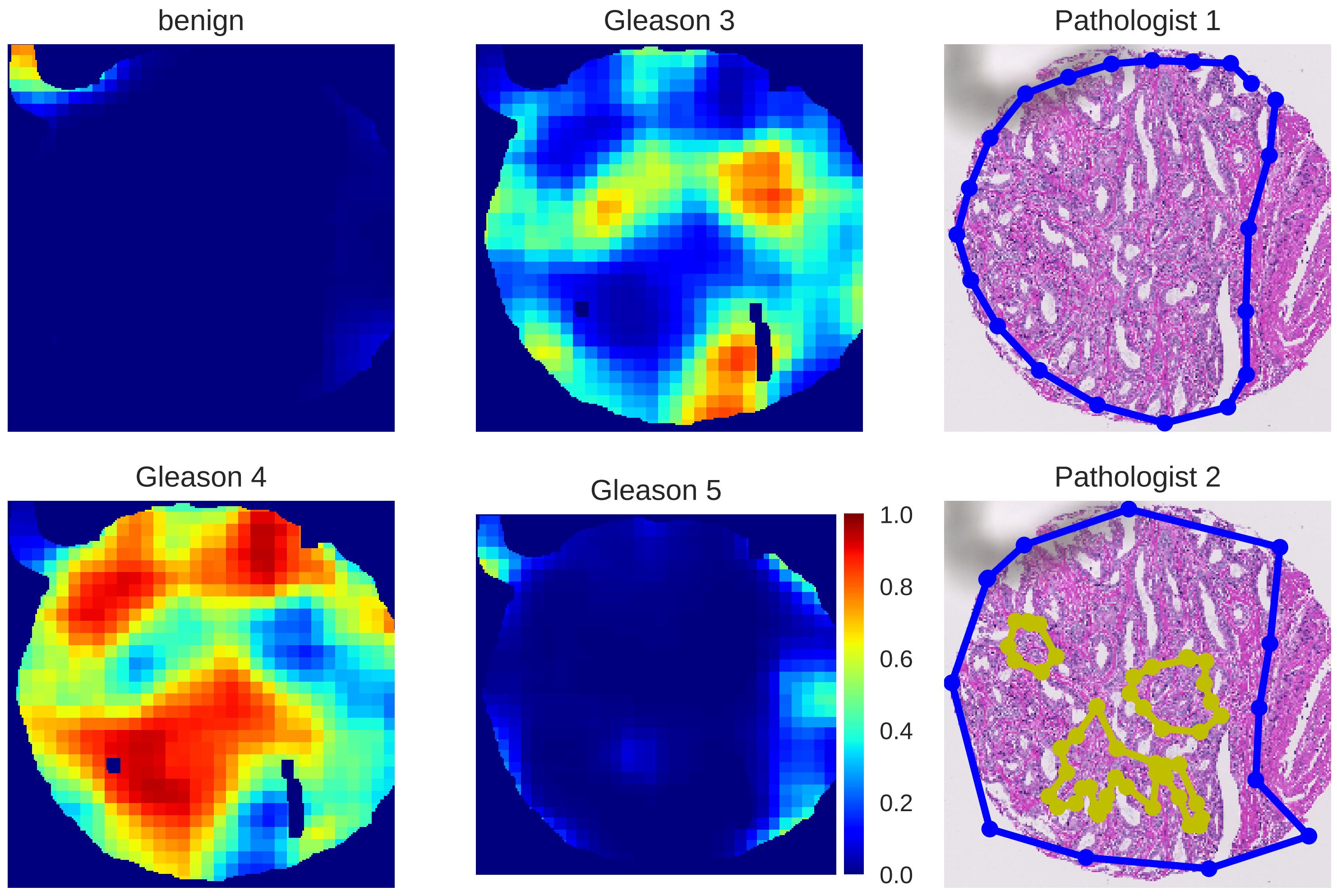
benign
Gleason 3
Pathologist 1
Gleason 4
Gleason 5
Pathologist 2
1.0
0.8
0.6
0.4
0.2
0.0

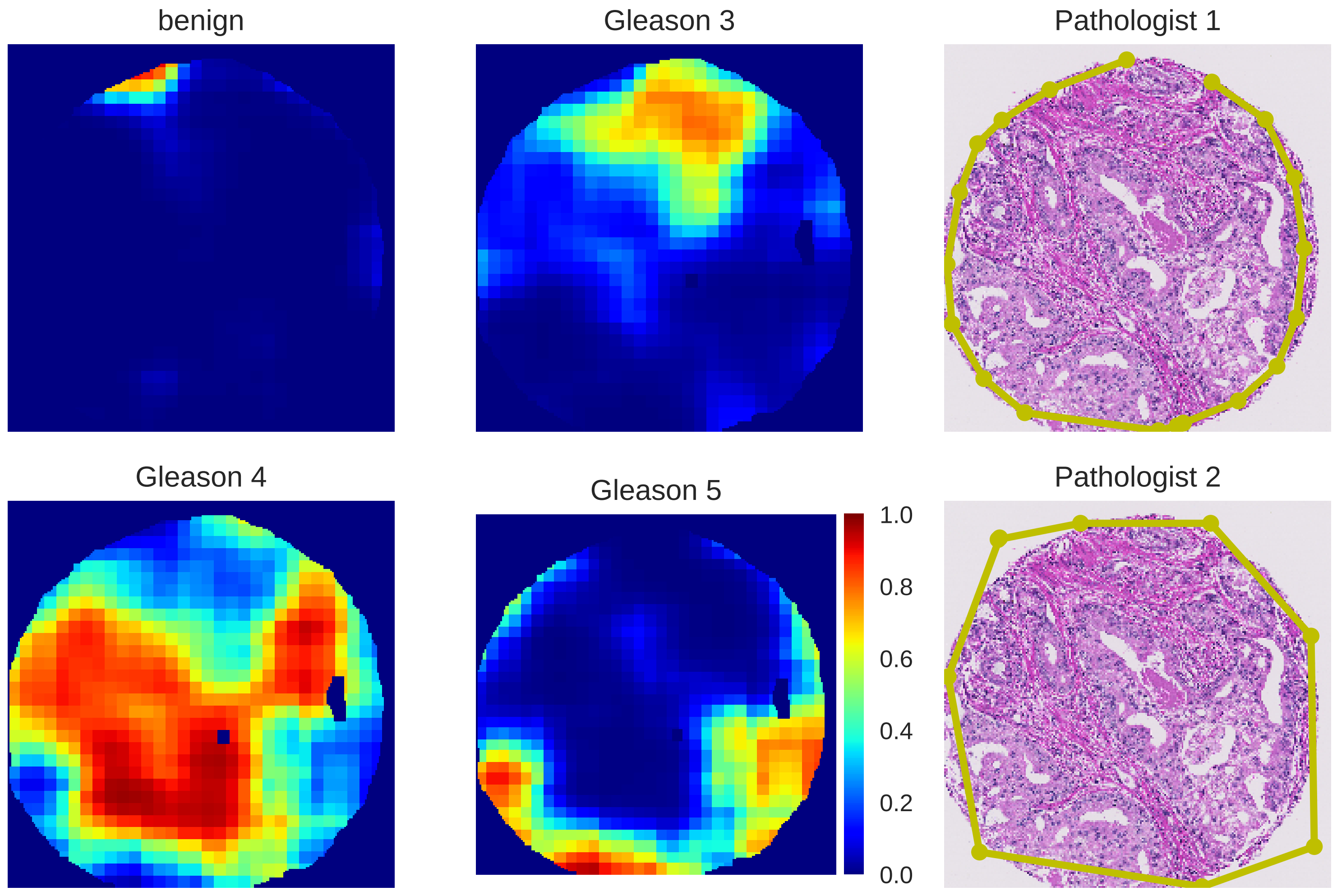

benign
Gleason 3
Pathologist 1
Gleason 4
Gleason 5
Pathologist 2
1.0
0.8
0.6
0.4
0.2
0.0

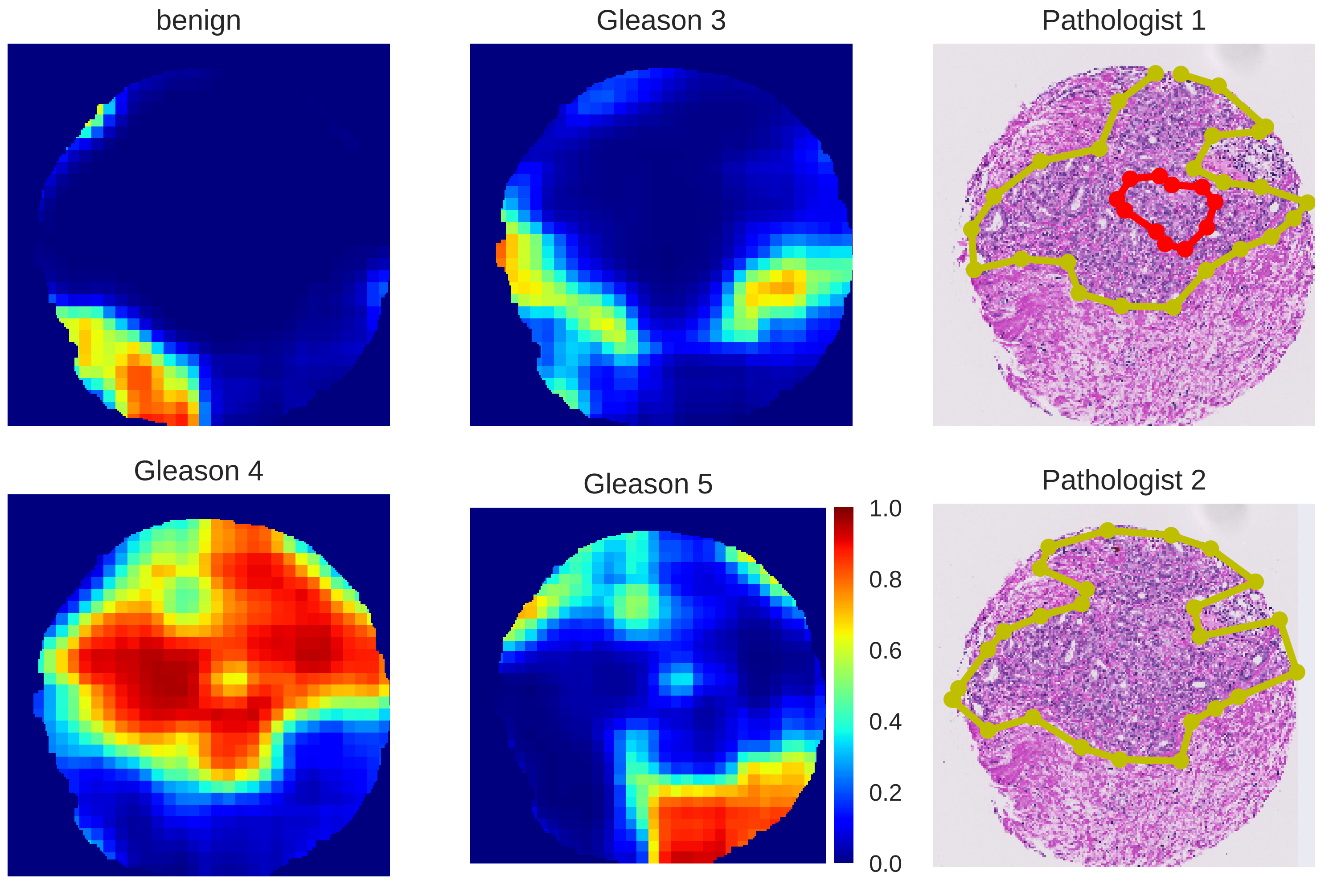
benign
Gleason 3
Pathologist 1
Gleason 4
Gleason 5
Pathologist 2
1.0
0.8
0.6
0.4
0.2
0.0

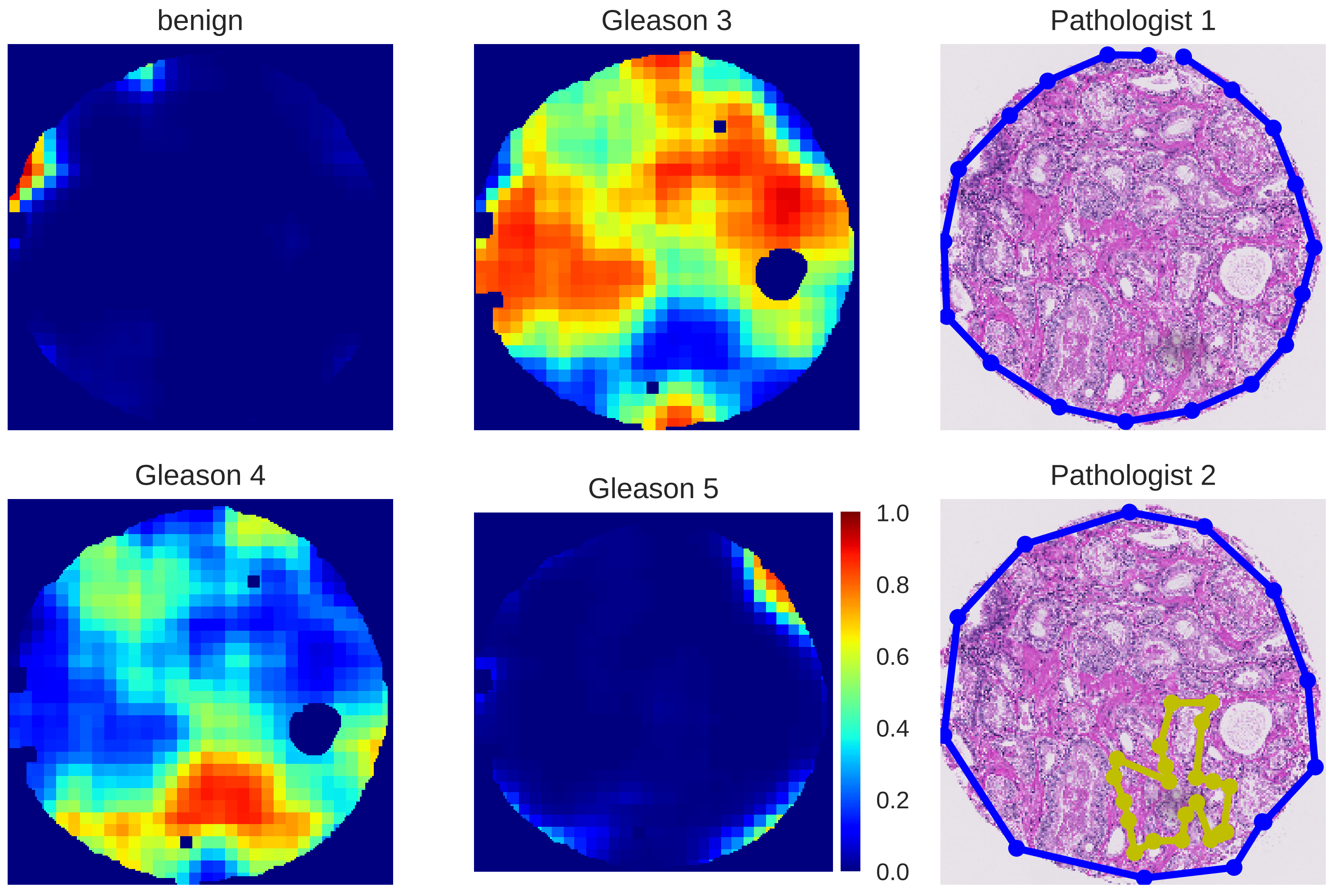
benign
Gleason 3
Pathologist 1
Gleason 4
Gleason 5
Pathologist 2
1.0
0.8
0.6
0.4
0.2
0.0

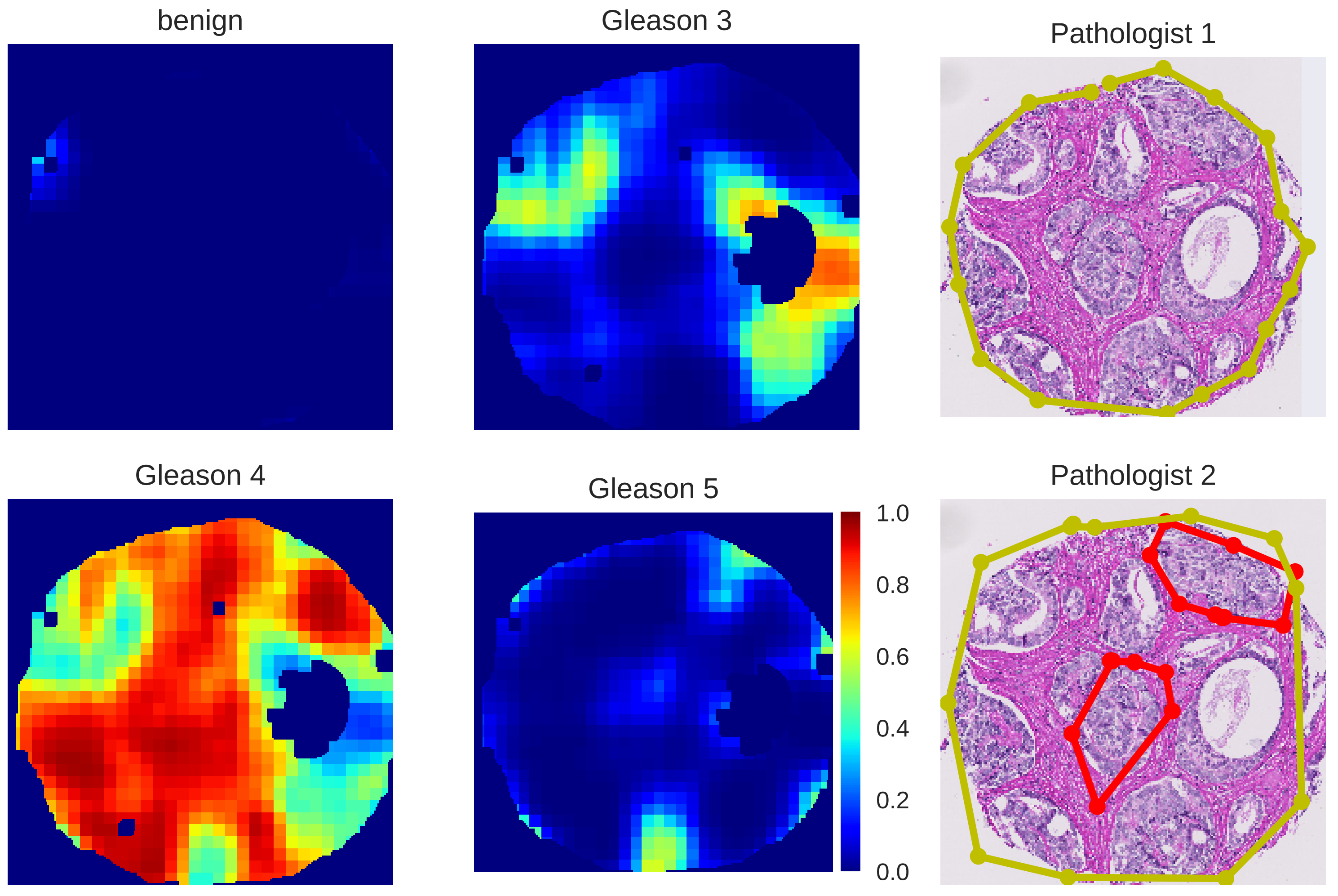
benign
Gleason 3
Pathologist 1
Gleason 4
Gleason 5
Pathologist 2
1.0
0.8
0.6
0.4
0.2
0.0

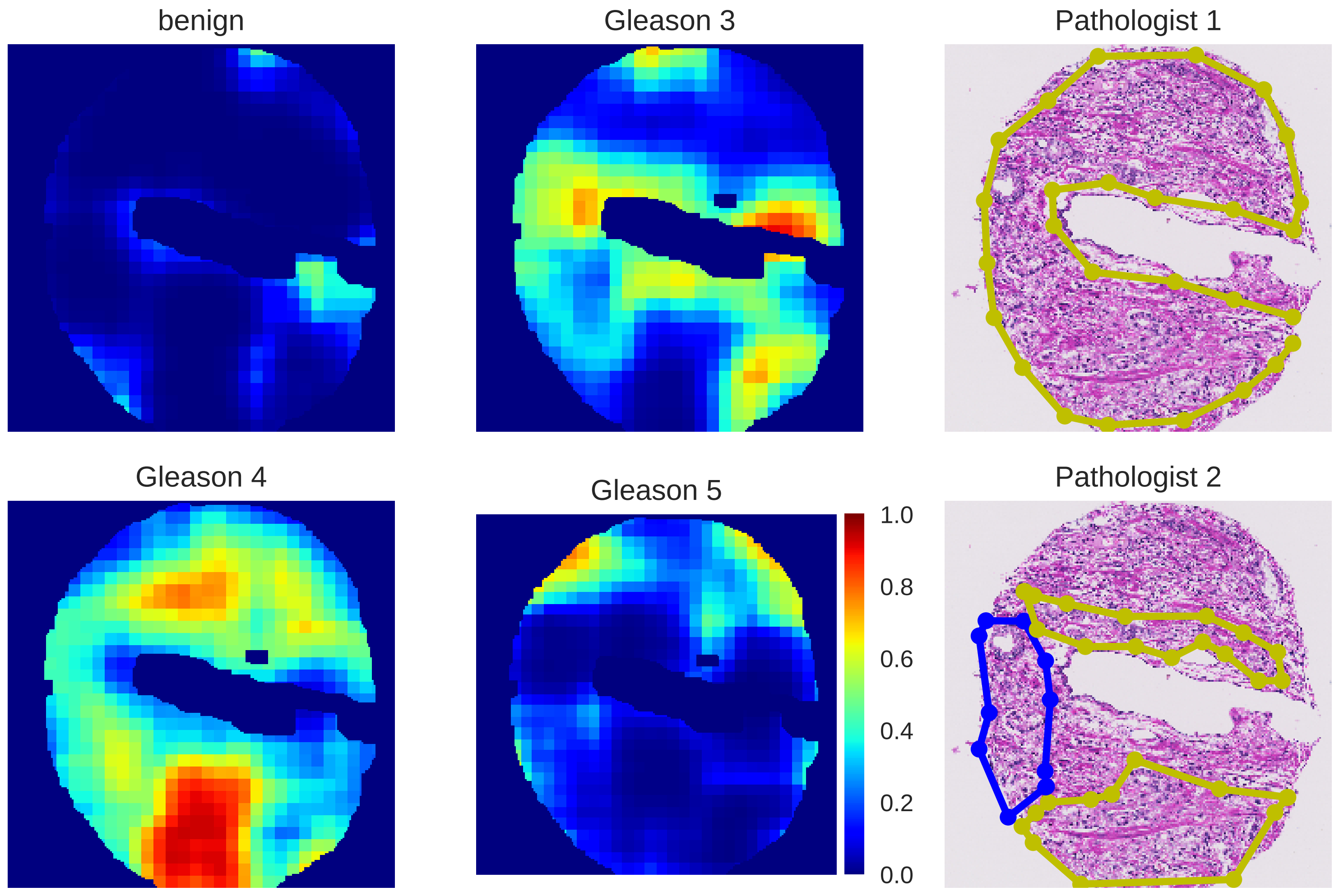
benign
Gleason 3
Pathologist 1
Gleason 4
Gleason 5
Pathologist 2
1.0
0.8
0.6
0.4
0.2
0.0

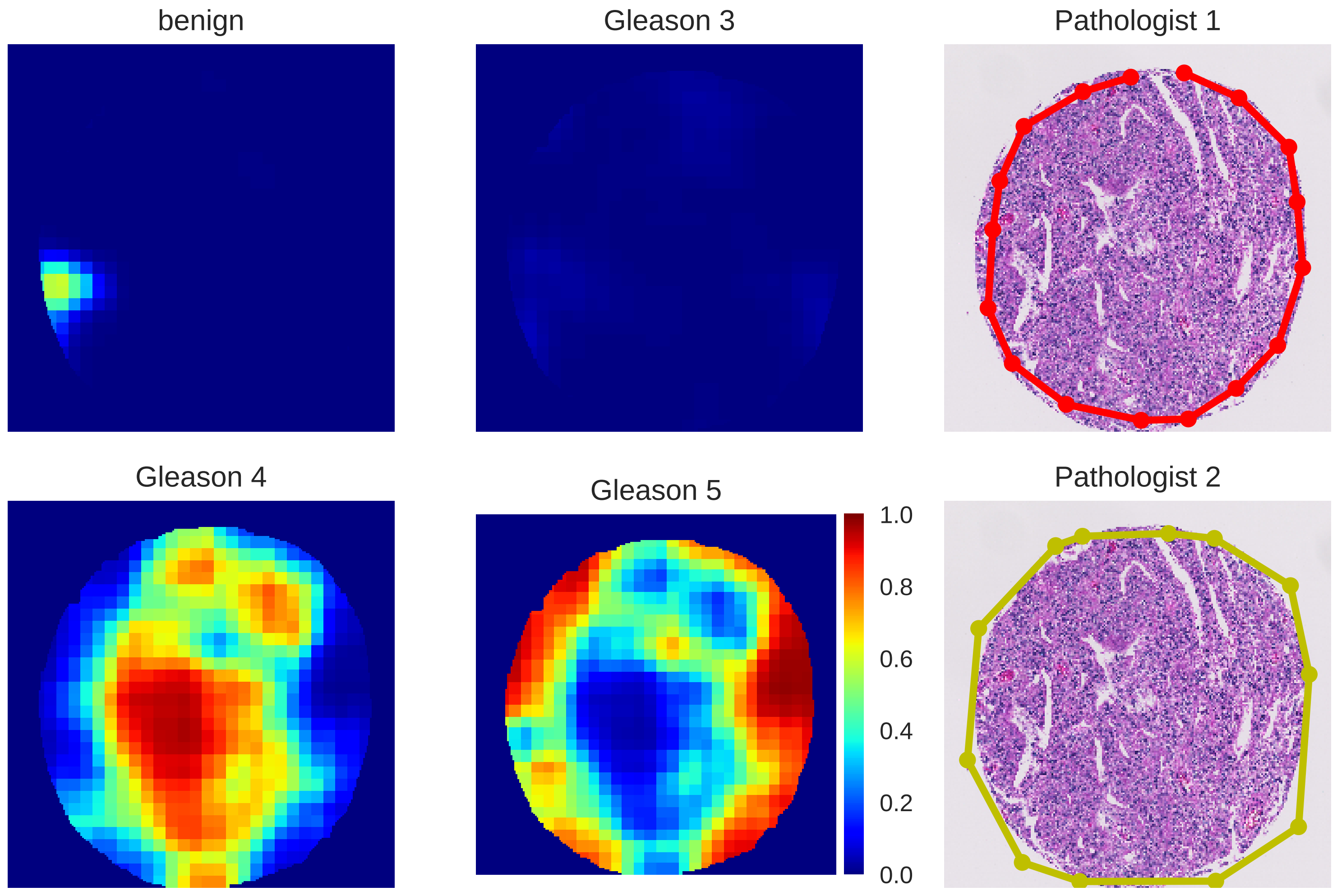
benign
Gleason 3
Pathologist 1
Gleason 4
Gleason 5
Pathologist 2
1.0
0.8
0.6
0.4
0.2
0.0

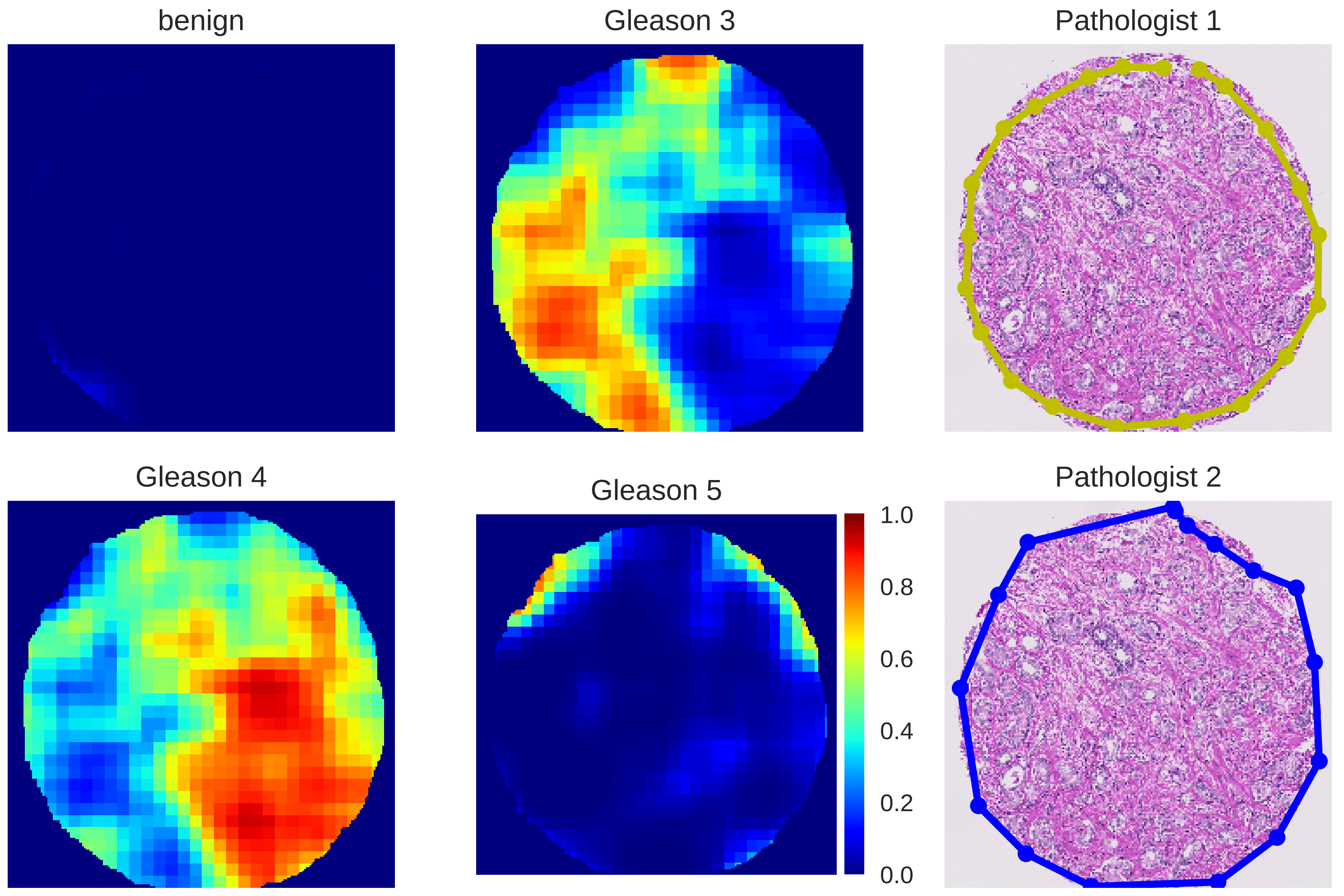
benign
Gleason 3
Pathologist 1
Gleason 4
Gleason 5
Pathologist 2
1.0
0.8
0.6
0.4
0.2
0.0

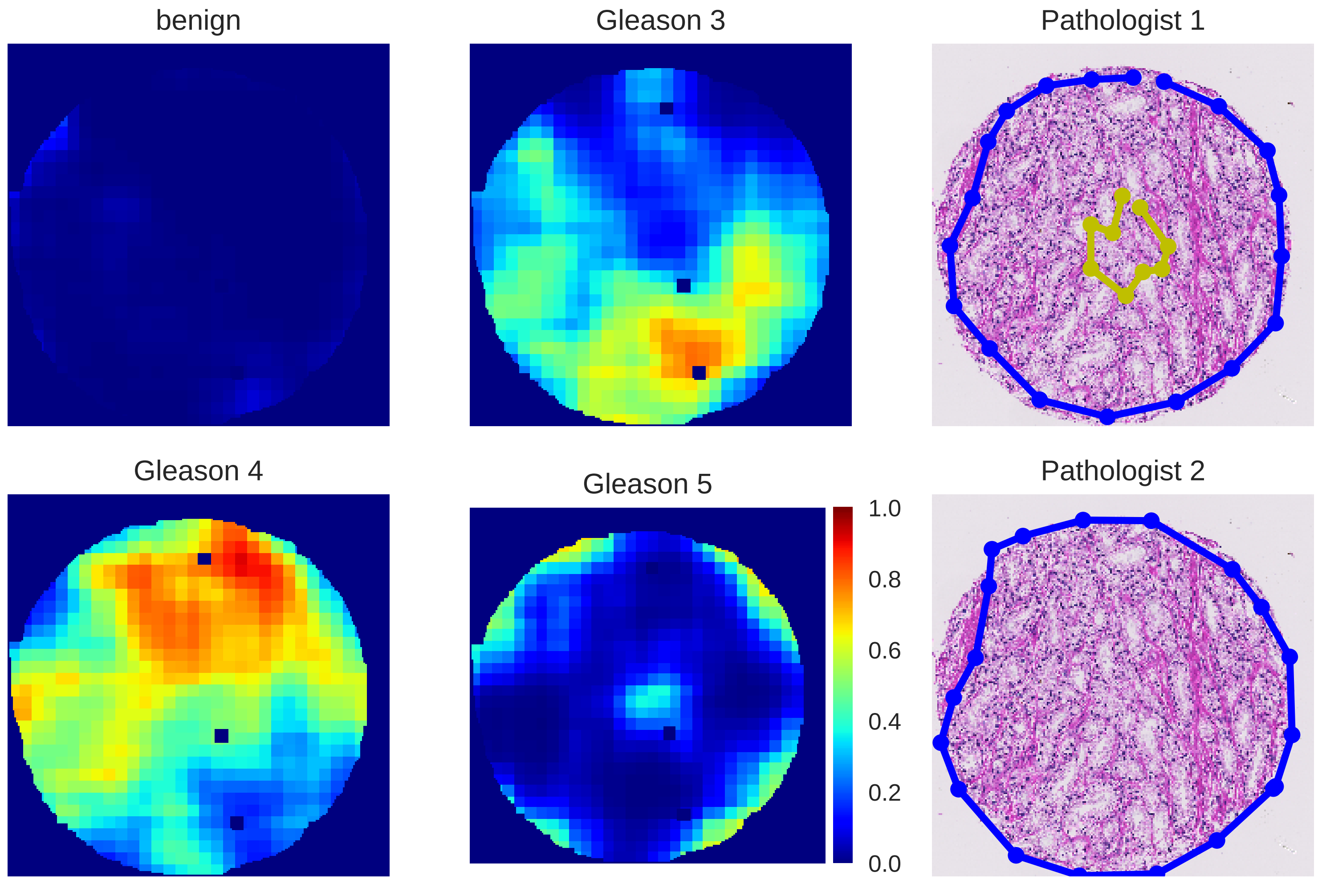
benign
Gleason 3
Pathologist 1
Gleason 4
Gleason 5
Pathologist 2
1.0
0.8
0.6
0.4
0.2
0.0

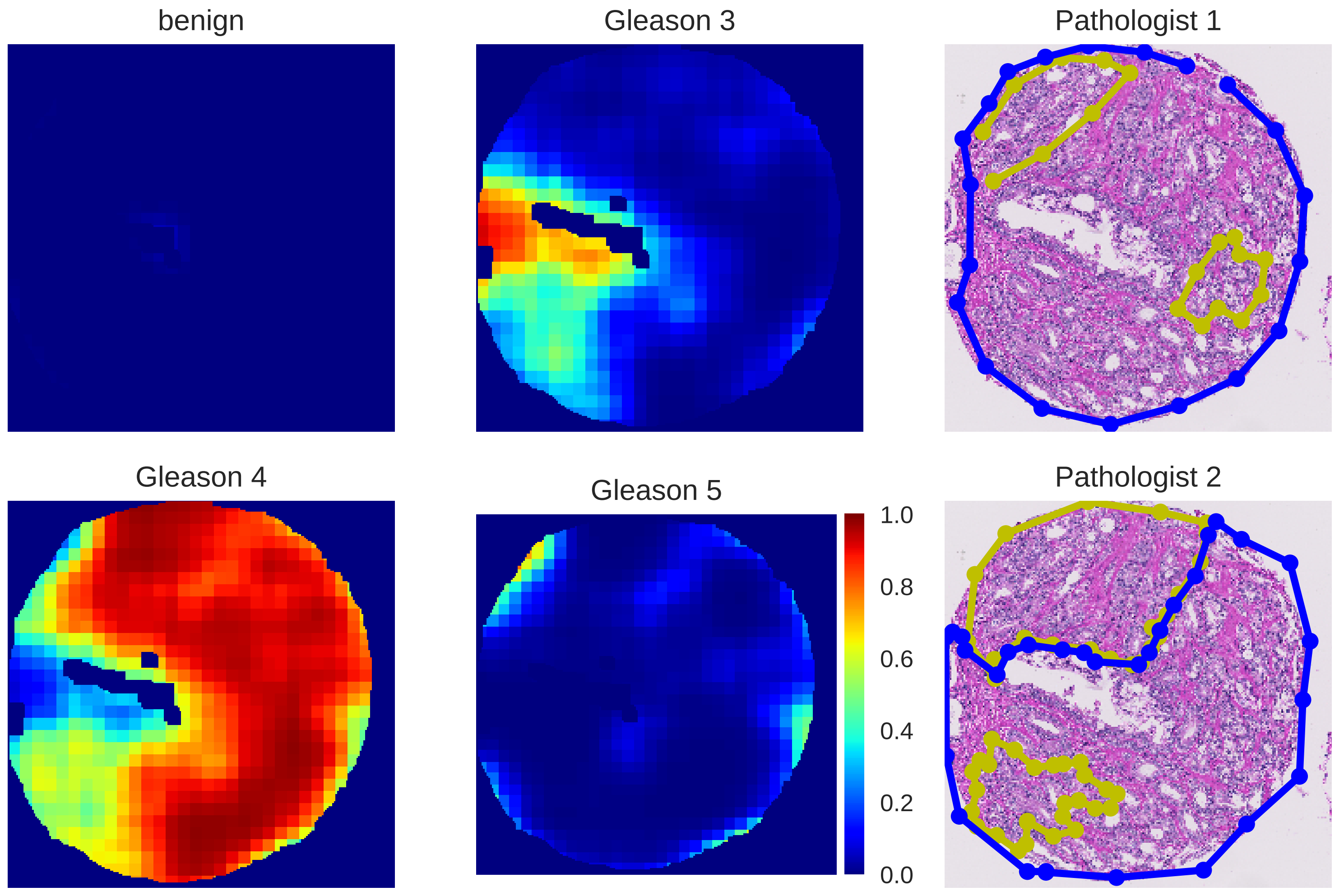
benign
Gleason 3
Pathologist 1
Gleason 4
Gleason 5
Pathologist 2
1.0
0.8
0.6
0.4
0.2
0.0

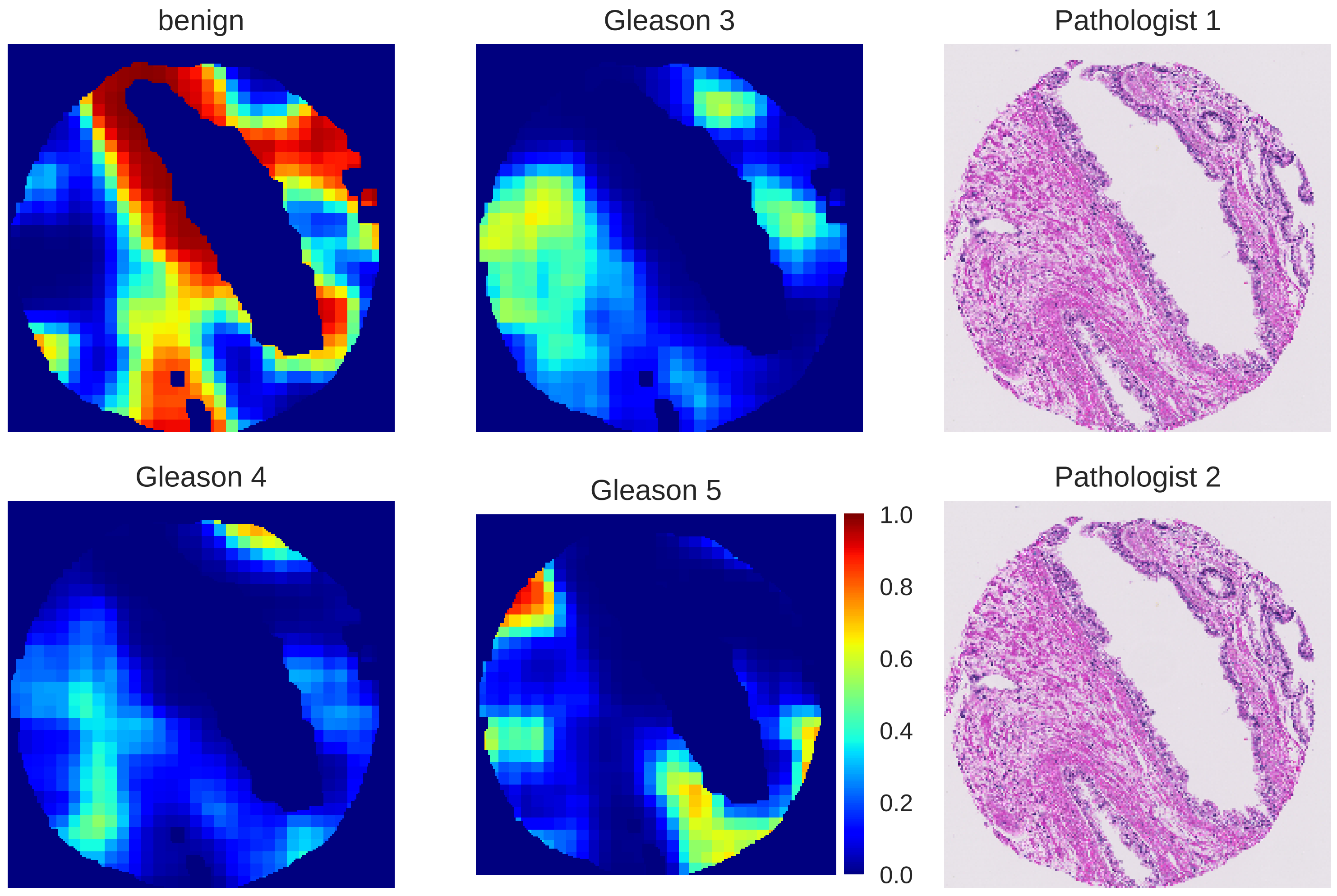
benign
Gleason 3
Pathologist 1
Gleason 4
Gleason 5
Pathologist 2
1.0
0.8
0.6
0.4
0.2
0.0

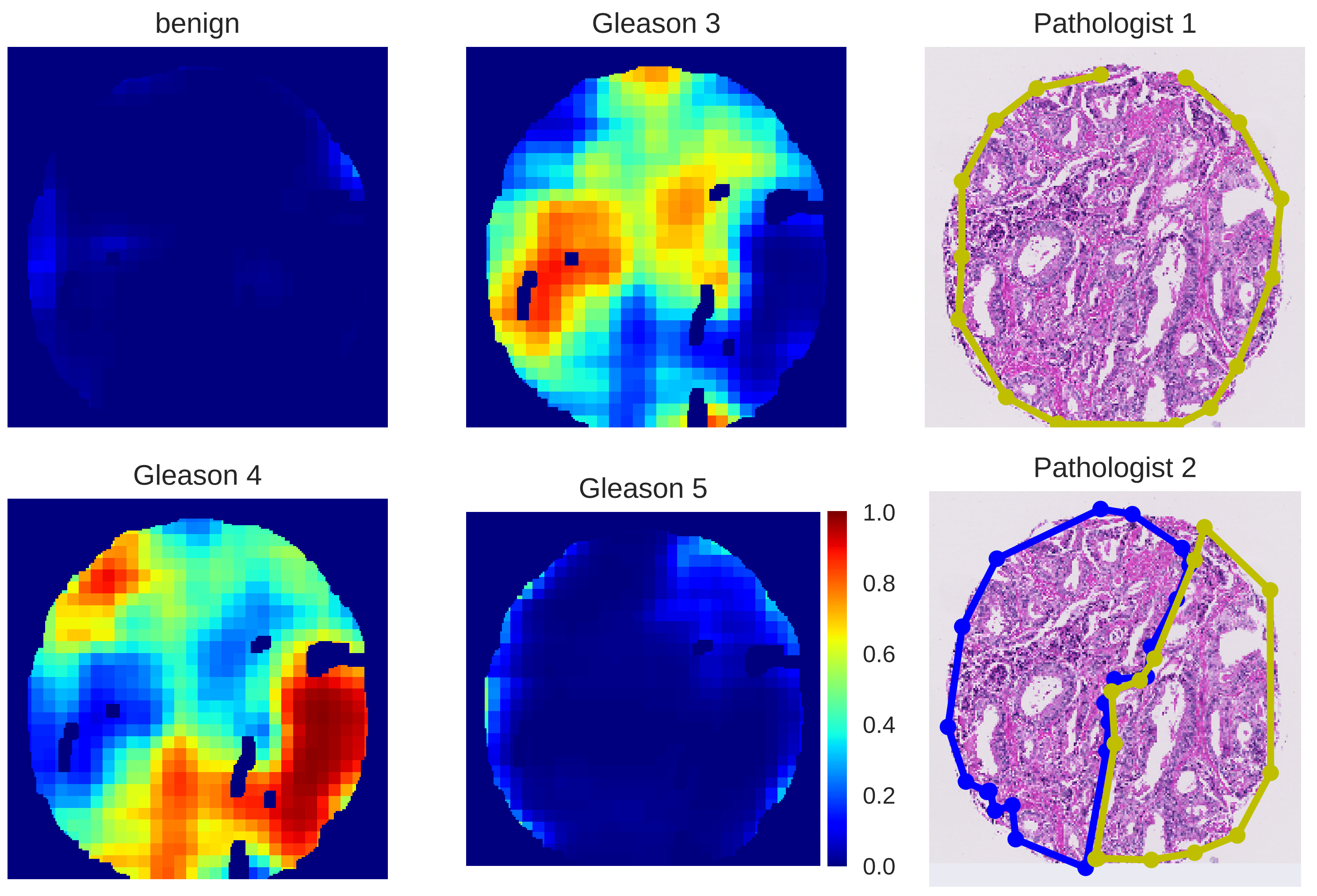
benign
Gleason 3
Pathologist 1
Gleason 4
Gleason 5
Pathologist 2
1.0
0.8
0.6
0.4
0.2
0.0

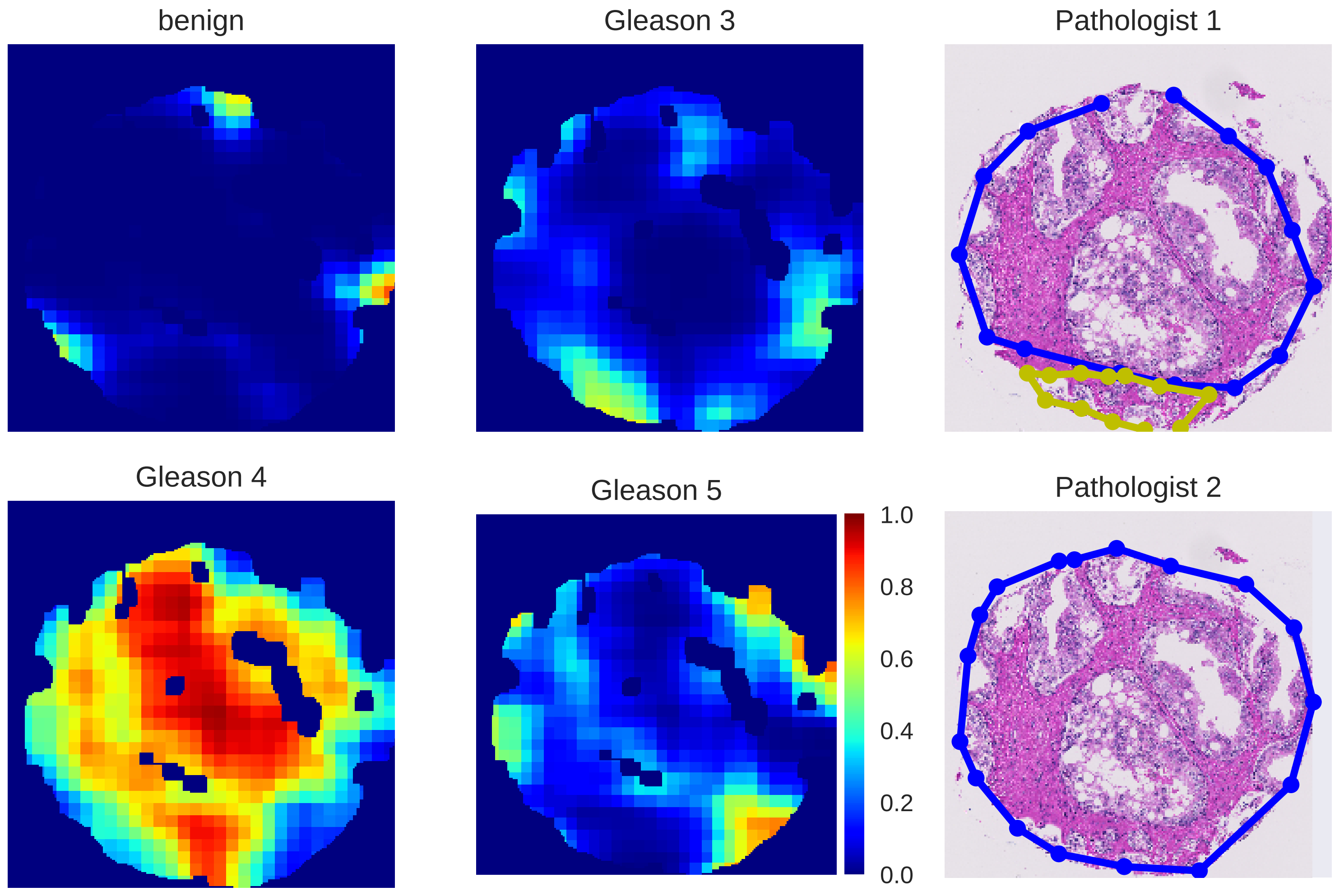
benign
Gleason 3
Pathologist 1
Gleason 4
Gleason 5
Pathologist 2
1.0
0.8
0.6
0.4
0.2
0.0

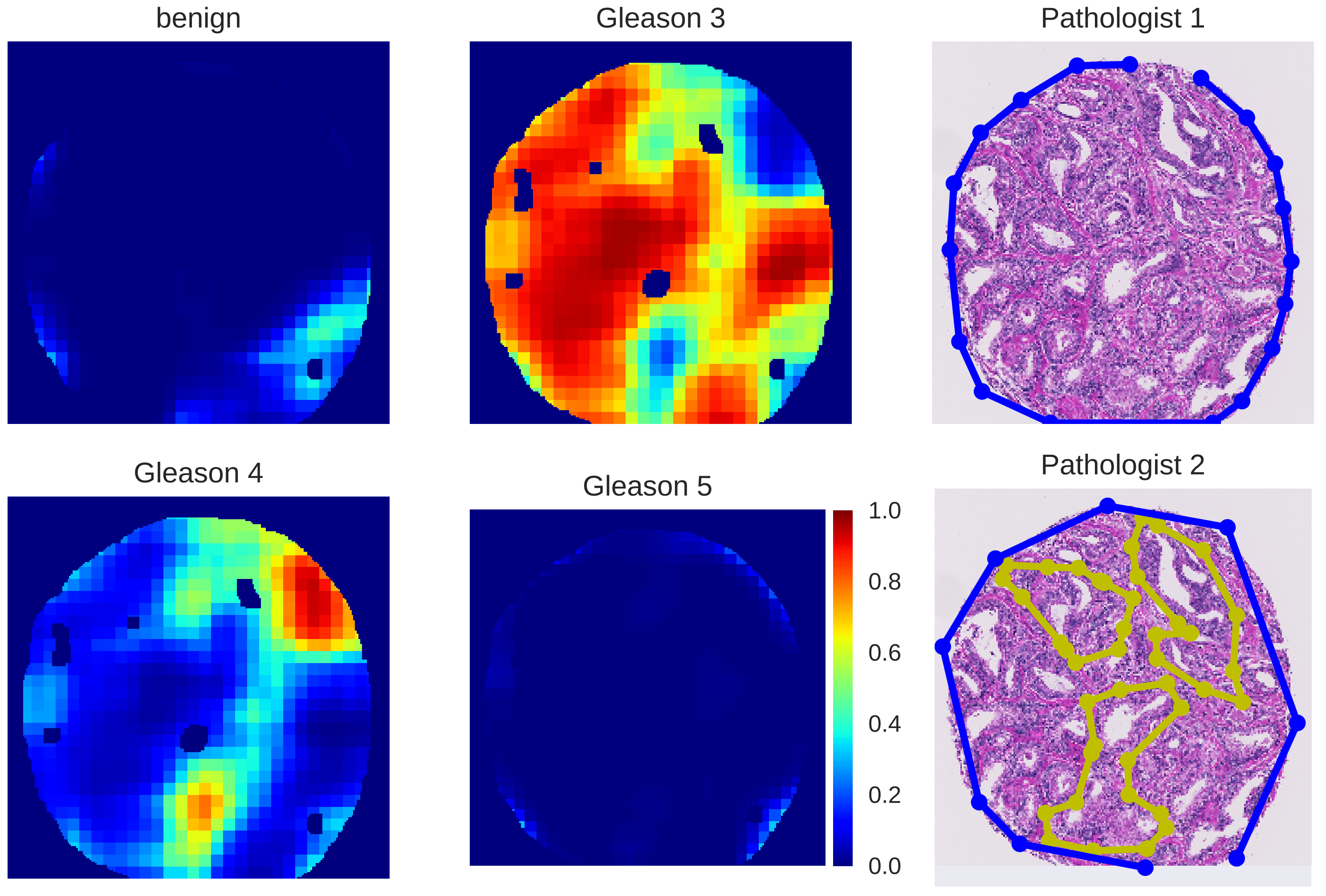
benign
Gleason 3
Pathologist 1
Gleason 4
Gleason 5
Pathologist 2
1.0
0.8
0.6
0.4
0.2
0.0

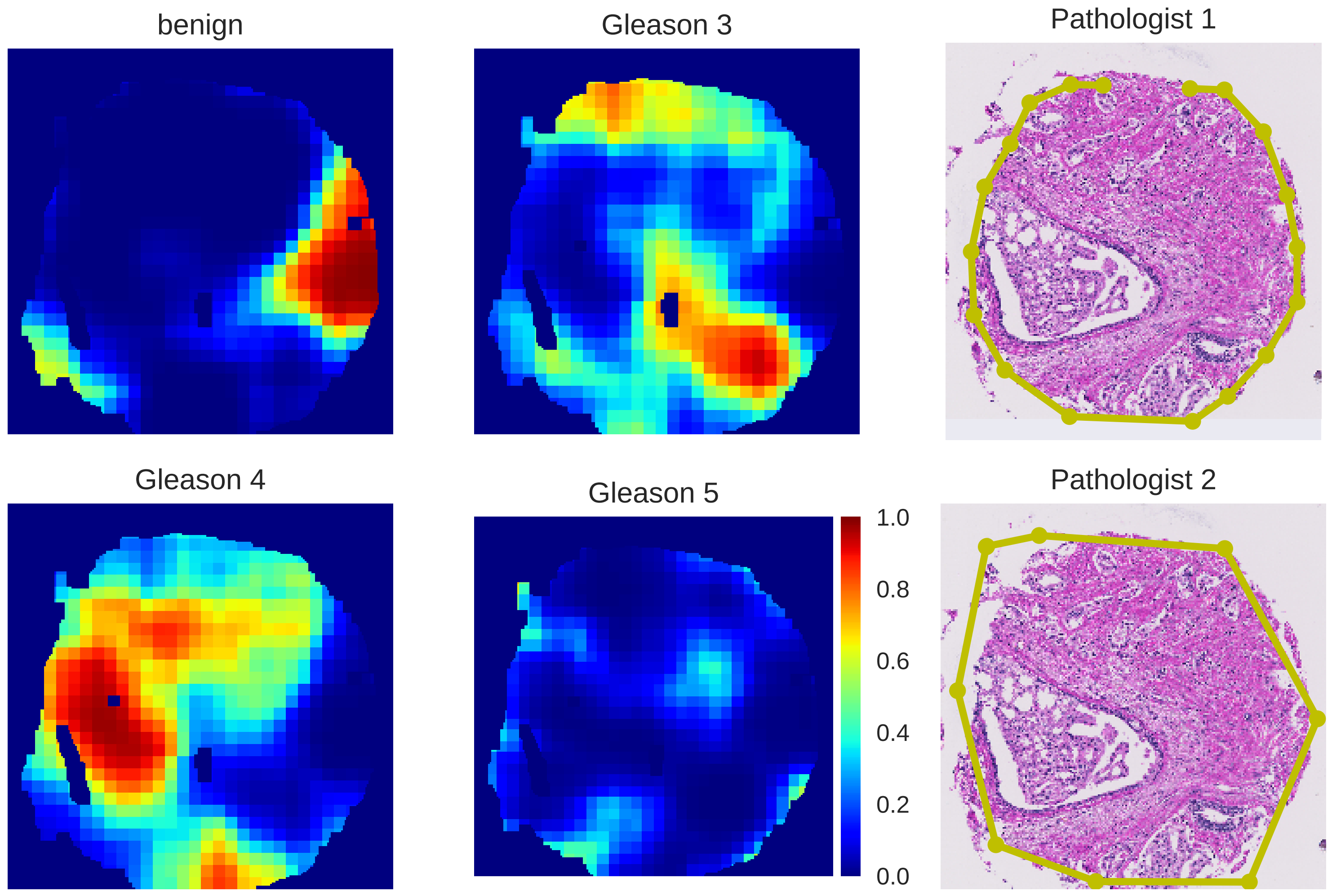
benign
Gleason 3
Pathologist 1
Gleason 4
Gleason 5
Pathologist 2
1.0
0.8
0.6
0.4
0.2
0.0

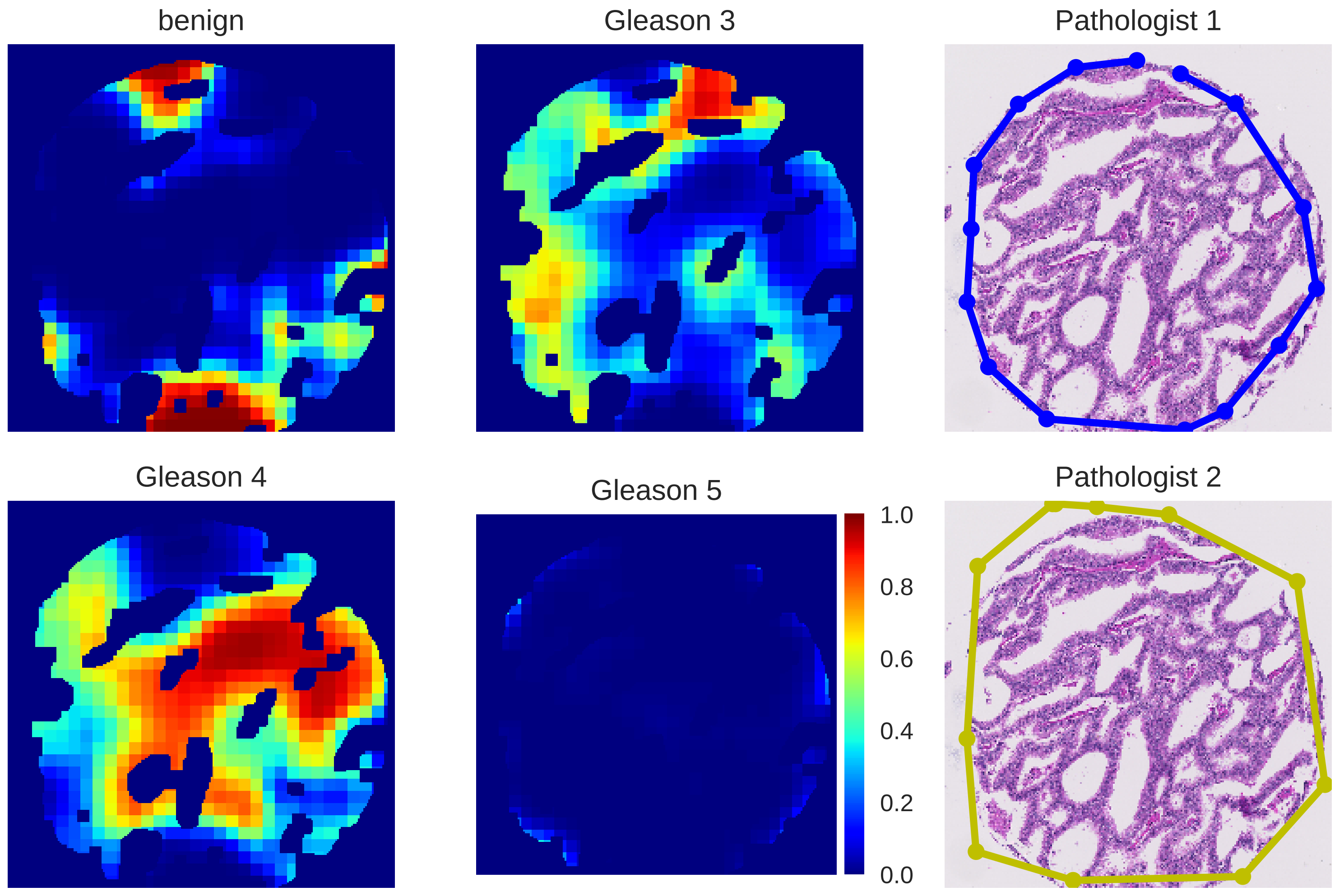

benign
Gleason 3
Pathologist 1
Gleason 4
Gleason 5
Pathologist 2
1.0
0.8
0.6
0.4
0.2
0.0

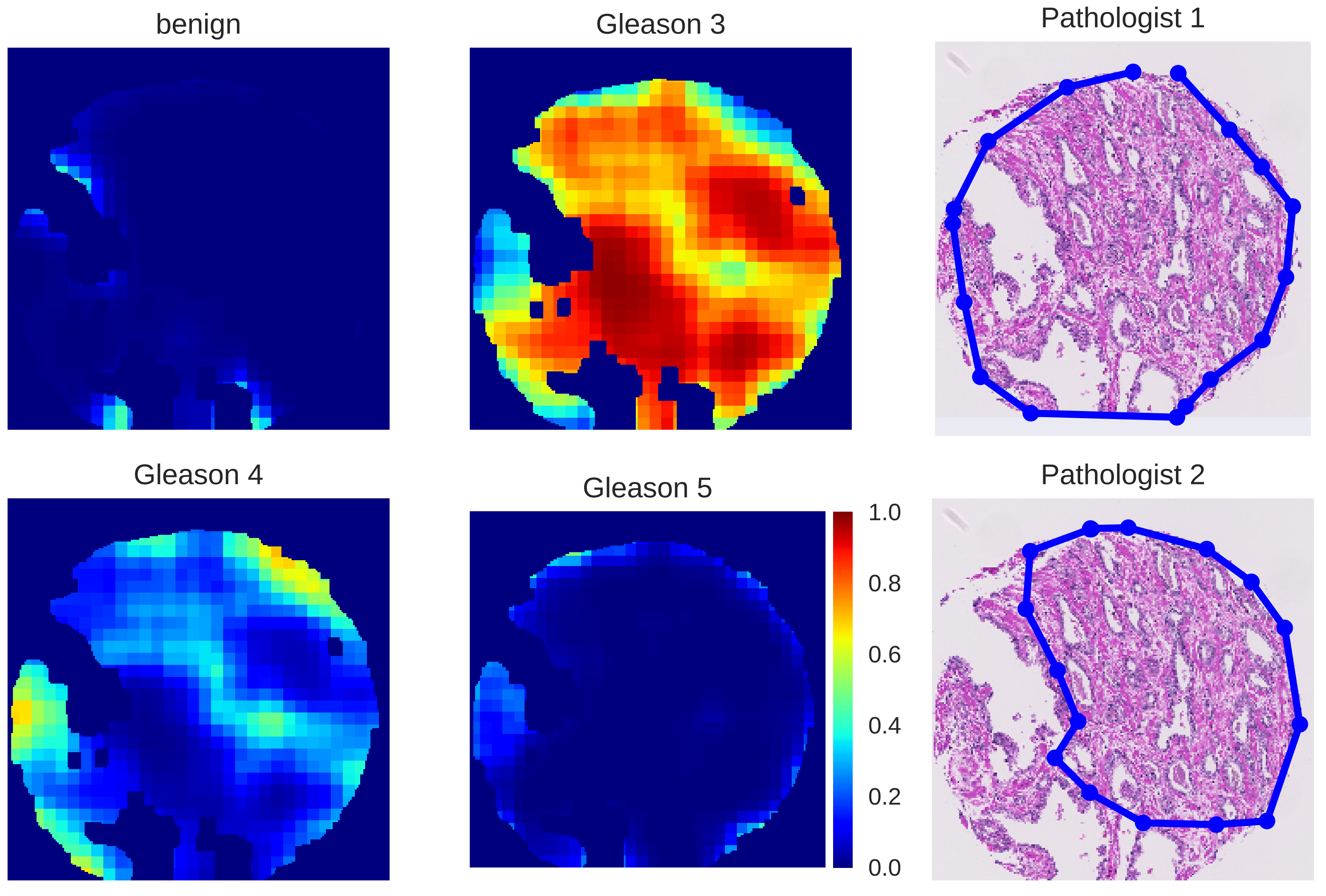
benign
Gleason 3
Pathologist 1
Gleason 4
Gleason 5
Pathologist 2
1.0
0.8
0.6
0.4
0.2
0.0

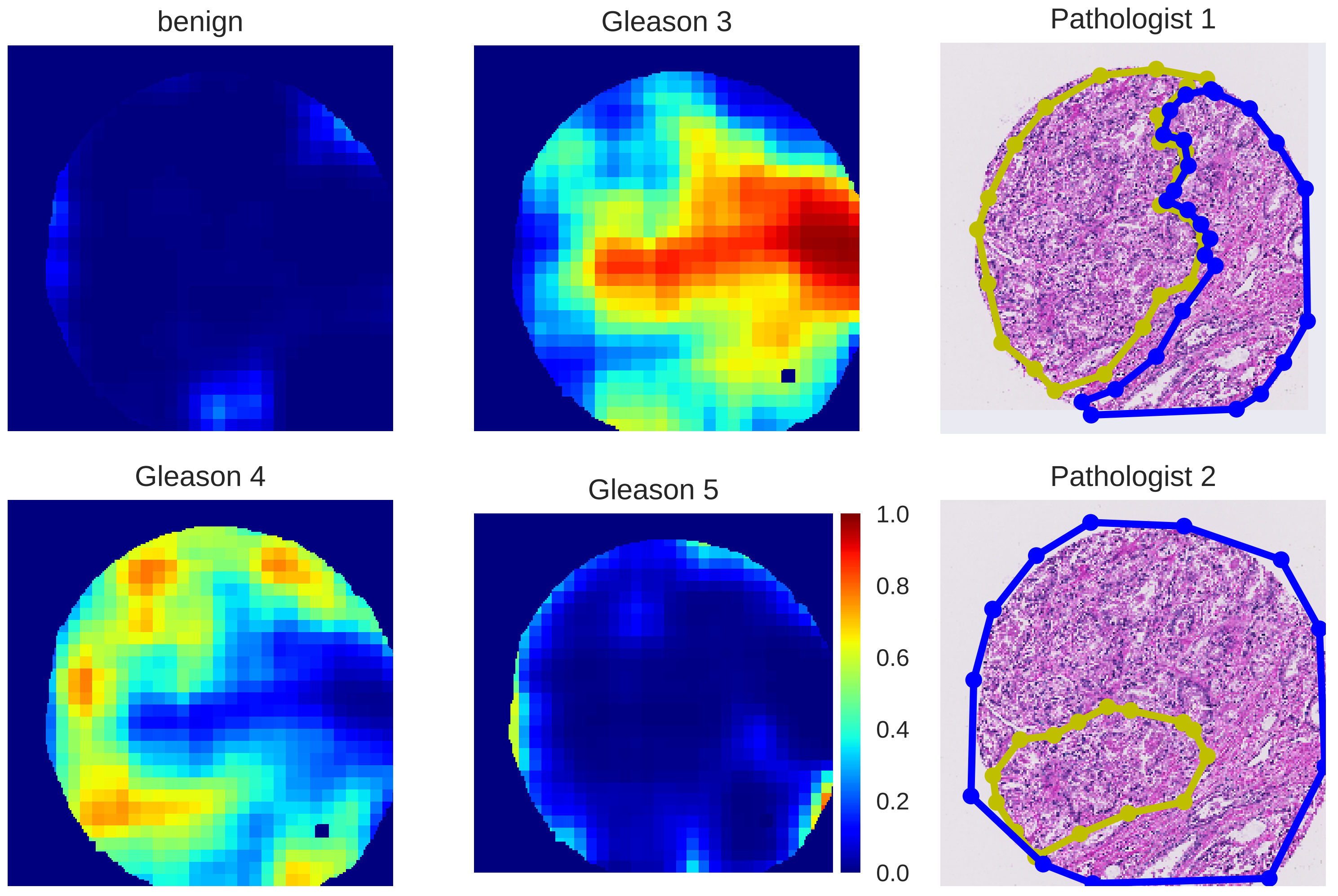

benign
Gleason 3
Pathologist 1
Gleason 4
Gleason 5
Pathologist 2
1.0
0.8
0.6
0.4
0.2
0.0

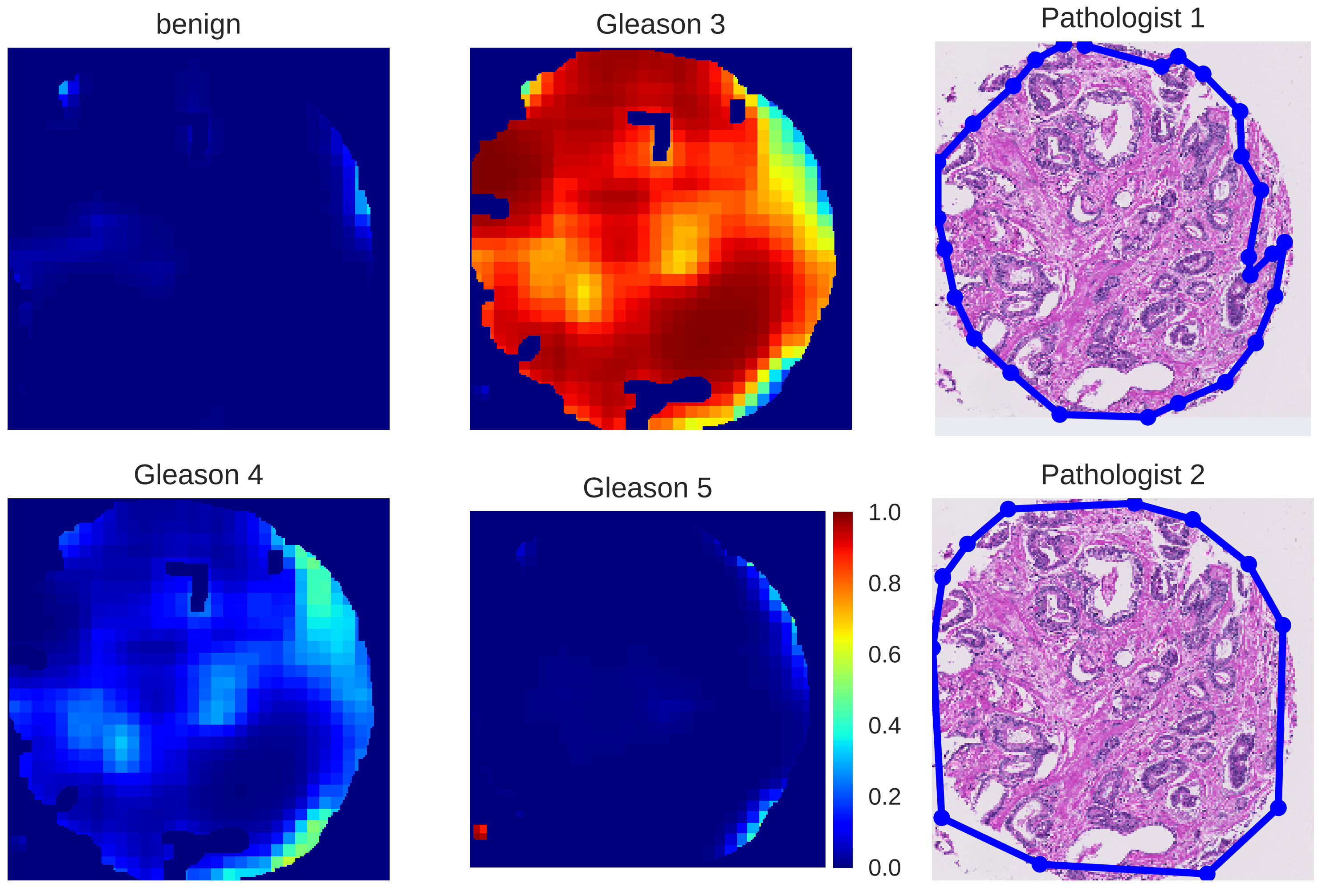

benign
Gleason 3
Pathologist 1
Gleason 4
Gleason 5
Pathologist 2
1.0
0.8
0.6
0.4
0.2
0.0

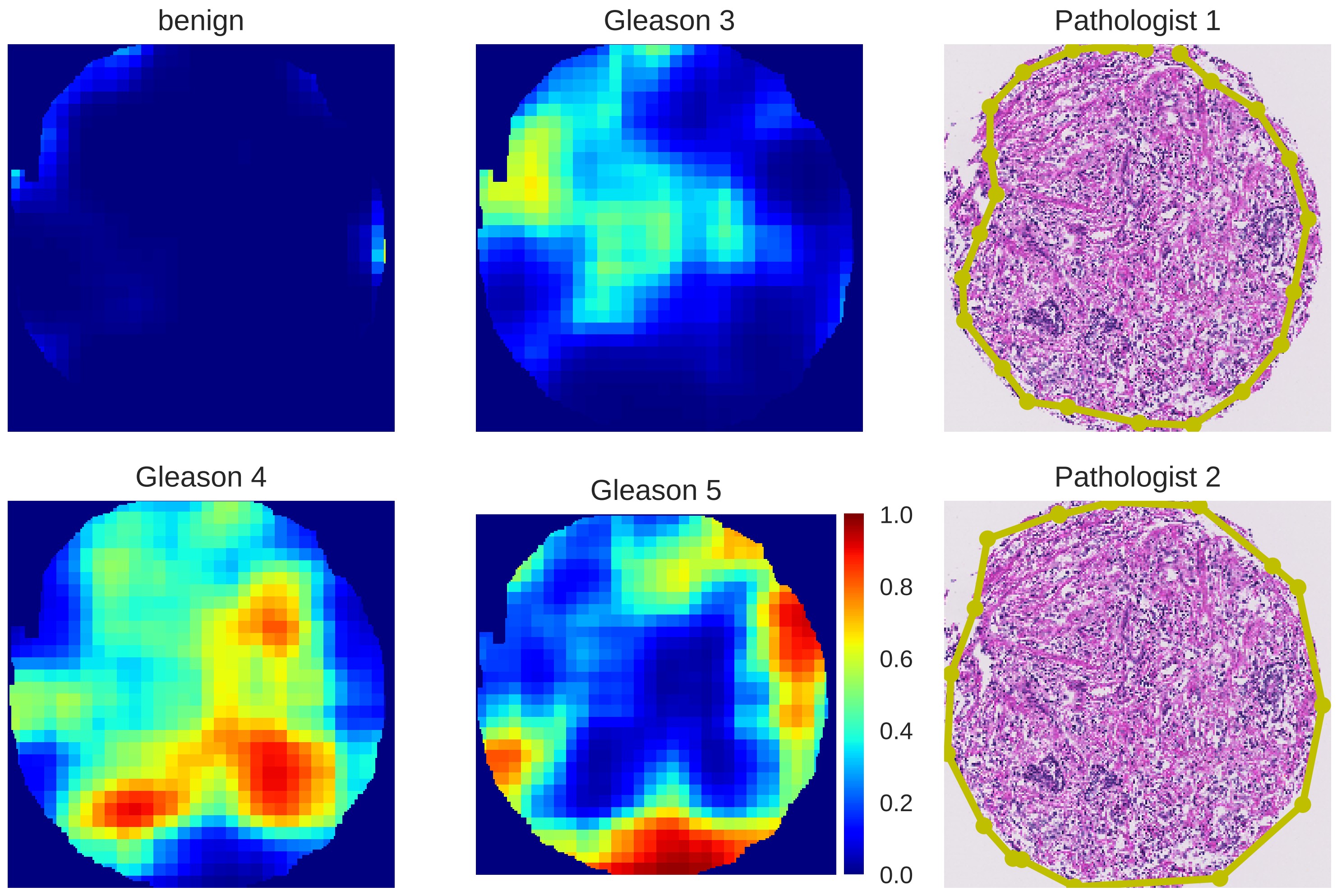
benign
Gleason 3
Pathologist 1
Gleason 4
Gleason 5
Pathologist 2
1.0
0.8
0.6
0.4
0.2
0.0

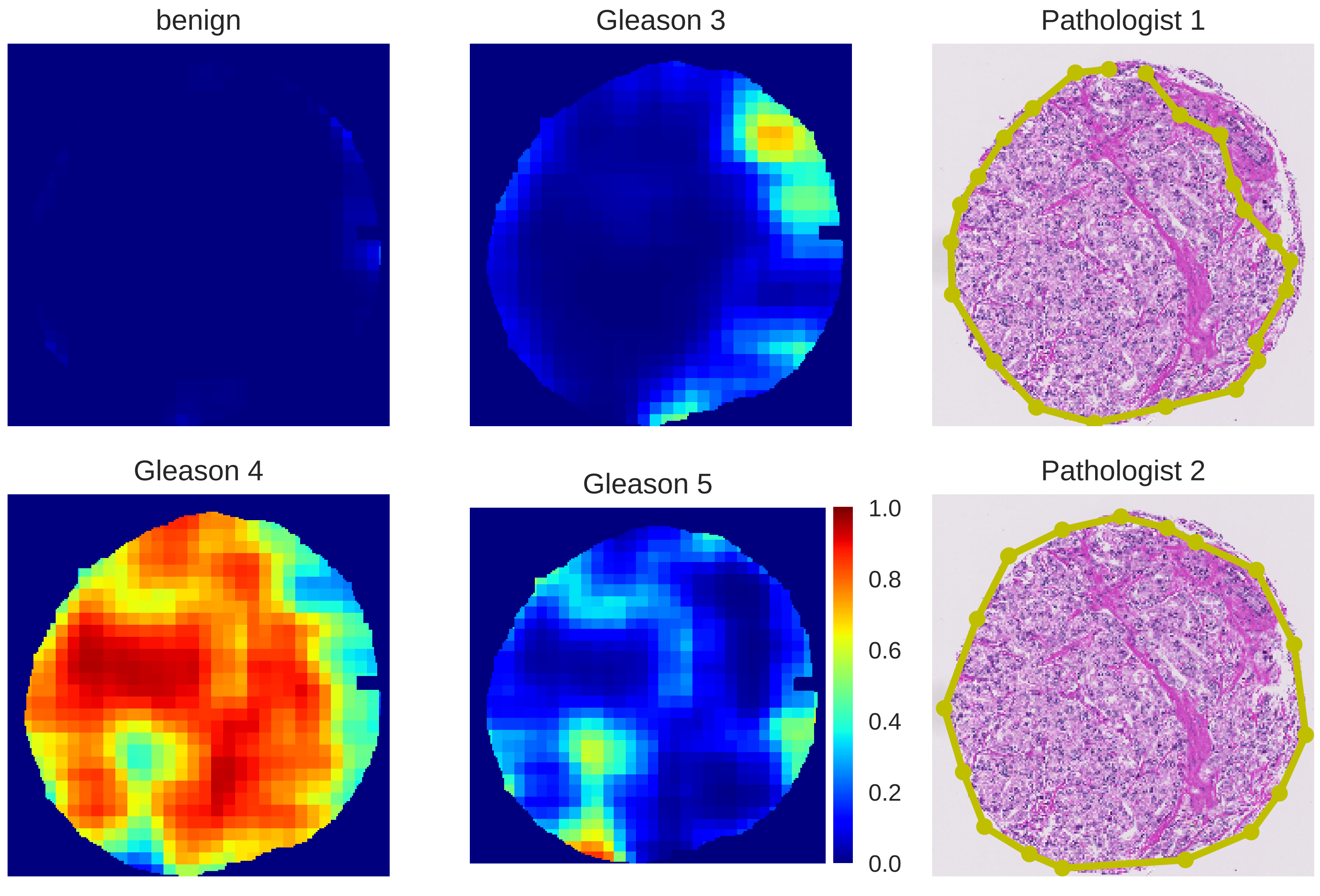
benign
Gleason 3
Pathologist 1
Gleason 4
Gleason 5
Pathologist 2
1.0
0.8
0.6
0.4
0.2
0.0

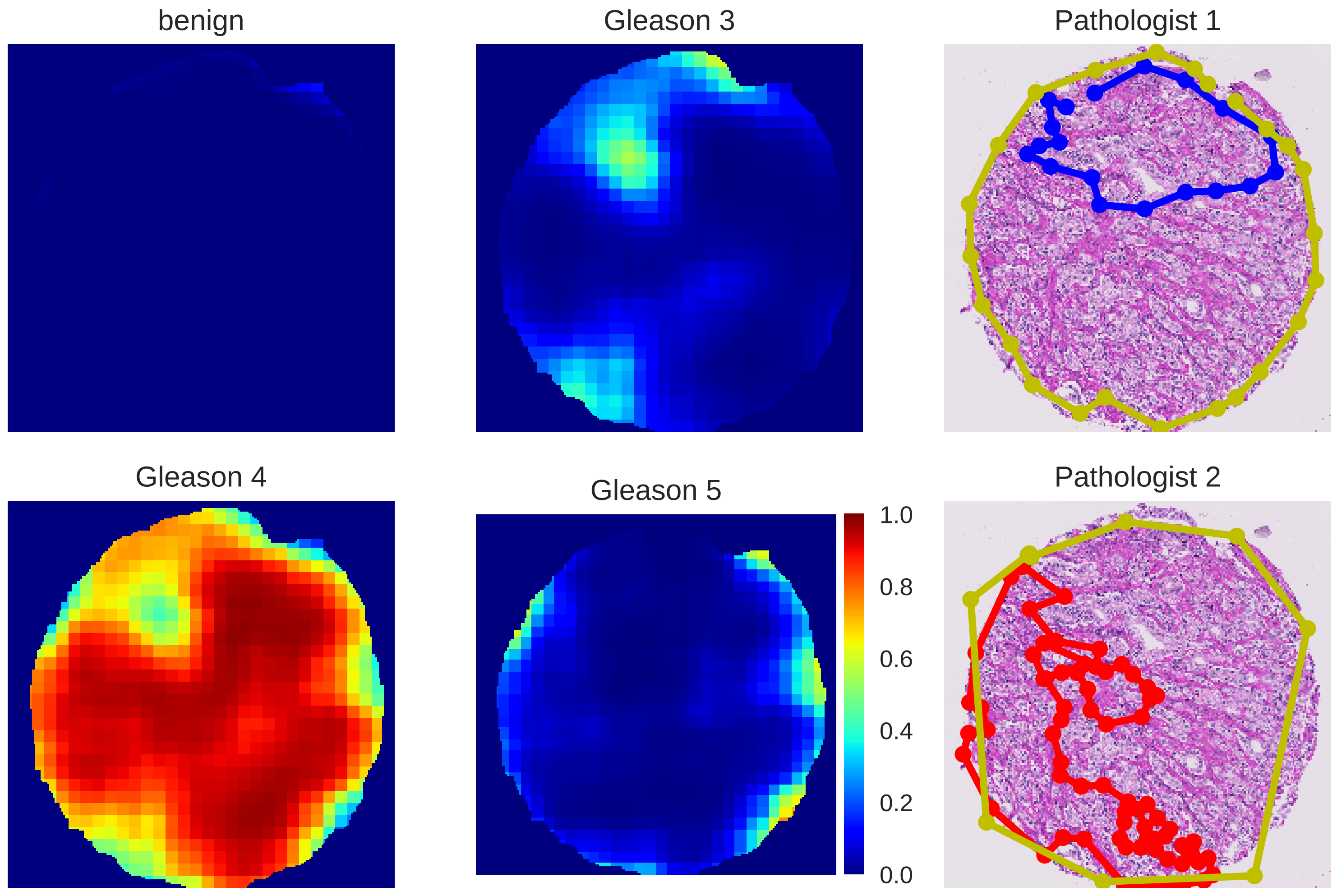
benign
Gleason 3
Pathologist 1
Gleason 4
Gleason 5
Pathologist 2
1.0
0.8
0.6
0.4
0.2
0.0

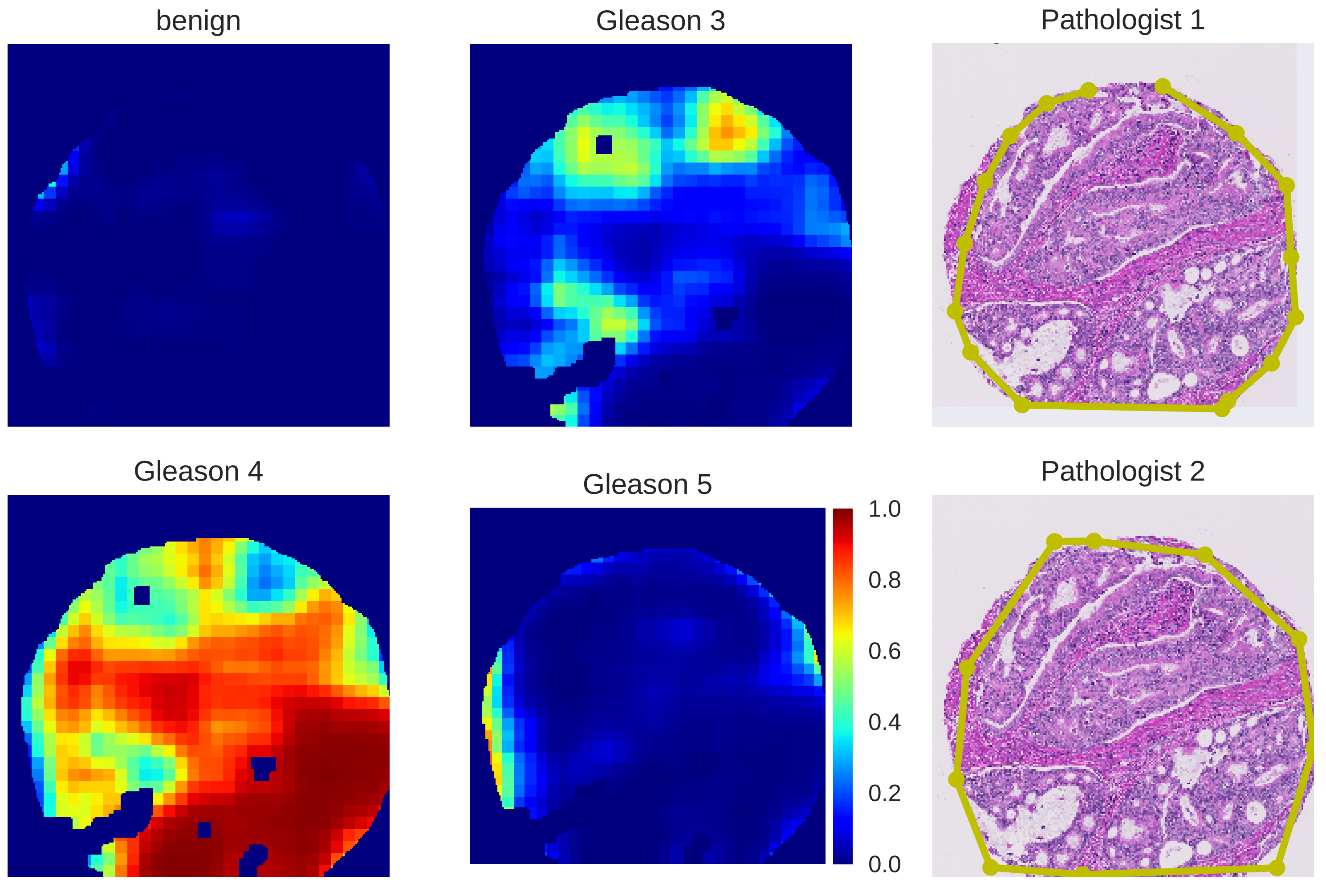

benign
Gleason 3
Pathologist 1
Gleason 4
Gleason 5
Pathologist 2
1.0
0.8
0.6
0.4
0.2
0.0

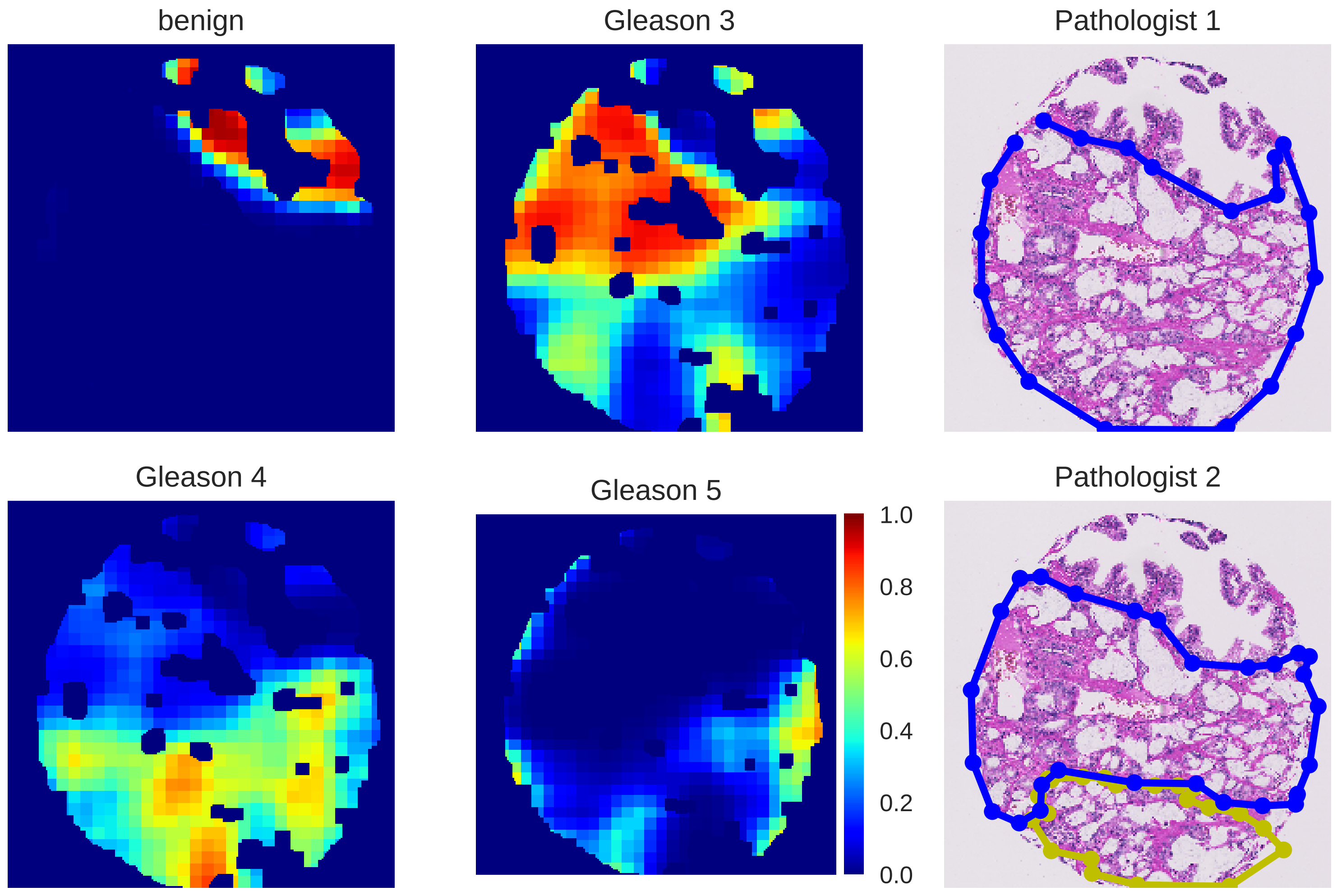
benign
Gleason 3
Pathologist 1
Gleason 4
Gleason 5
Pathologist 2
1.0
0.8
0.6
0.4
0.2
0.0

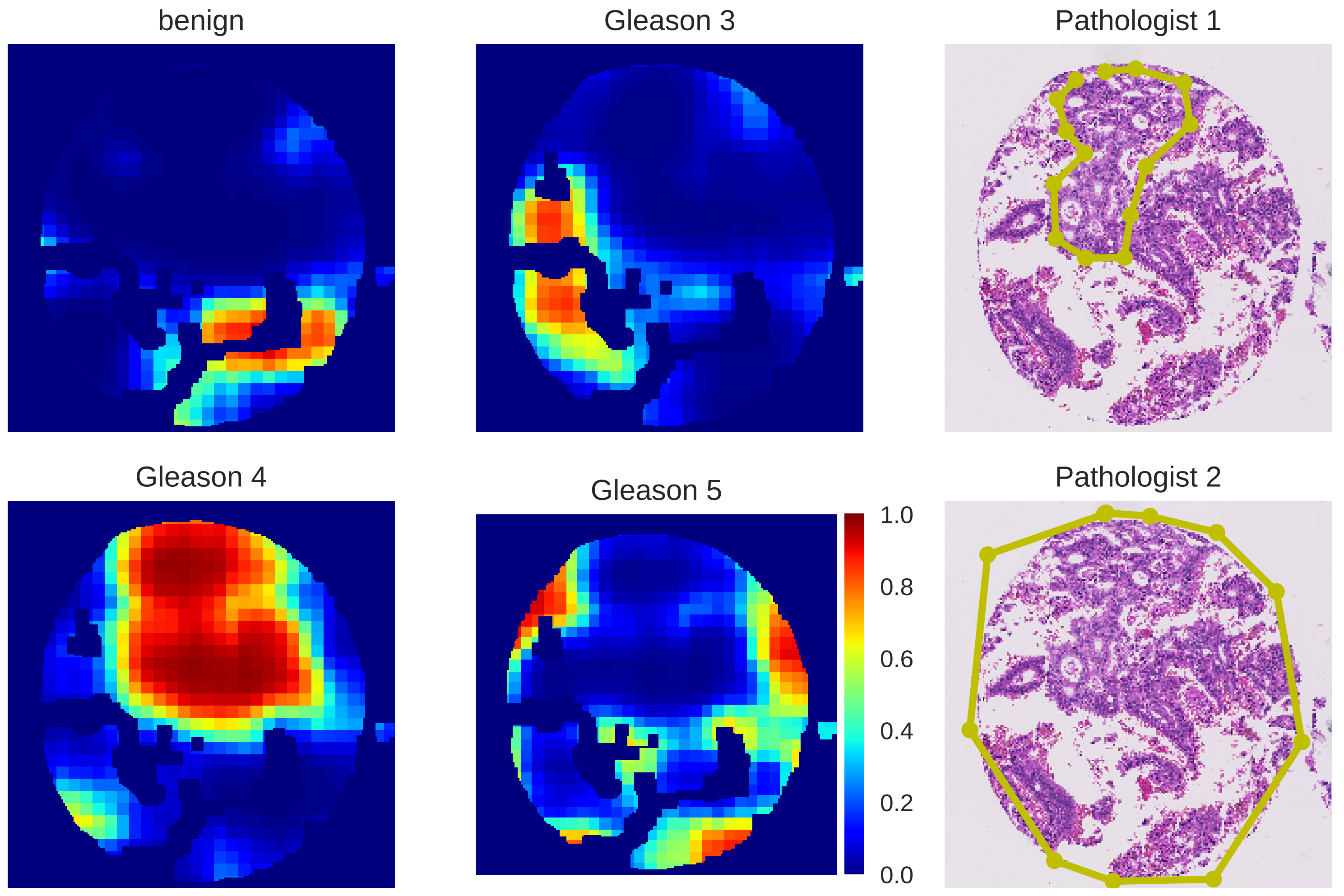
benign
Gleason 3
Pathologist 1
Gleason 4
Gleason 5
Pathologist 2
1.0
0.8
0.6
0.4
0.2
0.0

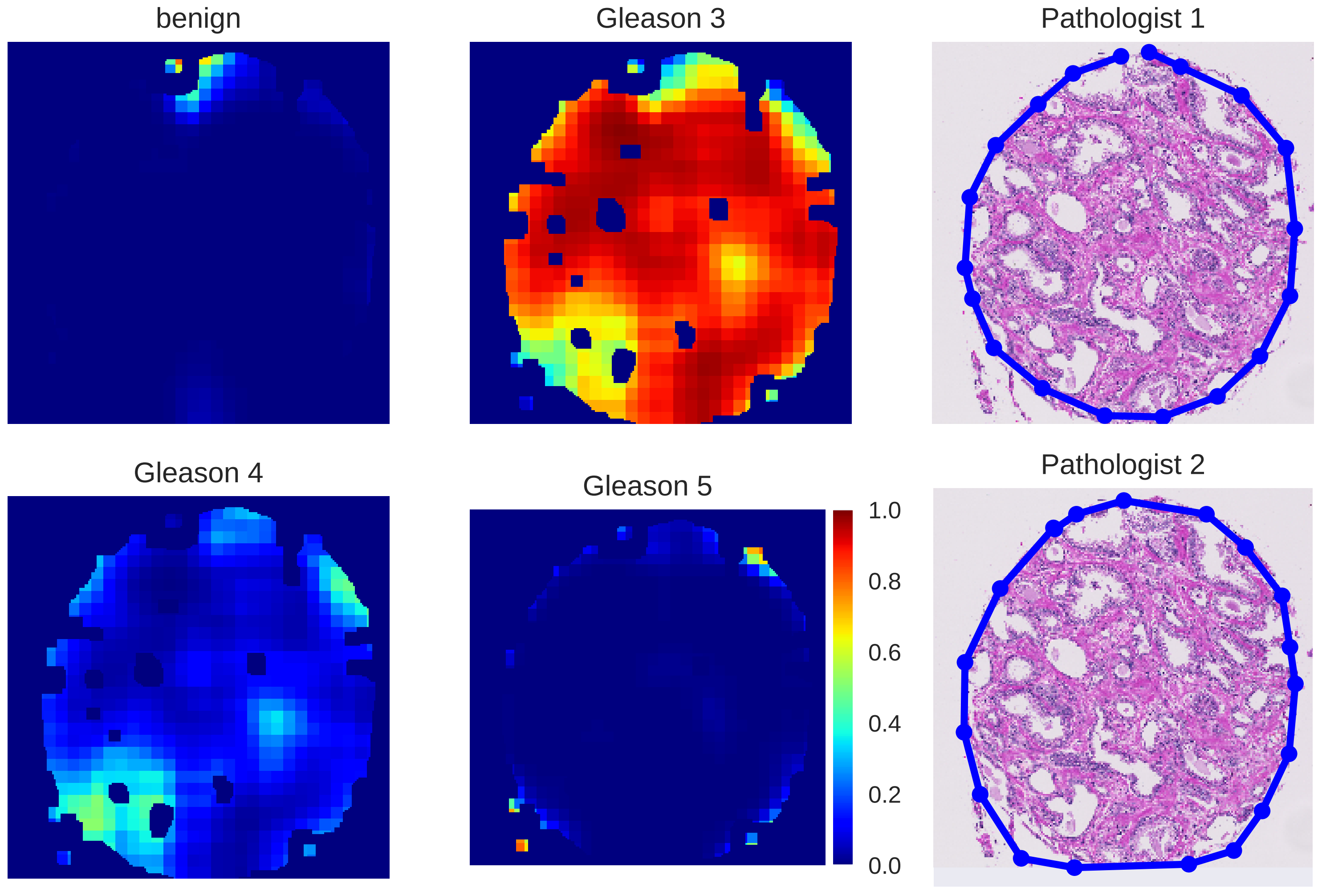

benign
Gleason 3
Pathologist 1
Gleason 4
Gleason 5
Pathologist 2
1.0
0.8
0.6
0.4
0.2
0.0

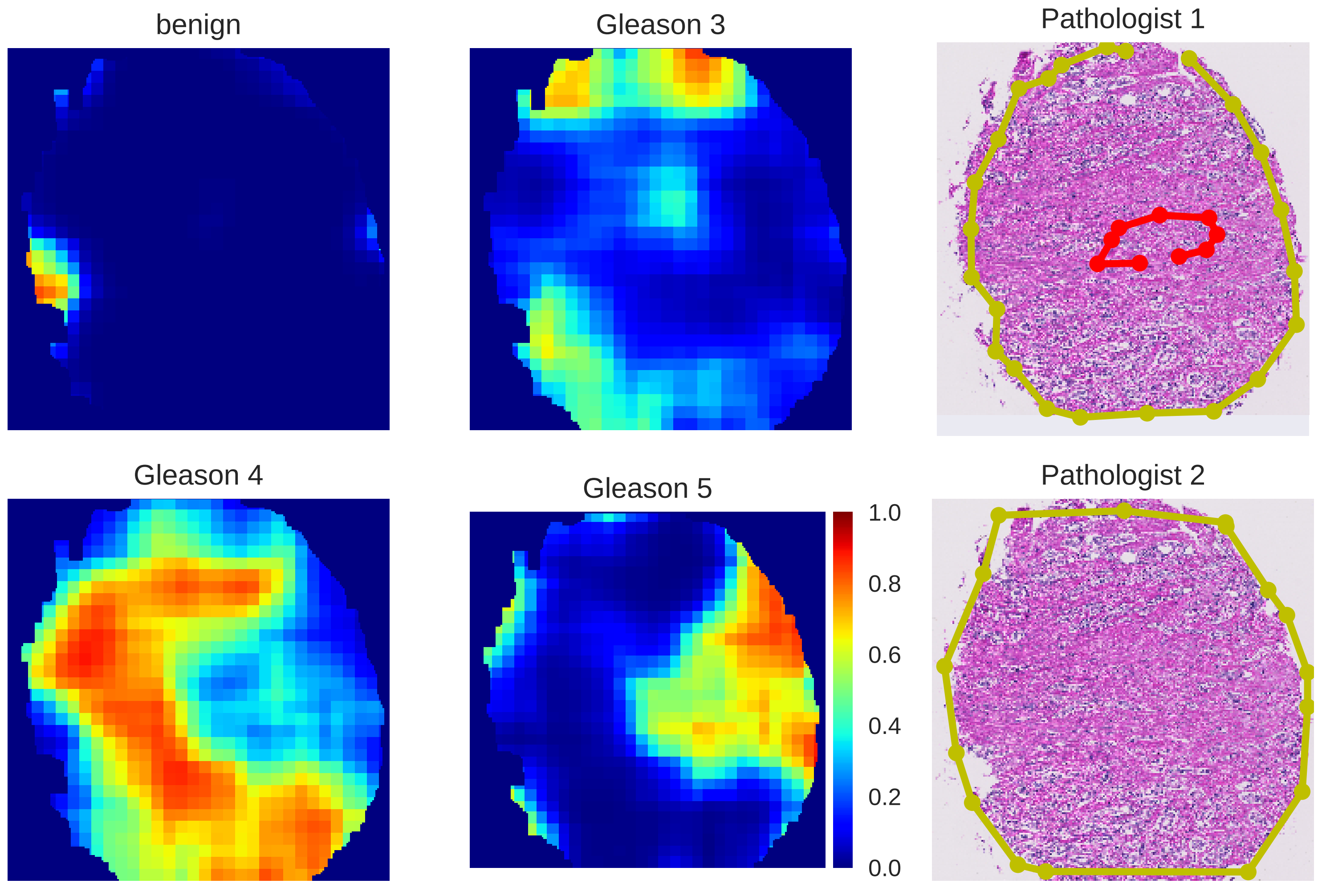

benign
Gleason 3
Pathologist 1
Gleason 4
Gleason 5
Pathologist 2
1.0
0.8
0.6
0.4
0.2
0.0

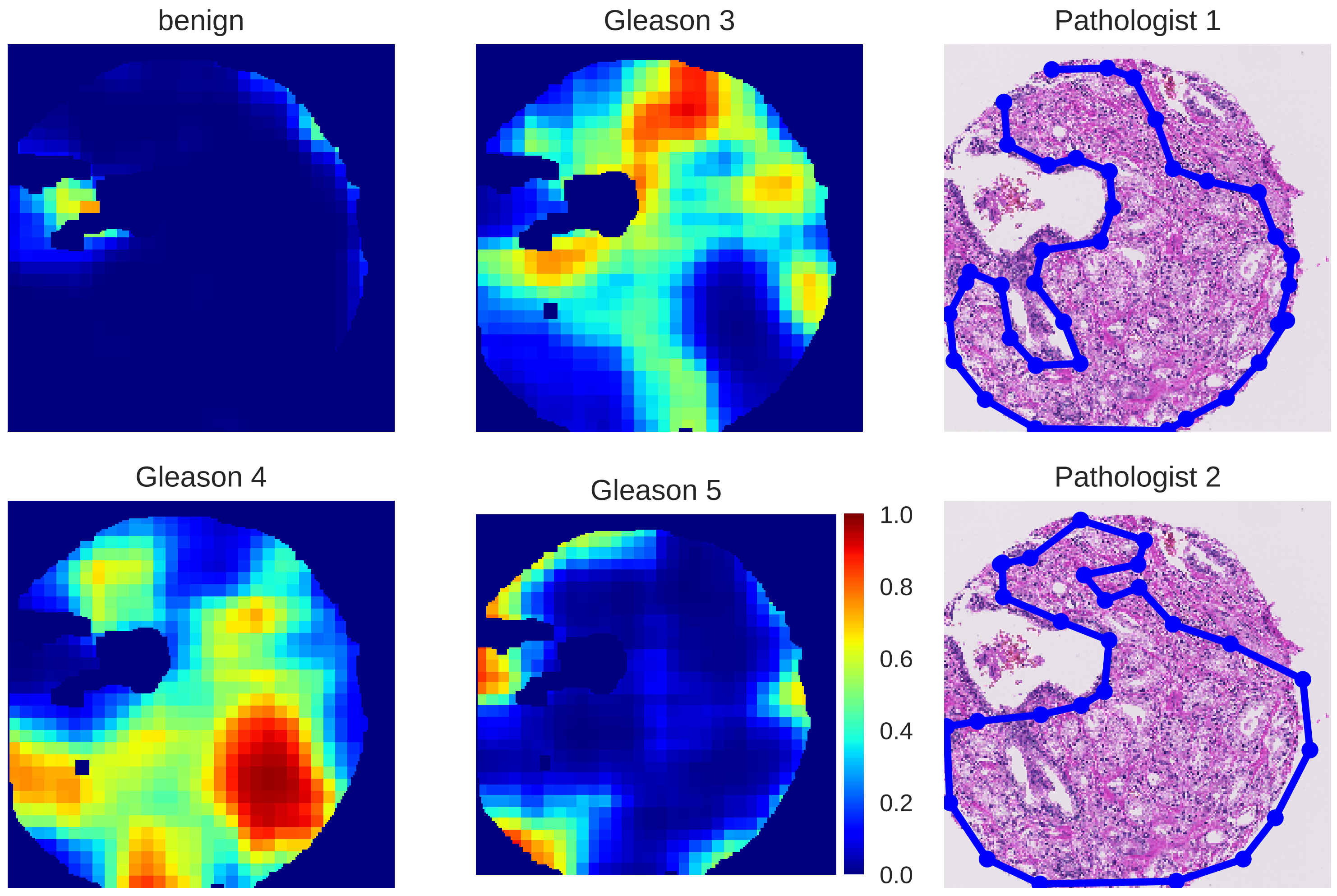
benign
Gleason 3
Pathologist 1
Gleason 4
Gleason 5
Pathologist 2
1.0
0.8
0.6
0.4
0.2
0.0

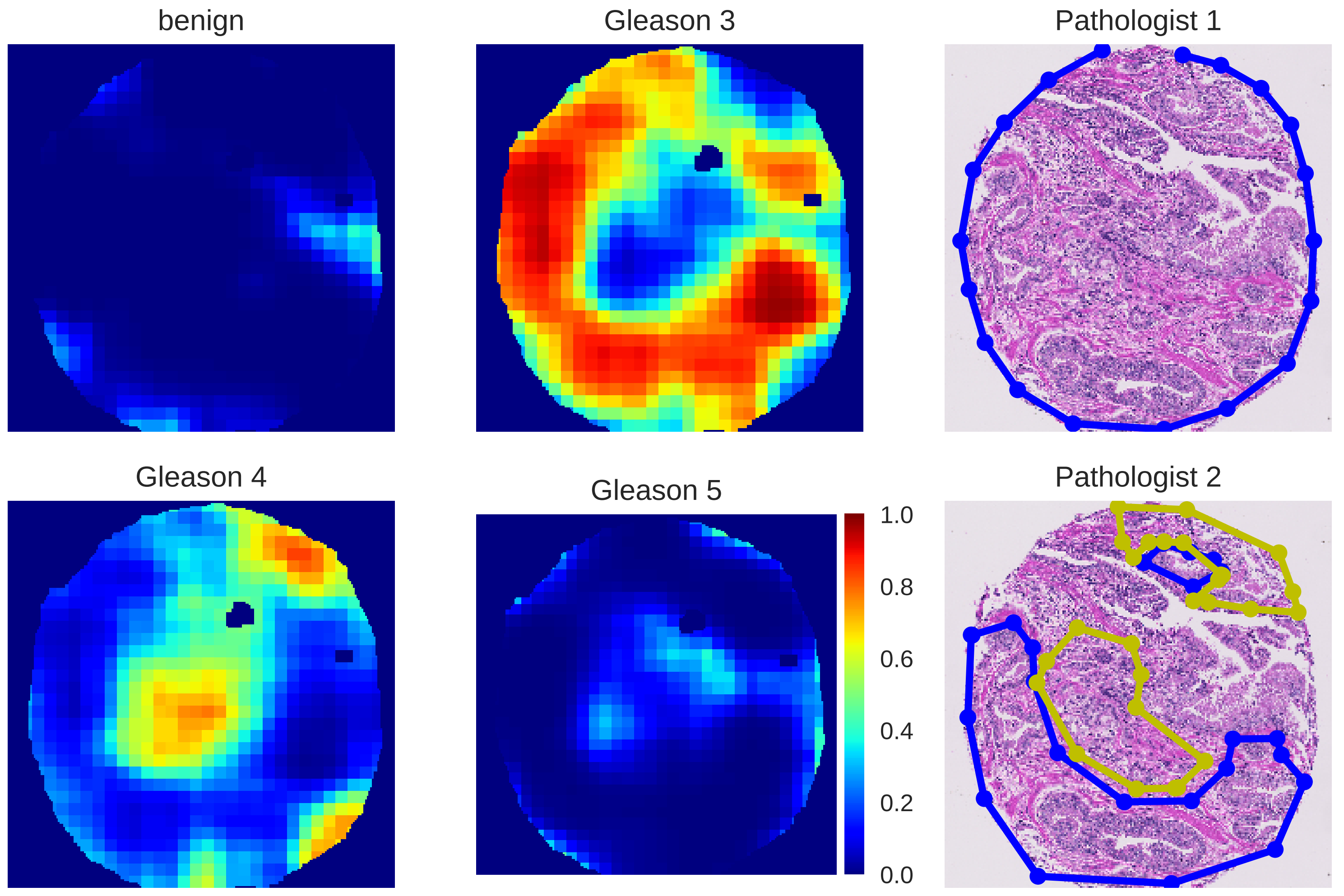
benign
Gleason 3
Pathologist 1
Gleason 4
Gleason 5
Pathologist 2
1.0
0.8
0.6
0.4
0.2
0.0

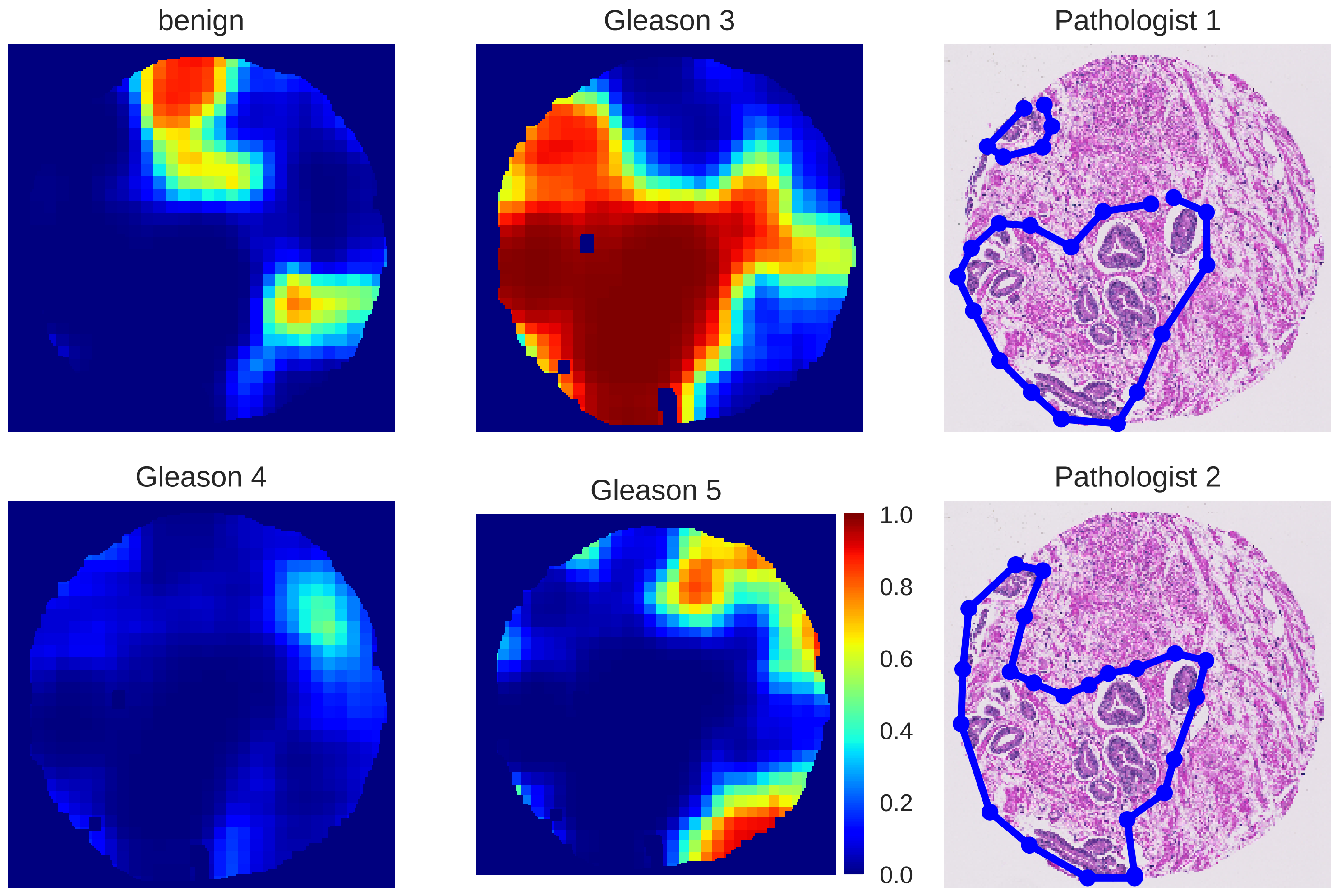

benign
Gleason 3
Pathologist 1
Gleason 4
Gleason 5
1.0
0.8
0.6
0.4
0.2
0.0
Pathologist 2

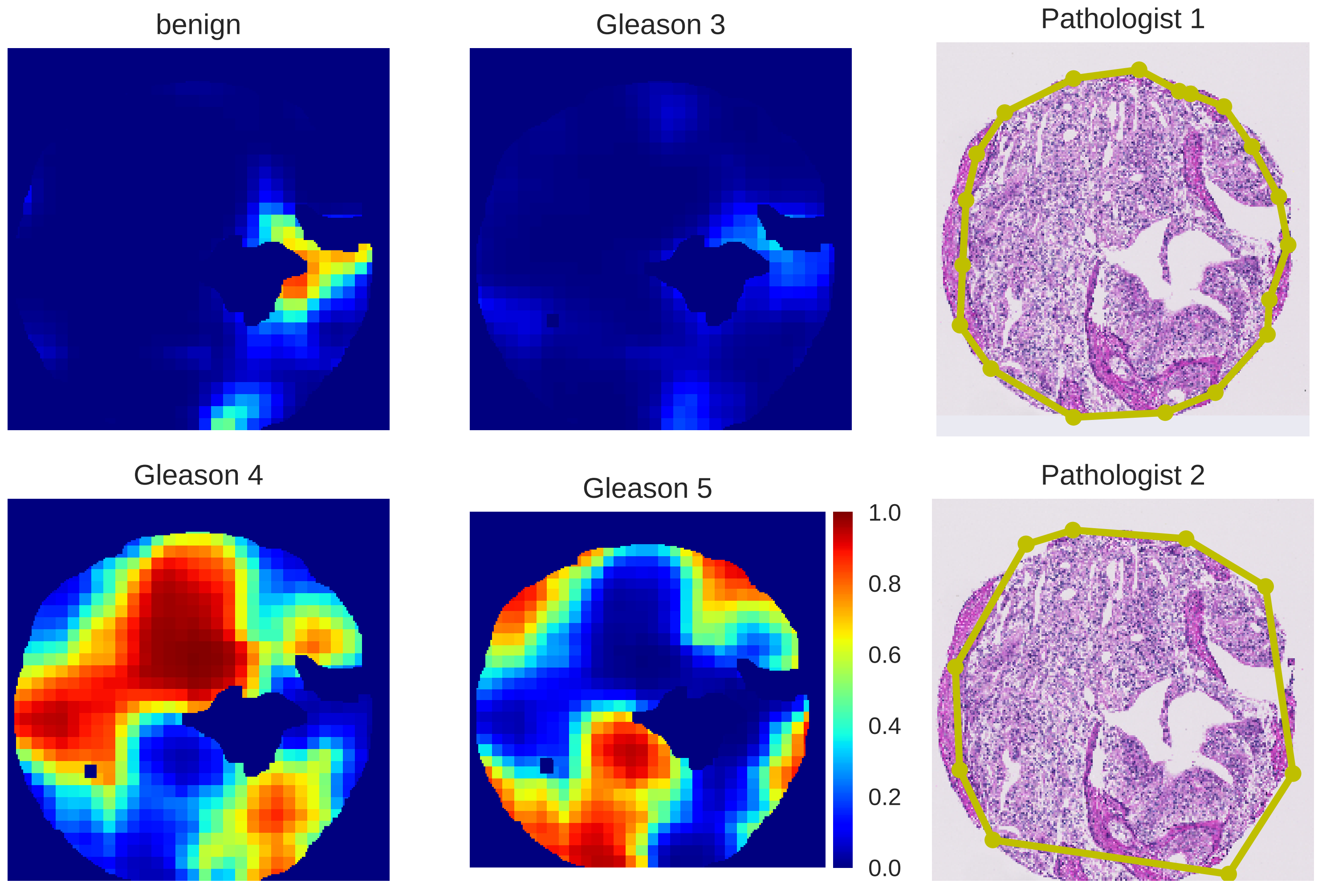

benign
Gleason 3
Pathologist 1
Gleason 4
Gleason 5
Pathologist 2
1.0
0.8
0.6
0.4
0.2
0.0

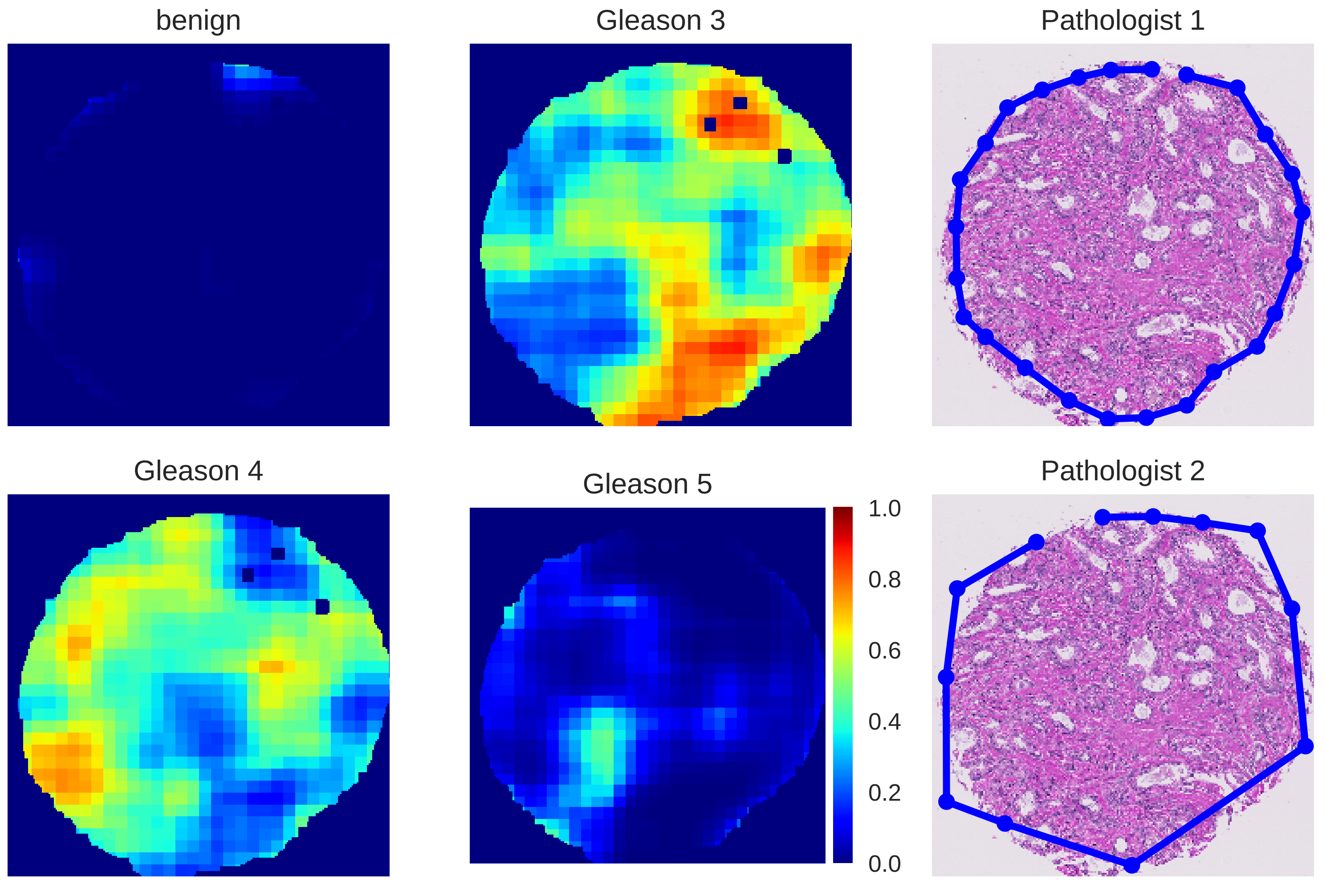

benign
Gleason 3
Pathologist 1
Gleason 4
Gleason 5
Pathologist 2
1.0
0.8
0.6
0.4
0.2
0.0

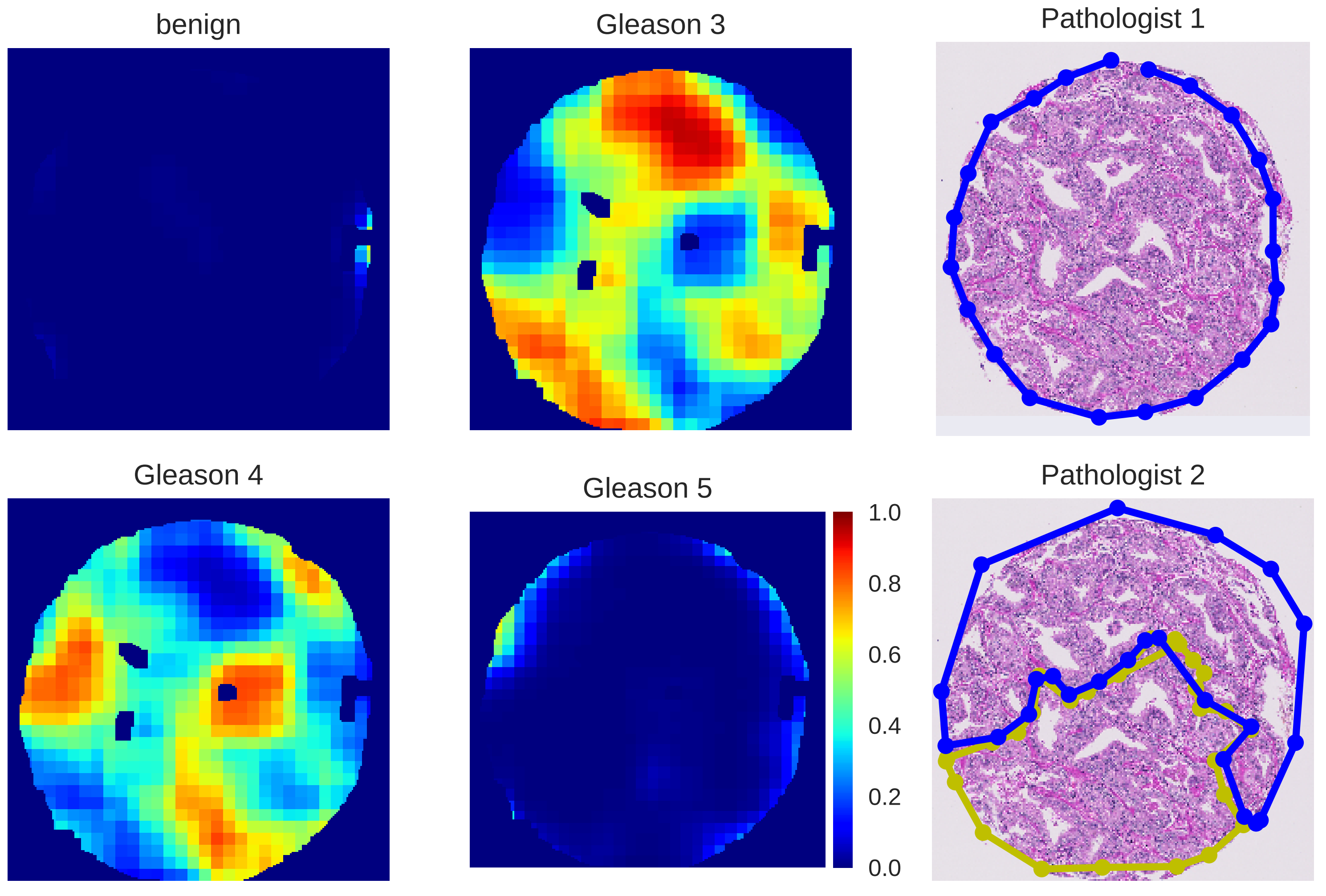

benign
Gleason 3
Pathologist 1
Gleason 4
Gleason 5
Pathologist 2
1.0
0.8
0.6
0.4
0.2
0.0

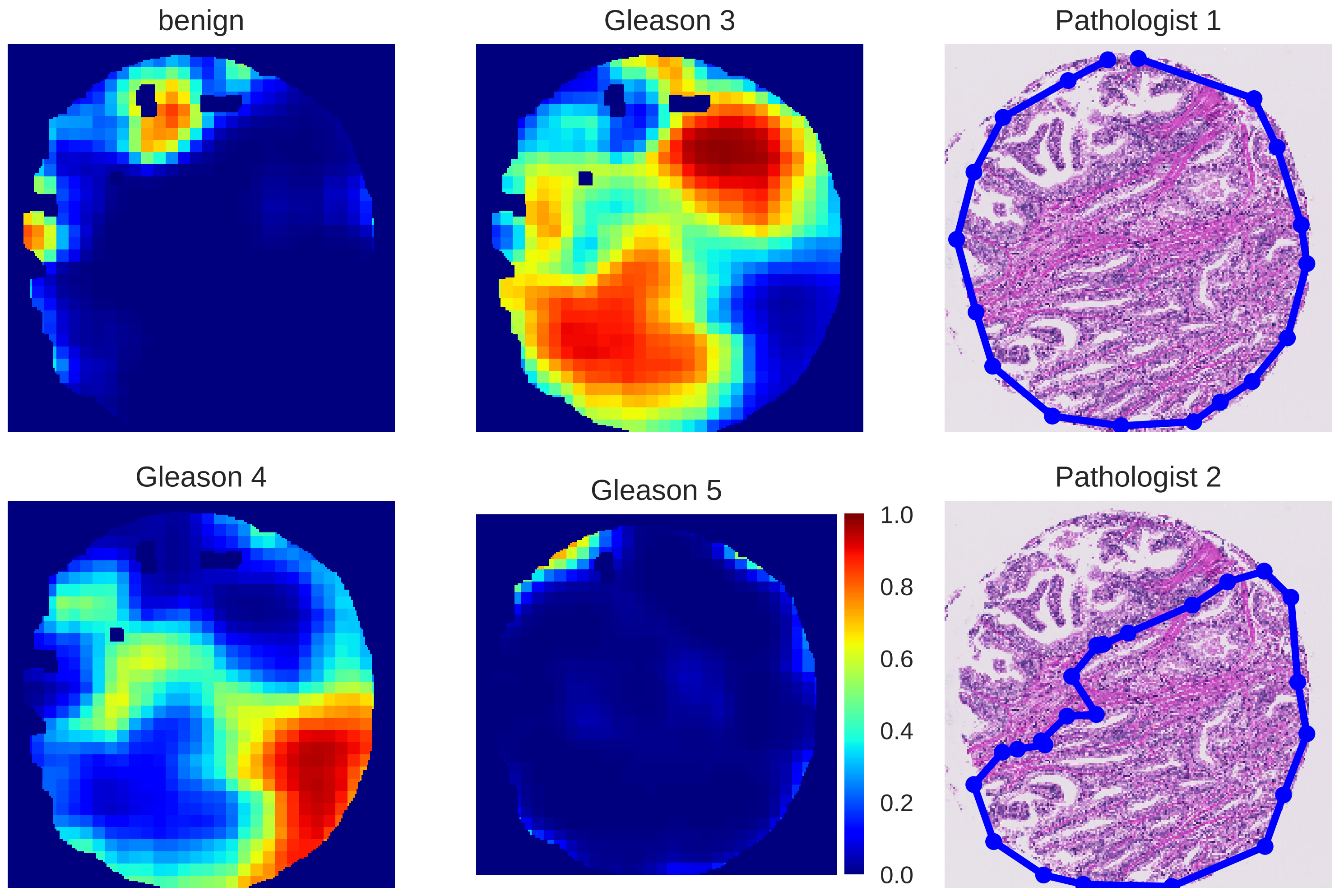
benign
Gleason 3
Pathologist 1
Gleason 4
Gleason 5
Pathologist 2
1.0
0.8
0.6
0.4
0.2
0.0

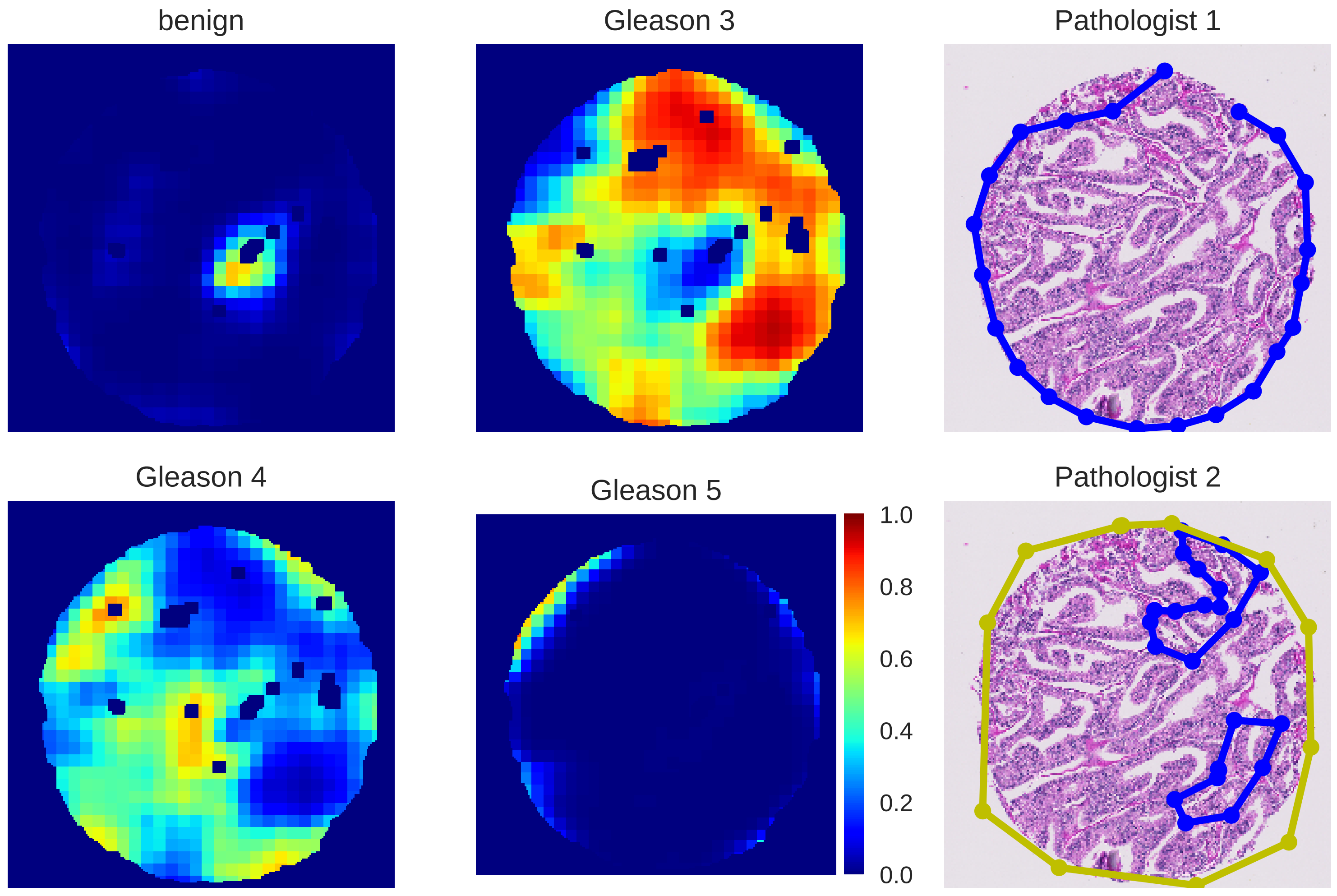
benign
Gleason 3
Pathologist 1
Gleason 4
Gleason 5
Pathologist 2
1.0
0.8
0.6
0.4
0.2
0.0

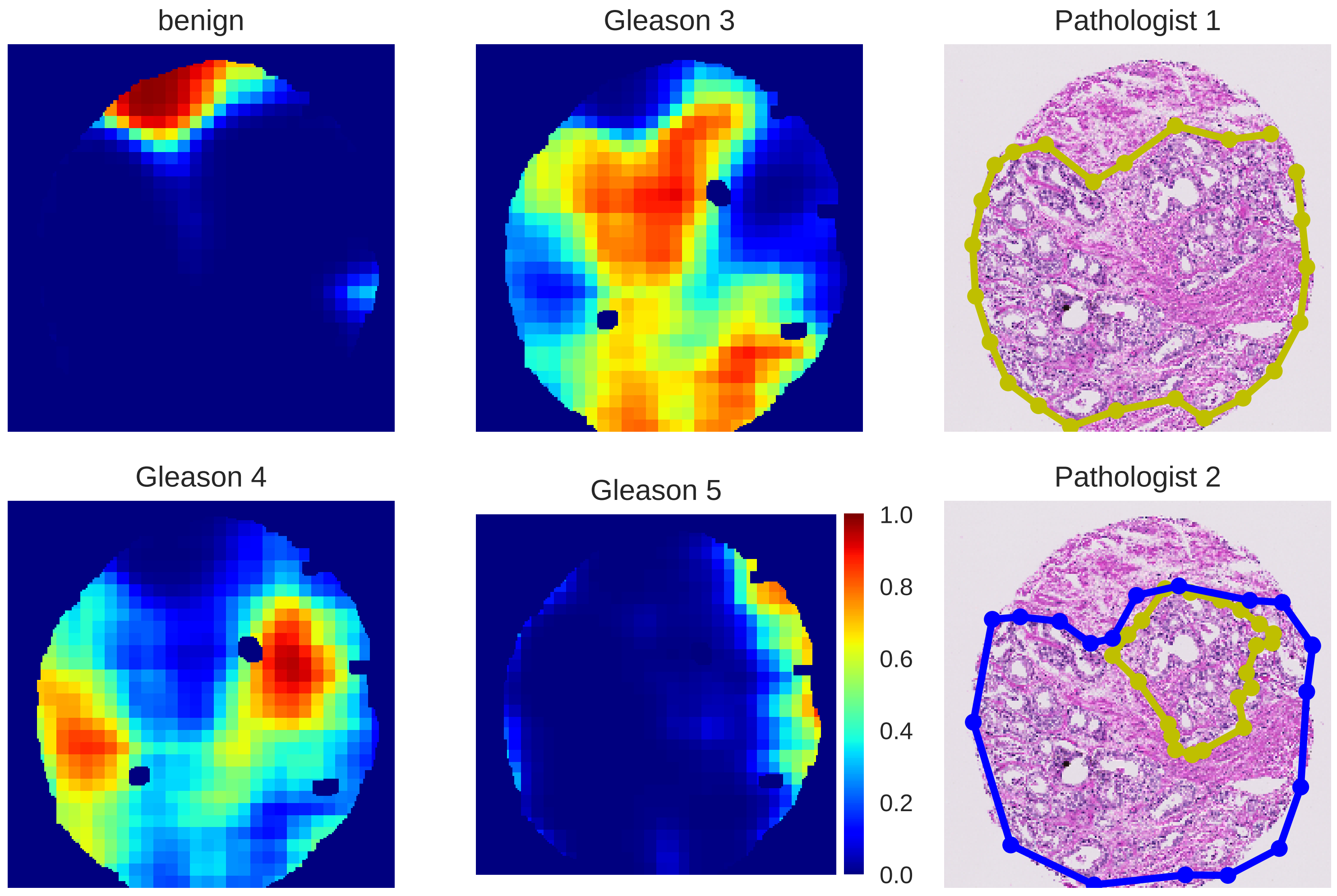

benign
Gleason 3
Pathologist 1
Gleason 4
Gleason 5
Pathologist 2
1.0
0.8
0.6
0.4
0.2
0.0

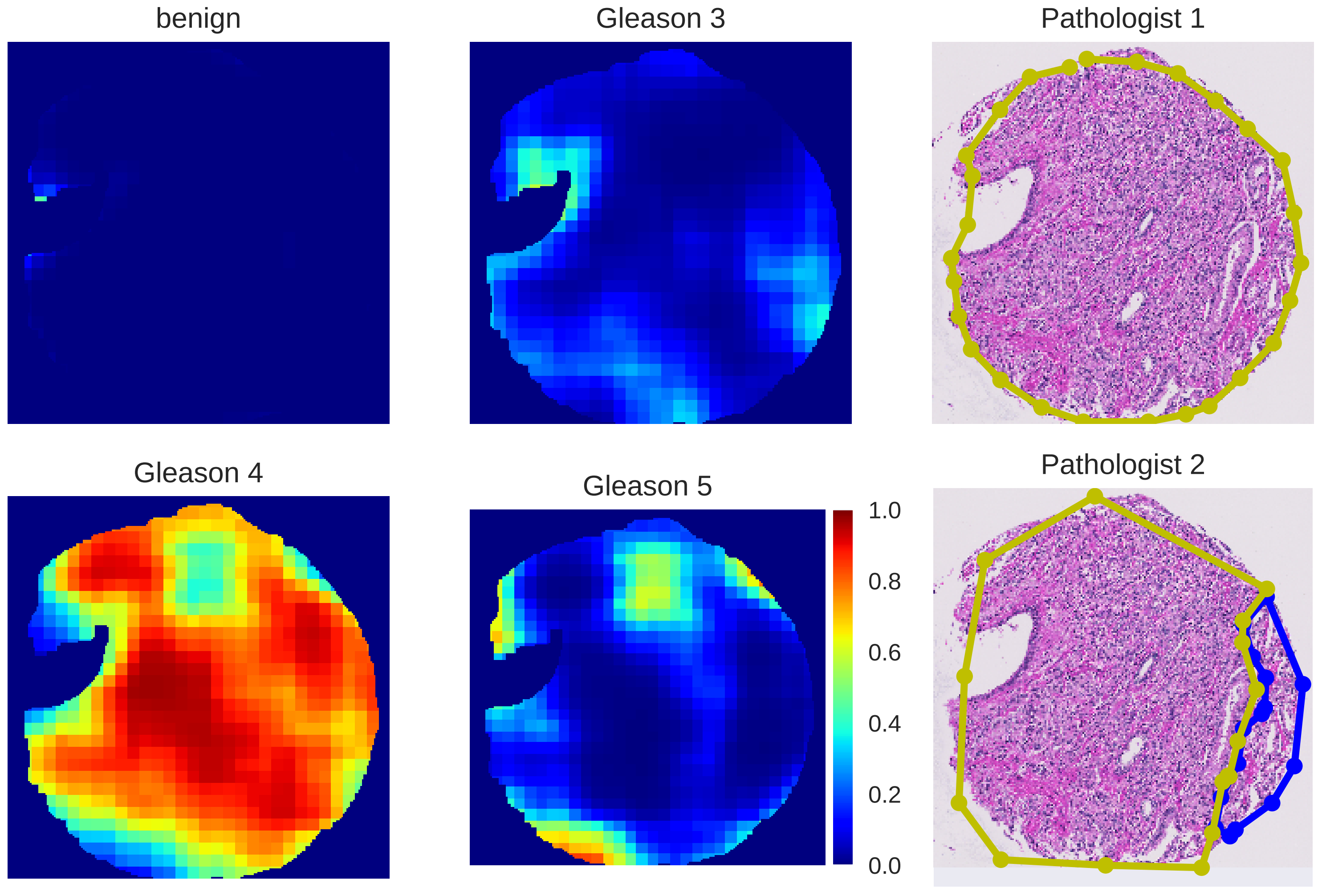

benign
Gleason 3
Pathologist 1
Gleason 4
Gleason 5
Pathologist 2
1.0
0.8
0.6
0.4
0.2
0.0

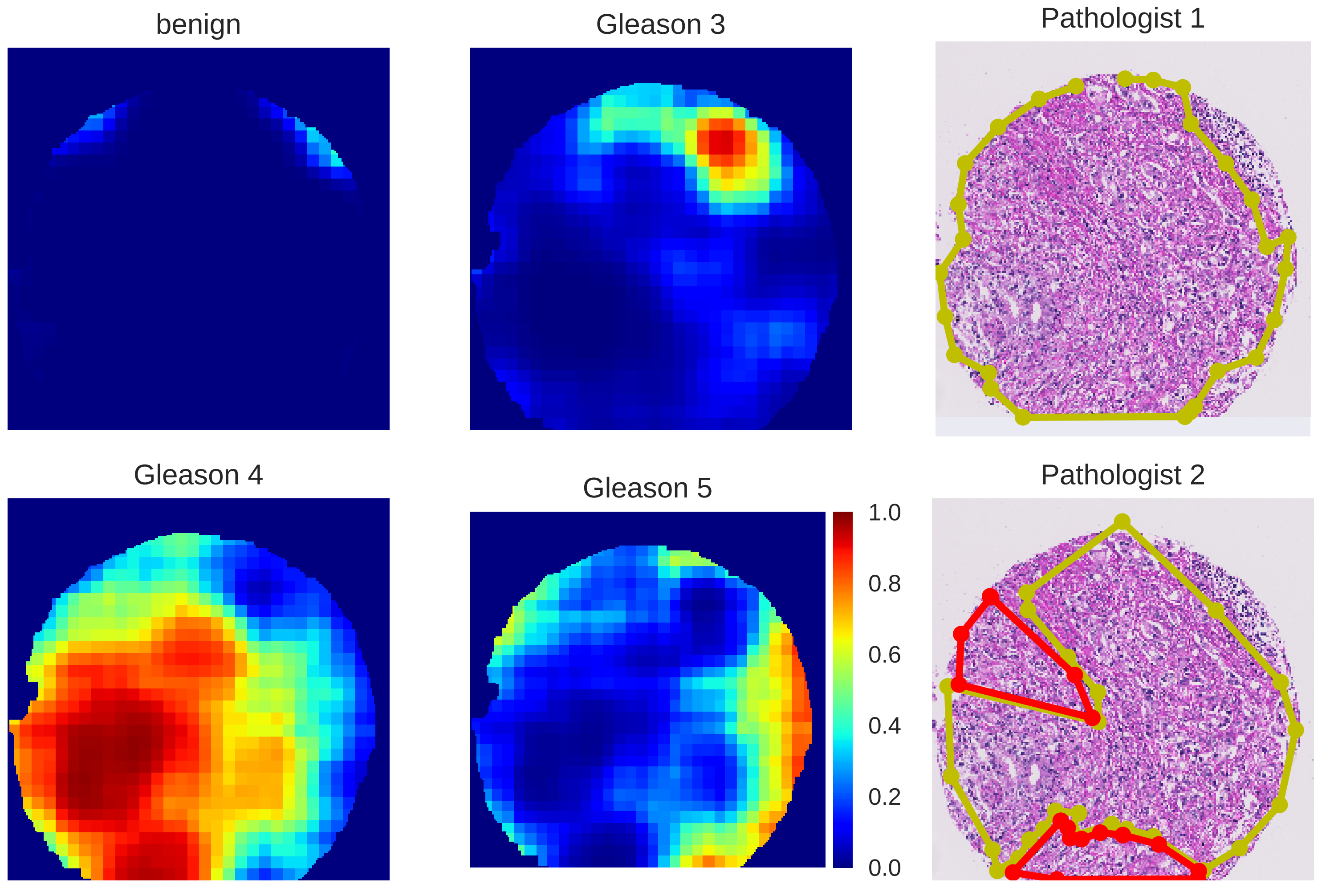

benign
Gleason 3
Pathologist 1
Gleason 4
Gleason 5
Pathologist 2
1.0
0.8
0.6
0.4
0.2
0.0

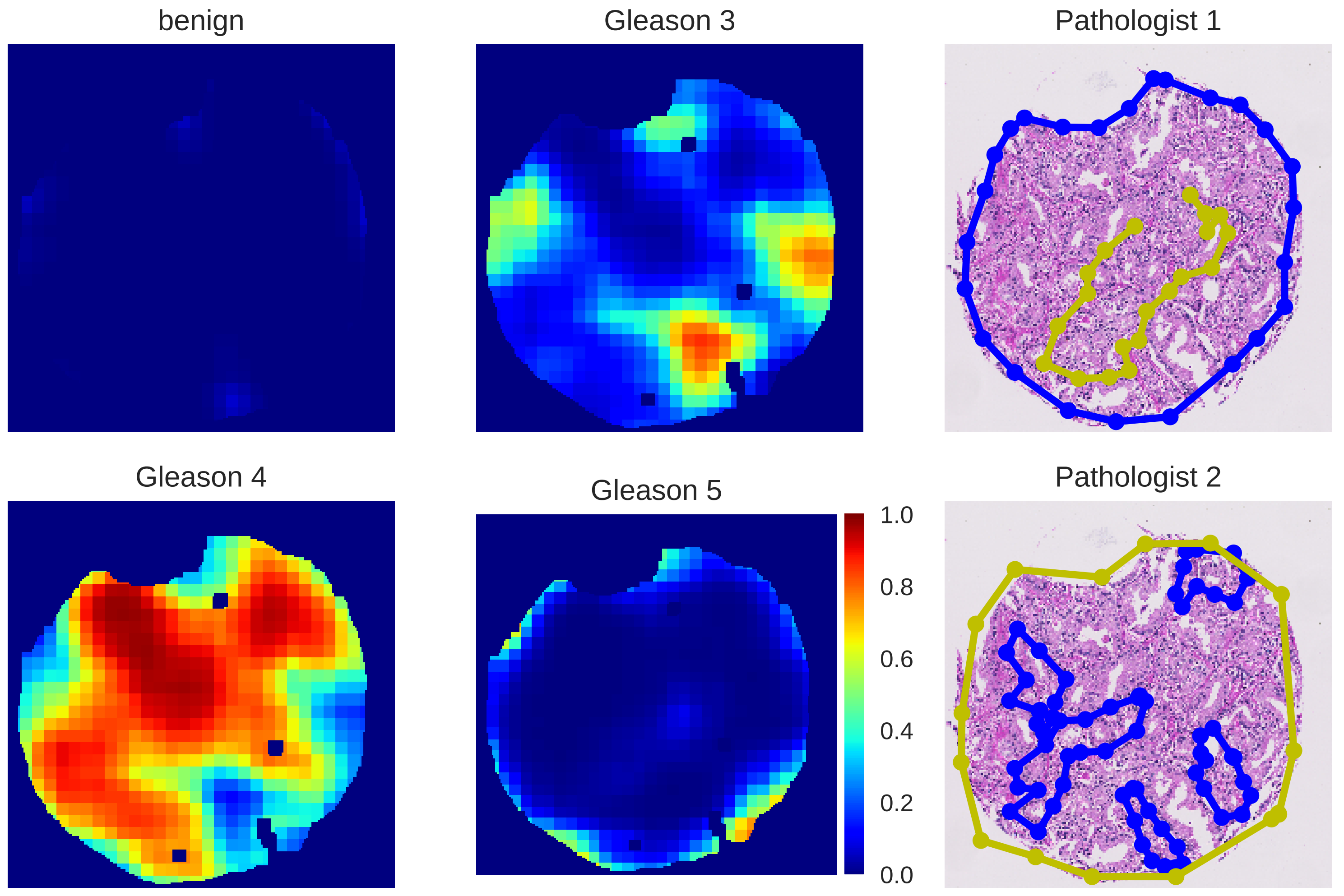
benign
Gleason 3
Pathologist 1
Gleason 4
Gleason 5
Pathologist 2
1.0
0.8
0.6
0.4
0.2
0.0

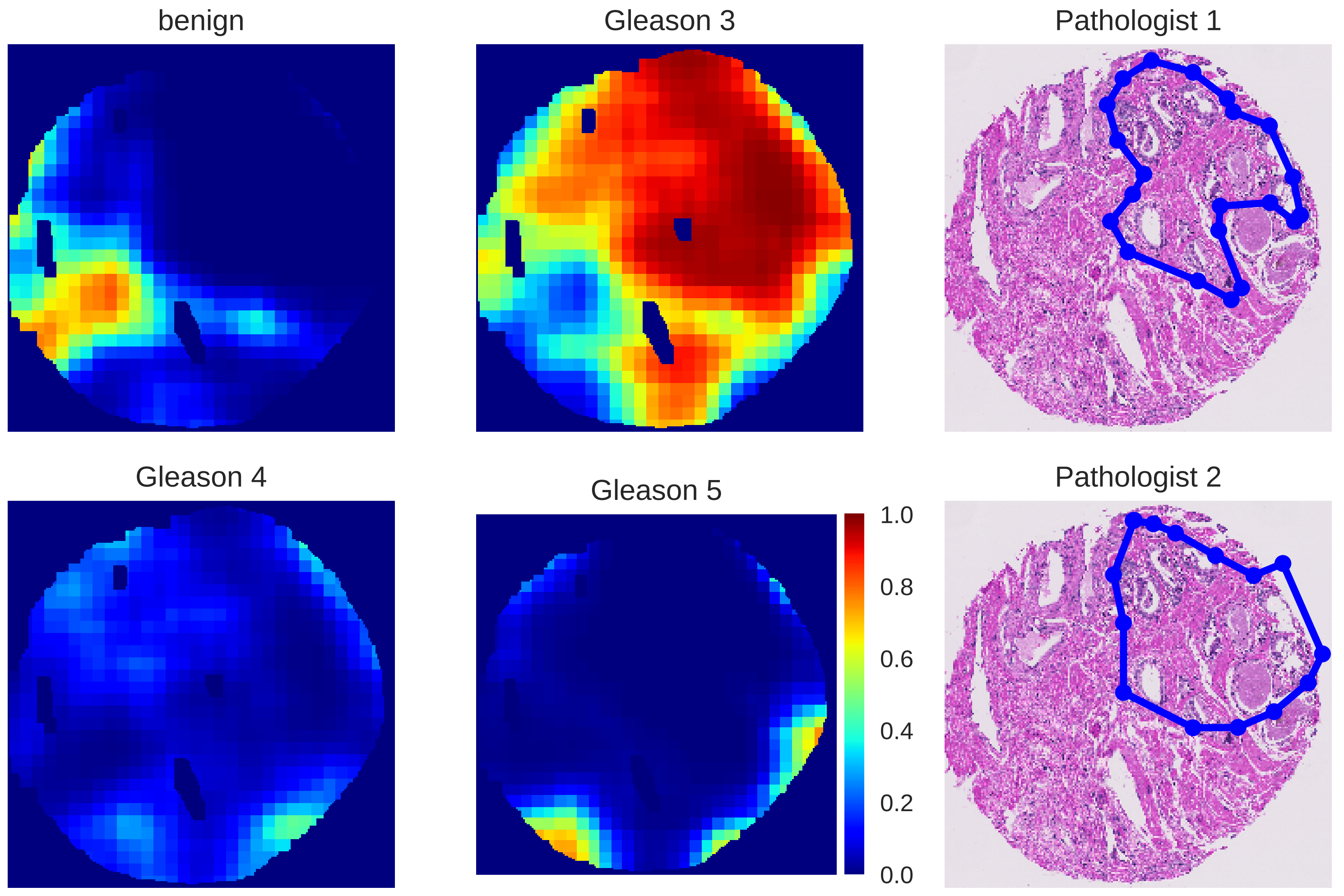

benign
Gleason 3
Pathologist 1
Gleason 4
Gleason 5
Pathologist 2
1.0
0.8
0.6
0.4
0.2
0.0

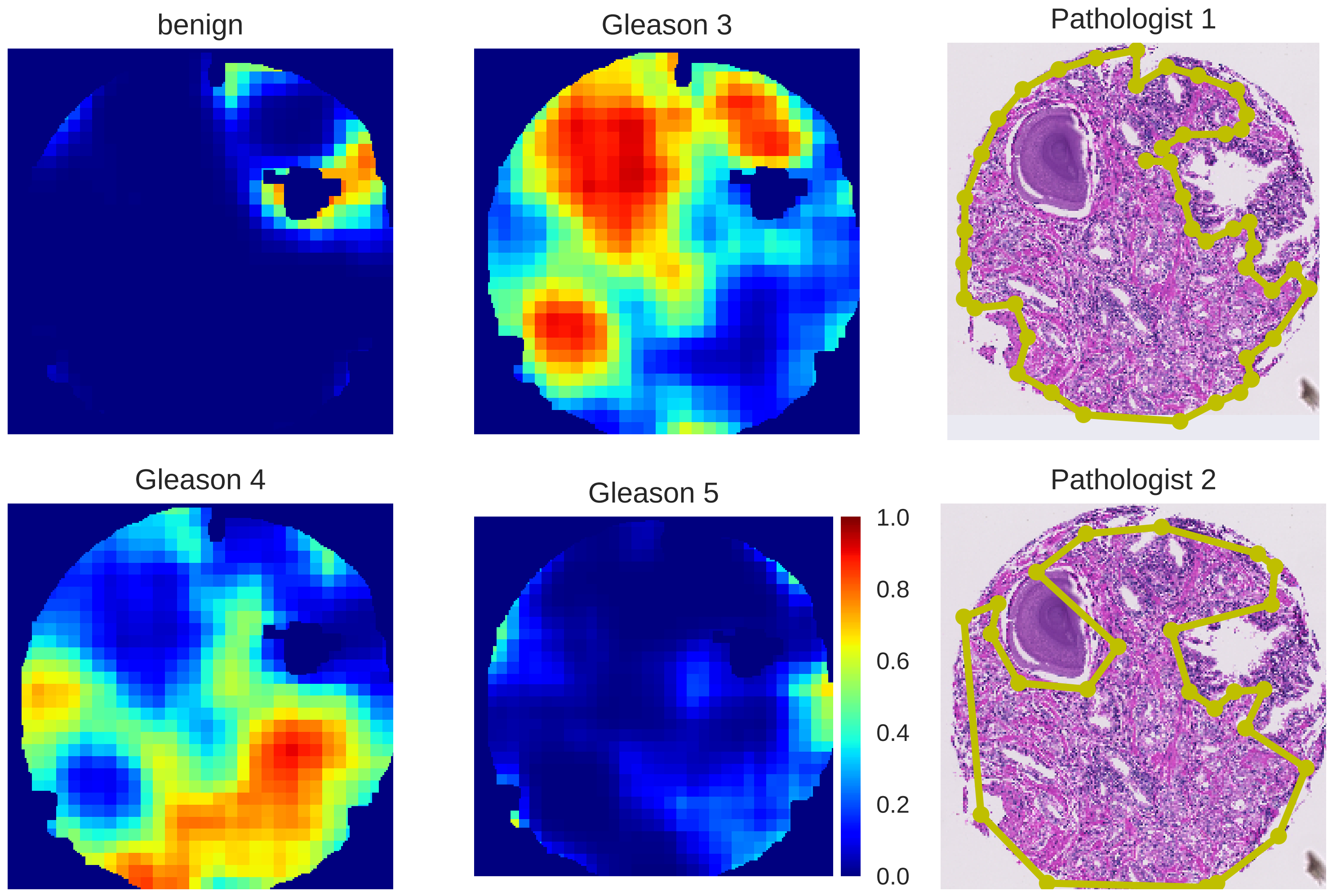
benign
Gleason 3
Pathologist 1
Gleason 4
Gleason 5
Pathologist 2
1.0
0.8
0.6
0.4
0.2
0.0

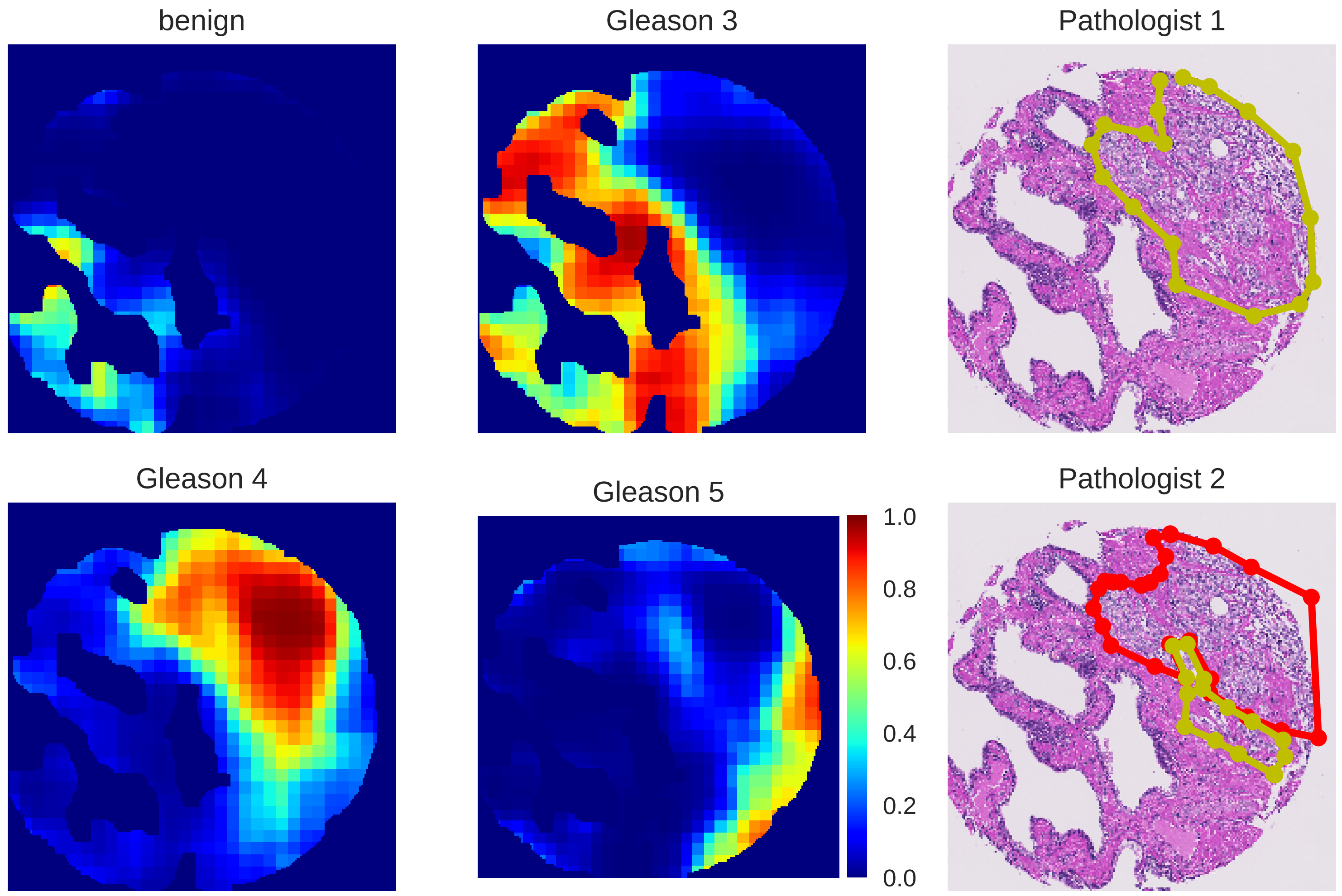

benign
Gleason 3
Pathologist 1
Gleason 4
Gleason 5
Pathologist 2
1.0
0.8
0.6
0.4
0.2
0.0

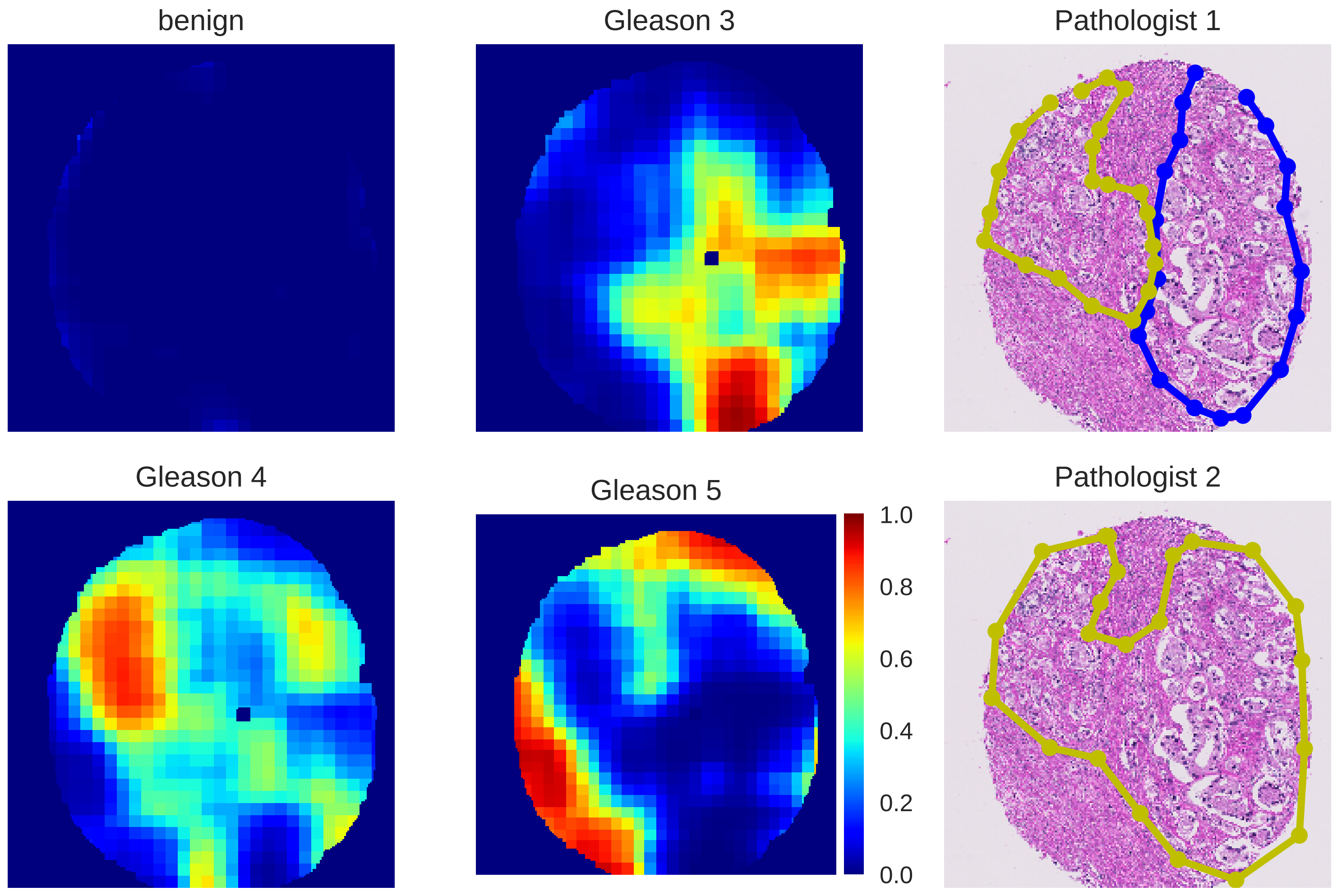

benign
Gleason 3
Pathologist 1
Gleason 4
Gleason 5
Pathologist 2
1.0
0.8
0.6
0.4
0.2
0.0

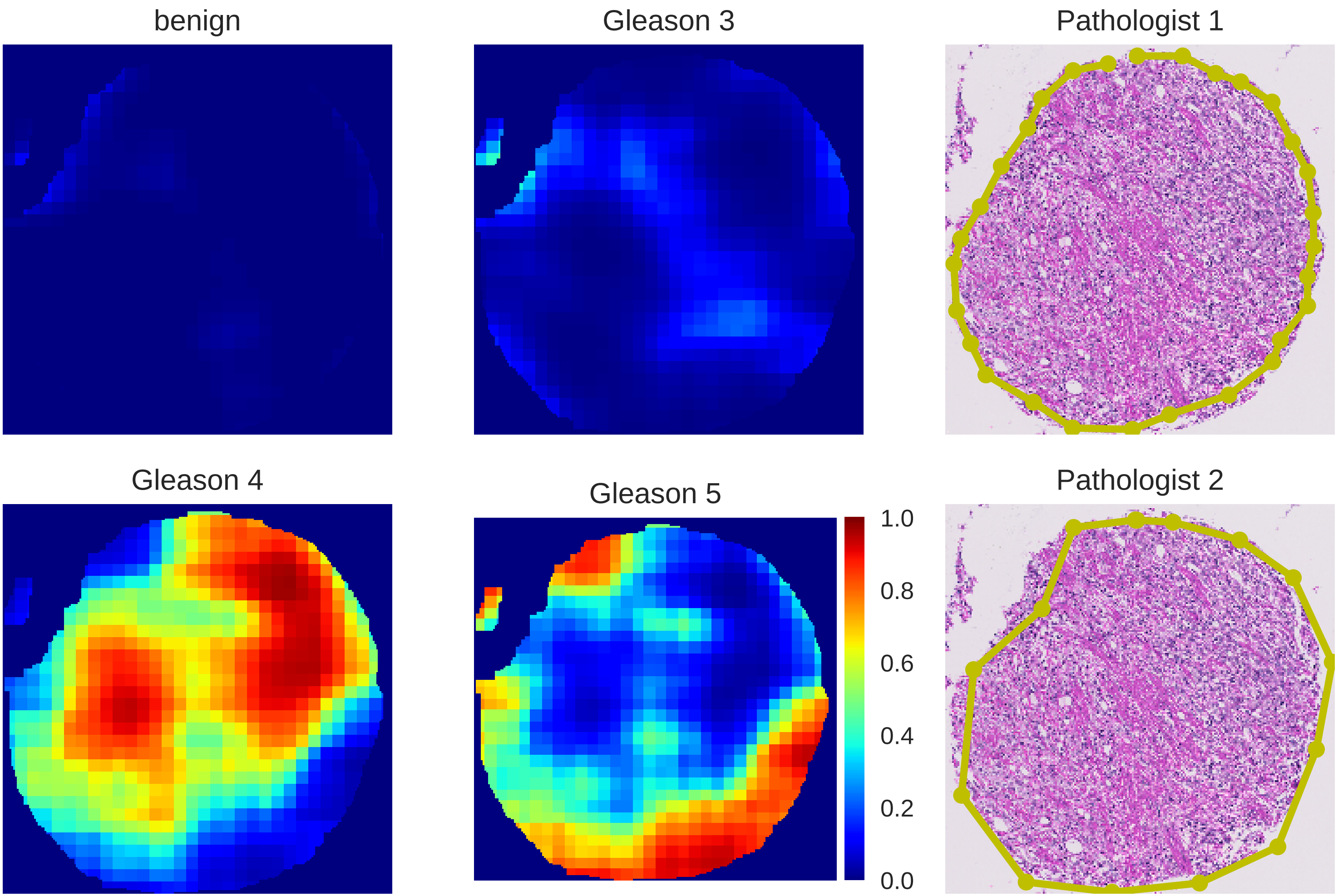
benign
Gleason 3
Pathologist 1
Gleason 4
Gleason 5
1.0
0.8
0.6
0.4
0.2
0.0
Pathologist 2

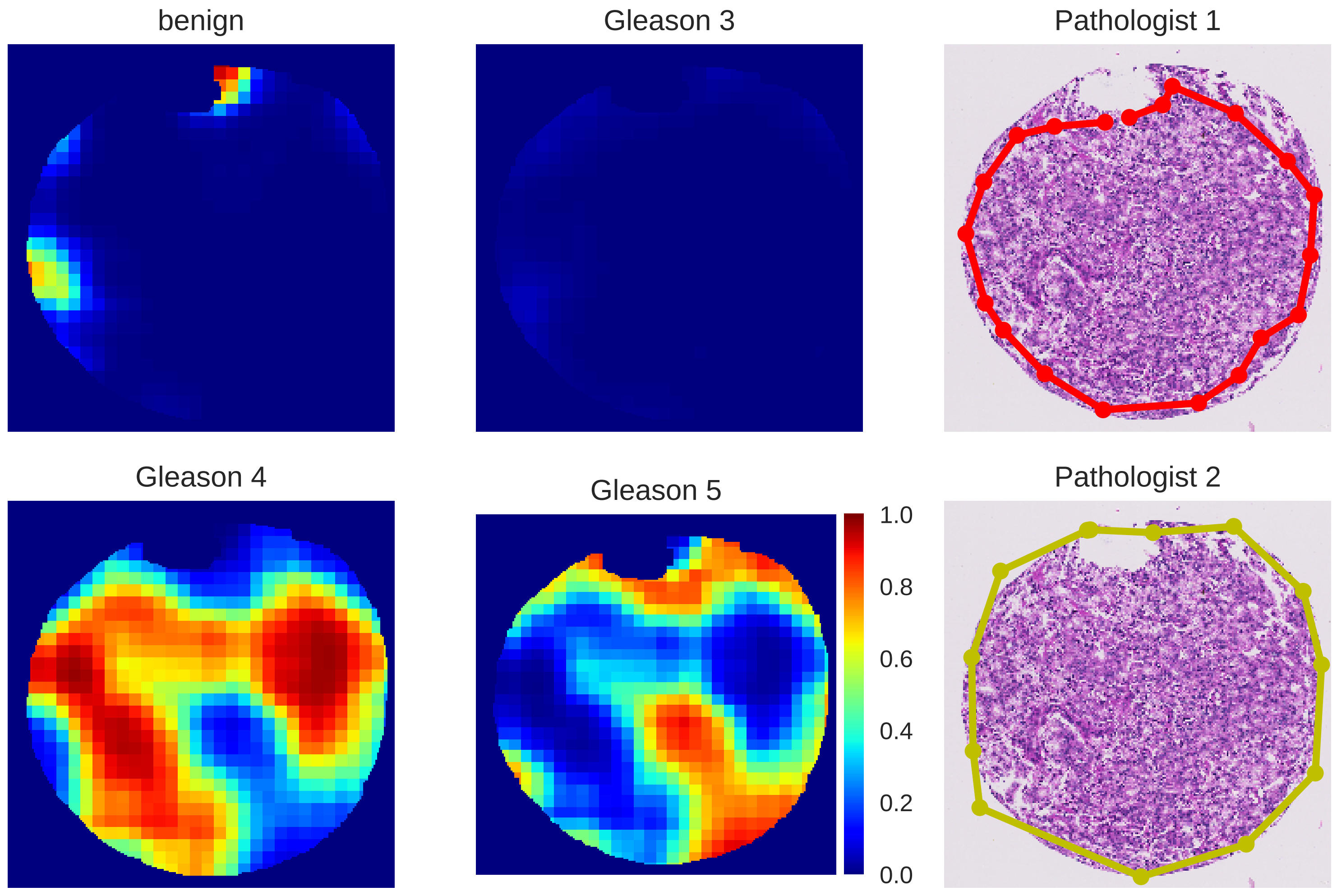
benign
Gleason 3
Pathologist 1
Gleason 4
Gleason 5
Pathologist 2
1.0
0.8
0.6
0.4
0.2
0.0

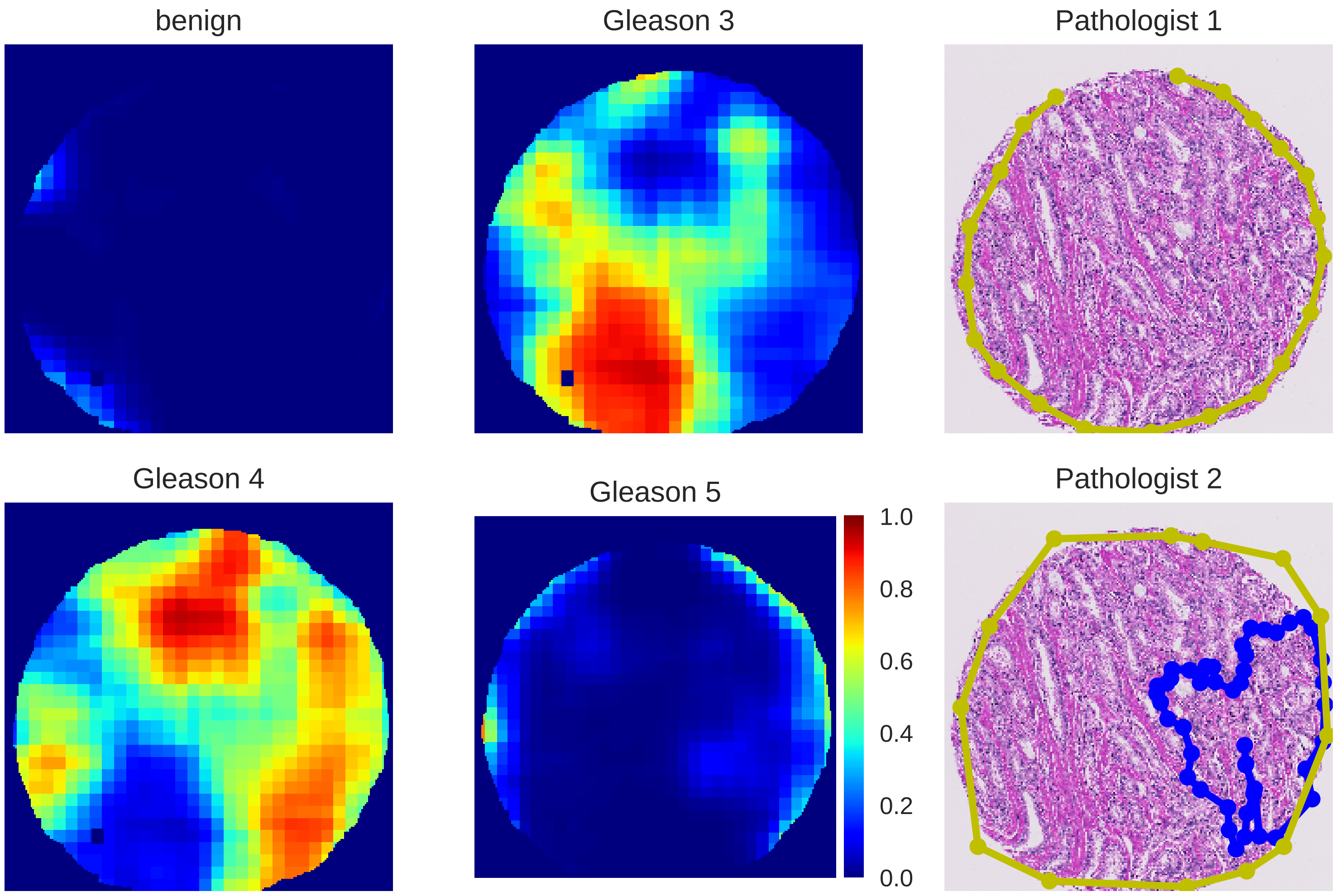
benign
Gleason 3
Pathologist 1
Gleason 4
Gleason 5
Pathologist 2
1.0
0.8
0.6
0.4
0.2
0.0

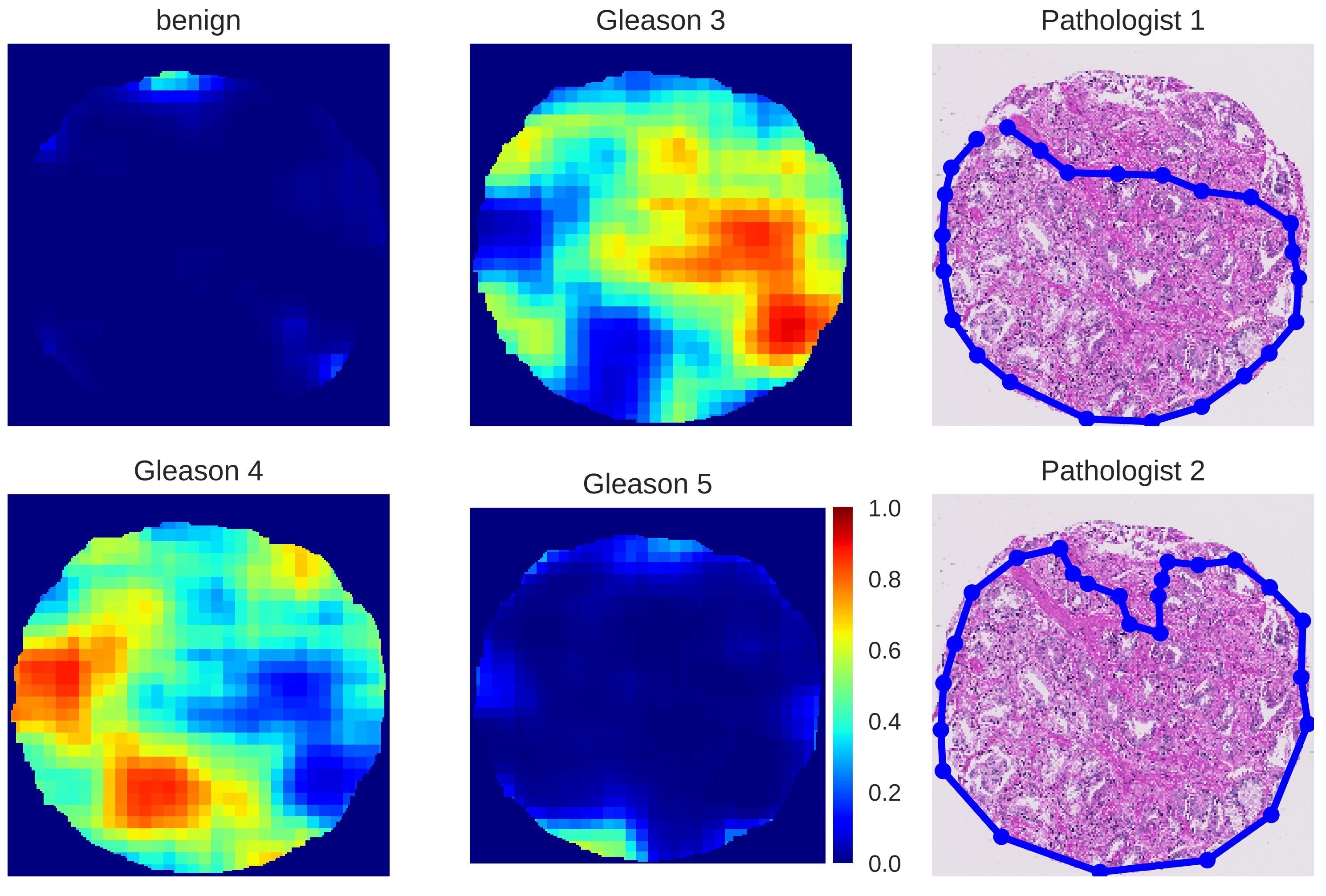
benign
Gleason 3
Pathologist 1
Gleason 4
Gleason 5
1.0
0.8
0.6
0.4
0.2
0.0
Pathologist 2

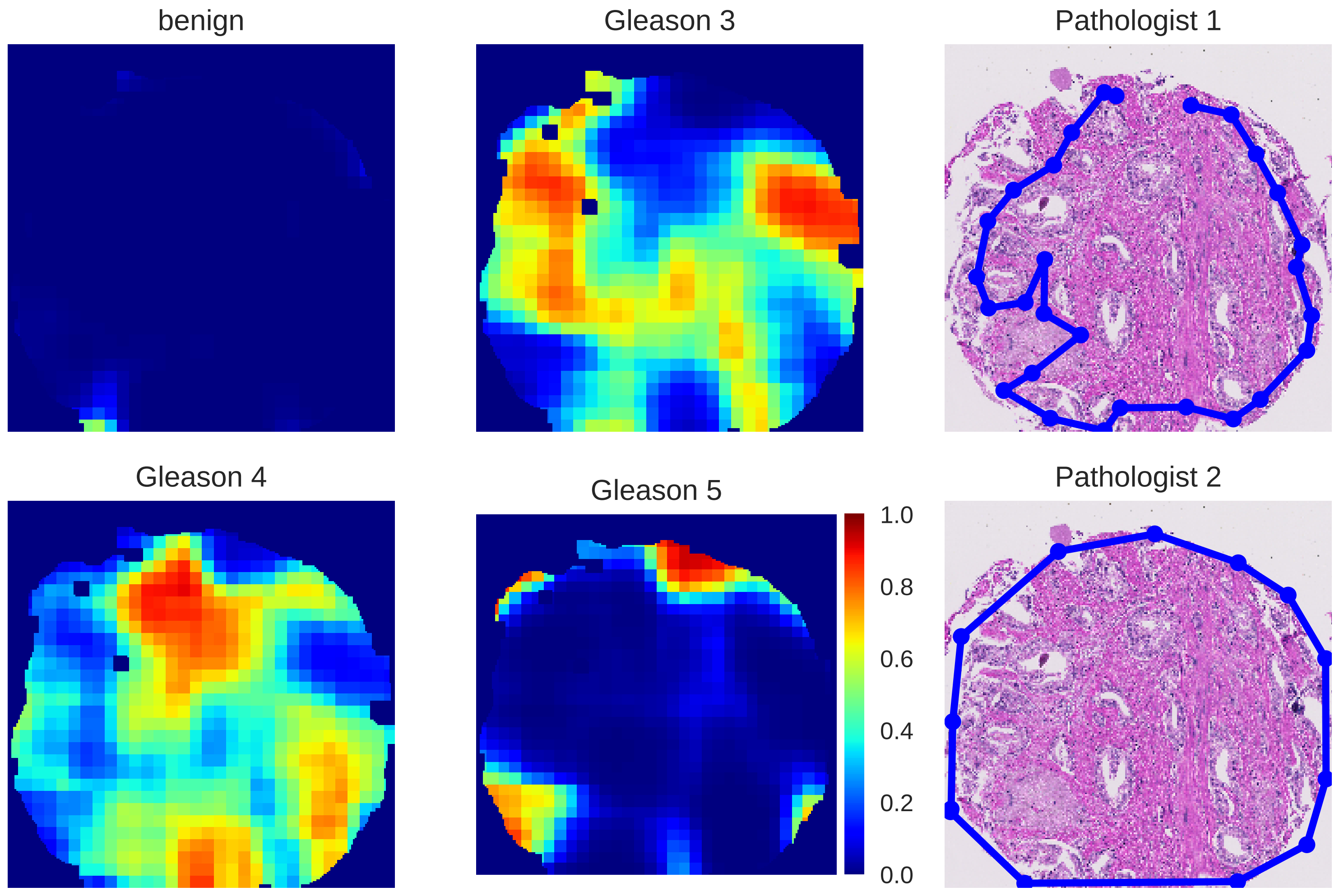
benign
Gleason 3
Pathologist 1
Gleason 4
Gleason 5
Pathologist 2
1.0
0.8
0.6
0.4
0.2
0.0

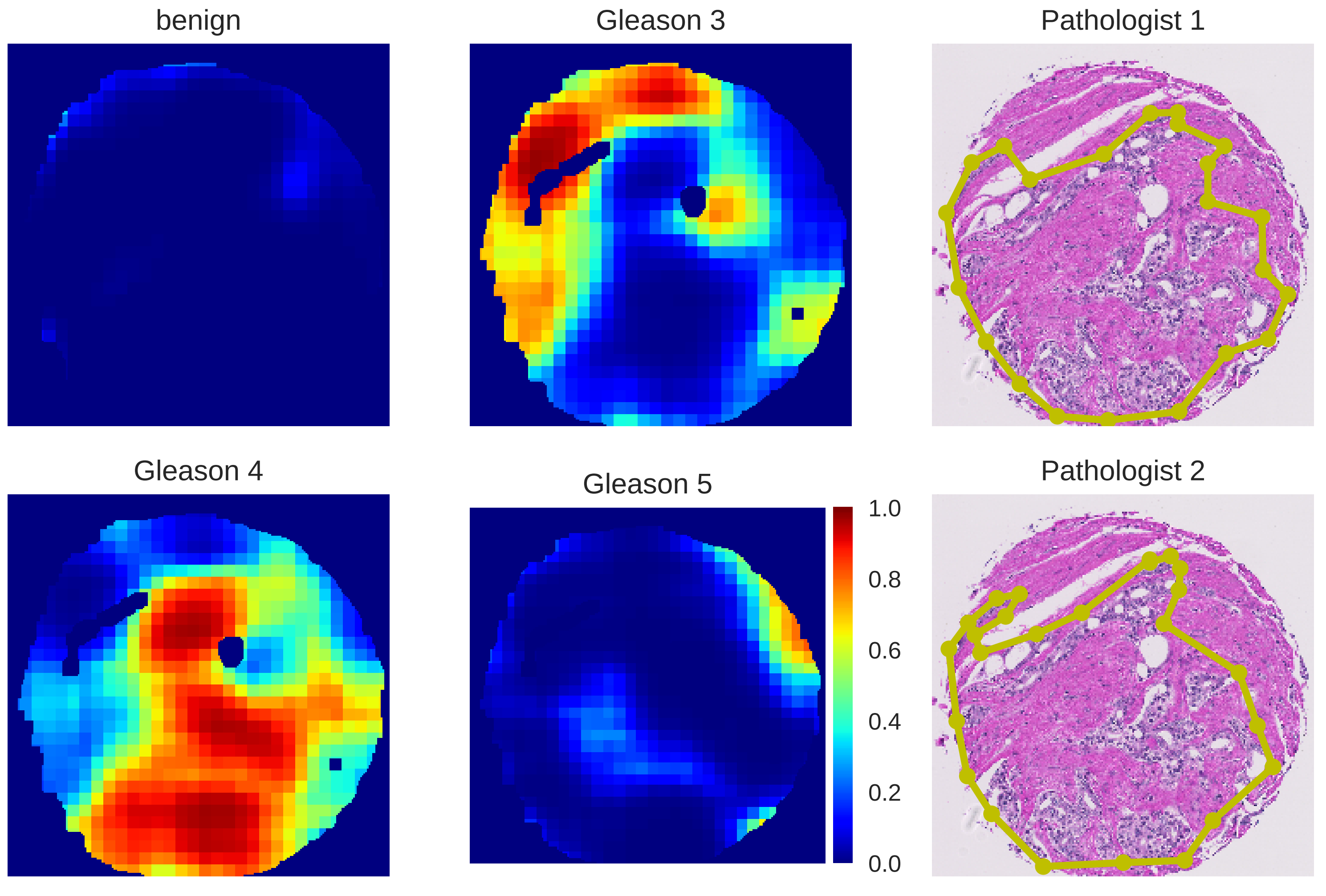
benign
Gleason 3
Pathologist 1
Gleason 4
Gleason 5
Pathologist 2
1.0
0.8
0.6
0.4
0.2
0.0

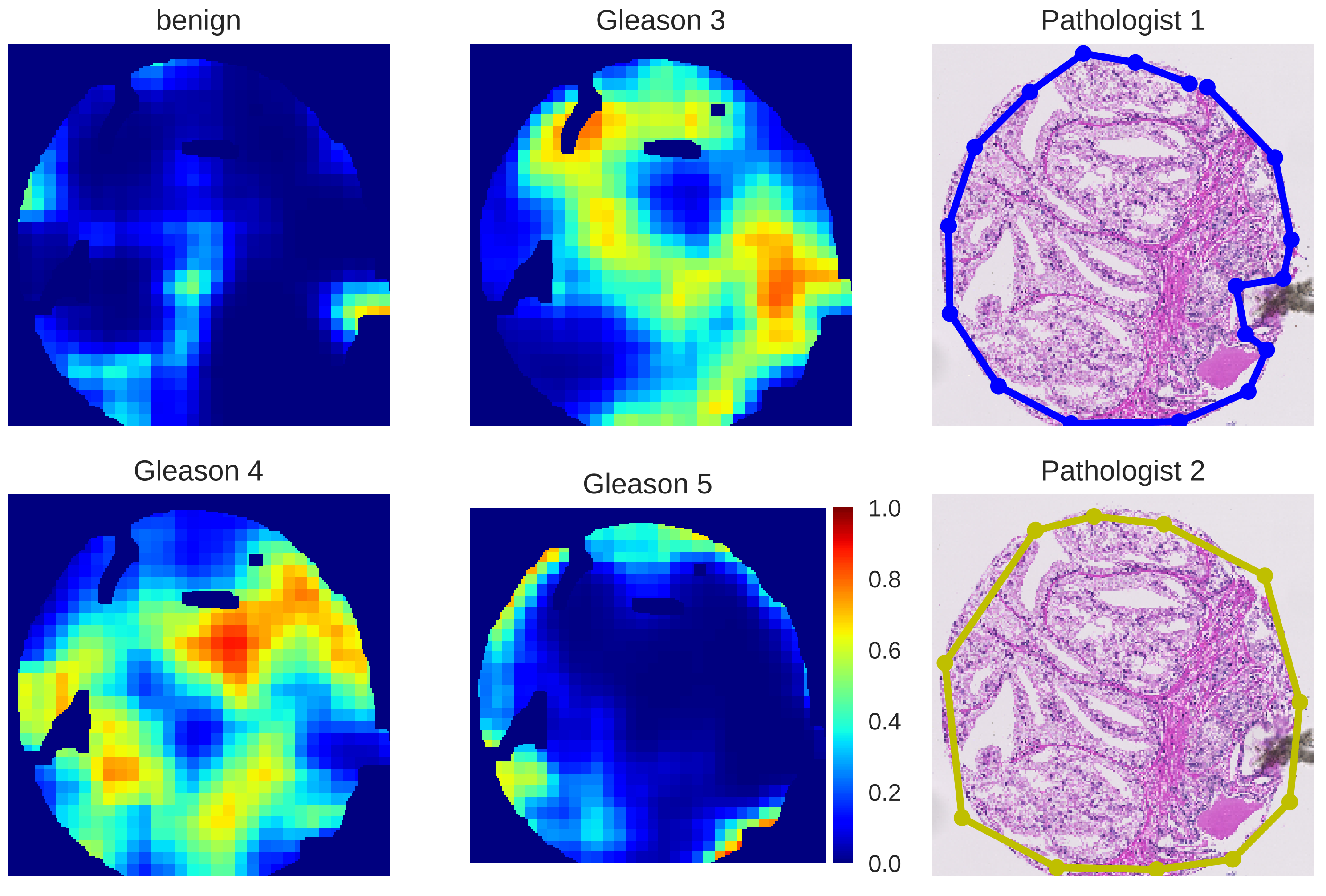

benign
Gleason 3
Pathologist 1
Gleason 4
Gleason 5
1.0
0.8
0.6
0.4
0.2
0.0
Pathologist 2

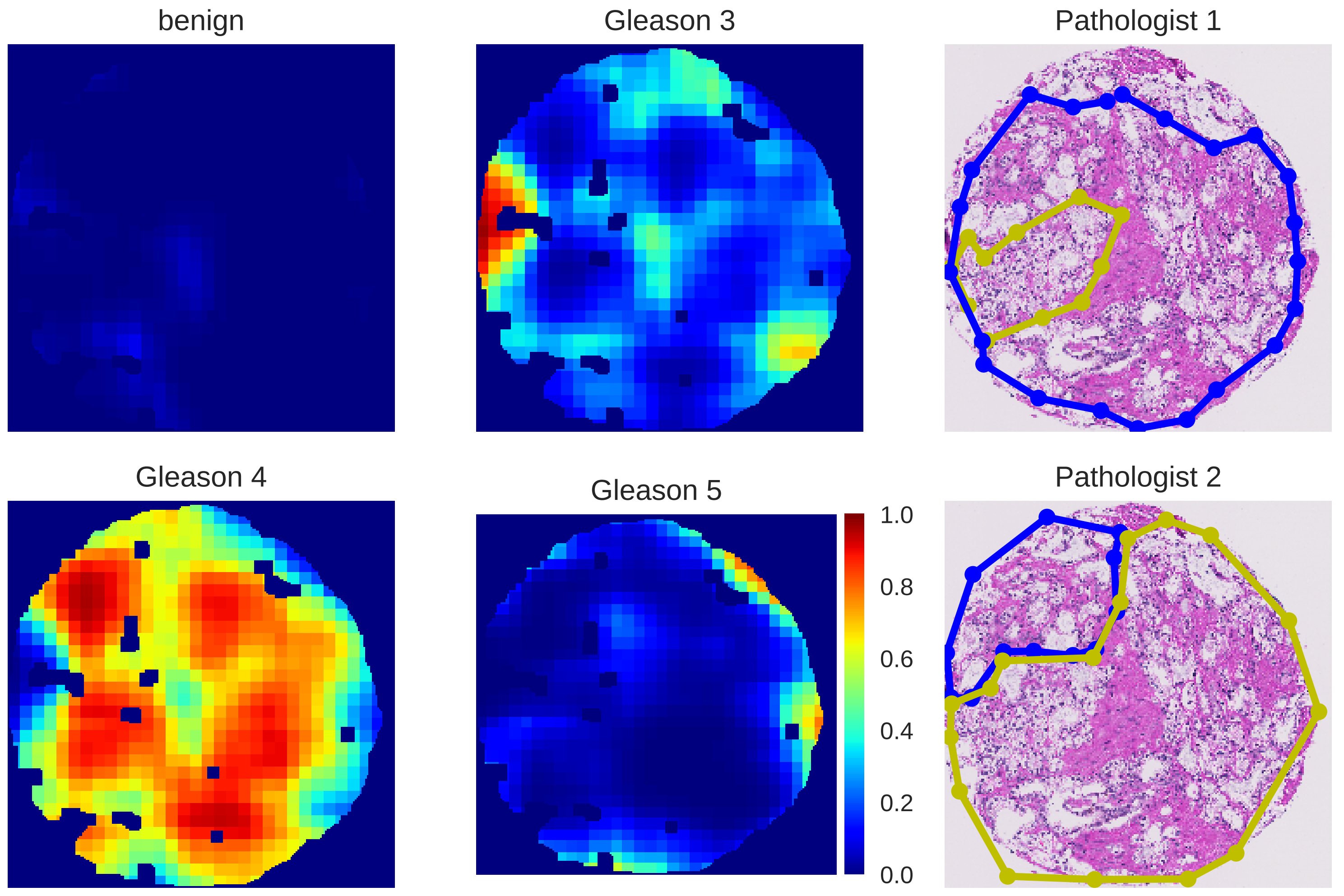

benign
Gleason 3
Pathologist 1
Gleason 4
Gleason 5
Pathologist 2
1.0
0.8
0.6
0.4
0.2
0.0

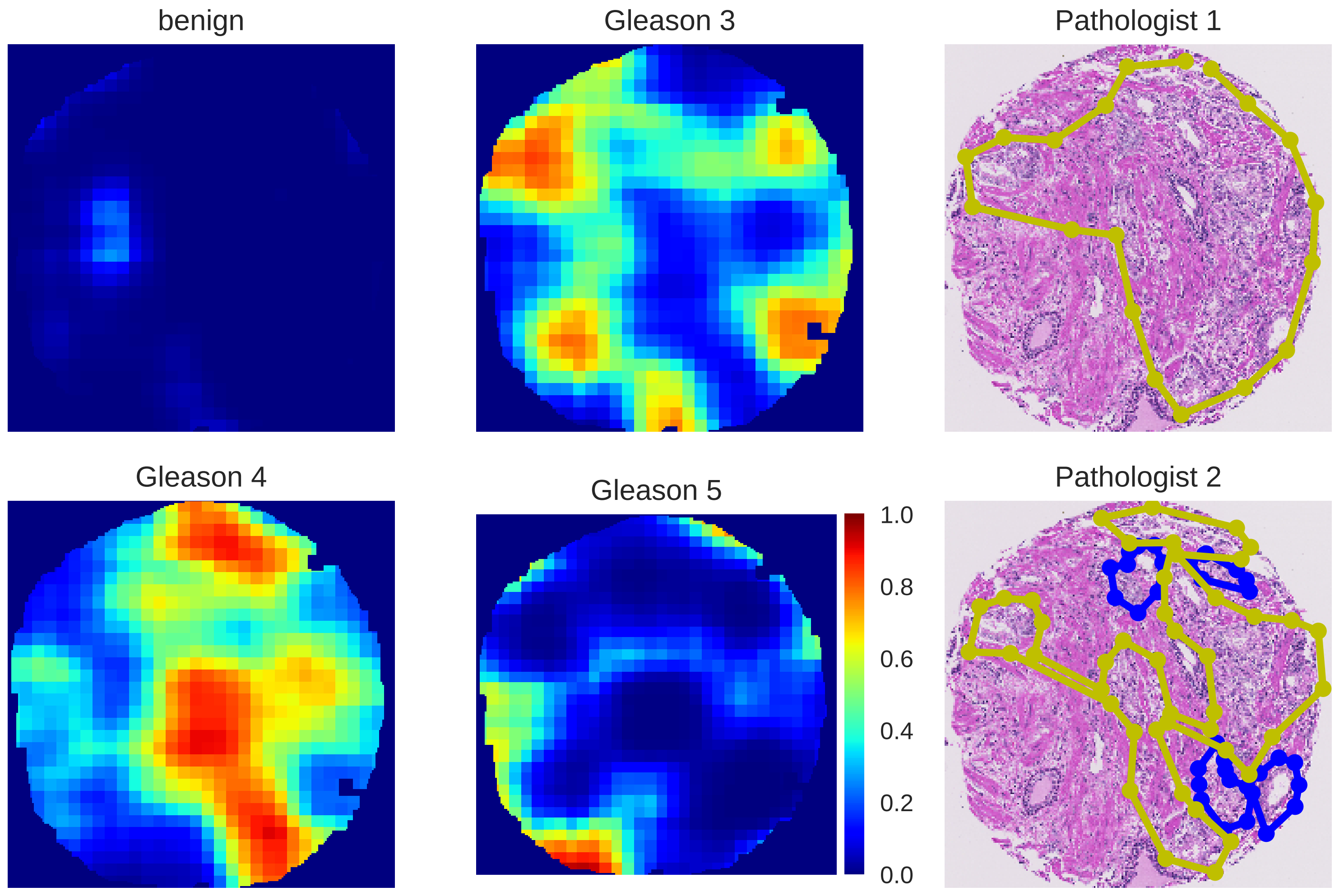

benign
Gleason 3
Pathologist 1
Gleason 4
Gleason 5
Pathologist 2
1.0
0.8
0.6
0.4
0.2
0.0

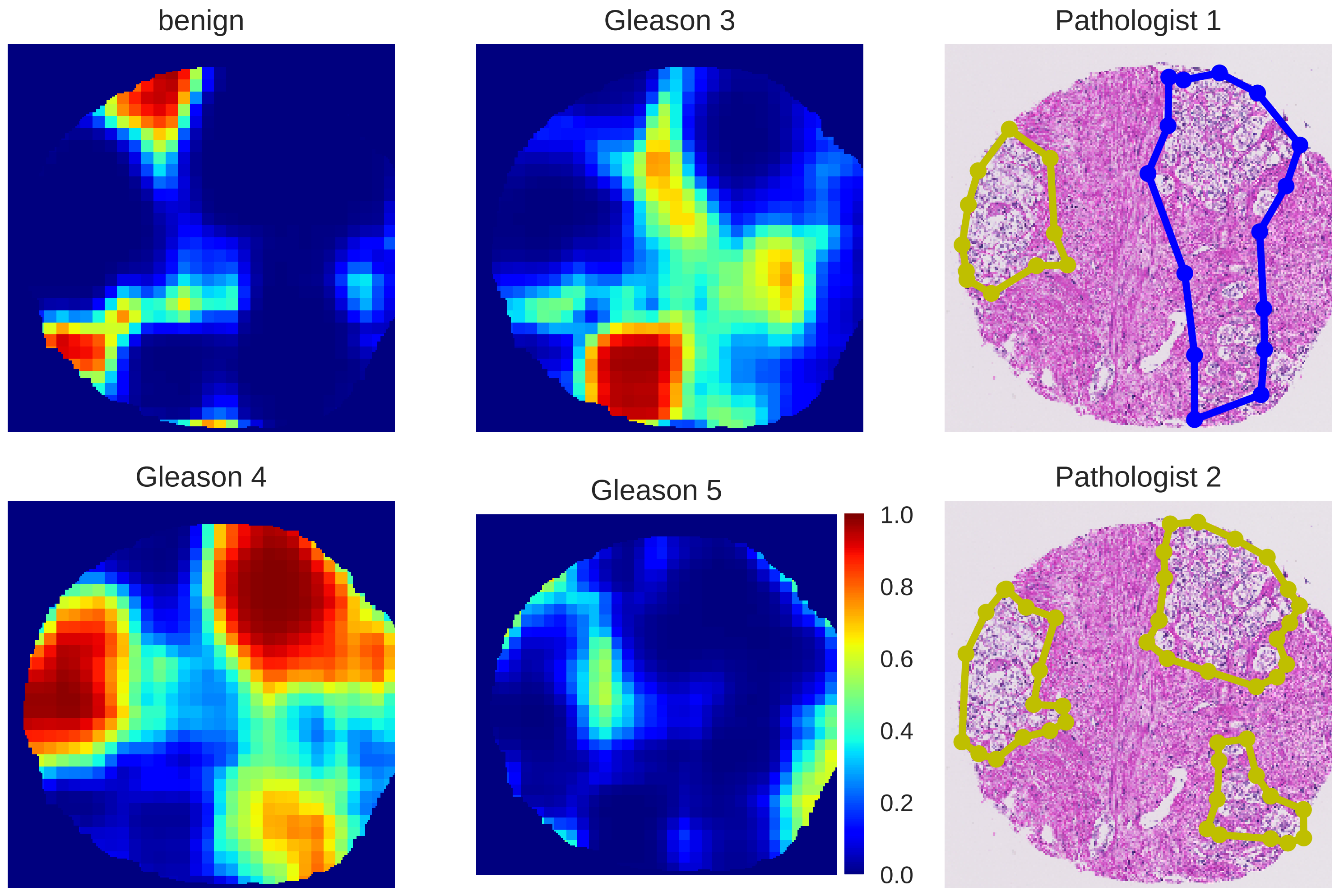

benign
Gleason 3
Pathologist 1
Gleason 4
Gleason 5
Pathologist 2
1.0
0.8
0.6
0.4
0.2
0.0

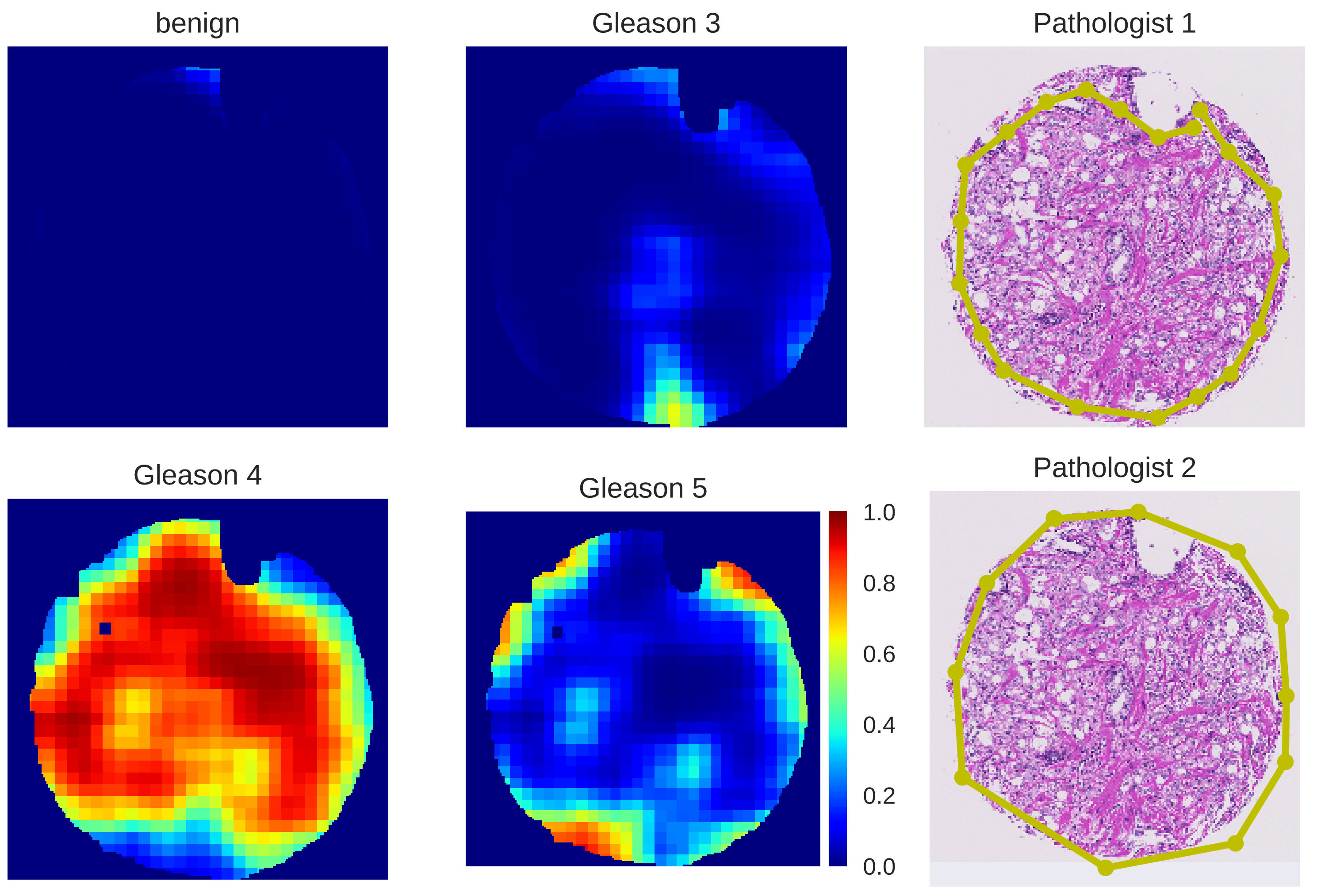
benign
Gleason 3
Pathologist 1
Gleason 4
Gleason 5
Pathologist 2
1.0
0.8
0.6
0.4
0.2
0.0

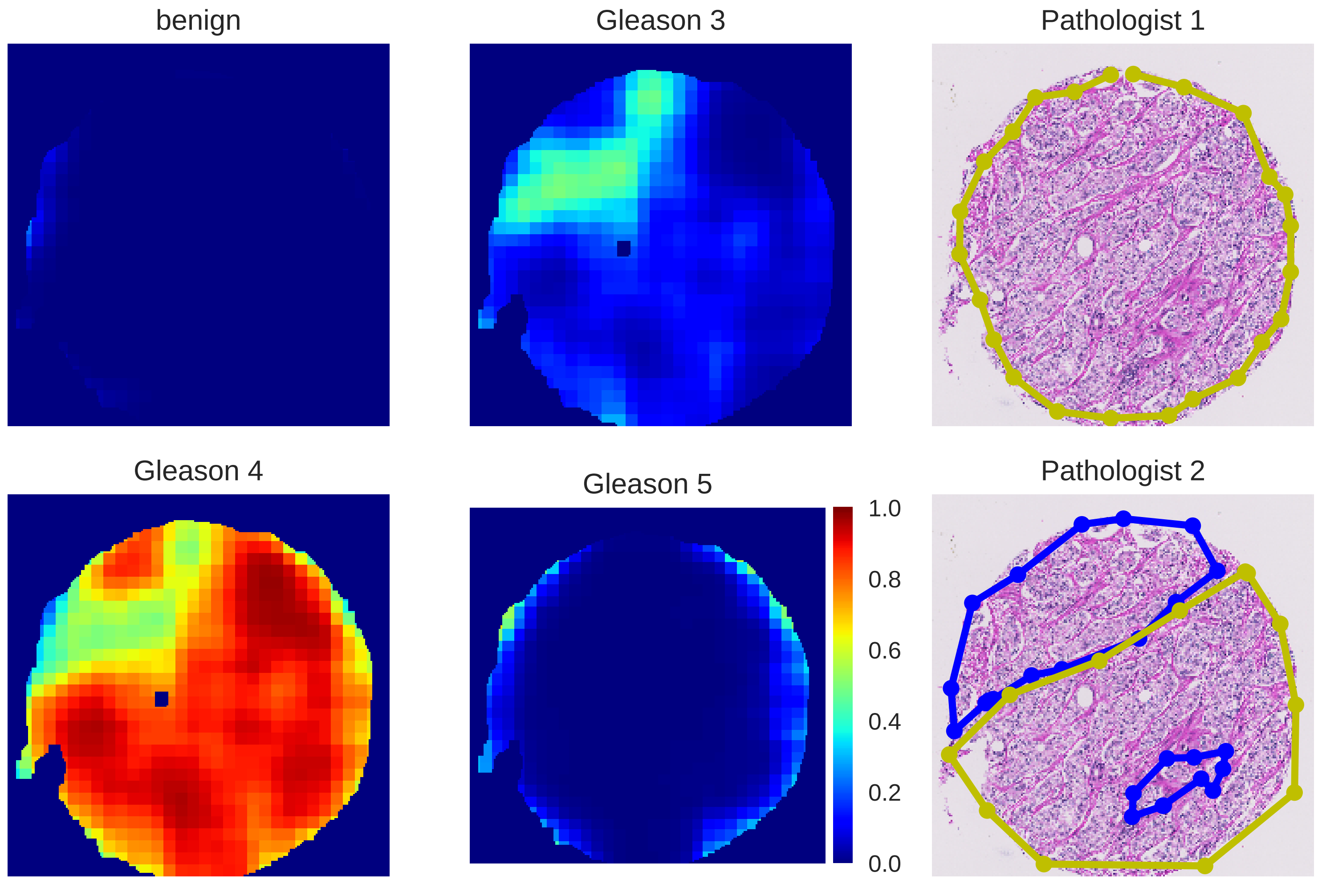
benign
Gleason 3
Pathologist 1
Gleason 4
Gleason 5
Pathologist 2
1.0
0.8
0.6
0.4
0.2
0.0

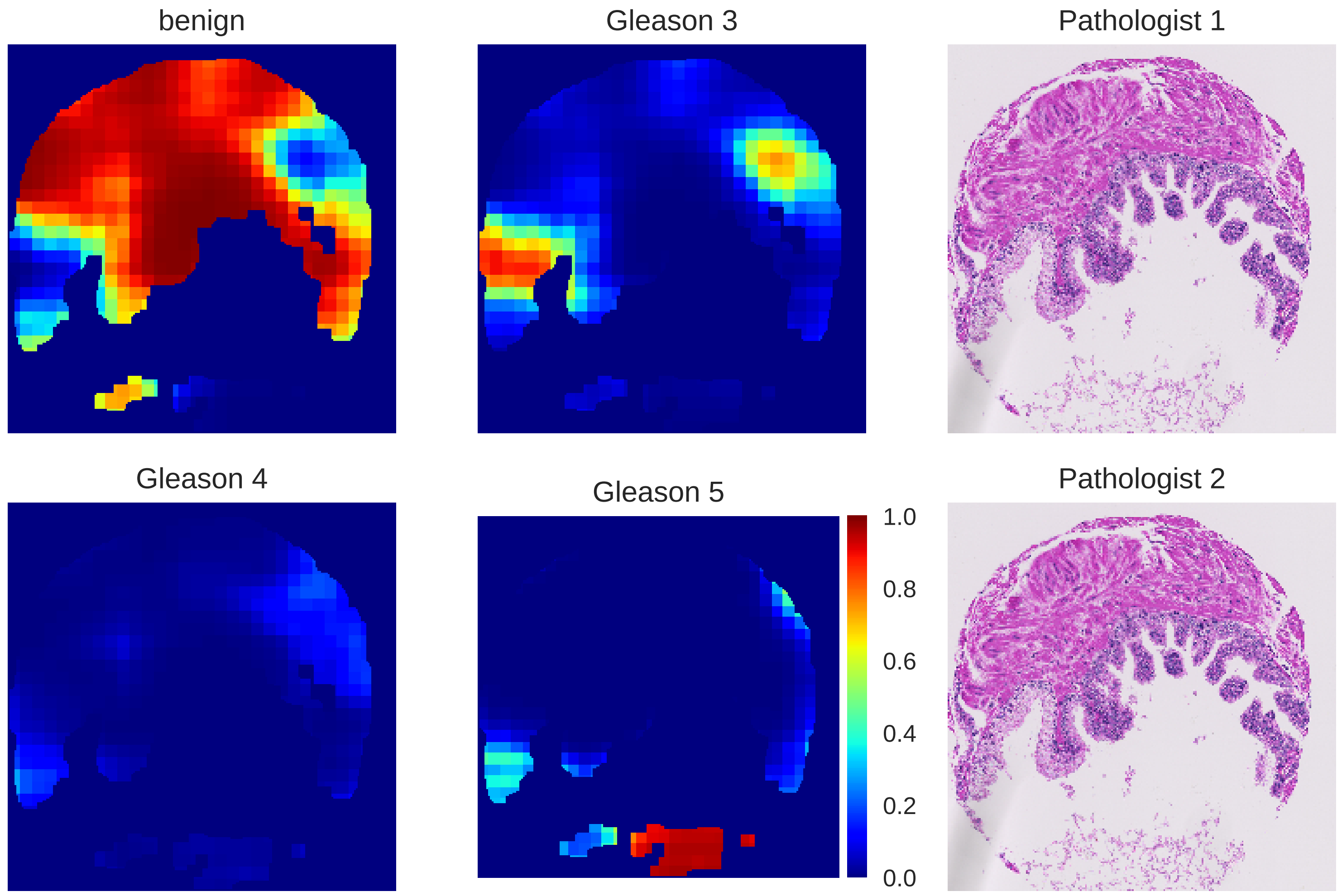
benign
Gleason 3
Pathologist 1
Gleason 4
Gleason 5
Pathologist 2
1.0
0.8
0.6
0.4
0.2
0.0

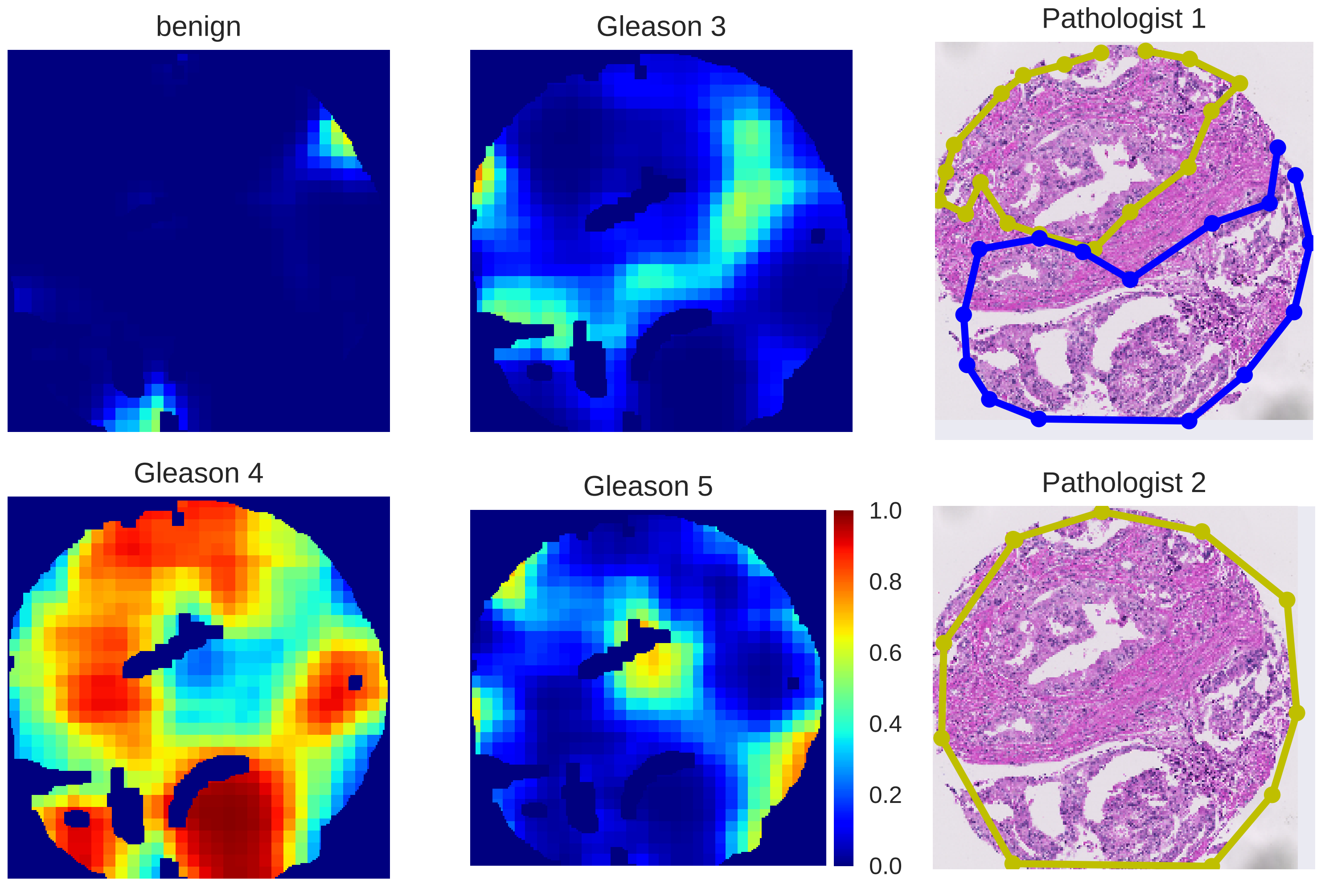

benign
Gleason 3
Pathologist 1
Gleason 4
Gleason 5
Pathologist 2
1.0
0.8
0.6
0.4
0.2
0.0

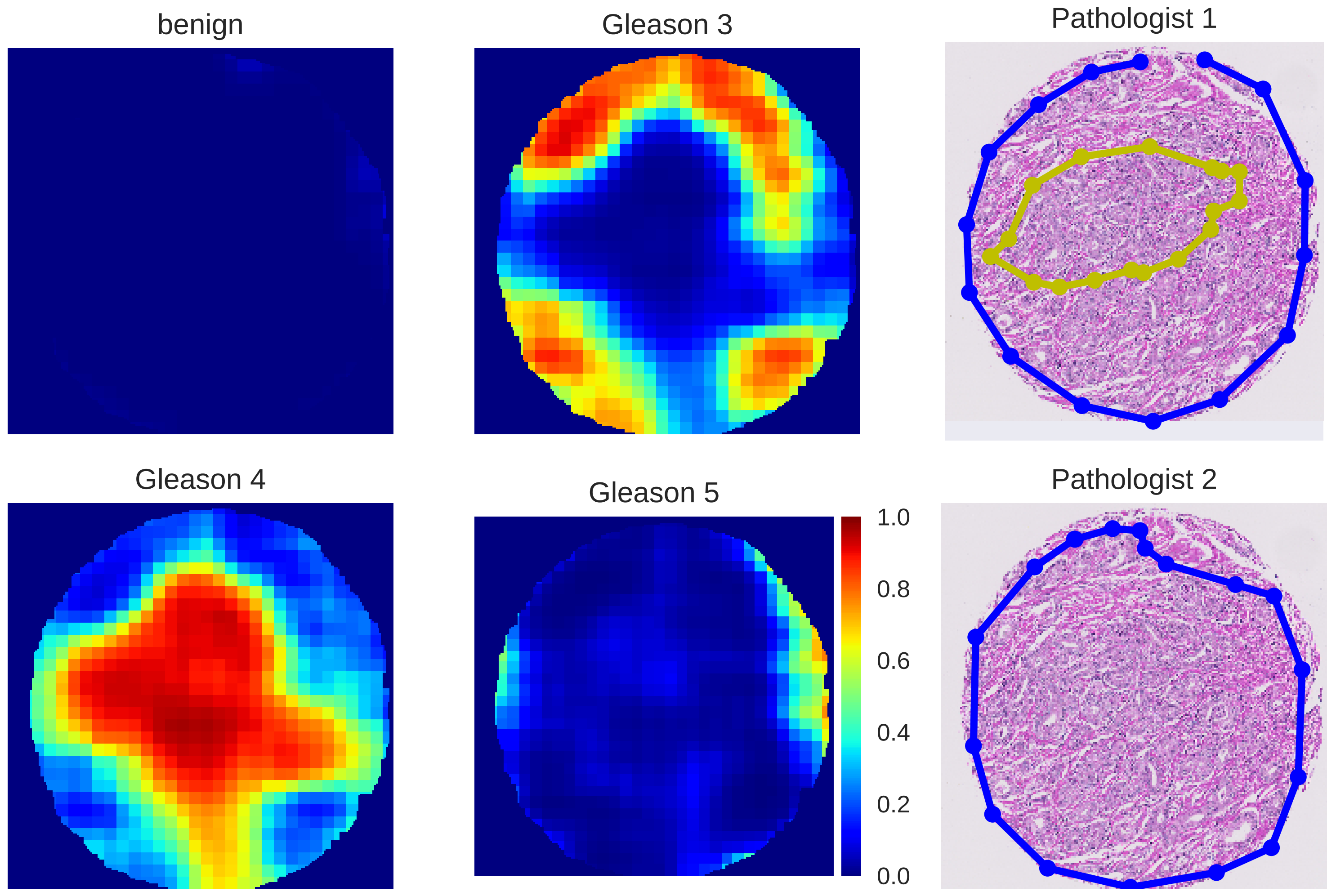
benign
Gleason 3
Pathologist 1
Gleason 4
Gleason 5
Pathologist 2
1.0
0.8
0.6
0.4
0.2
0.0

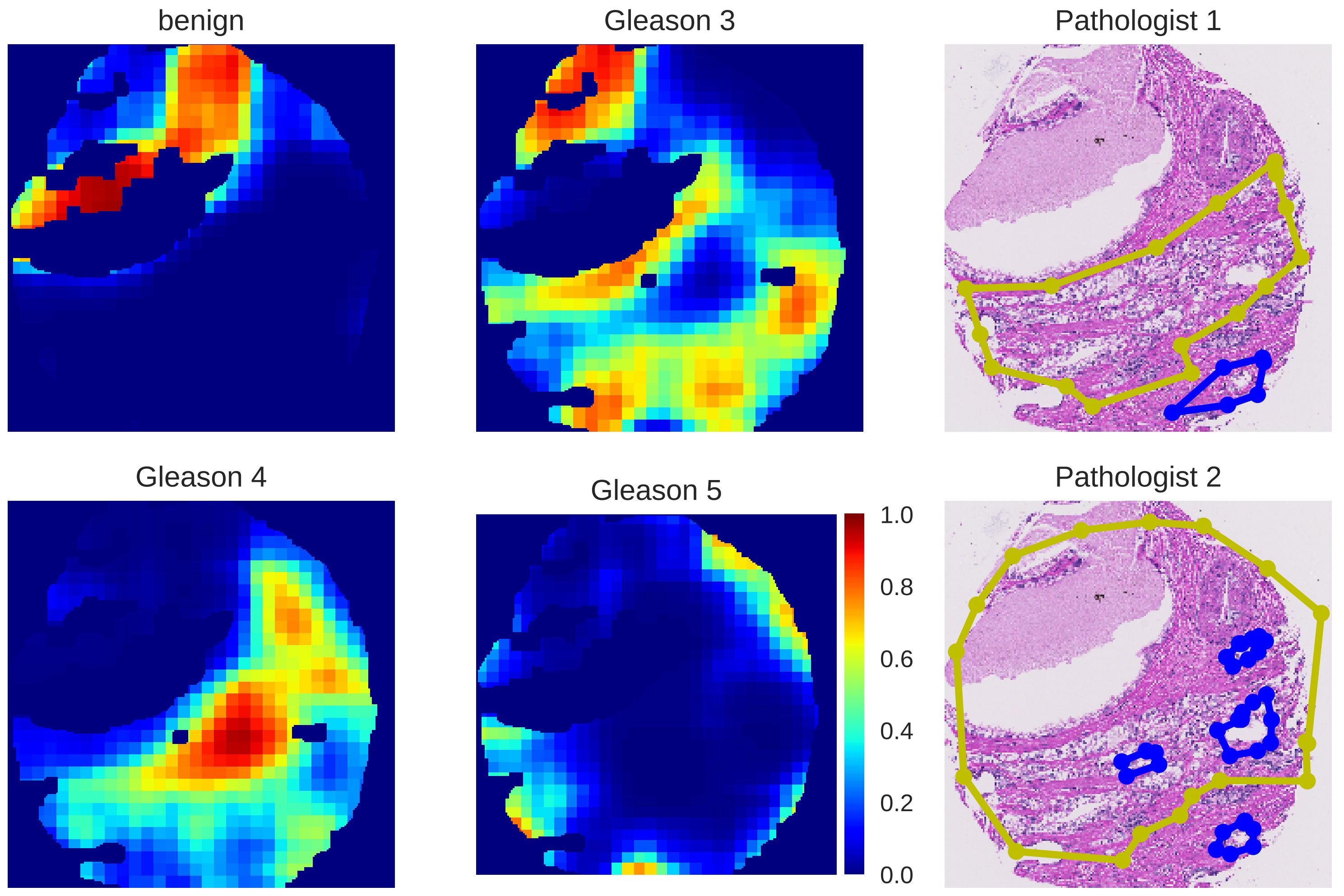

benign
Gleason 3
Pathologist 1
Gleason 4
Gleason 5
Pathologist 2
1.0
0.8
0.6
0.4
0.2
0.0

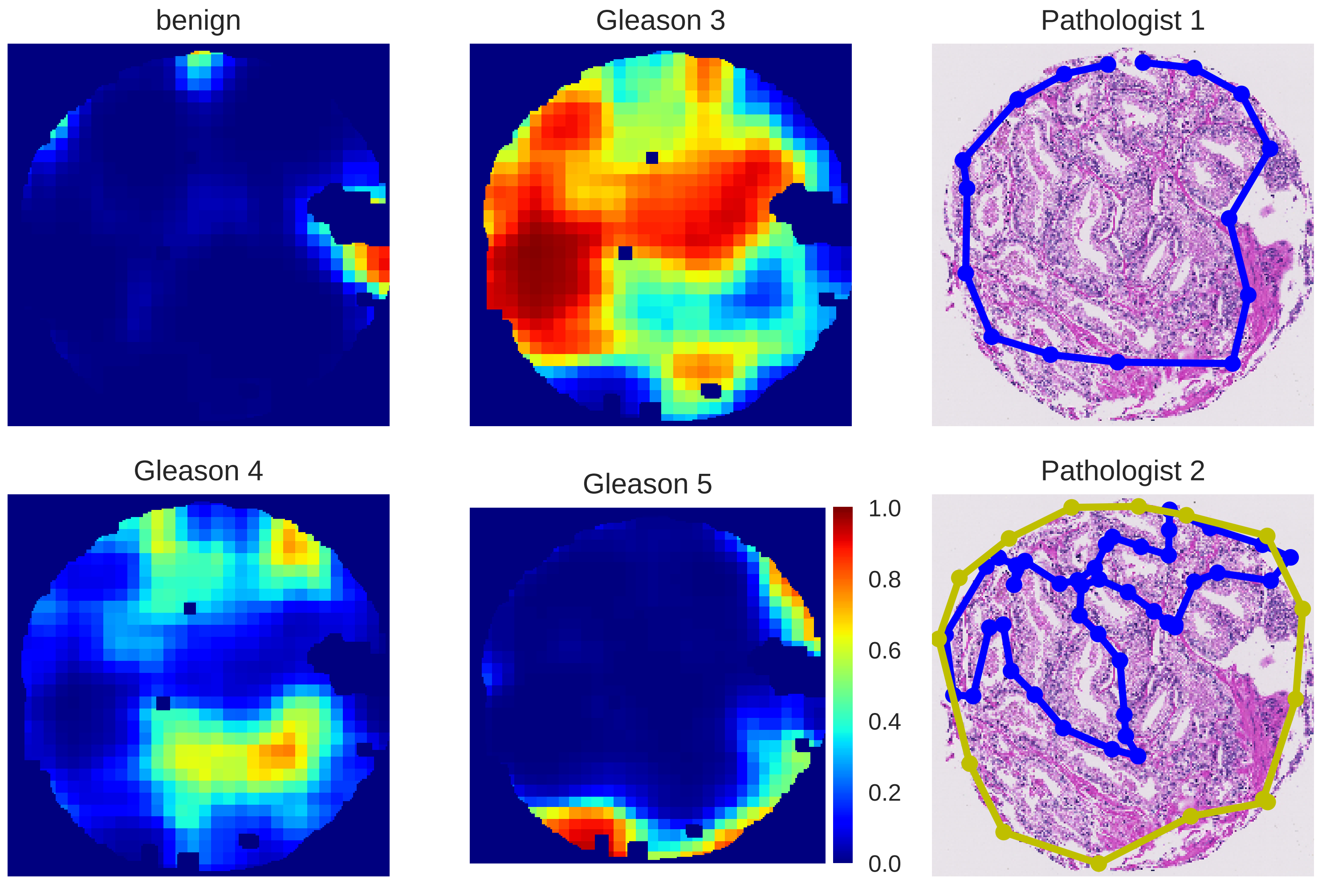
benign
Gleason 3
Pathologist 1
Gleason 4
Gleason 5
Pathologist 2
1.0
0.8
0.6
0.4
0.2
0.0

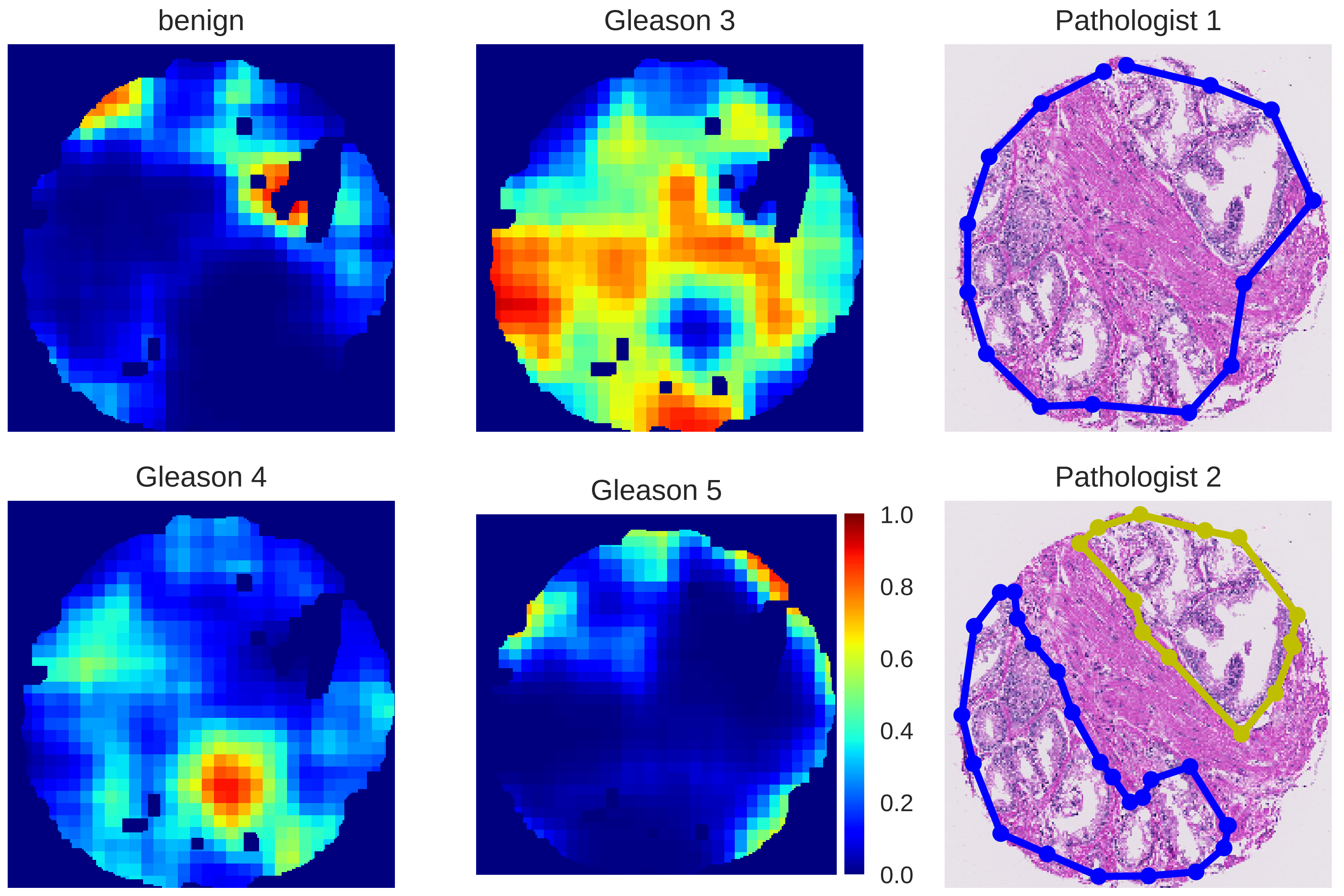

benign
Gleason 3
Pathologist 1
Gleason 4
Gleason 5
Pathologist 2
1.0
0.8
0.6
0.4
0.2
0.0

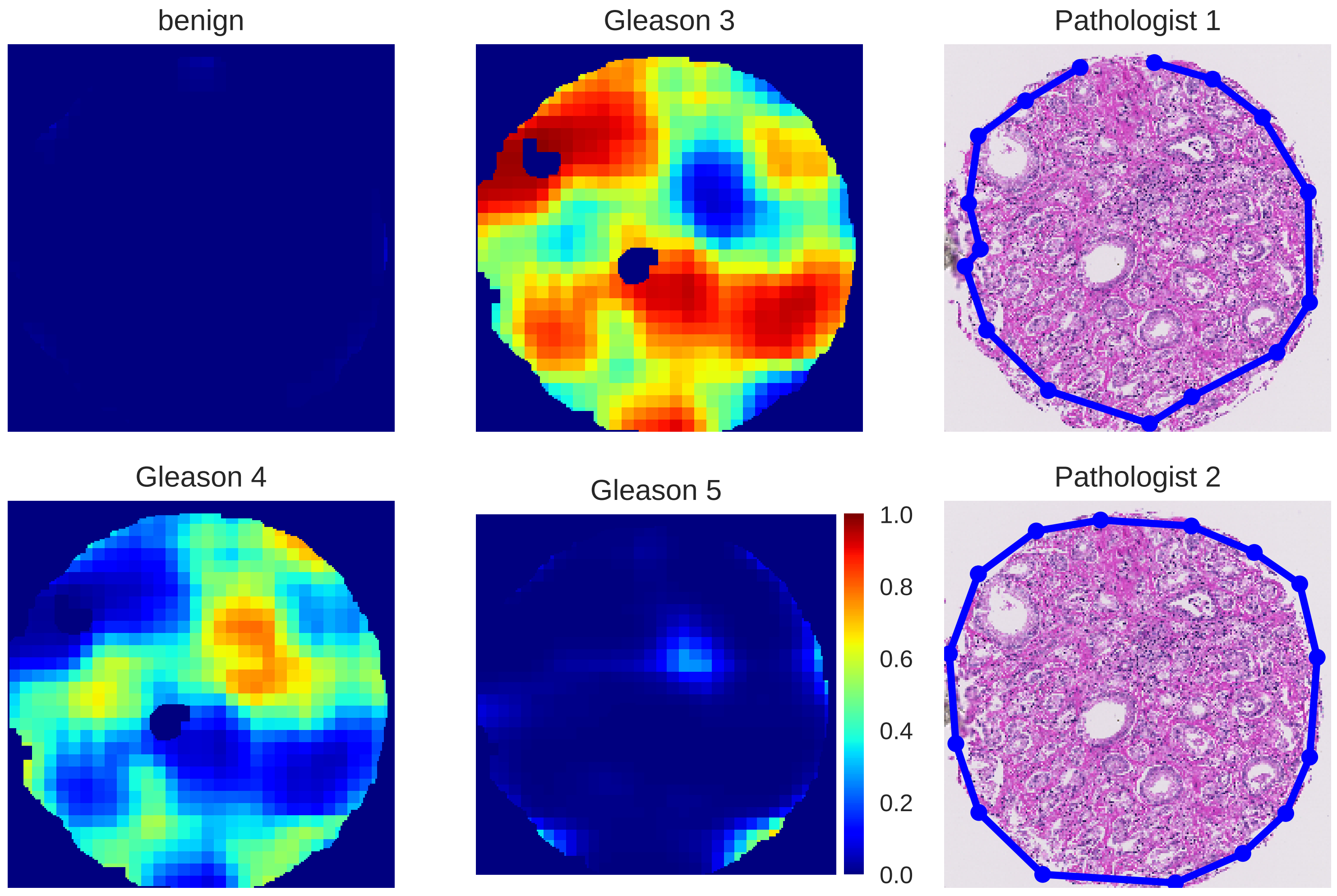
benign
Gleason 3
Pathologist 1
Gleason 4
Gleason 5
1.0
0.8
0.6
0.4
0.2
0.0
Pathologist 2

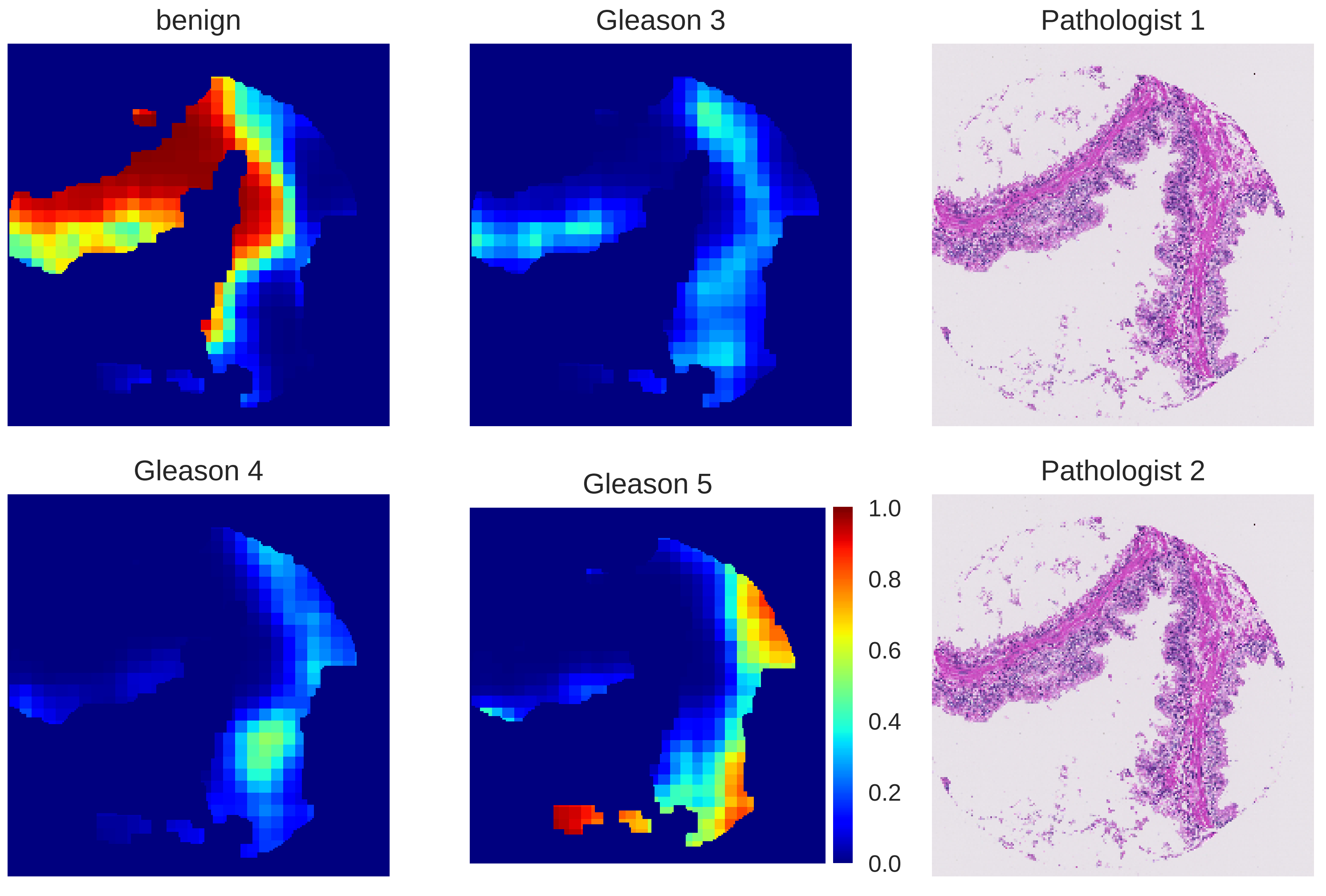

benign
Gleason 3
Pathologist 1
Gleason 4
Gleason 5
Pathologist 2
1.0
0.8
0.6
0.4
0.2
0.0

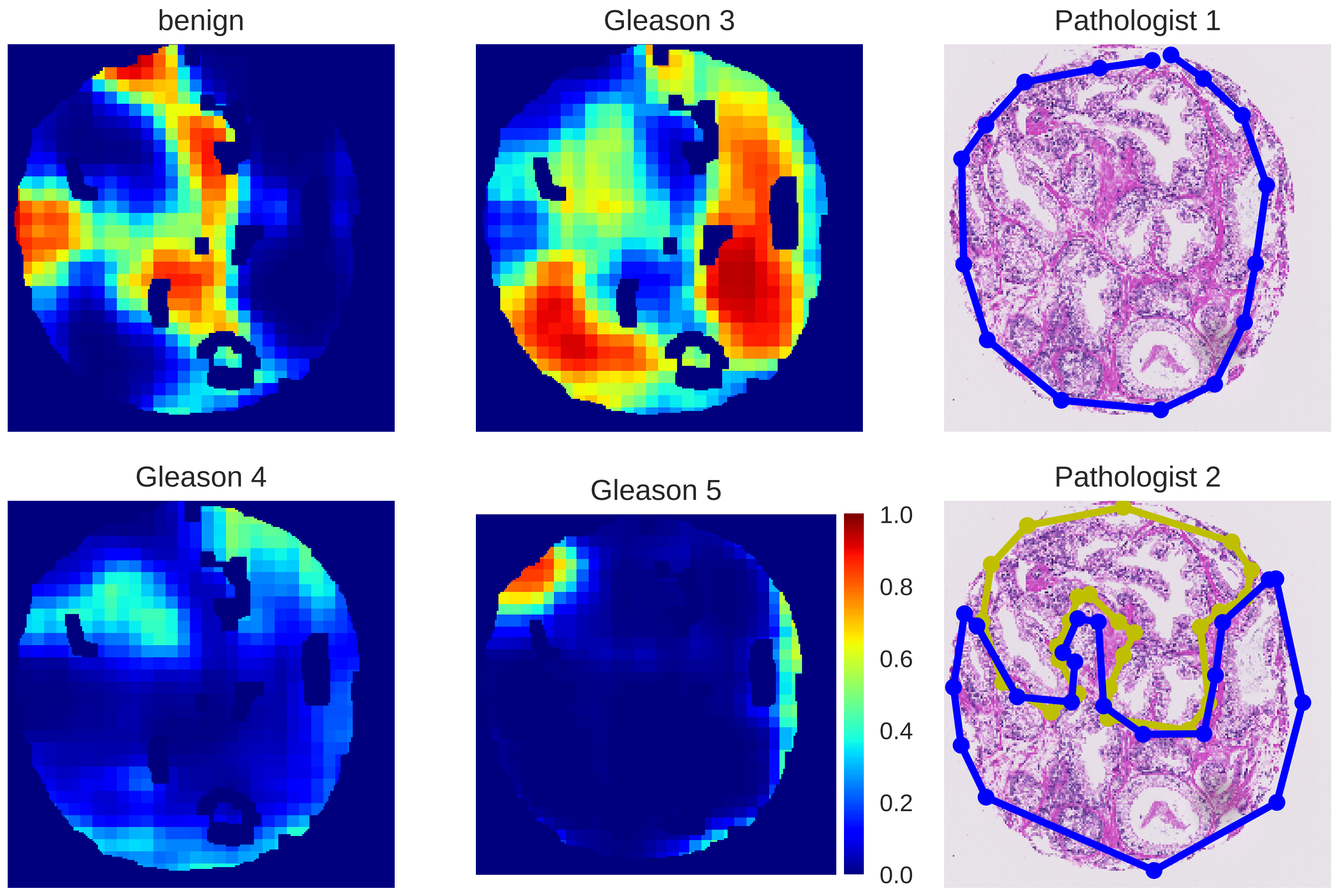

benign
Gleason 3
Pathologist 1
Gleason 4
Gleason 5
Pathologist 2
1.0
0.8
0.6
0.4
0.2
0.0

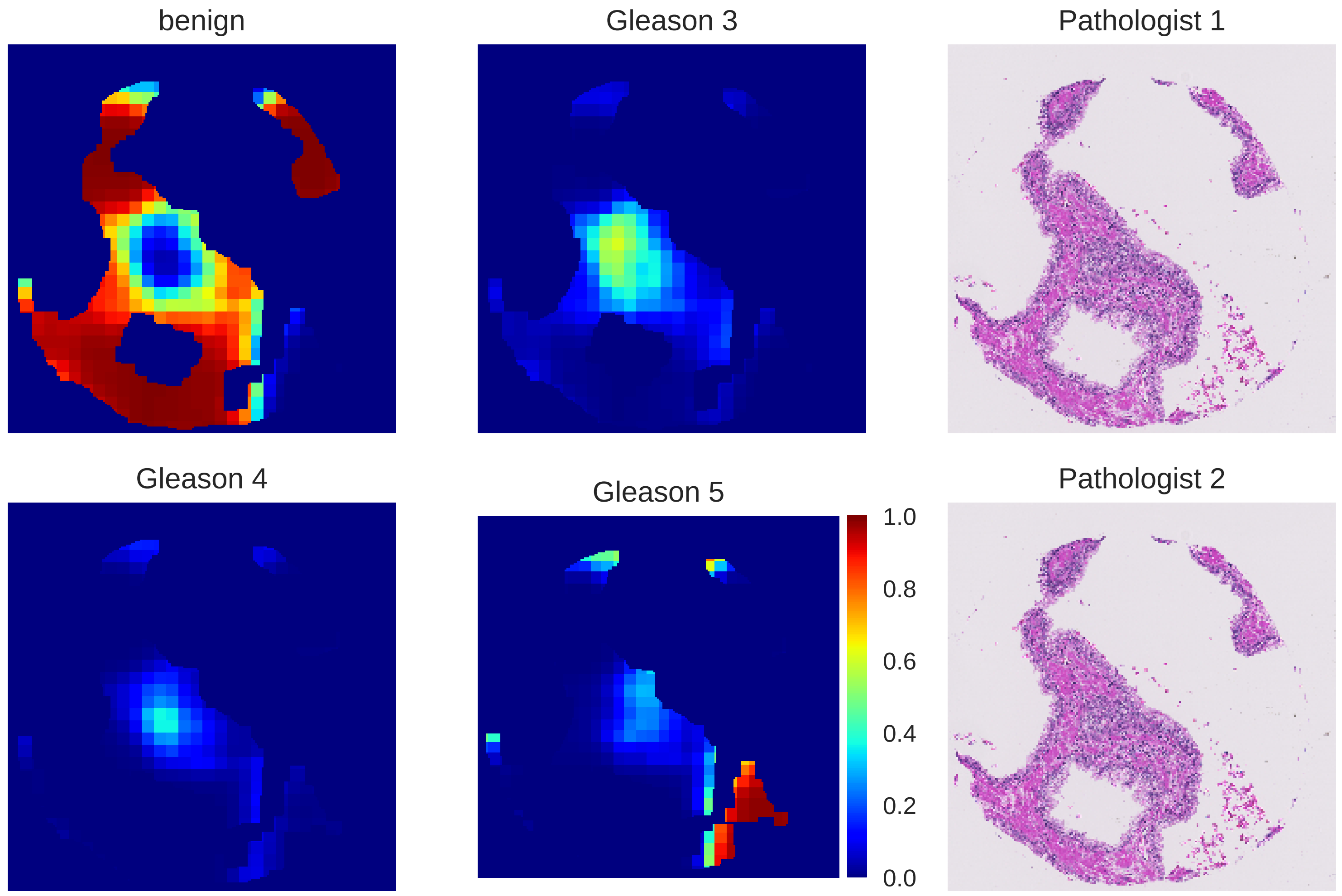

benign
Gleason 3
Pathologist 1
Gleason 4
Gleason 5
Pathologist 2
1.0
0.8
0.6
0.4
0.2
0.0

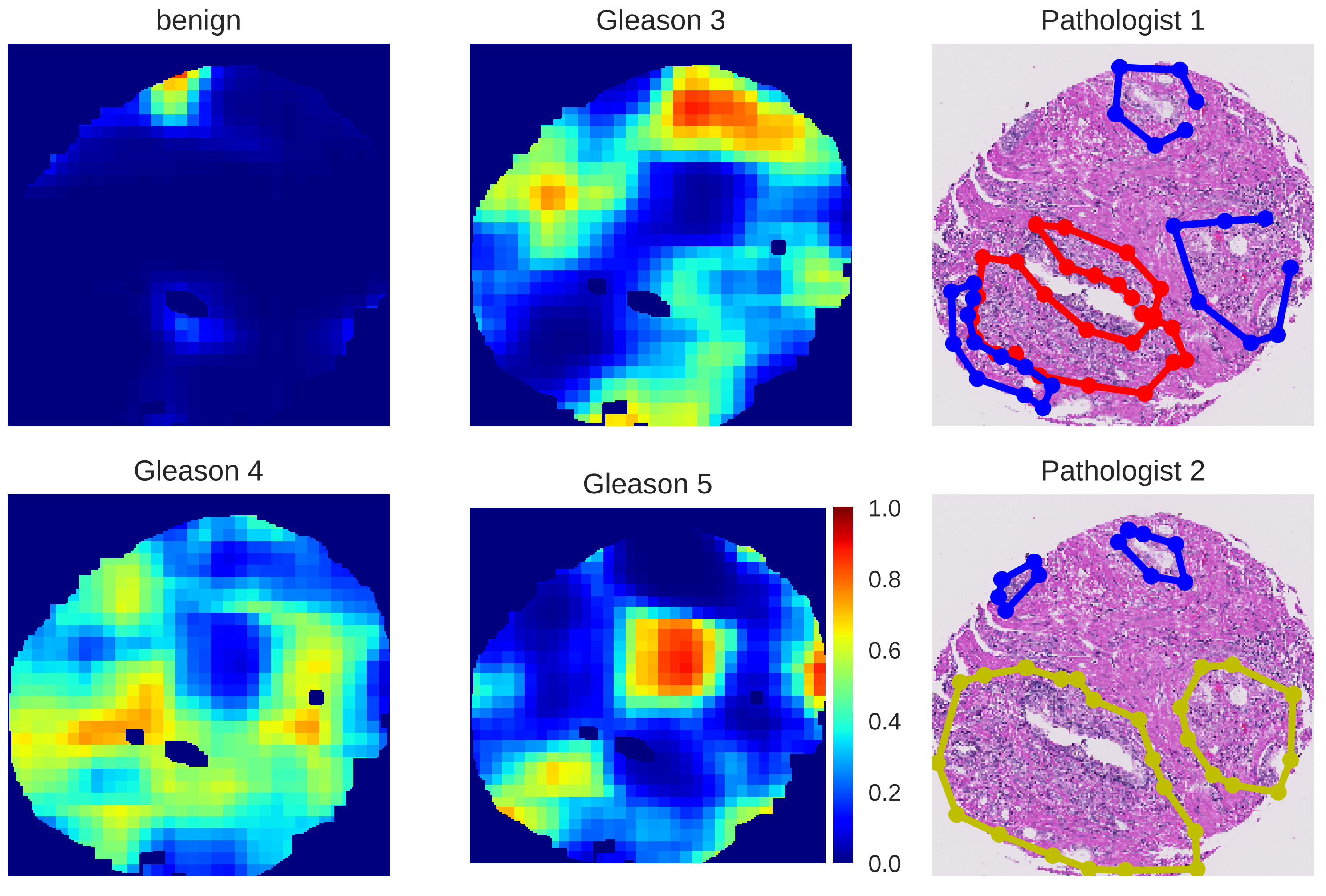

benign
Gleason 3
Pathologist 1
Gleason 4
Gleason 5
Pathologist 2
1.0
0.8
0.6
0.4
0.2
0.0

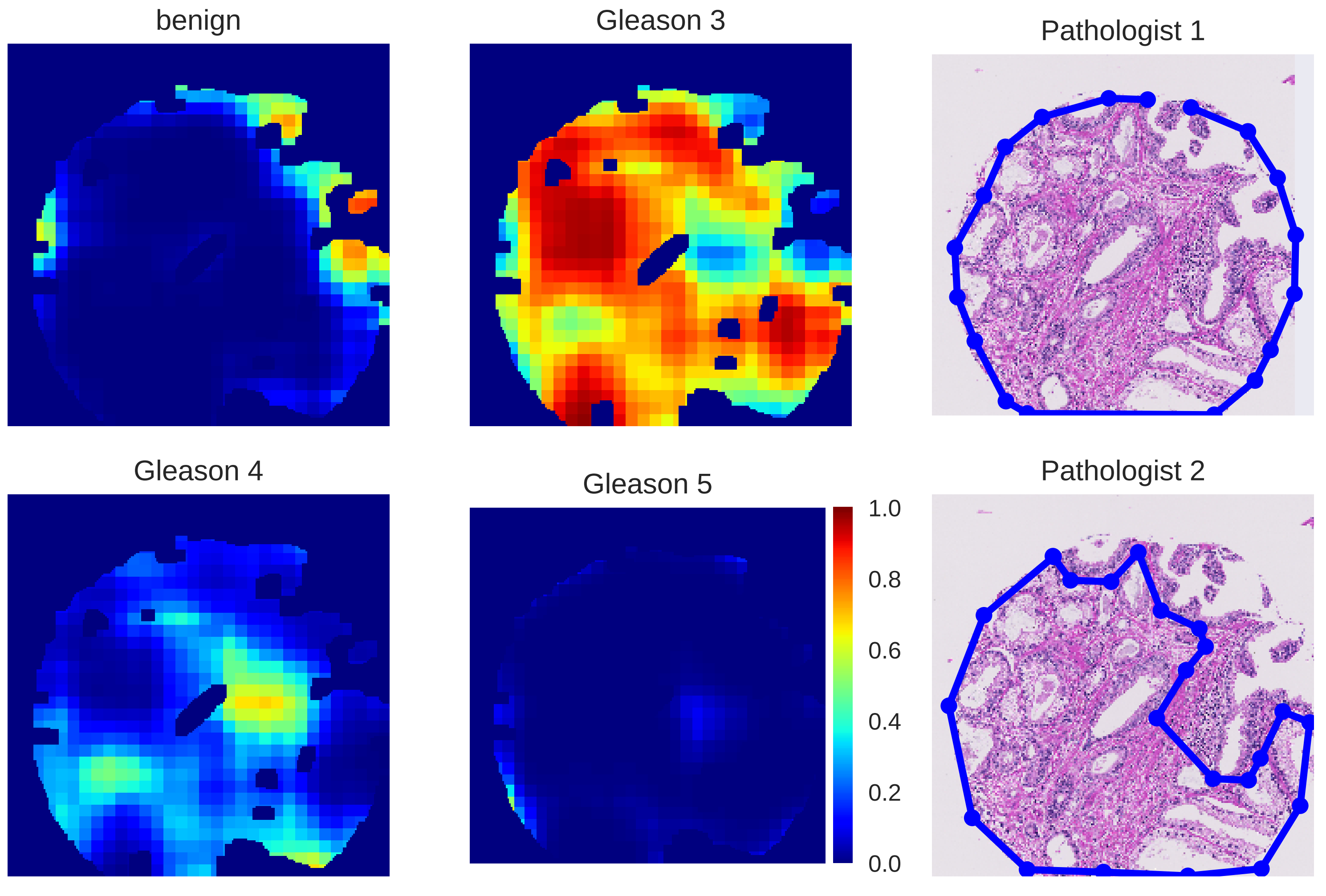
benign
Gleason 3
Pathologist 1
Gleason 4
Gleason 5
1.0
0.8
0.6
0.4
0.2
0.0
Pathologist 2

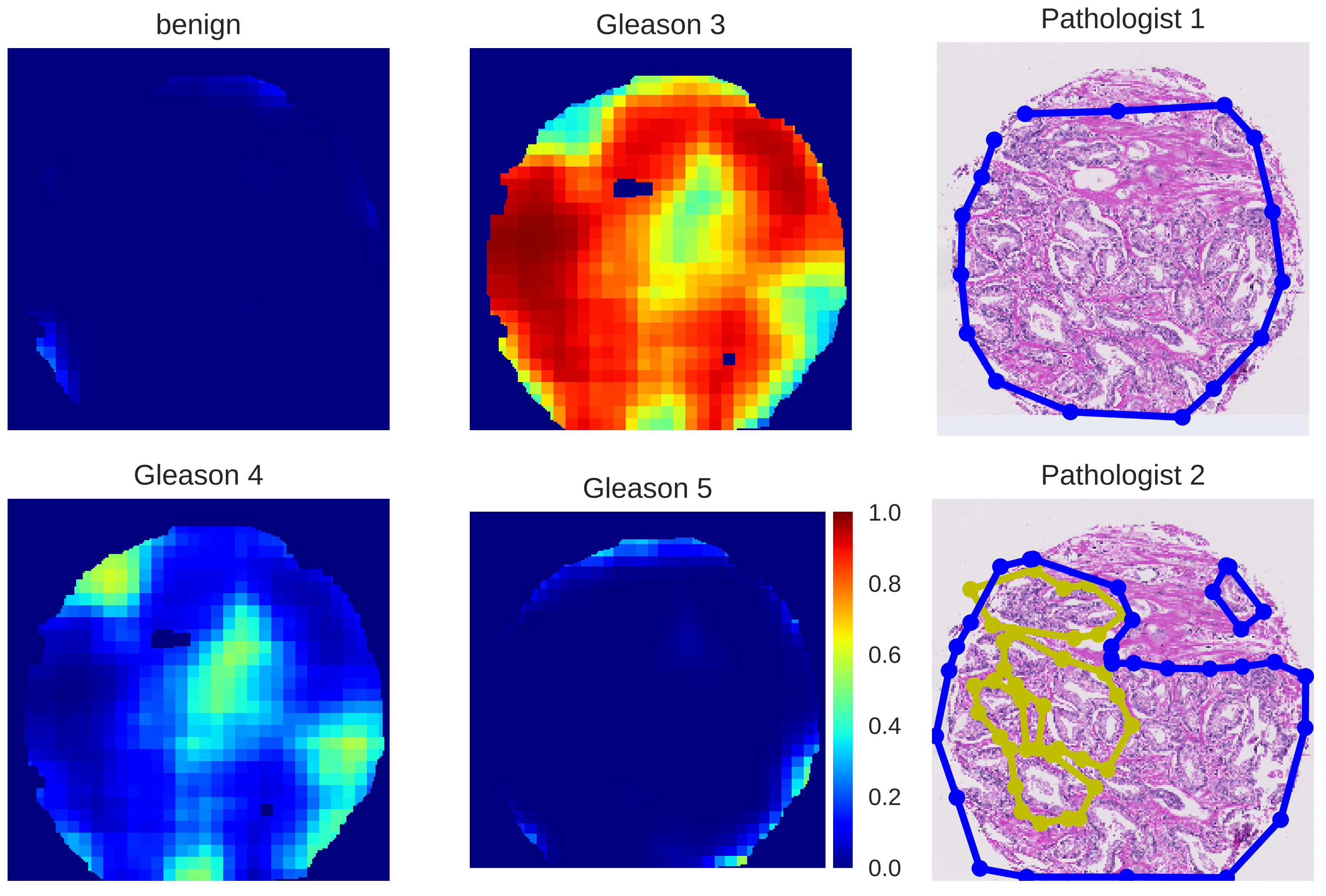

benign
Gleason 3
Pathologist 1
Gleason 4
Gleason 5
Pathologist 2
1.0
0.8
0.6
0.4
0.2
0.0

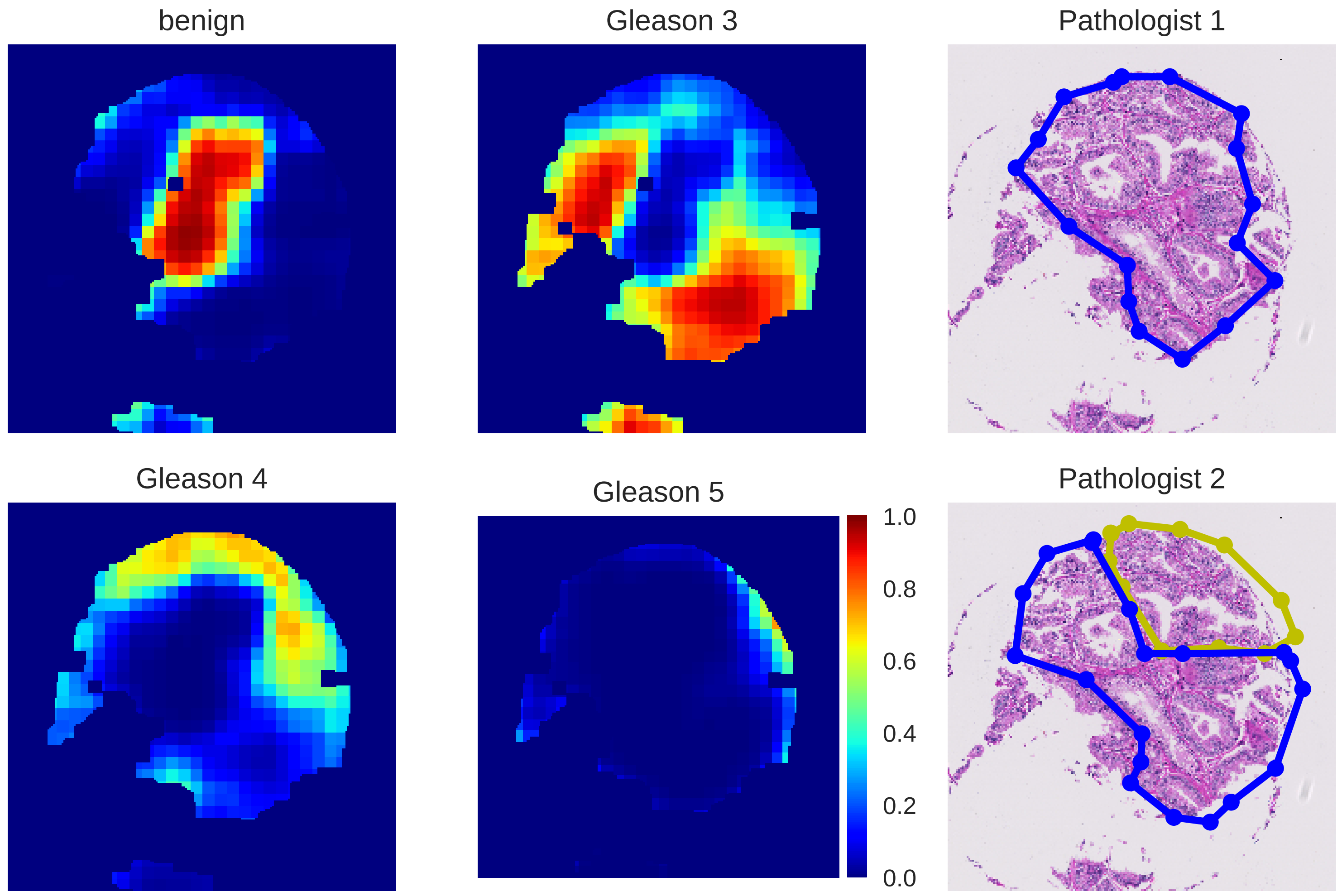

benign
Gleason 3
Pathologist 1
Gleason 4
Gleason 5
Pathologist 2
1.0
0.8
0.6
0.4
0.2
0.0

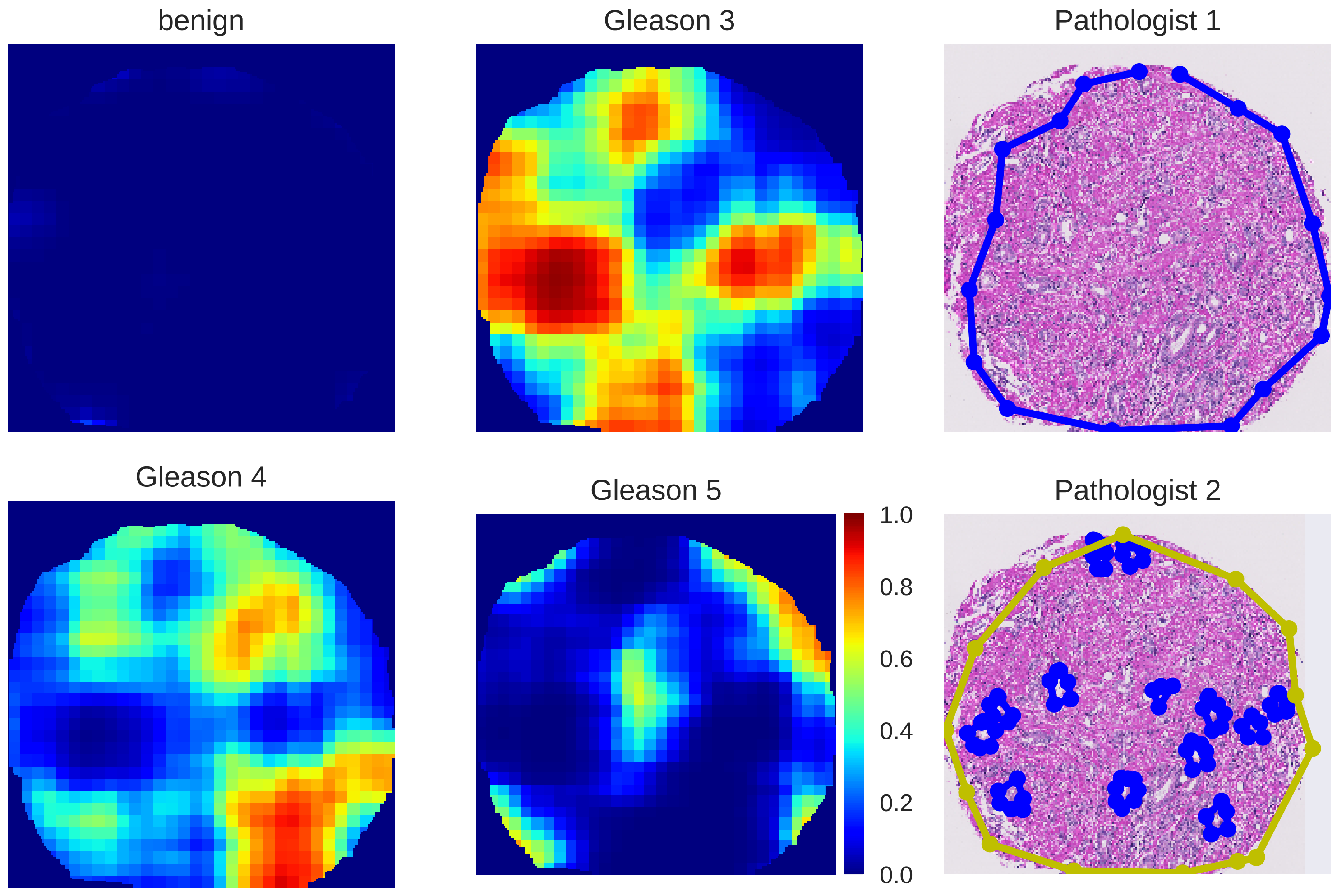
benign
Gleason 3
Pathologist 1
Gleason 4
Gleason 5
Pathologist 2
1.0
0.8
0.6
0.4
0.2
0.0

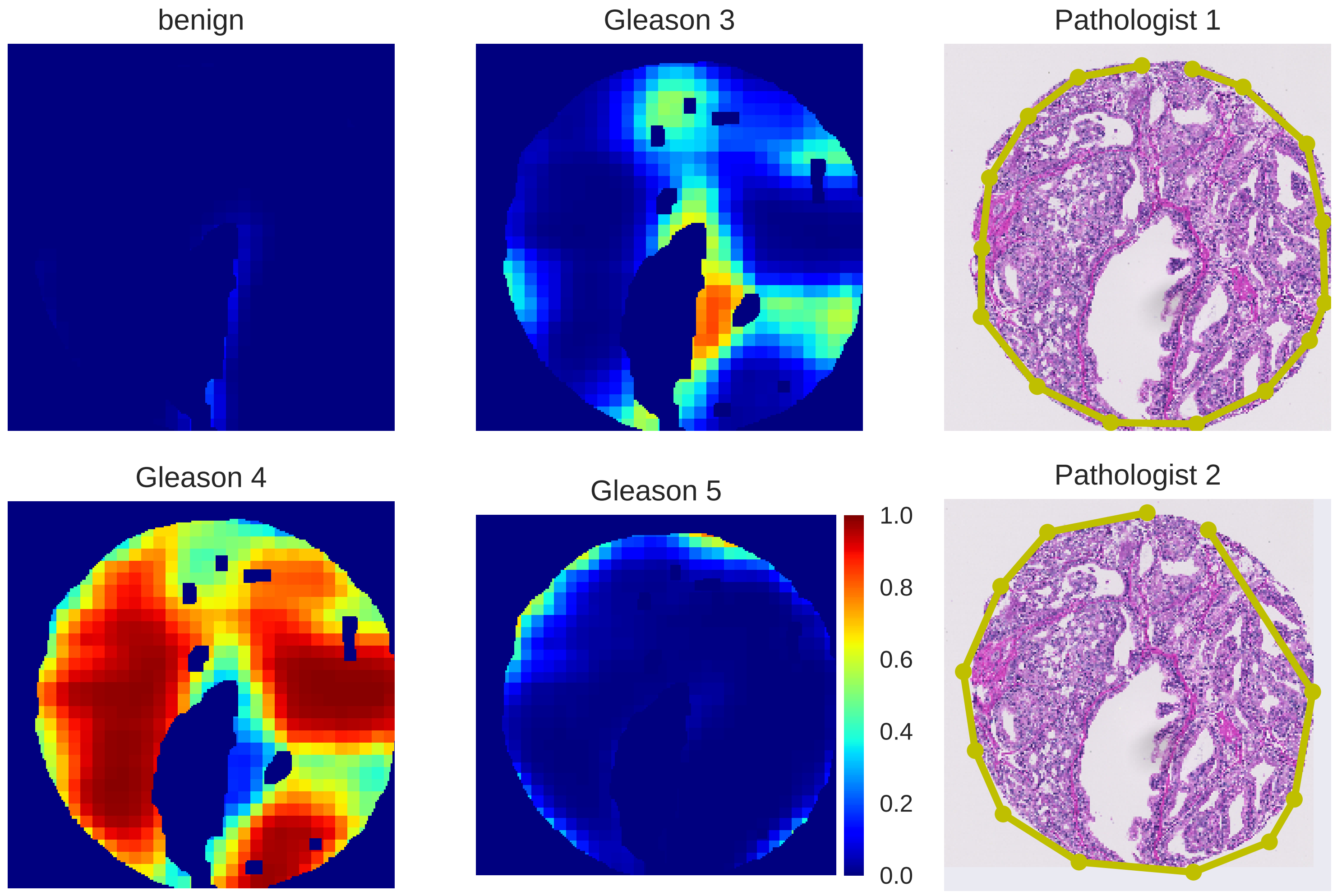
benign
Gleason 3
Pathologist 1
Gleason 4
Gleason 5
Pathologist 2
1.0
0.8
0.6
0.4
0.2
0.0

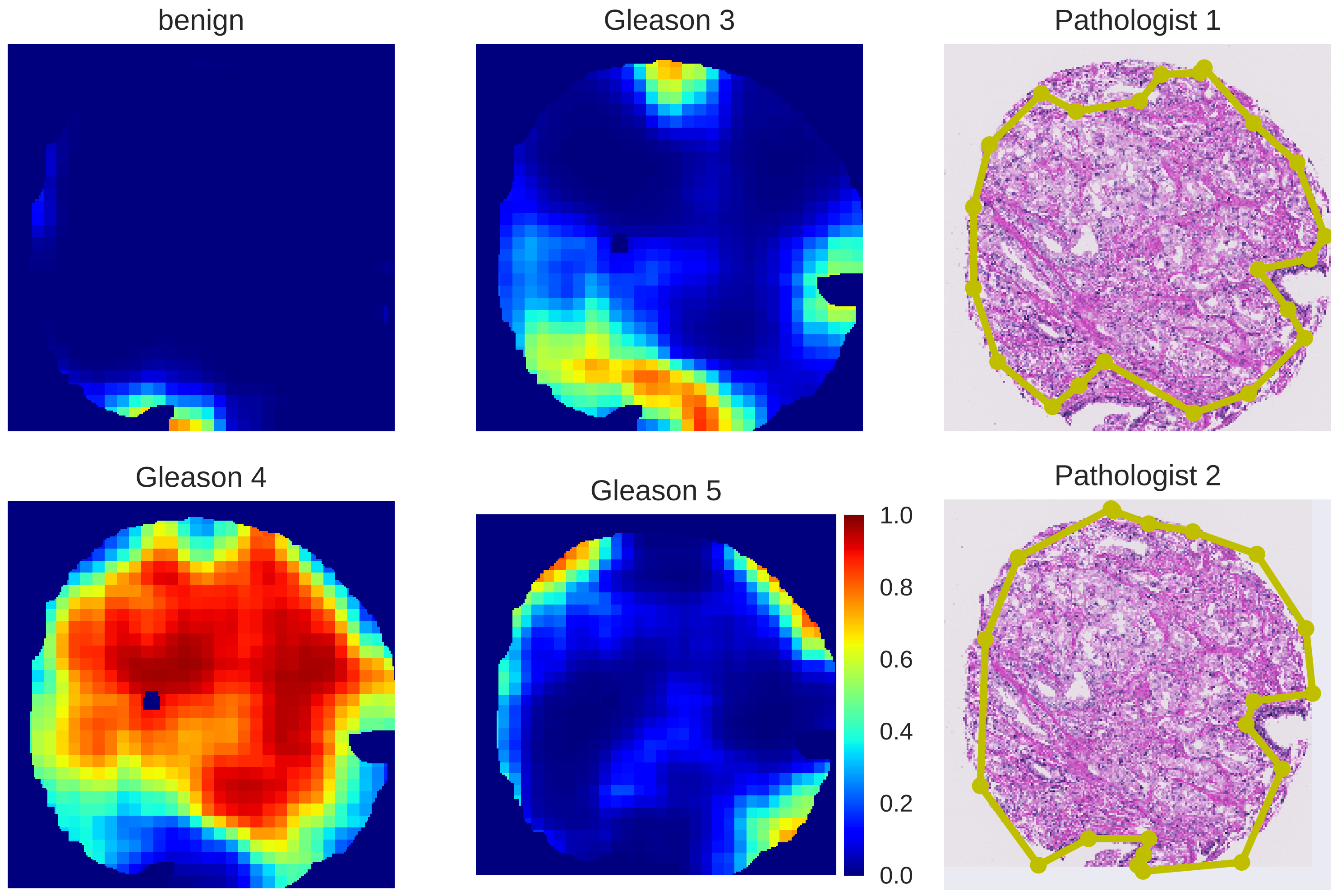

benign
Gleason 3
Pathologist 1
Gleason 4
Gleason 5
Pathologist 2
1.0
0.8
0.6
0.4
0.2
0.0

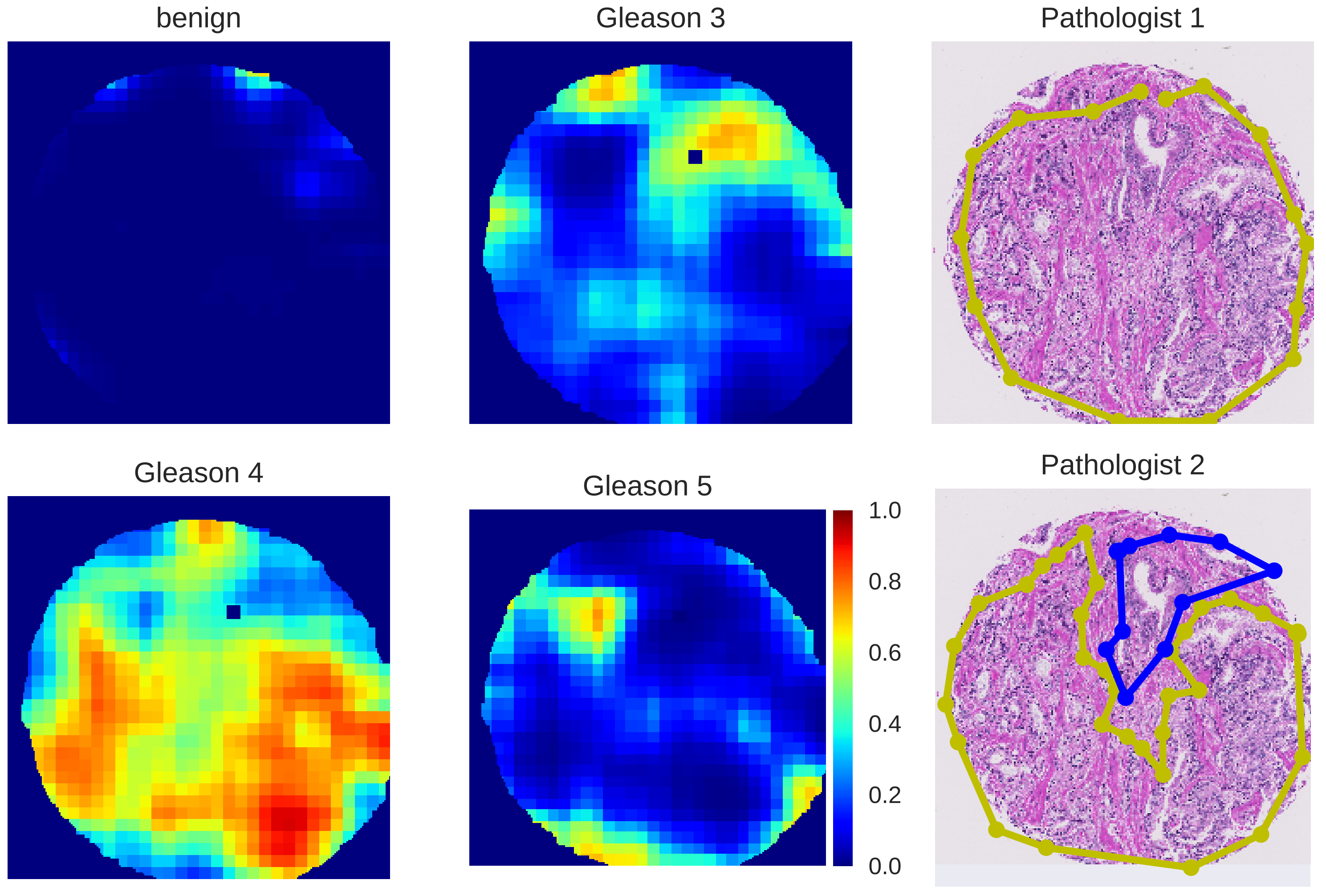
benign
Gleason 3
Pathologist 1
Gleason 4
Gleason 5
Pathologist 2
1.0
0.8
0.6
0.4
0.2
0.0

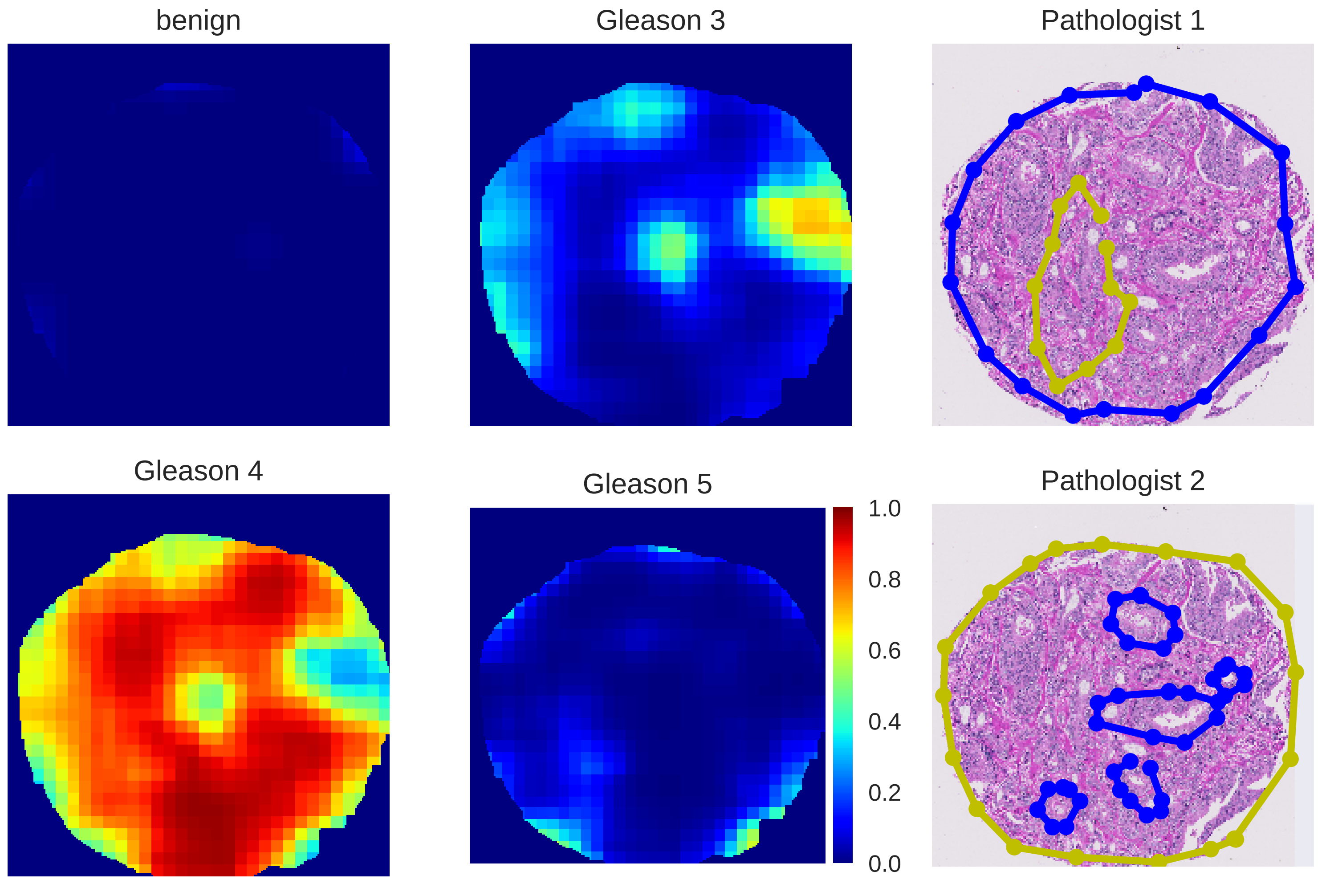

benign
Gleason 3
Pathologist 1
Gleason 4
Gleason 5
Pathologist 2
1.0
0.8
0.6
0.4
0.2
0.0

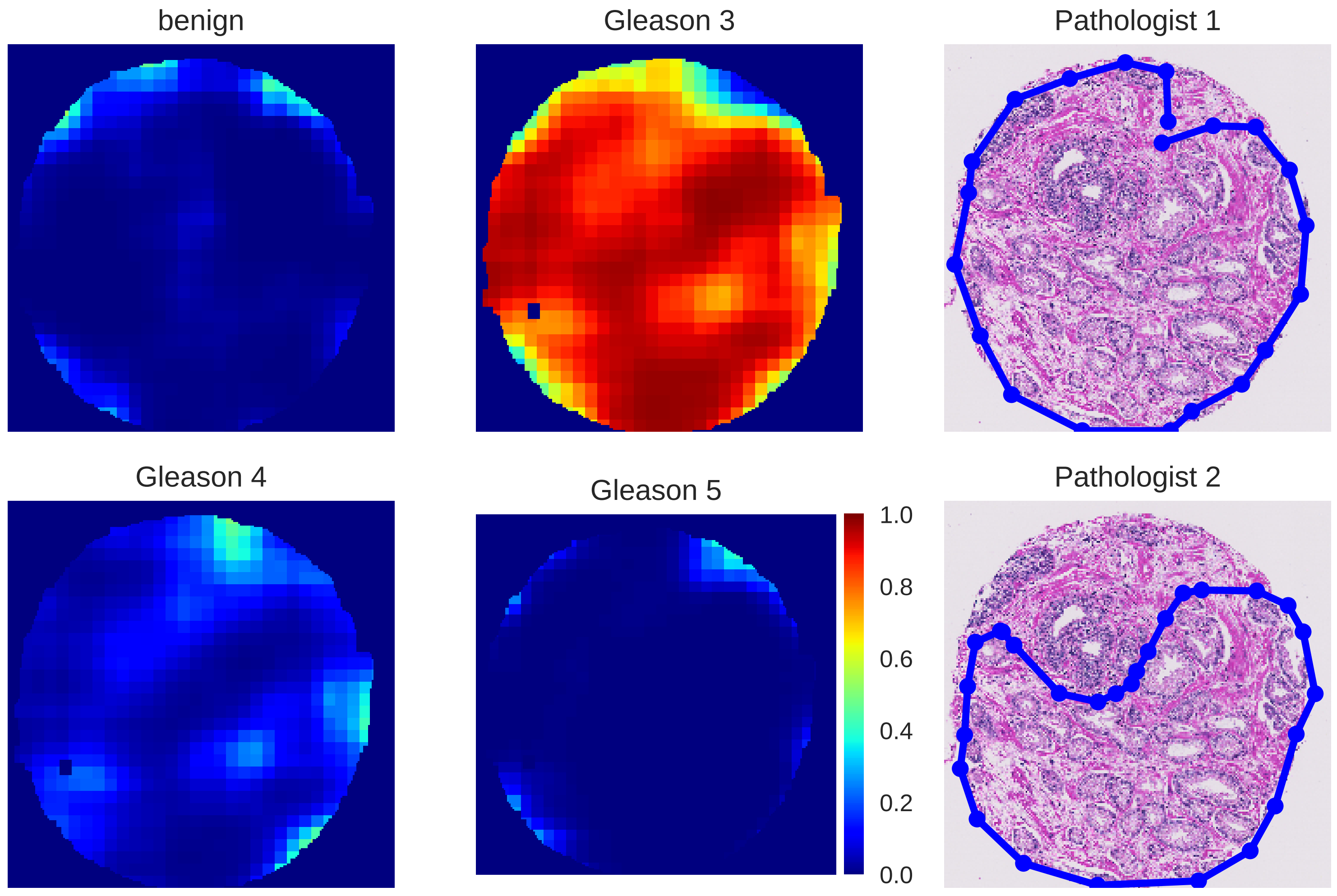

benign
Gleason 3
Pathologist 1
Gleason 4
Gleason 5
Pathologist 2
1.0
0.8
0.6
0.4
0.2
0.0

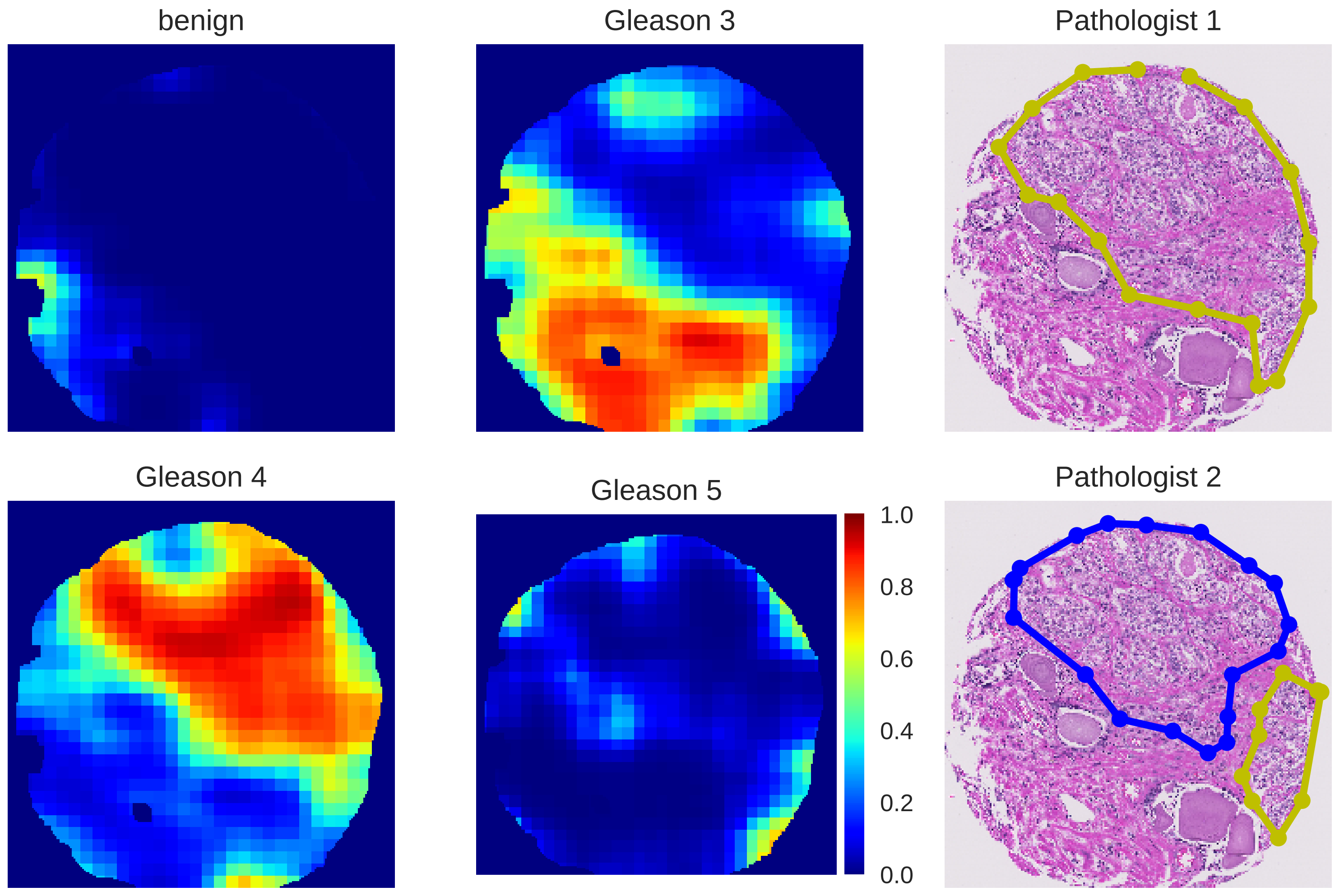

benign
Gleason 3
Pathologist 1
Gleason 4
Gleason 5
Pathologist 2
1.0
0.8
0.6
0.4
0.2
0.0

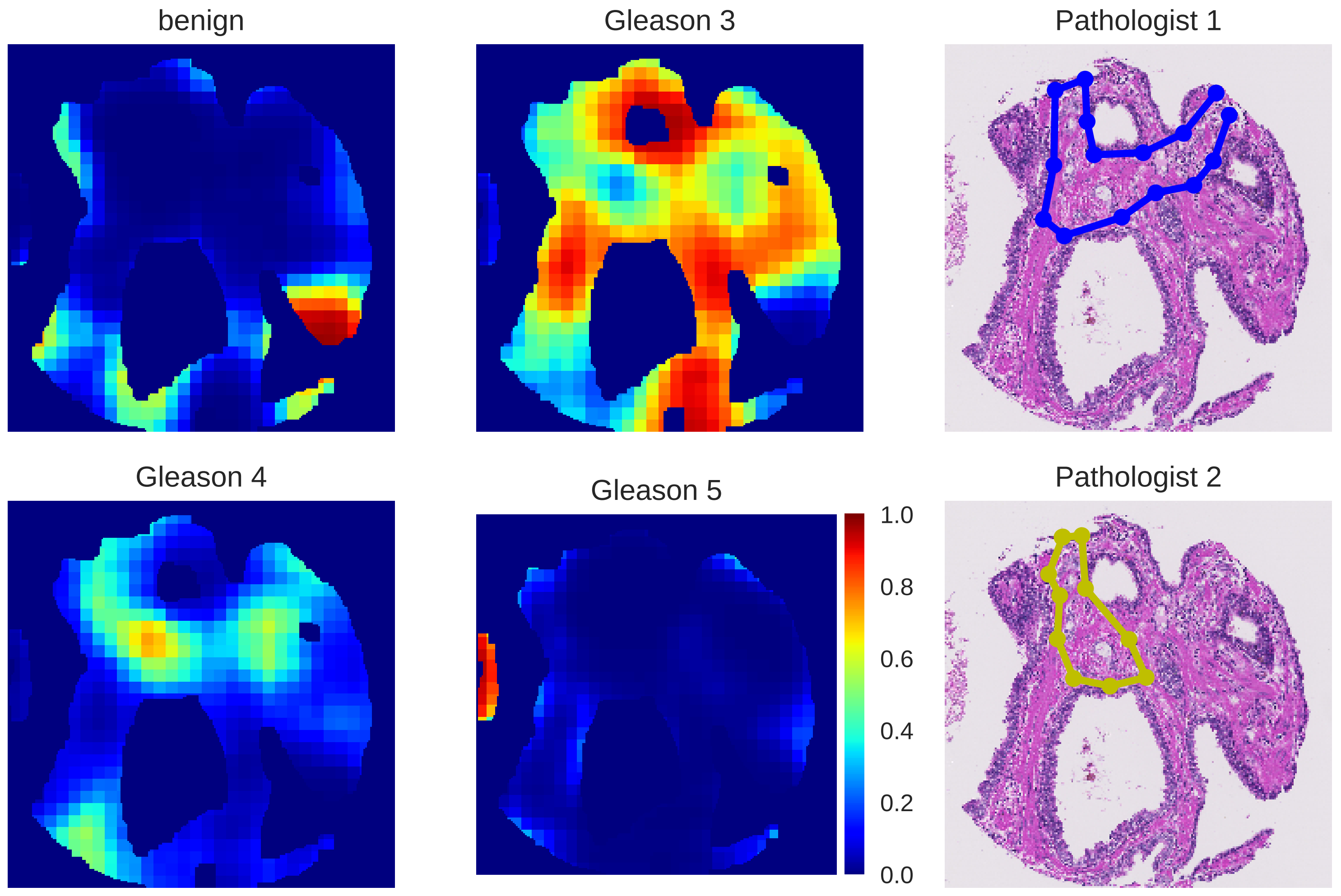
benign
Gleason 3
Pathologist 1
Gleason 4
Gleason 5
Pathologist 2
1.0
0.8
0.6
0.4
0.2
0.0

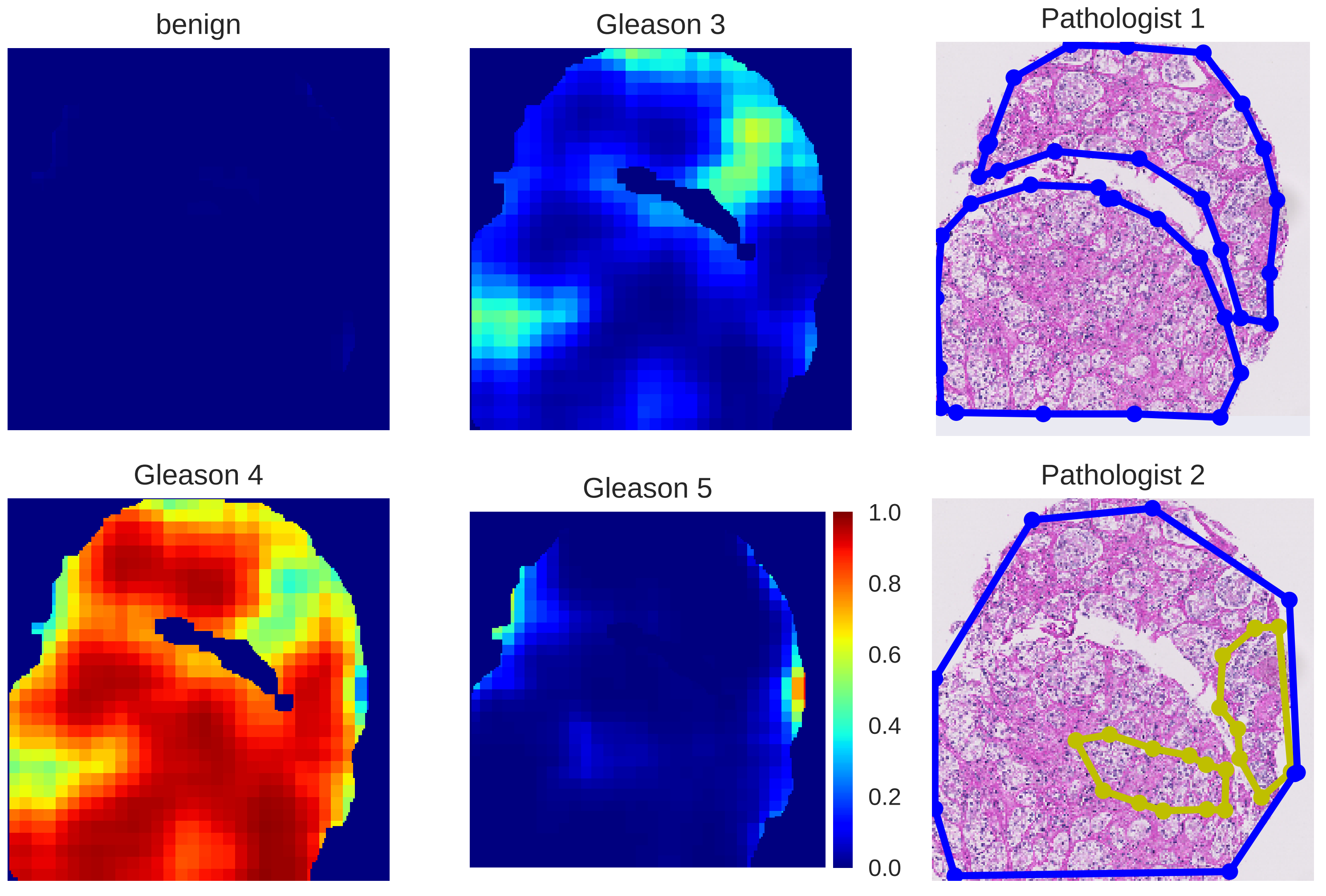
benign
Gleason 3
Pathologist 1
Gleason 4
Gleason 5
Pathologist 2
1.0
0.8
0.6
0.4
0.2
0.0

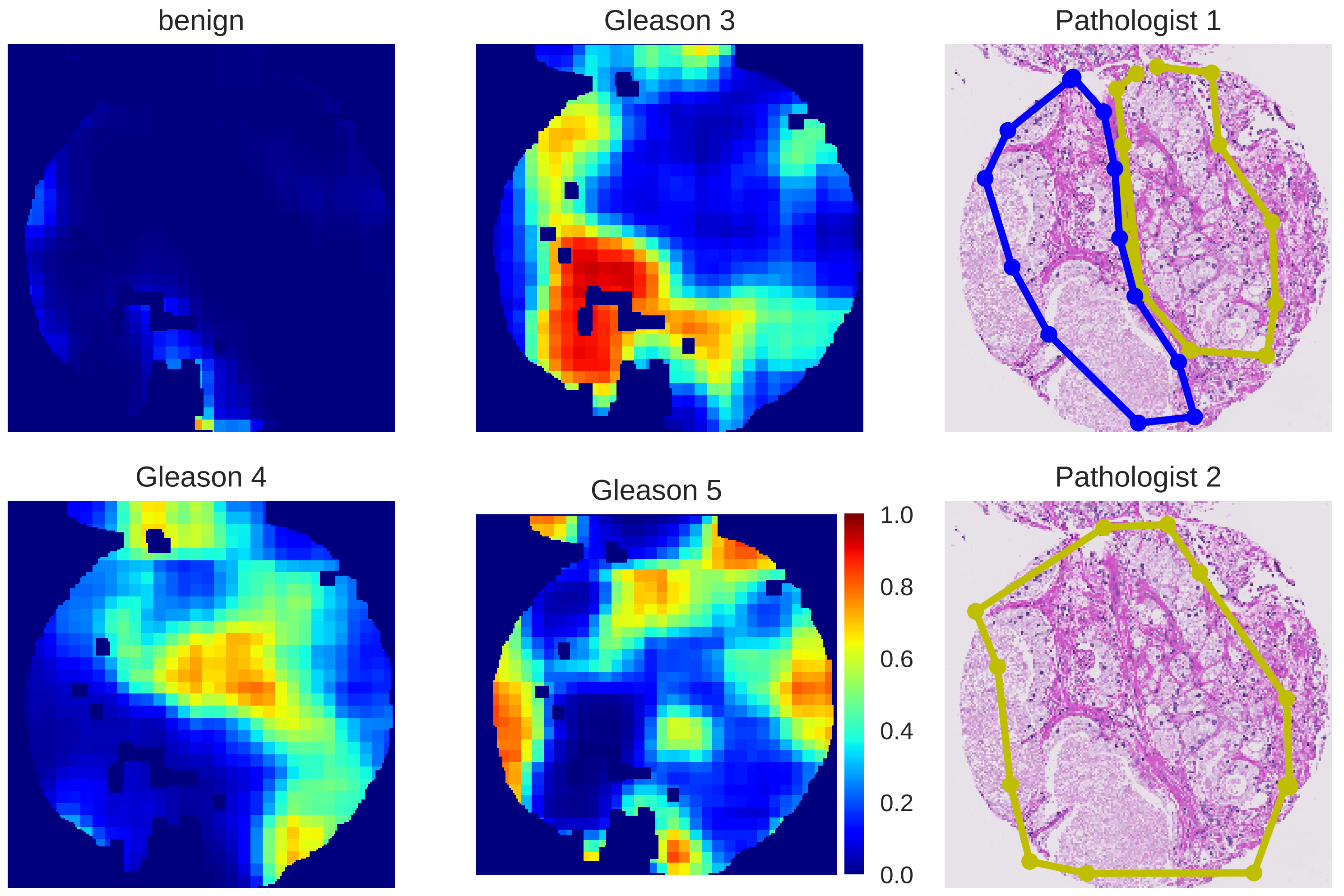

benign
Gleason 3
Pathologist 1
Gleason 4
Gleason 5
Pathologist 2
1.0
0.8
0.6
0.4
0.2
0.0

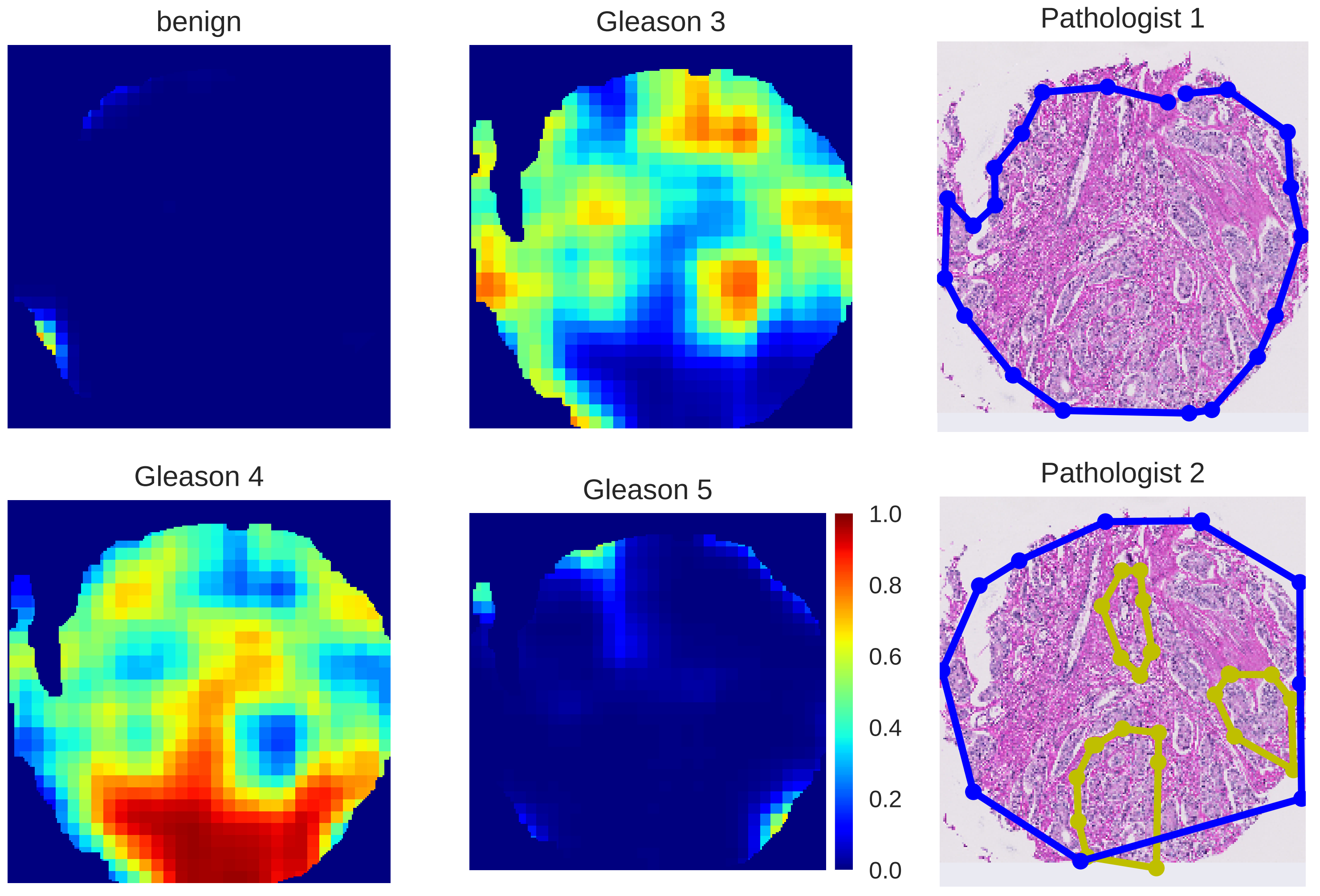

benign
Gleason 3
Pathologist 1
Gleason 4
Gleason 5
Pathologist 2
1.0
0.8
0.6
0.4
0.2
0.0

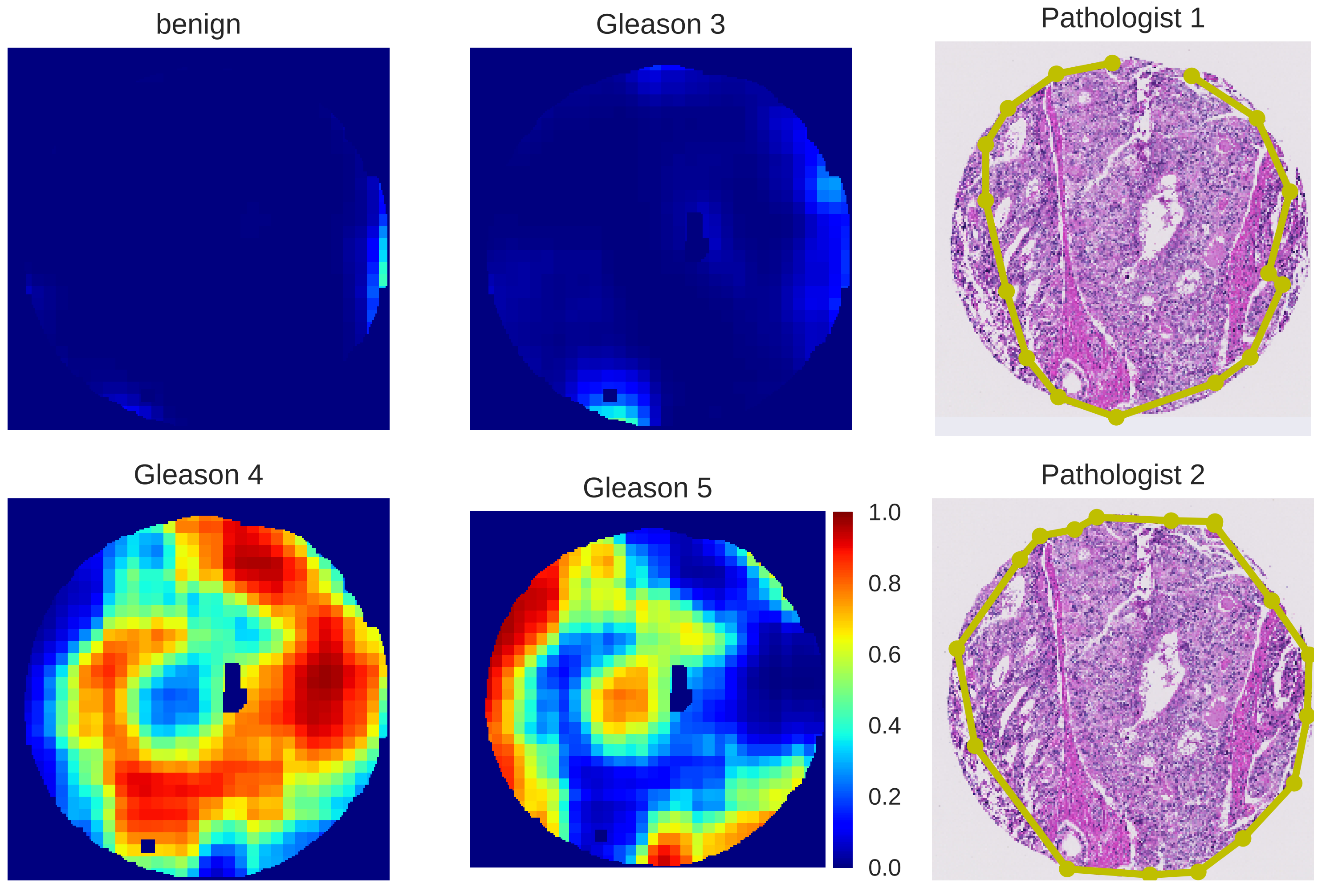

benign
Gleason 3
Pathologist 1
Gleason 4
Gleason 5
Pathologist 2
1.0
0.8
0.6
0.4
0.2
0.0
